# DNA copy number analysis of Grade II–III and Grade IV gliomas reveals differences in molecular ontogeny including chromothripsis associated with *IDH* mutation status

**DOI:** 10.1186/s40478-015-0213-3

**Published:** 2015-06-20

**Authors:** Adam Cohen, Mariko Sato, Kenneth Aldape, Clinton C. Mason, Kristin Alfaro-Munoz, Lindsey Heathcock, Sarah T. South, Lisa M. Abegglen, Joshua D. Schiffman, Howard Colman

**Affiliations:** Huntsman Cancer Institute, University of Utah, 2000 Circle of Hope, Salt Lake City, UT 84112 USA; Children’s Hospital, University of Iowa, 200 Hawkins Dr, 2524 JCP, Iowa City, IA 52242 USA; Department of Pathology, MD Anderson Cancer Center, Houston, TX 77030 USA; ARUP Laboratories, Salt Lake City, UT 84112 USA; Department of Pediatrics, University of Utah, Salt Lake City, UT 84112 USA; Department of Pathology, University of Utah, Salt Lake City, UT 84112 USA

**Keywords:** Isocitrate dehydrogenase, Glioma, Glioblastoma, Copy number alterations, Chromothripsis

## Abstract

**Introduction:**

Isocitrate dehydrogenase (*IDH*) mutation status and grade define subgroups of diffuse gliomas differing based on age, tumor location, presentation, and prognosis. While some biologic differences between *IDH* mutated (*IDH*^mut^) and wild-type (*IDH*^wt^) gliomas are clear, the distinct alterations associated with progression of the two subtypes to glioblastoma (GBM, Grade IV) have not been well described. We analyzed copy number alterations (CNAs) across grades (Grade II–III and GBM) in both *IDH*^mut^ and *IDH*^wt^ infiltrating gliomas using molecular inversion probe arrays.

**Results:**

Ninety four patient samples were divided into four groups: Grade II–III *IDH*^wt^ (*n* = 17), Grade II–III *IDH*^mut^ (*n* = 28), GBM *IDH*^wt^ (*n* = 25), and GBM *IDH*^mut^ (*n* = 24). We validated prior observations that *IDH*^wt^ GBM have a high frequency of chromosome 7 gain (including *EGFR*) and chromosome 10 loss (including *PTEN*) compared with *IDH*^mut^ GBM. Hierarchical clustering of *IDH*^mut^ gliomas demonstrated distinct CNA patterns distinguishing lower grade gliomas versus GBM. However, similar hierarchical clustering of *IDH*^wt^ gliomas demonstrated no CNA distinction between lower grade glioma and GBM. Functional analyses showed that *IDH*^wt^ gliomas had more chromosome gains in regions containing receptor tyrosine kinase pathways. In contrast, *IDH*^mut^ gliomas more commonly demonstrated amplification of cyclins and cyclin dependent kinase genes. One of the most common alterations associated with transformation of lower grade to GBM *IDH*^mut^ gliomas was the loss of chromosomal regions surrounding *PTEN. IDH*^mut^ GBM tumors demonstrated significantly higher levels of overall CNAs compared to lower grade *IDH*^mut^ tumors and all grades of *IDH*^wt^ tumors, and *IDH*^mut^ GBMs also demonstrated significant increase in incidence of chromothripsis.

**Conclusions:**

Taken together, these analyses demonstrate distinct molecular ontogeny between *IDH*^wt^ and *IDH*^mut^ gliomas. Our data also support the novel findings that malignant progression of *IDH*^mut^ gliomas to GBM involves increased genomic instability and genomic catastrophe, while *IDH*^wt^ lower grade tumors are virtually identical to GBMs at the level of DNA copy number alterations.

**Electronic supplementary material:**

The online version of this article (doi:10.1186/s40478-015-0213-3) contains supplementary material, which is available to authorized users.

## Introduction

Gliomas are the most frequent primary malignant brain tumors with an annual incidence of approximately 20,000 cases in the United States [9]. Glioblastoma (GBM) is the most common glioma and remains nearly uniformly fatal, with a median survival under 16 months in aggressively treated patients [17]. While these tumors are currently diagnosed by histopathology alone and generally treated based on histology and grade, recent findings identifying distinct molecular subgroups within these tumor types strongly suggest that improving treatments and patient survival will require detailed understanding of the biological and clinical differences between these subgroups.

Histopathologically, diffuse gliomas are categorized according to the WHO by histology (Astrocytoma, Oligodendroglioma, or Oligoastrocytoma) and grade [lower grade (grade II/III) versus glioblastoma (GBM, grade IV)]. Clinically, GBMs have been classified as primary or secondary on the basis of clinical presentation [34]. Secondary GBMs, which are more common in young adults, display evidence of progression from a lower-grade tumor, whereas primary GBMs, which are more common in older adults, present as advanced cancers at diagnosis [28]. Recently, large scale efforts have been made to identify the major genetic and epigenetic alterations and to define important molecular subtypes in GBM and lower grade gliomas [30, 43, 41]. The strongest prognostic factor for all glioma histologies is mutation in one of the isocitrate dehydrogenase genes (*IDH1* or *IDH2*) [48], and mutation of these genes is seen at higher frequencies in lower grade gliomas and secondary GBMs.

Chromosome abnormalities in gliomas have been associated with various subgroups. A summary of over 400 GBMs showed gains in *EGFR* (7p12) in 30 %, *GLI/CDK4/MDM2* (12q13-14) in 13 %, *PIK3C2B/MDM4* (1q32) in 8 %, *PDGFRA* (4q12) in 8 %, and *MET* (7q31) in 4 % with deletions in *CDKN2A/2B* (9p21) in 47 %, *PTEN* (10q23) in 10 %, and *RB1* (13q14) in 6 % [31]. Primary GBMs commonly have gains in chromosome 7 and 19 and loss of chromosome 10 [31, 22, 25, 26, 46, 2]. Secondary or *IDH* mutated glioblastomas are less likely to have the above alterations and more likely to have gains in 8q and 10q accompanying simpler karyotypes [27, 22, 20, 25]. Grade II astrocytomas have been less well studied, with gains in 7q described in two studies [7, 12], and other alterations not replicated [7, 12, 46]. Although some groups have found worse prognosis in GBMs with either *EGFR* or chromosome 7 amplifications [15, 6, 26, 13], others, including the largest study (*n* = 532), found no association with outcome [14, 46, 13]. One reason for this inconsistency may be confounding due to the association of *EGFR* amplification with other known prognostic factors, such as age, G-CIMP status, or IDH mutation status [8, 3, 27, 22, 25, 20]. Only one study has looked at the prognosis of copy nuber alterations (CNAs) within subgroups defined by IDH status, suggesting that chromosome 7p gain and *TP53* loss are prognostic in grade III gliomas with IDH mutation [37].

Gliomas have chromosomal instability, with a propensity for recurring patterns of CNAs [21, 36]. Although multiple mechanisms may be responsible for CNAs in gliomas, chromothripsis is a recently described form of localized CNA due to chromosomal catastrophe that may occur commonly in gliomas [18, 23]. Chromothripsis, which literally means “chromosome shattering,” can be identified from CNA technologies such as SNP microarrays. The association of chromothripsis with clinical factors and prognosis in gliomas has not been explored to date.

The purpose of this study is to determine CNAs within glioma subgroups defined by grade and IDH status. We deliberately chose to maximize the percent of *IDH*^mut^ grade IV gliomas and *IDH*^wt^ lower grade gliomas, as these are rare in most other studies. In addition, we examine prognostic CNAs within each glioma subgroup and chromothripsis as a function of grade and IDH status.

## Materials and methods

### Samples and nucleic acid extraction

We analyzed formalin-fixed, paraffin-embedded (FFPE) glioma specimens from 94 patients from M.D. Anderson Cancer Center (Houston, TX) and 20 autopsied normal brains (controls) from Huntsman Cancer Institute, University of Utah (Salt Lake City, UT). IRB approval was obtained from each institution. *IDH* mutation status was confirmed using direct sequencing [42]. Gliomas were categorized by a single neuropathologist (KA) as either high grade (GBM) or lower grade (grade II or III). DNA was isolated using the Recoverall Total Nucleic Acid Isolation kit (AM1975, Applied Biosystems/Ambion, Austin, TX) and quantified with a high sensitivity, double strand specific, nucleic acid, fluorescent stain (PicoGreen, P7589, Invitrogen, Carlsbad, CA).

#### Copy number analysis

The DNA was plated in a 96-well plate with concentration goal of 7.5 ng/ul in a total volume of 40ul (300 ng total). The completed plates were sent to the Affymetrix Research Services Laboratory at Santa Clara, CA, and the OncoScan™ FFPE Express MIP assay was run as previously described [45, 35, 44]. The raw MIP data from the completed assay was loaded into Nexus Copy Number (BioDiscovery, Inc., El Segundo, CA). Stringency cutoffs for probe performance included call rates ≥90 % and standard deviations ≤0.3. BioDiscovery’s SNP-Rank Segmentation algorithm with Quadratic Wave Correction, a statistically based algorithm similar to Circular Binary Segmentation (CBS), was used to make copy number and loss of heterozygosity (LOH) calls [29]. The significance threshold for segmentation was set at 5.0E-7 and required a minimum of five probes per segment. CNA thresholds were based on sample mosaicism, and set at 0.4 and −0.4 units of copy number from diploidy. High gain and homozygous loss were denoted by 1.2 and −1.2 units of copy number from diploidy. Genes were assigned to regions using the NCBI36/hg18 genome assembly on the UCSC genome browser. Gene gain was considered based on the copy number for that gene, without regard for entire chromosome gain or loss.

#### Statistics

In order to assess the significance of the genomic alterations, Genomic Identification of Significant Targets in Cancer (GISTIC) was used to define deletions and gains and to calculate the *q*-value [40, 39], taking into account the frequency, amplitude and focality of the observed gains and deletions. CNAs with *q* < 0.25 were considered significant. Univariate and multivariate Cox proportional hazards models were fit to the data using Cox regression in SAS 9.3. Multivariable models were built using backward stepwise regression from a model including all variables with *p* < 0.1 in univariate analysis and maintaining variables with *p* < 0.05 in the multivariate analysis. Hazard ratios and *p*-values from the associated log-rank tests were reported. *P*-values for copy number alterations were adjusted using the Benjamini & Hochberg step-up false discovery rate (FDR) controlling procedure [1]. The adjustment was done separately for each analysis. Hierarchical clustering was done using complete linkage disregarding the sex chromosomes. We also used gene ontology analysis to identify altered pathways within and between glioma subgroups using the ToppGene system [5]. We disregarded pathways determined by genes grouped together on a single chromosome and, thus, affected as a single group by large CNAs. The heatmap in Fig. 3d was generated using the HeatMapImage module from Genepattern using the default color scheme [33]. Fisher’s exact test was used for contingency table analyses.

## Results

### Population

**Fig. 1:**
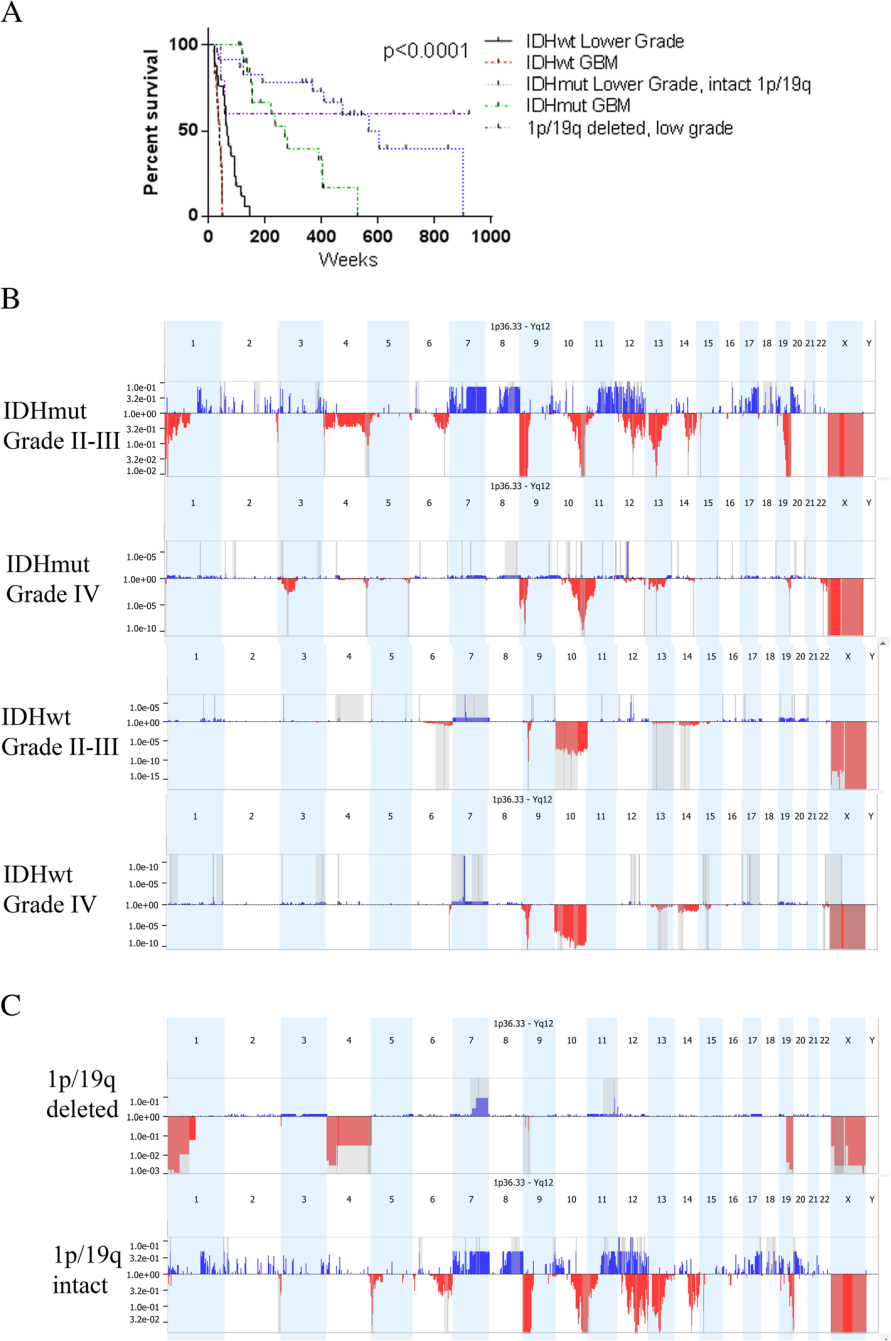
**a**. Kaplan-Meier curves of five groups of gliomas determined by grade, *IDH* mutation status, and 1p/19q deletion. All 94 patients were included. **b**. Chromosome maps from GISTIC analysis of four subgroups of gliomas defined by *IDH* status and grade. The y-axis gives GISTIC *q*-values. *Red* indicates deletions and *blue* indicates gains. **c**. Chromosome maps from GISTIC analysis for grade II–III *IDH* mutated gliomas separated by 1p/19q status

The cohort included a total of 94 diffuse gliomas: 17 Grade II–III IDH1 wild type (IDH1^wt^), 28 Grade II–III IDH1 mutant (IDH1^mut^), 25 Grade IV (glioblastoma, GBM) IDH1^wt^, and 24 Grade IV IDH1^mut^. Thirty-four patients (36 %) were female, and sixty (64 %) were male. The median survival for the population as a whole was 112 weeks, comparable to previously reported survival data. As expected, tumor grade (HR = 2.2, *p* = 0.003) and *IDH* status (HR = 26.7, *p* < 0.0001) were independent predictors of survival (Fig. 1a). The median survival was 37.4 weeks for patients with *IDH*^wt^ GBM, 65.4 weeks for patients with *IDH*^wt^ Grade II–III gliomas, 270.3 weeks for patients with *IDH*^mut^ GBM, and 604.3 weeks for patients with *IDH*^mut^ Grade II–III tumors (Fig. 1a). Indeed, IDH mutation status was a stronger prognostic factor than grade, as IDH^wt^ lower grade gliomas had a worse prognosis than IDH^mut^ grade IV gliomas, a finding previously observed in independent datasets [48, 11].

### Copy number alterations (CNAs) by subgroup

CNAs identified as significant within each of the four clinical/molecular subgroups using GISTIC *q*-values for CNAs are shown in Fig. 1b Due to their distinct chromosomal abnormalities and clinical characteristics, the lower grade oligodendroglial tumors with 1p/19q co-deletion (*n* = 5) were analyzed separately (Fig. 1a,c). A complete list of GISTIC significant CNAs in each subgroup defined by IDH status and grade is given in Additional file 1: Table S1.

**Fig. 2:**
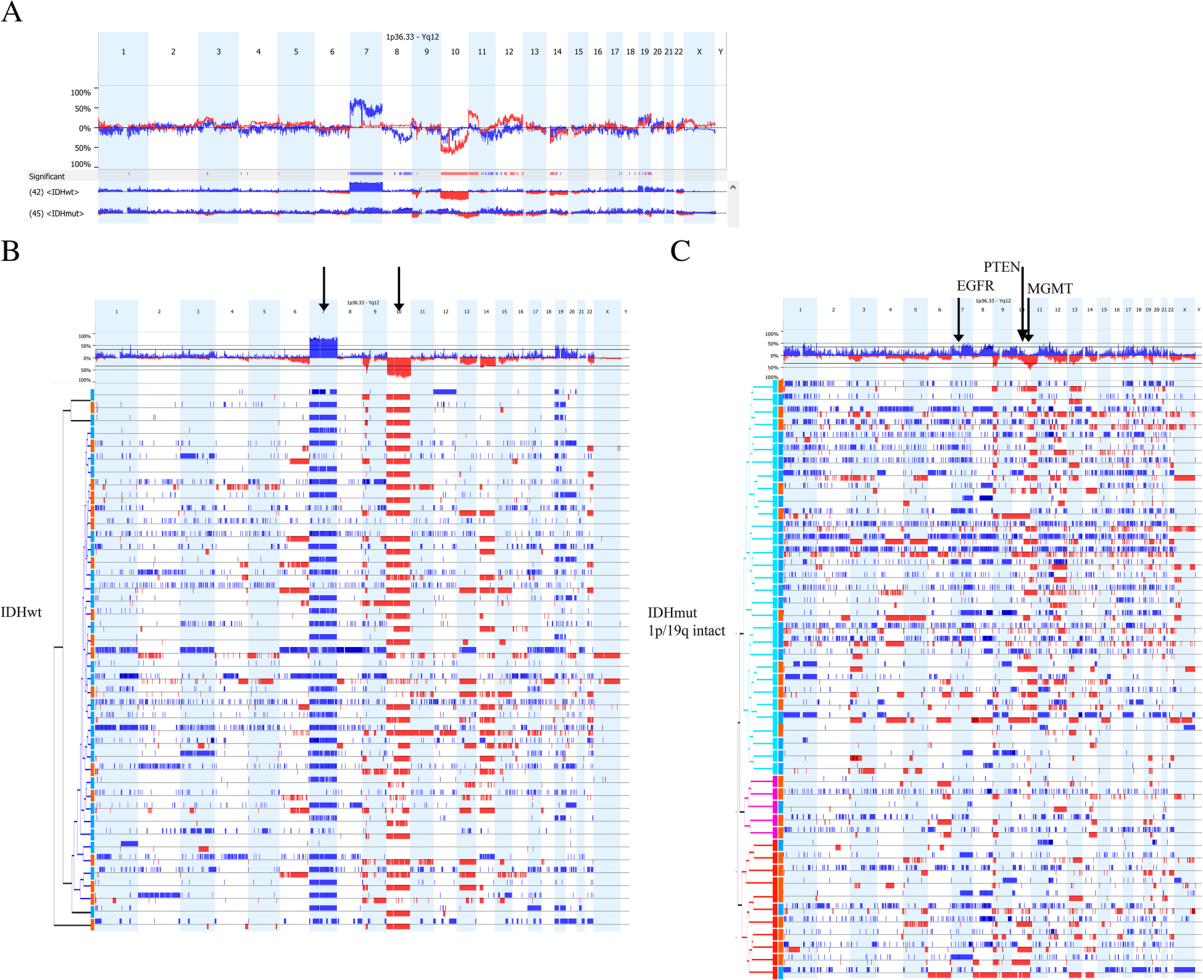
In all chromosome maps, chromosomes are along the x-axis. The y-axis gives the percent of samples with deletion (*red*) or gain (*blue*) at that locus. For individual samples,chromosome abnormality calls are shown. **a**. Comparison of *IDH* wild type gliomas and *IDH* mutated gliomas. For each type of glioma, the chromosome map is shown. The *top graph* indicates the difference in the percent of samples with gains (*blue*) and deletions (*red*) at each locus between the two groups. Up means more common in *IDH* wild type gliomas and down means more common in *IDH* mutated gliomas. **b**. Hierarchical clustering of *IDH* wild type gliomas is shown below a chromosome map of all *IDH* wildtype gliomas. Grade is indicated by the *rectangles* next to the hierarchy tree with *blue* indicating grade IV and *orange* indicating lower grade. **c**. Hierarchical clustering for 1p/19q non-co-deleted, *IDH* mutated gliomas is shown below a chromosome map of all1p/19q non-co-deleted, *IDH* mutated gliomas. Grade is indicated by the *rectangles* next to the hierarchy tree with *blue* indicating grade IV and *orange* indicating lower grade

On a global scale, different patterns of CNA were seen in *IDH*^mut^ and *IDH*^wt^ gliomas. Within *IDH*^wt^ gliomas, the significant CNAs observed in lower grade and GBM tumors were generally very similar, including gain of entire copies of chromosome 7, loss of entire copies of chromosome 10, and focal losses at chromosome 9 around the *CDKN2A/CDKN2B* locus. On the other hand, *IDH*^mut^ lower grade gliomas and GBMs demonstrate distinct CNAs associated with grade (described further below). Copy number differences between *IDH*^wt^ gliomas and *IDH*^mut^ gliomas, regardless of grade, are listed in Additional file 2: Table S2 and shown in Fig. 2a.

### *IDH*^wt^ gliomas are similar regardless of grade

To examine molecular subgroups within tumors separated by *IDH* and 1p/19q status, we used unsupervised hierarchical clustering. In *IDH*^wt^ gliomas, lower grade and grade IV gliomas clustered together in one large top level cluster (Fig. 2b), indicating that lower and high grade *IDH*^wt^ tumors share similar CNA alterations. The CNA alterations seen most frequently across all grades of *IDH*^wt^ gliomas were broad gain of chromosome 7 and loss of chromosome 10 (Arrows, Fig. 2b). This pattern of large CNAs in *IDH*^*wt*^ gliomas contrasts with *IDH*^mut^ gliomas, in which changes on chromosomes 7 and 10 were either absent or more focal around the *EGFR*, *MGMT*, and/or *PTEN* genes (Arrows, Fig. 2c).

**Fig. 3:**
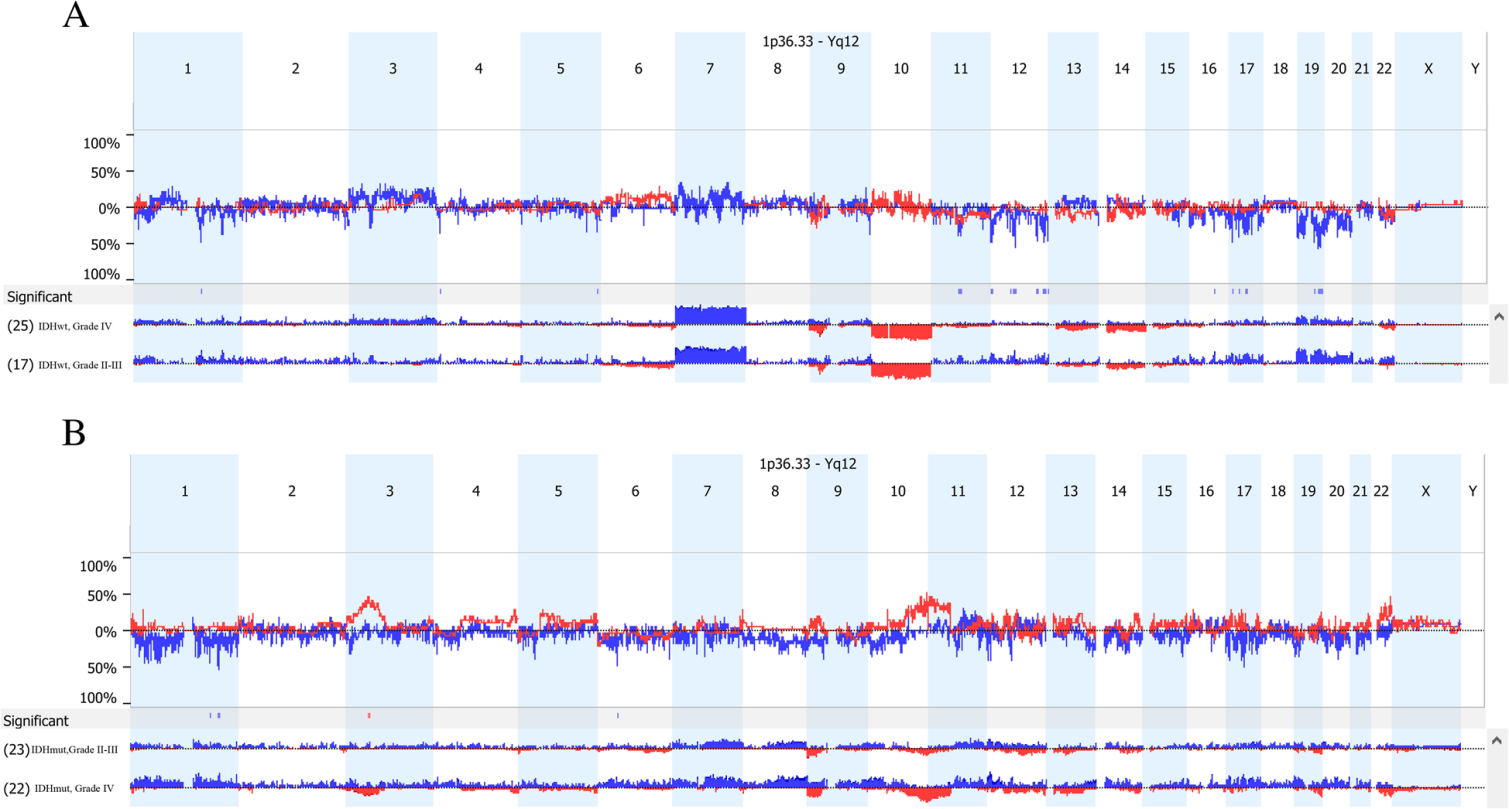
**a**. Comparison of *IDH* wild type lower grade and grade IV gliomas. For each type of glioma, the chromosome map is shown. The *top graph* indicates the difference in the percent of samples with gains (*blue*) and deletions (*red*) at each locus between the two groups. Up means more common in lower grade gliomas and down means more common in grade IV gliomas. **b**. Comparison of *IDH* mutated, 1p/19q non-co-deleted lower grade and grade IV gliomas. For each type of glioma, the chromosome map is shown. The *top graph* indicates the difference in the percent of samples with gains (*blue*) and deletions (*red*) at each locus between the two groups. Up means more common in lower grade gliomas and down means more common in grade IV gliomas

Despite the similarity between *IDH*^wt^ lower grade versus grade IV gliomas on clustering analysis, there were a few chromosome areas with significant differences between the two grades (Additional file 3: Table S3 and Fig. 3a). Interestingly, there were no CNAs that were more common in high grade *IDH*^wt^ gliomas than in lower grade *IDH*^wt^ gliomas. Rather, there were several chromosomal regions, which are listed in Additional file 3: Table S3, that were less likely to be gained in grade IV (*IDH*^wt^) gliomas than in lower grade *IDH*^mut^ gliomas. Many of these regions contain tumor suppressor genes such as *TP53* or *XRCC1*, as well as putative proto-oncogenes *BCL3*, *CDK4*, and *HIF3A*. The fact that no CNAs were more common in high grade *IDH*^wt^ gliomas than in lower grade *IDH*^wt^ gliomas supports the concept that the recurring copy number aberrations seen in *IDH*^wt^ GBM are likely to be present in grade II–III precursor tumors. Alternatively, the data are consistent with the possibility that subclones from the lower grade *IDH*^wt^ tumors can progress into grade IV gliomas (Additional file 3: Table S3 and Fig. 3a).

Using functional gene ontology analysis to identify relevant pathways associated with significant CNAs, we found that *IDH*^wt^ lower grade gliomas were enriched for alterations in pathways involving RB/checkpoint signaling, kinase binding, PI3K/AKT signaling, and cell cycle control. We also identified the pathways enriched in CNAs that differed between lower and high grade *IDH*^wt^ gliomas. These pathways included base excision repair, telomerase extension, nucleotide excision repair, and repair of abasic sites, suggesting a small window of sensitivity may exist to DNA damaging agents early in *IDH*^wt^ GBM development.

### Progression to grade IV in *IDH*^mut^ gliomas involves losses on chromosome 10 and increased chromosome instability

Among 1p/19q non-co-deleted *IDH*^mut^ gliomas, unsupervised clustering identified two major clusters with significantly different percent of high and lower grade gliomas in each cluster (*p* = 0.018). One large cluster included 83 % (20) of the *IDH*^mut^ GBMs but only 48 % (11) of the lower grade *IDH*^mut^ gliomas. The other predominant cluster contained 35 % (8) of the lower grade *IDH*^mut^ gliomas and 12 % (3) of the *IDH*^*mut*^ GBMs. A third smaller cluster contained one GBM and four lower grade gliomas (Fig. 2c). The most significant difference (*P* = 5 × 10^−5^) between the two largest cluster*s* was loss of the terminal end of the q arm of chromosome 10 including *MGMT*, which occurred in 80 % of the cluster with most of the GBMs and 9 % of the cluster with primarily lower grade gliomas (Additional file 4: Table S4). Loss of *PTEN*, which is more proximal on chromosome 10, was also associated with the two largest clusters, although not as tightly. Thus, it is not clear if the important gene on chromosome 10 is *PTEN* or *MGMT* or both.

Grade IV IDH^mut^ gliomas are considered to be secondary GBMs that have progressed from lower grade gliomas. Therefore, differences between lower grade and grade IV *IDH*^mut^ gliomas may indicate genes or pathways that are important for progression of IDH^mut^ gliomas. In addition to the losses in 10q indicated above, grade IV *IDH*^mut^ gliomas were more likely to have gains of 1q25.3 (*SMG7*, *NCF2*), 1q32.1 (*KIF14*, *DDX59*, *BTG2*), 6p21.1 (*HSP90AB1* and other genes) and loss of 3p21 (multiple genes). A broad loss of heterozygosity (LOH) on 11p15 was also more common in the grade IV gliomas (Additional file 5: Table S5 and Fig. 3b). Applying functional gene ontology analysis to genes on these chromosome segments, the only enriched pathway was nitrogen compound transport (Additional file 6: Table S6). Both lower grade and grade IV *IDH*^mut^ gliomas were enriched foralterations in the PI3K/AKT pathway. However, only *IDH*^mut^ grade IV gliomas were enriched for alterations in pathways involving regulation of actin cytoskeleton, RAS, and EGFR. These differences suggest that RAS signaling and cytoskeletal abnormalities may play a role in progression of *IDH*^mut^ gliomas.

### Increased genomic instability is observed in *IDH*^mut^ gliomas

**Fig. 4:**
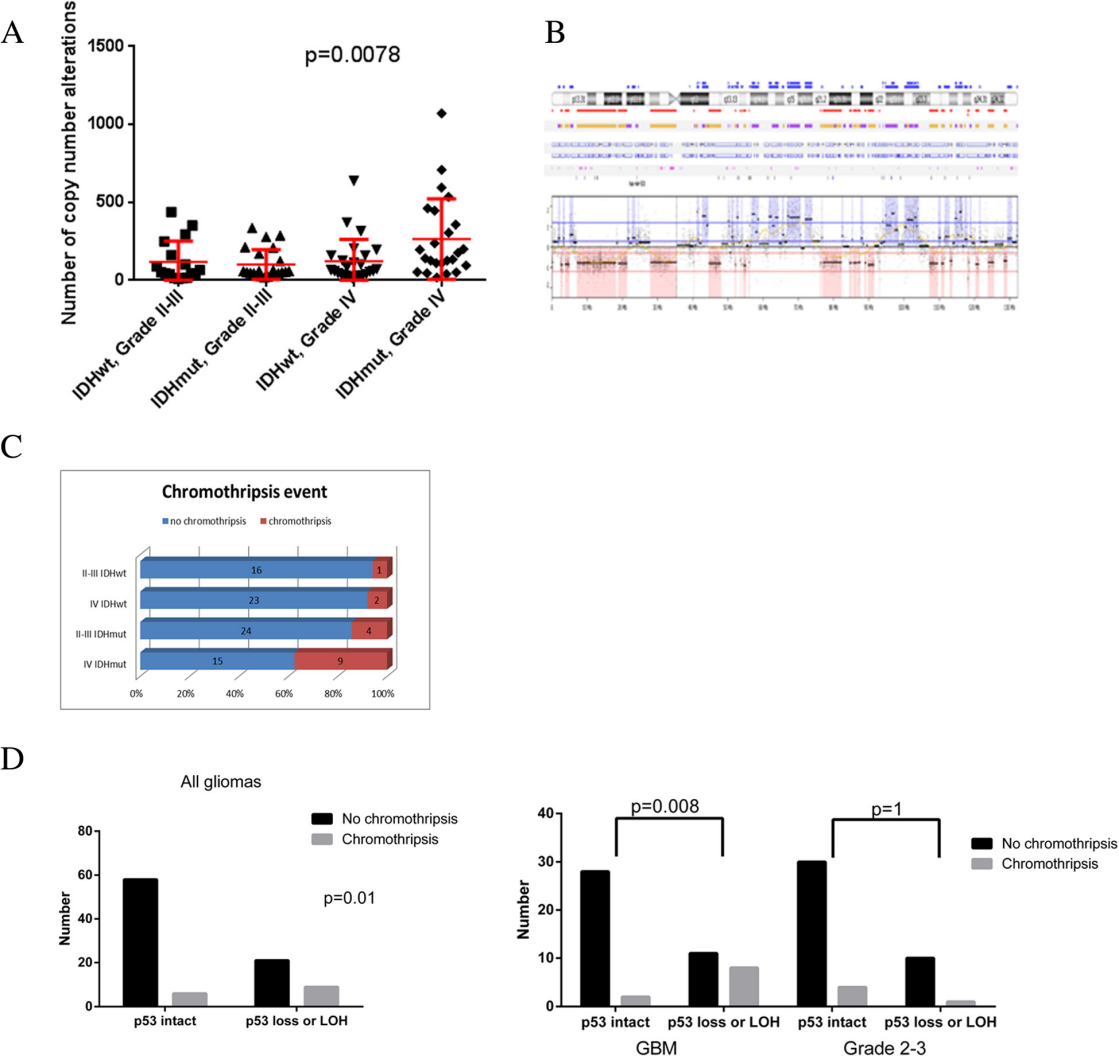
**a**. Scatter plot of the number of copy number alterations in the five groups of gliomas. *Horizontal bar* indicates mean with 95 % confidence interval shown. **b**. Example of a chromosome from one of the glioma samples with chromothripsis. **c**. Bar graph of the frequency of chromothripsis in each group of gliomas. **d**. Association of chromothripsis and p53 alterations in all glioma samples and stratified by grade

We observed a mean number of gains and losses of 150 CNA/sample (range 11–1070) in all samples. Overall, Grade IV tumors had higher CNA frequency than lower grade tumors. Unexpectedly, the highest frequency of alterations was seen in *IDH*^mut^ grade IV gliomas. Grade IV *IDH*^mut^ gliomas had more than double th number of CNA than any of the other three groups (*p* = 0.0078 by ANOVA, with pairwise *p*-values <0.008 for all three pairs, Fig. 4a). Although the absolute number of chromosome abnormalities can change based on analysis threshold parameters and our analysis was designed to minimize undercalling, the differences between groups were not affected by varying thresholds. These findings suggest that increasing chromosome instability is a hallmark of the progression of *IDH*^mut^ lower grade gliomas into high grade. Whether this chromosome instability is a cause or effect of increasing grade cannot be determined from our data.

We also examined the TCGA GBM and lower grade glioma datasets for total number of copy number alterations. Significantly more copy number alterations were seen in both the *IDH*^mut^ Grade IV (mean 132.1, median 105 per sample) and *IDH*^wt^ Grade IV (mean 132.8, median 96.5 per sample) tumors compared to the lower grade *IDH*^mut^ (mean 63.04, median 53 per sample) and *IDH*^wt^ (mean 53.9, median 40 per sample) gliomas. However, due to the small number of *IDH*^mut^ GBM with copy number data (17), the power for comparing the number of CNA between *IDH*^mut^ and *IDH*^wt^ GBM was low. Moreover, unlike our samples, the lower grade and grade IV samples in the TCGA were run separately, so batch effects are possible.

Given the subgroup differences in CNA frequency, we examined the specific patterns of alterations across the whole genome and looked within groups at the specific CNAs on chromosomes with a high number of alterations. The term chromothripsis describes situations in which there are a large number of chromosomal rearrangements over localized chromosomal regions [10, 38]. In our analysis, we used the definition of at least 10 switches between two copy-number states (gain and loss) on at least one individual chromosome for a tumor to be considered to have chromothripsis. An example of a chromosome with chromothripsis is shown in Fig. 4b. By this definition, 11 of our samples contained chromothripsis. Chromothripsis was significantly more common in *IDH*^mut^ Grade IV tumors than *IDH*^wt^ (*p* = 0.002) or lower grade *IDH*^mut^ (*p* = 0.05, Fig. 4c).

We hypothesized that loss of function of p53 would predispose to chromothripsis because of the inability of p53 deficient cells to undergo apoptosis in the face of chromosome shattering. Indeed, gliomas with chromosome loss at the *TP53* locus or LOH at the *TP53* locus were more likely to have chromothripsis than those with no alteration of *TP53* (Fig. 4d), although this relationship was limited to Grade IV tumors.

The prognostic significance of chromothripsis is unknown. In our cohort, chromothripsis was not prognostic.

### Alterations in cancer associated genes reveal the biological differences between molecular subtypes

**Fig. 5:**
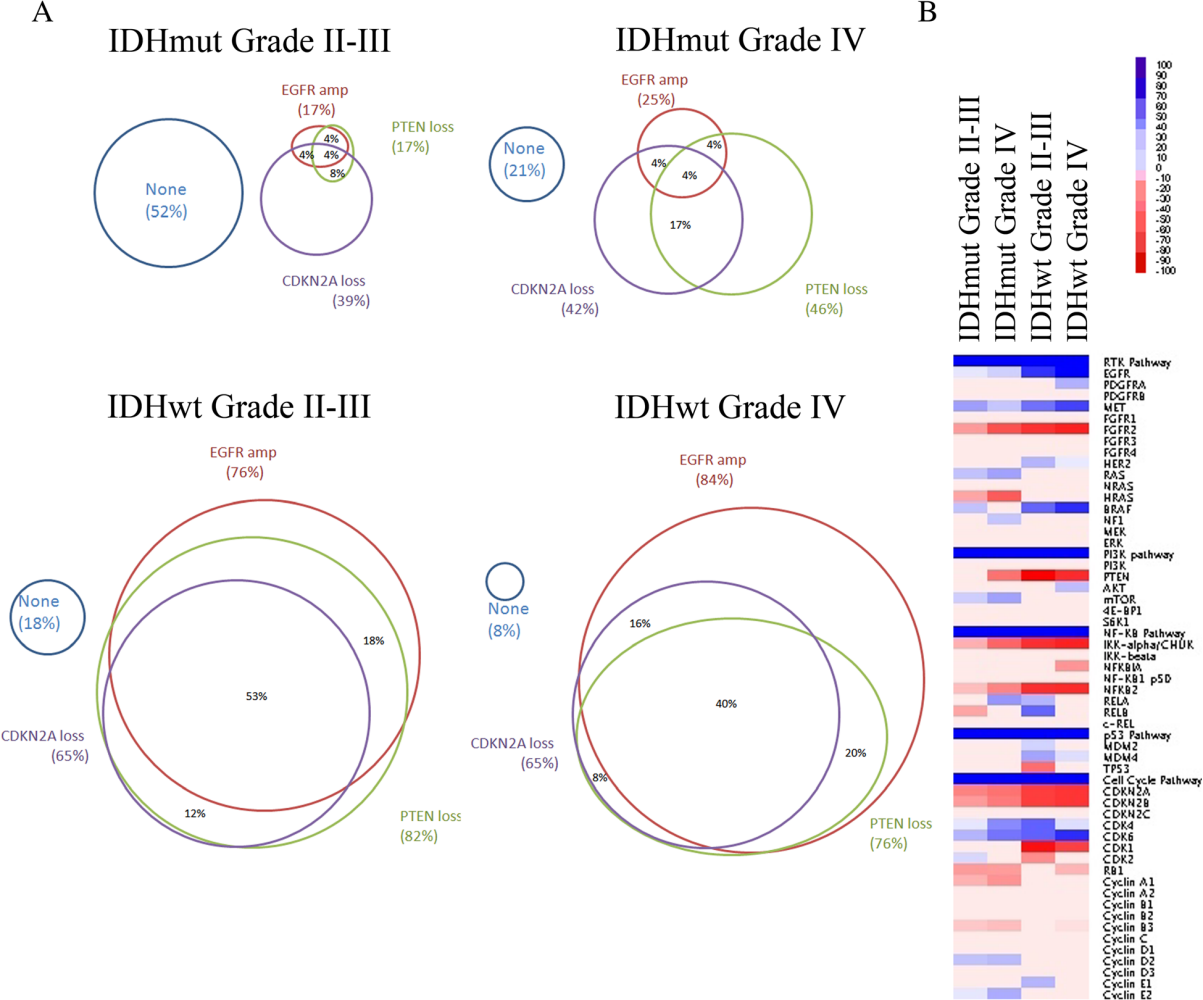
**a**. Venn diagrams of the percent of tumors in each of the 1p/19q non-co-deleted glioma groups with gain of *EGFR*, *PTEN* loss, and/or *CDKN2A* loss. Percents are given for intersecting regions. The diameter of each circle is proportional to the percent of tumors in each subgroup with a CNA affecting the gene. **b**. Heatmap of known glioma-associated genes and pathways in each of the four 1p/19q non-co-deleted groups of gliomas. Only chromosome abnormalities significant by GISTIC were included. *Blue* indicates gain and *red* indicates loss. The strength of the color indicates the percent of tumors with that alteration

To illustrate the similarities and differences between the four subgroups of 1p/19q non-co-deleted gliomas, we examined the pattern of alterations of three well-described glioma associated genes. We used Venn diagrams to visualize patterns of CNAs in the oncogene *EGFR* (7p11.2) and the tumor suppressor genes *CDKN2A* (9p21.3) and *PTEN* (10q23.31) (Although our data cannot determine whether CNAs affecting these genes are functionally targeting these genes or nearby ones, these are genes with known functional significance in gliomas.). For this analysis, we only included CNAs that affected the whole gene, (6 % of samples had losses within CDKN2A or PTEN and 11 % of samples had gains within EGFR that did not affect the whole gene) (Fig. 5a).

Gain of *EGFR* and loss of *PTEN* and *CDKN2A* occur together frequently in both *IDH*^wt^ lower grade and grade IV gliomas (all three occurring together in 53 % and 40 %, respectively), with no significant differences of these alterations by grade. On the other hand, *EGFR* gain is significantly rarer overall in *IDH*^mut^ gliomas (17 % grade II–III, 25 % grade IV) and *CDKN2A* loss is slightly lower (39 % grade II–III, 42 % grade IV) compared with *IDH*^wt^. Moreover, seeing all three alterations is very rare in *IDH*^mut^ gliomas, only occurring in 4 % regardless of grade. We did observe significant differences in *PTEN* loss associated with grade in *IDH*^mut^ tumors (17 % *IDH*^mut^ grade II–III and 46 % *IDH*^mut^ grade IV [*p* = 0.025]). These findings suggest that loss of *PTEN* or genes near it on chromosome 10q may be a key and unique factor associated with progression of *IDH*^mut^ tumors to grade IV.

To examine the functional significance of the chromosome alterations seen in the different groups, we examined a predetermined list of genes in pathways previously shown to be altered and functionally important in gliomas, including receptor tyrosine kinases (RTK), phosphatidyl-inositol-3-kinase, NF-κB, P53, and cell cycle regulators (Fig. 5b). These genes were considered altered if they were in an extended region identified by GISTIC analysis as having a *q*-value <0.25. Although most gliomas show alterations in all of these pathways, the mechanism by which the pathways are altered can differ. *IDH*^wt^ gliomas had significantly more chromosome alterations affecting RTK signaling than *IDH*^mut^ gliomas. PI3K pathway activation also differed based on *IDH* status: upstream changes such as *PTEN* deletion or *AKT* gain were more common in *IDH*^wt^ gliomas and *MTOR* gain was significantly less common (*p* = 2 × 10^−7^, 0.002, and 1 × 10^−5^, respectively). Such differences could have implications for application of multiple targeted treatments to these glioma subtypes. Among cell cycle regulators, *IDH*^wt^ gliomas were significantly more likely to have CDK1 loss and less likely to have cyclin A1 gene loss or cyclin D1 or E2 gene gain.

### Prognostic factors

The strongest prognostic factors in the whole population were *IDH* status and grade (Fig. 1c). In multivariate analysis, the other significant variables were loss of the estrogen receptor B (ESR2), gain of *CDKN1C*, and *TP53* loss, each of which was a negative prognostic factor (Additional file 7: Table S7). Given the biologic and clinical differences between the four subgroups defined by *IDH* status and grade, we sought to identify distinct prognostic factors within each subgroup and within the entire *IDH*^mut^ and *IDH*^*wt*^ groups. Although univariate analysis identified distinct copy number alterations in each subgroup that were significantly associated with survival in our cohort, none were significantly associated with survival when we attempted to validate them using 433 GBM and 181 lower grade glioma samples from the TCGA obtained via Nexus premier.

## Discussion

We present one of the first comparative analyses of CNAs among glioma subgroups defined by WHO grade and *IDH* mutation status. Confirming prior observations, we observe significant chromosomal differences between *IDH*-mutant and *IDH*-wild type tumors. When analyzing subgroups by grade and mutation status, we find few significant copy number differences between *IDH*^wt^ lower grade and *IDH*^wt^ grade IV gliomas. These genomic similarities support the concept that despite their histologic appearance, biologically these lower grade *IDH*^wt^ tumors are pre-glioblastomas with a median survival a mere 7 months longer than grade IV gliomas, with few long-term survivors [24]. In contrast, among *IDH*^mut^ tumors, clustering based on copy number demonstrates that lower grade and grade IV gliomas with *IDH* mutations are distinct biologic entities; they also have distinct prognosis. The progression of *IDH*^mut^ gliomas from lower grade to grade IV involves multiple CNAs, particularly on chromosome 10q, affecting biologically relevant pathways including: activation of PI3K signaling through loss of *PTEN* and gain of mTOR, as well as activation of cell cycle signaling through gain of CDK4, CDK6, and cyclinE2. *MGMT* loss may play a role as well, consistent with the resistance of *MGMT* unmethylated^t^ gliomas to alkylating agents.

In comparison to *IDH*^*mut*^ gliomas, *IDH*^wt^ gliomas have greater activation of receptor tyrosine kinase signaling through *EGFR* gain, *MET* gain, and *BRAF* gain, in addition to increased gains in cell cycle activators and losses of cell cycle inhibitors compared to *IDH*^mut^ gliomas. This is likely to be biologically relevant, as others have shown that the number of CNAs in the receptor tyrosine kinase pathway correlates with pathway activation measured by downstream kinase phosphorylation [16]. Amplification of *EGFR* has been shown to separate GBM into distinct clusters [8, 26, 2]. Although *IDH* mutation status was not reported in these clustering papers, alterations seen in the non-*EGFR* amplified group, such as losses on chromosome 13, mirror those seen in our *IDH*^mut^ glioblastomas. The lack of *EGFR* amplification in *IDH*^mut^ glioblastoma was also seen in the TCGA samples [4]. A similar pattern of chromosome gains and losses distinguished primary and secondary glioblastoma, which have a high rate of *IDH* mutations [22]. These results validate our findings that growth factor receptor signaling, particularly in the EGFR pathway, differs between *IDH*^mut^ and *IDH*^wt^ gliomas.

We found an unexpectedly large number of intrachromosomal breakpoints, also known as chromothripsis, in our *IDH*^mut^ GBM tumors. Upon closer inspection, we observed that chromothripsis is more likely when *TP53* is altered through deletion and/or LOH, and others have found that astrocytes lacking p53 have more chromosome breaks [47] and medulloblastoma due to inherited *TP53* mutations have increased chromothripsis [32]. Different definitions of chromothripsis have been proposed in the literature, and although many of our samples meet the common definition of chromothripsis, they do not all fit into every definition of chromothripsis [38, 19]. Therefore, we cannot conclude whether the massive intrachromosomal instability seen in *IDH*^mut^ gliomas in our samples occurs in one event (“true” chromothripsis) or in sequential events over time (severe chromosomal instability). Nevertheless, our data support that *IDH*^mut^ high grade tumors contain the highest number of alternating, intrachromosomal breakpoints.

Our study strengths include the relatively large number of *IDH*^mut^ GBM and *IDH*^wt^ lower grade gliomas relative to other data sets (including TCGA), allowing us to better characterize these uncommon groups. In addition, we have been able to use relatively novel, high-resolution MIP technology to analyze archived FFPE tissue with associated clinical variables and mature outcome data. Weaknesses include the overall small size of the series, which means that conclusions, particularly about between group differences and within group prognostic factors, must be taken as hypothesis-generating.

## Conclusions

In conclusion, we have shown that *IDH* and grade define four distinct groups of 1p/19q non-co-deleted gliomas determined by functionally important CNAs and unique prognostic factors. *IDH*^wt^ lower grade gliomas and grade IV gliomas are closely related and driven by common and well known alterations including *EGFR* amplification and *PTEN* deletion, while *IDH*^mut^ lower grade gliomas remain functionally distinct from grade IV gliomas. The transition of *IDH*^mut^ lower grade gliomas to grade IV gliomas involves loss of *PTEN* and dysregulation of cell cycle regulators, in addition to an apparent higher frequency of chromosomal instability and/or chromothripsis.

## Additional files

Additional file 1: Table S1. Loci with copy number alterations with an FDR <0.25 using the GISTIC algorithm in (A) *IDH* ^mut^ GBM, (B) *IDH* ^mut^ Grade II–III gliomas, (C) *IDH* ^wt^ GBM, and (D) *IDH* ^wt^ Grade II–III gliomas. Columns give the percent of samples within each subgroup with allelic imbalance, low level copy number gain, low level copy number loss, high level copy number gain, and homozygoud deletion at each locus. Additional file 2: Table S2. Loci with copy number alterations that are significantly different between *IDH* ^mut^ and *IDH* ^wt^ gliomas, regardless of grade, with FDR <0.25. Additional file 3: Table S3. Loci with copy number alterations that are significantly different between low and high grade *IDH* ^wt^ gliomas with FDR <0.25. Additional file 4: Table S4. Loci with copy number alterations that are significantly different between the two clusters of *IDH* ^mut^ gliomas identified by hierarchical clustering with FDR <0.25. Additional file 5: Table S5. Loci with copy number alterations that are significantly different between low and high grade *IDH* ^mut^ gliomas with FDR <0.25. Additional file 6: Table S6. Gene Ontology categories and pathways enriched using the ToppGene algorithm for (A) *IDH* ^mut^ Grade II–III gliomas, (B) *IDH* ^mut^ GBM, (C) *IDH* ^wt^ Grade II–III gliomas, (D) *IDH* ^wt^ GBM, (E) differences between *IDH* ^mut^ lower grade and high grade gliomas, and (F) differences between *IDH* ^wt^ low and high grade gliomas. Additional file 7: Table S7. Univariate and multivariate survival analysis in the entire population and within the four subgroups of 1p/19q non-co-deleted gliomas.
