# Supplementary material for: DNA copy number analysis of Grade II–III and Grade IV gliomas reveals differences in molecular ontogeny including chromothripsis associated with IDH mutation status
Source: Acta Neuropathol Commun. 2015 Jun 20;3:34. doi: 10.1186/s40478-015-0213-3 (PMC4474351; doi:10.1186/s40478-015-0213-3)
Supplement: Additional file 1: Table S1. — Loci with copy number alterations with an FDR <0.25 using the GISTIC algorithm in (A) IDH mut GBM, (B) IDH mut Grade II–III gliomas, (C) IDH wt GBM, and (D) IDH wt Grade II–III gliomas. Columns give the percent of samples within each subgroup with allelic imbalance, low level copy number gain, low level copy number loss, high level copy number gain, and homozygoud deletion at each locus. [file 40478_2015_213_MOESM1_ESM.doc]

Supplemental Table 1

A IDH mutant, GBM

| **Term** | **Allelic Imbalance %** | **CN Gain %** | **CN Loss %** | **High Copy Gain %** | **Homozygous Copy Loss %** | **LOH %** | **Locus** | **Q-Bound** | **G-Score** | **Genes** |
| --- | --- | --- | --- | --- | --- | --- | --- | --- | --- | --- |
| **chr1:9,676,608-9,767,003** | 12.5 | 16.67 | 8.33 | 8.33 | 0 | 25 | p36.22 | 0.244 | 145.229 | PIK3CD  CLSTN1 |
| **chr1:154,584,253-154,900,178** | 16.67 | 54.17 | 0 | 16.67 | 0 | 4.17 | q22-q23.1 | 0.244 | 60.647 | RHBG  C1orf61  MIR9-1  MEF2D  IQGAP3  TTC24  APOA1BP  GPATCH4  HAPLN2  BCAN |
| **chr2:15,991,443-16,124,756** | 16.67 | 0 | 0 | 12.5 | 0 | 12.5 | p24.3 | 0.124 | 154.174 | MYCNOS  MYCN |
| **chr2:58,236,094-58,326,829** | 12.5 | 8.33 | 0 | 4.17 | 0 | 16.67 | p16.1 | 0.244 | 126.116 | VRK2  FANCL |
| **chr3:182,613,173-182,803,666** | 20.83 | 29.17 | 4.17 | 8.33 | 0 | 4.17 | q26.33 | 0.244 | 31.540 | SOX2-OT |
| **chr3:20,116,875-20,140,190** | 8.33 | 25 | 25 | 8.33 | 4.17 | 29.17 | p24.3 | 0.248 | 30.802 | KAT2B |
| **chr4:55,221,698-55,235,941** | 8.33 | 29.17 | 0 | 12.5 | 0 | 8.33 | q12 | 0.244 | 124.402 | KIT |
| **chr6:44,305,868-44,327,246** | 20.83 | 37.5 | 0 | 8.33 | 0 | 0 | p21.1 | 0.244 | 33.231 | SLC29A1  HSP90AB1 |
| **chr6:163,516,896-163,908,991** | 16.67 | 41.67 | 12.5 | 4.17 | 0 | 0 | q26 | 0.245 | 31.117 | PACRG  PACRG-AS1  DKFZp451B082  CAHM  QKI |
| **chr7:54,834,444-55,141,816** | 12.5 | 37.5 | 0 | 8.33 | 0 | 8.33 | p11.2 | 0.244 | 132.640 | RP11-745C15.2  EGFR |
| **chr7:92,151,211-92,285,322** | 12.5 | 50 | 0 | 12.5 | 0 | 33.33 | q21.2 | 0.244 | 75.150 | CDK6 |
| **chr8:131,180,164-131,423,663** | 29.17 | 16.67 | 4.17 | 29.17 | 0 | 8.33 | q24.21 | 0.244 | 56.625 | ASAP1  ASAP1-IT1 |
| **chr9:127,263,932-127,365,296** | 16.67 | 37.5 | 4.17 | 8.33 | 0 | 29.17 | q33.3 | 0.244 | 37.856 | MAPKAP1 |
| **chr10:70,226,704-70,300,188** | 16.67 | 12.5 | 20.83 | 4.17 | 0 | 20.83 | q21.3 | 0.124 | 169.493 | STOX1 |
| **chr10:126,139,826-126,222,887** | 20.83 | 0 | 54.17 | 4.17 | 0 | 37.5 | q26.13 | 0.244 | 102.925 | LHPP |
| **chr10:127,605,604-127,721,128** | 25 | 0 | 66.67 | 4.17 | 0 | 45.83 | q26.2 | 0.244 | 102.767 | FANK1  FANK1-AS1  ADAM12 |
| **chr10:123,233,091-123,356,844** | 16.67 | 0 | 58.33 | 4.17 | 0 | 45.83 | q26.13 | 0.244 | 101.358 | FGFR2 |
| **chr10:128,567,061-128,692,103** | 20.83 | 0 | 62.5 | 4.17 | 0 | 37.5 | q26.2 | 0.244 | 97.749 | DOCK1 |
| **chr10:103,318,090-103,401,402** | 20.83 | 4.17 | 37.5 | 8.33 | 0 | 29.17 | q24.32 | 0.244 | 84.622 | DOCK1  POLL  DPCD  MIR3158-2  MIR3158-1  FBXW4 |
| **chr10:73,782,839-73,913,506** | 16.67 | 16.67 | 20.83 | 4.17 | 0 | 41.67 | q22.1 | 0.244 | 74.780 | DNAJB12  MICU1 |
| **chr10:22,932,002-22,936,942** | 29.17 | 25 | 8.33 | 16.67 | 0 | 20.83 | p12.2 | 0.244 | 47.850 | PIP4K2A |
| **chr11:82,107,748-82,248,892** | 16.67 | 25 | 0 | 16.67 | 0 | 8.33 | q14.1 | 0.244 | 45.666 | FAM181B  PRCP |
| **chr12:56,283,731-56,432,370** | 29.17 | 12.5 | 4.17 | 37.5 | 0 | 50 | q13.3-q14.1 | 0.000 | 410.197 | DTX3  ARHGEF25  SLC26A10  B4GALNT1  OS9  AGAP2  AGAP2-AS1  TSPAN31  CDK4  MIR6759 |
| **chr12:3,787,066-3,954,590** | 25 | 12.5 | 12.5 | 25 | 0 | 20.83 | p13.32 | 0.244 | 134.753 | PARP11 |
| **chr12:51,060,816-51,253,712** | 20.83 | 12.5 | 29.17 | 8.33 | 0 | 25 | q13.13 | 0.244 | 74.623 | KRT84  KRT82  KRT75  KRT6B  KRT6C  KRT6A  KRT5  KRT71  KRT74 |
| **chr12:25,226,961-25,288,759** | 29.17 | 29.17 | 4.17 | 16.67 | 0 | 12.5 | p12.1 | 0.244 | 44.058 | CASC1  LYRM5  KRAS |
| **chr12:6,640,017-6,749,722** | 25 | 37.5 | 4.17 | 29.17 | 0 | 25 | p13.31 | 0.244 | 32.696 | ING4  ZNF384  PIANP  COPS7A  MLF2  PTMS |
| **chr13:114,025,734-114,031,966** | 25 | 20.83 | 8.33 | 20.83 | 0 | 12.5 | q34 | 0.244 | 43.029 | CDC16 |
| **chr14:26,310,352-26,536,264** | 16.67 | 41.67 | 0 | 4.17 | 0 | 0 | q12 | 0.244 | 31.735 | RP11-626P14.1  LOC102724890  RP11-384J4.1  MIR4307 |
| **chr15:91,225,281-91,319,897** | 8.33 | 29.17 | 4.17 | 12.5 | 0 | 4.17 | q26.1 | 0.244 | 36.463 | LOC100507217  CHD2  MIR3175 |
| **chr16:56,216,776-56,237,322** | 0 | 54.17 | 4.17 | 4.17 | 0 | 4.17 | q13 | 0.244 | 35.485 | GPR56 |
| **chr17:26,662,575-26,671,981** | 8.33 | 45.83 | 8.33 | 4.17 | 0 | 16.67 | q11.2 | 0.244 | 37.741 | NF1  EVI2B  EVI2A |
| **chr19:1,148,106-1,314,459** | 20.83 | 54.17 | 0 | 0 | 0 | 0 | p13.3 | 0.244 | 32.681 | STK11  C19orf26  ATP5D  MIDN  CIRBP-AS1  CIRBP  C19orf24  EFNA2  MUM1 |
| **chr20:22,959,947-22,974,954** | 4.17 | 12.5 | 0 | 4.17 | 0 | 4.17 | p11.21 | 0.244 | 82.060 | SSTR4  THBD |
| **chr20:62,251,998-62,435,964** | 12.5 | 37.5 | 4.17 | 8.33 | 4.17 | 8.33 | q13.33 | 0.245 | 31.462 | MYT1  PCMTD2  LINC00266-1 |
| **chr1:3,913,275-4,622,610** | 16.67 | 8.33 | 37.5 | 0 | 0 | 8.33 | p36.32 | 0.085 | 19.878 | RP5-1166F10.1  AJAP1 |
| **chr3:43,495,700-44,087,293** | 20.83 | 4.17 | 41.67 | 0 | 4.17 | 29.17 | p22.1-p21.33 | 0.001 | 27.908 | ANO10  ABHD5 |
| **chr4:185,225,878-185,361,726** | 8.33 | 4.17 | 33.33 | 0 | 0 | 20.83 | q35.1 | 0.013 | 23.624 | ENPP6 |
| **chr5:173,712,324-174,625,292** | 29.17 | 0 | 33.33 | 0 | 0 | 12.5 | q35.2 | 0.145 | 18.634 | MSX2  MIR4634  FLJ16171 |
| **chr9:21,958,027-21,968,400** | 25 | 16.67 | 29.17 | 4.17 | 16.67 | 33.33 | p21.3 | 0.000 | 43.841 | CDKN2A |
| **chr10:131,188,181-131,222,058** | 20.83 | 0 | 83.33 | 0 | 0 | 37.5 | q26.3 | 0.000 | 46.320 | MGMT |
| **chr11:2,089,547-2,550,210** | 16.67 | 0 | 75 | 0 | 0 | 62.5 | p15.5 | 0.000 | 42.979 | INS-IGF2  IGF2  MIR483  IGF2-AS  INS  TH  MIR4686  ASCL2  C11orf21  TSPAN32  CD81-AS1  CD81  TSSC4  TRPM5  KCNQ1 |
| **chr12:129,083,911-129,102,059** | 20.83 | 0 | 41.67 | 0 | 0 | 16.67 | q24.33 | 0.067 | 20.409 | RP11-474D1.3 |
| **chr13:48,222,949-48,293,839** | 16.67 | 0 | 45.83 | 0 | 0 | 29.17 | q14.2 | 0.002 | 27.166 |  |
| **chr14:95,248,258-95,490,565** | 33.33 | 16.67 | 33.33 | 0 | 0 | 4.17 | q32.13-q32.2 | 0.211 | 17.743 | TCL1A  TUNAR |
| **chr19:61,095,127-61,263,042** | 20.83 | 4.17 | 50 | 0 | 0 | 20.83 | q13.42 | 0.002 | 26.781 | NLRP13  NLRP8  NLRP5 |
| **chr22:32,665,429-33,387,909** | 16.67 | 0 | 41.67 | 0 | 0 | 25 | q12.3 | 0.004 | 25.843 |  |
| **chrX:3,219,016-3,503,088** | 0 | 0 | 29.17 | 0 | 0 | 16.67 | p22.33 | 0.000 | 61.727 | MXRA5 |
| **chrX:129,792,316-131,244,790** | 8.33 | 0 | 25 | 0 | 0 | 20.83 | q25-q26.2 | 0.000 | 58.376 | ENOX2  ARHGAP36  IGSF1  OR13H1  FIRRE  MST4  FRMD7  RAP2C  RAP2C-AS1 |

B IDH wild type, GBM

| **Term** | **Allelic Imbalance %** | **CN Gain %** | **CN Loss %** | **High Copy Gain %** | **Homozygous Copy Loss %** | **LOH %** | **Locus** | **Q-Bound** | **G-Score** | **Genes** |
| --- | --- | --- | --- | --- | --- | --- | --- | --- | --- | --- |
| **chr1:203,320,779-203,376,205** | 4.55 | 9.09 | 0 | 18.18 | 0 | 18.18 | q32.1 | 0.02 | 158.87 | RBBP5 |
| **chr1:241,972,641-242,065,530** | 4.55 | 27.27 | 0 | 9.09 | 0 | 9.09 | q44 | 0.21 | 109.18 | AKT3 |
| **chr1:17,454,930-17,622,420** | 13.64 | 9.09 | 0 | 4.55 | 0 | 9.09 | p36.13 | 0.24 | 25.76 | PADI3  PADI4  PADI6  RCC2 |
| **chr3:182,784,320-183,051,627** | 13.64 | 18.18 | 0 | 9.09 | 0 | 9.09 | q26.33 | 0.22 | 28.60 | SOX2-OT  SOX2 |
| **chr3:10,165,710-10,166,538** | 9.09 | 40.91 | 0 | 4.55 | 0 | 13.64 | p25.3 | 0.25 | 25.33 | VHL |
| **chr4:54,392,642-54,847,902** | 18.18 | 4.55 | 0 | 22.73 | 0 | 18.18 | q12 | 0.21 | 78.29 | RPL21P44  CHIC2  GSX2  PDGFRA |
| **chr7:55,180,181-55,219,961** | 40.91 | 54.55 | 0 | 45.45 | 0 | 36.36 | p11.2 | 0.00 | 685.83 | EGFR  EGFR-AS1 |
| **chr7:116,269,147-116,350,060** | 63.64 | 77.27 | 0 | 4.55 | 0 | 13.64 | q31.2 | 0.21 | 60.92 | CAPZA2 |
| **chr7:46,766,095-47,224,441** | 63.64 | 63.64 | 0 | 13.64 | 0 | 9.09 | p12.3 | 0.21 | 41.81 |  |
| **chr12:96,086,847-96,240,839** | 0 | 0 | 0 | 4.55 | 0 | 9.09 | q23.1 | 0.21 | 84.12 |  |
| **chr12:61,572,045-61,782,274** | 13.64 | 0 | 0 | 4.55 | 0 | 4.55 | q14.2 | 0.21 | 73.06 | PPM1H |
| **chr12:98,126,671-98,418,179** | 9.09 | 0 | 0 | 4.55 | 0 | 18.18 | q23.1 | 0.21 | 59.33 | ANKS1B |
| **chr15:23,266,948-23,301,342** | 13.64 | 4.55 | 13.64 | 4.55 | 0 | 18.18 | q11.2 | 0.21 | 76.22 |  |
| **chr15:31,595,436-31,653,142** | 13.64 | 0 | 22.73 | 4.55 | 0 | 9.09 | q12 | 0.21 | 71.88 | RYR3 |
| **chr17:211,452-381,179** | 27.27 | 13.64 | 4.55 | 4.55 | 0 | 13.64 | q14 | 0.21 | 102.19 | C17orf97  FAM101B  VPS53 |
| **chr17:35,114,719-35,157,614** | 9.09 | 13.64 | 4.55 | 4.55 | 0 | 45.45 | p13.3 | 0.21 | 87.39 | ERBB2  MIR4728  MIEN1  GRB7 |
| **chr17:31,991,991-32,050,432** | 27.27 | 0 | 4.55 | 4.55 | 0 | 9.09 | q12 | 0.21 | 54.15 | GGNBP2  DHRS11  MRM1 |
| **chr17:24,443,366-24,757,842** | 18.18 | 4.55 | 0 | 4.55 | 0 | 18.18 | q11.2 | 0.23 | 28.15 | MYO18A  CRYBA1  NUFIP2  MIR4523  TAOK1 |
| **chr19:933,404-1,312,594** | 27.27 | 27.27 | 0 | 9.09 | 0 | 0 | p13.3 | 0.23 | 27.61 | WDR18  GRIN3B  TMEM259  CNN2  ABCA7  HMHA1  POLR2E  GPX4  SBNO2  STK11  C19orf26  ATP5D  MIDN  CIRBP-AS1  CIRBP  C19orf24  EFNA2  MUM1 |
| **chr22:44,101,332-44,129,123** | 18.18 | 4.55 | 22.73 | 4.55 | 0 | 18.18 | q13.31 | 0.21 | 80.35 | FAM118A  SMC1B |
| **chr22:34,729,680-34,838,470** | 36.36 | 4.55 | 22.73 | 4.55 | 0 | 9.09 | q12.3 | 0.21 | 72.74 | RBFOX2 |
| **chrX:51,453,907-52,358,342** | 0 | 0 | 4.55 | 4.55 | 0 | 27.27 | p11.22 | 0.21 | 31.27 | CENPVP1  CENPVP2  GSPT2  MAGED1  SNORA11D  SNORA11E  MAGED4B  MAGED4  MIR8088  XAGE2  XAGE2B  XAGE1A  XAGE1C  XAGE1E  XAGE1B  XAGE1D |
| **chr6:161,744,633-161,802,882** | 22.73 | 0 | 27.27 | 0 | 4.55 | 9.09 | q26 | 0.00 | 21.32 | PARK2 |
| **chr9:21,975,897-21,988,593** | 27.27 | 13.64 | 27.27 | 0 | 31.82 | 4.55 | p21.3 | 0.00 | 52.11 | CDKN2A  CDKN2B-AS1 |
| **chr10:89,679,777-89,694,536** | 22.73 | 4.55 | 63.64 | 0 | 13.64 | 40.91 | q23.31 | 0.00 | 48.43 | PTEN |
| **chr10:28,056,643-28,500,466** | 40.91 | 0 | 72.73 | 0 | 0 | 40.91 | p12.1-p11.3 | 0.00 | 36.02 | MKX  ARMC4  MPP7 |
| **chr13:54,521,376-54,970,723** | 18.18 | 9.09 | 36.36 | 0 | 0 | 31.82 | q21.1-q12.2 | 0.01 | 20.16 | MIR5007 |
| **chr14:36,213,028-36,493,939** | 45.45 | 4.55 | 50 | 0 | 0 | 45.45 | q13.3 | 0.01 | 21.03 | PAX9  SLC25A21  MIR4503 |
| **chr15:40,157,746-40,473,051** | 18.18 | 0 | 27.27 | 0 | 0 | 45.45 | q15.1 | 0.04 | 17.88 | PLA2G4D  PLA2G4F  VPS39  MIR627  TMEM87A  GANC  CAPN3 |
| **chr22:27,455,669-28,349,968** | 40.91 | 0 | 31.82 | 0 | 0 | 36.36 | q12.1 | 0.09 | 15.98 | CHEK2  HSCB  CCDC117  XBP1  ZNRF3  ZNRF3-AS1  C22orf31  KREMEN1  EMID1  RHBDD3  EWSR1  GAS2L1  RASL10A  AP1B1  MIR3653  SNORD125  RFPL1S  RFPL1  NEFH  THOC5  NIPSNAP1  NF2 |
| **chrX:2,709,968-51,453,907** | 18.18 | 4.55 | 4.55 | 0 | 0 | 40.91 | p22.33-p11.22 | 0.00 | 49.24 | 330 genes |
| **chrX:61,860,760-154,913,754** | 22.73 | 4.55 | 4.55 | 4.55 | 0 | 40.91 | q11.1-q28 | 0.00 | 48.48 | 648 genes |

C. IDH mutant Grade II-III

| **Term** | **Allelic Imbalance %** | **CN Gain %** | **CN Loss %** | **High Copy Gain %** | **Homozygous Copy Loss %** | **LOH %** | **Locus** | **Q-Bound** | **G-Score** | **Genes** |
| --- | --- | --- | --- | --- | --- | --- | --- | --- | --- | --- |
| **chr1:241,878,972-242,058,804** | 17.02 | 46.81 | 2.13 | 4.26 | 0 | 29.79 | q44 | 0.20 | 26.71 | AKT3 |
| **chr1:11,222,280-11,228,859** | 8.51 | 34.04 | 0 | 2.13 | 0 | 10.64 | p36.22 | 0.21 | 19.30 | MTOR |
| **chr2:11,709,756-11,754,253** | 25.53 | 10.64 | 0 | 6.38 | 0 | 4.26 | p25.1 | 0.23 | 18.69 | NTSR2  LPIN1 |
| **chr6:26,264,942-26,319,170** | 4.26 | 27.66 | 2.13 | 4.26 | 0 | 17.02 | p22.1 | 0.22 | 18.95 | HIST1H1E  HIST1H2BD  HIST1H2BD  HIST1H2BE  HIST1H4D  HIST1H3D  HIST1H2AD  HIST1H2BF  HIST1H4E |
| **chr7:55,060,282-55,147,471** | 10.64 | 31.91 | 0 | 8.51 | 0 | 8.51 | p11.2 | 0.20 | 82.59 | EGFR |
| **chr7:116,132,107-116,384,973** | 29.79 | 34.04 | 0 | 6.38 | 0 | 19.15 | q31.2 | 0.20 | 43.38 | MET  CAPZA2  ST7-AS1  ST7  ST7-OT4 |
| **chr7:104,118,770-104,124,954** | 21.28 | 29.79 | 0 | 2.13 | 0 | 12.77 | q22.1 | 0.21 | 19.69 | LHFPL3 |
| **chr8:115,270,507-116,001,285** | 19.15 | 25.53 | 4.26 | 17.02 | 0 | 19.15 | q23.3 | 0.20 | 34.78 |  |
| **chr9:138,162,492-138,714,976** | 19.15 | 21.28 | 10.64 | 6.38 | 0 | 6.38 | q34.3 | 0.21 | 19.19 | LHX3  QSOX2  DKFZP434A062  GPSM1  DNLZ  CARD9  SNAPC4  SDCCAG3  PMPCA  INPP5E  SEC16A  C9orf163  NOTCH1  MIR4673  MIR4674  EGFL7  MIR126  AGPAT2 |
| **chr10:89,551,034-89,557,840** | 27.66 | 21.28 | 31.91 | 2.13 | 0 | 14.89 | q23.2 | 0.20 | 23.63 | ATAD1 |
| **chr11:77,608,255-78,038,842** | 25.53 | 25.53 | 0 | 6.38 | 0 | 12.77 | q14.1 | 0.20 | 57.25 | GAB2  NARS2 |
| **chr12:56,345,952-56,421,720** | 19.15 | 10.64 | 6.38 | 25.53 | 0 | 36.17 | q14.1 | 0.02 | 174.24 | OS9  AGAP2  AGAP2-AS1 |
| **chr12:60,378,662-60,476,969** | 21.28 | 6.38 | 19.15 | 4.26 | 0 | 10.64 | q14.1 | 0.06 | 140.74 | FAM19A2 |
| **chr12:4,191,249-4,448,501** | 25.53 | 12.77 | 8.51 | 19.15 | 0 | 17.02 | p13.32 | 0.09 | 138.10 | CCND2  C12orf5  FGF23  FGF6 |
| **chr12:100,127,883-100,286,568** | 21.28 | 2.13 | 23.4 | 4.26 | 0 | 10.64 | q23.2 | 0.20 | 115.08 | SLC5A8  UTP20 |
| **chr12:58,668,427-58,859,082** | 25.53 | 4.26 | 19.15 | 4.26 | 0 | 8.51 | q14.1 | 0.20 | 114.99 |  |
| **chr12:85,150,916-85,420,459** | 21.28 | 12.77 | 23.4 | 2.13 | 0 | 27.66 | q21.32 | 0.20 | 110.39 | MGAT4C |
| **chr12:59,166,189-59,291,633** | 19.15 | 4.26 | 17.02 | 2.13 | 0 | 14.89 | q14.1 | 0.20 | 109.39 |  |
| **chr12:30,836,498-31,274,728** | 21.28 | 6.38 | 8.51 | 17.02 | 0 | 17.02 | p11.21 | 0.20 | 89.23 | LINC00941  TSPAN11  DDX11-AS1  DDX11 |
| **chr12:65,780,077-65,910,153** | 17.02 | 4.26 | 25.53 | 2.13 | 0 | 17.02 | q14.3 | 0.20 | 87.60 |  |
| **chr12:21,221,630-21,301,950** | 14.89 | 12.77 | 12.77 | 12.77 | 0 | 25.53 | p12.1 | 0.20 | 27.04 | SLCO1B1 |
| **chr12:114,913,661-114,932,608** | 21.28 | 19.15 | 17.02 | 4.26 | 0 | 12.77 | q24.21 | 0.20 | 22.78 | MED13L |
| **chr17:74,877,523-74,904,333** | 25.53 | 21.28 | 8.51 | 6.38 | 0 | 0 | q25.3 | 0.20 | 23.68 | RBFOX3 |
| **chr18:53,351,497-55,394,239** | 17.02 | 12.77 | 0 | 2.13 | 0 | 10.64 | q21.31-q21.32 | 0.20 | 50.64 | FECH  NARS  RP11-35G9.3  ATP8B1  NEDD4L  MIR122  MIR3591  ALPK2  LOC101927322  MALT1  ZNF532  OACYLP  SEC11C  GRP  RAX  CPLX4  LMAN1  CCBE1 |
| **chr20:1,481,583-1,576,633** | 19.15 | 19.15 | 4.26 | 8.51 | 0 | 4.26 | p13 | 0.20 | 31.19 | SIRPD  SIRPB1  SIRPG  RP11-77C3.3 |
| **chr2:237,908,775-238,153,692** | 12.77 | 2.13 | 19.15 | 0 | 0 | 6.38 | q37.3 | 0.22 | 13.15 | COL6A3  MLPH  MIR6811  PRLH  RAB17 |
| **chr5:2,224,444-2,794,049** | 19.15 | 6.38 | 36.17 | 0 | 0 | 6.38 | p15.33 | 0.03 | 17.52 | LOC100506858 |
| **chr6:150,200,034-150,435,810** | 12.77 | 4.26 | 19.15 | 0 | 0 | 21.28 | q25.1 | 0.17 | 13.96 | LRP11  RAET1E-AS1  RAET1E  RAET1G  ULBP2  ULBP1  RAET1K  RAET1L  ULBP3 |
| **chr9:0-658,114** | 31.91 | 10.64 | 42.55 | 2.13 | 6.38 | 23.4 | p24.3 | 0.00 | 35.38 | DDX11L5  WASH1  FAM138C  FOXD4  CBWD1  C9orf66  DOCK8  KANK1 |
| **chr10:131,415,314-131,949,667** | 23.4 | 0 | 63.83 | 0 | 0 | 27.66 | q26.3 | 0.00 | 22.47 | MGMT  EBF3  MIR4297  LINC00959  CTAGE7P  GLRX3 |
| **chr11:5,279,480-5,370,675** | 23.4 | 2.13 | 48.94 | 0 | 0 | 31.91 | p15.4 | 0.05 | 16.60 | OR51B4  OR51B2  OR51B5  OR51B6  OR51M1 |
| **chr12:78,979,684-79,129,129** | 19.15 | 14.89 | 34.04 | 0 | 0 | 29.79 | q21.31 | 0.01 | 19.88 | OTOGL |
| **chr12:128,728,313-129,367,190** | 21.28 | 4.26 | 36.17 | 2.13 | 0 | 10.64 | q24.33 | 0.23 | 12.97 | TMEM132D  RP11-474D1.3  FZD10-AS1  FZD10 |
| **chr13:46,284,501-47,765,523** | 27.66 | 4.26 | 44.68 | 0 | 0 | 44.68 | q14.2 | 0.00 | 21.96 | HTR2A  HTR2A-AS1  SUCLA2  NUDT15  MED4  MED4-AS1  ITM2B |
| **chr14:71,737,169-71,997,347** | 25.53 | 6.38 | 27.66 | 0 | 0 | 12.77 | q24.2 | 0.03 | 17.54 | RGS6 |
| **chr15:18,421,386-18,942,177** | 19.15 | 4.26 | 21.28 | 0 | 0 | 12.77 | q11.2 | 0.07 | 15.74 | CHEK2P2  HERC2P3 |
| **chr19:62,178,860-62,435,459** | 14.89 | 2.13 | 38.3 | 0 | 0 | 17.02 | q13.43 | 0.02 | 17.99 | USP29  ZIM3  DUXA  ZNF264  AURKC |
| **chrX:12,301,052-12,732,459** | 19.15 | 6.38 | 23.4 | 0 | 0 | 10.64 | p22.2 | 0.00 | 43.56 | FRMPD4  PRPS2 |
| **chrX:153,674,058-153,810,477** | 12.77 | 6.38 | 10.64 | 0 | 2.13 | 34.04 | q28 | 0.00 | 39.51 | MPP1  SMIM9  F8  H2AFB2  H2AFB1  H2AFB3  F8A1  F8A2  F8A3  MIR1184-3  MIR1184-1  MIR1184-2 |

D. IDH wild-type, Grade II-III

| **Term** | **Allelic Imbalance %** | **CN Gain %** | **CN Loss %** | **High Copy Gain %** | **Homozygous Copy Loss %** | **LOH %** | **Locus** | **Q-Bound** | **G-Score** | **Genes** |
| --- | --- | --- | --- | --- | --- | --- | --- | --- | --- | --- |
| **chr1:202,800,228-202,937,333** | 17.65 | 35.29 | 0 | 11.76 | 0 | 5.88 | q32.1 | 0.09 | 37.38 | LRRN2 |
| **chr1:154,825,039-154,887,861** | 17.65 | 52.94 | 0 | 11.76 | 0 | 0 | q23.1 | 0.18 | 16.86 | APOA1BP  GPATCH4  HAPLN2  BCAN |
| **chr3:10,165,710-10,168,983** | 0 | 47.06 | 0 | 5.88 | 0 | 11.76 | p25.3 | 0.09 | 24.09 | VHL |
| **chr4:53,809,605-54,000,483** | 0 | 11.76 | 0 | 11.76 | 0 | 23.53 | q12 | 0.09 | 73.56 | SCFD2  FIP1L1 |
| **chr4:47,247,560-47,349,557** | 0 | 17.65 | 0 | 5.88 | 0 | 11.76 | p12 | 0.12 | 19.32 | ATP10D  CORIN  MIR8053 |
| **chr5:149,502,901-149,545,912** | 5.88 | 41.18 | 0 | 5.88 | 0 | 0 | q33.1 | 0.10 | 20.75 | PDGFRB  CDX1 |
| **chr5:1,336,647-1,496,170** | 0 | 41.18 | 5.88 | 0 | 0 | 0 | p15.33 | 0.17 | 17.36 | TERT  MIR4457  CLPTM1L  SLC6A3 |
| **chr6:26,255,341-26,325,618** | 0 | 35.29 | 5.88 | 5.88 | 0 | 23.53 | p22.1 | 0.09 | 22.18 | HIST1H1E  HIST1H2BD  HIST1H2BE  HIST1H4D  HIST1H3D  HIST1H2AD  HIST1H2BF  HIST1H4E  HIST1H2BG  HIST1H2AE |
| **chr7:55,050,384-55,147,471** | 29.41 | 23.53 | 0 | 52.94 | 0 | 41.18 | p11.2 | 0.00 | 467.98 | EGFR |
| **chr7:92,151,211-92,238,685** | 35.29 | 41.18 | 0 | 23.53 | 0 | 47.06 | q21.2 | 0.09 | 124.01 | CDK6 |
| **chr9:137,127,903-137,224,802** | 17.65 | 41.18 | 11.76 | 5.88 | 0 | 5.88 | q34.3 | 0.10 | 21.44 | OLFM1  LOC401557 |
| **chr9:44,115,323-45,623,725** | 5.88 | 35.29 | 5.88 | 5.88 | 0 | 64.71 | p11.2 | 0.10 | 20.84 | LINC01189  FAM27C  FAM27A  FAM27E2 |
| **chr9:37,813,214-37,959,138** | 17.65 | 29.41 | 5.88 | 5.88 | 0 | 5.88 | p13.2 | 0.18 | 16.79 | DCAF10  SLC25A51  SHB |
| **chr11:64,941,898-65,197,105** | 0 | 29.41 | 0 | 5.88 | 0 | 0 | q13.1 | 0.13 | 18.72 | NEAT1  MIR612  MALAT1  MIR548AR  MIR548BA  SCYL1  LTBP3  SSSCA1-AS1  SSSCA1  FAM89B  EHBP1L1  KCNK7  MAP3K11  PCNXL3  MIR4690  SIPA1  MIR4489  RELA |
| **chr12:56,413,988-56,468,659** | 17.65 | 41.18 | 0 | 23.53 | 0 | 23.53 | q14.1 | 0.00 | 297.90 | AGAP2  TSPAN31  CDK4  MIR6759  MARCH9  CYP27B1  METTL1  METTL21B  TSFM |
| **chr12:67,339,248-67,642,488** | 17.65 | 11.76 | 0 | 11.76 | 0 | 11.76 | q15 | 0.01 | 187.69 | RAP1B  RP11-637A17.2  NUP107  SLC35E3  LOC100130075  MDM2  CPM |
| **chr12:56,835,862-56,909,509** | 5.88 | 11.76 | 5.88 | 5.88 | 0 | 17.65 | q14.1 | 0.09 | 132.48 |  |
| **chr12:58,840,996-59,617,191** | 11.76 | 5.88 | 5.88 | 5.88 | 0 | 29.41 | q14.1 | 0.09 | 96.26 |  |
| **chr12:6,826,519-6,833,936** | 0 | 41.18 | 0 | 5.88 | 0 | 5.88 | p13.31 | 0.20 | 16.39 | GNB3  CDCA3  USP5 |
| **chr16:56,221,243-56,232,272** | 11.76 | 41.18 | 0 | 17.65 | 0 | 0 | q13 | 0.09 | 34.17 | GPR56 |
| **chr16:0-230,273** | 5.88 | 41.18 | 0 | 5.88 | 0 | 11.76 | p13.3 | 0.10 | 21.40 | DDX11L10  LOC100288778  MIR6859-1  MIR6859-2  POLR3K  SNRNP25  RHBDF1  MPG  NPRL3  HBZ  HBM  HBA2  HBA1  HBQ1  LUC7L  ITFG3 |
| **chr17:7,975,653-8,048,365** | 11.76 | 41.18 | 0 | 5.88 | 0 | 0 | p13.1 | 0.09 | 23.01 | PER1  MIR6883  VAMP2  TMEM107  MIR4521  C17orf59 |
| **chr19:56,927,216-56,975,821** | 17.65 | 29.41 | 0 | 5.88 | 0 | 5.88 | q13.33 | 0.09 | 108.54 | FPR1  FPR2 |
| **chr19:4,700,984-4,940,283** | 23.53 | 76.47 | 0 | 0 | 0 | 11.76 | p13.3 | 0.09 | 30.50 | MIR7-3HG  MIR7-3  FEM1A  TICAM1  PLIN3  ARRDC5  UHRF1  MIR4747  KDM4B |
| **chr20:62,223,697-62,435,964** | 29.41 | 47.06 | 0 | 5.88 | 0 | 5.88 | q13.33 | 0.09 | 26.52 | MYT1  PCMTD2  LINC00266-1 |
| **chr20:3,679,613-3,792,743** | 23.53 | 52.94 | 0 | 0 | 0 | 5.88 | p13 | 0.10 | 21.16 | HSPA12B  C20orf27  SPEF1  CENPB  CDC25B  LOC101929125  AP5S1  MAVS |
| **chr6:134,551,413-134,707,666** | 23.53 | 5.88 | 35.29 | 0 | 0 | 11.76 | q23.2 | 0.06 | 15.83 | SGK1 |
| **chr9:22,099,154-22,137,966** | 17.65 | 5.88 | 41.18 | 0 | 23.53 | 5.88 | p21.3 | 0.00 | 38.36 | CDKN2B-AS1 |
| **chr10:67,613,328-67,744,979** | 41.18 | 0 | 76.47 | 0 | 5.88 | 35.29 | q21.3 | 0.00 | 37.15 | CTNNA3 |
| **chr10:37,330,440-37,806,180** | 35.29 | 0 | 82.35 | 0 | 0 | 47.06 | p11.21 | 0.00 | 34.27 | ANKRD30A  LINC00993 |
| **chr13:34,506,021-35,015,412** | 0 | 0 | 11.76 | 0 | 5.88 | 11.76 | q13.2-q13.3 | 0.22 | 13.03 | NBEA  MAB21L1  MIR548F5 |
| **chr14:42,433,139-44,043,237** | 29.41 | 0 | 41.18 | 0 | 0 | 35.29 | q21.2-q21.2 | 0.07 | 15.61 | FSCB |
| **chrX:141,365,813-141,888,817** | 0 | 0 | 5.88 | 0 | 5.88 | 5.88 | q27.2 | 0.00 | 48.20 |  |
| **chrX:38,873,400-40,118,466** | 5.88 | 0 | 0 | 0 | 5.88 | 5.88 | p11.4 | 0.00 | 47.53 | RP11-265P11.2  RP11-157D23.1  BCOR  RP11-320G24.1 |
