# Supplementary material for: DNA copy number analysis of Grade II–III and Grade IV gliomas reveals differences in molecular ontogeny including chromothripsis associated with IDH mutation status
Source: Acta Neuropathol Commun. 2015 Jun 20;3:34. doi: 10.1186/s40478-015-0213-3 (PMC4474351; doi:10.1186/s40478-015-0213-3)
Supplement: Additional file 2: Table S2. — Loci with copy number alterations that are significantly different between IDH mut and IDH wt gliomas, regardless of grade, with FDR <0.25. [file 40478_2015_213_MOESM2_ESM.docx]

| Region | Cytoband Location | Event | Region Length | Freq. in <IDHwt> (%) | Freq. in <IDHmut> (%) | Difference | p-value | q-bound | Gene Symbols |
| --- | --- | --- | --- | --- | --- | --- | --- | --- | --- |
| chr7:52,797,666-52,918,119 | p12.1 | Allelic Imbalance | 120453 | 71.43 | 8.89 | 62.54 | 0.00 | 0.00 |  |
| chr7:52,972,637-53,071,341 | p12.1 | Allelic Imbalance | 98704 | 71.43 | 8.89 | 62.54 | 0.00 | 0.00 | POM121L12 |
| chr7:53,072,961-53,143,429 | p12.1 | Allelic Imbalance | 70468 | 71.43 | 8.89 | 62.54 | 0.00 | 0.00 |  |
| chr7:53,143,429-53,286,535 | p12.1 | Allelic Imbalance | 143106 | 73.81 | 8.89 | 64.92 | 0.00 | 0.00 |  |
| chr7:53,286,535-53,378,628 | p12.1 | Allelic Imbalance | 92093 | 71.43 | 8.89 | 62.54 | 0.00 | 0.00 |  |
| chr7:6,662,134-6,693,160 | p22.1 | Allelic Imbalance | 31026 | 69.05 | 6.67 | 62.38 | 0.00 | 0.00 |  |
| chr7:6,718,012-7,166,118 | p22.1 | Allelic Imbalance | 448106 | 69.05 | 6.67 | 62.38 | 0.00 | 0.00 | PMS2CL, RSPH10B, RSPH10B2, CCZ1B, LOC100131257 |
| chr7:52,754,576-52,797,666 | p12.1 | Allelic Imbalance | 43090 | 69.05 | 8.89 | 60.16 | 0.00 | 0.00 |  |
| chr7:53,071,341-53,072,961 | p12.1 | Allelic Imbalance | 1620 | 69.05 | 8.89 | 60.16 | 0.00 | 0.00 | POM121L12 |
| chr7:53,378,628-53,423,410 | p12.1 | Allelic Imbalance | 44782 | 69.05 | 8.89 | 60.16 | 0.00 | 0.00 |  |
| chr7:54,138,849-54,144,169 | p11.2 | Allelic Imbalance | 5320 | 66.67 | 6.67 | 60.00 | 0.00 | 0.00 |  |
| chr7:6,616,394-6,662,134 | p22.1 | Allelic Imbalance | 45740 | 66.67 | 6.67 | 60.00 | 0.00 | 0.00 | ZNF853 |
| chr7:6,693,160-6,718,012 | p22.1 | Allelic Imbalance | 24852 | 66.67 | 6.67 | 60.00 | 0.00 | 0.00 | ZNF12 |
| chr7:7,166,118-7,224,058 | p22.1 - p21.3 | Allelic Imbalance | 57940 | 69.05 | 8.89 | 60.16 | 0.00 | 0.00 | C1GALT1 |
| chr7:47,047,297-47,203,061 | p12.3 | Allelic Imbalance | 155764 | 71.43 | 11.11 | 60.32 | 0.00 | 0.00 |  |
| chr7:52,918,119-52,972,637 | p12.1 | Allelic Imbalance | 54518 | 71.43 | 11.11 | 60.32 | 0.00 | 0.00 |  |
| chr7:7,664,369-7,797,881 | p21.3 | Allelic Imbalance | 133512 | 71.43 | 11.11 | 60.32 | 0.00 | 0.00 | RPA3, LOC729852 |
| chr7:18,917,849-18,925,565 | p21.1 | Allelic Imbalance | 7716 | 73.81 | 13.33 | 60.48 | 0.00 | 0.00 | HDAC9 |
| chr7:21,783,380-21,812,763 | p15.3 | Allelic Imbalance | 29383 | 73.81 | 13.33 | 60.48 | 0.00 | 0.00 | DNAH11 |
| chr7:29,107,410-29,117,386 | p15.1 | Allelic Imbalance | 9976 | 73.81 | 13.33 | 60.48 | 0.00 | 0.00 | CPVL |
| chr7:36,548,284-36,576,274 | p14.2 | Allelic Imbalance | 27990 | 73.81 | 13.33 | 60.48 | 0.00 | 0.00 | AOAH |
| chr7:20,824,508-20,841,653 | p15.3 | Allelic Imbalance | 17145 | 76.19 | 15.56 | 60.63 | 0.00 | 0.00 | RPL23P8 |
| chr7:54,105,645-54,138,849 | p11.2 | Allelic Imbalance | 33204 | 64.29 | 6.67 | 57.62 | 0.00 | 0.00 |  |
| chr7:63,244,003-63,292,700 | q11.21 | Allelic Imbalance | 48697 | 64.29 | 6.67 | 57.62 | 0.00 | 0.00 |  |
| chr7:18,713,198-18,917,849 | p21.1 | Allelic Imbalance | 204651 | 71.43 | 13.33 | 58.10 | 0.00 | 0.00 | HDAC9 |
| chr7:18,925,565-18,944,645 | p21.1 | Allelic Imbalance | 19080 | 71.43 | 13.33 | 58.10 | 0.00 | 0.00 | HDAC9 |
| chr7:21,769,801-21,783,380 | p15.3 | Allelic Imbalance | 13579 | 71.43 | 13.33 | 58.10 | 0.00 | 0.00 | DNAH11 |
| chr7:25,094,379-25,154,934 | p15.2 | Allelic Imbalance | 60555 | 69.05 | 11.11 | 57.94 | 0.00 | 0.00 | CYCS, C7orf31 |
| chr7:29,067,262-29,075,563 | p15.1 | Allelic Imbalance | 8301 | 71.43 | 13.33 | 58.10 | 0.00 | 0.00 | CPVL |
| chr7:29,098,378-29,107,410 | p15.1 | Allelic Imbalance | 9032 | 71.43 | 13.33 | 58.10 | 0.00 | 0.00 | CPVL |
| chr7:29,117,386-29,121,047 | p15.1 | Allelic Imbalance | 3661 | 71.43 | 13.33 | 58.10 | 0.00 | 0.00 | CPVL |
| chr7:29,164,187-29,282,391 | p15.1 | Allelic Imbalance | 118204 | 71.43 | 13.33 | 58.10 | 0.00 | 0.00 | CHN2 |
| chr7:34,342,737-34,663,902 | p14.3 | Allelic Imbalance | 321165 | 71.43 | 13.33 | 58.10 | 0.00 | 0.00 | NPSR1-AS1 |
| chr7:34,732,532-34,848,935 | p14.3 | Allelic Imbalance | 116403 | 71.43 | 13.33 | 58.10 | 0.00 | 0.00 | NPSR1-AS1, NPSR1 |
| chr7:35,631,918-35,642,220 | p14.2 | Allelic Imbalance | 10302 | 69.05 | 11.11 | 57.94 | 0.00 | 0.00 | HERPUD2 |
| chr7:36,469,984-36,548,284 | p14.2 | Allelic Imbalance | 78300 | 71.43 | 13.33 | 58.10 | 0.00 | 0.00 | AOAH |
| chr7:36,576,274-36,585,980 | p14.2 | Allelic Imbalance | 9706 | 71.43 | 13.33 | 58.10 | 0.00 | 0.00 | AOAH |
| chr7:46,433,290-46,669,696 | p13 - p12.3 | Allelic Imbalance | 236406 | 69.05 | 11.11 | 57.94 | 0.00 | 0.00 |  |
| chr7:47,035,963-47,047,297 | p12.3 | Allelic Imbalance | 11334 | 69.05 | 11.11 | 57.94 | 0.00 | 0.00 |  |
| chr7:47,203,061-47,287,659 | p12.3 | Allelic Imbalance | 84598 | 71.43 | 13.33 | 58.10 | 0.00 | 0.00 | TNS3 |
| chr7:49,014,545-49,235,117 | p12.3 | Allelic Imbalance | 220572 | 71.43 | 13.33 | 58.10 | 0.00 | 0.00 |  |
| chr7:49,547,609-49,824,191 | p12.3 - p12.2 | Allelic Imbalance | 276582 | 71.43 | 13.33 | 58.10 | 0.00 | 0.00 | VWC2 |
| chr7:5,230,208-5,243,488 | p22.1 | Allelic Imbalance | 13280 | 69.05 | 11.11 | 57.94 | 0.00 | 0.00 | WIPI2 |
| chr7:52,733,961-52,754,576 | p12.1 | Allelic Imbalance | 20615 | 66.67 | 8.89 | 57.78 | 0.00 | 0.00 |  |
| chr7:53,423,410-53,476,633 | p12.1 | Allelic Imbalance | 53223 | 66.67 | 8.89 | 57.78 | 0.00 | 0.00 |  |
| chr7:6,535,396-6,576,544 | p22.1 | Allelic Imbalance | 41148 | 66.67 | 8.89 | 57.78 | 0.00 | 0.00 | GRID2IP |
| chr7:7,224,058-7,387,070 | p21.3 | Allelic Imbalance | 163012 | 66.67 | 8.89 | 57.78 | 0.00 | 0.00 | C1GALT1, COL28A1 |
| chr7:7,654,248-7,664,369 | p21.3 | Allelic Imbalance | 10121 | 69.05 | 11.11 | 57.94 | 0.00 | 0.00 | RPA3, LOC729852 |
| chr7:7,797,881-7,812,215 | p21.3 | Allelic Imbalance | 14334 | 69.05 | 11.11 | 57.94 | 0.00 | 0.00 | LOC729852 |
| chr7:7,816,952-8,548,176 | p21.3 | Allelic Imbalance | 731224 | 69.05 | 11.11 | 57.94 | 0.00 | 0.00 | LOC729852, GLCCI1, ICA1, NXPH1 |
| chr7:8,694,332-8,902,807 | p21.3 | Allelic Imbalance | 208475 | 69.05 | 11.11 | 57.94 | 0.00 | 0.00 | NXPH1 |
| chr7:20,740,297-20,824,508 | p15.3 | Allelic Imbalance | 84211 | 73.81 | 15.56 | 58.25 | 0.00 | 0.00 | ABCB5, SP8 |
| chr7:20,841,653-20,914,841 | p15.3 | Allelic Imbalance | 73188 | 73.81 | 15.56 | 58.25 | 0.00 | 0.00 |  |
| chr7:21,812,763-21,820,890 | p15.3 | Allelic Imbalance | 8127 | 73.81 | 15.56 | 58.25 | 0.00 | 0.00 | DNAH11 |
| chr7:35,358,042-35,370,075 | p14.3 | Allelic Imbalance | 12033 | 73.81 | 15.56 | 58.25 | 0.00 | 0.00 | LOC401324 |
| chr7:21,913,073-21,922,352 | p15.3 | Allelic Imbalance | 9279 | 76.19 | 17.78 | 58.41 | 0.00 | 0.00 | CDCA7L |
| chr7:53,791,295-54,105,645 | p12.1 - p11.2 | Allelic Imbalance | 314350 | 61.90 | 6.67 | 55.24 | 0.00 | 0.00 | FLJ45974 |
| chr7:54,144,169-54,283,958 | p11.2 | Allelic Imbalance | 139789 | 61.90 | 6.67 | 55.24 | 0.00 | 0.00 | HPVC1 |
| chr7:63,215,617-63,244,003 | q11.21 | Allelic Imbalance | 28386 | 61.90 | 6.67 | 55.24 | 0.00 | 0.00 |  |
| chr7:25,154,934-25,181,383 | p15.2 | Allelic Imbalance | 26449 | 66.67 | 11.11 | 55.56 | 0.00 | 0.00 | C7orf31 |
| chr7:35,621,552-35,631,918 | p14.2 | Allelic Imbalance | 10366 | 66.67 | 11.11 | 55.56 | 0.00 | 0.00 |  |
| chr7:35,642,220-36,044,727 | p14.2 | Allelic Imbalance | 402507 | 66.67 | 11.11 | 55.56 | 0.00 | 0.00 | HERPUD2, SEPT7 |
| chr7:46,669,696-47,035,963 | p12.3 | Allelic Imbalance | 366267 | 66.67 | 11.11 | 55.56 | 0.00 | 0.00 |  |
| chr7:5,243,488-5,308,230 | p22.1 | Allelic Imbalance | 64742 | 66.67 | 11.11 | 55.56 | 0.00 | 0.00 | SLC29A4 |
| chr7:50,025,684-50,210,831 | p12.2 | Allelic Imbalance | 185147 | 66.67 | 11.11 | 55.56 | 0.00 | 0.00 | ZPBP, C7orf72 |
| chr7:7,554,528-7,654,248 | p21.3 | Allelic Imbalance | 99720 | 66.67 | 11.11 | 55.56 | 0.00 | 0.00 | MIOS, RPA3, LOC729852 |
| chr7:7,812,215-7,816,952 | p21.3 | Allelic Imbalance | 4737 | 66.67 | 11.11 | 55.56 | 0.00 | 0.00 | LOC729852 |
| chr7:8,902,807-9,439,477 | p21.3 | Allelic Imbalance | 536670 | 66.67 | 11.11 | 55.56 | 0.00 | 0.00 |  |
| chr7:9,496,765-9,597,808 | p21.3 | Allelic Imbalance | 101043 | 66.67 | 11.11 | 55.56 | 0.00 | 0.00 |  |
| chr7:2,844,506-2,877,377 | p22.2 | Allelic Imbalance | 32871 | 69.05 | 13.33 | 55.71 | 0.00 | 0.00 | GNA12 |
| chr7:21,749,241-21,769,801 | p15.3 | Allelic Imbalance | 20560 | 69.05 | 13.33 | 55.71 | 0.00 | 0.00 | DNAH11 |
| chr7:24,966,823-24,989,356 | p15.2 | Allelic Imbalance | 22533 | 69.05 | 13.33 | 55.71 | 0.00 | 0.00 | OSBPL3 |
| chr7:25,000,230-25,050,970 | p15.2 | Allelic Imbalance | 50740 | 69.05 | 13.33 | 55.71 | 0.00 | 0.00 |  |
| chr7:25,061,601-25,094,379 | p15.2 | Allelic Imbalance | 32778 | 69.05 | 13.33 | 55.71 | 0.00 | 0.00 |  |
| chr7:26,197,014-26,203,953 | p15.2 | Allelic Imbalance | 6939 | 69.05 | 13.33 | 55.71 | 0.00 | 0.00 | HNRNPA2B1 |
| chr7:29,075,563-29,098,378 | p15.1 | Allelic Imbalance | 22815 | 69.05 | 13.33 | 55.71 | 0.00 | 0.00 | CPVL |
| chr7:29,282,391-29,296,387 | p15.1 | Allelic Imbalance | 13996 | 69.05 | 13.33 | 55.71 | 0.00 | 0.00 | CHN2 |
| chr7:34,663,902-34,732,532 | p14.3 | Allelic Imbalance | 68630 | 69.05 | 13.33 | 55.71 | 0.00 | 0.00 | NPSR1-AS1, NPSR1 |
| chr7:36,323,487-36,469,984 | p14.2 | Allelic Imbalance | 146497 | 69.05 | 13.33 | 55.71 | 0.00 | 0.00 | KIAA0895, ANLN |
| chr7:4,764,833-5,230,208 | p22.1 | Allelic Imbalance | 465375 | 69.05 | 13.33 | 55.71 | 0.00 | 0.00 | FOXK1, AP5Z1, MIR4656, RADIL, PAPOLB, MMD2, RNF216P1, RBAK, RBAK-LOC389458, LOC389458, ZNF890P, WIPI2 |
| chr7:46,315,756-46,433,290 | p13 | Allelic Imbalance | 117534 | 69.05 | 13.33 | 55.71 | 0.00 | 0.00 |  |
| chr7:48,967,936-49,014,545 | p12.3 | Allelic Imbalance | 46609 | 69.05 | 13.33 | 55.71 | 0.00 | 0.00 |  |
| chr7:49,824,191-49,962,198 | p12.2 | Allelic Imbalance | 138007 | 69.05 | 13.33 | 55.71 | 0.00 | 0.00 | VWC2, ZPBP |
| chr7:52,718,691-52,733,961 | p12.1 | Allelic Imbalance | 15270 | 64.29 | 8.89 | 55.40 | 0.00 | 0.00 |  |
| chr7:53,476,633-53,625,020 | p12.1 | Allelic Imbalance | 148387 | 64.29 | 8.89 | 55.40 | 0.00 | 0.00 |  |
| chr7:6,396,240-6,535,396 | p22.1 | Allelic Imbalance | 139156 | 64.29 | 8.89 | 55.40 | 0.00 | 0.00 | RAC1, DAGLB, KDELR2, GRID2IP |
| chr7:6,576,544-6,616,394 | p22.1 | Allelic Imbalance | 39850 | 64.29 | 8.89 | 55.40 | 0.00 | 0.00 | ZDHHC4, C7orf26 |
| chr7:63,292,700-63,296,496 | q11.21 | Allelic Imbalance | 3796 | 64.29 | 8.89 | 55.40 | 0.00 | 0.00 |  |
| chr7:63,723,844-63,746,613 | q11.21 | Allelic Imbalance | 22769 | 64.29 | 8.89 | 55.40 | 0.00 | 0.00 |  |
| chr7:66,248,419-66,406,553 | q11.22 | Allelic Imbalance | 158134 | 69.05 | 13.33 | 55.71 | 0.00 | 0.00 | TYW1, PMS2P4, STAG3L4 |
| chr7:7,387,070-7,482,373 | p21.3 | Allelic Imbalance | 95303 | 64.29 | 8.89 | 55.40 | 0.00 | 0.00 | COL28A1 |
| chr7:8,548,176-8,694,332 | p21.3 | Allelic Imbalance | 146156 | 69.05 | 13.33 | 55.71 | 0.00 | 0.00 | NXPH1 |
| chr7:18,679,615-18,713,198 | p21.1 | Allelic Imbalance | 33583 | 71.43 | 15.56 | 55.87 | 0.00 | 0.00 | HDAC9 |
| chr7:18,944,645-18,965,594 | p21.1 | Allelic Imbalance | 20949 | 71.43 | 15.56 | 55.87 | 0.00 | 0.00 | HDAC9 |
| chr7:20,703,206-20,740,297 | p15.3 | Allelic Imbalance | 37091 | 71.43 | 15.56 | 55.87 | 0.00 | 0.00 | ABCB5 |
| chr7:20,914,841-21,032,513 | p15.3 | Allelic Imbalance | 117672 | 71.43 | 15.56 | 55.87 | 0.00 | 0.00 |  |
| chr7:29,031,858-29,067,262 | p15.1 | Allelic Imbalance | 35404 | 71.43 | 15.56 | 55.87 | 0.00 | 0.00 | CPVL |
| chr7:29,121,047-29,125,965 | p15.1 | Allelic Imbalance | 4918 | 71.43 | 15.56 | 55.87 | 0.00 | 0.00 | CPVL |
| chr7:29,151,713-29,164,187 | p15.1 | Allelic Imbalance | 12474 | 71.43 | 15.56 | 55.87 | 0.00 | 0.00 | CPVL |
| chr7:33,633,403-33,659,108 | p14.3 | Allelic Imbalance | 25705 | 71.43 | 15.56 | 55.87 | 0.00 | 0.00 |  |
| chr7:34,111,273-34,342,737 | p14.3 | Allelic Imbalance | 231464 | 71.43 | 15.56 | 55.87 | 0.00 | 0.00 | BMPER |
| chr7:34,848,935-35,358,042 | p14.3 | Allelic Imbalance | 509107 | 71.43 | 15.56 | 55.87 | 0.00 | 0.00 | NPSR1, DPY19L1, DPY19L2P1, TBX20, LOC401324 |
| chr7:35,370,075-35,399,685 | p14.3 | Allelic Imbalance | 29610 | 71.43 | 15.56 | 55.87 | 0.00 | 0.00 | LOC401324 |
| chr7:42,140,857-42,606,969 | p14.1 | Allelic Imbalance | 466112 | 71.43 | 15.56 | 55.87 | 0.00 | 0.00 | GLI3 |
| chr7:47,287,659-47,436,662 | p12.3 | Allelic Imbalance | 149003 | 71.43 | 15.56 | 55.87 | 0.00 | 0.00 | TNS3 |
| chr7:49,235,117-49,547,609 | p12.3 | Allelic Imbalance | 312492 | 71.43 | 15.56 | 55.87 | 0.00 | 0.00 |  |
| chr7:54,283,958-54,357,468 | p11.2 | Allelic Imbalance | 73510 | 59.52 | 6.67 | 52.86 | 0.00 | 0.00 |  |
| chr7:6,339,685-6,385,764 | p22.1 | Allelic Imbalance | 46079 | 59.52 | 6.67 | 52.86 | 0.00 | 0.00 | FAM220A, RAC1 |
| chr7:62,726,391-63,215,617 | q11.21 | Allelic Imbalance | 489226 | 59.52 | 6.67 | 52.86 | 0.00 | 0.00 | LOC100506050, ZNF727 |
| chr7:18,592,389-18,598,841 | p21.1 | Allelic Imbalance | 6452 | 73.81 | 17.78 | 56.03 | 0.00 | 0.00 | HDAC9 |
| chr7:21,820,890-21,913,073 | p15.3 | Allelic Imbalance | 92183 | 73.81 | 17.78 | 56.03 | 0.00 | 0.00 | DNAH11, CDCA7L |
| chr7:21,922,352-21,938,425 | p15.3 | Allelic Imbalance | 16073 | 73.81 | 17.78 | 56.03 | 0.00 | 0.00 | CDCA7L |
| chr7:31,134,287-31,234,529 | p15.1 | Allelic Imbalance | 100242 | 73.81 | 17.78 | 56.03 | 0.00 | 0.00 |  |
| chr7:24,414,785-24,431,062 | p15.3 | Allelic Imbalance | 16277 | 76.19 | 20.00 | 56.19 | 0.00 | 0.00 |  |
| chr7:31,493,306-31,524,007 | p15.1 | Allelic Imbalance | 30701 | 76.19 | 20.00 | 56.19 | 0.00 | 0.00 | CCDC129 |
| chr7:15,716,427-15,723,726 | p21.1 | Allelic Imbalance | 7299 | 64.29 | 11.11 | 53.17 | 0.00 | 0.00 |  |
| chr7:25,181,383-25,238,604 | p15.2 | Allelic Imbalance | 57221 | 64.29 | 11.11 | 53.17 | 0.00 | 0.00 | C7orf31, NPVF |
| chr7:29,431,434-29,443,697 | p15.1 | Allelic Imbalance | 12263 | 64.29 | 11.11 | 53.17 | 0.00 | 0.00 | CHN2 |
| chr7:38,136,443-38,182,749 | p14.1 | Allelic Imbalance | 46306 | 64.29 | 11.11 | 53.17 | 0.00 | 0.00 |  |
| chr7:5,308,230-5,483,402 | p22.1 | Allelic Imbalance | 175172 | 64.29 | 11.11 | 53.17 | 0.00 | 0.00 | SLC29A4, TNRC18, FBXL18 |
| chr7:50,210,831-50,288,030 | p12.2 | Allelic Imbalance | 77199 | 64.29 | 11.11 | 53.17 | 0.00 | 0.00 |  |
| chr7:51,721,578-51,916,117 | p12.1 | Allelic Imbalance | 194539 | 64.29 | 11.11 | 53.17 | 0.00 | 0.00 |  |
| chr7:63,746,613-63,777,126 | q11.21 | Allelic Imbalance | 30513 | 64.29 | 11.11 | 53.17 | 0.00 | 0.00 | ZNF107 |
| chr7:7,482,373-7,554,528 | p21.3 | Allelic Imbalance | 72155 | 64.29 | 11.11 | 53.17 | 0.00 | 0.00 | COL28A1 |
| chr7:9,439,477-9,496,765 | p21.3 | Allelic Imbalance | 57288 | 64.29 | 11.11 | 53.17 | 0.00 | 0.00 |  |
| chr7:37,913,658-37,954,700 | p14.1 | Allelic Imbalance | 41042 | 61.90 | 8.89 | 53.02 | 0.00 | 0.00 | SFRP4, EPDR1 |
| chr7:50,334,750-50,398,028 | p12.2 | Allelic Imbalance | 63278 | 61.90 | 8.89 | 53.02 | 0.00 | 0.00 | IKZF1 |
| chr7:52,623,829-52,718,691 | p12.1 | Allelic Imbalance | 94862 | 61.90 | 8.89 | 53.02 | 0.00 | 0.00 |  |
| chr7:53,625,020-53,632,163 | p12.1 | Allelic Imbalance | 7143 | 61.90 | 8.89 | 53.02 | 0.00 | 0.00 |  |
| chr7:53,643,162-53,693,063 | p12.1 | Allelic Imbalance | 49901 | 61.90 | 8.89 | 53.02 | 0.00 | 0.00 | FLJ45974 |
| chr7:53,698,754-53,791,295 | p12.1 | Allelic Imbalance | 92541 | 61.90 | 8.89 | 53.02 | 0.00 | 0.00 | FLJ45974 |
| chr7:57,260,703-57,358,655 | p11.2 | Allelic Imbalance | 97952 | 61.90 | 8.89 | 53.02 | 0.00 | 0.00 |  |
| chr7:6,391,673-6,396,240 | p22.1 | Allelic Imbalance | 4567 | 61.90 | 8.89 | 53.02 | 0.00 | 0.00 | RAC1 |
| chr7:63,296,496-63,723,844 | q11.21 | Allelic Imbalance | 427348 | 61.90 | 8.89 | 53.02 | 0.00 | 0.00 | ZNF735, ZNF679, ZNF736, LOC649395, ZNF680, LOC641746 |
| chr7:15,172,308-15,178,367 | p21.2 | Allelic Imbalance | 6059 | 66.67 | 13.33 | 53.33 | 0.00 | 0.00 |  |
| chr7:2,812,958-2,844,506 | p22.2 | Allelic Imbalance | 31548 | 66.67 | 13.33 | 53.33 | 0.00 | 0.00 | GNA12 |
| chr7:2,877,377-2,910,528 | p22.2 | Allelic Imbalance | 33151 | 66.67 | 13.33 | 53.33 | 0.00 | 0.00 |  |
| chr7:21,623,206-21,702,340 | p15.3 | Allelic Imbalance | 79134 | 66.67 | 13.33 | 53.33 | 0.00 | 0.00 | DNAH11 |
| chr7:21,716,747-21,749,241 | p15.3 | Allelic Imbalance | 32494 | 66.67 | 13.33 | 53.33 | 0.00 | 0.00 | DNAH11 |
| chr7:24,989,356-25,000,230 | p15.2 | Allelic Imbalance | 10874 | 66.67 | 13.33 | 53.33 | 0.00 | 0.00 |  |
| chr7:25,050,970-25,061,601 | p15.2 | Allelic Imbalance | 10631 | 66.67 | 13.33 | 53.33 | 0.00 | 0.00 |  |
| chr7:29,296,387-29,309,599 | p15.1 | Allelic Imbalance | 13212 | 66.67 | 13.33 | 53.33 | 0.00 | 0.00 | CHN2 |
| chr7:29,453,408-29,474,745 | p15.1 | Allelic Imbalance | 21337 | 66.67 | 13.33 | 53.33 | 0.00 | 0.00 | CHN2 |
| chr7:3,802,708-4,162,560 | p22.2 | Allelic Imbalance | 359852 | 66.67 | 13.33 | 53.33 | 0.00 | 0.00 | SDK1 |
| chr7:32,876,195-32,908,569 | p14.3 | Allelic Imbalance | 32374 | 66.67 | 13.33 | 53.33 | 0.00 | 0.00 | KBTBD2 |
| chr7:32,929,404-33,021,450 | p14.3 | Allelic Imbalance | 92046 | 66.67 | 13.33 | 53.33 | 0.00 | 0.00 | RP9P, FKBP9, NT5C3 |
| chr7:35,476,174-35,621,552 | p14.3 - p14.2 | Allelic Imbalance | 145378 | 66.67 | 13.33 | 53.33 | 0.00 | 0.00 |  |
| chr7:36,044,727-36,080,777 | p14.2 | Allelic Imbalance | 36050 | 66.67 | 13.33 | 53.33 | 0.00 | 0.00 |  |
| chr7:36,101,572-36,112,227 | p14.2 | Allelic Imbalance | 10655 | 66.67 | 13.33 | 53.33 | 0.00 | 0.00 |  |
| chr7:36,585,980-36,588,880 | p14.2 | Allelic Imbalance | 2900 | 66.67 | 13.33 | 53.33 | 0.00 | 0.00 | AOAH |
| chr7:37,355,603-37,439,064 | p14.2 | Allelic Imbalance | 83461 | 66.67 | 13.33 | 53.33 | 0.00 | 0.00 | ELMO1 |
| chr7:38,304,265-38,325,095 | p14.1 | Allelic Imbalance | 20830 | 66.67 | 13.33 | 53.33 | 0.00 | 0.00 |  |
| chr7:4,750,577-4,764,833 | p22.1 | Allelic Imbalance | 14256 | 66.67 | 13.33 | 53.33 | 0.00 | 0.00 | FOXK1 |
| chr7:41,384,428-41,625,459 | p14.1 | Allelic Imbalance | 241031 | 66.67 | 13.33 | 53.33 | 0.00 | 0.00 |  |
| chr7:46,254,227-46,315,756 | p13 | Allelic Imbalance | 61529 | 66.67 | 13.33 | 53.33 | 0.00 | 0.00 |  |
| chr7:49,962,198-50,025,684 | p12.2 | Allelic Imbalance | 63486 | 66.67 | 13.33 | 53.33 | 0.00 | 0.00 | ZPBP |
| chr7:66,221,827-66,248,419 | q11.22 | Allelic Imbalance | 26592 | 66.67 | 13.33 | 53.33 | 0.00 | 0.00 | TYW1 |
| chr7:9,597,808-9,623,824 | p21.3 | Allelic Imbalance | 26016 | 66.67 | 13.33 | 53.33 | 0.00 | 0.00 |  |
| chr7:1,930,494-2,201,310 | p22.3 - p22.2 | Allelic Imbalance | 270816 | 69.05 | 15.56 | 53.49 | 0.00 | 0.00 | MAD1L1 |
| chr7:13,428,855-13,439,770 | p21.2 | Allelic Imbalance | 10915 | 69.05 | 15.56 | 53.49 | 0.00 | 0.00 |  |
| chr7:18,965,594-18,988,397 | p21.1 | Allelic Imbalance | 22803 | 69.05 | 15.56 | 53.49 | 0.00 | 0.00 | HDAC9 |
| chr7:18,994,724-19,298,320 | p21.1 | Allelic Imbalance | 303596 | 69.05 | 15.56 | 53.49 | 0.00 | 0.00 | HDAC9, TWIST1, FERD3L |
| chr7:20,027,858-20,439,334 | p15.3 | Allelic Imbalance | 411476 | 69.05 | 15.56 | 53.49 | 0.00 | 0.00 | MACC1-AS1, MACC1, ITGB8 |
| chr7:20,447,312-20,703,206 | p15.3 | Allelic Imbalance | 255894 | 69.05 | 15.56 | 53.49 | 0.00 | 0.00 | ABCB5 |
| chr7:21,032,513-21,055,966 | p15.3 | Allelic Imbalance | 23453 | 69.05 | 15.56 | 53.49 | 0.00 | 0.00 |  |
| chr7:24,944,241-24,966,823 | p15.2 | Allelic Imbalance | 22582 | 69.05 | 15.56 | 53.49 | 0.00 | 0.00 | OSBPL3 |
| chr7:26,106,311-26,121,322 | p15.2 | Allelic Imbalance | 15011 | 69.05 | 15.56 | 53.49 | 0.00 | 0.00 |  |
| chr7:26,195,558-26,197,014 | p15.2 | Allelic Imbalance | 1456 | 69.05 | 15.56 | 53.49 | 0.00 | 0.00 | HNRNPA2B1 |
| chr7:26,203,953-26,548,204 | p15.2 | Allelic Imbalance | 344251 | 69.05 | 15.56 | 53.49 | 0.00 | 0.00 | HNRNPA2B1, CBX3, SNX10, LOC441204, KIAA0087 |
| chr7:28,693,233-29,008,608 | p15.1 | Allelic Imbalance | 315375 | 69.05 | 15.56 | 53.49 | 0.00 | 0.00 | CREB5, TRIL, LOC100506497, CPVL |
| chr7:29,016,810-29,031,858 | p15.1 | Allelic Imbalance | 15048 | 69.05 | 15.56 | 53.49 | 0.00 | 0.00 | LOC100506497, CPVL |
| chr7:33,568,258-33,633,403 | p14.3 | Allelic Imbalance | 65145 | 69.05 | 15.56 | 53.49 | 0.00 | 0.00 | BBS9 |
| chr7:33,659,108-33,716,469 | p14.3 | Allelic Imbalance | 57361 | 69.05 | 15.56 | 53.49 | 0.00 | 0.00 |  |
| chr7:34,095,861-34,111,273 | p14.3 | Allelic Imbalance | 15412 | 69.05 | 15.56 | 53.49 | 0.00 | 0.00 | BMPER |
| chr7:35,399,685-35,461,849 | p14.3 | Allelic Imbalance | 62164 | 69.05 | 15.56 | 53.49 | 0.00 | 0.00 |  |
| chr7:36,305,571-36,323,487 | p14.2 | Allelic Imbalance | 17916 | 69.05 | 15.56 | 53.49 | 0.00 | 0.00 | EEPD1 |
| chr7:42,094,647-42,140,857 | p14.1 | Allelic Imbalance | 46210 | 69.05 | 15.56 | 53.49 | 0.00 | 0.00 | GLI3 |
| chr7:42,606,969-42,837,425 | p14.1 | Allelic Imbalance | 230456 | 69.05 | 15.56 | 53.49 | 0.00 | 0.00 |  |
| chr7:45,704,814-45,722,719 | p13 | Allelic Imbalance | 17905 | 69.05 | 15.56 | 53.49 | 0.00 | 0.00 | ADCY1 |
| chr7:47,436,662-47,791,935 | p12.3 | Allelic Imbalance | 355273 | 69.05 | 15.56 | 53.49 | 0.00 | 0.00 | TNS3, C7orf65, LINC00525, PKD1L1 |
| chr7:48,698,567-48,967,936 | p12.3 | Allelic Imbalance | 269369 | 69.05 | 15.56 | 53.49 | 0.00 | 0.00 | CDC14C |
| chr7:5,702,238-5,864,043 | p22.1 | Allelic Imbalance | 161805 | 57.14 | 6.67 | 50.48 | 0.00 | 0.00 | RNF216, ZNF815P |
| chr7:54,357,468-54,446,322 | p11.2 | Allelic Imbalance | 88854 | 57.14 | 6.67 | 50.48 | 0.00 | 0.00 |  |
| chr7:56,153,386-56,190,951 | p11.2 | Allelic Imbalance | 37565 | 57.14 | 6.67 | 50.48 | 0.00 | 0.00 |  |
| chr7:56,517,621-56,548,662 | p11.2 | Allelic Imbalance | 31041 | 57.14 | 6.67 | 50.48 | 0.00 | 0.00 | DKFZp434L192 |
| chr7:6,106,496-6,339,685 | p22.1 | Allelic Imbalance | 233189 | 57.14 | 6.67 | 50.48 | 0.00 | 0.00 | USP42, CYTH3, FAM220A |
| chr7:66,406,553-66,432,187 | q11.22 | Allelic Imbalance | 25634 | 69.05 | 15.56 | 53.49 | 0.00 | 0.00 | STAG3L4 |
| chr7:1,091,341-1,170,818 | p22.3 | Allelic Imbalance | 79477 | 71.43 | 17.78 | 53.65 | 0.00 | 0.00 | C7orf50, GPER, ZFAND2A |
| chr7:18,598,841-18,679,615 | p21.1 | Allelic Imbalance | 80774 | 71.43 | 17.78 | 53.65 | 0.00 | 0.00 | HDAC9 |
| chr7:21,938,425-22,167,895 | p15.3 | Allelic Imbalance | 229470 | 71.43 | 17.78 | 53.65 | 0.00 | 0.00 | CDCA7L, RAPGEF5 |
| chr7:29,125,965-29,151,713 | p15.1 | Allelic Imbalance | 25748 | 71.43 | 17.78 | 53.65 | 0.00 | 0.00 | CPVL |
| chr7:31,104,374-31,134,287 | p15.1 | Allelic Imbalance | 29913 | 71.43 | 17.78 | 53.65 | 0.00 | 0.00 | ADCYAP1R1 |
| chr7:18,585,006-18,592,389 | p21.1 | Allelic Imbalance | 7383 | 73.81 | 20.00 | 53.81 | 0.00 | 0.00 | HDAC9 |
| chr7:24,397,177-24,414,785 | p15.3 | Allelic Imbalance | 17608 | 73.81 | 20.00 | 53.81 | 0.00 | 0.00 |  |
| chr7:24,431,062-24,441,276 | p15.3 | Allelic Imbalance | 10214 | 73.81 | 20.00 | 53.81 | 0.00 | 0.00 |  |
| chr7:31,234,529-31,493,306 | p15.1 | Allelic Imbalance | 258777 | 73.81 | 20.00 | 53.81 | 0.00 | 0.00 | NEUROD6 |
| chr7:31,524,007-31,559,974 | p15.1 | Allelic Imbalance | 35967 | 76.19 | 22.22 | 53.97 | 0.00 | 0.00 | CCDC129 |
| chr7:50,398,028-50,421,269 | p12.2 | Allelic Imbalance | 23241 | 59.52 | 8.89 | 50.63 | 0.00 | 0.00 | IKZF1 |
| chr7:50,778,322-50,800,982 | p12.2 | Allelic Imbalance | 22660 | 59.52 | 8.89 | 50.63 | 0.00 | 0.00 | GRB10 |
| chr7:52,197,376-52,258,119 | p12.1 | Allelic Imbalance | 60743 | 59.52 | 8.89 | 50.63 | 0.00 | 0.00 |  |
| chr7:52,608,872-52,623,829 | p12.1 | Allelic Imbalance | 14957 | 59.52 | 8.89 | 50.63 | 0.00 | 0.00 |  |
| chr7:53,632,163-53,643,162 | p12.1 | Allelic Imbalance | 10999 | 59.52 | 8.89 | 50.63 | 0.00 | 0.00 |  |
| chr7:53,693,063-53,698,754 | p12.1 | Allelic Imbalance | 5691 | 59.52 | 8.89 | 50.63 | 0.00 | 0.00 | FLJ45974 |
| chr7:57,358,655-57,369,389 | p11.2 | Allelic Imbalance | 10734 | 59.52 | 8.89 | 50.63 | 0.00 | 0.00 |  |
| chr7:57,459,937-58,014,651 | p11.1 | Allelic Imbalance | 554714 | 59.52 | 8.89 | 50.63 | 0.00 | 0.00 | MIR3147, ZNF716 |
| chr7:6,385,764-6,391,673 | p22.1 | Allelic Imbalance | 5909 | 59.52 | 8.89 | 50.63 | 0.00 | 0.00 | RAC1 |
| chr7:62,312,983-62,331,635 | q11.21 | Allelic Imbalance | 18652 | 59.52 | 8.89 | 50.63 | 0.00 | 0.00 |  |
| chr7:62,475,979-62,726,391 | q11.21 | Allelic Imbalance | 250412 | 59.52 | 8.89 | 50.63 | 0.00 | 0.00 | LOC100287834, MIR4283-1, MIR4283-2 |
| chr7:64,549,625-64,698,576 | q11.21 | Allelic Imbalance | 148951 | 59.52 | 8.89 | 50.63 | 0.00 | 0.00 |  |
| chr7:15,087,638-15,172,308 | p21.2 | Allelic Imbalance | 84670 | 64.29 | 13.33 | 50.95 | 0.00 | 0.00 |  |
| chr7:15,178,367-15,343,262 | p21.2 - p21.1 | Allelic Imbalance | 164895 | 64.29 | 13.33 | 50.95 | 0.00 | 0.00 | AGMO |
| chr7:15,650,138-15,673,961 | p21.1 | Allelic Imbalance | 23823 | 64.29 | 13.33 | 50.95 | 0.00 | 0.00 | MEOX2 |
| chr7:19,762,917-19,764,808 | p15.3 | Allelic Imbalance | 1891 | 64.29 | 13.33 | 50.95 | 0.00 | 0.00 | TMEM196 |
| chr7:19,971,519-20,013,365 | p15.3 | Allelic Imbalance | 41846 | 64.29 | 13.33 | 50.95 | 0.00 | 0.00 |  |
| chr7:2,484,901-2,812,958 | p22.2 | Allelic Imbalance | 328057 | 64.29 | 13.33 | 50.95 | 0.00 | 0.00 | LFNG, MIR4648, BRAT1, IQCE, TTYH3, AMZ1, GNA12 |
| chr7:2,910,528-2,938,309 | p22.2 | Allelic Imbalance | 27781 | 64.29 | 13.33 | 50.95 | 0.00 | 0.00 | CARD11 |
| chr7:21,586,433-21,623,206 | p15.3 | Allelic Imbalance | 36773 | 64.29 | 13.33 | 50.95 | 0.00 | 0.00 | DNAH11 |
| chr7:21,702,340-21,716,747 | p15.3 | Allelic Imbalance | 14407 | 64.29 | 13.33 | 50.95 | 0.00 | 0.00 | DNAH11 |
| chr7:25,238,604-25,258,723 | p15.2 | Allelic Imbalance | 20119 | 64.29 | 13.33 | 50.95 | 0.00 | 0.00 |  |
| chr7:29,309,599-29,362,209 | p15.1 | Allelic Imbalance | 52610 | 64.29 | 13.33 | 50.95 | 0.00 | 0.00 | CHN2 |
| chr7:29,424,668-29,431,434 | p15.1 | Allelic Imbalance | 6766 | 64.29 | 13.33 | 50.95 | 0.00 | 0.00 | CHN2 |
| chr7:29,443,697-29,453,408 | p15.1 | Allelic Imbalance | 9711 | 64.29 | 13.33 | 50.95 | 0.00 | 0.00 | CHN2 |
| chr7:3,644,011-3,802,708 | p22.2 | Allelic Imbalance | 158697 | 64.29 | 13.33 | 50.95 | 0.00 | 0.00 | SDK1 |
| chr7:32,864,610-32,876,195 | p14.3 | Allelic Imbalance | 11585 | 64.29 | 13.33 | 50.95 | 0.00 | 0.00 | KBTBD2 |
| chr7:33,021,450-33,272,592 | p14.3 | Allelic Imbalance | 251142 | 64.29 | 13.33 | 50.95 | 0.00 | 0.00 | NT5C3, RP9, BBS9 |
| chr7:36,080,777-36,101,572 | p14.2 | Allelic Imbalance | 20795 | 64.29 | 13.33 | 50.95 | 0.00 | 0.00 |  |
| chr7:36,588,880-36,602,479 | p14.2 | Allelic Imbalance | 13599 | 64.29 | 13.33 | 50.95 | 0.00 | 0.00 | AOAH |
| chr7:36,735,144-36,753,789 | p14.2 | Allelic Imbalance | 18645 | 64.29 | 13.33 | 50.95 | 0.00 | 0.00 |  |
| chr7:37,352,230-37,355,603 | p14.2 | Allelic Imbalance | 3373 | 64.29 | 13.33 | 50.95 | 0.00 | 0.00 | ELMO1 |
| chr7:37,439,064-37,471,590 | p14.2 | Allelic Imbalance | 32526 | 64.29 | 13.33 | 50.95 | 0.00 | 0.00 | ELMO1 |
| chr7:38,182,749-38,304,265 | p14.1 | Allelic Imbalance | 121516 | 64.29 | 13.33 | 50.95 | 0.00 | 0.00 | STARD3NL, TARP |
| chr7:38,325,095-38,327,615 | p14.1 | Allelic Imbalance | 2520 | 64.29 | 13.33 | 50.95 | 0.00 | 0.00 |  |
| chr7:39,294,627-39,327,967 | p14.1 | Allelic Imbalance | 33340 | 64.29 | 13.33 | 50.95 | 0.00 | 0.00 | POU6F2 |
| chr7:4,162,560-4,172,579 | p22.2 | Allelic Imbalance | 10019 | 64.29 | 13.33 | 50.95 | 0.00 | 0.00 | SDK1 |
| chr7:40,707,728-40,837,206 | p14.1 | Allelic Imbalance | 129478 | 64.29 | 13.33 | 50.95 | 0.00 | 0.00 | C7orf10 |
| chr7:41,351,690-41,384,428 | p14.1 | Allelic Imbalance | 32738 | 64.29 | 13.33 | 50.95 | 0.00 | 0.00 |  |
| chr7:41,625,459-41,706,426 | p14.1 | Allelic Imbalance | 80967 | 64.29 | 13.33 | 50.95 | 0.00 | 0.00 | INHBA, INHBA-AS1 |
| chr7:41,730,121-41,837,475 | p14.1 | Allelic Imbalance | 107354 | 64.29 | 13.33 | 50.95 | 0.00 | 0.00 | INHBA-AS1 |
| chr7:46,005,076-46,254,227 | p13 | Allelic Imbalance | 249151 | 64.29 | 13.33 | 50.95 | 0.00 | 0.00 |  |
| chr7:47,903,633-47,961,637 | p12.3 | Allelic Imbalance | 58004 | 64.29 | 13.33 | 50.95 | 0.00 | 0.00 | PKD1L1 |
| chr7:50,877,606-50,898,976 | p12.2 | Allelic Imbalance | 21370 | 64.29 | 13.33 | 50.95 | 0.00 | 0.00 |  |
| chr7:66,133,800-66,221,827 | q11.22 | Allelic Imbalance | 88027 | 64.29 | 13.33 | 50.95 | 0.00 | 0.00 | MIR4650-1, MIR4650-2, TYW1 |
| chr7:9,623,824-9,669,483 | p21.3 | Allelic Imbalance | 45659 | 64.29 | 13.33 | 50.95 | 0.00 | 0.00 | PER4 |
| chr7:5,864,043-6,002,537 | p22.1 | Allelic Imbalance | 138494 | 54.76 | 6.67 | 48.10 | 0.00 | 0.00 | OCM, CCZ1, RSPH10B, RSPH10B2, PMS2 |
| chr7:50,620,657-50,626,309 | p12.2 | Allelic Imbalance | 5652 | 54.76 | 6.67 | 48.10 | 0.00 | 0.00 | GRB10 |
| chr7:56,146,881-56,153,386 | p11.2 | Allelic Imbalance | 6505 | 54.76 | 6.67 | 48.10 | 0.00 | 0.00 | NUPR1L |
| chr7:56,190,951-56,215,192 | p11.2 | Allelic Imbalance | 24241 | 54.76 | 6.67 | 48.10 | 0.00 | 0.00 |  |
| chr7:56,263,476-56,517,621 | p11.2 | Allelic Imbalance | 254145 | 54.76 | 6.67 | 48.10 | 0.00 | 0.00 | LOC650226 |
| chr7:6,011,314-6,012,880 | p22.1 | Allelic Imbalance | 1566 | 54.76 | 6.67 | 48.10 | 0.00 | 0.00 | PMS2 |
| chr7:6,019,436-6,106,496 | p22.1 | Allelic Imbalance | 87060 | 54.76 | 6.67 | 48.10 | 0.00 | 0.00 | AIMP2, ANKRD61, EIF2AK1 |
| chr7:1,659,595-1,930,494 | p22.3 | Allelic Imbalance | 270899 | 66.67 | 15.56 | 51.11 | 0.00 | 0.00 | ELFN1, MIR4655, MAD1L1 |
| chr7:13,327,924-13,428,855 | p21.2 | Allelic Imbalance | 100931 | 66.67 | 15.56 | 51.11 | 0.00 | 0.00 |  |
| chr7:13,439,770-13,650,153 | p21.2 | Allelic Imbalance | 210383 | 66.67 | 15.56 | 51.11 | 0.00 | 0.00 |  |
| chr7:13,842,133-13,868,052 | p21.2 | Allelic Imbalance | 25919 | 66.67 | 15.56 | 51.11 | 0.00 | 0.00 |  |
| chr7:15,685,744-15,716,427 | p21.1 | Allelic Imbalance | 30683 | 61.90 | 11.11 | 50.79 | 0.00 | 0.00 | MEOX2 |
| chr7:15,723,726-15,725,078 | p21.1 | Allelic Imbalance | 1352 | 61.90 | 11.11 | 50.79 | 0.00 | 0.00 |  |
| chr7:16,620,664-16,718,357 | p21.1 | Allelic Imbalance | 97693 | 66.67 | 15.56 | 51.11 | 0.00 | 0.00 | ANKMY2, BZW2 |
| chr7:18,988,397-18,994,724 | p21.1 | Allelic Imbalance | 6327 | 66.67 | 15.56 | 51.11 | 0.00 | 0.00 | HDAC9 |
| chr7:19,298,320-19,312,666 | p21.1 | Allelic Imbalance | 14346 | 66.67 | 15.56 | 51.11 | 0.00 | 0.00 |  |
| chr7:2,201,310-2,336,756 | p22.2 | Allelic Imbalance | 135446 | 66.67 | 15.56 | 51.11 | 0.00 | 0.00 | MAD1L1, FTSJ2, NUDT1, SNX8 |
| chr7:20,439,334-20,447,312 | p15.3 | Allelic Imbalance | 7978 | 66.67 | 15.56 | 51.11 | 0.00 | 0.00 |  |
| chr7:21,055,966-21,109,118 | p15.3 | Allelic Imbalance | 53152 | 66.67 | 15.56 | 51.11 | 0.00 | 0.00 |  |
| chr7:21,555,192-21,586,433 | p15.3 | Allelic Imbalance | 31241 | 61.90 | 11.11 | 50.79 | 0.00 | 0.00 | DNAH11 |
| chr7:22,273,812-22,293,192 | p15.3 | Allelic Imbalance | 19380 | 66.67 | 15.56 | 51.11 | 0.00 | 0.00 | RAPGEF5 |
| chr7:22,304,758-22,321,997 | p15.3 | Allelic Imbalance | 17239 | 66.67 | 15.56 | 51.11 | 0.00 | 0.00 | RAPGEF5 |
| chr7:25,403,520-25,436,977 | p15.2 | Allelic Imbalance | 33457 | 66.67 | 15.56 | 51.11 | 0.00 | 0.00 |  |
| chr7:25,497,347-26,106,311 | p15.2 | Allelic Imbalance | 608964 | 66.67 | 15.56 | 51.11 | 0.00 | 0.00 | MIR148A |
| chr7:26,548,204-26,609,989 | p15.2 | Allelic Imbalance | 61785 | 66.67 | 15.56 | 51.11 | 0.00 | 0.00 |  |
| chr7:29,008,608-29,016,810 | p15.1 | Allelic Imbalance | 8202 | 66.67 | 15.56 | 51.11 | 0.00 | 0.00 | LOC100506497, CPVL |
| chr7:3,323,991-3,432,262 | p22.2 | Allelic Imbalance | 108271 | 61.90 | 11.11 | 50.79 | 0.00 | 0.00 | SDK1 |
| chr7:33,537,301-33,568,258 | p14.3 | Allelic Imbalance | 30957 | 66.67 | 15.56 | 51.11 | 0.00 | 0.00 | BBS9 |
| chr7:33,716,469-33,842,336 | p14.3 | Allelic Imbalance | 125867 | 66.67 | 15.56 | 51.11 | 0.00 | 0.00 |  |
| chr7:33,949,655-34,095,861 | p14.3 | Allelic Imbalance | 146206 | 66.67 | 15.56 | 51.11 | 0.00 | 0.00 | BMPER |
| chr7:35,461,849-35,476,174 | p14.3 | Allelic Imbalance | 14325 | 66.67 | 15.56 | 51.11 | 0.00 | 0.00 |  |
| chr7:36,112,227-36,305,571 | p14.2 | Allelic Imbalance | 193344 | 66.67 | 15.56 | 51.11 | 0.00 | 0.00 | EEPD1 |
| chr7:37,471,590-37,495,760 | p14.2 | Allelic Imbalance | 24170 | 61.90 | 11.11 | 50.79 | 0.00 | 0.00 |  |
| chr7:37,521,701-37,673,322 | p14.1 | Allelic Imbalance | 151621 | 61.90 | 11.11 | 50.79 | 0.00 | 0.00 |  |
| chr7:37,899,096-37,913,658 | p14.1 | Allelic Imbalance | 14562 | 61.90 | 11.11 | 50.79 | 0.00 | 0.00 | NME8, SFRP4 |
| chr7:37,954,700-38,136,443 | p14.1 | Allelic Imbalance | 181743 | 61.90 | 11.11 | 50.79 | 0.00 | 0.00 | EPDR1 |
| chr7:38,809,423-38,910,686 | p14.1 | Allelic Imbalance | 101263 | 66.67 | 15.56 | 51.11 | 0.00 | 0.00 | VPS41 |
| chr7:4,239,764-4,750,577 | p22.2 - p22.1 | Allelic Imbalance | 510813 | 66.67 | 15.56 | 51.11 | 0.00 | 0.00 | SDK1, FOXK1 |
| chr7:42,051,755-42,094,647 | p14.1 | Allelic Imbalance | 42892 | 66.67 | 15.56 | 51.11 | 0.00 | 0.00 | GLI3 |
| chr7:42,837,425-42,890,678 | p14.1 | Allelic Imbalance | 53253 | 66.67 | 15.56 | 51.11 | 0.00 | 0.00 |  |
| chr7:44,211,453-44,307,448 | p13 | Allelic Imbalance | 95995 | 66.67 | 15.56 | 51.11 | 0.00 | 0.00 | YKT6, CAMK2B |
| chr7:45,632,558-45,704,814 | p13 | Allelic Imbalance | 72256 | 66.67 | 15.56 | 51.11 | 0.00 | 0.00 | ADCY1 |
| chr7:45,722,719-45,909,069 | p13 | Allelic Imbalance | 186350 | 66.67 | 15.56 | 51.11 | 0.00 | 0.00 | ADCY1, SEPT7P2, IGFBP1 |
| chr7:47,791,935-47,804,625 | p12.3 | Allelic Imbalance | 12690 | 66.67 | 15.56 | 51.11 | 0.00 | 0.00 | C7orf69, PKD1L1 |
| chr7:47,866,456-47,903,633 | p12.3 | Allelic Imbalance | 37177 | 66.67 | 15.56 | 51.11 | 0.00 | 0.00 | PKD1L1 |
| chr7:48,675,081-48,698,567 | p12.3 | Allelic Imbalance | 23486 | 66.67 | 15.56 | 51.11 | 0.00 | 0.00 |  |
| chr7:5,483,402-5,559,400 | p22.1 | Allelic Imbalance | 75998 | 61.90 | 11.11 | 50.79 | 0.00 | 0.00 | FBXL18, MIR589, ACTB |
| chr7:50,288,030-50,334,750 | p12.2 | Allelic Imbalance | 46720 | 61.90 | 11.11 | 50.79 | 0.00 | 0.00 | IKZF1 |
| chr7:51,660,765-51,721,578 | p12.1 | Allelic Imbalance | 60813 | 61.90 | 11.11 | 50.79 | 0.00 | 0.00 |  |
| chr7:51,916,117-51,991,681 | p12.1 | Allelic Imbalance | 75564 | 61.90 | 11.11 | 50.79 | 0.00 | 0.00 |  |
| chr7:52,516,283-52,608,872 | p12.1 | Allelic Imbalance | 92589 | 61.90 | 11.11 | 50.79 | 0.00 | 0.00 |  |
| chr7:56,940,600-57,260,703 | p11.2 | Allelic Imbalance | 320103 | 61.90 | 11.11 | 50.79 | 0.00 | 0.00 | MIR4283-1, MIR4283-2, ZNF479, GUSBP10 |
| chr7:63,777,126-63,806,423 | q11.21 | Allelic Imbalance | 29297 | 61.90 | 11.11 | 50.79 | 0.00 | 0.00 | ZNF107 |
| chr7:1,170,818-1,259,080 | p22.3 | Allelic Imbalance | 88262 | 69.05 | 17.78 | 51.27 | 0.00 | 0.00 | UNCX |
| chr7:22,167,895-22,206,531 | p15.3 | Allelic Imbalance | 38636 | 69.05 | 17.78 | 51.27 | 0.00 | 0.00 | RAPGEF5 |
| chr7:24,940,170-24,944,241 | p15.2 | Allelic Imbalance | 4071 | 69.05 | 17.78 | 51.27 | 0.00 | 0.00 | OSBPL3 |
| chr7:26,121,322-26,195,558 | p15.2 | Allelic Imbalance | 74236 | 69.05 | 17.78 | 51.27 | 0.00 | 0.00 | NFE2L3 |
| chr7:26,609,989-26,627,252 | p15.2 | Allelic Imbalance | 17263 | 69.05 | 17.78 | 51.27 | 0.00 | 0.00 |  |
| chr7:28,683,026-28,693,233 | p15.1 | Allelic Imbalance | 10207 | 69.05 | 17.78 | 51.27 | 0.00 | 0.00 | CREB5 |
| chr7:31,055,371-31,104,374 | p15.1 | Allelic Imbalance | 49003 | 69.05 | 17.78 | 51.27 | 0.00 | 0.00 | ADCYAP1R1 |
| chr7:859,990-1,091,341 | p22.3 | Allelic Imbalance | 231351 | 69.05 | 17.78 | 51.27 | 0.00 | 0.00 | SUN1, GET4, ADAP1, COX19, CYP2W1, MIR339, GPR146, C7orf50 |
| chr7:24,000,328-24,127,939 | p15.3 | Allelic Imbalance | 127611 | 71.43 | 20.00 | 51.43 | 0.00 | 0.00 |  |
| chr7:24,297,003-24,301,323 | p15.3 | Allelic Imbalance | 4320 | 71.43 | 20.00 | 51.43 | 0.00 | 0.00 | NPY |
| chr7:24,305,877-24,397,177 | p15.3 | Allelic Imbalance | 91300 | 71.43 | 20.00 | 51.43 | 0.00 | 0.00 |  |
| chr7:24,441,276-24,469,803 | p15.3 | Allelic Imbalance | 28527 | 71.43 | 20.00 | 51.43 | 0.00 | 0.00 |  |
| chr7:24,567,103-24,677,711 | p15.3 | Allelic Imbalance | 110608 | 71.43 | 20.00 | 51.43 | 0.00 | 0.00 | MPP6 |
| chr7:66,503,669-66,567,955 | q11.22 | Allelic Imbalance | 64286 | 71.43 | 20.00 | 51.43 | 0.00 | 0.00 |  |
| chr7:66,837,690-67,399,309 | q11.22 | Allelic Imbalance | 561619 | 71.43 | 20.00 | 51.43 | 0.00 | 0.00 |  |
| chr7:18,470,984-18,551,521 | p21.1 | Allelic Imbalance | 80537 | 73.81 | 22.22 | 51.59 | 0.00 | 0.00 | HDAC9 |
| chr7:18,559,295-18,585,006 | p21.1 | Allelic Imbalance | 25711 | 73.81 | 22.22 | 51.59 | 0.00 | 0.00 | HDAC9 |
| chr7:24,677,711-24,724,081 | p15.3 | Allelic Imbalance | 46370 | 73.81 | 22.22 | 51.59 | 0.00 | 0.00 | MPP6, DFNA5 |
| chr7:24,736,860-24,744,422 | p15.3 | Allelic Imbalance | 7562 | 73.81 | 22.22 | 51.59 | 0.00 | 0.00 | DFNA5 |
| chr7:31,559,974-31,742,288 | p15.1 | Allelic Imbalance | 182314 | 73.81 | 22.22 | 51.59 | 0.00 | 0.00 | CCDC129, PPP1R17 |
| chr7:5,613,717-5,696,765 | p22.1 | Allelic Imbalance | 83048 | 57.14 | 8.89 | 48.25 | 0.00 | 0.00 | RNF216-IT1, RNF216 |
| chr7:50,716,759-50,778,322 | p12.2 | Allelic Imbalance | 61563 | 57.14 | 8.89 | 48.25 | 0.00 | 0.00 | GRB10 |
| chr7:54,446,322-54,519,554 | p11.2 | Allelic Imbalance | 73232 | 57.14 | 8.89 | 48.25 | 0.00 | 0.00 |  |
| chr7:56,548,662-56,647,884 | p11.2 | Allelic Imbalance | 99222 | 57.14 | 8.89 | 48.25 | 0.00 | 0.00 |  |
| chr7:57,369,389-57,459,937 | p11.2 - p11.1 | Allelic Imbalance | 90548 | 57.14 | 8.89 | 48.25 | 0.00 | 0.00 |  |
| chr7:62,238,498-62,282,415 | q11.21 | Allelic Imbalance | 43917 | 57.14 | 8.89 | 48.25 | 0.00 | 0.00 |  |
| chr7:62,303,261-62,312,983 | q11.21 | Allelic Imbalance | 9722 | 57.14 | 8.89 | 48.25 | 0.00 | 0.00 |  |
| chr7:62,331,635-62,475,979 | q11.21 | Allelic Imbalance | 144344 | 57.14 | 8.89 | 48.25 | 0.00 | 0.00 | ZNF733P, LOC100287704, LOC100287834 |
| chr7:64,520,749-64,549,625 | q11.21 | Allelic Imbalance | 28876 | 57.14 | 8.89 | 48.25 | 0.00 | 0.00 |  |
| chr7:15,673,961-15,685,744 | p21.1 | Allelic Imbalance | 11783 | 61.90 | 13.33 | 48.57 | 0.00 | 0.00 | MEOX2 |
| chr7:16,316,271-16,352,183 | p21.1 | Allelic Imbalance | 35912 | 61.90 | 13.33 | 48.57 | 0.00 | 0.00 | ISPD |
| chr7:19,764,808-19,971,519 | p15.3 | Allelic Imbalance | 206711 | 61.90 | 13.33 | 48.57 | 0.00 | 0.00 | TMEM196 |
| chr7:2,336,756-2,484,901 | p22.2 | Allelic Imbalance | 148145 | 61.90 | 13.33 | 48.57 | 0.00 | 0.00 | EIF3B, CHST12 |
| chr7:2,938,309-2,980,171 | p22.2 | Allelic Imbalance | 41862 | 61.90 | 13.33 | 48.57 | 0.00 | 0.00 | CARD11 |
| chr7:21,132,525-21,170,946 | p15.3 | Allelic Imbalance | 38421 | 61.90 | 13.33 | 48.57 | 0.00 | 0.00 |  |
| chr7:25,258,723-25,278,822 | p15.2 | Allelic Imbalance | 20099 | 61.90 | 13.33 | 48.57 | 0.00 | 0.00 |  |
| chr7:27,654,219-27,879,985 | p15.2 | Allelic Imbalance | 225766 | 61.90 | 13.33 | 48.57 | 0.00 | 0.00 | HIBADH, TAX1BP1, JAZF1 |
| chr7:27,903,468-27,919,047 | p15.2 | Allelic Imbalance | 15579 | 61.90 | 13.33 | 48.57 | 0.00 | 0.00 | JAZF1 |
| chr7:29,362,209-29,369,735 | p15.1 | Allelic Imbalance | 7526 | 61.90 | 13.33 | 48.57 | 0.00 | 0.00 | CHN2 |
| chr7:29,378,303-29,424,668 | p15.1 | Allelic Imbalance | 46365 | 61.90 | 13.33 | 48.57 | 0.00 | 0.00 | CHN2 |
| chr7:3,011,400-3,092,402 | p22.2 | Allelic Imbalance | 81002 | 61.90 | 13.33 | 48.57 | 0.00 | 0.00 | CARD11 |
| chr7:3,187,084-3,323,991 | p22.2 | Allelic Imbalance | 136907 | 61.90 | 13.33 | 48.57 | 0.00 | 0.00 | SDK1 |
| chr7:3,534,872-3,644,011 | p22.2 | Allelic Imbalance | 109139 | 61.90 | 13.33 | 48.57 | 0.00 | 0.00 | SDK1 |
| chr7:32,392,044-32,425,818 | p14.3 | Allelic Imbalance | 33774 | 61.90 | 13.33 | 48.57 | 0.00 | 0.00 |  |
| chr7:32,466,042-32,864,610 | p14.3 | Allelic Imbalance | 398568 | 61.90 | 13.33 | 48.57 | 0.00 | 0.00 | LSM5, AVL9, DPY19L1P1, ZNRF2P1, MIR550A2, MIR550B2, LOC401321 |
| chr7:32,908,569-32,929,404 | p14.3 | Allelic Imbalance | 20835 | 61.90 | 13.33 | 48.57 | 0.00 | 0.00 | RP9P |
| chr7:33,272,592-33,308,441 | p14.3 | Allelic Imbalance | 35849 | 61.90 | 13.33 | 48.57 | 0.00 | 0.00 | BBS9 |
| chr7:36,753,789-36,769,075 | p14.2 | Allelic Imbalance | 15286 | 61.90 | 13.33 | 48.57 | 0.00 | 0.00 |  |
| chr7:37,341,670-37,352,230 | p14.2 | Allelic Imbalance | 10560 | 61.90 | 13.33 | 48.57 | 0.00 | 0.00 | ELMO1 |
| chr7:37,495,760-37,521,701 | p14.2 - p14.1 | Allelic Imbalance | 25941 | 61.90 | 13.33 | 48.57 | 0.00 | 0.00 |  |
| chr7:38,327,615-38,382,985 | p14.1 | Allelic Imbalance | 55370 | 61.90 | 13.33 | 48.57 | 0.00 | 0.00 | LOC100506776 |
| chr7:38,459,202-38,504,642 | p14.1 | Allelic Imbalance | 45440 | 61.90 | 13.33 | 48.57 | 0.00 | 0.00 | AMPH |
| chr7:39,152,119-39,294,627 | p14.1 | Allelic Imbalance | 142508 | 61.90 | 13.33 | 48.57 | 0.00 | 0.00 | POU6F2 |
| chr7:40,697,708-40,707,728 | p14.1 | Allelic Imbalance | 10020 | 61.90 | 13.33 | 48.57 | 0.00 | 0.00 | C7orf10 |
| chr7:40,837,206-40,868,268 | p14.1 | Allelic Imbalance | 31062 | 61.90 | 13.33 | 48.57 | 0.00 | 0.00 | C7orf10 |
| chr7:41,706,426-41,730,121 | p14.1 | Allelic Imbalance | 23695 | 61.90 | 13.33 | 48.57 | 0.00 | 0.00 | INHBA, INHBA-AS1 |
| chr7:43,930,608-43,962,406 | p13 | Allelic Imbalance | 31798 | 61.90 | 13.33 | 48.57 | 0.00 | 0.00 | URGCP, UBE2D4, POLR2J4 |
| chr7:44,131,299-44,172,084 | p13 | Allelic Imbalance | 40785 | 61.90 | 13.33 | 48.57 | 0.00 | 0.00 | MYL7, GCK |
| chr7:47,961,637-47,974,644 | p12.3 | Allelic Imbalance | 13007 | 61.90 | 13.33 | 48.57 | 0.00 | 0.00 | HUS1 |
| chr7:48,014,877-48,052,464 | p12.3 | Allelic Imbalance | 37587 | 61.90 | 13.33 | 48.57 | 0.00 | 0.00 | SUN3, C7orf57 |
| chr7:50,858,010-50,877,606 | p12.2 | Allelic Imbalance | 19596 | 61.90 | 13.33 | 48.57 | 0.00 | 0.00 |  |
| chr7:50,898,976-51,064,589 | p12.2 - p12.1 | Allelic Imbalance | 165613 | 61.90 | 13.33 | 48.57 | 0.00 | 0.00 | COBL |
| chr7:51,355,238-51,611,528 | p12.1 | Allelic Imbalance | 256290 | 61.90 | 13.33 | 48.57 | 0.00 | 0.00 |  |
| chr7:9,669,483-9,731,723 | p21.3 | Allelic Imbalance | 62240 | 61.90 | 13.33 | 48.57 | 0.00 | 0.00 |  |
| chr7:54,767,240-54,775,777 | p11.2 | Allelic Imbalance | 8537 | 52.38 | 6.67 | 45.71 | 0.00 | 0.00 |  |
| chr7:55,649,706-55,657,954 | p11.2 | Allelic Imbalance | 8248 | 52.38 | 6.67 | 45.71 | 0.00 | 0.00 |  |
| chr7:55,663,391-55,693,954 | p11.2 | Allelic Imbalance | 30563 | 52.38 | 6.67 | 45.71 | 0.00 | 0.00 |  |
| chr7:56,116,382-56,146,881 | p11.2 | Allelic Imbalance | 30499 | 52.38 | 6.67 | 45.71 | 0.00 | 0.00 | PHKG1, CHCHD2 |
| chr7:56,215,192-56,263,476 | p11.2 | Allelic Imbalance | 48284 | 52.38 | 6.67 | 45.71 | 0.00 | 0.00 |  |
| chr7:6,002,537-6,011,314 | p22.1 | Allelic Imbalance | 8777 | 52.38 | 6.67 | 45.71 | 0.00 | 0.00 | PMS2 |
| chr7:6,012,880-6,019,436 | p22.1 | Allelic Imbalance | 6556 | 52.38 | 6.67 | 45.71 | 0.00 | 0.00 | PMS2, AIMP2 |
| chr7:64,698,576-64,727,417 | q11.21 | Allelic Imbalance | 28841 | 52.38 | 6.67 | 45.71 | 0.00 | 0.00 |  |
| chr7:11,625,427-11,758,953 | p21.3 | Allelic Imbalance | 133526 | 64.29 | 15.56 | 48.73 | 0.00 | 0.00 | THSD7A |
| chr7:13,650,153-13,842,133 | p21.2 | Allelic Imbalance | 191980 | 64.29 | 15.56 | 48.73 | 0.00 | 0.00 |  |
| chr7:13,868,052-13,873,703 | p21.2 | Allelic Imbalance | 5651 | 64.29 | 15.56 | 48.73 | 0.00 | 0.00 |  |
| chr7:13,911,400-13,912,470 | p21.2 | Allelic Imbalance | 1070 | 64.29 | 15.56 | 48.73 | 0.00 | 0.00 | ETV1 |
| chr7:13,981,720-13,995,003 | p21.2 | Allelic Imbalance | 13283 | 64.29 | 15.56 | 48.73 | 0.00 | 0.00 | ETV1 |
| chr7:14,834,547-14,957,764 | p21.2 | Allelic Imbalance | 123217 | 64.29 | 15.56 | 48.73 | 0.00 | 0.00 | DGKB |
| chr7:15,041,363-15,087,638 | p21.2 | Allelic Imbalance | 46275 | 64.29 | 15.56 | 48.73 | 0.00 | 0.00 |  |
| chr7:15,343,262-15,375,375 | p21.1 | Allelic Imbalance | 32113 | 64.29 | 15.56 | 48.73 | 0.00 | 0.00 | AGMO |
| chr7:15,639,822-15,650,138 | p21.1 | Allelic Imbalance | 10316 | 64.29 | 15.56 | 48.73 | 0.00 | 0.00 | MEOX2 |
| chr7:15,725,078-15,748,630 | p21.1 | Allelic Imbalance | 23552 | 59.52 | 11.11 | 48.41 | 0.00 | 0.00 |  |
| chr7:16,537,508-16,620,664 | p21.1 | Allelic Imbalance | 83156 | 64.29 | 15.56 | 48.73 | 0.00 | 0.00 | LRRC72, ANKMY2 |
| chr7:16,784,904-16,788,509 | p21.1 | Allelic Imbalance | 3605 | 64.29 | 15.56 | 48.73 | 0.00 | 0.00 | TSPAN13 |
| chr7:19,312,666-19,319,384 | p21.1 | Allelic Imbalance | 6718 | 64.29 | 15.56 | 48.73 | 0.00 | 0.00 |  |
| chr7:19,744,624-19,762,917 | p15.3 | Allelic Imbalance | 18293 | 64.29 | 15.56 | 48.73 | 0.00 | 0.00 | TMEM196 |
| chr7:20,013,365-20,027,858 | p15.3 | Allelic Imbalance | 14493 | 64.29 | 15.56 | 48.73 | 0.00 | 0.00 |  |
| chr7:21,109,118-21,124,367 | p15.3 | Allelic Imbalance | 15249 | 64.29 | 15.56 | 48.73 | 0.00 | 0.00 |  |
| chr7:21,501,489-21,555,192 | p15.3 | Allelic Imbalance | 53703 | 59.52 | 11.11 | 48.41 | 0.00 | 0.00 | SP4, DNAH11 |
| chr7:22,293,192-22,304,758 | p15.3 | Allelic Imbalance | 11566 | 64.29 | 15.56 | 48.73 | 0.00 | 0.00 | RAPGEF5 |
| chr7:22,321,997-22,416,092 | p15.3 | Allelic Imbalance | 94095 | 64.29 | 15.56 | 48.73 | 0.00 | 0.00 | RAPGEF5 |
| chr7:22,469,420-22,608,651 | p15.3 | Allelic Imbalance | 139231 | 64.29 | 15.56 | 48.73 | 0.00 | 0.00 | STEAP1B, LOC100506178 |
| chr7:25,436,977-25,497,347 | p15.2 | Allelic Imbalance | 60370 | 64.29 | 15.56 | 48.73 | 0.00 | 0.00 |  |
| chr7:29,474,745-29,483,640 | p15.1 | Allelic Imbalance | 8895 | 64.29 | 15.56 | 48.73 | 0.00 | 0.00 | CHN2 |
| chr7:3,432,262-3,534,872 | p22.2 | Allelic Imbalance | 102610 | 59.52 | 11.11 | 48.41 | 0.00 | 0.00 | SDK1 |
| chr7:33,517,890-33,537,301 | p14.3 | Allelic Imbalance | 19411 | 64.29 | 15.56 | 48.73 | 0.00 | 0.00 | BBS9 |
| chr7:33,842,336-33,949,655 | p14.3 | Allelic Imbalance | 107319 | 64.29 | 15.56 | 48.73 | 0.00 | 0.00 | BMPER |
| chr7:36,602,479-36,603,935 | p14.2 | Allelic Imbalance | 1456 | 64.29 | 15.56 | 48.73 | 0.00 | 0.00 | AOAH |
| chr7:36,612,064-36,627,720 | p14.2 | Allelic Imbalance | 15656 | 64.29 | 15.56 | 48.73 | 0.00 | 0.00 | AOAH |
| chr7:36,725,100-36,735,144 | p14.2 | Allelic Imbalance | 10044 | 64.29 | 15.56 | 48.73 | 0.00 | 0.00 | AOAH |
| chr7:37,673,322-37,899,096 | p14.1 | Allelic Imbalance | 225774 | 59.52 | 11.11 | 48.41 | 0.00 | 0.00 | GPR141, NME8 |
| chr7:38,796,733-38,809,423 | p14.1 | Allelic Imbalance | 12690 | 64.29 | 15.56 | 48.73 | 0.00 | 0.00 | VPS41 |
| chr7:38,910,686-39,012,326 | p14.1 | Allelic Imbalance | 101640 | 64.29 | 15.56 | 48.73 | 0.00 | 0.00 | VPS41, POU6F2 |
| chr7:39,327,967-39,509,301 | p14.1 | Allelic Imbalance | 181334 | 64.29 | 15.56 | 48.73 | 0.00 | 0.00 | POU6F2, POU6F2-AS1 |
| chr7:4,172,579-4,239,764 | p22.2 | Allelic Imbalance | 67185 | 64.29 | 15.56 | 48.73 | 0.00 | 0.00 | SDK1 |
| chr7:40,890,703-41,040,684 | p14.1 | Allelic Imbalance | 149981 | 64.29 | 15.56 | 48.73 | 0.00 | 0.00 |  |
| chr7:41,060,898-41,351,690 | p14.1 | Allelic Imbalance | 290792 | 64.29 | 15.56 | 48.73 | 0.00 | 0.00 |  |
| chr7:41,837,475-41,939,083 | p14.1 | Allelic Imbalance | 101608 | 64.29 | 15.56 | 48.73 | 0.00 | 0.00 |  |
| chr7:42,005,458-42,051,755 | p14.1 | Allelic Imbalance | 46297 | 64.29 | 15.56 | 48.73 | 0.00 | 0.00 | GLI3 |
| chr7:42,890,678-43,193,067 | p14.1 | Allelic Imbalance | 302389 | 64.29 | 15.56 | 48.73 | 0.00 | 0.00 | C7orf25, PSMA2, MRPL32, MIR3943, HECW1 |
| chr7:43,234,748-43,571,582 | p14.1 - p13 | Allelic Imbalance | 336834 | 64.29 | 15.56 | 48.73 | 0.00 | 0.00 | HECW1, LOC100506895 |
| chr7:44,189,786-44,211,453 | p13 | Allelic Imbalance | 21667 | 64.29 | 15.56 | 48.73 | 0.00 | 0.00 | GCK, YKT6 |
| chr7:45,909,069-45,937,609 | p13 | Allelic Imbalance | 28540 | 64.29 | 15.56 | 48.73 | 0.00 | 0.00 | IGFBP3 |
| chr7:45,995,372-46,005,076 | p13 | Allelic Imbalance | 9704 | 64.29 | 15.56 | 48.73 | 0.00 | 0.00 |  |
| chr7:48,337,086-48,453,363 | p12.3 | Allelic Imbalance | 116277 | 64.29 | 15.56 | 48.73 | 0.00 | 0.00 | ABCA13 |
| chr7:48,485,285-48,675,081 | p12.3 | Allelic Imbalance | 189796 | 64.29 | 15.56 | 48.73 | 0.00 | 0.00 | ABCA13 |
| chr7:50,421,269-50,430,030 | p12.2 | Allelic Imbalance | 8761 | 59.52 | 11.11 | 48.41 | 0.00 | 0.00 | IKZF1 |
| chr7:50,800,982-50,858,010 | p12.2 | Allelic Imbalance | 57028 | 59.52 | 11.11 | 48.41 | 0.00 | 0.00 | GRB10 |
| chr7:51,647,235-51,660,765 | p12.1 | Allelic Imbalance | 13530 | 59.52 | 11.11 | 48.41 | 0.00 | 0.00 |  |
| chr7:51,991,681-52,140,959 | p12.1 | Allelic Imbalance | 149278 | 59.52 | 11.11 | 48.41 | 0.00 | 0.00 |  |
| chr7:52,177,267-52,197,376 | p12.1 | Allelic Imbalance | 20109 | 59.52 | 11.11 | 48.41 | 0.00 | 0.00 |  |
| chr7:52,258,119-52,418,942 | p12.1 | Allelic Imbalance | 160823 | 59.52 | 11.11 | 48.41 | 0.00 | 0.00 |  |
| chr7:52,455,191-52,516,283 | p12.1 | Allelic Imbalance | 61092 | 59.52 | 11.11 | 48.41 | 0.00 | 0.00 |  |
| chr7:56,687,422-56,940,600 | p11.2 | Allelic Imbalance | 253178 | 59.52 | 11.11 | 48.41 | 0.00 | 0.00 | LOC100130849 |
| chr7:61,068,460-61,926,360 | q11.1 - q11.21 | Allelic Imbalance | 857900 | 59.52 | 11.11 | 48.41 | 0.00 | 0.00 |  |
| chr7:63,806,423-63,916,085 | q11.21 | Allelic Imbalance | 109662 | 59.52 | 11.11 | 48.41 | 0.00 | 0.00 | ZNF107, ZNF138 |
| chr7:1,259,080-1,659,595 | p22.3 | Allelic Imbalance | 400515 | 66.67 | 17.78 | 48.89 | 0.00 | 0.00 | MICALL2, INTS1, MAFK, TMEM184A, PSMG3, PSMG3-AS1, TFAMP1 |
| chr7:13,267,440-13,327,924 | p21.3 - p21.2 | Allelic Imbalance | 60484 | 66.67 | 17.78 | 48.89 | 0.00 | 0.00 |  |
| chr7:16,718,357-16,752,851 | p21.1 | Allelic Imbalance | 34494 | 66.67 | 17.78 | 48.89 | 0.00 | 0.00 |  |
| chr7:17,019,608-17,080,342 | p21.1 | Allelic Imbalance | 60734 | 66.67 | 17.78 | 48.89 | 0.00 | 0.00 |  |
| chr7:17,929,857-17,979,651 | p21.1 | Allelic Imbalance | 49794 | 66.67 | 17.78 | 48.89 | 0.00 | 0.00 | SNX13 |
| chr7:22,206,531-22,273,812 | p15.3 | Allelic Imbalance | 67281 | 66.67 | 17.78 | 48.89 | 0.00 | 0.00 | RAPGEF5 |
| chr7:24,864,588-24,940,170 | p15.3 - p15.2 | Allelic Imbalance | 75582 | 66.67 | 17.78 | 48.89 | 0.00 | 0.00 | OSBPL3 |
| chr7:26,627,252-26,657,099 | p15.2 | Allelic Imbalance | 29847 | 66.67 | 17.78 | 48.89 | 0.00 | 0.00 | C7orf71 |
| chr7:28,019,062-28,100,428 | p15.1 | Allelic Imbalance | 81366 | 66.67 | 17.78 | 48.89 | 0.00 | 0.00 | JAZF1 |
| chr7:28,398,172-28,416,523 | p15.1 | Allelic Imbalance | 18351 | 66.67 | 17.78 | 48.89 | 0.00 | 0.00 | CREB5 |
| chr7:28,671,364-28,683,026 | p15.1 | Allelic Imbalance | 11662 | 66.67 | 17.78 | 48.89 | 0.00 | 0.00 | CREB5 |
| chr7:30,983,733-31,055,371 | p15.1 | Allelic Imbalance | 71638 | 66.67 | 17.78 | 48.89 | 0.00 | 0.00 | GHRHR |
| chr7:45,212,875-45,261,547 | p13 | Allelic Imbalance | 48672 | 66.67 | 17.78 | 48.89 | 0.00 | 0.00 |  |
| chr7:45,572,908-45,632,558 | p13 | Allelic Imbalance | 59650 | 66.67 | 17.78 | 48.89 | 0.00 | 0.00 | ADCY1 |
| chr7:47,804,625-47,866,456 | p12.3 | Allelic Imbalance | 61831 | 66.67 | 17.78 | 48.89 | 0.00 | 0.00 | C7orf69, PKD1L1 |
| chr7:23,928,891-24,000,328 | p15.3 | Allelic Imbalance | 71437 | 69.05 | 20.00 | 49.05 | 0.00 | 0.00 |  |
| chr7:24,127,939-24,246,633 | p15.3 | Allelic Imbalance | 118694 | 69.05 | 20.00 | 49.05 | 0.00 | 0.00 |  |
| chr7:24,301,323-24,305,877 | p15.3 | Allelic Imbalance | 4554 | 69.05 | 20.00 | 49.05 | 0.00 | 0.00 |  |
| chr7:24,469,803-24,567,103 | p15.3 | Allelic Imbalance | 97300 | 69.05 | 20.00 | 49.05 | 0.00 | 0.00 |  |
| chr7:24,744,422-24,763,523 | p15.3 | Allelic Imbalance | 19101 | 69.05 | 20.00 | 49.05 | 0.00 | 0.00 | DFNA5 |
| chr7:24,859,387-24,864,588 | p15.3 | Allelic Imbalance | 5201 | 69.05 | 20.00 | 49.05 | 0.00 | 0.00 | OSBPL3 |
| chr7:66,432,187-66,503,669 | q11.22 | Allelic Imbalance | 71482 | 69.05 | 20.00 | 49.05 | 0.00 | 0.00 |  |
| chr7:67,399,309-67,509,266 | q11.22 | Allelic Imbalance | 109957 | 69.05 | 20.00 | 49.05 | 0.00 | 0.00 |  |
| chr7:18,185,031-18,218,425 | p21.1 | Allelic Imbalance | 33394 | 71.43 | 22.22 | 49.21 | 0.00 | 0.00 | HDAC9 |
| chr7:18,275,054-18,470,984 | p21.1 | Allelic Imbalance | 195930 | 71.43 | 22.22 | 49.21 | 0.00 | 0.00 | HDAC9 |
| chr7:18,551,521-18,559,295 | p21.1 | Allelic Imbalance | 7774 | 71.43 | 22.22 | 49.21 | 0.00 | 0.00 | HDAC9 |
| chr7:24,278,536-24,297,003 | p15.3 | Allelic Imbalance | 18467 | 71.43 | 22.22 | 49.21 | 0.00 | 0.00 | NPY |
| chr7:24,724,081-24,736,860 | p15.3 | Allelic Imbalance | 12779 | 71.43 | 22.22 | 49.21 | 0.00 | 0.00 | DFNA5 |
| chr7:66,567,955-66,837,690 | q11.22 | Allelic Imbalance | 269735 | 71.43 | 22.22 | 49.21 | 0.00 | 0.00 |  |
| chr7:67,662,105-67,903,623 | q11.22 | Allelic Imbalance | 241518 | 71.43 | 22.22 | 49.21 | 0.00 | 0.00 |  |
| chr7:150,119,136-150,123,222 | q36.1 | Allelic Imbalance | 4086 | 73.81 | 24.44 | 49.37 | 0.00 | 0.00 | TMEM176B |
| chr7:31,742,288-31,768,622 | p15.1 | Allelic Imbalance | 26334 | 73.81 | 24.44 | 49.37 | 0.00 | 0.00 | PDE1C |
| chr7:5,696,765-5,702,238 | p22.1 | Allelic Imbalance | 5473 | 54.76 | 8.89 | 45.87 | 0.00 | 0.00 | RNF216 |
| chr7:50,701,192-50,716,759 | p12.2 | Allelic Imbalance | 15567 | 54.76 | 8.89 | 45.87 | 0.00 | 0.00 | GRB10 |
| chr7:54,519,554-54,557,930 | p11.2 | Allelic Imbalance | 38376 | 54.76 | 8.89 | 45.87 | 0.00 | 0.00 |  |
| chr7:62,201,402-62,238,498 | q11.21 | Allelic Imbalance | 37096 | 54.76 | 8.89 | 45.87 | 0.00 | 0.00 |  |
| chr7:62,282,415-62,303,261 | q11.21 | Allelic Imbalance | 20846 | 54.76 | 8.89 | 45.87 | 0.00 | 0.00 |  |
| chr7:65,896,840-66,058,299 | q11.21 | Allelic Imbalance | 161459 | 54.76 | 8.89 | 45.87 | 0.00 | 0.00 | RABGEF1, GTF2IRD1P1, TMEM248 |
| chr7:54,663,589-54,767,240 | p11.2 | Allelic Imbalance | 103651 | 50.00 | 6.67 | 43.33 | 0.00 | 0.00 |  |
| chr7:54,775,777-54,834,444 | p11.2 | Allelic Imbalance | 58667 | 50.00 | 6.67 | 43.33 | 0.00 | 0.00 | SEC61G |
| chr7:55,633,578-55,649,706 | p11.2 | Allelic Imbalance | 16128 | 50.00 | 6.67 | 43.33 | 0.00 | 0.00 |  |
| chr7:55,657,954-55,663,391 | p11.2 | Allelic Imbalance | 5437 | 50.00 | 6.67 | 43.33 | 0.00 | 0.00 |  |
| chr7:55,693,954-55,796,838 | p11.2 | Allelic Imbalance | 102884 | 50.00 | 6.67 | 43.33 | 0.00 | 0.00 | FKBP9L |
| chr7:56,109,418-56,116,382 | p11.2 | Allelic Imbalance | 6964 | 50.00 | 6.67 | 43.33 | 0.00 | 0.00 | SUMF2, PHKG1 |
| chr7:64,727,417-65,007,623 | q11.21 | Allelic Imbalance | 280206 | 50.00 | 6.67 | 43.33 | 0.00 | 0.00 | INTS4L2, LOC441242, SNORA22, CCT6P1, VKORC1L1 |
| chr7:11,207,928-11,211,524 | p21.3 | Allelic Imbalance | 3596 | 61.90 | 15.56 | 46.35 | 0.00 | 0.00 |  |
| chr7:13,873,703-13,911,400 | p21.2 | Allelic Imbalance | 37697 | 61.90 | 15.56 | 46.35 | 0.00 | 0.00 | ETV1 |
| chr7:13,912,470-13,981,720 | p21.2 | Allelic Imbalance | 69250 | 61.90 | 15.56 | 46.35 | 0.00 | 0.00 | ETV1 |
| chr7:14,671,355-14,834,547 | p21.2 | Allelic Imbalance | 163192 | 61.90 | 15.56 | 46.35 | 0.00 | 0.00 | DGKB |
| chr7:15,375,375-15,461,629 | p21.1 | Allelic Imbalance | 86254 | 61.90 | 15.56 | 46.35 | 0.00 | 0.00 | AGMO |
| chr7:15,636,775-15,639,822 | p21.1 | Allelic Imbalance | 3047 | 61.90 | 15.56 | 46.35 | 0.00 | 0.00 | MEOX2 |
| chr7:15,748,630-15,885,002 | p21.1 | Allelic Imbalance | 136372 | 57.14 | 11.11 | 46.03 | 0.00 | 0.00 |  |
| chr7:15,932,227-16,089,440 | p21.1 | Allelic Imbalance | 157213 | 57.14 | 11.11 | 46.03 | 0.00 | 0.00 |  |
| chr7:16,352,183-16,537,508 | p21.1 | Allelic Imbalance | 185325 | 61.90 | 15.56 | 46.35 | 0.00 | 0.00 | ISPD, SOSTDC1, LRRC72 |
| chr7:17,708,337-17,711,698 | p21.1 | Allelic Imbalance | 3361 | 61.90 | 15.56 | 46.35 | 0.00 | 0.00 |  |
| chr7:19,319,384-19,402,543 | p21.1 | Allelic Imbalance | 83159 | 61.90 | 15.56 | 46.35 | 0.00 | 0.00 |  |
| chr7:19,731,765-19,744,624 | p15.3 | Allelic Imbalance | 12859 | 61.90 | 15.56 | 46.35 | 0.00 | 0.00 | TMEM196 |
| chr7:2,980,171-3,011,400 | p22.2 | Allelic Imbalance | 31229 | 61.90 | 15.56 | 46.35 | 0.00 | 0.00 | CARD11 |
| chr7:21,124,367-21,132,525 | p15.3 | Allelic Imbalance | 8158 | 61.90 | 15.56 | 46.35 | 0.00 | 0.00 |  |
| chr7:22,608,651-22,666,077 | p15.3 | Allelic Imbalance | 57426 | 61.90 | 15.56 | 46.35 | 0.00 | 0.00 |  |
| chr7:22,865,137-22,877,287 | p15.3 | Allelic Imbalance | 12150 | 61.90 | 15.56 | 46.35 | 0.00 | 0.00 |  |
| chr7:25,307,248-25,403,520 | p15.2 | Allelic Imbalance | 96272 | 61.90 | 15.56 | 46.35 | 0.00 | 0.00 |  |
| chr7:27,919,047-27,919,830 | p15.2 | Allelic Imbalance | 783 | 61.90 | 15.56 | 46.35 | 0.00 | 0.00 | JAZF1 |
| chr7:29,483,640-29,485,324 | p15.1 | Allelic Imbalance | 1684 | 61.90 | 15.56 | 46.35 | 0.00 | 0.00 | CHN2 |
| chr7:29,825,743-29,908,550 | p15.1 | Allelic Imbalance | 82807 | 61.90 | 15.56 | 46.35 | 0.00 | 0.00 | WIPF3 |
| chr7:3,092,402-3,187,084 | p22.2 | Allelic Imbalance | 94682 | 61.90 | 15.56 | 46.35 | 0.00 | 0.00 |  |
| chr7:30,737,908-30,817,488 | p15.1 | Allelic Imbalance | 79580 | 61.90 | 15.56 | 46.35 | 0.00 | 0.00 | INMT, INMT-FAM188B, FAM188B |
| chr7:30,954,073-30,967,845 | p15.1 | Allelic Imbalance | 13772 | 61.90 | 15.56 | 46.35 | 0.00 | 0.00 |  |
| chr7:33,437,332-33,517,890 | p14.3 | Allelic Imbalance | 80558 | 61.90 | 15.56 | 46.35 | 0.00 | 0.00 | BBS9 |
| chr7:36,603,935-36,612,064 | p14.2 | Allelic Imbalance | 8129 | 61.90 | 15.56 | 46.35 | 0.00 | 0.00 | AOAH-IT1, AOAH |
| chr7:37,231,564-37,266,962 | p14.2 | Allelic Imbalance | 35398 | 57.14 | 11.11 | 46.03 | 0.00 | 0.00 | ELMO1 |
| chr7:38,504,642-38,573,586 | p14.1 | Allelic Imbalance | 68944 | 61.90 | 15.56 | 46.35 | 0.00 | 0.00 | AMPH |
| chr7:38,593,291-38,682,386 | p14.1 | Allelic Imbalance | 89095 | 61.90 | 15.56 | 46.35 | 0.00 | 0.00 | AMPH |
| chr7:38,747,997-38,796,733 | p14.1 | Allelic Imbalance | 48736 | 61.90 | 15.56 | 46.35 | 0.00 | 0.00 | VPS41 |
| chr7:39,012,326-39,152,119 | p14.1 | Allelic Imbalance | 139793 | 61.90 | 15.56 | 46.35 | 0.00 | 0.00 | POU6F2 |
| chr7:39,811,304-39,851,800 | p14.1 | Allelic Imbalance | 40496 | 61.90 | 15.56 | 46.35 | 0.00 | 0.00 |  |
| chr7:40,868,268-40,890,703 | p14.1 | Allelic Imbalance | 22435 | 61.90 | 15.56 | 46.35 | 0.00 | 0.00 |  |
| chr7:41,040,684-41,060,898 | p14.1 | Allelic Imbalance | 20214 | 61.90 | 15.56 | 46.35 | 0.00 | 0.00 |  |
| chr7:42,004,998-42,005,458 | p14.1 | Allelic Imbalance | 460 | 61.90 | 15.56 | 46.35 | 0.00 | 0.00 | GLI3 |
| chr7:43,193,067-43,234,748 | p14.1 | Allelic Imbalance | 41681 | 61.90 | 15.56 | 46.35 | 0.00 | 0.00 | HECW1 |
| chr7:43,603,608-43,827,456 | p13 | Allelic Imbalance | 223848 | 61.90 | 15.56 | 46.35 | 0.00 | 0.00 | STK17A, COA1, BLVRA |
| chr7:43,906,419-43,930,608 | p13 | Allelic Imbalance | 24189 | 61.90 | 15.56 | 46.35 | 0.00 | 0.00 | URGCP-MRPS24, URGCP |
| chr7:44,172,084-44,184,021 | p13 | Allelic Imbalance | 11937 | 61.90 | 15.56 | 46.35 | 0.00 | 0.00 | GCK |
| chr7:44,189,161-44,189,786 | p13 | Allelic Imbalance | 625 | 61.90 | 15.56 | 46.35 | 0.00 | 0.00 | GCK |
| chr7:44,307,448-44,318,700 | p13 | Allelic Imbalance | 11252 | 61.90 | 15.56 | 46.35 | 0.00 | 0.00 | CAMK2B |
| chr7:44,948,337-45,006,088 | p13 | Allelic Imbalance | 57751 | 61.90 | 15.56 | 46.35 | 0.00 | 0.00 | MYO1G, SNHG15, SNORA9, CCM2 |
| chr7:45,937,609-45,995,372 | p13 | Allelic Imbalance | 57763 | 61.90 | 15.56 | 46.35 | 0.00 | 0.00 |  |
| chr7:48,052,464-48,070,152 | p12.3 | Allelic Imbalance | 17688 | 61.90 | 15.56 | 46.35 | 0.00 | 0.00 | C7orf57 |
| chr7:48,292,400-48,337,086 | p12.3 | Allelic Imbalance | 44686 | 61.90 | 15.56 | 46.35 | 0.00 | 0.00 | ABCA13 |
| chr7:48,453,363-48,485,285 | p12.3 | Allelic Imbalance | 31922 | 61.90 | 15.56 | 46.35 | 0.00 | 0.00 | ABCA13 |
| chr7:5,559,400-5,613,717 | p22.1 | Allelic Imbalance | 54317 | 57.14 | 11.11 | 46.03 | 0.00 | 0.00 | FSCN1 |
| chr7:50,430,030-50,487,892 | p12.2 | Allelic Imbalance | 57862 | 57.14 | 11.11 | 46.03 | 0.00 | 0.00 | IKZF1, FIGNL1 |
| chr7:52,140,959-52,177,267 | p12.1 | Allelic Imbalance | 36308 | 57.14 | 11.11 | 46.03 | 0.00 | 0.00 |  |
| chr7:52,418,942-52,455,191 | p12.1 | Allelic Imbalance | 36249 | 57.14 | 11.11 | 46.03 | 0.00 | 0.00 |  |
| chr7:56,647,884-56,687,422 | p11.2 | Allelic Imbalance | 39538 | 57.14 | 11.11 | 46.03 | 0.00 | 0.00 |  |
| chr7:61,926,360-62,084,576 | q11.21 | Allelic Imbalance | 158216 | 57.14 | 11.11 | 46.03 | 0.00 | 0.00 |  |
| chr7:64,454,218-64,520,749 | q11.21 | Allelic Imbalance | 66531 | 57.14 | 11.11 | 46.03 | 0.00 | 0.00 | ZNF92 |
| chr7:80,077,643-80,120,174 | q21.11 | Allelic Imbalance | 42531 | 61.90 | 15.56 | 46.35 | 0.00 | 0.00 | CD36 |
| chr7:80,127,909-80,134,799 | q21.11 | Allelic Imbalance | 6890 | 61.90 | 15.56 | 46.35 | 0.00 | 0.00 | CD36 |
| chr7:9,956,694-10,133,371 | p21.3 | Allelic Imbalance | 176677 | 57.14 | 11.11 | 46.03 | 0.00 | 0.00 |  |
| chr7:11,592,580-11,625,427 | p21.3 | Allelic Imbalance | 32847 | 64.29 | 17.78 | 46.51 | 0.00 | 0.00 | THSD7A |
| chr7:11,758,953-11,825,303 | p21.3 | Allelic Imbalance | 66350 | 64.29 | 17.78 | 46.51 | 0.00 | 0.00 | THSD7A |
| chr7:12,671,897-12,699,495 | p21.3 | Allelic Imbalance | 27598 | 64.29 | 17.78 | 46.51 | 0.00 | 0.00 | ARL4A |
| chr7:13,218,344-13,267,440 | p21.3 | Allelic Imbalance | 49096 | 64.29 | 17.78 | 46.51 | 0.00 | 0.00 |  |
| chr7:13,995,003-14,237,951 | p21.2 | Allelic Imbalance | 242948 | 64.29 | 17.78 | 46.51 | 0.00 | 0.00 | ETV1, DGKB |
| chr7:14,957,764-15,041,363 | p21.2 | Allelic Imbalance | 83599 | 64.29 | 17.78 | 46.51 | 0.00 | 0.00 |  |
| chr7:16,752,851-16,784,904 | p21.1 | Allelic Imbalance | 32053 | 64.29 | 17.78 | 46.51 | 0.00 | 0.00 | TSPAN13 |
| chr7:16,788,509-17,019,608 | p21.1 | Allelic Imbalance | 231099 | 64.29 | 17.78 | 46.51 | 0.00 | 0.00 | TSPAN13, AGR2, AGR3 |
| chr7:17,080,342-17,471,812 | p21.1 | Allelic Imbalance | 391470 | 64.29 | 17.78 | 46.51 | 0.00 | 0.00 | AHR |
| chr7:17,741,772-17,929,857 | p21.1 | Allelic Imbalance | 188085 | 64.29 | 17.78 | 46.51 | 0.00 | 0.00 | SNX13 |
| chr7:22,416,092-22,469,420 | p15.3 | Allelic Imbalance | 53328 | 64.29 | 17.78 | 46.51 | 0.00 | 0.00 | STEAP1B |
| chr7:23,343,897-23,366,369 | p15.3 | Allelic Imbalance | 22472 | 64.29 | 17.78 | 46.51 | 0.00 | 0.00 | IGF2BP3 |
| chr7:23,515,124-23,569,781 | p15.3 | Allelic Imbalance | 54657 | 64.29 | 17.78 | 46.51 | 0.00 | 0.00 | TRA2A |
| chr7:26,657,099-26,796,849 | p15.2 | Allelic Imbalance | 139750 | 64.29 | 17.78 | 46.51 | 0.00 | 0.00 | SKAP2 |
| chr7:27,961,327-28,019,062 | p15.2 - p15.1 | Allelic Imbalance | 57735 | 64.29 | 17.78 | 46.51 | 0.00 | 0.00 | JAZF1 |
| chr7:28,333,198-28,398,172 | p15.1 | Allelic Imbalance | 64974 | 64.29 | 17.78 | 46.51 | 0.00 | 0.00 | CREB5 |
| chr7:30,973,998-30,983,733 | p15.1 | Allelic Imbalance | 9735 | 64.29 | 17.78 | 46.51 | 0.00 | 0.00 | GHRHR |
| chr7:36,627,720-36,725,100 | p14.2 | Allelic Imbalance | 97380 | 64.29 | 17.78 | 46.51 | 0.00 | 0.00 | AOAH |
| chr7:39,509,301-39,545,124 | p14.1 | Allelic Imbalance | 35823 | 64.29 | 17.78 | 46.51 | 0.00 | 0.00 |  |
| chr7:41,939,083-41,963,372 | p14.1 | Allelic Imbalance | 24289 | 64.29 | 17.78 | 46.51 | 0.00 | 0.00 |  |
| chr7:45,021,796-45,118,802 | p13 | Allelic Imbalance | 97006 | 64.29 | 17.78 | 46.51 | 0.00 | 0.00 | CCM2, NACAD, SNORA5A, SNORA5C, TBRG4, SNORA5B |
| chr7:45,204,789-45,212,875 | p13 | Allelic Imbalance | 8086 | 64.29 | 17.78 | 46.51 | 0.00 | 0.00 |  |
| chr7:45,261,547-45,286,297 | p13 | Allelic Imbalance | 24750 | 64.29 | 17.78 | 46.51 | 0.00 | 0.00 |  |
| chr7:73,156,969-73,159,597 | q11.23 | Allelic Imbalance | 2628 | 64.29 | 17.78 | 46.51 | 0.00 | 0.00 | LIMK1 |
| chr7:0-859,990 | p22.3 | Allelic Imbalance | 859990 | 66.67 | 20.00 | 46.67 | 0.00 | 0.00 | FAM20C, PDGFA, FLJ44511, PRKAR1B, HEATR2, SUN1 |
| chr7:10,185,686-10,302,444 | p21.3 | Allelic Imbalance | 116758 | 59.52 | 13.33 | 46.19 | 0.00 | 0.00 |  |
| chr7:16,246,528-16,316,271 | p21.1 | Allelic Imbalance | 69743 | 59.52 | 13.33 | 46.19 | 0.00 | 0.00 | LOC100506025, ISPD |
| chr7:17,979,651-17,993,188 | p21.1 | Allelic Imbalance | 13537 | 66.67 | 20.00 | 46.67 | 0.00 | 0.00 |  |
| chr7:21,170,946-21,213,701 | p15.3 | Allelic Imbalance | 42755 | 59.52 | 13.33 | 46.19 | 0.00 | 0.00 |  |
| chr7:21,405,985-21,440,602 | p15.3 | Allelic Imbalance | 34617 | 59.52 | 13.33 | 46.19 | 0.00 | 0.00 | SP4 |
| chr7:21,490,771-21,501,489 | p15.3 | Allelic Imbalance | 10718 | 59.52 | 13.33 | 46.19 | 0.00 | 0.00 | SP4 |
| chr7:22,681,042-22,720,499 | p15.3 | Allelic Imbalance | 39457 | 59.52 | 13.33 | 46.19 | 0.00 | 0.00 |  |
| chr7:22,758,001-22,789,389 | p15.3 | Allelic Imbalance | 31388 | 59.52 | 13.33 | 46.19 | 0.00 | 0.00 |  |
| chr7:23,672,337-23,695,272 | p15.3 | Allelic Imbalance | 22935 | 66.67 | 20.00 | 46.67 | 0.00 | 0.00 | FAM221A |
| chr7:23,919,456-23,928,891 | p15.3 | Allelic Imbalance | 9435 | 66.67 | 20.00 | 46.67 | 0.00 | 0.00 |  |
| chr7:27,513,350-27,654,219 | p15.2 | Allelic Imbalance | 140869 | 59.52 | 13.33 | 46.19 | 0.00 | 0.00 | HIBADH |
| chr7:27,879,985-27,903,468 | p15.2 | Allelic Imbalance | 23483 | 59.52 | 13.33 | 46.19 | 0.00 | 0.00 | JAZF1 |
| chr7:28,100,428-28,329,390 | p15.1 | Allelic Imbalance | 228962 | 66.67 | 20.00 | 46.67 | 0.00 | 0.00 | JAZF1, JAZF1-AS1, CREB5 |
| chr7:29,369,735-29,378,303 | p15.1 | Allelic Imbalance | 8568 | 59.52 | 13.33 | 46.19 | 0.00 | 0.00 | CHN2 |
| chr7:30,847,962-30,867,030 | p15.1 | Allelic Imbalance | 19068 | 59.52 | 13.33 | 46.19 | 0.00 | 0.00 | INMT-FAM188B, FAM188B |
| chr7:30,919,450-30,953,259 | p15.1 | Allelic Imbalance | 33809 | 59.52 | 13.33 | 46.19 | 0.00 | 0.00 | AQP1 |
| chr7:32,353,333-32,392,044 | p14.3 | Allelic Imbalance | 38711 | 59.52 | 13.33 | 46.19 | 0.00 | 0.00 |  |
| chr7:32,425,818-32,466,042 | p14.3 | Allelic Imbalance | 40224 | 59.52 | 13.33 | 46.19 | 0.00 | 0.00 | LOC100130673 |
| chr7:36,769,075-36,774,956 | p14.2 | Allelic Imbalance | 5881 | 59.52 | 13.33 | 46.19 | 0.00 | 0.00 |  |
| chr7:37,277,852-37,341,670 | p14.2 | Allelic Imbalance | 63818 | 59.52 | 13.33 | 46.19 | 0.00 | 0.00 | ELMO1 |
| chr7:38,382,985-38,459,202 | p14.1 | Allelic Imbalance | 76217 | 59.52 | 13.33 | 46.19 | 0.00 | 0.00 | LOC100506776, AMPH |
| chr7:40,480,004-40,697,708 | p14.1 | Allelic Imbalance | 217704 | 59.52 | 13.33 | 46.19 | 0.00 | 0.00 | C7orf10 |
| chr7:43,962,406-44,131,299 | p13 | Allelic Imbalance | 168893 | 59.52 | 13.33 | 46.19 | 0.00 | 0.00 | POLR2J4, SPDYE1, RASA4CP, FLJ35390, DBNL, PGAM2, POLM, AEBP1, MIR4649, POLD2 |
| chr7:45,286,297-45,572,908 | p13 | Allelic Imbalance | 286611 | 66.67 | 20.00 | 46.67 | 0.00 | 0.00 |  |
| chr7:47,974,644-48,014,877 | p12.3 | Allelic Imbalance | 40233 | 59.52 | 13.33 | 46.19 | 0.00 | 0.00 | HUS1, SUN3 |
| chr7:51,064,589-51,192,173 | p12.1 | Allelic Imbalance | 127584 | 59.52 | 13.33 | 46.19 | 0.00 | 0.00 | COBL |
| chr7:51,253,684-51,355,238 | p12.1 | Allelic Imbalance | 101554 | 59.52 | 13.33 | 46.19 | 0.00 | 0.00 | COBL |
| chr7:51,611,528-51,647,235 | p12.1 | Allelic Imbalance | 35707 | 59.52 | 13.33 | 46.19 | 0.00 | 0.00 |  |
| chr7:63,916,085-64,007,185 | q11.21 | Allelic Imbalance | 91100 | 59.52 | 13.33 | 46.19 | 0.00 | 0.00 | ZNF138, ZNF273 |
| chr7:73,184,116-73,291,039 | q11.23 | Allelic Imbalance | 106923 | 66.67 | 20.00 | 46.67 | 0.00 | 0.00 | EIF4H, MIR590, LAT2, RFC2 |
| chr7:73,355,277-73,510,016 | q11.23 | Allelic Imbalance | 154739 | 66.67 | 20.00 | 46.67 | 0.00 | 0.00 | CLIP2, GTF2IRD1 |
| chr7:9,731,723-9,923,433 | p21.3 | Allelic Imbalance | 191710 | 59.52 | 13.33 | 46.19 | 0.00 | 0.00 |  |
| chr7:50,578,638-50,620,657 | p12.2 | Allelic Imbalance | 42019 | 52.38 | 8.89 | 43.49 | 0.00 | 0.00 | DDC, LOC100129427 |
| chr7:50,626,309-50,652,836 | p12.2 | Allelic Imbalance | 26527 | 52.38 | 8.89 | 43.49 | 0.00 | 0.00 | GRB10 |
| chr7:50,698,143-50,701,192 | p12.2 | Allelic Imbalance | 3049 | 52.38 | 8.89 | 43.49 | 0.00 | 0.00 | GRB10 |
| chr7:54,557,930-54,596,462 | p11.2 | Allelic Imbalance | 38532 | 52.38 | 8.89 | 43.49 | 0.00 | 0.00 | VSTM2A, LOC285878 |
| chr7:65,677,234-65,896,840 | q11.21 | Allelic Imbalance | 219606 | 52.38 | 8.89 | 43.49 | 0.00 | 0.00 | LOC493754, KCTD7, RABGEF1 |
| chr7:18,013,711-18,185,031 | p21.1 | Allelic Imbalance | 171320 | 69.05 | 22.22 | 46.83 | 0.00 | 0.00 | PRPS1L1, HDAC9 |
| chr7:24,246,633-24,268,864 | p15.3 | Allelic Imbalance | 22231 | 69.05 | 22.22 | 46.83 | 0.00 | 0.00 |  |
| chr7:24,763,523-24,859,387 | p15.3 | Allelic Imbalance | 95864 | 69.05 | 22.22 | 46.83 | 0.00 | 0.00 | DFNA5, OSBPL3 |
| chr7:67,509,266-67,662,105 | q11.22 | Allelic Imbalance | 152839 | 69.05 | 22.22 | 46.83 | 0.00 | 0.00 |  |
| chr7:67,903,623-67,942,306 | q11.22 | Allelic Imbalance | 38683 | 69.05 | 22.22 | 46.83 | 0.00 | 0.00 |  |
| chr7:69,751,351-69,759,386 | q11.22 | Allelic Imbalance | 8035 | 69.05 | 22.22 | 46.83 | 0.00 | 0.00 | AUTS2 |
| chr7:70,467,698-70,544,019 | q11.22 | Allelic Imbalance | 76321 | 69.05 | 22.22 | 46.83 | 0.00 | 0.00 | WBSCR17 |
| chr7:150,082,700-150,119,136 | q36.1 | Allelic Imbalance | 36436 | 73.81 | 26.67 | 47.14 | 0.00 | 0.00 |  |
| chr7:150,123,222-150,131,202 | q36.1 | Allelic Imbalance | 7980 | 73.81 | 26.67 | 47.14 | 0.00 | 0.00 | TMEM176B, TMEM176A |
| chr7:18,218,425-18,275,054 | p21.1 | Allelic Imbalance | 56629 | 71.43 | 24.44 | 46.98 | 0.00 | 0.00 | HDAC9 |
| chr10:54,604,331-54,614,317 | q21.1 | Allelic Imbalance | 9986 | 47.62 | 6.67 | 40.95 | 0.00 | 0.00 |  |
| chr7:54,834,444-54,888,319 | p11.2 | Allelic Imbalance | 53875 | 47.62 | 6.67 | 40.95 | 0.00 | 0.00 |  |
| chr7:55,560,082-55,633,578 | p11.2 | Allelic Imbalance | 73496 | 47.62 | 6.67 | 40.95 | 0.00 | 0.00 | VOPP1 |
| chr7:55,796,838-55,862,308 | p11.2 | Allelic Imbalance | 65470 | 47.62 | 6.67 | 40.95 | 0.00 | 0.00 | 14-Sep |
| chr7:56,062,566-56,109,418 | p11.2 | Allelic Imbalance | 46852 | 47.62 | 6.67 | 40.95 | 0.00 | 0.00 | PSPH, CCT6A, SNORA15, SUMF2 |
| chr10:59,735,018-59,994,847 | q21.1 | Allelic Imbalance | 259829 | 42.86 | 4.44 | 38.41 | 0.00 | 0.00 | UBE2D1, TFAM, BICC1 |
| chr10:60,061,821-60,167,540 | q21.1 | Allelic Imbalance | 105719 | 42.86 | 4.44 | 38.41 | 0.00 | 0.00 | BICC1, FAM133CP |
| chr7:15,885,002-15,932,227 | p21.1 | Allelic Imbalance | 47225 | 54.76 | 11.11 | 43.65 | 0.00 | 0.00 |  |
| chr7:37,195,603-37,231,564 | p14.2 | Allelic Imbalance | 35961 | 54.76 | 11.11 | 43.65 | 0.00 | 0.00 | ELMO1 |
| chr7:50,487,892-50,496,460 | p12.2 | Allelic Imbalance | 8568 | 54.76 | 11.11 | 43.65 | 0.00 | 0.00 | DDC |
| chr7:62,084,576-62,201,402 | q11.21 | Allelic Imbalance | 116826 | 54.76 | 11.11 | 43.65 | 0.00 | 0.00 |  |
| chr7:66,058,299-66,086,119 | q11.21 | Allelic Imbalance | 27820 | 54.76 | 11.11 | 43.65 | 0.00 | 0.00 | TMEM248 |
| chr7:10,338,109-10,442,914 | p21.3 | Allelic Imbalance | 104805 | 59.52 | 15.56 | 43.97 | 0.00 | 0.00 |  |
| chr7:10,775,339-11,207,928 | p21.3 | Allelic Imbalance | 432589 | 59.52 | 15.56 | 43.97 | 0.00 | 0.00 | NDUFA4, PHF14 |
| chr7:11,211,524-11,371,445 | p21.3 | Allelic Imbalance | 159921 | 59.52 | 15.56 | 43.97 | 0.00 | 0.00 |  |
| chr7:14,659,095-14,671,355 | p21.2 | Allelic Imbalance | 12260 | 59.52 | 15.56 | 43.97 | 0.00 | 0.00 | DGKB |
| chr7:15,461,629-15,636,775 | p21.1 | Allelic Imbalance | 175146 | 59.52 | 15.56 | 43.97 | 0.00 | 0.00 | AGMO, MEOX2 |
| chr7:17,492,285-17,596,224 | p21.1 | Allelic Imbalance | 103939 | 59.52 | 15.56 | 43.97 | 0.00 | 0.00 |  |
| chr7:17,697,529-17,708,337 | p21.1 | Allelic Imbalance | 10808 | 59.52 | 15.56 | 43.97 | 0.00 | 0.00 |  |
| chr7:19,402,543-19,731,765 | p21.1 - p15.3 | Allelic Imbalance | 329222 | 59.52 | 15.56 | 43.97 | 0.00 | 0.00 | TWISTNB, MIR3146, TMEM196 |
| chr7:22,666,077-22,681,042 | p15.3 | Allelic Imbalance | 14965 | 59.52 | 15.56 | 43.97 | 0.00 | 0.00 |  |
| chr7:22,789,389-22,865,137 | p15.3 | Allelic Imbalance | 75748 | 59.52 | 15.56 | 43.97 | 0.00 | 0.00 | TOMM7, SNORD93 |
| chr7:25,278,822-25,307,248 | p15.2 | Allelic Imbalance | 28426 | 59.52 | 15.56 | 43.97 | 0.00 | 0.00 |  |
| chr7:27,240,938-27,367,698 | p15.2 | Allelic Imbalance | 126760 | 59.52 | 15.56 | 43.97 | 0.00 | 0.00 | EVX1 |
| chr7:27,499,035-27,513,350 | p15.2 | Allelic Imbalance | 14315 | 59.52 | 15.56 | 43.97 | 0.00 | 0.00 |  |
| chr7:29,485,324-29,504,719 | p15.1 | Allelic Imbalance | 19395 | 59.52 | 15.56 | 43.97 | 0.00 | 0.00 | CHN2 |
| chr7:29,624,969-29,825,743 | p15.1 | Allelic Imbalance | 200774 | 59.52 | 15.56 | 43.97 | 0.00 | 0.00 | LOC646762, MIR550A3, ZNRF2P2, DPY19L2P3, WIPF3 |
| chr7:29,908,550-29,919,563 | p15.1 | Allelic Imbalance | 11013 | 59.52 | 15.56 | 43.97 | 0.00 | 0.00 | WIPF3 |
| chr7:30,728,972-30,737,908 | p15.1 | Allelic Imbalance | 8936 | 59.52 | 15.56 | 43.97 | 0.00 | 0.00 |  |
| chr7:30,817,488-30,847,962 | p15.1 | Allelic Imbalance | 30474 | 59.52 | 15.56 | 43.97 | 0.00 | 0.00 | INMT-FAM188B, FAM188B |
| chr7:30,953,259-30,954,073 | p15.1 | Allelic Imbalance | 814 | 59.52 | 15.56 | 43.97 | 0.00 | 0.00 |  |
| chr7:32,010,313-32,017,261 | p14.3 | Allelic Imbalance | 6948 | 59.52 | 15.56 | 43.97 | 0.00 | 0.00 | PDE1C |
| chr7:32,174,464-32,353,333 | p14.3 | Allelic Imbalance | 178869 | 59.52 | 15.56 | 43.97 | 0.00 | 0.00 | PDE1C |
| chr7:33,308,441-33,437,332 | p14.3 | Allelic Imbalance | 128891 | 59.52 | 15.56 | 43.97 | 0.00 | 0.00 | BBS9 |
| chr7:38,573,586-38,593,291 | p14.1 | Allelic Imbalance | 19705 | 59.52 | 15.56 | 43.97 | 0.00 | 0.00 | AMPH |
| chr7:38,682,386-38,747,997 | p14.1 | Allelic Imbalance | 65611 | 59.52 | 15.56 | 43.97 | 0.00 | 0.00 | FAM183B, VPS41 |
| chr7:39,851,800-39,869,503 | p14.1 | Allelic Imbalance | 17703 | 59.52 | 15.56 | 43.97 | 0.00 | 0.00 |  |
| chr7:43,571,582-43,603,608 | p13 | Allelic Imbalance | 32026 | 59.52 | 15.56 | 43.97 | 0.00 | 0.00 | STK17A |
| chr7:43,827,456-43,906,419 | p13 | Allelic Imbalance | 78963 | 59.52 | 15.56 | 43.97 | 0.00 | 0.00 | MRPS24, URGCP-MRPS24, URGCP |
| chr7:44,184,021-44,189,161 | p13 | Allelic Imbalance | 5140 | 59.52 | 15.56 | 43.97 | 0.00 | 0.00 | GCK |
| chr7:48,070,152-48,251,630 | p12.3 | Allelic Imbalance | 181478 | 59.52 | 15.56 | 43.97 | 0.00 | 0.00 | UPP1, ABCA13 |
| chr7:48,269,866-48,292,400 | p12.3 | Allelic Imbalance | 22534 | 59.52 | 15.56 | 43.97 | 0.00 | 0.00 | ABCA13 |
| chr7:51,192,173-51,253,684 | p12.1 | Allelic Imbalance | 61511 | 59.52 | 15.56 | 43.97 | 0.00 | 0.00 | COBL |
| chr7:79,976,074-80,077,643 | q21.11 | Allelic Imbalance | 101569 | 59.52 | 15.56 | 43.97 | 0.00 | 0.00 | GNAT3, CD36 |
| chr7:80,120,174-80,127,909 | q21.11 | Allelic Imbalance | 7735 | 59.52 | 15.56 | 43.97 | 0.00 | 0.00 | CD36 |
| chr7:11,588,126-11,592,580 | p21.3 | Allelic Imbalance | 4454 | 61.90 | 17.78 | 44.13 | 0.00 | 0.00 | THSD7A |
| chr7:11,825,303-11,834,144 | p21.3 | Allelic Imbalance | 8841 | 61.90 | 17.78 | 44.13 | 0.00 | 0.00 | THSD7A |
| chr7:12,108,903-12,198,433 | p21.3 | Allelic Imbalance | 89530 | 61.90 | 17.78 | 44.13 | 0.00 | 0.00 |  |
| chr7:12,398,378-12,460,048 | p21.3 | Allelic Imbalance | 61670 | 61.90 | 17.78 | 44.13 | 0.00 | 0.00 | VWDE |
| chr7:12,655,524-12,671,897 | p21.3 | Allelic Imbalance | 16373 | 61.90 | 17.78 | 44.13 | 0.00 | 0.00 | SCIN |
| chr7:12,699,495-13,218,344 | p21.3 | Allelic Imbalance | 518849 | 61.90 | 17.78 | 44.13 | 0.00 | 0.00 |  |
| chr7:14,237,951-14,244,866 | p21.2 | Allelic Imbalance | 6915 | 61.90 | 17.78 | 44.13 | 0.00 | 0.00 | DGKB |
| chr7:17,471,812-17,471,992 | p21.1 | Allelic Imbalance | 180 | 61.90 | 17.78 | 44.13 | 0.00 | 0.00 |  |
| chr7:17,711,698-17,741,772 | p21.1 | Allelic Imbalance | 30074 | 61.90 | 17.78 | 44.13 | 0.00 | 0.00 |  |
| chr7:22,877,287-22,902,076 | p15.3 | Allelic Imbalance | 24789 | 61.90 | 17.78 | 44.13 | 0.00 | 0.00 |  |
| chr7:23,366,369-23,386,698 | p15.3 | Allelic Imbalance | 20329 | 61.90 | 17.78 | 44.13 | 0.00 | 0.00 | IGF2BP3 |
| chr7:23,401,419-23,515,124 | p15.3 | Allelic Imbalance | 113705 | 61.90 | 17.78 | 44.13 | 0.00 | 0.00 | IGF2BP3, RPS2P32, TRA2A |
| chr7:26,796,849-27,037,561 | p15.2 | Allelic Imbalance | 240712 | 61.90 | 17.78 | 44.13 | 0.00 | 0.00 | SKAP2 |
| chr7:27,919,830-27,961,327 | p15.2 | Allelic Imbalance | 41497 | 61.90 | 17.78 | 44.13 | 0.00 | 0.00 | JAZF1 |
| chr7:28,416,523-28,460,895 | p15.1 | Allelic Imbalance | 44372 | 61.90 | 17.78 | 44.13 | 0.00 | 0.00 | CREB5 |
| chr7:30,967,845-30,973,998 | p15.1 | Allelic Imbalance | 6153 | 61.90 | 17.78 | 44.13 | 0.00 | 0.00 | GHRHR |
| chr7:39,545,124-39,811,304 | p14.1 | Allelic Imbalance | 266180 | 61.90 | 17.78 | 44.13 | 0.00 | 0.00 | YAE1D1, LOC646999, RALA, LINC00265 |
| chr7:41,963,372-42,004,998 | p14.1 | Allelic Imbalance | 41626 | 61.90 | 17.78 | 44.13 | 0.00 | 0.00 | GLI3 |
| chr7:45,006,088-45,021,796 | p13 | Allelic Imbalance | 15708 | 61.90 | 17.78 | 44.13 | 0.00 | 0.00 | CCM2 |
| chr7:45,118,802-45,204,789 | p13 | Allelic Imbalance | 85987 | 61.90 | 17.78 | 44.13 | 0.00 | 0.00 | RAMP3 |
| chr7:80,134,799-80,211,139 | q21.11 | Allelic Imbalance | 76340 | 61.90 | 17.78 | 44.13 | 0.00 | 0.00 | CD36, SEMA3C |
| chr7:10,133,371-10,185,686 | p21.3 | Allelic Imbalance | 52315 | 57.14 | 13.33 | 43.81 | 0.00 | 0.00 |  |
| chr7:10,302,444-10,322,852 | p21.3 | Allelic Imbalance | 20408 | 57.14 | 13.33 | 43.81 | 0.00 | 0.00 |  |
| chr7:16,089,440-16,246,528 | p21.1 | Allelic Imbalance | 157088 | 57.14 | 13.33 | 43.81 | 0.00 | 0.00 | LOC100506025, ISPD |
| chr7:21,213,701-21,405,985 | p15.3 | Allelic Imbalance | 192284 | 57.14 | 13.33 | 43.81 | 0.00 | 0.00 |  |
| chr7:21,440,602-21,490,771 | p15.3 | Allelic Imbalance | 50169 | 57.14 | 13.33 | 43.81 | 0.00 | 0.00 | SP4 |
| chr7:22,720,499-22,723,806 | p15.3 | Allelic Imbalance | 3307 | 57.14 | 13.33 | 43.81 | 0.00 | 0.00 |  |
| chr7:22,735,472-22,758,001 | p15.3 | Allelic Imbalance | 22529 | 57.14 | 13.33 | 43.81 | 0.00 | 0.00 | IL6 |
| chr7:30,867,030-30,919,450 | p15.1 | Allelic Imbalance | 52420 | 57.14 | 13.33 | 43.81 | 0.00 | 0.00 | INMT-FAM188B, FAM188B, AQP1 |
| chr7:36,774,956-36,816,524 | p14.2 | Allelic Imbalance | 41568 | 57.14 | 13.33 | 43.81 | 0.00 | 0.00 |  |
| chr7:37,266,962-37,277,852 | p14.2 | Allelic Imbalance | 10890 | 57.14 | 13.33 | 43.81 | 0.00 | 0.00 | ELMO1 |
| chr7:40,376,865-40,480,004 | p14.1 | Allelic Imbalance | 103139 | 57.14 | 13.33 | 43.81 | 0.00 | 0.00 | C7orf10 |
| chr7:64,007,185-64,041,639 | q11.21 | Allelic Imbalance | 34454 | 57.14 | 13.33 | 43.81 | 0.00 | 0.00 | ZNF273 |
| chr7:64,064,269-64,454,218 | q11.21 | Allelic Imbalance | 389949 | 57.14 | 13.33 | 43.81 | 0.00 | 0.00 | ZNF117, ERV3-1, CCT6P3 |
| chr7:9,923,433-9,956,694 | p21.3 | Allelic Imbalance | 33261 | 57.14 | 13.33 | 43.81 | 0.00 | 0.00 |  |
| chr7:22,999,391-23,092,388 | p15.3 | Allelic Imbalance | 92997 | 64.29 | 20.00 | 44.29 | 0.00 | 0.00 | FAM126A |
| chr7:23,319,542-23,343,897 | p15.3 | Allelic Imbalance | 24355 | 64.29 | 20.00 | 44.29 | 0.00 | 0.00 | IGF2BP3 |
| chr7:23,569,781-23,672,337 | p15.3 | Allelic Imbalance | 102556 | 64.29 | 20.00 | 44.29 | 0.00 | 0.00 | CLK2P, CCDC126 |
| chr7:23,695,272-23,919,456 | p15.3 | Allelic Imbalance | 224184 | 64.29 | 20.00 | 44.29 | 0.00 | 0.00 | FAM221A, STK31 |
| chr7:28,329,390-28,333,198 | p15.1 | Allelic Imbalance | 3808 | 64.29 | 20.00 | 44.29 | 0.00 | 0.00 | CREB5 |
| chr7:54,596,462-54,663,589 | p11.2 | Allelic Imbalance | 67127 | 50.00 | 8.89 | 41.11 | 0.00 | 0.00 | VSTM2A, LOC285878 |
| chr7:65,007,623-65,122,532 | q11.21 | Allelic Imbalance | 114909 | 50.00 | 8.89 | 41.11 | 0.00 | 0.00 | VKORC1L1, GUSB |
| chr7:65,156,280-65,677,234 | q11.21 | Allelic Imbalance | 520954 | 50.00 | 8.89 | 41.11 | 0.00 | 0.00 | ASL, CRCP, TPST1, LINC00174, LOC493754 |
| chr7:68,154,771-68,160,972 | q11.22 | Allelic Imbalance | 6201 | 64.29 | 20.00 | 44.29 | 0.00 | 0.00 |  |
| chr7:73,117,754-73,156,969 | q11.23 | Allelic Imbalance | 39215 | 64.29 | 20.00 | 44.29 | 0.00 | 0.00 | ELN, LIMK1 |
| chr7:73,159,597-73,184,116 | q11.23 | Allelic Imbalance | 24519 | 64.29 | 20.00 | 44.29 | 0.00 | 0.00 | LIMK1 |
| chr7:73,510,016-73,612,283 | q11.23 | Allelic Imbalance | 102267 | 64.29 | 20.00 | 44.29 | 0.00 | 0.00 | GTF2IRD1 |
| chr7:73,695,900-73,832,620 | q11.23 | Allelic Imbalance | 136720 | 64.29 | 20.00 | 44.29 | 0.00 | 0.00 | GTF2I, NCF1 |
| chr7:79,922,849-79,937,083 | q21.11 | Allelic Imbalance | 14234 | 64.29 | 20.00 | 44.29 | 0.00 | 0.00 | GNAT3 |
| chr7:82,755,956-82,858,101 | q21.11 | Allelic Imbalance | 102145 | 64.29 | 20.00 | 44.29 | 0.00 | 0.00 | SEMA3E |
| chr7:17,993,188-18,013,711 | p21.1 | Allelic Imbalance | 20523 | 66.67 | 22.22 | 44.44 | 0.00 | 0.00 |  |
| chr7:24,268,864-24,278,536 | p15.3 | Allelic Imbalance | 9672 | 66.67 | 22.22 | 44.44 | 0.00 | 0.00 |  |
| chr7:28,615,981-28,671,364 | p15.1 | Allelic Imbalance | 55383 | 66.67 | 22.22 | 44.44 | 0.00 | 0.00 | CREB5 |
| chr7:31,782,540-31,788,707 | p15.1 | Allelic Imbalance | 6167 | 66.67 | 22.22 | 44.44 | 0.00 | 0.00 | PDE1C |
| chr7:67,942,306-67,993,274 | q11.22 | Allelic Imbalance | 50968 | 66.67 | 22.22 | 44.44 | 0.00 | 0.00 |  |
| chr7:69,746,779-69,751,351 | q11.22 | Allelic Imbalance | 4572 | 66.67 | 22.22 | 44.44 | 0.00 | 0.00 | AUTS2 |
| chr7:69,759,386-69,782,896 | q11.22 | Allelic Imbalance | 23510 | 66.67 | 22.22 | 44.44 | 0.00 | 0.00 | AUTS2 |
| chr7:70,357,280-70,378,352 | q11.22 | Allelic Imbalance | 21072 | 66.67 | 22.22 | 44.44 | 0.00 | 0.00 | WBSCR17 |
| chr7:70,438,989-70,467,698 | q11.22 | Allelic Imbalance | 28709 | 66.67 | 22.22 | 44.44 | 0.00 | 0.00 | WBSCR17 |
| chr7:70,544,019-70,590,464 | q11.22 | Allelic Imbalance | 46445 | 66.67 | 22.22 | 44.44 | 0.00 | 0.00 | WBSCR17 |
| chr7:70,813,658-70,954,838 | q11.22 | Allelic Imbalance | 141180 | 66.67 | 22.22 | 44.44 | 0.00 | 0.00 | WBSCR17, CALN1 |
| chr7:73,291,039-73,355,277 | q11.23 | Allelic Imbalance | 64238 | 66.67 | 22.22 | 44.44 | 0.00 | 0.00 | RFC2, CLIP2 |
| chr7:82,604,151-82,699,093 | q21.11 | Allelic Imbalance | 94942 | 66.67 | 22.22 | 44.44 | 0.00 | 0.00 | PCLO |
| chr7:155,454,286-155,694,205 | q36.3 | Allelic Imbalance | 239919 | 73.81 | 28.89 | 44.92 | 0.00 | 0.00 |  |
| chr7:31,768,622-31,782,540 | p15.1 | Allelic Imbalance | 13918 | 69.05 | 24.44 | 44.60 | 0.00 | 0.00 | PDE1C |
| chr7:71,148,476-71,306,179 | q11.22 | Allelic Imbalance | 157703 | 69.05 | 24.44 | 44.60 | 0.00 | 0.00 | CALN1 |
| chr7:150,131,202-150,228,513 | q36.1 | Allelic Imbalance | 97311 | 71.43 | 26.67 | 44.76 | 0.00 | 0.00 | TMEM176A, ABP1 |
| chr10:54,309,563-54,604,331 | q21.1 | Allelic Imbalance | 294768 | 45.24 | 6.67 | 38.57 | 0.00 | 0.00 |  |
| chr10:55,468,953-55,496,482 | q21.1 | Allelic Imbalance | 27529 | 45.24 | 6.67 | 38.57 | 0.00 | 0.00 | PCDH15 |
| chr10:55,529,635-55,595,127 | q21.1 | Allelic Imbalance | 65492 | 45.24 | 6.67 | 38.57 | 0.00 | 0.00 | PCDH15 |
| chr10:55,621,439-55,673,814 | q21.1 | Allelic Imbalance | 52375 | 45.24 | 6.67 | 38.57 | 0.00 | 0.00 | PCDH15 |
| chr7:54,888,319-55,112,125 | p11.2 | Allelic Imbalance | 223806 | 45.24 | 6.67 | 38.57 | 0.00 | 0.00 | EGFR |
| chr7:55,368,981-55,378,896 | p11.2 | Allelic Imbalance | 9915 | 45.24 | 6.67 | 38.57 | 0.00 | 0.00 |  |
| chr7:55,534,711-55,560,082 | p11.2 | Allelic Imbalance | 25371 | 45.24 | 6.67 | 38.57 | 0.00 | 0.00 | VOPP1 |
| chr7:55,862,308-56,062,566 | p11.2 | Allelic Imbalance | 200258 | 45.24 | 6.67 | 38.57 | 0.00 | 0.00 | SEPT14, ZNF713, MRPS17, GBAS, PSPH |
| chr7:86,971,354-86,989,374 | q21.12 | Allelic Imbalance | 18020 | 45.24 | 6.67 | 38.57 | 0.00 | 0.00 | ABCB1 |
| chr10:59,025,460-59,132,718 | q21.1 | Allelic Imbalance | 107258 | 35.71 | 2.22 | 33.49 | 0.00 | 0.00 |  |
| chr10:59,716,198-59,735,018 | q21.1 | Allelic Imbalance | 18820 | 40.48 | 4.44 | 36.03 | 0.00 | 0.00 | CISD1 |
| chr10:59,994,847-60,061,821 | q21.1 | Allelic Imbalance | 66974 | 40.48 | 4.44 | 36.03 | 0.00 | 0.00 | BICC1 |
| chr10:60,167,540-60,384,659 | q21.1 | Allelic Imbalance | 217119 | 40.48 | 4.44 | 36.03 | 0.00 | 0.00 | BICC1 |
| chr10:60,531,514-60,554,850 | q21.1 | Allelic Imbalance | 23336 | 40.48 | 4.44 | 36.03 | 0.00 | 0.00 |  |
| chr10:63,142,274-63,177,218 | q21.2 | Allelic Imbalance | 34944 | 40.48 | 4.44 | 36.03 | 0.00 | 0.00 | C10orf107 |
| chr7:50,496,460-50,578,638 | p12.2 | Allelic Imbalance | 82178 | 52.38 | 11.11 | 41.27 | 0.00 | 0.00 | DDC, LOC100129427 |
| chr7:50,652,836-50,666,845 | p12.2 | Allelic Imbalance | 14009 | 52.38 | 11.11 | 41.27 | 0.00 | 0.00 | GRB10 |
| chr7:50,671,632-50,698,143 | p12.2 | Allelic Imbalance | 26511 | 52.38 | 11.11 | 41.27 | 0.00 | 0.00 | GRB10 |
| chr7:10,322,852-10,338,109 | p21.3 | Allelic Imbalance | 15257 | 57.14 | 15.56 | 41.59 | 0.00 | 0.00 |  |
| chr7:14,338,871-14,343,016 | p21.2 | Allelic Imbalance | 4145 | 57.14 | 15.56 | 41.59 | 0.00 | 0.00 | DGKB |
| chr7:14,646,648-14,659,095 | p21.2 | Allelic Imbalance | 12447 | 57.14 | 15.56 | 41.59 | 0.00 | 0.00 | DGKB |
| chr7:17,596,224-17,694,541 | p21.1 | Allelic Imbalance | 98317 | 57.14 | 15.56 | 41.59 | 0.00 | 0.00 |  |
| chr7:17,695,624-17,697,529 | p21.1 | Allelic Imbalance | 1905 | 57.14 | 15.56 | 41.59 | 0.00 | 0.00 |  |
| chr7:27,367,698-27,443,901 | p15.2 | Allelic Imbalance | 76203 | 57.14 | 15.56 | 41.59 | 0.00 | 0.00 |  |
| chr7:27,453,036-27,499,035 | p15.2 | Allelic Imbalance | 45999 | 57.14 | 15.56 | 41.59 | 0.00 | 0.00 |  |
| chr7:29,504,719-29,624,969 | p15.1 | Allelic Imbalance | 120250 | 57.14 | 15.56 | 41.59 | 0.00 | 0.00 | CHN2, PRR15 |
| chr7:29,919,563-30,151,713 | p15.1 | Allelic Imbalance | 232150 | 57.14 | 15.56 | 41.59 | 0.00 | 0.00 | WIPF3, SCRN1, FKBP14, PLEKHA8, C7orf41 |
| chr7:30,399,028-30,415,251 | p15.1 | Allelic Imbalance | 16223 | 57.14 | 15.56 | 41.59 | 0.00 | 0.00 |  |
| chr7:30,712,192-30,728,972 | p15.1 | Allelic Imbalance | 16780 | 57.14 | 15.56 | 41.59 | 0.00 | 0.00 |  |
| chr7:32,006,858-32,010,313 | p14.3 | Allelic Imbalance | 3455 | 57.14 | 15.56 | 41.59 | 0.00 | 0.00 | PDE1C |
| chr7:32,017,261-32,174,464 | p14.3 | Allelic Imbalance | 157203 | 57.14 | 15.56 | 41.59 | 0.00 | 0.00 | PDE1C |
| chr7:39,869,503-39,896,083 | p14.1 | Allelic Imbalance | 26580 | 57.14 | 15.56 | 41.59 | 0.00 | 0.00 |  |
| chr7:40,338,391-40,376,865 | p14.1 | Allelic Imbalance | 38474 | 57.14 | 15.56 | 41.59 | 0.00 | 0.00 | C7orf10 |
| chr7:44,318,700-44,538,490 | p13 | Allelic Imbalance | 219790 | 57.14 | 15.56 | 41.59 | 0.00 | 0.00 | CAMK2B, NUDCD3, NPC1L1 |
| chr7:48,251,630-48,269,866 | p12.3 | Allelic Imbalance | 18236 | 57.14 | 15.56 | 41.59 | 0.00 | 0.00 | ABCA13 |
| chr7:75,863,445-75,926,859 | q11.23 | Allelic Imbalance | 63414 | 57.14 | 15.56 | 41.59 | 0.00 | 0.00 | SRCRB4D, ZP3 |
| chr7:10,442,914-10,608,887 | p21.3 | Allelic Imbalance | 165973 | 59.52 | 17.78 | 41.75 | 0.00 | 0.00 |  |
| chr7:10,707,069-10,775,339 | p21.3 | Allelic Imbalance | 68270 | 59.52 | 17.78 | 41.75 | 0.00 | 0.00 |  |
| chr7:11,371,445-11,490,785 | p21.3 | Allelic Imbalance | 119340 | 59.52 | 17.78 | 41.75 | 0.00 | 0.00 | THSD7A |
| chr7:11,513,420-11,586,087 | p21.3 | Allelic Imbalance | 72667 | 59.52 | 17.78 | 41.75 | 0.00 | 0.00 | THSD7A |
| chr7:11,587,221-11,588,126 | p21.3 | Allelic Imbalance | 905 | 59.52 | 17.78 | 41.75 | 0.00 | 0.00 | THSD7A |
| chr7:11,834,144-11,855,660 | p21.3 | Allelic Imbalance | 21516 | 59.52 | 17.78 | 41.75 | 0.00 | 0.00 | THSD7A |
| chr7:12,081,420-12,108,903 | p21.3 | Allelic Imbalance | 27483 | 59.52 | 17.78 | 41.75 | 0.00 | 0.00 |  |
| chr7:12,198,433-12,398,378 | p21.3 | Allelic Imbalance | 199945 | 59.52 | 17.78 | 41.75 | 0.00 | 0.00 | TMEM106B, VWDE |
| chr7:12,460,048-12,484,016 | p21.3 | Allelic Imbalance | 23968 | 59.52 | 17.78 | 41.75 | 0.00 | 0.00 |  |
| chr7:14,244,866-14,253,868 | p21.2 | Allelic Imbalance | 9002 | 59.52 | 17.78 | 41.75 | 0.00 | 0.00 | DGKB |
| chr7:17,471,992-17,492,285 | p21.1 | Allelic Imbalance | 20293 | 59.52 | 17.78 | 41.75 | 0.00 | 0.00 |  |
| chr7:23,386,698-23,401,419 | p15.3 | Allelic Imbalance | 14721 | 59.52 | 17.78 | 41.75 | 0.00 | 0.00 | IGF2BP3 |
| chr7:27,037,561-27,170,310 | p15.2 | Allelic Imbalance | 132749 | 59.52 | 17.78 | 41.75 | 0.00 | 0.00 | HOXA1, HOTAIRM1, HOXA2, HOXA3, HOXA4, HOXA5, HOXA6, HOXA-AS3, HOXA7, HOXA9, HOXA10-HOXA9 |
| chr7:27,210,694-27,240,938 | p15.2 | Allelic Imbalance | 30244 | 59.52 | 17.78 | 41.75 | 0.00 | 0.00 | HOTTIP |
| chr7:30,238,804-30,399,028 | p15.1 | Allelic Imbalance | 160224 | 59.52 | 17.78 | 41.75 | 0.00 | 0.00 | MIR550A1, MIR550B1, ZNRF2, DKFZP586I1420 |
| chr7:44,818,865-44,948,337 | p13 | Allelic Imbalance | 129472 | 59.52 | 17.78 | 41.75 | 0.00 | 0.00 | H2AFV, PURB, MIR4657 |
| chr7:22,723,806-22,735,472 | p15.3 | Allelic Imbalance | 11666 | 54.76 | 13.33 | 41.43 | 0.00 | 0.00 | IL6 |
| chr7:36,816,524-36,854,706 | p14.2 | Allelic Imbalance | 38182 | 54.76 | 13.33 | 41.43 | 0.00 | 0.00 |  |
| chr7:37,117,378-37,195,603 | p14.2 | Allelic Imbalance | 78225 | 54.76 | 13.33 | 41.43 | 0.00 | 0.00 | ELMO1 |
| chr7:64,041,639-64,064,269 | q11.21 | Allelic Imbalance | 22630 | 54.76 | 13.33 | 41.43 | 0.00 | 0.00 |  |
| chr7:66,086,119-66,133,800 | q11.21 - q11.22 | Allelic Imbalance | 47681 | 54.76 | 13.33 | 41.43 | 0.00 | 0.00 | SBDS, TYW1 |
| chr10:54,614,317-54,964,923 | q21.1 | Allelic Imbalance | 350606 | 47.62 | 8.89 | 38.73 | 0.00 | 0.00 |  |
| chr7:65,122,532-65,156,280 | q11.21 | Allelic Imbalance | 33748 | 47.62 | 8.89 | 38.73 | 0.00 | 0.00 |  |
| chr7:12,624,995-12,655,524 | p21.3 | Allelic Imbalance | 30529 | 61.90 | 20.00 | 41.90 | 0.00 | 0.00 | SCIN |
| chr7:22,902,076-22,999,391 | p15.3 | Allelic Imbalance | 97315 | 61.90 | 20.00 | 41.90 | 0.00 | 0.00 | FAM126A |
| chr7:23,185,379-23,319,542 | p15.3 | Allelic Imbalance | 134163 | 61.90 | 20.00 | 41.90 | 0.00 | 0.00 | NUPL2, GPNMB, MALSU1, IGF2BP3 |
| chr7:28,460,895-28,538,065 | p15.1 | Allelic Imbalance | 77170 | 61.90 | 20.00 | 41.90 | 0.00 | 0.00 | CREB5 |
| chr7:68,160,972-68,271,070 | q11.22 | Allelic Imbalance | 110098 | 61.90 | 20.00 | 41.90 | 0.00 | 0.00 |  |
| chr7:73,612,283-73,695,900 | q11.23 | Allelic Imbalance | 83617 | 61.90 | 20.00 | 41.90 | 0.00 | 0.00 | GTF2IRD1 |
| chr7:75,086,498-75,160,600 | q11.23 | Allelic Imbalance | 74102 | 61.90 | 20.00 | 41.90 | 0.00 | 0.00 | HIP1 |
| chr7:75,212,324-75,237,031 | q11.23 | Allelic Imbalance | 24707 | 61.90 | 20.00 | 41.90 | 0.00 | 0.00 | CCL26 |
| chr7:76,310,444-76,592,220 | q11.23 | Allelic Imbalance | 281776 | 61.90 | 20.00 | 41.90 | 0.00 | 0.00 | DTX2P1-UPK3BP1-PMS2P11, LOC100132832, CCDC146 |
| chr7:79,937,083-79,976,074 | q21.11 | Allelic Imbalance | 38991 | 61.90 | 20.00 | 41.90 | 0.00 | 0.00 | GNAT3 |
| chr7:80,211,139-80,273,682 | q21.11 | Allelic Imbalance | 62543 | 61.90 | 20.00 | 41.90 | 0.00 | 0.00 | SEMA3C |
| chr7:112,503,253-112,504,151 | q31.1 | Allelic Imbalance | 898 | 64.29 | 22.22 | 42.06 | 0.00 | 0.00 |  |
| chr7:139,102,805-139,104,971 | q34 | Allelic Imbalance | 2166 | 64.29 | 22.22 | 42.06 | 0.00 | 0.00 | HIPK2 |
| chr7:28,612,305-28,615,981 | p15.1 | Allelic Imbalance | 3676 | 64.29 | 22.22 | 42.06 | 0.00 | 0.00 | CREB5 |
| chr7:68,146,692-68,154,771 | q11.22 | Allelic Imbalance | 8079 | 64.29 | 22.22 | 42.06 | 0.00 | 0.00 |  |
| chr7:69,707,735-69,746,779 | q11.22 | Allelic Imbalance | 39044 | 64.29 | 22.22 | 42.06 | 0.00 | 0.00 | AUTS2 |
| chr7:69,782,896-69,867,807 | q11.22 | Allelic Imbalance | 84911 | 64.29 | 22.22 | 42.06 | 0.00 | 0.00 | AUTS2 |
| chr7:70,164,529-70,357,280 | q11.22 | Allelic Imbalance | 192751 | 64.29 | 22.22 | 42.06 | 0.00 | 0.00 | WBSCR17 |
| chr7:70,663,106-70,813,658 | q11.22 | Allelic Imbalance | 150552 | 64.29 | 22.22 | 42.06 | 0.00 | 0.00 | WBSCR17 |
| chr7:70,954,838-71,031,973 | q11.22 | Allelic Imbalance | 77135 | 64.29 | 22.22 | 42.06 | 0.00 | 0.00 | CALN1 |
| chr7:71,641,261-71,789,465 | q11.22 | Allelic Imbalance | 148204 | 64.29 | 22.22 | 42.06 | 0.00 | 0.00 | TYW1B |
| chr7:72,890,453-73,117,754 | q11.23 | Allelic Imbalance | 227301 | 64.29 | 22.22 | 42.06 | 0.00 | 0.00 | WBSCR27, WBSCR28, ELN |
| chr7:73,832,620-75,007,035 | q11.23 | Allelic Imbalance | 1174415 | 64.29 | 22.22 | 42.06 | 0.00 | 0.00 | NCF1, GTF2IRD2, STAG3L2, PMS2P5, GATSL1, WBSCR16, GTF2IRD2B, NCF1C, GTF2IP1, LOC100093631, GATSL2, SPDYE8P, SPDYE8P, PMS2L2, STAG3L1, LOC541473, TRIM73, NSUN5P1, POM121C, SPDYE5, PMS2P3, HIP1 |
| chr7:79,705,358-79,721,748 | q21.11 | Allelic Imbalance | 16390 | 64.29 | 22.22 | 42.06 | 0.00 | 0.00 |  |
| chr7:79,754,309-79,922,849 | q21.11 | Allelic Imbalance | 168540 | 64.29 | 22.22 | 42.06 | 0.00 | 0.00 |  |
| chr7:82,600,261-82,604,151 | q21.11 | Allelic Imbalance | 3890 | 64.29 | 22.22 | 42.06 | 0.00 | 0.00 | PCLO |
| chr7:82,699,093-82,755,956 | q21.11 | Allelic Imbalance | 56863 | 64.29 | 22.22 | 42.06 | 0.00 | 0.00 |  |
| chr7:82,858,101-82,926,656 | q21.11 | Allelic Imbalance | 68555 | 64.29 | 22.22 | 42.06 | 0.00 | 0.00 | SEMA3E |
| chr7:155,694,205-155,718,090 | q36.3 | Allelic Imbalance | 23885 | 73.81 | 31.11 | 42.70 | 0.00 | 0.00 |  |
| chr7:67,993,274-68,066,204 | q11.22 | Allelic Imbalance | 72930 | 66.67 | 24.44 | 42.22 | 0.00 | 0.00 |  |
| chr7:70,378,352-70,438,989 | q11.22 | Allelic Imbalance | 60637 | 66.67 | 24.44 | 42.22 | 0.00 | 0.00 | MIR3914-1, MIR3914-2, WBSCR17 |
| chr7:71,134,418-71,148,476 | q11.22 | Allelic Imbalance | 14058 | 66.67 | 24.44 | 42.22 | 0.00 | 0.00 | CALN1 |
| chr7:71,306,179-71,434,554 | q11.22 | Allelic Imbalance | 128375 | 66.67 | 24.44 | 42.22 | 0.00 | 0.00 | CALN1 |
| chr7:82,572,283-82,600,261 | q21.11 | Allelic Imbalance | 27978 | 66.67 | 24.44 | 42.22 | 0.00 | 0.00 | PCLO |
| chr7:139,317,236-139,331,338 | q34 | Allelic Imbalance | 14102 | 69.05 | 26.67 | 42.38 | 0.00 | 0.00 | TBXAS1 |
| chr7:139,331,338-139,352,208 | q34 | Allelic Imbalance | 20870 | 71.43 | 28.89 | 42.54 | 0.00 | 0.00 | TBXAS1 |
| chr7:139,709,945-139,780,488 | q34 | Allelic Imbalance | 70543 | 69.05 | 26.67 | 42.38 | 0.00 | 0.00 | SLC37A3, RAB19 |
| chr7:148,129,191-148,303,811 | q36.1 | Allelic Imbalance | 174620 | 69.05 | 26.67 | 42.38 | 0.00 | 0.00 | EZH2 |
| chr7:148,396,034-148,421,975 | q36.1 | Allelic Imbalance | 25941 | 69.05 | 26.67 | 42.38 | 0.00 | 0.00 | ZNF786 |
| chr7:150,073,353-150,082,700 | q36.1 | Allelic Imbalance | 9347 | 69.05 | 26.67 | 42.38 | 0.00 | 0.00 |  |
| chr7:150,228,513-150,230,186 | q36.1 | Allelic Imbalance | 1673 | 69.05 | 26.67 | 42.38 | 0.00 | 0.00 |  |
| chr7:155,438,978-155,454,286 | q36.3 | Allelic Imbalance | 15308 | 71.43 | 28.89 | 42.54 | 0.00 | 0.00 |  |
| chr10:55,307,693-55,404,303 | q21.1 | Allelic Imbalance | 96610 | 42.86 | 6.67 | 36.19 | 0.00 | 0.00 | PCDH15 |
| chr10:55,496,482-55,529,635 | q21.1 | Allelic Imbalance | 33153 | 42.86 | 6.67 | 36.19 | 0.00 | 0.00 | PCDH15 |
| chr10:55,673,814-55,693,823 | q21.1 | Allelic Imbalance | 20009 | 42.86 | 6.67 | 36.19 | 0.00 | 0.00 | PCDH15 |
| chr10:61,053,026-61,057,077 | q21.1 | Allelic Imbalance | 4051 | 42.86 | 6.67 | 36.19 | 0.00 | 0.00 |  |
| chr7:55,112,125-55,123,739 | p11.2 | Allelic Imbalance | 11614 | 42.86 | 6.67 | 36.19 | 0.00 | 0.00 | EGFR |
| chr7:55,378,896-55,405,856 | p11.2 | Allelic Imbalance | 26960 | 42.86 | 6.67 | 36.19 | 0.00 | 0.00 | LANCL2 |
| chr7:55,454,264-55,534,711 | p11.2 | Allelic Imbalance | 80447 | 42.86 | 6.67 | 36.19 | 0.00 | 0.00 | LANCL2, VOPP1 |
| chr7:86,989,374-87,007,465 | q21.12 | Allelic Imbalance | 18091 | 42.86 | 6.67 | 36.19 | 0.00 | 0.00 | ABCB1 |
| chr10:58,453,012-58,590,125 | q21.1 | Allelic Imbalance | 137113 | 33.33 | 2.22 | 31.11 | 0.00 | 0.00 |  |
| chr10:58,999,516-59,025,460 | q21.1 | Allelic Imbalance | 25944 | 33.33 | 2.22 | 31.11 | 0.00 | 0.00 |  |
| chr10:59,396,568-59,716,198 | q21.1 | Allelic Imbalance | 319630 | 38.10 | 4.44 | 33.65 | 0.00 | 0.00 | IPMK, CISD1 |
| chr10:60,384,659-60,531,514 | q21.1 | Allelic Imbalance | 146855 | 38.10 | 4.44 | 33.65 | 0.00 | 0.00 |  |
| chr10:62,869,953-63,142,274 | q21.2 | Allelic Imbalance | 272321 | 38.10 | 4.44 | 33.65 | 0.00 | 0.00 | TMEM26, C10orf107 |
| chr10:63,177,218-63,571,471 | q21.2 | Allelic Imbalance | 394253 | 38.10 | 4.44 | 33.65 | 0.00 | 0.00 | C10orf107, MIR548AV, ARID5B |
| chr7:139,888,653-139,900,303 | q34 | Allelic Imbalance | 11650 | 50.00 | 11.11 | 38.89 | 0.00 | 0.00 | DENND2A |
| chr7:10,608,887-10,700,376 | p21.3 | Allelic Imbalance | 91489 | 57.14 | 17.78 | 39.37 | 0.00 | 0.00 |  |
| chr7:103,465,325-103,473,824 | q22.1 | Allelic Imbalance | 8499 | 57.14 | 17.78 | 39.37 | 0.00 | 0.00 |  |
| chr7:11,490,785-11,495,362 | p21.3 | Allelic Imbalance | 4577 | 57.14 | 17.78 | 39.37 | 0.00 | 0.00 | THSD7A |
| chr7:11,586,087-11,587,221 | p21.3 | Allelic Imbalance | 1134 | 57.14 | 17.78 | 39.37 | 0.00 | 0.00 | THSD7A |
| chr7:11,855,660-12,011,388 | p21.3 | Allelic Imbalance | 155728 | 57.14 | 17.78 | 39.37 | 0.00 | 0.00 |  |
| chr7:12,016,357-12,081,420 | p21.3 | Allelic Imbalance | 65063 | 57.14 | 17.78 | 39.37 | 0.00 | 0.00 |  |
| chr7:139,871,356-139,875,465 | q34 | Allelic Imbalance | 4109 | 57.14 | 17.78 | 39.37 | 0.00 | 0.00 | DENND2A |
| chr7:14,253,868-14,338,871 | p21.2 | Allelic Imbalance | 85003 | 57.14 | 17.78 | 39.37 | 0.00 | 0.00 | DGKB |
| chr7:14,555,842-14,592,966 | p21.2 | Allelic Imbalance | 37124 | 57.14 | 17.78 | 39.37 | 0.00 | 0.00 | DGKB |
| chr7:27,170,310-27,210,694 | p15.2 | Allelic Imbalance | 40384 | 57.14 | 17.78 | 39.37 | 0.00 | 0.00 | HOXA9, MIR196B, HOXA-AS4, HOXA10-HOXA9, HOXA10, HOXA11, HOXA11-AS, HOXA13, HOTTIP |
| chr7:30,151,713-30,166,723 | p15.1 | Allelic Imbalance | 15010 | 57.14 | 17.78 | 39.37 | 0.00 | 0.00 | C7orf41 |
| chr7:30,204,321-30,238,804 | p15.1 | Allelic Imbalance | 34483 | 57.14 | 17.78 | 39.37 | 0.00 | 0.00 |  |
| chr7:44,756,597-44,818,865 | p13 | Allelic Imbalance | 62268 | 57.14 | 17.78 | 39.37 | 0.00 | 0.00 | ZMIZ2, PPIA |
| chr7:75,926,859-76,006,044 | q11.23 | Allelic Imbalance | 79185 | 57.14 | 17.78 | 39.37 | 0.00 | 0.00 | FDPSL2A, DTX2, UPK3B |
| chr7:14,399,392-14,452,912 | p21.2 | Allelic Imbalance | 53520 | 52.38 | 13.33 | 39.05 | 0.00 | 0.00 | DGKB |
| chr7:36,854,706-36,946,835 | p14.2 | Allelic Imbalance | 92129 | 52.38 | 13.33 | 39.05 | 0.00 | 0.00 | MIR1200, ELMO1 |
| chr7:36,957,467-37,117,378 | p14.2 | Allelic Imbalance | 159911 | 52.38 | 13.33 | 39.05 | 0.00 | 0.00 | ELMO1 |
| chr7:50,666,845-50,671,632 | p12.2 | Allelic Imbalance | 4787 | 52.38 | 13.33 | 39.05 | 0.00 | 0.00 | GRB10 |
| chr7:87,016,825-87,017,170 | q21.12 | Allelic Imbalance | 345 | 52.38 | 13.33 | 39.05 | 0.00 | 0.00 | ABCB1 |
| chr10:54,190,555-54,309,563 | q21.1 | Allelic Imbalance | 119008 | 45.24 | 8.89 | 36.35 | 0.00 | 0.00 | MBL2 |
| chr10:55,595,127-55,621,439 | q21.1 | Allelic Imbalance | 26312 | 45.24 | 8.89 | 36.35 | 0.00 | 0.00 | PCDH15 |
| chr10:61,500,236-61,510,999 | q21.2 | Allelic Imbalance | 10763 | 45.24 | 8.89 | 36.35 | 0.00 | 0.00 | ANK3 |
| chr10:89,589,445-89,591,425 | q23.2 | Allelic Imbalance | 1980 | 45.24 | 8.89 | 36.35 | 0.00 | 0.00 | CFL1P1 |
| chr7:55,354,196-55,368,981 | p11.2 | Allelic Imbalance | 14785 | 45.24 | 8.89 | 36.35 | 0.00 | 0.00 |  |
| chr7:107,778,790-107,790,223 | q31.1 | Allelic Imbalance | 11433 | 59.52 | 20.00 | 39.52 | 0.00 | 0.01 | NRCAM |
| chr7:12,484,016-12,624,995 | p21.3 | Allelic Imbalance | 140979 | 59.52 | 20.00 | 39.52 | 0.00 | 0.01 | SCIN |
| chr7:23,092,388-23,185,379 | p15.3 | Allelic Imbalance | 92991 | 59.52 | 20.00 | 39.52 | 0.00 | 0.01 | KLHL7-AS1, KLHL7 |
| chr7:68,271,070-68,324,994 | q11.22 | Allelic Imbalance | 53924 | 59.52 | 20.00 | 39.52 | 0.00 | 0.01 |  |
| chr7:75,160,600-75,212,324 | q11.23 | Allelic Imbalance | 51724 | 59.52 | 20.00 | 39.52 | 0.00 | 0.01 | HIP1 |
| chr7:75,237,031-75,258,480 | q11.23 | Allelic Imbalance | 21449 | 59.52 | 20.00 | 39.52 | 0.00 | 0.01 | CCL26 |
| chr7:76,269,702-76,310,444 | q11.23 | Allelic Imbalance | 40742 | 59.52 | 20.00 | 39.52 | 0.00 | 0.01 |  |
| chr7:76,592,220-76,632,268 | q11.23 | Allelic Imbalance | 40048 | 59.52 | 20.00 | 39.52 | 0.00 | 0.01 | CCDC146 |
| chr7:76,833,867-76,871,105 | q11.23 | Allelic Imbalance | 37238 | 59.52 | 20.00 | 39.52 | 0.00 | 0.01 | PION |
| chr7:93,894,066-93,896,390 | q21.3 | Allelic Imbalance | 2324 | 59.52 | 20.00 | 39.52 | 0.00 | 0.01 | COL1A2 |
| chr7:110,629,251-110,850,513 | q31.1 | Allelic Imbalance | 221262 | 61.90 | 22.22 | 39.68 | 0.00 | 0.01 | IMMP2L |
| chr7:112,498,642-112,503,253 | q31.1 | Allelic Imbalance | 4611 | 61.90 | 22.22 | 39.68 | 0.00 | 0.01 |  |
| chr7:118,550,175-118,586,835 | q31.31 | Allelic Imbalance | 36660 | 61.90 | 22.22 | 39.68 | 0.00 | 0.01 |  |
| chr7:119,206,369-119,410,902 | q31.31 | Allelic Imbalance | 204533 | 61.90 | 22.22 | 39.68 | 0.00 | 0.01 |  |
| chr7:119,595,870-119,902,067 | q31.31 | Allelic Imbalance | 306197 | 61.90 | 22.22 | 39.68 | 0.00 | 0.01 | KCND2 |
| chr7:120,046,994-120,136,066 | q31.31 | Allelic Imbalance | 89072 | 61.90 | 22.22 | 39.68 | 0.00 | 0.01 | KCND2 |
| chr7:139,104,971-139,108,674 | q34 | Allelic Imbalance | 3703 | 61.90 | 22.22 | 39.68 | 0.00 | 0.01 | HIPK2 |
| chr7:28,538,065-28,612,305 | p15.1 | Allelic Imbalance | 74240 | 61.90 | 22.22 | 39.68 | 0.00 | 0.01 | CREB5 |
| chr7:69,661,863-69,707,735 | q11.22 | Allelic Imbalance | 45872 | 61.90 | 22.22 | 39.68 | 0.00 | 0.01 | AUTS2 |
| chr7:69,867,807-70,164,529 | q11.22 | Allelic Imbalance | 296722 | 61.90 | 22.22 | 39.68 | 0.00 | 0.01 | AUTS2 |
| chr7:71,789,465-72,000,166 | q11.22 - q11.23 | Allelic Imbalance | 210701 | 61.90 | 22.22 | 39.68 | 0.00 | 0.01 | TYW1B, MIR4650-1, MIR4650-2, SBDSP1, SPDYE7P, POM121 |
| chr7:72,826,461-72,890,453 | q11.23 | Allelic Imbalance | 63992 | 61.90 | 22.22 | 39.68 | 0.00 | 0.01 | CLDN4, WBSCR27 |
| chr7:75,007,035-75,086,498 | q11.23 | Allelic Imbalance | 79463 | 61.90 | 22.22 | 39.68 | 0.00 | 0.01 | HIP1 |
| chr7:79,145,372-79,169,814 | q21.11 | Allelic Imbalance | 24442 | 61.90 | 22.22 | 39.68 | 0.00 | 0.01 |  |
| chr7:79,667,002-79,705,358 | q21.11 | Allelic Imbalance | 38356 | 61.90 | 22.22 | 39.68 | 0.00 | 0.01 | GNAI1 |
| chr7:80,273,682-80,329,170 | q21.11 | Allelic Imbalance | 55488 | 61.90 | 22.22 | 39.68 | 0.00 | 0.01 | SEMA3C |
| chr7:107,801,171-107,810,976 | q31.1 | Allelic Imbalance | 9805 | 64.29 | 24.44 | 39.84 | 0.00 | 0.01 | NRCAM |
| chr7:110,223,455-110,262,348 | q31.1 | Allelic Imbalance | 38893 | 64.29 | 24.44 | 39.84 | 0.00 | 0.01 | IMMP2L |
| chr7:112,504,151-112,512,556 | q31.1 | Allelic Imbalance | 8405 | 64.29 | 24.44 | 39.84 | 0.00 | 0.01 | GPR85 |
| chr7:112,522,392-112,730,896 | q31.1 | Allelic Imbalance | 208504 | 64.29 | 24.44 | 39.84 | 0.00 | 0.01 | LOC401397 |
| chr7:112,753,367-112,802,822 | q31.1 | Allelic Imbalance | 49455 | 64.29 | 24.44 | 39.84 | 0.00 | 0.01 |  |
| chr7:139,063,589-139,102,805 | q34 | Allelic Imbalance | 39216 | 64.29 | 24.44 | 39.84 | 0.00 | 0.01 | HIPK2 |
| chr7:139,240,301-139,259,463 | q34 | Allelic Imbalance | 19162 | 64.29 | 24.44 | 39.84 | 0.00 | 0.01 | TBXAS1 |
| chr7:139,267,231-139,279,153 | q34 | Allelic Imbalance | 11922 | 64.29 | 24.44 | 39.84 | 0.00 | 0.01 | TBXAS1 |
| chr7:68,066,204-68,146,692 | q11.22 | Allelic Imbalance | 80488 | 64.29 | 24.44 | 39.84 | 0.00 | 0.01 |  |
| chr7:70,590,464-70,663,106 | q11.22 | Allelic Imbalance | 72642 | 64.29 | 24.44 | 39.84 | 0.00 | 0.01 | WBSCR17 |
| chr7:71,031,973-71,045,831 | q11.22 | Allelic Imbalance | 13858 | 64.29 | 24.44 | 39.84 | 0.00 | 0.01 | CALN1 |
| chr7:71,079,378-71,134,418 | q11.22 | Allelic Imbalance | 55040 | 64.29 | 24.44 | 39.84 | 0.00 | 0.01 | CALN1 |
| chr7:71,434,554-71,484,591 | q11.22 | Allelic Imbalance | 50037 | 64.29 | 24.44 | 39.84 | 0.00 | 0.01 | CALN1 |
| chr7:71,611,578-71,641,261 | q11.22 | Allelic Imbalance | 29683 | 64.29 | 24.44 | 39.84 | 0.00 | 0.01 |  |
| chr7:77,450,864-77,485,597 | q21.11 | Allelic Imbalance | 34733 | 64.29 | 24.44 | 39.84 | 0.00 | 0.01 | MAGI2 |
| chr7:77,542,383-77,609,277 | q21.11 | Allelic Imbalance | 66894 | 64.29 | 24.44 | 39.84 | 0.00 | 0.01 | MAGI2 |
| chr7:78,352,166-78,371,776 | q21.11 | Allelic Imbalance | 19610 | 64.29 | 24.44 | 39.84 | 0.00 | 0.01 | MAGI2 |
| chr7:79,721,748-79,754,309 | q21.11 | Allelic Imbalance | 32561 | 64.29 | 24.44 | 39.84 | 0.00 | 0.01 |  |
| chr7:82,926,656-83,011,673 | q21.11 | Allelic Imbalance | 85017 | 64.29 | 24.44 | 39.84 | 0.00 | 0.01 | SEMA3E |
| chr10:55,177,986-55,307,693 | q21.1 | Allelic Imbalance | 129707 | 40.48 | 6.67 | 33.81 | 0.00 | 0.01 | PCDH15 |
| chr10:55,693,823-55,872,960 | q21.1 | Allelic Imbalance | 179137 | 40.48 | 6.67 | 33.81 | 0.00 | 0.01 | PCDH15 |
| chr10:60,554,850-60,741,132 | q21.1 | Allelic Imbalance | 186282 | 40.48 | 6.67 | 33.81 | 0.00 | 0.01 | PHYHIPL, FAM13C |
| chr10:61,003,062-61,053,026 | q21.1 | Allelic Imbalance | 49964 | 40.48 | 6.67 | 33.81 | 0.00 | 0.01 |  |
| chr10:61,198,238-61,260,776 | q21.1 - q21.2 | Allelic Imbalance | 62538 | 40.48 | 6.67 | 33.81 | 0.00 | 0.01 | CCDC6 |
| chr10:61,462,423-61,468,789 | q21.2 | Allelic Imbalance | 6366 | 40.48 | 6.67 | 33.81 | 0.00 | 0.01 | ANK3 |
| chr7:139,875,465-139,888,653 | q34 | Allelic Imbalance | 13188 | 54.76 | 15.56 | 39.21 | 0.00 | 0.01 | DENND2A |
| chr7:139,980,098-140,065,381 | q34 | Allelic Imbalance | 85283 | 40.48 | 6.67 | 33.81 | 0.00 | 0.01 | ADCK2, NDUFB2-AS1, NDUFB2 |
| chr7:14,343,016-14,392,750 | p21.2 | Allelic Imbalance | 49734 | 54.76 | 15.56 | 39.21 | 0.00 | 0.01 | DGKB |
| chr7:14,603,713-14,646,648 | p21.2 | Allelic Imbalance | 42935 | 54.76 | 15.56 | 39.21 | 0.00 | 0.01 | DGKB |
| chr7:17,694,541-17,695,624 | p21.1 | Allelic Imbalance | 1083 | 54.76 | 15.56 | 39.21 | 0.00 | 0.01 |  |
| chr7:27,443,901-27,453,036 | p15.2 | Allelic Imbalance | 9135 | 54.76 | 15.56 | 39.21 | 0.00 | 0.01 |  |
| chr7:30,415,251-30,464,604 | p15.1 | Allelic Imbalance | 49353 | 54.76 | 15.56 | 39.21 | 0.00 | 0.01 | NOD1 |
| chr7:32,004,562-32,006,858 | p14.3 | Allelic Imbalance | 2296 | 54.76 | 15.56 | 39.21 | 0.00 | 0.01 | PDE1C |
| chr7:39,896,083-40,338,391 | p14.1 | Allelic Imbalance | 442308 | 54.76 | 15.56 | 39.21 | 0.00 | 0.01 | CDK13, MPLKIP, C7orf10 |
| chr7:44,604,117-44,663,873 | p13 | Allelic Imbalance | 59756 | 54.76 | 15.56 | 39.21 | 0.00 | 0.01 | OGDH |
| chr7:55,123,739-55,129,372 | p11.2 | Allelic Imbalance | 5633 | 40.48 | 6.67 | 33.81 | 0.00 | 0.01 | EGFR |
| chr7:55,405,856-55,454,264 | p11.2 | Allelic Imbalance | 48408 | 40.48 | 6.67 | 33.81 | 0.00 | 0.01 | LANCL2 |
| chr7:75,814,733-75,863,445 | q11.23 | Allelic Imbalance | 48712 | 54.76 | 15.56 | 39.21 | 0.00 | 0.01 | YWHAG, SRCRB4D |
| chr7:110,269,660-110,270,451 | q31.1 | Allelic Imbalance | 791 | 66.67 | 26.67 | 40.00 | 0.00 | 0.01 | IMMP2L |
| chr7:138,989,098-139,006,498 | q34 | Allelic Imbalance | 17400 | 71.43 | 31.11 | 40.32 | 0.00 | 0.01 | HIPK2 |
| chr7:139,280,650-139,317,236 | q34 | Allelic Imbalance | 36586 | 66.67 | 26.67 | 40.00 | 0.00 | 0.01 | TBXAS1 |
| chr7:146,859,298-147,042,077 | q35 | Allelic Imbalance | 182779 | 71.43 | 31.11 | 40.32 | 0.00 | 0.01 | CNTNAP2 |
| chr7:147,992,669-148,129,191 | q36.1 | Allelic Imbalance | 136522 | 66.67 | 26.67 | 40.00 | 0.00 | 0.01 | CUL1 |
| chr7:148,303,811-148,396,034 | q36.1 | Allelic Imbalance | 92223 | 66.67 | 26.67 | 40.00 | 0.00 | 0.01 | PDIA4 |
| chr7:150,063,512-150,073,353 | q36.1 | Allelic Imbalance | 9841 | 66.67 | 26.67 | 40.00 | 0.00 | 0.01 | GIMAP1-GIMAP5, GIMAP5 |
| chr7:151,263,015-151,313,355 | q36.1 | Allelic Imbalance | 50340 | 71.43 | 31.11 | 40.32 | 0.00 | 0.01 | GALNTL5 |
| chr7:154,276,703-154,279,934 | q36.2 | Allelic Imbalance | 3231 | 66.67 | 26.67 | 40.00 | 0.00 | 0.01 | DPP6 |
| chr7:155,398,994-155,438,978 | q36.3 | Allelic Imbalance | 39984 | 71.43 | 31.11 | 40.32 | 0.00 | 0.01 |  |
| chr7:155,718,090-155,761,154 | q36.3 | Allelic Imbalance | 43064 | 71.43 | 31.11 | 40.32 | 0.00 | 0.01 |  |
| chr7:78,338,911-78,342,384 | q21.11 | Allelic Imbalance | 3473 | 66.67 | 26.67 | 40.00 | 0.00 | 0.01 | MAGI2 |
| chr7:81,707,927-81,799,137 | q21.11 | Allelic Imbalance | 91210 | 66.67 | 26.67 | 40.00 | 0.00 | 0.01 | CACNA2D1 |
| chr7:81,803,500-81,824,444 | q21.11 | Allelic Imbalance | 20944 | 66.67 | 26.67 | 40.00 | 0.00 | 0.01 | CACNA2D1 |
| chr7:81,943,891-82,016,375 | q21.11 | Allelic Imbalance | 72484 | 66.67 | 26.67 | 40.00 | 0.00 | 0.01 |  |
| chr7:82,400,356-82,572,283 | q21.11 | Allelic Imbalance | 171927 | 66.67 | 26.67 | 40.00 | 0.00 | 0.01 | PCLO |
| chr7:139,352,208-139,365,975 | q34 | Allelic Imbalance | 13767 | 69.05 | 28.89 | 40.16 | 0.00 | 0.01 | TBXAS1 |
| chr7:139,387,224-139,709,945 | q34 | Allelic Imbalance | 322721 | 69.05 | 28.89 | 40.16 | 0.00 | 0.01 | PARP12, JHDM1D, LOC100134229, SLC37A3 |
| chr7:139,780,488-139,793,959 | q34 | Allelic Imbalance | 13471 | 69.05 | 28.89 | 40.16 | 0.00 | 0.01 |  |
| chr7:148,421,975-148,567,026 | q36.1 | Allelic Imbalance | 145051 | 69.05 | 28.89 | 40.16 | 0.00 | 0.01 | ZNF425, ZNF398, ZNF282 |
| chr7:150,230,186-150,330,339 | q36.1 | Allelic Imbalance | 100153 | 69.05 | 28.89 | 40.16 | 0.00 | 0.01 | KCNH2, NOS3 |
| chr7:151,326,668-151,419,516 | q36.1 | Allelic Imbalance | 92848 | 69.05 | 28.89 | 40.16 | 0.00 | 0.01 | GALNTL5, GALNT11 |
| chr7:78,179,402-78,310,757 | q21.11 | Allelic Imbalance | 131355 | 69.05 | 28.89 | 40.16 | 0.00 | 0.01 | MAGI2 |
| chr7:81,842,467-81,849,015 | q21.11 | Allelic Imbalance | 6548 | 69.05 | 28.89 | 40.16 | 0.00 | 0.01 | CACNA2D1 |
| chr7:82,061,107-82,297,407 | q21.11 | Allelic Imbalance | 236300 | 69.05 | 28.89 | 40.16 | 0.00 | 0.01 | PCLO |
| chr7:93,287,872-93,300,316 | q21.3 | Allelic Imbalance | 12444 | 69.05 | 28.89 | 40.16 | 0.00 | 0.01 |  |
| chr7:93,403,117-93,411,986 | q21.3 | Allelic Imbalance | 8869 | 69.05 | 28.89 | 40.16 | 0.00 | 0.01 |  |
| chr10:58,590,125-58,999,516 | q21.1 | Allelic Imbalance | 409391 | 30.95 | 2.22 | 28.73 | 0.00 | 0.01 | MIR3924 |
| chr10:59,132,718-59,396,568 | q21.1 | Allelic Imbalance | 263850 | 35.71 | 4.44 | 31.27 | 0.00 | 0.01 |  |
| chr10:102,982,234-103,175,985 | q24.31 - q24.32 | Allelic Imbalance | 193751 | 47.62 | 11.11 | 36.51 | 0.00 | 0.01 | FLJ41350, BTRC |
| chr10:42,906,746-42,908,150 | q11.21 | Allelic Imbalance | 1404 | 47.62 | 11.11 | 36.51 | 0.00 | 0.01 | RET |
| chr10:54,964,923-54,976,332 | q21.1 | Allelic Imbalance | 11409 | 47.62 | 11.11 | 36.51 | 0.00 | 0.01 |  |
| chr7:86,971,077-86,971,354 | q21.12 | Allelic Imbalance | 277 | 47.62 | 11.11 | 36.51 | 0.00 | 0.01 | ABCB1 |
| chr7:10,700,376-10,707,069 | p21.3 | Allelic Imbalance | 6693 | 54.76 | 17.78 | 36.98 | 0.00 | 0.01 |  |
| chr7:12,011,388-12,016,357 | p21.3 | Allelic Imbalance | 4969 | 54.76 | 17.78 | 36.98 | 0.00 | 0.01 |  |
| chr7:14,547,478-14,555,842 | p21.2 | Allelic Imbalance | 8364 | 54.76 | 17.78 | 36.98 | 0.00 | 0.01 | DGKB |
| chr7:14,592,966-14,603,713 | p21.2 | Allelic Imbalance | 10747 | 54.76 | 17.78 | 36.98 | 0.00 | 0.01 | DGKB |
| chr7:30,166,723-30,204,321 | p15.1 | Allelic Imbalance | 37598 | 54.76 | 17.78 | 36.98 | 0.00 | 0.01 | C7orf41 |
| chr7:44,663,873-44,756,597 | p13 | Allelic Imbalance | 92724 | 54.76 | 17.78 | 36.98 | 0.00 | 0.01 | OGDH, ZMIZ2 |
| chr7:94,676,864-94,739,051 | q21.3 | Allelic Imbalance | 62187 | 54.76 | 17.78 | 36.98 | 0.00 | 0.01 | PPP1R9A |
| chr7:94,741,472-94,745,737 | q21.3 | Allelic Imbalance | 4265 | 54.76 | 17.78 | 36.98 | 0.00 | 0.01 | PPP1R9A |
| chr8:128,475,694-128,554,995 | q24.21 | Allelic Imbalance | 79301 | 2.38 | 31.11 | -28.73 | 0.00 | 0.01 | POU5F1B, LOC727677 |
| chr10:89,572,968-89,573,109 | q23.2 | Allelic Imbalance | 141 | 50.00 | 13.33 | 36.67 | 0.00 | 0.01 | CFL1P1 |
| chr7:36,946,835-36,957,467 | p14.2 | Allelic Imbalance | 10632 | 50.00 | 13.33 | 36.67 | 0.00 | 0.01 | ELMO1 |
| chr7:86,930,000-86,930,652 | q21.12 | Allelic Imbalance | 652 | 50.00 | 13.33 | 36.67 | 0.00 | 0.01 | ABCB4 |
| chr7:87,017,170-87,028,004 | q21.12 | Allelic Imbalance | 10834 | 50.00 | 13.33 | 36.67 | 0.00 | 0.01 | ABCB1 |
| chr10:54,075,410-54,190,555 | q21.1 | Allelic Imbalance | 115145 | 42.86 | 8.89 | 33.97 | 0.00 | 0.01 |  |
| chr10:55,404,303-55,468,953 | q21.1 | Allelic Imbalance | 64650 | 42.86 | 8.89 | 33.97 | 0.00 | 0.01 | PCDH15 |
| chr10:61,057,077-61,144,083 | q21.1 | Allelic Imbalance | 87006 | 42.86 | 8.89 | 33.97 | 0.00 | 0.01 | SLC16A9 |
| chr10:61,307,876-61,365,680 | q21.2 | Allelic Imbalance | 57804 | 42.86 | 8.89 | 33.97 | 0.00 | 0.01 | CCDC6 |
| chr10:61,473,643-61,500,236 | q21.2 | Allelic Imbalance | 26593 | 42.86 | 8.89 | 33.97 | 0.00 | 0.01 | ANK3 |
| chr10:89,591,425-89,604,361 | q23.2 - q23.31 | Allelic Imbalance | 12936 | 42.86 | 8.89 | 33.97 | 0.00 | 0.01 | CFL1P1 |
| chr7:139,928,889-139,980,098 | q34 | Allelic Imbalance | 51209 | 42.86 | 8.89 | 33.97 | 0.00 | 0.01 | DENND2A |
| chr7:87,007,465-87,008,363 | q21.12 | Allelic Imbalance | 898 | 42.86 | 8.89 | 33.97 | 0.00 | 0.01 | ABCB1 |
| chr8:133,110,494-133,115,806 | q24.22 | Allelic Imbalance | 5312 | 4.76 | 35.56 | -30.79 | 0.00 | 0.01 | OC90 |
| chr7:103,473,824-103,503,438 | q22.1 | Allelic Imbalance | 29614 | 57.14 | 20.00 | 37.14 | 0.00 | 0.01 |  |
| chr7:11,495,362-11,513,420 | p21.3 | Allelic Imbalance | 18058 | 57.14 | 20.00 | 37.14 | 0.00 | 0.01 | THSD7A |
| chr7:139,851,179-139,871,356 | q34 | Allelic Imbalance | 20177 | 57.14 | 20.00 | 37.14 | 0.00 | 0.01 | DENND2A |
| chr7:68,324,994-68,357,393 | q11.22 | Allelic Imbalance | 32399 | 57.14 | 20.00 | 37.14 | 0.00 | 0.01 |  |
| chr7:69,568,964-69,587,511 | q11.22 | Allelic Imbalance | 18547 | 57.14 | 20.00 | 37.14 | 0.00 | 0.01 | AUTS2 |
| chr7:75,258,480-75,280,376 | q11.23 | Allelic Imbalance | 21896 | 57.14 | 20.00 | 37.14 | 0.00 | 0.01 | CCL24 |
| chr7:76,006,044-76,269,702 | q11.23 | Allelic Imbalance | 263658 | 57.14 | 20.00 | 37.14 | 0.00 | 0.01 | LOC100133091, POMZP3 |
| chr7:76,632,268-76,723,790 | q11.23 | Allelic Imbalance | 91522 | 57.14 | 20.00 | 37.14 | 0.00 | 0.01 | FGL2, CCDC146 |
| chr7:76,767,781-76,833,867 | q11.23 | Allelic Imbalance | 66086 | 57.14 | 20.00 | 37.14 | 0.00 | 0.01 | PION |
| chr7:76,871,105-76,969,577 | q11.23 | Allelic Imbalance | 98472 | 57.14 | 20.00 | 37.14 | 0.00 | 0.01 | PION |
| chr7:78,928,245-79,126,757 | q21.11 | Allelic Imbalance | 198512 | 57.14 | 20.00 | 37.14 | 0.00 | 0.01 | MAGI2-AS3 |
| chr7:103,362,053-103,368,272 | q22.1 | Allelic Imbalance | 6219 | 59.52 | 22.22 | 37.30 | 0.00 | 0.01 | RELN |
| chr7:107,734,426-107,778,790 | q31.1 | Allelic Imbalance | 44364 | 59.52 | 22.22 | 37.30 | 0.00 | 0.01 | NRCAM |
| chr7:107,790,223-107,795,686 | q31.1 | Allelic Imbalance | 5463 | 59.52 | 22.22 | 37.30 | 0.00 | 0.01 | NRCAM |
| chr7:110,477,882-110,614,851 | q31.1 | Allelic Imbalance | 136969 | 59.52 | 22.22 | 37.30 | 0.00 | 0.01 | LRRN3, IMMP2L |
| chr7:112,393,863-112,498,642 | q31.1 | Allelic Imbalance | 104779 | 59.52 | 22.22 | 37.30 | 0.00 | 0.01 |  |
| chr7:114,171,357-114,204,088 | q31.1 | Allelic Imbalance | 32731 | 59.52 | 22.22 | 37.30 | 0.00 | 0.01 |  |
| chr7:118,586,835-118,947,971 | q31.31 | Allelic Imbalance | 361136 | 59.52 | 22.22 | 37.30 | 0.00 | 0.01 |  |
| chr7:118,964,411-119,206,369 | q31.31 | Allelic Imbalance | 241958 | 59.52 | 22.22 | 37.30 | 0.00 | 0.01 |  |
| chr7:119,410,902-119,595,870 | q31.31 | Allelic Imbalance | 184968 | 59.52 | 22.22 | 37.30 | 0.00 | 0.01 |  |
| chr7:119,902,067-119,957,583 | q31.31 | Allelic Imbalance | 55516 | 59.52 | 22.22 | 37.30 | 0.00 | 0.01 | KCND2 |
| chr7:119,987,820-120,046,994 | q31.31 | Allelic Imbalance | 59174 | 59.52 | 22.22 | 37.30 | 0.00 | 0.01 | KCND2 |
| chr7:120,136,066-120,286,614 | q31.31 | Allelic Imbalance | 150548 | 59.52 | 22.22 | 37.30 | 0.00 | 0.01 | KCND2, TSPAN12 |
| chr7:139,108,674-139,112,494 | q34 | Allelic Imbalance | 3820 | 59.52 | 22.22 | 37.30 | 0.00 | 0.01 | HIPK2 |
| chr7:69,587,511-69,635,374 | q11.22 | Allelic Imbalance | 47863 | 59.52 | 22.22 | 37.30 | 0.00 | 0.01 | AUTS2 |
| chr7:72,000,166-72,652,178 | q11.23 | Allelic Imbalance | 652012 | 59.52 | 22.22 | 37.30 | 0.00 | 0.01 | POM121, NSUN5P2, TRIM74, STAG3L3, SPDYE8P, PMS2L2, LOC100093631, GTF2IP1, NCF1B, GTF2IRD2P1, NSUN5, TRIM50, FKBP6, FZD9, BAZ1B, BCL7B, TBL2, MLXIPL |
| chr7:72,802,975-72,826,461 | q11.23 | Allelic Imbalance | 23486 | 59.52 | 22.22 | 37.30 | 0.00 | 0.01 | CLDN3 |
| chr7:77,280,466-77,442,001 | q11.23 - q21.11 | Allelic Imbalance | 161535 | 59.52 | 22.22 | 37.30 | 0.00 | 0.01 | PHTF2 |
| chr7:79,127,529-79,145,372 | q21.11 | Allelic Imbalance | 17843 | 59.52 | 22.22 | 37.30 | 0.00 | 0.01 |  |
| chr7:79,635,580-79,667,002 | q21.11 | Allelic Imbalance | 31422 | 59.52 | 22.22 | 37.30 | 0.00 | 0.01 | GNAI1 |
| chr7:83,440,259-83,483,821 | q21.11 | Allelic Imbalance | 43562 | 59.52 | 22.22 | 37.30 | 0.00 | 0.01 | SEMA3A |
| chr7:84,811,995-84,858,497 | q21.11 | Allelic Imbalance | 46502 | 59.52 | 22.22 | 37.30 | 0.00 | 0.01 |  |
| chr7:87,965,478-87,989,500 | q21.12 | Allelic Imbalance | 24022 | 59.52 | 22.22 | 37.30 | 0.00 | 0.01 |  |
| chr7:93,892,307-93,894,066 | q21.3 | Allelic Imbalance | 1759 | 59.52 | 22.22 | 37.30 | 0.00 | 0.01 | COL1A2 |
| chr10:111,962,358-111,994,113 | q25.2 | Allelic Imbalance | 31755 | 38.10 | 6.67 | 31.43 | 0.00 | 0.01 | MXI1 |
| chr10:112,026,531-112,030,002 | q25.2 | Allelic Imbalance | 3471 | 38.10 | 6.67 | 31.43 | 0.00 | 0.01 | MXI1 |
| chr10:55,165,861-55,177,986 | q21.1 | Allelic Imbalance | 12125 | 38.10 | 6.67 | 31.43 | 0.00 | 0.01 |  |
| chr10:60,741,132-61,003,062 | q21.1 | Allelic Imbalance | 261930 | 38.10 | 6.67 | 31.43 | 0.00 | 0.01 | FAM13C |
| chr10:62,826,768-62,869,953 | q21.2 | Allelic Imbalance | 43185 | 38.10 | 6.67 | 31.43 | 0.00 | 0.01 | TMEM26 |
| chr10:63,571,471-63,653,874 | q21.2 | Allelic Imbalance | 82403 | 38.10 | 6.67 | 31.43 | 0.00 | 0.01 | RTKN2 |
| chr7:103,368,272-103,368,952 | q22.1 | Allelic Imbalance | 680 | 61.90 | 24.44 | 37.46 | 0.00 | 0.01 | RELN |
| chr7:107,185,501-107,189,334 | q22.3 | Allelic Imbalance | 3833 | 61.90 | 24.44 | 37.46 | 0.00 | 0.01 | CBLL1 |
| chr7:107,795,686-107,801,171 | q31.1 | Allelic Imbalance | 5485 | 61.90 | 24.44 | 37.46 | 0.00 | 0.01 | NRCAM |
| chr7:107,810,976-107,811,759 | q31.1 | Allelic Imbalance | 783 | 61.90 | 24.44 | 37.46 | 0.00 | 0.01 | NRCAM |
| chr7:110,292,691-110,302,970 | q31.1 | Allelic Imbalance | 10279 | 61.90 | 24.44 | 37.46 | 0.00 | 0.01 | IMMP2L |
| chr7:110,850,513-110,992,580 | q31.1 | Allelic Imbalance | 142067 | 61.90 | 24.44 | 37.46 | 0.00 | 0.01 | IMMP2L |
| chr7:112,513,885-112,522,392 | q31.1 | Allelic Imbalance | 8507 | 61.90 | 24.44 | 37.46 | 0.00 | 0.01 | GPR85 |
| chr7:112,730,896-112,753,367 | q31.1 | Allelic Imbalance | 22471 | 61.90 | 24.44 | 37.46 | 0.00 | 0.01 |  |
| chr7:118,413,994-118,550,175 | q31.31 | Allelic Imbalance | 136181 | 61.90 | 24.44 | 37.46 | 0.00 | 0.01 |  |
| chr7:14,392,750-14,399,392 | p21.2 | Allelic Imbalance | 6642 | 52.38 | 15.56 | 36.83 | 0.00 | 0.01 | DGKB |
| chr7:14,452,912-14,547,478 | p21.2 | Allelic Imbalance | 94566 | 52.38 | 15.56 | 36.83 | 0.00 | 0.01 | DGKB |
| chr7:140,065,381-140,157,207 | q34 | Allelic Imbalance | 91826 | 38.10 | 6.67 | 31.43 | 0.00 | 0.01 | BRAF |
| chr7:30,464,604-30,525,989 | p15.1 | Allelic Imbalance | 61385 | 52.38 | 15.56 | 36.83 | 0.00 | 0.01 | NOD1, GGCT |
| chr7:30,698,848-30,712,192 | p15.1 | Allelic Imbalance | 13344 | 52.38 | 15.56 | 36.83 | 0.00 | 0.01 | CRHR2 |
| chr7:31,788,707-31,815,579 | p15.1 - p14.3 | Allelic Imbalance | 26872 | 61.90 | 24.44 | 37.46 | 0.00 | 0.01 | PDE1C |
| chr7:32,003,820-32,004,562 | p14.3 | Allelic Imbalance | 742 | 52.38 | 15.56 | 36.83 | 0.00 | 0.01 | PDE1C |
| chr7:44,538,490-44,604,117 | p13 | Allelic Imbalance | 65627 | 52.38 | 15.56 | 36.83 | 0.00 | 0.01 | NPC1L1, DDX56, TMED4 |
| chr7:68,357,393-68,406,645 | q11.22 | Allelic Imbalance | 49252 | 52.38 | 15.56 | 36.83 | 0.00 | 0.01 |  |
| chr7:71,069,600-71,079,378 | q11.22 | Allelic Imbalance | 9778 | 61.90 | 24.44 | 37.46 | 0.00 | 0.01 | CALN1 |
| chr7:71,484,591-71,611,578 | q11.22 | Allelic Imbalance | 126987 | 61.90 | 24.44 | 37.46 | 0.00 | 0.01 | CALN1 |
| chr7:75,784,078-75,814,733 | q11.23 | Allelic Imbalance | 30655 | 52.38 | 15.56 | 36.83 | 0.00 | 0.01 | YWHAG |
| chr7:77,442,001-77,450,864 | q21.11 | Allelic Imbalance | 8863 | 61.90 | 24.44 | 37.46 | 0.00 | 0.01 |  |
| chr7:77,485,597-77,542,383 | q21.11 | Allelic Imbalance | 56786 | 61.90 | 24.44 | 37.46 | 0.00 | 0.01 | MAGI2 |
| chr7:79,169,814-79,196,647 | q21.11 | Allelic Imbalance | 26833 | 61.90 | 24.44 | 37.46 | 0.00 | 0.01 |  |
| chr7:80,329,170-80,394,648 | q21.11 | Allelic Imbalance | 65478 | 61.90 | 24.44 | 37.46 | 0.00 | 0.01 | SEMA3C |
| chr7:81,375,889-81,409,895 | q21.11 | Allelic Imbalance | 34006 | 61.90 | 24.44 | 37.46 | 0.00 | 0.01 |  |
| chr7:83,377,069-83,419,593 | q21.11 | Allelic Imbalance | 42524 | 61.90 | 24.44 | 37.46 | 0.00 | 0.01 |  |
| chr7:88,150,427-88,151,557 | q21.13 | Allelic Imbalance | 1130 | 38.10 | 6.67 | 31.43 | 0.00 | 0.01 |  |
| chr7:88,216,552-88,235,285 | q21.13 | Allelic Imbalance | 18733 | 38.10 | 6.67 | 31.43 | 0.00 | 0.01 | ZNF804B |
| chr7:93,868,376-93,869,114 | q21.3 | Allelic Imbalance | 738 | 61.90 | 24.44 | 37.46 | 0.00 | 0.01 | COL1A2 |
| chr7:93,884,799-93,885,702 | q21.3 | Allelic Imbalance | 903 | 61.90 | 24.44 | 37.46 | 0.00 | 0.01 | COL1A2 |
| chr7:93,885,869-93,890,348 | q21.3 | Allelic Imbalance | 4479 | 61.90 | 24.44 | 37.46 | 0.00 | 0.01 | COL1A2 |
| chr7:94,527,575-94,584,928 | q21.3 | Allelic Imbalance | 57353 | 52.38 | 15.56 | 36.83 | 0.00 | 0.01 | PPP1R9A |
| chr7:103,368,952-103,369,461 | q22.1 | Allelic Imbalance | 509 | 64.29 | 26.67 | 37.62 | 0.00 | 0.01 | RELN |
| chr7:107,813,205-107,818,456 | q31.1 | Allelic Imbalance | 5251 | 64.29 | 26.67 | 37.62 | 0.00 | 0.01 | NRCAM |
| chr7:109,431,871-109,461,585 | q31.1 | Allelic Imbalance | 29714 | 66.67 | 28.89 | 37.78 | 0.00 | 0.01 |  |
| chr7:109,473,916-109,605,031 | q31.1 | Allelic Imbalance | 131115 | 66.67 | 28.89 | 37.78 | 0.00 | 0.01 |  |
| chr7:109,711,773-109,718,984 | q31.1 | Allelic Imbalance | 7211 | 66.67 | 28.89 | 37.78 | 0.00 | 0.01 |  |
| chr7:110,104,915-110,106,642 | q31.1 | Allelic Imbalance | 1727 | 64.29 | 26.67 | 37.62 | 0.00 | 0.01 | IMMP2L |
| chr7:110,187,389-110,223,455 | q31.1 | Allelic Imbalance | 36066 | 64.29 | 26.67 | 37.62 | 0.00 | 0.01 | IMMP2L |
| chr7:110,262,348-110,269,660 | q31.1 | Allelic Imbalance | 7312 | 64.29 | 26.67 | 37.62 | 0.00 | 0.01 | IMMP2L |
| chr7:110,270,451-110,270,992 | q31.1 | Allelic Imbalance | 541 | 64.29 | 26.67 | 37.62 | 0.00 | 0.01 | IMMP2L |
| chr7:112,802,822-112,827,631 | q31.1 | Allelic Imbalance | 24809 | 64.29 | 26.67 | 37.62 | 0.00 | 0.01 |  |
| chr7:126,737,836-126,842,316 | q31.33 | Allelic Imbalance | 104480 | 71.43 | 33.33 | 38.10 | 0.00 | 0.01 | ZNF800 |
| chr7:127,665,418-127,679,814 | q32.1 | Allelic Imbalance | 14396 | 71.43 | 33.33 | 38.10 | 0.00 | 0.01 | LEP |
| chr7:139,010,174-139,045,026 | q34 | Allelic Imbalance | 34852 | 66.67 | 28.89 | 37.78 | 0.00 | 0.01 | HIPK2 |
| chr7:139,047,853-139,063,589 | q34 | Allelic Imbalance | 15736 | 64.29 | 26.67 | 37.62 | 0.00 | 0.01 | HIPK2 |
| chr7:139,235,455-139,240,301 | q34 | Allelic Imbalance | 4846 | 64.29 | 26.67 | 37.62 | 0.00 | 0.01 | TBXAS1 |
| chr7:139,259,463-139,267,231 | q34 | Allelic Imbalance | 7768 | 64.29 | 26.67 | 37.62 | 0.00 | 0.01 | TBXAS1 |
| chr7:139,279,153-139,280,650 | q34 | Allelic Imbalance | 1497 | 64.29 | 26.67 | 37.62 | 0.00 | 0.01 | TBXAS1 |
| chr7:139,365,975-139,387,224 | q34 | Allelic Imbalance | 21249 | 66.67 | 28.89 | 37.78 | 0.00 | 0.01 | TBXAS1, PARP12 |
| chr7:146,595,871-146,859,298 | q35 | Allelic Imbalance | 263427 | 69.05 | 31.11 | 37.94 | 0.00 | 0.01 | CNTNAP2, MIR548F4 |
| chr7:147,042,077-147,131,428 | q35 | Allelic Imbalance | 89351 | 69.05 | 31.11 | 37.94 | 0.00 | 0.01 | CNTNAP2 |
| chr7:147,982,869-147,992,669 | q36.1 | Allelic Imbalance | 9800 | 66.67 | 28.89 | 37.78 | 0.00 | 0.01 |  |
| chr7:148,567,026-148,647,119 | q36.1 | Allelic Imbalance | 80093 | 69.05 | 31.11 | 37.94 | 0.00 | 0.01 | ZNF212, ZNF783, LOC155060 |
| chr7:149,983,809-150,003,095 | q36.1 | Allelic Imbalance | 19286 | 64.29 | 26.67 | 37.62 | 0.00 | 0.01 |  |
| chr7:150,017,433-150,063,512 | q36.1 | Allelic Imbalance | 46079 | 66.67 | 28.89 | 37.78 | 0.00 | 0.01 | GIMAP2, GIMAP1, GIMAP1-GIMAP5 |
| chr7:150,330,339-150,363,570 | q36.1 | Allelic Imbalance | 33231 | 66.67 | 28.89 | 37.78 | 0.00 | 0.01 | NOS3, ATG9B, ABCB8 |
| chr7:151,214,176-151,263,015 | q36.1 | Allelic Imbalance | 48839 | 69.05 | 31.11 | 37.94 | 0.00 | 0.01 |  |
| chr7:151,313,355-151,326,668 | q36.1 | Allelic Imbalance | 13313 | 69.05 | 31.11 | 37.94 | 0.00 | 0.01 | GALNTL5 |
| chr7:151,419,516-151,835,301 | q36.1 | Allelic Imbalance | 415785 | 69.05 | 31.11 | 37.94 | 0.00 | 0.01 | GALNT11, MLL3, FABP5P3, LOC100128822 |
| chr7:152,001,822-152,011,733 | q36.1 | Allelic Imbalance | 9911 | 69.05 | 31.11 | 37.94 | 0.00 | 0.01 | XRCC2 |
| chr7:152,313,363-152,608,826 | q36.2 | Allelic Imbalance | 295463 | 71.43 | 33.33 | 38.10 | 0.00 | 0.01 |  |
| chr7:152,712,136-152,847,409 | q36.2 | Allelic Imbalance | 135273 | 69.05 | 31.11 | 37.94 | 0.00 | 0.01 |  |
| chr7:153,837,617-154,028,562 | q36.2 | Allelic Imbalance | 190945 | 69.05 | 31.11 | 37.94 | 0.00 | 0.01 | DPP6 |
| chr7:154,105,588-154,115,577 | q36.2 | Allelic Imbalance | 9989 | 69.05 | 31.11 | 37.94 | 0.00 | 0.01 | DPP6 |
| chr7:154,224,828-154,276,703 | q36.2 | Allelic Imbalance | 51875 | 66.67 | 28.89 | 37.78 | 0.00 | 0.01 | DPP6 |
| chr7:154,279,934-154,319,803 | q36.2 | Allelic Imbalance | 39869 | 66.67 | 28.89 | 37.78 | 0.00 | 0.01 | DPP6 |
| chr7:155,358,532-155,398,994 | q36.3 | Allelic Imbalance | 40462 | 71.43 | 33.33 | 38.10 | 0.00 | 0.01 |  |
| chr7:155,761,154-155,776,367 | q36.3 | Allelic Imbalance | 15213 | 69.05 | 31.11 | 37.94 | 0.00 | 0.01 |  |
| chr7:156,036,209-156,054,523 | q36.3 | Allelic Imbalance | 18314 | 69.05 | 31.11 | 37.94 | 0.00 | 0.01 |  |
| chr7:156,063,466-156,089,261 | q36.3 | Allelic Imbalance | 25795 | 69.05 | 31.11 | 37.94 | 0.00 | 0.01 |  |
| chr7:156,407,649-156,431,889 | q36.3 | Allelic Imbalance | 24240 | 69.05 | 31.11 | 37.94 | 0.00 | 0.01 |  |
| chr7:77,609,277-77,638,108 | q21.11 | Allelic Imbalance | 28831 | 64.29 | 26.67 | 37.62 | 0.00 | 0.01 | MAGI2 |
| chr7:77,660,863-77,722,079 | q21.11 | Allelic Imbalance | 61216 | 66.67 | 28.89 | 37.78 | 0.00 | 0.01 | MAGI2 |
| chr7:77,723,283-77,852,528 | q21.11 | Allelic Imbalance | 129245 | 66.67 | 28.89 | 37.78 | 0.00 | 0.01 | RPL13AP17, MIR548AU, MAGI2 |
| chr7:77,852,528-78,044,847 | q21.11 | Allelic Imbalance | 192319 | 69.05 | 31.11 | 37.94 | 0.00 | 0.01 | MAGI2 |
| chr7:78,113,291-78,179,402 | q21.11 | Allelic Imbalance | 66111 | 66.67 | 28.89 | 37.78 | 0.00 | 0.01 | MAGI2 |
| chr7:78,310,757-78,338,911 | q21.11 | Allelic Imbalance | 28154 | 66.67 | 28.89 | 37.78 | 0.00 | 0.01 | MAGI2 |
| chr7:78,342,384-78,352,166 | q21.11 | Allelic Imbalance | 9782 | 64.29 | 26.67 | 37.62 | 0.00 | 0.01 | MAGI2 |
| chr7:80,394,648-80,735,294 | q21.11 | Allelic Imbalance | 340646 | 64.29 | 26.67 | 37.62 | 0.00 | 0.01 |  |
| chr7:81,333,710-81,375,889 | q21.11 | Allelic Imbalance | 42179 | 64.29 | 26.67 | 37.62 | 0.00 | 0.01 |  |
| chr7:81,660,620-81,707,927 | q21.11 | Allelic Imbalance | 47307 | 64.29 | 26.67 | 37.62 | 0.00 | 0.01 | CACNA2D1 |
| chr7:81,799,137-81,803,500 | q21.11 | Allelic Imbalance | 4363 | 64.29 | 26.67 | 37.62 | 0.00 | 0.01 | CACNA2D1 |
| chr7:81,824,444-81,842,467 | q21.11 | Allelic Imbalance | 18023 | 66.67 | 28.89 | 37.78 | 0.00 | 0.01 | CACNA2D1 |
| chr7:81,849,015-81,943,891 | q21.11 | Allelic Imbalance | 94876 | 66.67 | 28.89 | 37.78 | 0.00 | 0.01 | CACNA2D1 |
| chr7:82,016,375-82,061,107 | q21.11 | Allelic Imbalance | 44732 | 66.67 | 28.89 | 37.78 | 0.00 | 0.01 |  |
| chr7:82,297,407-82,400,356 | q21.11 | Allelic Imbalance | 102949 | 66.67 | 28.89 | 37.78 | 0.00 | 0.01 | PCLO |
| chr7:83,102,980-83,172,002 | q21.11 | Allelic Imbalance | 69022 | 64.29 | 26.67 | 37.62 | 0.00 | 0.01 | SEMA3E |
| chr7:83,283,744-83,346,287 | q21.11 | Allelic Imbalance | 62543 | 64.29 | 26.67 | 37.62 | 0.00 | 0.01 |  |
| chr7:93,232,213-93,234,315 | q21.3 | Allelic Imbalance | 2102 | 64.29 | 26.67 | 37.62 | 0.00 | 0.01 |  |
| chr7:93,247,814-93,258,752 | q21.3 | Allelic Imbalance | 10938 | 64.29 | 26.67 | 37.62 | 0.00 | 0.01 |  |
| chr7:93,258,752-93,287,872 | q21.3 | Allelic Imbalance | 29120 | 66.67 | 28.89 | 37.78 | 0.00 | 0.01 |  |
| chr7:93,300,316-93,322,371 | q21.3 | Allelic Imbalance | 22055 | 66.67 | 28.89 | 37.78 | 0.00 | 0.01 |  |
| chr7:93,388,857-93,403,117 | q21.3 | Allelic Imbalance | 14260 | 66.67 | 28.89 | 37.78 | 0.00 | 0.01 | GNG11 |
| chr7:93,411,986-93,469,062 | q21.3 | Allelic Imbalance | 57076 | 66.67 | 28.89 | 37.78 | 0.00 | 0.01 | BET1 |
| chr7:94,772,450-94,775,228 | q21.3 | Allelic Imbalance | 2778 | 64.29 | 26.67 | 37.62 | 0.00 | 0.01 | PON1 |
| chr7:94,786,731-94,787,021 | q21.3 | Allelic Imbalance | 290 | 66.67 | 28.89 | 37.78 | 0.00 | 0.01 | PON1 |
| chr7:96,672,581-96,717,720 | q21.3 | Allelic Imbalance | 45139 | 71.43 | 33.33 | 38.10 | 0.00 | 0.01 |  |
| chr10:42,847,259-42,906,746 | q11.21 | Allelic Imbalance | 59487 | 45.24 | 11.11 | 34.13 | 0.00 | 0.01 | RET |
| chr10:42,908,150-42,916,354 | q11.21 | Allelic Imbalance | 8204 | 45.24 | 11.11 | 34.13 | 0.00 | 0.01 | RET |
| chr10:42,925,288-42,944,475 | q11.21 | Allelic Imbalance | 19187 | 45.24 | 11.11 | 34.13 | 0.00 | 0.01 | RET |
| chr10:43,076,205-43,148,868 | q11.21 | Allelic Imbalance | 72663 | 45.24 | 11.11 | 34.13 | 0.00 | 0.01 | RASGEF1A |
| chr10:43,181,039-43,372,822 | q11.21 | Allelic Imbalance | 191783 | 45.24 | 11.11 | 34.13 | 0.00 | 0.01 | FXYD4, HNRNPF, ZNF487P, ZNF239 |
| chr10:54,976,332-55,028,162 | q21.1 | Allelic Imbalance | 51830 | 45.24 | 11.11 | 34.13 | 0.00 | 0.01 |  |
| chr10:61,510,999-61,609,061 | q21.2 | Allelic Imbalance | 98062 | 45.24 | 11.11 | 34.13 | 0.00 | 0.01 | ANK3 |
| chr10:67,386,799-67,429,028 | q21.3 | Allelic Imbalance | 42229 | 45.24 | 11.11 | 34.13 | 0.00 | 0.01 | CTNNA3 |
| chr10:96,026,671-96,088,423 | q23.33 | Allelic Imbalance | 61752 | 45.24 | 11.11 | 34.13 | 0.00 | 0.01 | PLCE1, LOC100128054, NOC3L |
| chr10:96,117,843-96,181,830 | q23.33 | Allelic Imbalance | 63987 | 45.24 | 11.11 | 34.13 | 0.00 | 0.01 | TBC1D12 |
| chr10:58,265,871-58,453,012 | q21.1 | Allelic Imbalance | 187141 | 33.33 | 4.44 | 28.89 | 0.00 | 0.01 |  |
| chr10:89,635,430-89,651,150 | q23.31 | Allelic Imbalance | 15720 | 33.33 | 4.44 | 28.89 | 0.00 | 0.01 | PTEN |
| chr10:89,696,558-89,709,816 | q23.31 | Allelic Imbalance | 13258 | 33.33 | 4.44 | 28.89 | 0.00 | 0.01 | PTEN |
| chr7:75,370,392-75,404,650 | q11.23 | Allelic Imbalance | 34258 | 52.38 | 17.78 | 34.60 | 0.00 | 0.02 | MIR4651, POR |
| chr7:94,584,928-94,622,324 | q21.3 | Allelic Imbalance | 37396 | 52.38 | 17.78 | 34.60 | 0.00 | 0.02 | PPP1R9A |
| chr7:94,739,051-94,741,472 | q21.3 | Allelic Imbalance | 2421 | 52.38 | 17.78 | 34.60 | 0.00 | 0.02 | PPP1R9A |
| chr8:102,005,243-102,184,509 | q22.3 | Allelic Imbalance | 179266 | 2.38 | 28.89 | -26.51 | 0.00 | 0.02 | YWHAZ, FLJ42969 |
| chr8:128,414,171-128,475,694 | q24.21 | Allelic Imbalance | 61523 | 2.38 | 28.89 | -26.51 | 0.00 | 0.02 |  |
| chr10:102,933,195-102,982,234 | q24.31 | Allelic Imbalance | 49039 | 47.62 | 13.33 | 34.29 | 0.00 | 0.02 | LBX1, FLJ41350 |
| chr10:103,175,985-103,296,940 | q24.32 | Allelic Imbalance | 120955 | 47.62 | 13.33 | 34.29 | 0.00 | 0.02 | BTRC |
| chr10:103,307,503-103,329,837 | q24.32 | Allelic Imbalance | 22334 | 47.62 | 13.33 | 34.29 | 0.00 | 0.02 | POLL |
| chr10:103,477,737-103,547,646 | q24.32 | Allelic Imbalance | 69909 | 47.62 | 13.33 | 34.29 | 0.00 | 0.02 | FGF8, NPM3, MGEA5 |
| chr10:67,453,940-67,462,305 | q21.3 | Allelic Imbalance | 8365 | 47.62 | 13.33 | 34.29 | 0.00 | 0.02 | CTNNA3 |
| chr10:67,483,647-67,568,465 | q21.3 | Allelic Imbalance | 84818 | 47.62 | 13.33 | 34.29 | 0.00 | 0.02 | CTNNA3 |
| chr10:89,564,793-89,570,790 | q23.2 | Allelic Imbalance | 5997 | 47.62 | 13.33 | 34.29 | 0.00 | 0.02 | ATAD1, CFL1P1 |
| chr10:89,573,109-89,581,382 | q23.2 | Allelic Imbalance | 8273 | 47.62 | 13.33 | 34.29 | 0.00 | 0.02 | CFL1P1 |
| chr7:75,514,595-75,771,996 | q11.23 | Allelic Imbalance | 257401 | 47.62 | 13.33 | 34.29 | 0.00 | 0.02 | STYXL1, MDH2, SRRM3, HSPB1 |
| chr7:86,912,215-86,930,000 | q21.12 | Allelic Imbalance | 17785 | 47.62 | 13.33 | 34.29 | 0.00 | 0.02 | ABCB4 |
| chr7:86,930,652-86,971,077 | q21.12 | Allelic Imbalance | 40425 | 47.62 | 13.33 | 34.29 | 0.00 | 0.02 | ABCB4 |
| chr7:87,008,363-87,016,825 | q21.12 | Allelic Imbalance | 8462 | 47.62 | 13.33 | 34.29 | 0.00 | 0.02 | ABCB1 |
| chr7:87,028,004-87,039,047 | q21.12 | Allelic Imbalance | 11043 | 47.62 | 13.33 | 34.29 | 0.00 | 0.02 | ABCB1 |
| chr10:100,698,769-100,720,746 | q24.2 | Allelic Imbalance | 21977 | 40.48 | 8.89 | 31.59 | 0.00 | 0.02 | HPSE2 |
| chr10:111,845,768-111,867,071 | q25.2 | Allelic Imbalance | 21303 | 40.48 | 8.89 | 31.59 | 0.00 | 0.02 | ADD3 |
| chr10:111,919,277-111,962,358 | q25.2 | Allelic Imbalance | 43081 | 40.48 | 8.89 | 31.59 | 0.00 | 0.02 | MXI1 |
| chr10:112,054,945-112,114,718 | q25.2 | Allelic Imbalance | 59773 | 40.48 | 8.89 | 31.59 | 0.00 | 0.02 |  |
| chr10:51,831,324-52,017,130 | q11.23 | Allelic Imbalance | 185806 | 40.48 | 8.89 | 31.59 | 0.00 | 0.02 | SGMS1 |
| chr10:54,064,982-54,075,410 | q21.1 | Allelic Imbalance | 10428 | 40.48 | 8.89 | 31.59 | 0.00 | 0.02 |  |
| chr10:55,872,960-55,913,461 | q21.1 | Allelic Imbalance | 40501 | 40.48 | 8.89 | 31.59 | 0.00 | 0.02 | PCDH15 |
| chr10:56,558,234-56,670,257 | q21.1 | Allelic Imbalance | 112023 | 40.48 | 8.89 | 31.59 | 0.00 | 0.02 |  |
| chr10:61,144,083-61,198,238 | q21.1 | Allelic Imbalance | 54155 | 40.48 | 8.89 | 31.59 | 0.00 | 0.02 | M1 |
| chr10:61,260,776-61,307,876 | q21.2 | Allelic Imbalance | 47100 | 40.48 | 8.89 | 31.59 | 0.00 | 0.02 | CCDC6 |
| chr10:61,365,680-61,411,501 | q21.2 | Allelic Imbalance | 45821 | 40.48 | 8.89 | 31.59 | 0.00 | 0.02 | C10orf40 |
| chr10:61,462,237-61,462,423 | q21.2 | Allelic Imbalance | 186 | 40.48 | 8.89 | 31.59 | 0.00 | 0.02 | ANK3 |
| chr10:61,468,789-61,473,643 | q21.2 | Allelic Imbalance | 4854 | 40.48 | 8.89 | 31.59 | 0.00 | 0.02 | ANK3 |
| chr10:62,315,315-62,759,084 | q21.2 | Allelic Imbalance | 443769 | 40.48 | 8.89 | 31.59 | 0.00 | 0.02 | RHOBTB1 |
| chr10:78,834,008-78,840,531 | q22.3 | Allelic Imbalance | 6523 | 40.48 | 8.89 | 31.59 | 0.00 | 0.02 | KCNMA1 |
| chr10:89,604,361-89,624,076 | q23.31 | Allelic Imbalance | 19715 | 40.48 | 8.89 | 31.59 | 0.00 | 0.02 | KLLN, PTEN |
| chr10:96,459,916-96,511,605 | q23.33 | Allelic Imbalance | 51689 | 40.48 | 8.89 | 31.59 | 0.00 | 0.02 | CYP2C18 |
| chr7:140,360,276-140,426,097 | q34 | Allelic Imbalance | 65821 | 40.48 | 8.89 | 31.59 | 0.00 | 0.02 | MRPS33, TMEM178B |
| chr7:55,129,372-55,139,473 | p11.2 | Allelic Imbalance | 10101 | 40.48 | 8.89 | 31.59 | 0.00 | 0.02 | EGFR |
| chr7:55,329,860-55,354,196 | p11.2 | Allelic Imbalance | 24336 | 40.48 | 8.89 | 31.59 | 0.00 | 0.02 |  |
| chr12:94,912,728-94,942,811 | q23.1 | Allelic Imbalance | 30083 | 4.76 | 33.33 | -28.57 | 0.00 | 0.02 | HAL, LTA4H |
| chr8:124,897,388-125,019,184 | q24.13 | Allelic Imbalance | 121796 | 4.76 | 33.33 | -28.57 | 0.00 | 0.02 | FER1L6 |
| chr8:125,572,223-125,665,275 | q24.13 | Allelic Imbalance | 93052 | 4.76 | 33.33 | -28.57 | 0.00 | 0.02 | TATDN1, NDUFB9, MTSS1 |
| chr8:132,868,217-132,938,492 | q24.22 | Allelic Imbalance | 70275 | 4.76 | 33.33 | -28.57 | 0.00 | 0.02 |  |
| chr8:133,087,722-133,110,494 | q24.22 | Allelic Imbalance | 22772 | 4.76 | 33.33 | -28.57 | 0.00 | 0.02 | EFR3A, OC90 |
| chr8:133,115,806-133,303,383 | q24.22 | Allelic Imbalance | 187577 | 4.76 | 33.33 | -28.57 | 0.00 | 0.02 | OC90, HHLA1, KCNQ3 |
| chr8:133,419,796-133,492,508 | q24.22 | Allelic Imbalance | 72712 | 4.76 | 33.33 | -28.57 | 0.00 | 0.02 | KCNQ3 |
| chr7:103,434,275-103,465,325 | q22.1 | Allelic Imbalance | 31050 | 54.76 | 20.00 | 34.76 | 0.00 | 0.02 |  |
| chr7:103,503,438-103,577,645 | q22.1 | Allelic Imbalance | 74207 | 54.76 | 20.00 | 34.76 | 0.00 | 0.02 | ORC5 |
| chr7:150,761,906-150,795,792 | q36.1 | Allelic Imbalance | 33886 | 54.76 | 20.00 | 34.76 | 0.00 | 0.02 | CRYGN, RHEB |
| chr7:76,723,790-76,767,781 | q11.23 | Allelic Imbalance | 43991 | 54.76 | 20.00 | 34.76 | 0.00 | 0.02 | CCDC146 |
| chr7:93,896,390-93,897,212 | q21.3 | Allelic Imbalance | 822 | 54.76 | 20.00 | 34.76 | 0.00 | 0.02 | COL1A2 |
| chr7:94,745,737-94,747,716 | q21.3 | Allelic Imbalance | 1979 | 54.76 | 20.00 | 34.76 | 0.00 | 0.02 | PPP1R9A |
| chr7:103,335,665-103,362,053 | q22.1 | Allelic Imbalance | 26388 | 57.14 | 22.22 | 34.92 | 0.00 | 0.02 | RELN |
| chr7:107,730,892-107,734,426 | q31.1 | Allelic Imbalance | 3534 | 57.14 | 22.22 | 34.92 | 0.00 | 0.02 | NRCAM |
| chr7:112,337,441-112,393,863 | q31.1 | Allelic Imbalance | 56422 | 57.14 | 22.22 | 34.92 | 0.00 | 0.02 | C7orf60 |
| chr7:114,158,774-114,171,357 | q31.1 | Allelic Imbalance | 12583 | 57.14 | 22.22 | 34.92 | 0.00 | 0.02 |  |
| chr7:114,259,496-114,311,926 | q31.1 | Allelic Imbalance | 52430 | 57.14 | 22.22 | 34.92 | 0.00 | 0.02 |  |
| chr7:118,947,971-118,964,411 | q31.31 | Allelic Imbalance | 16440 | 57.14 | 22.22 | 34.92 | 0.00 | 0.02 |  |
| chr7:119,957,583-119,987,820 | q31.31 | Allelic Imbalance | 30237 | 57.14 | 22.22 | 34.92 | 0.00 | 0.02 | KCND2 |
| chr7:120,286,614-120,317,858 | q31.31 | Allelic Imbalance | 31244 | 57.14 | 22.22 | 34.92 | 0.00 | 0.02 |  |
| chr7:139,823,246-139,851,179 | q34 | Allelic Imbalance | 27933 | 57.14 | 22.22 | 34.92 | 0.00 | 0.02 | MKRN1 |
| chr7:69,635,374-69,661,863 | q11.22 | Allelic Imbalance | 26489 | 57.14 | 22.22 | 34.92 | 0.00 | 0.02 | AUTS2 |
| chr7:72,652,178-72,687,607 | q11.23 | Allelic Imbalance | 35429 | 57.14 | 22.22 | 34.92 | 0.00 | 0.02 | MLXIPL |
| chr7:75,280,376-75,325,708 | q11.23 | Allelic Imbalance | 45332 | 57.14 | 22.22 | 34.92 | 0.00 | 0.02 | CCL24 |
| chr7:76,969,577-77,280,466 | q11.23 | Allelic Imbalance | 310889 | 57.14 | 22.22 | 34.92 | 0.00 | 0.02 | PTPN12, RSBN1L-AS1, RSBN1L, TMEM60, PHTF2 |
| chr7:78,914,336-78,928,245 | q21.11 | Allelic Imbalance | 13909 | 57.14 | 22.22 | 34.92 | 0.00 | 0.02 | MAGI2, MAGI2-AS3 |
| chr7:79,126,757-79,127,529 | q21.11 | Allelic Imbalance | 772 | 57.14 | 22.22 | 34.92 | 0.00 | 0.02 |  |
| chr7:83,787,452-83,808,043 | q21.11 | Allelic Imbalance | 20591 | 57.14 | 22.22 | 34.92 | 0.00 | 0.02 |  |
| chr7:83,827,146-84,467,763 | q21.11 | Allelic Imbalance | 640617 | 57.14 | 22.22 | 34.92 | 0.00 | 0.02 | SEMA3D |
| chr7:84,546,179-84,548,889 | q21.11 | Allelic Imbalance | 2710 | 57.14 | 22.22 | 34.92 | 0.00 | 0.02 | SEMA3D |
| chr7:84,619,151-84,811,995 | q21.11 | Allelic Imbalance | 192844 | 57.14 | 22.22 | 34.92 | 0.00 | 0.02 |  |
| chr7:84,858,497-84,944,015 | q21.11 | Allelic Imbalance | 85518 | 57.14 | 22.22 | 34.92 | 0.00 | 0.02 |  |
| chr7:84,969,738-85,019,008 | q21.11 | Allelic Imbalance | 49270 | 57.14 | 22.22 | 34.92 | 0.00 | 0.02 |  |
| chr7:87,989,500-88,029,584 | q21.12 - q21.13 | Allelic Imbalance | 40084 | 57.14 | 22.22 | 34.92 | 0.00 | 0.02 |  |
| chr10:131,794,751-131,795,666 | q26.3 | Allelic Imbalance | 915 | 50.00 | 15.56 | 34.44 | 0.00 | 0.02 | LOC387723, CTAGE7P |
| chr7:30,525,989-30,597,136 | p15.1 | Allelic Imbalance | 71147 | 50.00 | 15.56 | 34.44 | 0.00 | 0.02 | LOC401320 |
| chr7:30,688,920-30,698,848 | p15.1 | Allelic Imbalance | 9928 | 50.00 | 15.56 | 34.44 | 0.00 | 0.02 | CRHR2 |
| chr7:31,993,375-32,003,820 | p14.3 | Allelic Imbalance | 10445 | 50.00 | 15.56 | 34.44 | 0.00 | 0.02 | PDE1C |
| chr7:68,406,645-68,423,461 | q11.22 | Allelic Imbalance | 16816 | 50.00 | 15.56 | 34.44 | 0.00 | 0.02 |  |
| chr7:75,404,650-75,514,595 | q11.23 | Allelic Imbalance | 109945 | 50.00 | 15.56 | 34.44 | 0.00 | 0.02 | SNORA14A, POR, TMEM120A, STYXL1 |
| chr7:75,783,498-75,784,078 | q11.23 | Allelic Imbalance | 580 | 50.00 | 15.56 | 34.44 | 0.00 | 0.02 |  |
| chr7:94,482,458-94,527,575 | q21.3 | Allelic Imbalance | 45117 | 50.00 | 15.56 | 34.44 | 0.00 | 0.02 | PPP1R9A |
| chr10:111,994,113-112,026,531 | q25.2 | Allelic Imbalance | 32418 | 35.71 | 6.67 | 29.05 | 0.00 | 0.02 | MXI1 |
| chr10:63,653,874-64,190,304 | q21.2 | Allelic Imbalance | 536430 | 35.71 | 6.67 | 29.05 | 0.00 | 0.02 | RTKN2, ZNF365 |
| chr10:89,631,976-89,635,200 | q23.31 | Allelic Imbalance | 3224 | 35.71 | 6.67 | 29.05 | 0.00 | 0.02 | PTEN |
| chr7:103,860,120-103,873,345 | q22.1 | Allelic Imbalance | 13225 | 59.52 | 24.44 | 35.08 | 0.00 | 0.02 | LHFPL3 |
| chr7:110,302,970-110,477,882 | q31.1 | Allelic Imbalance | 174912 | 59.52 | 24.44 | 35.08 | 0.00 | 0.02 | IMMP2L |
| chr7:110,614,851-110,629,251 | q31.1 | Allelic Imbalance | 14400 | 59.52 | 24.44 | 35.08 | 0.00 | 0.02 | IMMP2L |
| chr7:112,512,556-112,513,885 | q31.1 | Allelic Imbalance | 1329 | 59.52 | 24.44 | 35.08 | 0.00 | 0.02 | GPR85 |
| chr7:112,881,911-112,886,751 | q31.1 | Allelic Imbalance | 4840 | 59.52 | 24.44 | 35.08 | 0.00 | 0.02 |  |
| chr7:114,204,088-114,206,651 | q31.1 | Allelic Imbalance | 2563 | 59.52 | 24.44 | 35.08 | 0.00 | 0.02 |  |
| chr7:118,391,551-118,413,994 | q31.31 | Allelic Imbalance | 22443 | 59.52 | 24.44 | 35.08 | 0.00 | 0.02 |  |
| chr7:120,362,749-120,484,016 | q31.31 | Allelic Imbalance | 121267 | 59.52 | 24.44 | 35.08 | 0.00 | 0.02 | ING3, CPED1 |
| chr7:126,602,381-126,619,179 | q31.33 | Allelic Imbalance | 16798 | 59.52 | 24.44 | 35.08 | 0.00 | 0.02 | GRM8 |
| chr7:139,112,494-139,128,363 | q34 | Allelic Imbalance | 15869 | 59.52 | 24.44 | 35.08 | 0.00 | 0.02 | HIPK2, TBXAS1 |
| chr7:139,793,959-139,806,629 | q34 | Allelic Imbalance | 12670 | 59.52 | 24.44 | 35.08 | 0.00 | 0.02 | MKRN1 |
| chr7:140,157,207-140,286,272 | q34 | Allelic Imbalance | 129065 | 35.71 | 6.67 | 29.05 | 0.00 | 0.02 | BRAF |
| chr7:150,642,870-150,689,292 | q36.1 | Allelic Imbalance | 46422 | 59.52 | 24.44 | 35.08 | 0.00 | 0.02 | NUB1 |
| chr7:55,200,221-55,212,053 | p11.2 | Allelic Imbalance | 11832 | 35.71 | 6.67 | 29.05 | 0.00 | 0.02 | EGFR |
| chr7:55,273,766-55,274,528 | p11.2 | Allelic Imbalance | 762 | 35.71 | 6.67 | 29.05 | 0.00 | 0.02 |  |
| chr7:55,276,830-55,277,390 | p11.2 | Allelic Imbalance | 560 | 35.71 | 6.67 | 29.05 | 0.00 | 0.02 |  |
| chr7:71,045,831-71,069,600 | q11.22 | Allelic Imbalance | 23769 | 59.52 | 24.44 | 35.08 | 0.00 | 0.02 | CALN1 |
| chr7:72,721,570-72,774,504 | q11.23 | Allelic Imbalance | 52934 | 59.52 | 24.44 | 35.08 | 0.00 | 0.02 | VPS37D, DNAJC30, WBSCR22, STX1A, MIR4284 |
| chr7:72,784,478-72,802,975 | q11.23 | Allelic Imbalance | 18497 | 59.52 | 24.44 | 35.08 | 0.00 | 0.02 | ABHD11-AS1, ABHD11 |
| chr7:78,371,776-78,683,155 | q21.11 | Allelic Imbalance | 311379 | 59.52 | 24.44 | 35.08 | 0.00 | 0.02 | MAGI2, MAGI2-AS2 |
| chr7:78,893,151-78,901,058 | q21.11 | Allelic Imbalance | 7907 | 59.52 | 24.44 | 35.08 | 0.00 | 0.02 | MAGI2 |
| chr7:79,604,310-79,635,580 | q21.11 | Allelic Imbalance | 31270 | 59.52 | 24.44 | 35.08 | 0.00 | 0.02 | GNAI1 |
| chr7:81,409,895-81,439,064 | q21.11 | Allelic Imbalance | 29169 | 59.52 | 24.44 | 35.08 | 0.00 | 0.02 | CACNA2D1 |
| chr7:83,419,593-83,440,259 | q21.11 | Allelic Imbalance | 20666 | 59.52 | 24.44 | 35.08 | 0.00 | 0.02 | SEMA3A |
| chr7:83,483,821-83,506,271 | q21.11 | Allelic Imbalance | 22450 | 59.52 | 24.44 | 35.08 | 0.00 | 0.02 | SEMA3A |
| chr7:83,627,028-83,742,659 | q21.11 | Allelic Imbalance | 115631 | 59.52 | 24.44 | 35.08 | 0.00 | 0.02 | SEMA3A |
| chr7:87,892,542-87,923,595 | q21.12 | Allelic Imbalance | 31053 | 59.52 | 24.44 | 35.08 | 0.00 | 0.02 |  |
| chr7:87,937,319-87,965,478 | q21.12 | Allelic Imbalance | 28159 | 59.52 | 24.44 | 35.08 | 0.00 | 0.02 |  |
| chr7:88,151,557-88,152,620 | q21.13 | Allelic Imbalance | 1063 | 35.71 | 6.67 | 29.05 | 0.00 | 0.02 |  |
| chr7:88,163,180-88,216,552 | q21.13 | Allelic Imbalance | 53372 | 35.71 | 6.67 | 29.05 | 0.00 | 0.02 |  |
| chr7:88,235,285-88,242,039 | q21.13 | Allelic Imbalance | 6754 | 35.71 | 6.67 | 29.05 | 0.00 | 0.02 | ZNF804B |
| chr7:93,865,673-93,868,376 | q21.3 | Allelic Imbalance | 2703 | 59.52 | 24.44 | 35.08 | 0.00 | 0.02 | COL1A2 |
| chr7:93,885,702-93,885,869 | q21.3 | Allelic Imbalance | 167 | 59.52 | 24.44 | 35.08 | 0.00 | 0.02 | COL1A2 |
| chr7:93,890,348-93,892,307 | q21.3 | Allelic Imbalance | 1959 | 59.52 | 24.44 | 35.08 | 0.00 | 0.02 | COL1A2 |
| chr12:3,028,927-3,051,199 | p13.33 | Allelic Imbalance | 22272 | 9.52 | 40.00 | -30.48 | 0.00 | 0.02 |  |
| chr7:103,369,461-103,374,174 | q22.1 | Allelic Imbalance | 4713 | 61.90 | 26.67 | 35.24 | 0.00 | 0.02 | RELN |
| chr7:103,376,974-103,380,416 | q22.1 | Allelic Imbalance | 3442 | 61.90 | 26.67 | 35.24 | 0.00 | 0.02 | RELN |
| chr7:103,921,146-103,942,302 | q22.1 | Allelic Imbalance | 21156 | 61.90 | 26.67 | 35.24 | 0.00 | 0.02 | LHFPL3 |
| chr7:105,258,774-105,359,608 | q22.2 | Allelic Imbalance | 100834 | 66.67 | 31.11 | 35.56 | 0.00 | 0.02 | ATXN7L1 |
| chr7:105,428,092-105,570,826 | q22.2 | Allelic Imbalance | 142734 | 69.05 | 33.33 | 35.71 | 0.00 | 0.02 | CDHR3, SYPL1 |
| chr7:107,112,019-107,185,501 | q22.3 | Allelic Imbalance | 73482 | 61.90 | 26.67 | 35.24 | 0.00 | 0.02 | SLC26A4, CBLL1 |
| chr7:107,189,334-107,242,125 | q22.3 - q31.1 | Allelic Imbalance | 52791 | 61.90 | 26.67 | 35.24 | 0.00 | 0.02 | SLC26A3 |
| chr7:107,811,759-107,813,205 | q31.1 | Allelic Imbalance | 1446 | 61.90 | 26.67 | 35.24 | 0.00 | 0.02 | NRCAM |
| chr7:107,818,456-107,896,915 | q31.1 | Allelic Imbalance | 78459 | 61.90 | 26.67 | 35.24 | 0.00 | 0.02 | NRCAM |
| chr7:109,431,217-109,431,871 | q31.1 | Allelic Imbalance | 654 | 64.29 | 28.89 | 35.40 | 0.00 | 0.02 |  |
| chr7:109,461,585-109,473,916 | q31.1 | Allelic Imbalance | 12331 | 64.29 | 28.89 | 35.40 | 0.00 | 0.02 |  |
| chr7:109,605,031-109,606,386 | q31.1 | Allelic Imbalance | 1355 | 66.67 | 31.11 | 35.56 | 0.00 | 0.02 |  |
| chr7:109,679,396-109,711,773 | q31.1 | Allelic Imbalance | 32377 | 64.29 | 28.89 | 35.40 | 0.00 | 0.02 |  |
| chr7:110,106,642-110,112,378 | q31.1 | Allelic Imbalance | 5736 | 61.90 | 26.67 | 35.24 | 0.00 | 0.02 | IMMP2L |
| chr7:110,151,655-110,187,389 | q31.1 | Allelic Imbalance | 35734 | 61.90 | 26.67 | 35.24 | 0.00 | 0.02 | IMMP2L |
| chr7:110,270,992-110,292,691 | q31.1 | Allelic Imbalance | 21699 | 61.90 | 26.67 | 35.24 | 0.00 | 0.02 | IMMP2L |
| chr7:110,992,580-111,047,341 | q31.1 | Allelic Imbalance | 54761 | 61.90 | 26.67 | 35.24 | 0.00 | 0.02 |  |
| chr7:112,827,631-112,836,424 | q31.1 | Allelic Imbalance | 8793 | 61.90 | 26.67 | 35.24 | 0.00 | 0.02 |  |
| chr7:112,843,468-112,875,707 | q31.1 | Allelic Imbalance | 32239 | 61.90 | 26.67 | 35.24 | 0.00 | 0.02 |  |
| chr7:121,609,051-121,777,022 | q31.32 | Allelic Imbalance | 167971 | 69.05 | 33.33 | 35.71 | 0.00 | 0.02 | FEZF1, FEZF1-AS1, CADPS2 |
| chr7:121,881,815-121,943,310 | q31.32 | Allelic Imbalance | 61495 | 66.67 | 31.11 | 35.56 | 0.00 | 0.02 | CADPS2 |
| chr7:126,619,179-126,634,889 | q31.33 | Allelic Imbalance | 15710 | 61.90 | 26.67 | 35.24 | 0.00 | 0.02 | GRM8 |
| chr7:126,661,477-126,737,836 | q31.33 | Allelic Imbalance | 76359 | 66.67 | 31.11 | 35.56 | 0.00 | 0.02 | GRM8 |
| chr7:127,630,406-127,665,418 | q32.1 | Allelic Imbalance | 35012 | 69.05 | 33.33 | 35.71 | 0.00 | 0.02 | MIR129-1 |
| chr7:134,292,215-134,301,472 | q33 | Allelic Imbalance | 9257 | 66.67 | 31.11 | 35.56 | 0.00 | 0.02 | CALD1 |
| chr7:138,985,366-138,989,098 | q34 | Allelic Imbalance | 3732 | 71.43 | 35.56 | 35.87 | 0.00 | 0.02 | HIPK2 |
| chr7:139,006,498-139,010,174 | q34 | Allelic Imbalance | 3676 | 66.67 | 31.11 | 35.56 | 0.00 | 0.02 | HIPK2 |
| chr7:139,045,026-139,047,853 | q34 | Allelic Imbalance | 2827 | 64.29 | 28.89 | 35.40 | 0.00 | 0.02 | HIPK2 |
| chr7:139,192,491-139,204,873 | q34 | Allelic Imbalance | 12382 | 61.90 | 26.67 | 35.24 | 0.00 | 0.02 | TBXAS1 |
| chr7:139,225,837-139,235,455 | q34 | Allelic Imbalance | 9618 | 64.29 | 28.89 | 35.40 | 0.00 | 0.02 | TBXAS1 |
| chr7:140,640,244-140,671,448 | q34 | Allelic Imbalance | 31204 | 64.29 | 28.89 | 35.40 | 0.00 | 0.02 | TMEM178B |
| chr7:146,208,878-146,231,477 | q35 | Allelic Imbalance | 22599 | 66.67 | 31.11 | 35.56 | 0.00 | 0.02 | CNTNAP2 |
| chr7:146,281,873-146,595,871 | q35 | Allelic Imbalance | 313998 | 69.05 | 33.33 | 35.71 | 0.00 | 0.02 | CNTNAP2 |
| chr7:147,131,428-147,200,573 | q35 | Allelic Imbalance | 69145 | 69.05 | 33.33 | 35.71 | 0.00 | 0.02 | CNTNAP2 |
| chr7:147,941,779-147,957,162 | q36.1 | Allelic Imbalance | 15383 | 66.67 | 31.11 | 35.56 | 0.00 | 0.02 | C7orf33 |
| chr7:148,647,119-148,672,644 | q36.1 | Allelic Imbalance | 25525 | 66.67 | 31.11 | 35.56 | 0.00 | 0.02 |  |
| chr7:149,773,216-149,793,802 | q36.1 | Allelic Imbalance | 20586 | 66.67 | 31.11 | 35.56 | 0.00 | 0.02 | LOC285972, GIMAP8 |
| chr7:149,912,645-149,983,809 | q36.1 | Allelic Imbalance | 71164 | 61.90 | 26.67 | 35.24 | 0.00 | 0.02 | GIMAP6 |
| chr7:150,003,095-150,017,433 | q36.1 | Allelic Imbalance | 14338 | 64.29 | 28.89 | 35.40 | 0.00 | 0.02 | GIMAP2 |
| chr7:150,363,570-150,394,158 | q36.1 | Allelic Imbalance | 30588 | 66.67 | 31.11 | 35.56 | 0.00 | 0.02 | ABCB8, ASIC3, CDK5, SLC4A2 |
| chr7:151,110,424-151,214,176 | q36.1 | Allelic Imbalance | 103752 | 66.67 | 31.11 | 35.56 | 0.00 | 0.02 | PRKAG2, PRKAG2-AS1 |
| chr7:151,835,301-151,899,796 | q36.1 | Allelic Imbalance | 64495 | 66.67 | 31.11 | 35.56 | 0.00 | 0.02 |  |
| chr7:151,981,357-152,001,822 | q36.1 | Allelic Imbalance | 20465 | 66.67 | 31.11 | 35.56 | 0.00 | 0.02 | XRCC2 |
| chr7:152,011,733-152,016,553 | q36.1 | Allelic Imbalance | 4820 | 69.05 | 33.33 | 35.71 | 0.00 | 0.02 |  |
| chr7:152,236,827-152,313,363 | q36.2 | Allelic Imbalance | 76536 | 69.05 | 33.33 | 35.71 | 0.00 | 0.02 |  |
| chr7:152,608,826-152,712,136 | q36.2 | Allelic Imbalance | 103310 | 69.05 | 33.33 | 35.71 | 0.00 | 0.02 |  |
| chr7:152,847,409-152,918,723 | q36.2 | Allelic Imbalance | 71314 | 69.05 | 33.33 | 35.71 | 0.00 | 0.02 |  |
| chr7:153,808,274-153,837,617 | q36.2 | Allelic Imbalance | 29343 | 66.67 | 31.11 | 35.56 | 0.00 | 0.02 | DPP6 |
| chr7:154,028,562-154,105,588 | q36.2 | Allelic Imbalance | 77026 | 69.05 | 33.33 | 35.71 | 0.00 | 0.02 | DPP6 |
| chr7:154,115,577-154,224,828 | q36.2 | Allelic Imbalance | 109251 | 66.67 | 31.11 | 35.56 | 0.00 | 0.02 | DPP6 |
| chr7:154,319,803-154,346,684 | q36.2 | Allelic Imbalance | 26881 | 66.67 | 31.11 | 35.56 | 0.00 | 0.02 |  |
| chr7:154,539,127-154,561,496 | q36.2 | Allelic Imbalance | 22369 | 66.67 | 31.11 | 35.56 | 0.00 | 0.02 |  |
| chr7:154,597,596-154,805,838 | q36.2 - q36.3 | Allelic Imbalance | 208242 | 69.05 | 33.33 | 35.71 | 0.00 | 0.02 | INSIG1 |
| chr7:155,332,517-155,358,532 | q36.3 | Allelic Imbalance | 26015 | 69.05 | 33.33 | 35.71 | 0.00 | 0.02 |  |
| chr7:155,776,367-156,036,209 | q36.3 | Allelic Imbalance | 259842 | 69.05 | 33.33 | 35.71 | 0.00 | 0.02 | LOC285889, LINC00244 |
| chr7:156,054,523-156,063,466 | q36.3 | Allelic Imbalance | 8943 | 66.67 | 31.11 | 35.56 | 0.00 | 0.02 |  |
| chr7:156,089,261-156,307,650 | q36.3 | Allelic Imbalance | 218389 | 66.67 | 31.11 | 35.56 | 0.00 | 0.02 | C7orf13, RNF32, LMBR1 |
| chr7:156,374,795-156,407,649 | q36.3 | Allelic Imbalance | 32854 | 66.67 | 31.11 | 35.56 | 0.00 | 0.02 | LMBR1 |
| chr7:156,431,889-156,503,804 | q36.3 | Allelic Imbalance | 71915 | 66.67 | 31.11 | 35.56 | 0.00 | 0.02 | NOM1, MNX1, LOC645249 |
| chr7:157,142,167-157,483,415 | q36.3 | Allelic Imbalance | 341248 | 69.05 | 33.33 | 35.71 | 0.00 | 0.02 | LOC100506585, PTPRN2 |
| chr7:77,638,108-77,660,863 | q21.11 | Allelic Imbalance | 22755 | 64.29 | 28.89 | 35.40 | 0.00 | 0.02 | MAGI2 |
| chr7:77,722,079-77,723,283 | q21.11 | Allelic Imbalance | 1204 | 64.29 | 28.89 | 35.40 | 0.00 | 0.02 | MAGI2 |
| chr7:78,044,847-78,113,291 | q21.11 | Allelic Imbalance | 68444 | 66.67 | 31.11 | 35.56 | 0.00 | 0.02 | MAGI2 |
| chr7:80,735,294-80,779,511 | q21.11 | Allelic Imbalance | 44217 | 61.90 | 26.67 | 35.24 | 0.00 | 0.02 |  |
| chr7:81,268,429-81,333,710 | q21.11 | Allelic Imbalance | 65281 | 61.90 | 26.67 | 35.24 | 0.00 | 0.02 |  |
| chr7:81,536,857-81,618,732 | q21.11 | Allelic Imbalance | 81875 | 64.29 | 28.89 | 35.40 | 0.00 | 0.02 | CACNA2D1 |
| chr7:81,618,732-81,660,620 | q21.11 | Allelic Imbalance | 41888 | 61.90 | 26.67 | 35.24 | 0.00 | 0.02 | CACNA2D1 |
| chr7:83,011,673-83,102,980 | q21.11 | Allelic Imbalance | 91307 | 61.90 | 26.67 | 35.24 | 0.00 | 0.02 | SEMA3E |
| chr7:83,172,002-83,283,744 | q21.11 | Allelic Imbalance | 111742 | 64.29 | 28.89 | 35.40 | 0.00 | 0.02 |  |
| chr7:83,346,287-83,377,069 | q21.11 | Allelic Imbalance | 30782 | 61.90 | 26.67 | 35.24 | 0.00 | 0.02 |  |
| chr7:89,173,989-89,176,316 | q21.13 | Allelic Imbalance | 2327 | 61.90 | 26.67 | 35.24 | 0.00 | 0.02 |  |
| chr7:90,493,213-90,550,913 | q21.13 | Allelic Imbalance | 57700 | 66.67 | 31.11 | 35.56 | 0.00 | 0.02 | CDK14 |
| chr7:90,579,228-90,581,440 | q21.13 | Allelic Imbalance | 2212 | 66.67 | 31.11 | 35.56 | 0.00 | 0.02 | CDK14 |
| chr7:93,038,016-93,232,213 | q21.3 | Allelic Imbalance | 194197 | 61.90 | 26.67 | 35.24 | 0.00 | 0.02 | CALCR, MIR4652 |
| chr7:93,234,315-93,247,814 | q21.3 | Allelic Imbalance | 13499 | 64.29 | 28.89 | 35.40 | 0.00 | 0.02 |  |
| chr7:93,322,371-93,388,857 | q21.3 | Allelic Imbalance | 66486 | 64.29 | 28.89 | 35.40 | 0.00 | 0.02 | TFPI2, GNGT1 |
| chr7:93,469,062-93,514,853 | q21.3 | Allelic Imbalance | 45791 | 64.29 | 28.89 | 35.40 | 0.00 | 0.02 | BET1 |
| chr7:93,869,114-93,873,499 | q21.3 | Allelic Imbalance | 4385 | 61.90 | 26.67 | 35.24 | 0.00 | 0.02 | COL1A2 |
| chr7:93,884,544-93,884,799 | q21.3 | Allelic Imbalance | 255 | 61.90 | 26.67 | 35.24 | 0.00 | 0.02 | COL1A2 |
| chr7:94,769,536-94,772,450 | q21.3 | Allelic Imbalance | 2914 | 61.90 | 26.67 | 35.24 | 0.00 | 0.02 | PON1 |
| chr7:94,775,228-94,786,731 | q21.3 | Allelic Imbalance | 11503 | 64.29 | 28.89 | 35.40 | 0.00 | 0.02 | PON1 |
| chr7:94,787,021-94,787,536 | q21.3 | Allelic Imbalance | 515 | 64.29 | 28.89 | 35.40 | 0.00 | 0.02 | PON1 |
| chr7:96,642,231-96,672,581 | q21.3 | Allelic Imbalance | 30350 | 69.05 | 33.33 | 35.71 | 0.00 | 0.02 | ACN9 |
| chr7:96,717,720-96,721,184 | q21.3 | Allelic Imbalance | 3464 | 69.05 | 33.33 | 35.71 | 0.00 | 0.02 |  |
| chr7:96,806,966-96,808,273 | q21.3 | Allelic Imbalance | 1307 | 69.05 | 33.33 | 35.71 | 0.00 | 0.02 |  |
| chr7:96,850,540-96,936,580 | q21.3 | Allelic Imbalance | 86040 | 69.05 | 33.33 | 35.71 | 0.00 | 0.02 |  |
| chr10:100,520,969-100,555,840 | q24.2 | Allelic Imbalance | 34871 | 42.86 | 11.11 | 31.75 | 0.00 | 0.02 | HPSE2 |
| chr10:43,148,868-43,181,039 | q11.21 | Allelic Imbalance | 32171 | 42.86 | 11.11 | 31.75 | 0.00 | 0.02 |  |
| chr10:43,372,822-43,398,120 | q11.21 | Allelic Imbalance | 25298 | 42.86 | 11.11 | 31.75 | 0.00 | 0.02 | ZNF239 |
| chr10:52,313,114-52,320,142 | q11.23 | Allelic Imbalance | 7028 | 42.86 | 11.11 | 31.75 | 0.00 | 0.02 | A1CF |
| chr10:52,816,873-52,899,243 | q11.23 | Allelic Imbalance | 82370 | 42.86 | 11.11 | 31.75 | 0.00 | 0.02 | PRKG1 |
| chr10:55,057,502-55,151,612 | q21.1 | Allelic Imbalance | 94110 | 42.86 | 11.11 | 31.75 | 0.00 | 0.02 |  |
| chr10:61,609,061-61,681,807 | q21.2 | Allelic Imbalance | 72746 | 42.86 | 11.11 | 31.75 | 0.00 | 0.02 | ANK3 |
| chr10:66,862,492-66,973,588 | q21.3 | Allelic Imbalance | 111096 | 42.86 | 11.11 | 31.75 | 0.00 | 0.02 |  |
| chr10:67,296,581-67,386,799 | q21.3 | Allelic Imbalance | 90218 | 42.86 | 11.11 | 31.75 | 0.00 | 0.02 | CTNNA3 |
| chr10:69,582,053-69,589,110 | q21.3 | Allelic Imbalance | 7057 | 42.86 | 11.11 | 31.75 | 0.00 | 0.02 | MYPN |
| chr10:71,783,052-71,954,470 | q22.1 | Allelic Imbalance | 171418 | 42.86 | 11.11 | 31.75 | 0.00 | 0.02 | LRRC20, EIF4EBP2, NODAL, PALD1 |
| chr10:94,515,569-94,528,069 | q23.33 | Allelic Imbalance | 12500 | 42.86 | 11.11 | 31.75 | 0.00 | 0.02 |  |
| chr10:95,960,867-96,026,671 | q23.33 | Allelic Imbalance | 65804 | 42.86 | 11.11 | 31.75 | 0.00 | 0.02 | PLCE1 |
| chr10:96,088,423-96,117,843 | q23.33 | Allelic Imbalance | 29420 | 42.86 | 11.11 | 31.75 | 0.00 | 0.02 | NOC3L |
| chr7:139,900,303-139,928,889 | q34 | Allelic Imbalance | 28586 | 42.86 | 11.11 | 31.75 | 0.00 | 0.02 | DENND2A |
| chr7:88,390,994-88,395,501 | q21.13 | Allelic Imbalance | 4507 | 42.86 | 11.11 | 31.75 | 0.00 | 0.02 | ZNF804B |
| chr7:92,138,211-92,151,211 | q21.2 | Allelic Imbalance | 13000 | 42.86 | 11.11 | 31.75 | 0.00 | 0.02 | CDK6 |
| chr7:92,170,827-92,194,800 | q21.2 | Allelic Imbalance | 23973 | 42.86 | 11.11 | 31.75 | 0.00 | 0.02 | CDK6 |
| chr10:58,199,360-58,265,871 | q21.1 | Allelic Imbalance | 66511 | 30.95 | 4.44 | 26.51 | 0.00 | 0.02 |  |
| chr10:89,651,150-89,676,743 | q23.31 | Allelic Imbalance | 25593 | 30.95 | 4.44 | 26.51 | 0.00 | 0.02 | PTEN |
| chr14:32,984,938-33,122,988 | q13.1 | Allelic Imbalance | 138050 | 30.95 | 4.44 | 26.51 | 0.00 | 0.02 | NPAS3 |
| chr12:2,315,215-2,376,278 | p13.33 | Allelic Imbalance | 61063 | 7.14 | 35.56 | -28.41 | 0.00 | 0.03 | CACNA1C |
| chr12:80,274,682-80,373,443 | q21.31 | Allelic Imbalance | 98761 | 7.14 | 35.56 | -28.41 | 0.00 | 0.03 | PPFIA2 |
| chr8:124,856,286-124,897,388 | q24.13 | Allelic Imbalance | 41102 | 7.14 | 35.56 | -28.41 | 0.00 | 0.03 | FAM91A1 |
| chr10:102,875,861-102,933,195 | q24.31 | Allelic Imbalance | 57334 | 45.24 | 13.33 | 31.90 | 0.00 | 0.03 | TLX1NB, TLX1 |
| chr10:103,329,837-103,477,737 | q24.32 | Allelic Imbalance | 147900 | 45.24 | 13.33 | 31.90 | 0.00 | 0.03 | POLL, DPCD, MIR3158-1, MIR3158-2, FBXW4 |
| chr10:114,724,956-114,878,155 | q25.2 | Allelic Imbalance | 153199 | 45.24 | 13.33 | 31.90 | 0.00 | 0.03 | TCF7L2 |
| chr10:42,805,486-42,847,259 | q11.21 | Allelic Imbalance | 41773 | 45.24 | 13.33 | 31.90 | 0.00 | 0.03 | MIR5100 |
| chr10:42,916,354-42,925,288 | q11.21 | Allelic Imbalance | 8934 | 45.24 | 13.33 | 31.90 | 0.00 | 0.03 | RET |
| chr10:47,955,419-47,975,145 | q11.22 | Allelic Imbalance | 19726 | 45.24 | 13.33 | 31.90 | 0.00 | 0.03 | ZNF488 |
| chr10:47,997,935-48,182,070 | q11.22 | Allelic Imbalance | 184135 | 45.24 | 13.33 | 31.90 | 0.00 | 0.03 | RBP3, GDF2, GDF10 |
| chr10:55,028,162-55,041,700 | q21.1 | Allelic Imbalance | 13538 | 45.24 | 13.33 | 31.90 | 0.00 | 0.03 |  |
| chr10:67,429,028-67,453,940 | q21.3 | Allelic Imbalance | 24912 | 45.24 | 13.33 | 31.90 | 0.00 | 0.03 | CTNNA3 |
| chr10:67,462,305-67,483,647 | q21.3 | Allelic Imbalance | 21342 | 45.24 | 13.33 | 31.90 | 0.00 | 0.03 | CTNNA3 |
| chr10:72,211,736-72,362,664 | q22.1 | Allelic Imbalance | 150928 | 45.24 | 13.33 | 31.90 | 0.00 | 0.03 | TBATA, SGPL1, PCBD1 |
| chr10:88,615,278-88,745,952 | q23.2 | Allelic Imbalance | 130674 | 45.24 | 13.33 | 31.90 | 0.00 | 0.03 | BMPR1A, MMRN2, SNCG, C10orf116, AGAP11 |
| chr10:89,570,790-89,572,968 | q23.2 | Allelic Imbalance | 2178 | 45.24 | 13.33 | 31.90 | 0.00 | 0.03 | CFL1P1 |
| chr10:89,581,382-89,587,936 | q23.2 | Allelic Imbalance | 6554 | 45.24 | 13.33 | 31.90 | 0.00 | 0.03 | CFL1P1 |
| chr10:92,411,116-92,482,444 | q23.31 | Allelic Imbalance | 71328 | 45.24 | 13.33 | 31.90 | 0.00 | 0.03 |  |
| chr10:95,549,205-95,573,792 | q23.33 | Allelic Imbalance | 24587 | 45.24 | 13.33 | 31.90 | 0.00 | 0.03 |  |
| chr10:96,181,830-96,377,369 | q23.33 | Allelic Imbalance | 195539 | 45.24 | 13.33 | 31.90 | 0.00 | 0.03 | TBC1D12, HELLS |
| chr7:140,438,682-140,452,289 | q34 | Allelic Imbalance | 13607 | 45.24 | 13.33 | 31.90 | 0.00 | 0.03 | TMEM178B |
| chr7:86,886,873-86,912,215 | q21.12 | Allelic Imbalance | 25342 | 45.24 | 13.33 | 31.90 | 0.00 | 0.03 | ABCB4 |
| chr7:87,039,047-87,047,262 | q21.12 | Allelic Imbalance | 8215 | 45.24 | 13.33 | 31.90 | 0.00 | 0.03 | ABCB1 |
| chr10:100,623,960-100,698,769 | q24.2 | Allelic Imbalance | 74809 | 38.10 | 8.89 | 29.21 | 0.00 | 0.03 | HPSE2 |
| chr10:100,720,746-101,093,245 | q24.2 | Allelic Imbalance | 372499 | 38.10 | 8.89 | 29.21 | 0.00 | 0.03 | HPSE2, CNNM1 |
| chr10:112,030,002-112,054,945 | q25.2 | Allelic Imbalance | 24943 | 38.10 | 8.89 | 29.21 | 0.00 | 0.03 | MXI1, SMNDC1 |
| chr10:123,626,944-123,903,280 | q26.13 | Allelic Imbalance | 276336 | 38.10 | 8.89 | 29.21 | 0.00 | 0.03 | ATE1, NSMCE4A, TACC2 |
| chr10:53,931,514-54,064,982 | q21.1 | Allelic Imbalance | 133468 | 38.10 | 8.89 | 29.21 | 0.00 | 0.03 |  |
| chr10:55,151,612-55,165,861 | q21.1 | Allelic Imbalance | 14249 | 38.10 | 8.89 | 29.21 | 0.00 | 0.03 |  |
| chr10:55,913,461-56,242,948 | q21.1 | Allelic Imbalance | 329487 | 38.10 | 8.89 | 29.21 | 0.00 | 0.03 | PCDH15 |
| chr10:56,338,167-56,384,264 | q21.1 | Allelic Imbalance | 46097 | 38.10 | 8.89 | 29.21 | 0.00 | 0.03 |  |
| chr10:56,399,622-56,558,234 | q21.1 | Allelic Imbalance | 158612 | 38.10 | 8.89 | 29.21 | 0.00 | 0.03 |  |
| chr10:56,670,257-56,673,081 | q21.1 | Allelic Imbalance | 2824 | 38.10 | 8.89 | 29.21 | 0.00 | 0.03 |  |
| chr10:62,306,334-62,315,315 | q21.2 | Allelic Imbalance | 8981 | 38.10 | 8.89 | 29.21 | 0.00 | 0.03 | RHOBTB1 |
| chr10:62,759,084-62,826,768 | q21.2 | Allelic Imbalance | 67684 | 38.10 | 8.89 | 29.21 | 0.00 | 0.03 |  |
| chr10:64,439,944-64,493,797 | q21.2 | Allelic Imbalance | 53853 | 38.10 | 8.89 | 29.21 | 0.00 | 0.03 |  |
| chr10:78,794,366-78,834,008 | q22.3 | Allelic Imbalance | 39642 | 38.10 | 8.89 | 29.21 | 0.00 | 0.03 | KCNMA1 |
| chr10:89,624,076-89,629,269 | q23.31 | Allelic Imbalance | 5193 | 38.10 | 8.89 | 29.21 | 0.00 | 0.03 | PTEN |
| chr10:89,711,233-89,713,016 | q23.31 | Allelic Imbalance | 1783 | 38.10 | 8.89 | 29.21 | 0.00 | 0.03 | PTEN |
| chr10:96,511,605-96,547,816 | q23.33 | Allelic Imbalance | 36211 | 38.10 | 8.89 | 29.21 | 0.00 | 0.03 | CYP2C19 |
| chr7:140,334,164-140,360,276 | q34 | Allelic Imbalance | 26112 | 38.10 | 8.89 | 29.21 | 0.00 | 0.03 | MRPS33 |
| chr7:55,197,966-55,199,037 | p11.2 | Allelic Imbalance | 1071 | 38.10 | 8.89 | 29.21 | 0.00 | 0.03 | EGFR |
| chr7:88,146,286-88,150,427 | q21.13 | Allelic Imbalance | 4141 | 38.10 | 8.89 | 29.21 | 0.00 | 0.03 |  |
| chr8:124,001,143-124,057,228 | q24.13 | Allelic Imbalance | 56085 | 4.76 | 31.11 | -26.35 | 0.00 | 0.03 | ZHX2 |
| chr8:125,019,184-125,108,223 | q24.13 | Allelic Imbalance | 89039 | 4.76 | 31.11 | -26.35 | 0.00 | 0.03 | FER1L6, FER1L6-AS1 |
| chr8:125,665,275-125,708,784 | q24.13 | Allelic Imbalance | 43509 | 4.76 | 31.11 | -26.35 | 0.00 | 0.03 | MTSS1 |
| chr8:125,752,243-125,756,300 | q24.13 | Allelic Imbalance | 4057 | 4.76 | 31.11 | -26.35 | 0.00 | 0.03 | MTSS1 |
| chr8:128,554,995-128,821,052 | q24.21 | Allelic Imbalance | 266057 | 4.76 | 31.11 | -26.35 | 0.00 | 0.03 | LOC727677, MYC |
| chr8:129,724,229-129,988,019 | q24.21 | Allelic Imbalance | 263790 | 4.76 | 31.11 | -26.35 | 0.00 | 0.03 |  |
| chr8:130,412,723-130,511,727 | q24.21 | Allelic Imbalance | 99004 | 4.76 | 31.11 | -26.35 | 0.00 | 0.03 |  |
| chr8:130,559,583-130,786,268 | q24.21 | Allelic Imbalance | 226685 | 4.76 | 31.11 | -26.35 | 0.00 | 0.03 |  |
| chr8:132,226,207-132,495,828 | q24.22 | Allelic Imbalance | 269621 | 4.76 | 31.11 | -26.35 | 0.00 | 0.03 |  |
| chr8:132,679,867-132,868,217 | q24.22 | Allelic Imbalance | 188350 | 4.76 | 31.11 | -26.35 | 0.00 | 0.03 |  |
| chr8:132,938,492-133,087,722 | q24.22 | Allelic Imbalance | 149230 | 4.76 | 31.11 | -26.35 | 0.00 | 0.03 | EFR3A |
| chr8:133,303,383-133,419,796 | q24.22 | Allelic Imbalance | 116413 | 4.76 | 31.11 | -26.35 | 0.00 | 0.03 | KCNQ3 |
| chr7:102,942,010-102,943,103 | q22.1 | Allelic Imbalance | 1093 | 52.38 | 20.00 | 32.38 | 0.00 | 0.03 | RELN |
| chr7:102,996,784-103,016,394 | q22.1 | Allelic Imbalance | 19610 | 52.38 | 20.00 | 32.38 | 0.00 | 0.03 | RELN |
| chr7:103,577,645-103,600,746 | q22.1 | Allelic Imbalance | 23101 | 52.38 | 20.00 | 32.38 | 0.00 | 0.03 | ORC5 |
| chr7:150,795,792-150,796,233 | q36.1 | Allelic Imbalance | 441 | 52.38 | 20.00 | 32.38 | 0.00 | 0.03 | RHEB |
| chr7:31,887,548-31,895,460 | p14.3 | Allelic Imbalance | 7912 | 52.38 | 20.00 | 32.38 | 0.00 | 0.03 | PDE1C |
| chr7:69,471,597-69,568,964 | q11.22 | Allelic Imbalance | 97367 | 52.38 | 20.00 | 32.38 | 0.00 | 0.03 | AUTS2 |
| chr7:94,622,324-94,676,864 | q21.3 | Allelic Imbalance | 54540 | 52.38 | 20.00 | 32.38 | 0.00 | 0.03 | PPP1R9A |
| chr7:103,431,256-103,434,275 | q22.1 | Allelic Imbalance | 3019 | 54.76 | 22.22 | 32.54 | 0.00 | 0.03 |  |
| chr7:103,790,821-103,791,118 | q22.1 | Allelic Imbalance | 297 | 54.76 | 22.22 | 32.54 | 0.00 | 0.03 | LHFPL3 |
| chr7:107,522,004-107,574,759 | q31.1 | Allelic Imbalance | 52755 | 54.76 | 22.22 | 32.54 | 0.00 | 0.03 | LAMB4 |
| chr7:107,717,075-107,730,892 | q31.1 | Allelic Imbalance | 13817 | 54.76 | 22.22 | 32.54 | 0.00 | 0.03 | NRCAM |
| chr7:114,123,978-114,158,774 | q31.1 | Allelic Imbalance | 34796 | 54.76 | 22.22 | 32.54 | 0.00 | 0.03 |  |
| chr7:114,206,651-114,259,496 | q31.1 | Allelic Imbalance | 52845 | 54.76 | 22.22 | 32.54 | 0.00 | 0.03 |  |
| chr7:114,311,926-114,367,107 | q31.1 | Allelic Imbalance | 55181 | 54.76 | 22.22 | 32.54 | 0.00 | 0.03 | MDFIC |
| chr7:150,695,580-150,761,906 | q36.1 | Allelic Imbalance | 66326 | 54.76 | 22.22 | 32.54 | 0.00 | 0.03 | NUB1, WDR86, WDR86-AS1, MIR3907, CRYGN |
| chr7:150,804,098-150,815,688 | q36.1 | Allelic Imbalance | 11590 | 54.76 | 22.22 | 32.54 | 0.00 | 0.03 | RHEB |
| chr7:75,325,708-75,341,632 | q11.23 | Allelic Imbalance | 15924 | 54.76 | 22.22 | 32.54 | 0.00 | 0.03 |  |
| chr7:83,808,043-83,827,146 | q21.11 | Allelic Imbalance | 19103 | 54.76 | 22.22 | 32.54 | 0.00 | 0.03 |  |
| chr7:84,467,763-84,546,179 | q21.11 | Allelic Imbalance | 78416 | 54.76 | 22.22 | 32.54 | 0.00 | 0.03 | SEMA3D |
| chr7:84,548,889-84,619,151 | q21.11 | Allelic Imbalance | 70262 | 54.76 | 22.22 | 32.54 | 0.00 | 0.03 | SEMA3D |
| chr7:84,944,015-84,969,738 | q21.11 | Allelic Imbalance | 25723 | 54.76 | 22.22 | 32.54 | 0.00 | 0.03 |  |
| chr7:88,029,584-88,036,995 | q21.13 | Allelic Imbalance | 7411 | 54.76 | 22.22 | 32.54 | 0.00 | 0.03 |  |
| chr7:93,855,940-93,858,639 | q21.3 | Allelic Imbalance | 2699 | 54.76 | 22.22 | 32.54 | 0.00 | 0.03 |  |
| chr7:93,897,212-93,897,413 | q21.3 | Allelic Imbalance | 201 | 54.76 | 22.22 | 32.54 | 0.00 | 0.03 | COL1A2 |
| chr7:94,232,111-94,246,181 | q21.3 | Allelic Imbalance | 14070 | 54.76 | 22.22 | 32.54 | 0.00 | 0.03 |  |
| chr7:98,249,602-98,255,696 | q22.1 | Allelic Imbalance | 6094 | 54.76 | 22.22 | 32.54 | 0.00 | 0.03 |  |
| chr10:103,296,940-103,307,503 | q24.32 | Allelic Imbalance | 10563 | 47.62 | 15.56 | 32.06 | 0.00 | 0.04 | BTRC |
| chr10:103,547,646-103,576,944 | q24.32 | Allelic Imbalance | 29298 | 47.62 | 15.56 | 32.06 | 0.00 | 0.04 | MGEA5, LOC100289509, KCNIP2 |
| chr10:131,441,030-131,556,396 | q26.3 | Allelic Imbalance | 115366 | 47.62 | 15.56 | 32.06 | 0.00 | 0.04 | MGMT, MIR4297, EBF3 |
| chr10:131,721,972-131,794,751 | q26.3 | Allelic Imbalance | 72779 | 47.62 | 15.56 | 32.06 | 0.00 | 0.04 | LOC387723, CTAGE7P |
| chr10:131,795,666-131,822,864 | q26.3 | Allelic Imbalance | 27198 | 47.62 | 15.56 | 32.06 | 0.00 | 0.04 | LOC387723, CTAGE7P |
| chr10:67,568,465-67,574,231 | q21.3 | Allelic Imbalance | 5766 | 47.62 | 15.56 | 32.06 | 0.00 | 0.04 | CTNNA3 |
| chr10:95,014,971-95,172,840 | q23.33 | Allelic Imbalance | 157869 | 47.62 | 15.56 | 32.06 | 0.00 | 0.04 | MYOF |
| chr7:30,597,136-30,688,920 | p15.1 | Allelic Imbalance | 91784 | 47.62 | 15.56 | 32.06 | 0.00 | 0.04 | GARS, CRHR2 |
| chr7:69,132,124-69,406,761 | q11.22 | Allelic Imbalance | 274637 | 47.62 | 15.56 | 32.06 | 0.00 | 0.04 | AUTS2 |
| chr7:75,771,996-75,783,498 | q11.23 | Allelic Imbalance | 11502 | 47.62 | 15.56 | 32.06 | 0.00 | 0.04 |  |
| chr7:86,641,092-86,697,939 | q21.12 | Allelic Imbalance | 56847 | 47.62 | 15.56 | 32.06 | 0.00 | 0.04 | DMTF1, TMEM243 |
| chr7:86,720,725-86,769,442 | q21.12 | Allelic Imbalance | 48717 | 47.62 | 15.56 | 32.06 | 0.00 | 0.04 |  |
| chr7:92,093,374-92,114,571 | q21.2 | Allelic Imbalance | 21197 | 47.62 | 15.56 | 32.06 | 0.00 | 0.04 | CDK6 |
| chr7:103,329,927-103,335,665 | q22.1 | Allelic Imbalance | 5738 | 57.14 | 24.44 | 32.70 | 0.00 | 0.04 | RELN |
| chr7:103,390,319-103,428,947 | q22.1 | Allelic Imbalance | 38628 | 57.14 | 24.44 | 32.70 | 0.00 | 0.04 | RELN |
| chr7:103,797,428-103,839,707 | q22.1 | Allelic Imbalance | 42279 | 57.14 | 24.44 | 32.70 | 0.00 | 0.04 | LHFPL3 |
| chr7:103,851,278-103,860,120 | q22.1 | Allelic Imbalance | 8842 | 57.14 | 24.44 | 32.70 | 0.00 | 0.04 | LHFPL3 |
| chr7:104,209,127-104,213,522 | q22.1 | Allelic Imbalance | 4395 | 57.14 | 24.44 | 32.70 | 0.00 | 0.04 | LHFPL3 |
| chr7:113,970,300-114,030,377 | q31.1 | Allelic Imbalance | 60077 | 57.14 | 24.44 | 32.70 | 0.00 | 0.04 | FOXP2 |
| chr7:114,079,539-114,086,123 | q31.1 | Allelic Imbalance | 6584 | 57.14 | 24.44 | 32.70 | 0.00 | 0.04 | FOXP2, MIR3666 |
| chr7:118,181,639-118,391,551 | q31.31 | Allelic Imbalance | 209912 | 57.14 | 24.44 | 32.70 | 0.00 | 0.04 |  |
| chr7:120,317,858-120,362,749 | q31.31 | Allelic Imbalance | 44891 | 57.14 | 24.44 | 32.70 | 0.00 | 0.04 |  |
| chr7:126,433,327-126,434,123 | q31.33 | Allelic Imbalance | 796 | 57.14 | 24.44 | 32.70 | 0.00 | 0.04 | GRM8 |
| chr7:126,584,846-126,602,381 | q31.33 | Allelic Imbalance | 17535 | 57.14 | 24.44 | 32.70 | 0.00 | 0.04 | GRM8 |
| chr7:137,925,410-137,945,778 | q34 | Allelic Imbalance | 20368 | 73.81 | 40.00 | 33.81 | 0.00 | 0.04 | SVOPL |
| chr7:139,806,629-139,823,246 | q34 | Allelic Imbalance | 16617 | 57.14 | 24.44 | 32.70 | 0.00 | 0.04 | MKRN1 |
| chr7:150,689,292-150,695,580 | q36.1 | Allelic Imbalance | 6288 | 57.14 | 24.44 | 32.70 | 0.00 | 0.04 | NUB1 |
| chr7:31,815,579-31,835,444 | p14.3 | Allelic Imbalance | 19865 | 57.14 | 24.44 | 32.70 | 0.00 | 0.04 | PDE1C |
| chr7:72,687,607-72,721,570 | q11.23 | Allelic Imbalance | 33963 | 57.14 | 24.44 | 32.70 | 0.00 | 0.04 | VPS37D |
| chr7:78,683,155-78,893,151 | q21.11 | Allelic Imbalance | 209996 | 57.14 | 24.44 | 32.70 | 0.00 | 0.04 | MAGI2 |
| chr7:78,901,058-78,914,336 | q21.11 | Allelic Imbalance | 13278 | 57.14 | 24.44 | 32.70 | 0.00 | 0.04 | MAGI2 |
| chr7:79,196,647-79,569,244 | q21.11 | Allelic Imbalance | 372597 | 57.14 | 24.44 | 32.70 | 0.00 | 0.04 |  |
| chr7:79,597,607-79,604,310 | q21.11 | Allelic Imbalance | 6703 | 57.14 | 24.44 | 32.70 | 0.00 | 0.04 | GNAI1 |
| chr7:83,506,271-83,627,028 | q21.11 | Allelic Imbalance | 120757 | 57.14 | 24.44 | 32.70 | 0.00 | 0.04 | SEMA3A |
| chr7:83,742,659-83,787,452 | q21.11 | Allelic Imbalance | 44793 | 57.14 | 24.44 | 32.70 | 0.00 | 0.04 |  |
| chr7:85,019,008-85,271,280 | q21.11 | Allelic Imbalance | 252272 | 57.14 | 24.44 | 32.70 | 0.00 | 0.04 |  |
| chr7:85,280,118-85,415,160 | q21.11 | Allelic Imbalance | 135042 | 57.14 | 24.44 | 32.70 | 0.00 | 0.04 |  |
| chr7:87,923,595-87,937,319 | q21.12 | Allelic Imbalance | 13724 | 57.14 | 24.44 | 32.70 | 0.00 | 0.04 |  |
| chr7:90,112,221-90,146,948 | q21.13 | Allelic Imbalance | 34727 | 57.14 | 24.44 | 32.70 | 0.00 | 0.04 |  |
| chr7:93,858,639-93,865,673 | q21.3 | Allelic Imbalance | 7034 | 57.14 | 24.44 | 32.70 | 0.00 | 0.04 | COL1A2 |
| chr7:94,923,934-94,939,244 | q21.3 | Allelic Imbalance | 15310 | 57.14 | 24.44 | 32.70 | 0.00 | 0.04 |  |
| chr10:100,484,754-100,520,969 | q24.2 | Allelic Imbalance | 36215 | 40.48 | 11.11 | 29.37 | 0.00 | 0.04 | HPSE2 |
| chr10:100,555,840-100,607,639 | q24.2 | Allelic Imbalance | 51799 | 40.48 | 11.11 | 29.37 | 0.00 | 0.04 | HPSE2 |
| chr10:111,672,784-111,845,768 | q25.1 - q25.2 | Allelic Imbalance | 172984 | 40.48 | 11.11 | 29.37 | 0.00 | 0.04 | XPNPEP1, LOC100505933, ADD3 |
| chr10:111,867,071-111,919,277 | q25.2 | Allelic Imbalance | 52206 | 40.48 | 11.11 | 29.37 | 0.00 | 0.04 | ADD3 |
| chr10:112,114,718-112,172,647 | q25.2 | Allelic Imbalance | 57929 | 40.48 | 11.11 | 29.37 | 0.00 | 0.04 |  |
| chr10:112,475,982-112,481,854 | q25.2 | Allelic Imbalance | 5872 | 40.48 | 11.11 | 29.37 | 0.00 | 0.04 | RBM20 |
| chr10:112,531,964-112,561,340 | q25.2 | Allelic Imbalance | 29376 | 40.48 | 11.11 | 29.37 | 0.00 | 0.04 | RBM20 |
| chr10:42,384,852-42,525,983 | q11.21 | Allelic Imbalance | 141131 | 40.48 | 11.11 | 29.37 | 0.00 | 0.04 | ZNF33B |
| chr10:43,398,120-43,561,309 | q11.21 | Allelic Imbalance | 163189 | 40.48 | 11.11 | 29.37 | 0.00 | 0.04 | ZNF485, ZNF32-AS1, ZNF32, ZNF32-AS2, ZNF32-AS3 |
| chr10:51,802,386-51,831,324 | q11.23 | Allelic Imbalance | 28938 | 40.48 | 11.11 | 29.37 | 0.00 | 0.04 | SGMS1 |
| chr10:52,017,130-52,050,261 | q11.23 | Allelic Imbalance | 33131 | 40.48 | 11.11 | 29.37 | 0.00 | 0.04 | SGMS1 |
| chr10:52,180,315-52,313,114 | q11.23 | Allelic Imbalance | 132799 | 40.48 | 11.11 | 29.37 | 0.00 | 0.04 | ASAH2B, A1CF |
| chr10:52,320,142-52,406,217 | q11.23 | Allelic Imbalance | 86075 | 40.48 | 11.11 | 29.37 | 0.00 | 0.04 |  |
| chr10:52,804,027-52,816,873 | q11.23 | Allelic Imbalance | 12846 | 40.48 | 11.11 | 29.37 | 0.00 | 0.04 | PRKG1 |
| chr10:52,899,243-52,907,481 | q11.23 | Allelic Imbalance | 8238 | 40.48 | 11.11 | 29.37 | 0.00 | 0.04 | PRKG1 |
| chr10:56,679,264-57,081,983 | q21.1 | Allelic Imbalance | 402719 | 40.48 | 11.11 | 29.37 | 0.00 | 0.04 | RNU6-59, MTRNR2L5 |
| chr10:61,411,501-61,462,237 | q21.2 | Allelic Imbalance | 50736 | 40.48 | 11.11 | 29.37 | 0.00 | 0.04 | ANK3 |
| chr10:61,681,807-61,710,220 | q21.2 | Allelic Imbalance | 28413 | 40.48 | 11.11 | 29.37 | 0.00 | 0.04 | ANK3 |
| chr10:61,735,036-61,910,178 | q21.2 | Allelic Imbalance | 175142 | 40.48 | 11.11 | 29.37 | 0.00 | 0.04 | ANK3 |
| chr10:62,214,824-62,233,965 | q21.2 | Allelic Imbalance | 19141 | 40.48 | 11.11 | 29.37 | 0.00 | 0.04 | CDK1 |
| chr10:66,626,581-66,762,961 | q21.3 | Allelic Imbalance | 136380 | 40.48 | 11.11 | 29.37 | 0.00 | 0.04 |  |
| chr10:66,766,562-66,862,492 | q21.3 | Allelic Imbalance | 95930 | 40.48 | 11.11 | 29.37 | 0.00 | 0.04 |  |
| chr10:67,166,055-67,169,251 | q21.3 | Allelic Imbalance | 3196 | 40.48 | 11.11 | 29.37 | 0.00 | 0.04 |  |
| chr10:67,295,121-67,296,581 | q21.3 | Allelic Imbalance | 1460 | 40.48 | 11.11 | 29.37 | 0.00 | 0.04 |  |
| chr10:69,578,883-69,582,053 | q21.3 | Allelic Imbalance | 3170 | 40.48 | 11.11 | 29.37 | 0.00 | 0.04 | MYPN |
| chr10:69,589,110-69,633,336 | q21.3 | Allelic Imbalance | 44226 | 40.48 | 11.11 | 29.37 | 0.00 | 0.04 | MYPN |
| chr10:71,717,229-71,783,052 | q22.1 | Allelic Imbalance | 65823 | 40.48 | 11.11 | 29.37 | 0.00 | 0.04 | LRRC20 |
| chr10:71,954,470-71,979,164 | q22.1 | Allelic Imbalance | 24694 | 40.48 | 11.11 | 29.37 | 0.00 | 0.04 | PALD1 |
| chr10:78,840,531-78,850,717 | q22.3 | Allelic Imbalance | 10186 | 40.48 | 11.11 | 29.37 | 0.00 | 0.04 | KCNMA1 |
| chr10:89,635,200-89,635,430 | q23.31 | Allelic Imbalance | 230 | 33.33 | 6.67 | 26.67 | 0.00 | 0.04 | PTEN |
| chr10:89,694,536-89,696,558 | q23.31 | Allelic Imbalance | 2022 | 33.33 | 6.67 | 26.67 | 0.00 | 0.04 | PTEN |
| chr10:94,483,669-94,515,569 | q23.33 | Allelic Imbalance | 31900 | 40.48 | 11.11 | 29.37 | 0.00 | 0.04 |  |
| chr10:96,377,369-96,459,916 | q23.33 | Allelic Imbalance | 82547 | 40.48 | 11.11 | 29.37 | 0.00 | 0.04 | CYP2C18 |
| chr12:2,987,189-3,028,927 | p13.33 | Allelic Imbalance | 41738 | 9.52 | 37.78 | -28.25 | 0.00 | 0.04 | TEAD4 |
| chr12:3,068,100-3,076,093 | p13.33 | Allelic Imbalance | 7993 | 9.52 | 37.78 | -28.25 | 0.00 | 0.04 | TSPAN9 |
| chr12:3,107,317-3,111,203 | p13.32 | Allelic Imbalance | 3886 | 9.52 | 37.78 | -28.25 | 0.00 | 0.04 | TSPAN9 |
| chr12:3,206,739-3,221,490 | p13.32 | Allelic Imbalance | 14751 | 9.52 | 37.78 | -28.25 | 0.00 | 0.04 | TSPAN9 |
| chr14:43,437,249-43,586,361 | q21.3 | Allelic Imbalance | 149112 | 33.33 | 6.67 | 26.67 | 0.00 | 0.04 |  |
| chr7:101,098,055-101,136,751 | q22.1 | Allelic Imbalance | 38696 | 64.29 | 31.11 | 33.17 | 0.00 | 0.04 |  |
| chr7:103,374,174-103,376,974 | q22.1 | Allelic Imbalance | 2800 | 59.52 | 26.67 | 32.86 | 0.00 | 0.04 | RELN |
| chr7:103,380,416-103,384,607 | q22.1 | Allelic Imbalance | 4191 | 59.52 | 26.67 | 32.86 | 0.00 | 0.04 | RELN |
| chr7:103,873,345-103,879,808 | q22.1 | Allelic Imbalance | 6463 | 59.52 | 26.67 | 32.86 | 0.00 | 0.04 | LHFPL3 |
| chr7:103,893,566-103,921,146 | q22.1 | Allelic Imbalance | 27580 | 59.52 | 26.67 | 32.86 | 0.00 | 0.04 | LHFPL3 |
| chr7:103,942,302-103,945,624 | q22.1 | Allelic Imbalance | 3322 | 59.52 | 26.67 | 32.86 | 0.00 | 0.04 | LHFPL3 |
| chr7:105,184,070-105,258,774 | q22.2 | Allelic Imbalance | 74704 | 64.29 | 31.11 | 33.17 | 0.00 | 0.04 | ATXN7L1 |
| chr7:105,359,608-105,362,959 | q22.2 | Allelic Imbalance | 3351 | 66.67 | 33.33 | 33.33 | 0.00 | 0.04 |  |
| chr7:105,410,120-105,428,092 | q22.2 | Allelic Imbalance | 17972 | 69.05 | 35.56 | 33.49 | 0.00 | 0.04 | CDHR3 |
| chr7:105,570,826-105,586,168 | q22.2 | Allelic Imbalance | 15342 | 66.67 | 33.33 | 33.33 | 0.00 | 0.04 |  |
| chr7:107,108,926-107,112,019 | q22.3 | Allelic Imbalance | 3093 | 59.52 | 26.67 | 32.86 | 0.00 | 0.04 | SLC26A4 |
| chr7:107,242,125-107,257,228 | q31.1 | Allelic Imbalance | 15103 | 59.52 | 26.67 | 32.86 | 0.00 | 0.04 |  |
| chr7:107,264,464-107,284,180 | q31.1 | Allelic Imbalance | 19716 | 59.52 | 26.67 | 32.86 | 0.00 | 0.04 |  |
| chr7:107,294,554-107,303,481 | q31.1 | Allelic Imbalance | 8927 | 59.52 | 26.67 | 32.86 | 0.00 | 0.04 |  |
| chr7:107,896,915-107,903,477 | q31.1 | Allelic Imbalance | 6562 | 61.90 | 28.89 | 33.02 | 0.00 | 0.04 | PNPLA8 |
| chr7:109,101,165-109,286,617 | q31.1 | Allelic Imbalance | 185452 | 64.29 | 31.11 | 33.17 | 0.00 | 0.04 |  |
| chr7:109,400,568-109,431,217 | q31.1 | Allelic Imbalance | 30649 | 61.90 | 28.89 | 33.02 | 0.00 | 0.04 |  |
| chr7:109,606,386-109,679,396 | q31.1 | Allelic Imbalance | 73010 | 64.29 | 31.11 | 33.17 | 0.00 | 0.04 |  |
| chr7:109,718,984-109,730,896 | q31.1 | Allelic Imbalance | 11912 | 64.29 | 31.11 | 33.17 | 0.00 | 0.04 |  |
| chr7:110,058,837-110,104,915 | q31.1 | Allelic Imbalance | 46078 | 59.52 | 26.67 | 32.86 | 0.00 | 0.04 | IMMP2L |
| chr7:110,112,378-110,151,655 | q31.1 | Allelic Imbalance | 39277 | 59.52 | 26.67 | 32.86 | 0.00 | 0.04 | IMMP2L |
| chr7:111,047,341-111,105,333 | q31.1 | Allelic Imbalance | 57992 | 59.52 | 26.67 | 32.86 | 0.00 | 0.04 |  |
| chr7:111,275,977-111,340,063 | q31.1 | Allelic Imbalance | 64086 | 61.90 | 28.89 | 33.02 | 0.00 | 0.04 | DOCK4 |
| chr7:111,500,003-111,505,799 | q31.1 | Allelic Imbalance | 5796 | 59.52 | 26.67 | 32.86 | 0.00 | 0.04 | DOCK4 |
| chr7:112,836,424-112,843,468 | q31.1 | Allelic Imbalance | 7044 | 59.52 | 26.67 | 32.86 | 0.00 | 0.04 |  |
| chr7:112,875,707-112,881,911 | q31.1 | Allelic Imbalance | 6204 | 59.52 | 26.67 | 32.86 | 0.00 | 0.04 |  |
| chr7:112,886,751-112,911,538 | q31.1 | Allelic Imbalance | 24787 | 59.52 | 26.67 | 32.86 | 0.00 | 0.04 |  |
| chr7:113,525,692-113,539,425 | q31.1 | Allelic Imbalance | 13733 | 61.90 | 28.89 | 33.02 | 0.00 | 0.04 | FOXP2 |
| chr7:113,551,212-113,772,898 | q31.1 | Allelic Imbalance | 221686 | 61.90 | 28.89 | 33.02 | 0.00 | 0.04 | FOXP2 |
| chr7:114,492,505-114,519,221 | q31.2 | Allelic Imbalance | 26716 | 59.52 | 26.67 | 32.86 | 0.00 | 0.04 |  |
| chr7:116,350,060-116,356,927 | q31.2 | Allelic Imbalance | 6867 | 61.90 | 28.89 | 33.02 | 0.00 | 0.04 |  |
| chr7:120,961,996-121,007,160 | q31.32 | Allelic Imbalance | 45164 | 69.05 | 35.56 | 33.49 | 0.00 | 0.04 |  |
| chr7:121,559,175-121,609,051 | q31.32 | Allelic Imbalance | 49876 | 66.67 | 33.33 | 33.33 | 0.00 | 0.04 | AASS |
| chr7:121,777,022-121,881,815 | q31.32 | Allelic Imbalance | 104793 | 66.67 | 33.33 | 33.33 | 0.00 | 0.04 | CADPS2 |
| chr7:121,943,310-122,399,659 | q31.32 | Allelic Imbalance | 456349 | 64.29 | 31.11 | 33.17 | 0.00 | 0.04 | CADPS2, RNF133, RNF148 |
| chr7:124,351,809-124,485,887 | q31.33 | Allelic Imbalance | 134078 | 61.90 | 28.89 | 33.02 | 0.00 | 0.04 | POT1 |
| chr7:124,561,482-124,601,344 | q31.33 | Allelic Imbalance | 39862 | 61.90 | 28.89 | 33.02 | 0.00 | 0.04 |  |
| chr7:125,395,600-125,737,125 | q31.33 | Allelic Imbalance | 341525 | 66.67 | 33.33 | 33.33 | 0.00 | 0.04 |  |
| chr7:126,364,181-126,423,517 | q31.33 | Allelic Imbalance | 59336 | 59.52 | 26.67 | 32.86 | 0.00 | 0.04 | GRM8 |
| chr7:126,634,889-126,644,433 | q31.33 | Allelic Imbalance | 9544 | 61.90 | 28.89 | 33.02 | 0.00 | 0.04 | GRM8 |
| chr7:126,644,433-126,661,477 | q31.33 | Allelic Imbalance | 17044 | 64.29 | 31.11 | 33.17 | 0.00 | 0.04 | GRM8 |
| chr7:127,562,661-127,630,406 | q32.1 | Allelic Imbalance | 67745 | 66.67 | 33.33 | 33.33 | 0.00 | 0.04 |  |
| chr7:127,679,814-127,704,829 | q32.1 | Allelic Imbalance | 25015 | 69.05 | 35.56 | 33.49 | 0.00 | 0.04 | LEP |
| chr7:127,704,829-127,768,638 | q32.1 | Allelic Imbalance | 63809 | 71.43 | 37.78 | 33.65 | 0.00 | 0.04 | MGC27345, RBM28 |
| chr7:134,283,526-134,292,215 | q33 | Allelic Imbalance | 8689 | 64.29 | 31.11 | 33.17 | 0.00 | 0.04 | CALD1 |
| chr7:134,301,472-134,526,248 | q33 | Allelic Imbalance | 224776 | 64.29 | 31.11 | 33.17 | 0.00 | 0.04 | CALD1, AGBL3, TMEM140, C7orf49, WDR91 |
| chr7:136,352,529-136,353,250 | q33 | Allelic Imbalance | 721 | 64.29 | 31.11 | 33.17 | 0.00 | 0.04 | LOC349160 |
| chr7:136,485,324-136,590,356 | q33 | Allelic Imbalance | 105032 | 66.67 | 33.33 | 33.33 | 0.00 | 0.04 | LOC349160, PTN |
| chr7:138,819,404-138,831,501 | q34 | Allelic Imbalance | 12097 | 71.43 | 37.78 | 33.65 | 0.00 | 0.04 |  |
| chr7:138,846,439-138,980,276 | q34 | Allelic Imbalance | 133837 | 71.43 | 37.78 | 33.65 | 0.00 | 0.04 | CLEC2L, HIPK2 |
| chr7:138,983,209-138,985,366 | q34 | Allelic Imbalance | 2157 | 71.43 | 37.78 | 33.65 | 0.00 | 0.04 | HIPK2 |
| chr7:139,128,363-139,168,996 | q34 | Allelic Imbalance | 40633 | 59.52 | 26.67 | 32.86 | 0.00 | 0.04 | TBXAS1 |
| chr7:139,171,571-139,192,491 | q34 | Allelic Imbalance | 20920 | 61.90 | 28.89 | 33.02 | 0.00 | 0.04 | TBXAS1 |
| chr7:139,204,873-139,225,837 | q34 | Allelic Imbalance | 20964 | 61.90 | 28.89 | 33.02 | 0.00 | 0.04 | TBXAS1 |
| chr7:140,426,097-140,427,004 | q34 | Allelic Imbalance | 907 | 40.48 | 11.11 | 29.37 | 0.00 | 0.04 | TMEM178B |
| chr7:140,600,041-140,640,244 | q34 | Allelic Imbalance | 40203 | 64.29 | 31.11 | 33.17 | 0.00 | 0.04 | TMEM178B |
| chr7:140,684,738-140,747,456 | q34 | Allelic Imbalance | 62718 | 61.90 | 28.89 | 33.02 | 0.00 | 0.04 | TMEM178B |
| chr7:143,722,670-143,770,590 | q35 | Allelic Imbalance | 47920 | 69.05 | 35.56 | 33.49 | 0.00 | 0.04 | NOBOX |
| chr7:146,197,766-146,208,878 | q35 | Allelic Imbalance | 11112 | 66.67 | 33.33 | 33.33 | 0.00 | 0.04 | CNTNAP2 |
| chr7:146,231,477-146,281,873 | q35 | Allelic Imbalance | 50396 | 66.67 | 33.33 | 33.33 | 0.00 | 0.04 | MIR548AQ, MIR548AR, CNTNAP2 |
| chr7:147,200,573-147,214,744 | q35 | Allelic Imbalance | 14171 | 66.67 | 33.33 | 33.33 | 0.00 | 0.04 | CNTNAP2 |
| chr7:147,889,037-147,941,779 | q36.1 | Allelic Imbalance | 52742 | 64.29 | 31.11 | 33.17 | 0.00 | 0.04 | C7orf33 |
| chr7:147,957,162-147,982,869 | q36.1 | Allelic Imbalance | 25707 | 66.67 | 33.33 | 33.33 | 0.00 | 0.04 |  |
| chr7:148,672,644-148,684,981 | q36.1 | Allelic Imbalance | 12337 | 66.67 | 33.33 | 33.33 | 0.00 | 0.04 |  |
| chr7:148,971,747-149,060,451 | q36.1 | Allelic Imbalance | 88704 | 66.67 | 33.33 | 33.33 | 0.00 | 0.04 | KRBA1 |
| chr7:149,378,035-149,773,216 | q36.1 | Allelic Imbalance | 395181 | 66.67 | 33.33 | 33.33 | 0.00 | 0.04 | ACTR3C, LRRC61, C7orf29, RARRES2, RNU6-33, RNU6-34, REPIN1, ZNF775, LOC728743, LOC285972 |
| chr7:149,793,802-149,844,348 | q36.1 | Allelic Imbalance | 50546 | 64.29 | 31.11 | 33.17 | 0.00 | 0.04 | GIMAP8, GIMAP7 |
| chr7:149,896,345-149,899,525 | q36.1 | Allelic Imbalance | 3180 | 64.29 | 31.11 | 33.17 | 0.00 | 0.04 | GIMAP4 |
| chr7:150,394,158-150,457,748 | q36.1 | Allelic Imbalance | 63590 | 64.29 | 31.11 | 33.17 | 0.00 | 0.04 | SLC4A2, FASTK, TMUB1, AGAP3 |
| chr7:150,507,569-150,535,334 | q36.1 | Allelic Imbalance | 27765 | 64.29 | 31.11 | 33.17 | 0.00 | 0.04 | ASB10 |
| chr7:150,585,156-150,642,870 | q36.1 | Allelic Imbalance | 57714 | 59.52 | 26.67 | 32.86 | 0.00 | 0.04 | SMARCD3 |
| chr7:151,075,138-151,110,424 | q36.1 | Allelic Imbalance | 35286 | 61.90 | 28.89 | 33.02 | 0.00 | 0.04 | PRKAG2 |
| chr7:151,899,796-151,981,357 | q36.1 | Allelic Imbalance | 81561 | 64.29 | 31.11 | 33.17 | 0.00 | 0.04 | XRCC2 |
| chr7:152,016,553-152,236,827 | q36.1 - q36.2 | Allelic Imbalance | 220274 | 69.05 | 35.56 | 33.49 | 0.00 | 0.04 | ACTR3B |
| chr7:152,918,723-152,944,313 | q36.2 | Allelic Imbalance | 25590 | 66.67 | 33.33 | 33.33 | 0.00 | 0.04 |  |
| chr7:153,270,294-153,284,083 | q36.2 | Allelic Imbalance | 13789 | 66.67 | 33.33 | 33.33 | 0.00 | 0.04 | DPP6 |
| chr7:153,602,196-153,662,831 | q36.2 | Allelic Imbalance | 60635 | 64.29 | 31.11 | 33.17 | 0.00 | 0.04 | DPP6 |
| chr7:153,692,954-153,742,234 | q36.2 | Allelic Imbalance | 49280 | 64.29 | 31.11 | 33.17 | 0.00 | 0.04 | DPP6 |
| chr7:153,792,967-153,808,274 | q36.2 | Allelic Imbalance | 15307 | 66.67 | 33.33 | 33.33 | 0.00 | 0.04 | DPP6 |
| chr7:154,346,684-154,357,439 | q36.2 | Allelic Imbalance | 10755 | 64.29 | 31.11 | 33.17 | 0.00 | 0.04 | LOC100132707 |
| chr7:154,494,920-154,539,127 | q36.2 | Allelic Imbalance | 44207 | 64.29 | 31.11 | 33.17 | 0.00 | 0.04 | HTR5A |
| chr7:154,561,496-154,597,596 | q36.2 | Allelic Imbalance | 36100 | 66.67 | 33.33 | 33.33 | 0.00 | 0.04 |  |
| chr7:154,805,838-155,063,225 | q36.3 | Allelic Imbalance | 257387 | 66.67 | 33.33 | 33.33 | 0.00 | 0.04 | EN2, CNPY1 |
| chr7:155,127,582-155,332,517 | q36.3 | Allelic Imbalance | 204935 | 66.67 | 33.33 | 33.33 | 0.00 | 0.04 | RBM33, SHH |
| chr7:156,307,650-156,374,795 | q36.3 | Allelic Imbalance | 67145 | 64.29 | 31.11 | 33.17 | 0.00 | 0.04 | LMBR1 |
| chr7:156,503,804-156,638,258 | q36.3 | Allelic Imbalance | 134454 | 64.29 | 31.11 | 33.17 | 0.00 | 0.04 | UBE3C |
| chr7:157,110,366-157,142,167 | q36.3 | Allelic Imbalance | 31801 | 66.67 | 33.33 | 33.33 | 0.00 | 0.04 | PTPRN2 |
| chr7:157,483,415-157,546,039 | q36.3 | Allelic Imbalance | 62624 | 66.67 | 33.33 | 33.33 | 0.00 | 0.04 | PTPRN2 |
| chr7:157,754,963-157,876,238 | q36.3 | Allelic Imbalance | 121275 | 64.29 | 31.11 | 33.17 | 0.00 | 0.04 | PTPRN2 |
| chr7:55,139,473-55,147,471 | p11.2 | Allelic Imbalance | 7998 | 40.48 | 11.11 | 29.37 | 0.00 | 0.04 | EGFR |
| chr7:55,188,127-55,191,959 | p11.2 | Allelic Imbalance | 3832 | 40.48 | 11.11 | 29.37 | 0.00 | 0.04 | EGFR |
| chr7:55,212,053-55,219,961 | p11.2 | Allelic Imbalance | 7908 | 33.33 | 6.67 | 26.67 | 0.00 | 0.04 | EGFR, EGFR-AS1 |
| chr7:55,268,190-55,273,766 | p11.2 | Allelic Imbalance | 5576 | 33.33 | 6.67 | 26.67 | 0.00 | 0.04 |  |
| chr7:55,274,528-55,276,830 | p11.2 | Allelic Imbalance | 2302 | 33.33 | 6.67 | 26.67 | 0.00 | 0.04 |  |
| chr7:55,297,359-55,329,860 | p11.2 | Allelic Imbalance | 32501 | 40.48 | 11.11 | 29.37 | 0.00 | 0.04 |  |
| chr7:72,774,504-72,784,478 | q11.23 | Allelic Imbalance | 9974 | 59.52 | 26.67 | 32.86 | 0.00 | 0.04 |  |
| chr7:80,779,511-80,862,829 | q21.11 | Allelic Imbalance | 83318 | 59.52 | 26.67 | 32.86 | 0.00 | 0.04 |  |
| chr7:81,195,147-81,268,429 | q21.11 | Allelic Imbalance | 73282 | 59.52 | 26.67 | 32.86 | 0.00 | 0.04 | HGF |
| chr7:81,439,064-81,499,527 | q21.11 | Allelic Imbalance | 60463 | 59.52 | 26.67 | 32.86 | 0.00 | 0.04 | CACNA2D1 |
| chr7:81,532,314-81,536,857 | q21.11 | Allelic Imbalance | 4543 | 61.90 | 28.89 | 33.02 | 0.00 | 0.04 | CACNA2D1 |
| chr7:85,443,979-85,452,460 | q21.11 | Allelic Imbalance | 8481 | 59.52 | 26.67 | 32.86 | 0.00 | 0.04 |  |
| chr7:86,284,795-86,297,029 | q21.12 | Allelic Imbalance | 12234 | 59.52 | 26.67 | 32.86 | 0.00 | 0.04 | GRM3 |
| chr7:87,819,852-87,826,289 | q21.12 | Allelic Imbalance | 6437 | 59.52 | 26.67 | 32.86 | 0.00 | 0.04 |  |
| chr7:87,877,083-87,892,542 | q21.12 | Allelic Imbalance | 15459 | 59.52 | 26.67 | 32.86 | 0.00 | 0.04 |  |
| chr7:88,152,620-88,163,180 | q21.13 | Allelic Imbalance | 10560 | 33.33 | 6.67 | 26.67 | 0.00 | 0.04 |  |
| chr7:88,242,039-88,251,749 | q21.13 | Allelic Imbalance | 9710 | 33.33 | 6.67 | 26.67 | 0.00 | 0.04 | ZNF804B |
| chr7:88,388,900-88,390,994 | q21.13 | Allelic Imbalance | 2094 | 40.48 | 11.11 | 29.37 | 0.00 | 0.04 | ZNF804B |
| chr7:89,145,086-89,173,989 | q21.13 | Allelic Imbalance | 28903 | 59.52 | 26.67 | 32.86 | 0.00 | 0.04 |  |
| chr7:90,357,518-90,378,465 | q21.13 | Allelic Imbalance | 20947 | 61.90 | 28.89 | 33.02 | 0.00 | 0.04 | CDK14 |
| chr7:90,383,840-90,493,213 | q21.13 | Allelic Imbalance | 109373 | 64.29 | 31.11 | 33.17 | 0.00 | 0.04 | CDK14 |
| chr7:90,550,913-90,579,228 | q21.13 | Allelic Imbalance | 28315 | 64.29 | 31.11 | 33.17 | 0.00 | 0.04 | CDK14 |
| chr7:90,581,440-90,582,151 | q21.13 | Allelic Imbalance | 711 | 64.29 | 31.11 | 33.17 | 0.00 | 0.04 | CDK14 |
| chr7:90,945,816-90,951,735 | q21.2 | Allelic Imbalance | 5919 | 59.52 | 26.67 | 32.86 | 0.00 | 0.04 |  |
| chr7:92,151,211-92,170,827 | q21.2 | Allelic Imbalance | 19616 | 40.48 | 11.11 | 29.37 | 0.00 | 0.04 | CDK6 |
| chr7:92,194,800-92,213,218 | q21.2 | Allelic Imbalance | 18418 | 40.48 | 11.11 | 29.37 | 0.00 | 0.04 | CDK6 |
| chr7:92,967,713-92,971,255 | q21.3 | Allelic Imbalance | 3542 | 64.29 | 31.11 | 33.17 | 0.00 | 0.04 | CALCR |
| chr7:93,025,111-93,038,016 | q21.3 | Allelic Imbalance | 12905 | 61.90 | 28.89 | 33.02 | 0.00 | 0.04 | CALCR |
| chr7:93,514,853-93,522,239 | q21.3 | Allelic Imbalance | 7386 | 61.90 | 28.89 | 33.02 | 0.00 | 0.04 |  |
| chr7:93,876,785-93,884,544 | q21.3 | Allelic Imbalance | 7759 | 59.52 | 26.67 | 32.86 | 0.00 | 0.04 | COL1A2 |
| chr7:94,939,244-94,944,848 | q21.3 | Allelic Imbalance | 5604 | 59.52 | 26.67 | 32.86 | 0.00 | 0.04 |  |
| chr7:94,951,046-95,014,651 | q21.3 | Allelic Imbalance | 63605 | 59.52 | 26.67 | 32.86 | 0.00 | 0.04 | ASB4 |
| chr7:96,433,910-96,498,608 | q21.3 | Allelic Imbalance | 64698 | 69.05 | 35.56 | 33.49 | 0.00 | 0.04 | DLX6-AS1, DLX6, DLX5 |
| chr7:96,547,406-96,642,231 | q21.3 | Allelic Imbalance | 94825 | 66.67 | 33.33 | 33.33 | 0.00 | 0.04 | ACN9 |
| chr7:96,721,184-96,806,966 | q21.3 | Allelic Imbalance | 85782 | 66.67 | 33.33 | 33.33 | 0.00 | 0.04 |  |
| chr7:96,808,273-96,850,540 | q21.3 | Allelic Imbalance | 42267 | 66.67 | 33.33 | 33.33 | 0.00 | 0.04 |  |
| chr7:96,936,580-96,957,172 | q21.3 | Allelic Imbalance | 20592 | 66.67 | 33.33 | 33.33 | 0.00 | 0.04 |  |
| chr10:119,710,535-119,725,906 | q26.11 | Allelic Imbalance | 15371 | 50.00 | 17.78 | 32.22 | 0.00 | 0.04 |  |
| chr7:102,993,766-102,994,865 | q22.1 | Allelic Imbalance | 1099 | 50.00 | 17.78 | 32.22 | 0.00 | 0.04 | RELN |
| chr7:103,722,124-103,741,287 | q22.1 | Allelic Imbalance | 19163 | 50.00 | 17.78 | 32.22 | 0.00 | 0.04 |  |
| chr7:31,895,460-31,922,867 | p14.3 | Allelic Imbalance | 27407 | 50.00 | 17.78 | 32.22 | 0.00 | 0.04 | PDE1C |
| chr7:31,925,320-31,955,926 | p14.3 | Allelic Imbalance | 30606 | 50.00 | 17.78 | 32.22 | 0.00 | 0.04 | PDE1C |
| chr7:68,423,461-68,546,404 | q11.22 | Allelic Imbalance | 122943 | 50.00 | 17.78 | 32.22 | 0.00 | 0.04 |  |
| chr7:86,707,577-86,720,725 | q21.12 | Allelic Imbalance | 13148 | 50.00 | 17.78 | 32.22 | 0.00 | 0.04 |  |
| chr7:94,455,390-94,482,458 | q21.3 | Allelic Imbalance | 27068 | 50.00 | 17.78 | 32.22 | 0.00 | 0.04 | PPP1R9A |
| chr12:103,563,294-103,626,127 | q23.3 | Allelic Imbalance | 62833 | 7.14 | 33.33 | -26.19 | 0.00 | 0.04 | CHST11 |
| chr12:2,376,278-2,400,836 | p13.33 | Allelic Imbalance | 24558 | 7.14 | 33.33 | -26.19 | 0.00 | 0.04 | CACNA1C |
| chr12:2,440,930-2,474,211 | p13.33 | Allelic Imbalance | 33281 | 7.14 | 33.33 | -26.19 | 0.00 | 0.04 | CACNA1C |
| chr12:2,493,272-2,557,034 | p13.33 | Allelic Imbalance | 63762 | 7.14 | 33.33 | -26.19 | 0.00 | 0.04 | CACNA1C |
| chr12:2,609,983-2,622,080 | p13.33 | Allelic Imbalance | 12097 | 7.14 | 33.33 | -26.19 | 0.00 | 0.04 | CACNA1C |
| chr12:69,859,683-69,909,961 | q21.1 | Allelic Imbalance | 50278 | 7.14 | 33.33 | -26.19 | 0.00 | 0.04 |  |
| chr12:80,172,424-80,274,682 | q21.31 | Allelic Imbalance | 102258 | 7.14 | 33.33 | -26.19 | 0.00 | 0.04 | ACSS3, PPFIA2 |
| chr12:80,607,659-80,724,940 | q21.31 | Allelic Imbalance | 117281 | 7.14 | 33.33 | -26.19 | 0.00 | 0.04 | PPFIA2 |
| chr12:94,942,811-94,978,170 | q23.1 | Allelic Imbalance | 35359 | 7.14 | 33.33 | -26.19 | 0.00 | 0.04 | LTA4H |
| chr8:124,661,921-124,856,286 | q24.13 | Allelic Imbalance | 194365 | 7.14 | 33.33 | -26.19 | 0.00 | 0.04 | KLHL38, ANXA13, FAM91A1 |
| chr8:125,567,515-125,572,223 | q24.13 | Allelic Imbalance | 4708 | 7.14 | 33.33 | -26.19 | 0.00 | 0.04 | RNF139, TATDN1 |
| chr8:133,492,508-133,559,237 | q24.22 | Allelic Imbalance | 66729 | 7.14 | 33.33 | -26.19 | 0.00 | 0.04 | KCNQ3 |
| chr10:110,711,333-111,324,857 | q25.1 | Allelic Imbalance | 613524 | 42.86 | 13.33 | 29.52 | 0.00 | 0.05 | RNU6-53 |
| chr10:114,878,155-114,899,092 | q25.2 | Allelic Imbalance | 20937 | 42.86 | 13.33 | 29.52 | 0.00 | 0.05 | TCF7L2 |
| chr10:115,017,620-115,140,144 | q25.3 | Allelic Imbalance | 122524 | 42.86 | 13.33 | 29.52 | 0.00 | 0.05 |  |
| chr10:123,127,548-123,134,793 | q26.13 | Allelic Imbalance | 7245 | 42.86 | 13.33 | 29.52 | 0.00 | 0.05 |  |
| chr10:42,740,266-42,805,486 | q11.21 | Allelic Imbalance | 65220 | 42.86 | 13.33 | 29.52 | 0.00 | 0.05 |  |
| chr10:42,944,475-43,076,205 | q11.21 | Allelic Imbalance | 131730 | 42.86 | 13.33 | 29.52 | 0.00 | 0.05 | RET, CSGALNACT2, RASGEF1A |
| chr10:47,328,768-47,955,419 | q11.22 | Allelic Imbalance | 626651 | 42.86 | 13.33 | 29.52 | 0.00 | 0.05 | FAM21B, CTSL1P2, BMS1P2, BMS1P6, AGAP9, FAM25B, FAM25C, FAM25G, ANXA8, ANXA8L1 |
| chr10:47,975,145-47,997,935 | q11.22 | Allelic Imbalance | 22790 | 42.86 | 13.33 | 29.52 | 0.00 | 0.05 | ZNF488 |
| chr10:48,182,070-48,249,890 | q11.22 | Allelic Imbalance | 67820 | 42.86 | 13.33 | 29.52 | 0.00 | 0.05 |  |
| chr10:49,066,938-49,101,158 | q11.22 | Allelic Imbalance | 34220 | 42.86 | 13.33 | 29.52 | 0.00 | 0.05 | FRMPD2 |
| chr10:55,041,700-55,057,502 | q21.1 | Allelic Imbalance | 15802 | 42.86 | 13.33 | 29.52 | 0.00 | 0.05 |  |
| chr10:66,254,389-66,309,752 | q21.3 | Allelic Imbalance | 55363 | 42.86 | 13.33 | 29.52 | 0.00 | 0.05 | ANXA2P3 |
| chr10:66,973,588-67,154,729 | q21.3 | Allelic Imbalance | 181141 | 42.86 | 13.33 | 29.52 | 0.00 | 0.05 |  |
| chr10:72,362,664-72,725,864 | q22.1 | Allelic Imbalance | 363200 | 42.86 | 13.33 | 29.52 | 0.00 | 0.05 | LOC728978, UNC5B |
| chr10:72,734,704-72,735,419 | q22.1 | Allelic Imbalance | 715 | 42.86 | 13.33 | 29.52 | 0.00 | 0.05 |  |
| chr10:86,511,959-86,552,499 | q23.1 | Allelic Imbalance | 40540 | 42.86 | 13.33 | 29.52 | 0.00 | 0.05 |  |
| chr10:86,589,093-86,641,504 | q23.1 | Allelic Imbalance | 52411 | 42.86 | 13.33 | 29.52 | 0.00 | 0.05 |  |
| chr10:87,646,690-87,655,709 | q23.1 | Allelic Imbalance | 9019 | 42.86 | 13.33 | 29.52 | 0.00 | 0.05 | GRID1 |
| chr10:88,566,198-88,615,278 | q23.2 | Allelic Imbalance | 49080 | 42.86 | 13.33 | 29.52 | 0.00 | 0.05 | BMPR1A |
| chr10:88,745,952-88,803,520 | q23.2 | Allelic Imbalance | 57568 | 42.86 | 13.33 | 29.52 | 0.00 | 0.05 | AGAP11, FAM25A, GLUD1 |
| chr10:89,587,936-89,589,445 | q23.2 | Allelic Imbalance | 1509 | 42.86 | 13.33 | 29.52 | 0.00 | 0.05 | CFL1P1 |
| chr10:91,567,256-91,681,905 | q23.31 | Allelic Imbalance | 114649 | 42.86 | 13.33 | 29.52 | 0.00 | 0.05 | LOC643529 |
| chr10:94,528,069-94,630,218 | q23.33 | Allelic Imbalance | 102149 | 42.86 | 13.33 | 29.52 | 0.00 | 0.05 | EXOC6 |
| chr10:95,954,926-95,960,867 | q23.33 | Allelic Imbalance | 5941 | 42.86 | 13.33 | 29.52 | 0.00 | 0.05 | PLCE1 |
| chr7:140,427,004-140,438,682 | q34 | Allelic Imbalance | 11678 | 42.86 | 13.33 | 29.52 | 0.00 | 0.05 | TMEM178B |
| chr7:92,135,417-92,138,211 | q21.2 | Allelic Imbalance | 2794 | 42.86 | 13.33 | 29.52 | 0.00 | 0.05 | CDK6 |
| chr10:124,033,480-124,090,650 | q26.13 | Allelic Imbalance | 57170 | 35.71 | 8.89 | 26.83 | 0.00 | 0.05 | BTBD16 |
| chr10:37,392,383-37,436,265 | p11.21 | Allelic Imbalance | 43882 | 35.71 | 8.89 | 26.83 | 0.00 | 0.05 |  |
| chr10:37,566,899-37,612,635 | p11.21 | Allelic Imbalance | 45736 | 35.71 | 8.89 | 26.83 | 0.00 | 0.05 |  |
| chr10:53,761,228-53,931,514 | q21.1 | Allelic Imbalance | 170286 | 35.71 | 8.89 | 26.83 | 0.00 | 0.05 |  |
| chr10:56,242,948-56,338,167 | q21.1 | Allelic Imbalance | 95219 | 35.71 | 8.89 | 26.83 | 0.00 | 0.05 |  |
| chr10:64,190,304-64,439,944 | q21.2 | Allelic Imbalance | 249640 | 35.71 | 8.89 | 26.83 | 0.00 | 0.05 | ADO, EGR2 |
| chr10:78,499,718-78,697,096 | q22.3 | Allelic Imbalance | 197378 | 35.71 | 8.89 | 26.83 | 0.00 | 0.05 | KCNMA1 |
| chr10:78,740,481-78,794,366 | q22.3 | Allelic Imbalance | 53885 | 35.71 | 8.89 | 26.83 | 0.00 | 0.05 | KCNMA1 |
| chr10:89,629,269-89,631,976 | q23.31 | Allelic Imbalance | 2707 | 35.71 | 8.89 | 26.83 | 0.00 | 0.05 | PTEN |
| chr10:89,709,816-89,711,233 | q23.31 | Allelic Imbalance | 1417 | 35.71 | 8.89 | 26.83 | 0.00 | 0.05 | PTEN |
| chr7:140,286,272-140,307,093 | q34 | Allelic Imbalance | 20821 | 35.71 | 8.89 | 26.83 | 0.00 | 0.05 |  |
| chr7:140,317,012-140,334,164 | q34 | Allelic Imbalance | 17152 | 35.71 | 8.89 | 26.83 | 0.00 | 0.05 |  |
| chr7:55,199,037-55,200,221 | p11.2 | Allelic Imbalance | 1184 | 35.71 | 8.89 | 26.83 | 0.00 | 0.05 | EGFR |
| chr7:55,277,390-55,297,359 | p11.2 | Allelic Imbalance | 19969 | 35.71 | 8.89 | 26.83 | 0.00 | 0.05 |  |
| chr10:119,696,063-119,710,535 | q26.11 | Allelic Imbalance | 14472 | 50.00 | 20.00 | 30.00 | 0.00 | 0.05 |  |
| chr10:132,820,857-132,848,187 | q26.3 | Allelic Imbalance | 27330 | 50.00 | 20.00 | 30.00 | 0.00 | 0.05 | TCERG1L |
| chr7:102,940,852-102,942,010 | q22.1 | Allelic Imbalance | 1158 | 50.00 | 20.00 | 30.00 | 0.00 | 0.05 | RELN |
| chr7:102,943,103-102,943,377 | q22.1 | Allelic Imbalance | 274 | 50.00 | 20.00 | 30.00 | 0.00 | 0.05 | RELN |
| chr7:102,994,865-102,996,784 | q22.1 | Allelic Imbalance | 1919 | 50.00 | 20.00 | 30.00 | 0.00 | 0.05 | RELN |
| chr7:103,016,394-103,040,850 | q22.1 | Allelic Imbalance | 24456 | 50.00 | 20.00 | 30.00 | 0.00 | 0.05 | RELN |
| chr7:103,600,746-103,643,542 | q22.1 | Allelic Imbalance | 42796 | 50.00 | 20.00 | 30.00 | 0.00 | 0.05 | ORC5 |
| chr7:103,741,287-103,757,207 | q22.1 | Allelic Imbalance | 15920 | 50.00 | 20.00 | 30.00 | 0.00 | 0.05 | LHFPL3 |
| chr7:69,406,761-69,471,597 | q11.22 | Allelic Imbalance | 64836 | 50.00 | 20.00 | 30.00 | 0.00 | 0.05 | AUTS2 |
| chr7:88,433,862-88,517,101 | q21.13 | Allelic Imbalance | 83239 | 50.00 | 20.00 | 30.00 | 0.00 | 0.05 | ZNF804B |
| chr7:88,521,184-88,532,597 | q21.13 | Allelic Imbalance | 11413 | 50.00 | 20.00 | 30.00 | 0.00 | 0.05 | ZNF804B |
| chr7:94,126,988-94,205,264 | q21.3 | Allelic Imbalance | 78276 | 50.00 | 20.00 | 30.00 | 0.00 | 0.05 | PEG10 |
| chr7:94,381,075-94,455,390 | q21.3 | Allelic Imbalance | 74315 | 50.00 | 20.00 | 30.00 | 0.00 | 0.05 | PPP1R9A |
| chr7:95,864,546-95,867,480 | q21.3 | Allelic Imbalance | 2934 | 50.00 | 20.00 | 30.00 | 0.00 | 0.05 |  |
| chr7:103,777,942-103,790,821 | q22.1 | Allelic Imbalance | 12879 | 52.38 | 22.22 | 30.16 | 0.00 | 0.05 | LHFPL3 |
| chr7:104,201,123-104,204,772 | q22.1 | Allelic Imbalance | 3649 | 52.38 | 22.22 | 30.16 | 0.00 | 0.05 | LHFPL3 |
| chr7:107,692,283-107,717,075 | q31.1 | Allelic Imbalance | 24792 | 52.38 | 22.22 | 30.16 | 0.00 | 0.05 | NRCAM |
| chr7:150,796,233-150,804,098 | q36.1 | Allelic Imbalance | 7865 | 52.38 | 22.22 | 30.16 | 0.00 | 0.05 | RHEB |
| chr7:150,815,688-150,831,042 | q36.1 | Allelic Imbalance | 15354 | 52.38 | 22.22 | 30.16 | 0.00 | 0.05 | RHEB |
| chr7:150,839,365-150,844,522 | q36.1 | Allelic Imbalance | 5157 | 52.38 | 22.22 | 30.16 | 0.00 | 0.05 | RHEB |
| chr7:31,855,606-31,887,548 | p14.3 | Allelic Imbalance | 31942 | 52.38 | 22.22 | 30.16 | 0.00 | 0.05 | PDE1C |
| chr7:75,341,632-75,370,392 | q11.23 | Allelic Imbalance | 28760 | 52.38 | 22.22 | 30.16 | 0.00 | 0.05 | RHBDD2 |
| chr7:88,036,995-88,062,447 | q21.13 | Allelic Imbalance | 25452 | 52.38 | 22.22 | 30.16 | 0.00 | 0.05 |  |
| chr7:89,211,933-89,295,870 | q21.13 | Allelic Imbalance | 83937 | 52.38 | 22.22 | 30.16 | 0.00 | 0.05 |  |
| chr7:93,897,413-93,929,156 | q21.3 | Allelic Imbalance | 31743 | 52.38 | 22.22 | 30.16 | 0.00 | 0.05 | COL1A2 |
| chr7:94,032,593-94,070,451 | q21.3 | Allelic Imbalance | 37858 | 52.38 | 22.22 | 30.16 | 0.00 | 0.05 | SGCE |
| chr7:94,205,264-94,232,111 | q21.3 | Allelic Imbalance | 26847 | 52.38 | 22.22 | 30.16 | 0.00 | 0.05 |  |
| chr7:98,232,546-98,249,602 | q22.1 | Allelic Imbalance | 17056 | 52.38 | 22.22 | 30.16 | 0.00 | 0.05 |  |
| chr7:98,255,696-98,282,056 | q22.1 | Allelic Imbalance | 26360 | 52.38 | 22.22 | 30.16 | 0.00 | 0.05 | TMEM130 |
| chr10:100,165,321-100,216,452 | q24.2 | Allelic Imbalance | 51131 | 45.24 | 15.56 | 29.68 | 0.00 | 0.06 | MIR4685, HPS1, HPSE2 |
| chr10:102,793,734-102,875,861 | q24.31 | Allelic Imbalance | 82127 | 45.24 | 15.56 | 29.68 | 0.00 | 0.06 | KAZALD1, TLX1NB |
| chr10:114,669,954-114,724,956 | q25.2 | Allelic Imbalance | 55002 | 45.24 | 15.56 | 29.68 | 0.00 | 0.06 | TCF7L2 |
| chr10:122,815,032-123,091,740 | q26.12 | Allelic Imbalance | 276708 | 45.24 | 15.56 | 29.68 | 0.00 | 0.06 |  |
| chr10:125,573,258-125,574,980 | q26.13 | Allelic Imbalance | 1722 | 45.24 | 15.56 | 29.68 | 0.00 | 0.06 | CPXM2 |
| chr10:131,415,314-131,416,136 | q26.3 | Allelic Imbalance | 822 | 45.24 | 15.56 | 29.68 | 0.00 | 0.06 | MGMT |
| chr10:131,822,864-131,871,477 | q26.3 | Allelic Imbalance | 48613 | 45.24 | 15.56 | 29.68 | 0.00 | 0.06 | GLRX3 |
| chr10:33,618,610-33,622,156 | p11.22 | Allelic Imbalance | 3546 | 45.24 | 15.56 | 29.68 | 0.00 | 0.06 | NRP1 |
| chr10:47,170,539-47,328,768 | q11.22 | Allelic Imbalance | 158229 | 45.24 | 15.56 | 29.68 | 0.00 | 0.06 | ANXA8L2 |
| chr10:67,574,231-67,704,528 | q21.3 | Allelic Imbalance | 130297 | 45.24 | 15.56 | 29.68 | 0.00 | 0.06 | CTNNA3 |
| chr10:72,745,847-72,775,843 | q22.1 | Allelic Imbalance | 29996 | 45.24 | 15.56 | 29.68 | 0.00 | 0.06 | SLC29A3 |
| chr10:88,413,415-88,462,004 | q23.2 | Allelic Imbalance | 48589 | 45.24 | 15.56 | 29.68 | 0.00 | 0.06 | OPN4, LDB3 |
| chr10:89,561,371-89,564,793 | q23.2 | Allelic Imbalance | 3422 | 45.24 | 15.56 | 29.68 | 0.00 | 0.06 | ATAD1 |
| chr10:91,720,547-91,728,113 | q23.31 | Allelic Imbalance | 7566 | 45.24 | 15.56 | 29.68 | 0.00 | 0.06 |  |
| chr10:91,758,999-91,994,760 | q23.31 | Allelic Imbalance | 235761 | 45.24 | 15.56 | 29.68 | 0.00 | 0.06 |  |
| chr10:92,271,189-92,411,116 | q23.31 | Allelic Imbalance | 139927 | 45.24 | 15.56 | 29.68 | 0.00 | 0.06 |  |
| chr10:92,482,444-92,495,371 | q23.31 | Allelic Imbalance | 12927 | 45.24 | 15.56 | 29.68 | 0.00 | 0.06 | HTR7 |
| chr10:94,898,927-95,014,971 | q23.33 | Allelic Imbalance | 116044 | 45.24 | 15.56 | 29.68 | 0.00 | 0.06 |  |
| chr10:95,172,840-95,180,018 | q23.33 | Allelic Imbalance | 7178 | 45.24 | 15.56 | 29.68 | 0.00 | 0.06 | MYOF |
| chr10:95,508,230-95,549,205 | q23.33 | Allelic Imbalance | 40975 | 45.24 | 15.56 | 29.68 | 0.00 | 0.06 | LGI1 |
| chr10:95,573,792-95,952,284 | q23.33 | Allelic Imbalance | 378492 | 45.24 | 15.56 | 29.68 | 0.00 | 0.06 | SLC35G1, PIPSL, PLCE1 |
| chr7:69,028,544-69,132,124 | q11.22 | Allelic Imbalance | 103580 | 45.24 | 15.56 | 29.68 | 0.00 | 0.06 | AUTS2 |
| chr7:86,634,858-86,641,092 | q21.12 | Allelic Imbalance | 6234 | 45.24 | 15.56 | 29.68 | 0.00 | 0.06 | DMTF1 |
| chr7:86,769,442-86,886,873 | q21.12 | Allelic Imbalance | 117431 | 45.24 | 15.56 | 29.68 | 0.00 | 0.06 | TP53TG1, CROT, ABCB4 |
| chr7:88,072,451-88,097,328 | q21.13 | Allelic Imbalance | 24877 | 45.24 | 15.56 | 29.68 | 0.00 | 0.06 |  |
| chr7:88,395,501-88,396,776 | q21.13 | Allelic Imbalance | 1275 | 45.24 | 15.56 | 29.68 | 0.00 | 0.06 | ZNF804B |
| chr7:92,114,571-92,117,464 | q21.2 | Allelic Imbalance | 2893 | 45.24 | 15.56 | 29.68 | 0.00 | 0.06 | CDK6 |
| chr7:103,428,947-103,431,256 | q22.1 | Allelic Imbalance | 2309 | 54.76 | 24.44 | 30.32 | 0.00 | 0.06 |  |
| chr7:103,839,707-103,851,278 | q22.1 | Allelic Imbalance | 11571 | 54.76 | 24.44 | 30.32 | 0.00 | 0.06 | LHFPL3 |
| chr7:104,204,772-104,209,127 | q22.1 | Allelic Imbalance | 4355 | 54.76 | 24.44 | 30.32 | 0.00 | 0.06 | LHFPL3 |
| chr7:104,213,522-104,281,297 | q22.1 | Allelic Imbalance | 67775 | 54.76 | 24.44 | 30.32 | 0.00 | 0.06 | LHFPL3, LHFPL3-AS1 |
| chr7:107,507,752-107,522,004 | q31.1 | Allelic Imbalance | 14252 | 54.76 | 24.44 | 30.32 | 0.00 | 0.06 | LAMB4 |
| chr7:107,627,625-107,661,561 | q31.1 | Allelic Imbalance | 33936 | 54.76 | 24.44 | 30.32 | 0.00 | 0.06 | NRCAM |
| chr7:114,086,123-114,123,978 | q31.1 | Allelic Imbalance | 37855 | 54.76 | 24.44 | 30.32 | 0.00 | 0.06 | FOXP2 |
| chr7:114,367,107-114,382,106 | q31.1 | Allelic Imbalance | 14999 | 54.76 | 24.44 | 30.32 | 0.00 | 0.06 | MDFIC |
| chr7:114,576,215-114,585,958 | q31.2 | Allelic Imbalance | 9743 | 54.76 | 24.44 | 30.32 | 0.00 | 0.06 |  |
| chr7:114,669,173-114,707,117 | q31.2 | Allelic Imbalance | 37944 | 54.76 | 24.44 | 30.32 | 0.00 | 0.06 |  |
| chr7:114,911,442-114,939,931 | q31.2 | Allelic Imbalance | 28489 | 54.76 | 24.44 | 30.32 | 0.00 | 0.06 |  |
| chr7:115,852,938-116,009,919 | q31.2 | Allelic Imbalance | 156981 | 54.76 | 24.44 | 30.32 | 0.00 | 0.06 | CAV2, CAV1 |
| chr7:118,132,866-118,160,470 | q31.31 | Allelic Imbalance | 27604 | 54.76 | 24.44 | 30.32 | 0.00 | 0.06 |  |
| chr7:126,434,123-126,445,740 | q31.33 | Allelic Imbalance | 11617 | 54.76 | 24.44 | 30.32 | 0.00 | 0.06 | GRM8 |
| chr7:126,499,942-126,535,302 | q31.33 | Allelic Imbalance | 35360 | 54.76 | 24.44 | 30.32 | 0.00 | 0.06 | GRM8 |
| chr7:126,550,310-126,577,210 | q31.33 | Allelic Imbalance | 26900 | 54.76 | 24.44 | 30.32 | 0.00 | 0.06 | GRM8 |
| chr7:126,579,264-126,584,846 | q31.33 | Allelic Imbalance | 5582 | 54.76 | 24.44 | 30.32 | 0.00 | 0.06 | GRM8 |
| chr7:140,461,125-140,464,165 | q34 | Allelic Imbalance | 3040 | 54.76 | 24.44 | 30.32 | 0.00 | 0.06 | TMEM178B |
| chr7:31,835,444-31,835,841 | p14.3 | Allelic Imbalance | 397 | 54.76 | 24.44 | 30.32 | 0.00 | 0.06 | PDE1C |
| chr7:79,569,244-79,597,607 | q21.11 | Allelic Imbalance | 28363 | 54.76 | 24.44 | 30.32 | 0.00 | 0.06 |  |
| chr7:85,271,280-85,280,118 | q21.11 | Allelic Imbalance | 8838 | 54.76 | 24.44 | 30.32 | 0.00 | 0.06 |  |
| chr7:86,212,094-86,241,305 | q21.12 | Allelic Imbalance | 29211 | 54.76 | 24.44 | 30.32 | 0.00 | 0.06 | GRM3 |
| chr7:87,729,066-87,756,703 | q21.12 | Allelic Imbalance | 27637 | 54.76 | 24.44 | 30.32 | 0.00 | 0.06 | STEAP4 |
| chr7:88,575,146-88,575,337 | q21.13 | Allelic Imbalance | 191 | 54.76 | 24.44 | 30.32 | 0.00 | 0.06 | ZNF804B |
| chr7:90,076,226-90,076,420 | q21.13 | Allelic Imbalance | 194 | 54.76 | 24.44 | 30.32 | 0.00 | 0.06 |  |
| chr7:90,096,922-90,112,221 | q21.13 | Allelic Imbalance | 15299 | 54.76 | 24.44 | 30.32 | 0.00 | 0.06 |  |
| chr7:93,819,684-93,839,802 | q21.3 | Allelic Imbalance | 20118 | 54.76 | 24.44 | 30.32 | 0.00 | 0.06 |  |
| chr7:94,747,716-94,760,609 | q21.3 | Allelic Imbalance | 12893 | 54.76 | 24.44 | 30.32 | 0.00 | 0.06 | PPP1R9A |
| chr7:94,917,327-94,923,934 | q21.3 | Allelic Imbalance | 6607 | 54.76 | 24.44 | 30.32 | 0.00 | 0.06 |  |
| chr12:1,915,853-2,084,312 | p13.33 | Allelic Imbalance | 168459 | 9.52 | 35.56 | -26.03 | 0.00 | 0.06 | LOC100271702, DCP1B, CACNA1C |
| chr12:16,166,354-16,409,456 | p12.3 | Allelic Imbalance | 243102 | 9.52 | 35.56 | -26.03 | 0.00 | 0.06 | SLC15A5, MGST1 |
| chr12:2,208,846-2,315,215 | p13.33 | Allelic Imbalance | 106369 | 9.52 | 35.56 | -26.03 | 0.00 | 0.06 | CACNA1C-IT3, CACNA1C |
| chr12:2,703,430-2,756,300 | p13.33 | Allelic Imbalance | 52870 | 9.52 | 35.56 | -26.03 | 0.00 | 0.06 | LOC283440 |
| chr12:3,076,093-3,107,317 | p13.33 - p13.32 | Allelic Imbalance | 31224 | 9.52 | 35.56 | -26.03 | 0.00 | 0.06 | TSPAN9 |
| chr12:3,111,203-3,157,839 | p13.32 | Allelic Imbalance | 46636 | 9.52 | 35.56 | -26.03 | 0.00 | 0.06 | TSPAN9 |
| chr12:6,022,954-6,024,074 | p13.31 | Allelic Imbalance | 1120 | 9.52 | 35.56 | -26.03 | 0.00 | 0.06 | VWF |
| chr12:76,736,040-76,743,296 | q21.2 | Allelic Imbalance | 7256 | 9.52 | 35.56 | -26.03 | 0.00 | 0.06 |  |
| chr12:80,373,443-80,410,492 | q21.31 | Allelic Imbalance | 37049 | 9.52 | 35.56 | -26.03 | 0.00 | 0.06 | PPFIA2 |
| chr7:103,384,607-103,390,319 | q22.1 | Allelic Imbalance | 5712 | 57.14 | 26.67 | 30.48 | 0.00 | 0.06 | RELN |
| chr7:103,791,118-103,797,428 | q22.1 | Allelic Imbalance | 6310 | 57.14 | 26.67 | 30.48 | 0.00 | 0.06 | LHFPL3 |
| chr7:103,879,808-103,893,566 | q22.1 | Allelic Imbalance | 13758 | 57.14 | 26.67 | 30.48 | 0.00 | 0.06 | LHFPL3 |
| chr7:103,945,624-103,995,196 | q22.1 | Allelic Imbalance | 49572 | 57.14 | 26.67 | 30.48 | 0.00 | 0.06 | LHFPL3 |
| chr7:107,100,836-107,108,926 | q22.3 | Allelic Imbalance | 8090 | 57.14 | 26.67 | 30.48 | 0.00 | 0.06 | SLC26A4 |
| chr7:107,264,179-107,264,464 | q31.1 | Allelic Imbalance | 285 | 57.14 | 26.67 | 30.48 | 0.00 | 0.06 |  |
| chr7:107,284,180-107,294,554 | q31.1 | Allelic Imbalance | 10374 | 57.14 | 26.67 | 30.48 | 0.00 | 0.06 |  |
| chr7:109,976,335-109,995,593 | q31.1 | Allelic Imbalance | 19258 | 57.14 | 26.67 | 30.48 | 0.00 | 0.06 |  |
| chr7:111,505,799-111,534,469 | q31.1 | Allelic Imbalance | 28670 | 57.14 | 26.67 | 30.48 | 0.00 | 0.06 | DOCK4 |
| chr7:112,911,538-112,916,347 | q31.1 | Allelic Imbalance | 4809 | 57.14 | 26.67 | 30.48 | 0.00 | 0.06 |  |
| chr7:114,030,377-114,079,539 | q31.1 | Allelic Imbalance | 49162 | 57.14 | 26.67 | 30.48 | 0.00 | 0.06 | FOXP2 |
| chr7:114,433,311-114,492,505 | q31.2 | Allelic Imbalance | 59194 | 57.14 | 26.67 | 30.48 | 0.00 | 0.06 | MDFIC |
| chr7:114,519,221-114,553,459 | q31.2 | Allelic Imbalance | 34238 | 57.14 | 26.67 | 30.48 | 0.00 | 0.06 |  |
| chr7:114,561,120-114,576,215 | q31.2 | Allelic Imbalance | 15095 | 57.14 | 26.67 | 30.48 | 0.00 | 0.06 |  |
| chr7:118,160,470-118,181,639 | q31.31 | Allelic Imbalance | 21169 | 57.14 | 26.67 | 30.48 | 0.00 | 0.06 |  |
| chr7:126,285,811-126,364,181 | q31.33 | Allelic Imbalance | 78370 | 57.14 | 26.67 | 30.48 | 0.00 | 0.06 | GRM8 |
| chr7:126,423,517-126,425,041 | q31.33 | Allelic Imbalance | 1524 | 57.14 | 26.67 | 30.48 | 0.00 | 0.06 | GRM8 |
| chr7:126,430,254-126,433,327 | q31.33 | Allelic Imbalance | 3073 | 57.14 | 26.67 | 30.48 | 0.00 | 0.06 | GRM8 |
| chr7:137,420,093-137,728,000 | q34 | Allelic Imbalance | 307907 | 71.43 | 40.00 | 31.43 | 0.00 | 0.06 | AKR1D1, MIR4468 |
| chr7:137,823,300-137,925,410 | q34 | Allelic Imbalance | 102110 | 71.43 | 40.00 | 31.43 | 0.00 | 0.06 | TRIM24 |
| chr7:137,945,778-137,961,238 | q34 | Allelic Imbalance | 15460 | 71.43 | 40.00 | 31.43 | 0.00 | 0.06 | SVOPL |
| chr7:138,831,501-138,846,439 | q34 | Allelic Imbalance | 14938 | 71.43 | 40.00 | 31.43 | 0.00 | 0.06 |  |
| chr7:143,784,595-143,832,077 | q35 | Allelic Imbalance | 47482 | 71.43 | 40.00 | 31.43 | 0.00 | 0.06 | TPK1 |
| chr7:80,862,829-80,936,959 | q21.11 | Allelic Imbalance | 74130 | 57.14 | 26.67 | 30.48 | 0.00 | 0.06 |  |
| chr7:81,139,023-81,195,147 | q21.11 | Allelic Imbalance | 56124 | 57.14 | 26.67 | 30.48 | 0.00 | 0.06 | HGF |
| chr7:85,432,209-85,443,979 | q21.11 | Allelic Imbalance | 11770 | 57.14 | 26.67 | 30.48 | 0.00 | 0.06 |  |
| chr7:85,452,460-85,454,603 | q21.11 | Allelic Imbalance | 2143 | 57.14 | 26.67 | 30.48 | 0.00 | 0.06 |  |
| chr7:86,242,327-86,284,795 | q21.12 | Allelic Imbalance | 42468 | 57.14 | 26.67 | 30.48 | 0.00 | 0.06 | GRM3 |
| chr7:87,795,243-87,815,311 | q21.12 | Allelic Imbalance | 20068 | 57.14 | 26.67 | 30.48 | 0.00 | 0.06 |  |
| chr7:87,826,289-87,877,083 | q21.12 | Allelic Imbalance | 50794 | 57.14 | 26.67 | 30.48 | 0.00 | 0.06 |  |
| chr7:89,140,165-89,145,086 | q21.13 | Allelic Imbalance | 4921 | 57.14 | 26.67 | 30.48 | 0.00 | 0.06 |  |
| chr7:89,176,316-89,181,425 | q21.13 | Allelic Imbalance | 5109 | 57.14 | 26.67 | 30.48 | 0.00 | 0.06 |  |
| chr7:90,076,420-90,081,882 | q21.13 | Allelic Imbalance | 5462 | 57.14 | 26.67 | 30.48 | 0.00 | 0.06 |  |
| chr7:90,852,411-90,945,816 | q21.13 - q21.2 | Allelic Imbalance | 93405 | 57.14 | 26.67 | 30.48 | 0.00 | 0.06 |  |
| chr7:90,951,735-91,016,399 | q21.2 | Allelic Imbalance | 64664 | 57.14 | 26.67 | 30.48 | 0.00 | 0.06 |  |
| chr7:91,205,070-91,239,969 | q21.2 | Allelic Imbalance | 34899 | 57.14 | 26.67 | 30.48 | 0.00 | 0.06 |  |
| chr7:93,873,499-93,876,785 | q21.3 | Allelic Imbalance | 3286 | 57.14 | 26.67 | 30.48 | 0.00 | 0.06 | COL1A2 |
| chr7:94,766,054-94,769,536 | q21.3 | Allelic Imbalance | 3482 | 57.14 | 26.67 | 30.48 | 0.00 | 0.06 | PON1 |
| chr7:98,140,629-98,170,405 | q22.1 | Allelic Imbalance | 29776 | 57.14 | 26.67 | 30.48 | 0.00 | 0.06 |  |
| chr10:100,607,639-100,623,960 | q24.2 | Allelic Imbalance | 16321 | 38.10 | 11.11 | 26.98 | 0.01 | 0.06 | HPSE2 |
| chr10:101,093,245-101,141,259 | q24.2 | Allelic Imbalance | 48014 | 38.10 | 11.11 | 26.98 | 0.01 | 0.06 | CNNM1 |
| chr10:102,360,696-102,378,207 | q24.31 | Allelic Imbalance | 17511 | 38.10 | 11.11 | 26.98 | 0.01 | 0.06 |  |
| chr10:112,172,647-112,475,982 | q25.2 | Allelic Imbalance | 303335 | 38.10 | 11.11 | 26.98 | 0.01 | 0.06 | DUSP5, SMC3, RBM20 |
| chr10:112,481,854-112,512,290 | q25.2 | Allelic Imbalance | 30436 | 38.10 | 11.11 | 26.98 | 0.01 | 0.06 | RBM20 |
| chr10:112,561,340-112,571,683 | q25.2 | Allelic Imbalance | 10343 | 38.10 | 11.11 | 26.98 | 0.01 | 0.06 | RBM20 |
| chr10:123,612,874-123,626,944 | q26.13 | Allelic Imbalance | 14070 | 38.10 | 11.11 | 26.98 | 0.01 | 0.06 | ATE1 |
| chr10:123,903,280-123,922,112 | q26.13 | Allelic Imbalance | 18832 | 38.10 | 11.11 | 26.98 | 0.01 | 0.06 | TACC2 |
| chr10:124,092,352-124,094,690 | q26.13 | Allelic Imbalance | 2338 | 38.10 | 11.11 | 26.98 | 0.01 | 0.06 |  |
| chr10:42,175,260-42,334,047 | q11.21 | Allelic Imbalance | 158787 | 38.10 | 11.11 | 26.98 | 0.01 | 0.06 | LOC441666, LINC00839, ZNF37BP |
| chr10:43,561,309-43,659,787 | q11.21 | Allelic Imbalance | 98478 | 38.10 | 11.11 | 26.98 | 0.01 | 0.06 | HNRNPA3P1 |
| chr10:43,662,889-43,673,730 | q11.21 | Allelic Imbalance | 10841 | 38.10 | 11.11 | 26.98 | 0.01 | 0.06 | LINC00619 |
| chr10:51,775,318-51,785,698 | q11.23 | Allelic Imbalance | 10380 | 38.10 | 11.11 | 26.98 | 0.01 | 0.06 | SGMS1 |
| chr10:52,050,261-52,180,315 | q11.23 | Allelic Imbalance | 130054 | 38.10 | 11.11 | 26.98 | 0.01 | 0.06 | SGMS1, ASAH2B |
| chr10:52,406,217-52,628,654 | q11.23 | Allelic Imbalance | 222437 | 38.10 | 11.11 | 26.98 | 0.01 | 0.06 | PRKG1 |
| chr10:53,569,944-53,590,659 | q21.1 | Allelic Imbalance | 20715 | 38.10 | 11.11 | 26.98 | 0.01 | 0.06 | PRKG1 |
| chr10:56,384,264-56,399,622 | q21.1 | Allelic Imbalance | 15358 | 38.10 | 11.11 | 26.98 | 0.01 | 0.06 |  |
| chr10:56,673,081-56,679,264 | q21.1 | Allelic Imbalance | 6183 | 38.10 | 11.11 | 26.98 | 0.01 | 0.06 |  |
| chr10:57,081,983-57,250,902 | q21.1 | Allelic Imbalance | 168919 | 38.10 | 11.11 | 26.98 | 0.01 | 0.06 |  |
| chr10:61,710,220-61,735,036 | q21.2 | Allelic Imbalance | 24816 | 38.10 | 11.11 | 26.98 | 0.01 | 0.06 | ANK3 |
| chr10:61,910,178-61,996,552 | q21.2 | Allelic Imbalance | 86374 | 38.10 | 11.11 | 26.98 | 0.01 | 0.06 | ANK3 |
| chr10:62,205,092-62,214,824 | q21.2 | Allelic Imbalance | 9732 | 38.10 | 11.11 | 26.98 | 0.01 | 0.06 | CDK1 |
| chr10:62,233,965-62,306,334 | q21.2 | Allelic Imbalance | 72369 | 38.10 | 11.11 | 26.98 | 0.01 | 0.06 | RHOBTB1 |
| chr10:64,493,797-64,588,262 | q21.2 | Allelic Imbalance | 94465 | 38.10 | 11.11 | 26.98 | 0.01 | 0.06 | NRBF2 |
| chr10:66,540,391-66,626,581 | q21.3 | Allelic Imbalance | 86190 | 38.10 | 11.11 | 26.98 | 0.01 | 0.06 |  |
| chr10:66,762,961-66,766,562 | q21.3 | Allelic Imbalance | 3601 | 38.10 | 11.11 | 26.98 | 0.01 | 0.06 |  |
| chr10:67,253,452-67,264,043 | q21.3 | Allelic Imbalance | 10591 | 38.10 | 11.11 | 26.98 | 0.01 | 0.06 |  |
| chr10:69,633,336-69,647,818 | q21.3 | Allelic Imbalance | 14482 | 38.10 | 11.11 | 26.98 | 0.01 | 0.06 | MYPN |
| chr10:71,352,640-71,717,229 | q22.1 | Allelic Imbalance | 364589 | 38.10 | 11.11 | 26.98 | 0.01 | 0.06 | COL13A1, H2AFY2, AIFM2, TYSND1, SAR1A, PPA1, NPFFR1 |
| chr10:78,850,717-79,030,361 | q22.3 | Allelic Imbalance | 179644 | 38.10 | 11.11 | 26.98 | 0.01 | 0.06 | KCNMA1 |
| chr10:82,819,599-82,877,391 | q23.1 | Allelic Imbalance | 57792 | 38.10 | 11.11 | 26.98 | 0.01 | 0.06 |  |
| chr10:89,713,016-89,715,023 | q23.31 | Allelic Imbalance | 2007 | 38.10 | 11.11 | 26.98 | 0.01 | 0.06 | PTEN |
| chr10:92,967,584-93,539,725 | q23.32 | Allelic Imbalance | 572141 | 38.10 | 11.11 | 26.98 | 0.01 | 0.06 | PCGF5, LOC100188947, HECTD2, PPP1R3C |
| chr10:94,471,281-94,483,669 | q23.33 | Allelic Imbalance | 12388 | 38.10 | 11.11 | 26.98 | 0.01 | 0.06 |  |
| chr10:96,547,816-96,599,650 | q23.33 | Allelic Imbalance | 51834 | 38.10 | 11.11 | 26.98 | 0.01 | 0.06 | CYP2C19 |
| chr7:100,434,793-100,491,053 | q22.1 | Allelic Imbalance | 56260 | 59.52 | 28.89 | 30.63 | 0.01 | 0.06 | MUC12, MUC17 |
| chr7:100,756,703-100,762,177 | q22.1 | Allelic Imbalance | 5474 | 61.90 | 31.11 | 30.79 | 0.01 | 0.06 |  |
| chr7:101,069,801-101,098,055 | q22.1 | Allelic Imbalance | 28254 | 61.90 | 31.11 | 30.79 | 0.01 | 0.06 |  |
| chr7:101,136,751-101,221,735 | q22.1 | Allelic Imbalance | 84984 | 61.90 | 31.11 | 30.79 | 0.01 | 0.06 |  |
| chr7:105,134,720-105,156,528 | q22.2 | Allelic Imbalance | 21808 | 59.52 | 28.89 | 30.63 | 0.01 | 0.06 | ATXN7L1 |
| chr7:105,176,377-105,184,070 | q22.2 | Allelic Imbalance | 7693 | 61.90 | 31.11 | 30.79 | 0.01 | 0.06 | ATXN7L1 |
| chr7:105,362,959-105,364,395 | q22.2 | Allelic Imbalance | 1436 | 64.29 | 33.33 | 30.95 | 0.01 | 0.06 |  |
| chr7:105,364,395-105,410,120 | q22.2 | Allelic Imbalance | 45725 | 66.67 | 35.56 | 31.11 | 0.01 | 0.06 | CDHR3 |
| chr7:105,586,168-105,596,739 | q22.2 | Allelic Imbalance | 10571 | 64.29 | 33.33 | 30.95 | 0.01 | 0.06 |  |
| chr7:106,232,316-106,247,893 | q22.3 | Allelic Imbalance | 15577 | 64.29 | 33.33 | 30.95 | 0.01 | 0.06 |  |
| chr7:107,257,228-107,262,831 | q31.1 | Allelic Imbalance | 5603 | 59.52 | 28.89 | 30.63 | 0.01 | 0.06 |  |
| chr7:107,303,481-107,369,646 | q31.1 | Allelic Imbalance | 66165 | 59.52 | 28.89 | 30.63 | 0.01 | 0.06 | DLD, LAMB1 |
| chr7:107,903,477-107,935,405 | q31.1 | Allelic Imbalance | 31928 | 61.90 | 31.11 | 30.79 | 0.01 | 0.06 | PNPLA8 |
| chr7:108,736,708-108,891,097 | q31.1 | Allelic Imbalance | 154389 | 59.52 | 28.89 | 30.63 | 0.01 | 0.06 |  |
| chr7:108,914,463-108,986,799 | q31.1 | Allelic Imbalance | 72336 | 59.52 | 28.89 | 30.63 | 0.01 | 0.06 |  |
| chr7:109,022,721-109,079,606 | q31.1 | Allelic Imbalance | 56885 | 59.52 | 28.89 | 30.63 | 0.01 | 0.06 |  |
| chr7:109,092,651-109,101,165 | q31.1 | Allelic Imbalance | 8514 | 61.90 | 31.11 | 30.79 | 0.01 | 0.06 |  |
| chr7:109,286,617-109,400,568 | q31.1 | Allelic Imbalance | 113951 | 61.90 | 31.11 | 30.79 | 0.01 | 0.06 | EIF3IP1 |
| chr7:109,730,896-109,759,587 | q31.1 | Allelic Imbalance | 28691 | 61.90 | 31.11 | 30.79 | 0.01 | 0.06 |  |
| chr7:111,105,333-111,118,883 | q31.1 | Allelic Imbalance | 13550 | 59.52 | 28.89 | 30.63 | 0.01 | 0.06 |  |
| chr7:111,200,942-111,224,079 | q31.1 | Allelic Imbalance | 23137 | 61.90 | 31.11 | 30.79 | 0.01 | 0.06 | DOCK4 |
| chr7:111,229,004-111,275,977 | q31.1 | Allelic Imbalance | 46973 | 59.52 | 28.89 | 30.63 | 0.01 | 0.06 | DOCK4 |
| chr7:111,379,731-111,435,044 | q31.1 | Allelic Imbalance | 55313 | 61.90 | 31.11 | 30.79 | 0.01 | 0.06 | DOCK4 |
| chr7:111,440,655-111,500,003 | q31.1 | Allelic Imbalance | 59348 | 59.52 | 28.89 | 30.63 | 0.01 | 0.06 | DOCK4 |
| chr7:113,501,722-113,525,692 | q31.1 | Allelic Imbalance | 23970 | 59.52 | 28.89 | 30.63 | 0.01 | 0.06 | FOXP2 |
| chr7:113,539,425-113,551,212 | q31.1 | Allelic Imbalance | 11787 | 61.90 | 31.11 | 30.79 | 0.01 | 0.06 | FOXP2 |
| chr7:113,772,898-113,948,266 | q31.1 | Allelic Imbalance | 175368 | 59.52 | 28.89 | 30.63 | 0.01 | 0.06 | FOXP2 |
| chr7:116,334,071-116,350,060 | q31.2 | Allelic Imbalance | 15989 | 59.52 | 28.89 | 30.63 | 0.01 | 0.06 | CAPZA2 |
| chr7:116,356,927-116,384,973 | q31.2 | Allelic Imbalance | 28046 | 61.90 | 31.11 | 30.79 | 0.01 | 0.06 | ST7-AS1, ST7-OT4, ST7 |
| chr7:116,446,680-116,592,568 | q31.2 | Allelic Imbalance | 145888 | 61.90 | 31.11 | 30.79 | 0.01 | 0.06 | ST7, ST7-AS2 |
| chr7:120,484,016-120,485,949 | q31.31 | Allelic Imbalance | 1933 | 59.52 | 28.89 | 30.63 | 0.01 | 0.06 | CPED1 |
| chr7:121,347,014-121,382,601 | q31.32 | Allelic Imbalance | 35587 | 66.67 | 35.56 | 31.11 | 0.01 | 0.06 | PTPRZ1 |
| chr7:121,510,922-121,559,175 | q31.32 | Allelic Imbalance | 48253 | 64.29 | 33.33 | 30.95 | 0.01 | 0.06 | AASS |
| chr7:122,399,659-122,561,902 | q31.32 | Allelic Imbalance | 162243 | 61.90 | 31.11 | 30.79 | 0.01 | 0.06 | TAS2R16, SLC13A1 |
| chr7:122,776,052-122,792,347 | q31.32 | Allelic Imbalance | 16295 | 59.52 | 28.89 | 30.63 | 0.01 | 0.06 |  |
| chr7:123,002,777-123,037,109 | q31.32 | Allelic Imbalance | 34332 | 59.52 | 28.89 | 30.63 | 0.01 | 0.06 | ASB15 |
| chr7:123,992,507-124,006,424 | q31.33 | Allelic Imbalance | 13917 | 59.52 | 28.89 | 30.63 | 0.01 | 0.06 |  |
| chr7:124,255,189-124,351,809 | q31.33 | Allelic Imbalance | 96620 | 59.52 | 28.89 | 30.63 | 0.01 | 0.06 | POT1 |
| chr7:124,485,887-124,561,482 | q31.33 | Allelic Imbalance | 75595 | 59.52 | 28.89 | 30.63 | 0.01 | 0.06 |  |
| chr7:124,601,344-124,656,313 | q31.33 | Allelic Imbalance | 54969 | 61.90 | 31.11 | 30.79 | 0.01 | 0.06 |  |
| chr7:125,208,951-125,395,600 | q31.33 | Allelic Imbalance | 186649 | 64.29 | 33.33 | 30.95 | 0.01 | 0.06 |  |
| chr7:125,737,125-125,769,768 | q31.33 | Allelic Imbalance | 32643 | 64.29 | 33.33 | 30.95 | 0.01 | 0.06 |  |
| chr7:126,264,961-126,273,175 | q31.33 | Allelic Imbalance | 8214 | 59.52 | 28.89 | 30.63 | 0.01 | 0.06 | GRM8 |
| chr7:126,842,316-126,934,959 | q31.33 - q32.1 | Allelic Imbalance | 92643 | 66.67 | 35.56 | 31.11 | 0.01 | 0.06 |  |
| chr7:127,519,185-127,562,661 | q32.1 | Allelic Imbalance | 43476 | 64.29 | 33.33 | 30.95 | 0.01 | 0.06 | SND1 |
| chr7:127,768,638-127,829,629 | q32.1 | Allelic Imbalance | 60991 | 69.05 | 37.78 | 31.27 | 0.01 | 0.06 | RBM28, PRRT4, IMPDH1 |
| chr7:134,277,814-134,283,526 | q33 | Allelic Imbalance | 5712 | 61.90 | 31.11 | 30.79 | 0.01 | 0.06 | CALD1 |
| chr7:134,526,248-134,558,472 | q33 | Allelic Imbalance | 32224 | 64.29 | 33.33 | 30.95 | 0.01 | 0.06 | WDR91 |
| chr7:136,249,607-136,258,342 | q33 | Allelic Imbalance | 8735 | 64.29 | 33.33 | 30.95 | 0.01 | 0.06 | CHRM2, LOC349160 |
| chr7:136,296,448-136,352,529 | q33 | Allelic Imbalance | 56081 | 61.90 | 31.11 | 30.79 | 0.01 | 0.06 | CHRM2, LOC349160 |
| chr7:136,353,250-136,356,383 | q33 | Allelic Imbalance | 3133 | 64.29 | 33.33 | 30.95 | 0.01 | 0.06 | LOC349160 |
| chr7:136,410,915-136,485,324 | q33 | Allelic Imbalance | 74409 | 64.29 | 33.33 | 30.95 | 0.01 | 0.06 | LOC349160 |
| chr7:137,311,281-137,339,485 | q34 | Allelic Imbalance | 28204 | 69.05 | 37.78 | 31.27 | 0.01 | 0.06 | CREB3L2 |
| chr7:138,810,321-138,819,404 | q34 | Allelic Imbalance | 9083 | 69.05 | 37.78 | 31.27 | 0.01 | 0.06 | KLRG2 |
| chr7:138,980,276-138,983,209 | q34 | Allelic Imbalance | 2933 | 69.05 | 37.78 | 31.27 | 0.01 | 0.06 | HIPK2 |
| chr7:139,168,996-139,171,571 | q34 | Allelic Imbalance | 2575 | 59.52 | 28.89 | 30.63 | 0.01 | 0.06 | TBXAS1 |
| chr7:140,586,580-140,600,041 | q34 | Allelic Imbalance | 13461 | 61.90 | 31.11 | 30.79 | 0.01 | 0.06 | TMEM178B |
| chr7:140,671,448-140,684,738 | q34 | Allelic Imbalance | 13290 | 59.52 | 28.89 | 30.63 | 0.01 | 0.06 | TMEM178B |
| chr7:140,747,456-140,765,311 | q34 | Allelic Imbalance | 17855 | 59.52 | 28.89 | 30.63 | 0.01 | 0.06 | TMEM178B |
| chr7:143,445,013-143,464,041 | q35 | Allelic Imbalance | 19028 | 64.29 | 33.33 | 30.95 | 0.01 | 0.06 | OR2A14 |
| chr7:143,603,500-143,722,670 | q35 | Allelic Imbalance | 119170 | 66.67 | 35.56 | 31.11 | 0.01 | 0.06 | LOC728377, OR2A20P, OR2A9P, OR2A42, OR2A1, RNU6-57, ARHGEF5 |
| chr7:143,770,590-143,784,595 | q35 | Allelic Imbalance | 14005 | 69.05 | 37.78 | 31.27 | 0.01 | 0.06 | TPK1 |
| chr7:144,058,341-144,213,180 | q35 | Allelic Imbalance | 154839 | 69.05 | 37.78 | 31.27 | 0.01 | 0.06 | TPK1 |
| chr7:144,554,339-144,969,753 | q35 | Allelic Imbalance | 415414 | 69.05 | 37.78 | 31.27 | 0.01 | 0.06 |  |
| chr7:145,095,279-145,404,680 | q35 | Allelic Imbalance | 309401 | 66.67 | 35.56 | 31.11 | 0.01 | 0.06 |  |
| chr7:145,555,078-145,876,441 | q35 | Allelic Imbalance | 321363 | 64.29 | 33.33 | 30.95 | 0.01 | 0.06 | CNTNAP2 |
| chr7:145,894,992-146,197,766 | q35 | Allelic Imbalance | 302774 | 64.29 | 33.33 | 30.95 | 0.01 | 0.06 | CNTNAP2 |
| chr7:147,214,744-147,253,309 | q35 | Allelic Imbalance | 38565 | 64.29 | 33.33 | 30.95 | 0.01 | 0.06 | CNTNAP2 |
| chr7:147,753,297-147,889,037 | q36.1 | Allelic Imbalance | 135740 | 64.29 | 33.33 | 30.95 | 0.01 | 0.06 |  |
| chr7:148,684,981-148,746,106 | q36.1 | Allelic Imbalance | 61125 | 66.67 | 35.56 | 31.11 | 0.01 | 0.06 |  |
| chr7:148,746,106-148,971,747 | q36.1 | Allelic Imbalance | 225641 | 64.29 | 33.33 | 30.95 | 0.01 | 0.06 | ZNF777, ZNF746, ZNF767 |
| chr7:149,060,451-149,156,722 | q36.1 | Allelic Imbalance | 96271 | 66.67 | 35.56 | 31.11 | 0.01 | 0.06 | KRBA1, ZNF467, SSPO |
| chr7:149,196,629-149,378,035 | q36.1 | Allelic Imbalance | 181406 | 64.29 | 33.33 | 30.95 | 0.01 | 0.06 | ATP6V0E2-AS1, ATP6V0E2 |
| chr7:149,844,348-149,896,345 | q36.1 | Allelic Imbalance | 51997 | 61.90 | 31.11 | 30.79 | 0.01 | 0.06 | GIMAP7, GIMAP4 |
| chr7:149,899,525-149,912,645 | q36.1 | Allelic Imbalance | 13120 | 61.90 | 31.11 | 30.79 | 0.01 | 0.06 | GIMAP4 |
| chr7:150,457,748-150,507,569 | q36.1 | Allelic Imbalance | 49821 | 61.90 | 31.11 | 30.79 | 0.01 | 0.06 | AGAP3, GBX1, ASB10 |
| chr7:150,535,334-150,575,233 | q36.1 | Allelic Imbalance | 39899 | 61.90 | 31.11 | 30.79 | 0.01 | 0.06 | ABCF2, CHPF2, MIR671, SMARCD3 |
| chr7:150,575,233-150,585,156 | q36.1 | Allelic Imbalance | 9923 | 59.52 | 28.89 | 30.63 | 0.01 | 0.06 | SMARCD3 |
| chr7:151,060,251-151,075,138 | q36.1 | Allelic Imbalance | 14887 | 59.52 | 28.89 | 30.63 | 0.01 | 0.06 | PRKAG2 |
| chr7:152,944,313-153,270,294 | q36.2 | Allelic Imbalance | 325981 | 66.67 | 35.56 | 31.11 | 0.01 | 0.06 | DPP6 |
| chr7:153,284,083-153,602,196 | q36.2 | Allelic Imbalance | 318113 | 64.29 | 33.33 | 30.95 | 0.01 | 0.06 | DPP6 |
| chr7:153,662,831-153,692,954 | q36.2 | Allelic Imbalance | 30123 | 61.90 | 31.11 | 30.79 | 0.01 | 0.06 | DPP6 |
| chr7:153,742,234-153,792,967 | q36.2 | Allelic Imbalance | 50733 | 64.29 | 33.33 | 30.95 | 0.01 | 0.06 | DPP6 |
| chr7:154,357,439-154,494,920 | q36.2 | Allelic Imbalance | 137481 | 61.90 | 31.11 | 30.79 | 0.01 | 0.06 | LOC100132707, PAXIP1, LOC202781, LOC100128264, HTR5A |
| chr7:155,063,225-155,127,582 | q36.3 | Allelic Imbalance | 64357 | 64.29 | 33.33 | 30.95 | 0.01 | 0.06 |  |
| chr7:156,638,258-156,655,927 | q36.3 | Allelic Imbalance | 17669 | 61.90 | 31.11 | 30.79 | 0.01 | 0.06 | UBE3C |
| chr7:156,996,134-157,110,366 | q36.3 | Allelic Imbalance | 114232 | 64.29 | 33.33 | 30.95 | 0.01 | 0.06 | MIR153-2, PTPRN2 |
| chr7:157,546,039-157,754,963 | q36.3 | Allelic Imbalance | 208924 | 64.29 | 33.33 | 30.95 | 0.01 | 0.06 | PTPRN2 |
| chr7:55,147,471-55,188,127 | p11.2 | Allelic Imbalance | 40656 | 38.10 | 11.11 | 26.98 | 0.01 | 0.06 | EGFR |
| chr7:55,191,959-55,197,966 | p11.2 | Allelic Imbalance | 6007 | 38.10 | 11.11 | 26.98 | 0.01 | 0.06 | EGFR |
| chr7:81,499,527-81,532,314 | q21.11 | Allelic Imbalance | 32787 | 59.52 | 28.89 | 30.63 | 0.01 | 0.06 | CACNA2D1 |
| chr7:87,817,299-87,819,852 | q21.12 | Allelic Imbalance | 2553 | 59.52 | 28.89 | 30.63 | 0.01 | 0.06 |  |
| chr7:88,386,946-88,388,900 | q21.13 | Allelic Imbalance | 1954 | 38.10 | 11.11 | 26.98 | 0.01 | 0.06 | ZNF804B |
| chr7:88,953,791-89,090,384 | q21.13 | Allelic Imbalance | 136593 | 59.52 | 28.89 | 30.63 | 0.01 | 0.06 |  |
| chr7:90,378,465-90,383,840 | q21.13 | Allelic Imbalance | 5375 | 61.90 | 31.11 | 30.79 | 0.01 | 0.06 | CDK14 |
| chr7:90,582,151-90,665,855 | q21.13 | Allelic Imbalance | 83704 | 61.90 | 31.11 | 30.79 | 0.01 | 0.06 | CDK14 |
| chr7:92,917,618-92,967,713 | q21.3 | Allelic Imbalance | 50095 | 61.90 | 31.11 | 30.79 | 0.01 | 0.06 | MIR653, MIR489, CALCR |
| chr7:92,971,255-93,025,111 | q21.3 | Allelic Imbalance | 53856 | 61.90 | 31.11 | 30.79 | 0.01 | 0.06 | CALCR |
| chr7:93,522,239-93,525,749 | q21.3 | Allelic Imbalance | 3510 | 59.52 | 28.89 | 30.63 | 0.01 | 0.06 |  |
| chr7:94,787,536-94,788,407 | q21.3 | Allelic Imbalance | 871 | 59.52 | 28.89 | 30.63 | 0.01 | 0.06 | PON1 |
| chr7:94,944,848-94,951,046 | q21.3 | Allelic Imbalance | 6198 | 59.52 | 28.89 | 30.63 | 0.01 | 0.06 |  |
| chr7:96,414,178-96,433,910 | q21.3 | Allelic Imbalance | 19732 | 66.67 | 35.56 | 31.11 | 0.01 | 0.06 |  |
| chr7:96,498,608-96,547,406 | q21.3 | Allelic Imbalance | 48798 | 66.67 | 35.56 | 31.11 | 0.01 | 0.06 |  |
| chr7:96,957,172-96,998,165 | q21.3 | Allelic Imbalance | 40993 | 64.29 | 33.33 | 30.95 | 0.01 | 0.06 |  |
| chr7:97,100,728-97,127,411 | q21.3 | Allelic Imbalance | 26683 | 61.90 | 31.11 | 30.79 | 0.01 | 0.06 |  |
| chr10:103,576,944-103,643,291 | q24.32 | Allelic Imbalance | 66347 | 47.62 | 17.78 | 29.84 | 0.01 | 0.06 | LOC100289509, KCNIP2, C10orf76 |
| chr10:119,725,906-120,009,563 | q26.11 | Allelic Imbalance | 283657 | 47.62 | 17.78 | 29.84 | 0.01 | 0.06 | RAB11FIP2, CASC2 |
| chr10:128,134,208-128,391,665 | q26.2 | Allelic Imbalance | 257457 | 47.62 | 17.78 | 29.84 | 0.01 | 0.06 | C10orf90 |
| chr10:131,418,502-131,441,030 | q26.3 | Allelic Imbalance | 22528 | 47.62 | 17.78 | 29.84 | 0.01 | 0.06 | MGMT |
| chr10:131,556,396-131,721,972 | q26.3 | Allelic Imbalance | 165576 | 47.62 | 17.78 | 29.84 | 0.01 | 0.06 | EBF3 |
| chr10:34,637,234-34,777,489 | p11.21 | Allelic Imbalance | 140255 | 47.62 | 17.78 | 29.84 | 0.01 | 0.06 | PARD3 |
| chr10:73,019,890-73,086,864 | q22.1 | Allelic Imbalance | 66974 | 47.62 | 17.78 | 29.84 | 0.01 | 0.06 | CDH23 |
| chr10:73,117,668-73,152,880 | q22.1 | Allelic Imbalance | 35212 | 47.62 | 17.78 | 29.84 | 0.01 | 0.06 | CDH23, C10orf105 |
| chr7:102,963,966-102,969,120 | q22.1 | Allelic Imbalance | 5154 | 47.62 | 17.78 | 29.84 | 0.01 | 0.06 | RELN |
| chr7:102,980,678-102,993,766 | q22.1 | Allelic Imbalance | 13088 | 47.62 | 17.78 | 29.84 | 0.01 | 0.06 | RELN |
| chr7:103,686,567-103,722,124 | q22.1 | Allelic Imbalance | 35557 | 47.62 | 17.78 | 29.84 | 0.01 | 0.06 |  |
| chr7:31,922,867-31,925,320 | p14.3 | Allelic Imbalance | 2453 | 47.62 | 17.78 | 29.84 | 0.01 | 0.06 | PDE1C |
| chr7:31,955,926-31,973,736 | p14.3 | Allelic Imbalance | 17810 | 47.62 | 17.78 | 29.84 | 0.01 | 0.06 | PDE1C |
| chr7:31,989,506-31,993,375 | p14.3 | Allelic Imbalance | 3869 | 47.62 | 17.78 | 29.84 | 0.01 | 0.06 | PDE1C |
| chr7:68,546,404-68,563,921 | q11.22 | Allelic Imbalance | 17517 | 47.62 | 17.78 | 29.84 | 0.01 | 0.06 |  |
| chr7:86,578,027-86,596,827 | q21.12 | Allelic Imbalance | 18800 | 47.62 | 17.78 | 29.84 | 0.01 | 0.06 |  |
| chr7:86,697,939-86,707,577 | q21.12 | Allelic Imbalance | 9638 | 47.62 | 17.78 | 29.84 | 0.01 | 0.06 |  |
| chr10:100,437,607-100,484,754 | q24.2 | Allelic Imbalance | 47147 | 40.48 | 13.33 | 27.14 | 0.01 | 0.07 | HPSE2 |
| chr10:101,230,064-101,258,958 | q24.2 | Allelic Imbalance | 28894 | 40.48 | 13.33 | 27.14 | 0.01 | 0.07 |  |
| chr10:107,703,511-107,764,192 | q25.1 | Allelic Imbalance | 60681 | 40.48 | 13.33 | 27.14 | 0.01 | 0.07 |  |
| chr10:111,324,857-111,361,950 | q25.1 | Allelic Imbalance | 37093 | 40.48 | 13.33 | 27.14 | 0.01 | 0.07 |  |
| chr10:111,621,324-111,672,784 | q25.1 | Allelic Imbalance | 51460 | 40.48 | 13.33 | 27.14 | 0.01 | 0.07 | XPNPEP1 |
| chr10:114,366,860-114,518,995 | q25.2 | Allelic Imbalance | 152135 | 40.48 | 13.33 | 27.14 | 0.01 | 0.07 | VTI1A, MIR4295 |
| chr10:114,899,092-115,017,620 | q25.2 - q25.3 | Allelic Imbalance | 118528 | 40.48 | 13.33 | 27.14 | 0.01 | 0.07 | TCF7L2 |
| chr10:115,140,144-115,156,486 | q25.3 | Allelic Imbalance | 16342 | 40.48 | 13.33 | 27.14 | 0.01 | 0.07 |  |
| chr10:117,958,646-117,962,410 | q25.3 | Allelic Imbalance | 3764 | 40.48 | 13.33 | 27.14 | 0.01 | 0.07 | GFRA1 |
| chr10:123,134,793-123,143,373 | q26.13 | Allelic Imbalance | 8580 | 40.48 | 13.33 | 27.14 | 0.01 | 0.07 |  |
| chr10:42,525,983-42,740,266 | q11.21 | Allelic Imbalance | 214283 | 40.48 | 13.33 | 27.14 | 0.01 | 0.07 | BMS1 |
| chr10:48,249,890-49,066,938 | q11.22 | Allelic Imbalance | 817048 | 40.48 | 13.33 | 27.14 | 0.01 | 0.07 | PTPN20A, PTPN20B, GLUD1P7, FAM25B, FAM25C, FAM25G, LOC399753, FRMPD2 |
| chr10:49,101,158-49,278,276 | q11.22 | Allelic Imbalance | 177118 | 40.48 | 13.33 | 27.14 | 0.01 | 0.07 | FRMPD2 |
| chr10:49,344,153-49,455,643 | q11.22 | Allelic Imbalance | 111490 | 40.48 | 13.33 | 27.14 | 0.01 | 0.07 | ARHGAP22 |
| chr10:52,907,481-53,026,634 | q11.23 | Allelic Imbalance | 119153 | 40.48 | 13.33 | 27.14 | 0.01 | 0.07 | PRKG1 |
| chr10:65,970,635-66,254,389 | q21.3 | Allelic Imbalance | 283754 | 40.48 | 13.33 | 27.14 | 0.01 | 0.07 |  |
| chr10:66,309,752-66,467,460 | q21.3 | Allelic Imbalance | 157708 | 40.48 | 13.33 | 27.14 | 0.01 | 0.07 |  |
| chr10:67,154,729-67,166,055 | q21.3 | Allelic Imbalance | 11326 | 40.48 | 13.33 | 27.14 | 0.01 | 0.07 |  |
| chr10:67,290,041-67,295,121 | q21.3 | Allelic Imbalance | 5080 | 40.48 | 13.33 | 27.14 | 0.01 | 0.07 |  |
| chr10:71,979,164-72,143,427 | q22.1 | Allelic Imbalance | 164263 | 40.48 | 13.33 | 27.14 | 0.01 | 0.07 | PALD1, PRF1, ADAMTS14 |
| chr10:72,205,255-72,211,736 | q22.1 | Allelic Imbalance | 6481 | 40.48 | 13.33 | 27.14 | 0.01 | 0.07 | TBATA |
| chr10:72,725,864-72,734,704 | q22.1 | Allelic Imbalance | 8840 | 40.48 | 13.33 | 27.14 | 0.01 | 0.07 | UNC5B |
| chr10:86,462,288-86,511,959 | q23.1 | Allelic Imbalance | 49671 | 40.48 | 13.33 | 27.14 | 0.01 | 0.07 |  |
| chr10:86,552,499-86,589,093 | q23.1 | Allelic Imbalance | 36594 | 40.48 | 13.33 | 27.14 | 0.01 | 0.07 |  |
| chr10:86,641,504-86,818,846 | q23.1 | Allelic Imbalance | 177342 | 40.48 | 13.33 | 27.14 | 0.01 | 0.07 |  |
| chr10:87,415,964-87,646,690 | q23.1 | Allelic Imbalance | 230726 | 40.48 | 13.33 | 27.14 | 0.01 | 0.07 | GRID1 |
| chr10:87,655,709-87,738,688 | q23.1 | Allelic Imbalance | 82979 | 40.48 | 13.33 | 27.14 | 0.01 | 0.07 | GRID1 |
| chr10:88,803,520-89,034,677 | q23.2 | Allelic Imbalance | 231157 | 40.48 | 13.33 | 27.14 | 0.01 | 0.07 | GLUD1, FAM35A, FAM22A, LOC728190 |
| chr10:91,108,066-91,140,106 | q23.31 | Allelic Imbalance | 32040 | 40.48 | 13.33 | 27.14 | 0.01 | 0.07 | IFIT1B |
| chr10:91,189,539-91,567,256 | q23.31 | Allelic Imbalance | 377717 | 40.48 | 13.33 | 27.14 | 0.01 | 0.07 | SLC16A12, MIR107, PANK1, FLJ37201, KIF20B |
| chr12:1,674,318-1,735,227 | p13.33 | Allelic Imbalance | 60909 | 11.90 | 37.78 | -25.87 | 0.01 | 0.07 | ADIPOR2 |
| chr12:1,787,815-1,829,850 | p13.33 | Allelic Imbalance | 42035 | 11.90 | 37.78 | -25.87 | 0.01 | 0.07 | LRTM2, CACNA2D4 |
| chr12:2,919,925-2,987,189 | p13.33 | Allelic Imbalance | 67264 | 11.90 | 37.78 | -25.87 | 0.01 | 0.07 | TULP3, TEAD4 |
| chr12:3,051,199-3,068,100 | p13.33 | Allelic Imbalance | 16901 | 11.90 | 37.78 | -25.87 | 0.01 | 0.07 | TSPAN9 |
| chr12:3,191,502-3,206,739 | p13.32 | Allelic Imbalance | 15237 | 11.90 | 37.78 | -25.87 | 0.01 | 0.07 | TSPAN9 |
| chr12:3,221,490-3,256,567 | p13.32 | Allelic Imbalance | 35077 | 11.90 | 37.78 | -25.87 | 0.01 | 0.07 | TSPAN9 |
| chr7:88,109,905-88,146,286 | q21.13 | Allelic Imbalance | 36381 | 40.48 | 13.33 | 27.14 | 0.01 | 0.07 |  |
| chr7:92,213,218-92,238,685 | q21.2 | Allelic Imbalance | 25467 | 40.48 | 13.33 | 27.14 | 0.01 | 0.07 | CDK6 |
| chr10:132,701,690-132,703,108 | q26.3 | Allelic Imbalance | 1418 | 50.00 | 22.22 | 27.78 | 0.01 | 0.08 |  |
| chr7:103,059,796-103,065,699 | q22.1 | Allelic Imbalance | 5903 | 50.00 | 22.22 | 27.78 | 0.01 | 0.08 | RELN |
| chr7:103,185,310-103,187,627 | q22.1 | Allelic Imbalance | 2317 | 50.00 | 22.22 | 27.78 | 0.01 | 0.08 | RELN |
| chr7:103,197,438-103,236,253 | q22.1 | Allelic Imbalance | 38815 | 50.00 | 22.22 | 27.78 | 0.01 | 0.08 | RELN |
| chr7:103,257,200-103,263,744 | q22.1 | Allelic Imbalance | 6544 | 50.00 | 22.22 | 27.78 | 0.01 | 0.08 | RELN |
| chr7:103,757,207-103,777,942 | q22.1 | Allelic Imbalance | 20735 | 50.00 | 22.22 | 27.78 | 0.01 | 0.08 | LHFPL3 |
| chr7:104,199,713-104,201,123 | q22.1 | Allelic Imbalance | 1410 | 50.00 | 22.22 | 27.78 | 0.01 | 0.08 | LHFPL3 |
| chr7:140,459,733-140,461,125 | q34 | Allelic Imbalance | 1392 | 50.00 | 22.22 | 27.78 | 0.01 | 0.08 | TMEM178B |
| chr7:150,831,042-150,839,365 | q36.1 | Allelic Imbalance | 8323 | 50.00 | 22.22 | 27.78 | 0.01 | 0.08 | RHEB |
| chr7:86,186,990-86,211,065 | q21.11 - q21.12 | Allelic Imbalance | 24075 | 50.00 | 22.22 | 27.78 | 0.01 | 0.08 | GRM3 |
| chr7:88,532,597-88,547,991 | q21.13 | Allelic Imbalance | 15394 | 50.00 | 22.22 | 27.78 | 0.01 | 0.08 | ZNF804B |
| chr7:89,295,870-89,424,635 | q21.13 | Allelic Imbalance | 128765 | 50.00 | 22.22 | 27.78 | 0.01 | 0.08 |  |
| chr7:93,929,156-94,032,593 | q21.3 | Allelic Imbalance | 103437 | 50.00 | 22.22 | 27.78 | 0.01 | 0.08 | CASD1 |
| chr7:94,070,451-94,099,259 | q21.3 | Allelic Imbalance | 28808 | 50.00 | 22.22 | 27.78 | 0.01 | 0.08 | SGCE |
| chr7:94,246,181-94,300,598 | q21.3 | Allelic Imbalance | 54417 | 50.00 | 22.22 | 27.78 | 0.01 | 0.08 |  |
| chr7:95,867,480-95,875,614 | q21.3 | Allelic Imbalance | 8134 | 50.00 | 22.22 | 27.78 | 0.01 | 0.08 |  |
| chr7:98,282,056-98,309,185 | q22.1 | Allelic Imbalance | 27129 | 50.00 | 22.22 | 27.78 | 0.01 | 0.08 | TMEM130 |
| chr7:98,338,181-98,345,537 | q22.1 | Allelic Imbalance | 7356 | 50.00 | 22.22 | 27.78 | 0.01 | 0.08 | TRRAP |
| chr7:98,479,020-98,483,179 | q22.1 | Allelic Imbalance | 4159 | 50.00 | 22.22 | 27.78 | 0.01 | 0.08 | SMURF1 |
| chr10:100,216,452-100,230,483 | q24.2 | Allelic Imbalance | 14031 | 42.86 | 15.56 | 27.30 | 0.01 | 0.08 | HPSE2 |
| chr10:102,777,222-102,793,734 | q24.31 | Allelic Imbalance | 16512 | 42.86 | 15.56 | 27.30 | 0.01 | 0.08 | PDZD7, SFXN3 |
| chr10:110,608,379-110,711,333 | q25.1 | Allelic Imbalance | 102954 | 42.86 | 15.56 | 27.30 | 0.01 | 0.08 |  |
| chr10:114,518,995-114,581,442 | q25.2 | Allelic Imbalance | 62447 | 42.86 | 15.56 | 27.30 | 0.01 | 0.08 | VTI1A |
| chr10:114,592,898-114,669,954 | q25.2 | Allelic Imbalance | 77056 | 42.86 | 15.56 | 27.30 | 0.01 | 0.08 |  |
| chr10:117,853,271-117,901,998 | q25.3 | Allelic Imbalance | 48727 | 42.86 | 15.56 | 27.30 | 0.01 | 0.08 | GFRA1 |
| chr10:123,091,740-123,127,548 | q26.12 - q26.13 | Allelic Imbalance | 35808 | 42.86 | 15.56 | 27.30 | 0.01 | 0.08 |  |
| chr10:125,042,418-125,163,807 | q26.13 | Allelic Imbalance | 121389 | 42.86 | 15.56 | 27.30 | 0.01 | 0.08 |  |
| chr10:125,545,765-125,573,258 | q26.13 | Allelic Imbalance | 27493 | 42.86 | 15.56 | 27.30 | 0.01 | 0.08 | CPXM2 |
| chr10:128,700,650-129,077,162 | q26.2 | Allelic Imbalance | 376512 | 42.86 | 15.56 | 27.30 | 0.01 | 0.08 | FAM196A, DOCK1 |
| chr10:131,361,550-131,372,128 | q26.3 | Allelic Imbalance | 10578 | 42.86 | 15.56 | 27.30 | 0.01 | 0.08 | MGMT |
| chr10:131,871,477-131,890,561 | q26.3 | Allelic Imbalance | 19084 | 42.86 | 15.56 | 27.30 | 0.01 | 0.08 |  |
| chr10:15,744,498-15,749,419 | p13 | Allelic Imbalance | 4921 | 42.86 | 15.56 | 27.30 | 0.01 | 0.08 | ITGA8 |
| chr10:33,575,847-33,618,610 | p11.22 | Allelic Imbalance | 42763 | 42.86 | 15.56 | 27.30 | 0.01 | 0.08 | NRP1 |
| chr10:34,578,938-34,625,302 | p11.21 | Allelic Imbalance | 46364 | 42.86 | 15.56 | 27.30 | 0.01 | 0.08 | PARD3 |
| chr10:67,704,528-67,769,990 | q21.3 | Allelic Imbalance | 65462 | 42.86 | 15.56 | 27.30 | 0.01 | 0.08 | CTNNA3 |
| chr10:72,735,419-72,745,847 | q22.1 | Allelic Imbalance | 10428 | 42.86 | 15.56 | 27.30 | 0.01 | 0.08 |  |
| chr10:80,015,928-80,225,006 | q22.3 | Allelic Imbalance | 209078 | 42.86 | 15.56 | 27.30 | 0.01 | 0.08 |  |
| chr10:81,757,755-81,872,956 | q22.3 | Allelic Imbalance | 115201 | 42.86 | 15.56 | 27.30 | 0.01 | 0.08 | TMEM254-AS1, TMEM254 |
| chr10:81,894,055-81,910,230 | q22.3 | Allelic Imbalance | 16175 | 42.86 | 15.56 | 27.30 | 0.01 | 0.08 | PLAC9, ANXA11 |
| chr10:86,411,989-86,451,383 | q23.1 | Allelic Imbalance | 39394 | 42.86 | 15.56 | 27.30 | 0.01 | 0.08 |  |
| chr10:88,101,581-88,295,280 | q23.2 | Allelic Imbalance | 193699 | 42.86 | 15.56 | 27.30 | 0.01 | 0.08 | GRID1, WAPAL |
| chr10:88,405,948-88,413,415 | q23.2 | Allelic Imbalance | 7467 | 42.86 | 15.56 | 27.30 | 0.01 | 0.08 | OPN4 |
| chr10:88,552,733-88,566,198 | q23.2 | Allelic Imbalance | 13465 | 42.86 | 15.56 | 27.30 | 0.01 | 0.08 | BMPR1A |
| chr10:90,053,050-90,254,102 | q23.31 | Allelic Imbalance | 201052 | 42.86 | 15.56 | 27.30 | 0.01 | 0.08 | RNLS |
| chr10:91,681,905-91,720,547 | q23.31 | Allelic Imbalance | 38642 | 42.86 | 15.56 | 27.30 | 0.01 | 0.08 |  |
| chr10:91,994,760-92,073,985 | q23.31 | Allelic Imbalance | 79225 | 42.86 | 15.56 | 27.30 | 0.01 | 0.08 |  |
| chr10:92,536,896-92,583,470 | q23.31 | Allelic Imbalance | 46574 | 42.86 | 15.56 | 27.30 | 0.01 | 0.08 | HTR7 |
| chr10:94,630,218-94,844,651 | q23.33 | Allelic Imbalance | 214433 | 42.86 | 15.56 | 27.30 | 0.01 | 0.08 | EXOC6, CYP26C1, CYP26A1 |
| chr10:94,860,696-94,898,927 | q23.33 | Allelic Imbalance | 38231 | 42.86 | 15.56 | 27.30 | 0.01 | 0.08 |  |
| chr10:95,180,018-95,190,706 | q23.33 | Allelic Imbalance | 10688 | 42.86 | 15.56 | 27.30 | 0.01 | 0.08 | MYOF |
| chr10:95,387,012-95,414,787 | q23.33 | Allelic Imbalance | 27775 | 42.86 | 15.56 | 27.30 | 0.01 | 0.08 | PDE6C |
| chr10:95,952,284-95,954,926 | q23.33 | Allelic Imbalance | 2642 | 42.86 | 15.56 | 27.30 | 0.01 | 0.08 | PLCE1 |
| chr10:98,779,927-98,832,274 | q24.1 | Allelic Imbalance | 52347 | 42.86 | 15.56 | 27.30 | 0.01 | 0.08 | SLIT1 |
| chr7:86,610,606-86,634,858 | q21.12 | Allelic Imbalance | 24252 | 42.86 | 15.56 | 27.30 | 0.01 | 0.08 | DMTF1 |
| chr7:88,097,328-88,109,905 | q21.13 | Allelic Imbalance | 12577 | 42.86 | 15.56 | 27.30 | 0.01 | 0.08 |  |
| chr7:92,077,374-92,093,374 | q21.2 | Allelic Imbalance | 16000 | 42.86 | 15.56 | 27.30 | 0.01 | 0.08 | CDK6 |
| chr7:92,117,464-92,135,417 | q21.2 | Allelic Imbalance | 17953 | 42.86 | 15.56 | 27.30 | 0.01 | 0.08 | CDK6 |
| chr7:103,324,227-103,329,927 | q22.1 | Allelic Imbalance | 5700 | 52.38 | 24.44 | 27.94 | 0.01 | 0.09 | RELN |
| chr7:104,281,297-104,299,860 | q22.1 | Allelic Imbalance | 18563 | 52.38 | 24.44 | 27.94 | 0.01 | 0.09 | LHFPL3 |
| chr7:107,574,759-107,627,625 | q31.1 | Allelic Imbalance | 52866 | 52.38 | 24.44 | 27.94 | 0.01 | 0.09 | NRCAM |
| chr7:107,661,561-107,692,283 | q31.1 | Allelic Imbalance | 30722 | 52.38 | 24.44 | 27.94 | 0.01 | 0.09 | NRCAM |
| chr7:114,585,958-114,669,173 | q31.2 | Allelic Imbalance | 83215 | 52.38 | 24.44 | 27.94 | 0.01 | 0.09 |  |
| chr7:114,707,117-114,911,442 | q31.2 | Allelic Imbalance | 204325 | 52.38 | 24.44 | 27.94 | 0.01 | 0.09 |  |
| chr7:115,759,605-115,852,938 | q31.2 | Allelic Imbalance | 93333 | 52.38 | 24.44 | 27.94 | 0.01 | 0.09 |  |
| chr7:116,009,919-116,040,196 | q31.2 | Allelic Imbalance | 30277 | 52.38 | 24.44 | 27.94 | 0.01 | 0.09 |  |
| chr7:126,535,302-126,550,310 | q31.33 | Allelic Imbalance | 15008 | 52.38 | 24.44 | 27.94 | 0.01 | 0.09 | GRM8 |
| chr7:126,577,210-126,579,264 | q31.33 | Allelic Imbalance | 2054 | 52.38 | 24.44 | 27.94 | 0.01 | 0.09 | GRM8 |
| chr7:150,844,522-150,845,891 | q36.1 | Allelic Imbalance | 1369 | 52.38 | 24.44 | 27.94 | 0.01 | 0.09 | RHEB |
| chr7:31,835,841-31,855,606 | p14.3 | Allelic Imbalance | 19765 | 52.38 | 24.44 | 27.94 | 0.01 | 0.09 | PDE1C |
| chr7:86,211,065-86,212,094 | q21.12 | Allelic Imbalance | 1029 | 52.38 | 24.44 | 27.94 | 0.01 | 0.09 | GRM3 |
| chr7:87,708,355-87,729,066 | q21.12 | Allelic Imbalance | 20711 | 52.38 | 24.44 | 27.94 | 0.01 | 0.09 |  |
| chr7:88,574,403-88,575,146 | q21.13 | Allelic Imbalance | 743 | 52.38 | 24.44 | 27.94 | 0.01 | 0.09 | ZNF804B |
| chr7:90,058,536-90,076,226 | q21.13 | Allelic Imbalance | 17690 | 52.38 | 24.44 | 27.94 | 0.01 | 0.09 |  |
| chr7:93,527,650-93,539,228 | q21.3 | Allelic Imbalance | 11578 | 52.38 | 24.44 | 27.94 | 0.01 | 0.09 |  |
| chr7:93,804,884-93,819,684 | q21.3 | Allelic Imbalance | 14800 | 52.38 | 24.44 | 27.94 | 0.01 | 0.09 |  |
| chr7:94,835,180-94,891,063 | q21.3 | Allelic Imbalance | 55883 | 52.38 | 24.44 | 27.94 | 0.01 | 0.09 | PON3, PON2 |
| chr7:94,895,160-94,896,812 | q21.3 | Allelic Imbalance | 1652 | 52.38 | 24.44 | 27.94 | 0.01 | 0.09 | PON2 |
| chr7:95,876,510-95,886,483 | q21.3 | Allelic Imbalance | 9973 | 52.38 | 24.44 | 27.94 | 0.01 | 0.09 |  |
| chr7:95,909,431-95,913,460 | q21.3 | Allelic Imbalance | 4029 | 52.38 | 24.44 | 27.94 | 0.01 | 0.09 |  |
| chr7:98,070,991-98,099,897 | q22.1 | Allelic Imbalance | 28906 | 52.38 | 24.44 | 27.94 | 0.01 | 0.09 | NPTX2 |
| chr7:98,215,603-98,232,546 | q22.1 | Allelic Imbalance | 16943 | 52.38 | 24.44 | 27.94 | 0.01 | 0.09 |  |
| chr7:107,093,153-107,100,836 | q22.3 | Allelic Imbalance | 7683 | 54.76 | 26.67 | 28.10 | 0.01 | 0.09 | SLC26A4 |
| chr7:107,490,264-107,507,752 | q31.1 | Allelic Imbalance | 17488 | 54.76 | 26.67 | 28.10 | 0.01 | 0.09 | LAMB4 |
| chr7:112,199,169-112,337,441 | q31.1 | Allelic Imbalance | 138272 | 54.76 | 26.67 | 28.10 | 0.01 | 0.09 | TMEM168, C7orf60 |
| chr7:114,397,163-114,433,311 | q31.1 - q31.2 | Allelic Imbalance | 36148 | 54.76 | 26.67 | 28.10 | 0.01 | 0.09 | MDFIC |
| chr7:114,553,459-114,561,120 | q31.2 | Allelic Imbalance | 7661 | 54.76 | 26.67 | 28.10 | 0.01 | 0.09 |  |
| chr7:114,939,931-115,104,728 | q31.2 | Allelic Imbalance | 164797 | 54.76 | 26.67 | 28.10 | 0.01 | 0.09 |  |
| chr7:115,678,816-115,721,362 | q31.2 | Allelic Imbalance | 42546 | 54.76 | 26.67 | 28.10 | 0.01 | 0.09 | TES |
| chr7:126,425,041-126,430,254 | q31.33 | Allelic Imbalance | 5213 | 54.76 | 26.67 | 28.10 | 0.01 | 0.09 | GRM8 |
| chr7:126,445,740-126,499,942 | q31.33 | Allelic Imbalance | 54202 | 54.76 | 26.67 | 28.10 | 0.01 | 0.09 | GRM8, MIR592 |
| chr7:133,909,697-133,953,528 | q33 | Allelic Imbalance | 43831 | 71.43 | 42.22 | 29.21 | 0.01 | 0.09 | AKR1B15 |
| chr7:137,961,238-138,145,312 | q34 | Allelic Imbalance | 184074 | 71.43 | 42.22 | 29.21 | 0.01 | 0.09 | SVOPL, ATP6V0A4, TMEM213 |
| chr7:143,832,077-143,971,150 | q35 | Allelic Imbalance | 139073 | 71.43 | 42.22 | 29.21 | 0.01 | 0.09 | TPK1 |
| chr7:80,936,959-81,139,023 | q21.11 | Allelic Imbalance | 202064 | 54.76 | 26.67 | 28.10 | 0.01 | 0.09 |  |
| chr7:85,415,160-85,432,209 | q21.11 | Allelic Imbalance | 17049 | 54.76 | 26.67 | 28.10 | 0.01 | 0.09 |  |
| chr7:86,241,305-86,242,327 | q21.12 | Allelic Imbalance | 1022 | 54.76 | 26.67 | 28.10 | 0.01 | 0.09 | GRM3 |
| chr7:86,297,029-86,382,347 | q21.12 | Allelic Imbalance | 85318 | 54.76 | 26.67 | 28.10 | 0.01 | 0.09 | GRM3, KIAA1324L |
| chr7:86,475,190-86,502,273 | q21.12 | Allelic Imbalance | 27083 | 54.76 | 26.67 | 28.10 | 0.01 | 0.09 | KIAA1324L |
| chr7:87,756,703-87,795,243 | q21.12 | Allelic Imbalance | 38540 | 54.76 | 26.67 | 28.10 | 0.01 | 0.09 | STEAP4 |
| chr7:88,575,337-88,575,355 | q21.13 | Allelic Imbalance | 18 | 54.76 | 26.67 | 28.10 | 0.01 | 0.09 | ZNF804B |
| chr7:90,081,882-90,096,922 | q21.13 | Allelic Imbalance | 15040 | 54.76 | 26.67 | 28.10 | 0.01 | 0.09 |  |
| chr7:90,805,481-90,852,411 | q21.13 | Allelic Imbalance | 46930 | 54.76 | 26.67 | 28.10 | 0.01 | 0.09 |  |
| chr7:91,056,093-91,126,045 | q21.2 | Allelic Imbalance | 69952 | 54.76 | 26.67 | 28.10 | 0.01 | 0.09 |  |
| chr7:91,239,969-91,268,541 | q21.2 | Allelic Imbalance | 28572 | 54.76 | 26.67 | 28.10 | 0.01 | 0.09 |  |
| chr7:93,525,749-93,527,650 | q21.3 | Allelic Imbalance | 1901 | 54.76 | 26.67 | 28.10 | 0.01 | 0.09 |  |
| chr7:93,839,802-93,855,940 | q21.3 | Allelic Imbalance | 16138 | 54.76 | 26.67 | 28.10 | 0.01 | 0.09 |  |
| chr7:94,760,609-94,766,054 | q21.3 | Allelic Imbalance | 5445 | 54.76 | 26.67 | 28.10 | 0.01 | 0.09 | PPP1R9A, PON1 |
| chr7:94,912,367-94,917,327 | q21.3 | Allelic Imbalance | 4960 | 54.76 | 26.67 | 28.10 | 0.01 | 0.09 |  |
| chr7:98,033,391-98,047,655 | q22.1 | Allelic Imbalance | 14264 | 54.76 | 26.67 | 28.10 | 0.01 | 0.09 |  |
| chr7:98,115,932-98,140,629 | q22.1 | Allelic Imbalance | 24697 | 54.76 | 26.67 | 28.10 | 0.01 | 0.09 |  |
| chr7:98,170,405-98,215,603 | q22.1 | Allelic Imbalance | 45198 | 54.76 | 26.67 | 28.10 | 0.01 | 0.09 |  |
| chr10:100,150,978-100,165,321 | q24.2 | Allelic Imbalance | 14343 | 45.24 | 17.78 | 27.46 | 0.01 | 0.09 | PYROXD2 |
| chr10:103,643,291-103,747,468 | q24.32 | Allelic Imbalance | 104177 | 45.24 | 17.78 | 27.46 | 0.01 | 0.09 | C10orf76 |
| chr10:110,131,767-110,242,889 | q25.1 | Allelic Imbalance | 111122 | 45.24 | 17.78 | 27.46 | 0.01 | 0.09 |  |
| chr10:110,554,024-110,562,791 | q25.1 | Allelic Imbalance | 8767 | 45.24 | 17.78 | 27.46 | 0.01 | 0.09 |  |
| chr10:121,193,281-121,211,569 | q26.11 | Allelic Imbalance | 18288 | 45.24 | 17.78 | 27.46 | 0.01 | 0.09 | GRK5 |
| chr10:122,287,624-122,336,194 | q26.12 | Allelic Imbalance | 48570 | 45.24 | 17.78 | 27.46 | 0.01 | 0.09 | PPAPDC1A, MIR5694 |
| chr10:122,756,517-122,815,032 | q26.12 | Allelic Imbalance | 58515 | 45.24 | 17.78 | 27.46 | 0.01 | 0.09 | MIR5694 |
| chr10:125,574,980-125,575,446 | q26.13 | Allelic Imbalance | 466 | 45.24 | 17.78 | 27.46 | 0.01 | 0.09 | CPXM2 |
| chr10:128,391,665-128,700,650 | q26.2 | Allelic Imbalance | 308985 | 45.24 | 17.78 | 27.46 | 0.01 | 0.09 | DOCK1 |
| chr10:129,553,629-129,792,042 | q26.2 | Allelic Imbalance | 238413 | 45.24 | 17.78 | 27.46 | 0.01 | 0.09 | CLRN3, PTPRE, MKI67 |
| chr10:131,392,552-131,415,314 | q26.3 | Allelic Imbalance | 22762 | 45.24 | 17.78 | 27.46 | 0.01 | 0.09 | MGMT |
| chr10:131,416,136-131,418,502 | q26.3 | Allelic Imbalance | 2366 | 45.24 | 17.78 | 27.46 | 0.01 | 0.09 | MGMT |
| chr10:33,622,156-33,654,612 | p11.22 | Allelic Imbalance | 32456 | 45.24 | 17.78 | 27.46 | 0.01 | 0.09 | NRP1 |
| chr10:34,625,302-34,637,234 | p11.21 | Allelic Imbalance | 11932 | 45.24 | 17.78 | 27.46 | 0.01 | 0.09 | PARD3 |
| chr10:34,777,489-34,854,189 | p11.21 | Allelic Imbalance | 76700 | 45.24 | 17.78 | 27.46 | 0.01 | 0.09 | PARD3 |
| chr10:72,775,843-73,019,890 | q22.1 | Allelic Imbalance | 244047 | 45.24 | 17.78 | 27.46 | 0.01 | 0.09 | SLC29A3, CDH23 |
| chr10:73,152,880-73,209,270 | q22.1 | Allelic Imbalance | 56390 | 45.24 | 17.78 | 27.46 | 0.01 | 0.09 | CDH23, C10orf105, C10orf54 |
| chr10:88,462,004-88,525,460 | q23.2 | Allelic Imbalance | 63456 | 45.24 | 17.78 | 27.46 | 0.01 | 0.09 | LDB3, BMPR1A |
| chr10:89,557,840-89,561,371 | q23.2 | Allelic Imbalance | 3531 | 45.24 | 17.78 | 27.46 | 0.01 | 0.09 | ATAD1 |
| chr10:91,728,701-91,758,999 | q23.31 | Allelic Imbalance | 30298 | 45.24 | 17.78 | 27.46 | 0.01 | 0.09 |  |
| chr10:92,094,560-92,271,189 | q23.31 | Allelic Imbalance | 176629 | 45.24 | 17.78 | 27.46 | 0.01 | 0.09 |  |
| chr7:100,434,694-100,434,793 | q22.1 | Allelic Imbalance | 99 | 57.14 | 28.89 | 28.25 | 0.01 | 0.09 | MUC12 |
| chr7:100,491,053-100,542,136 | q22.1 | Allelic Imbalance | 51083 | 59.52 | 31.11 | 28.41 | 0.01 | 0.09 | TRIM56 |
| chr7:100,571,228-100,595,807 | q22.1 | Allelic Imbalance | 24579 | 61.90 | 33.33 | 28.57 | 0.01 | 0.09 | AP1S1, MIR4653, VGF |
| chr7:100,732,713-100,756,703 | q22.1 | Allelic Imbalance | 23990 | 61.90 | 33.33 | 28.57 | 0.01 | 0.09 | RABL5 |
| chr7:100,762,177-100,853,878 | q22.1 | Allelic Imbalance | 91701 | 59.52 | 31.11 | 28.41 | 0.01 | 0.09 | EMID2 |
| chr7:100,973,729-100,995,705 | q22.1 | Allelic Imbalance | 21976 | 59.52 | 31.11 | 28.41 | 0.01 | 0.09 | EMID2 |
| chr7:101,053,139-101,069,801 | q22.1 | Allelic Imbalance | 16662 | 59.52 | 31.11 | 28.41 | 0.01 | 0.09 | MYL10 |
| chr7:101,336,367-101,522,364 | q22.1 | Allelic Imbalance | 185997 | 61.90 | 33.33 | 28.57 | 0.01 | 0.09 | CUX1 |
| chr7:101,810,575-101,840,289 | q22.1 | Allelic Imbalance | 29714 | 57.14 | 28.89 | 28.25 | 0.01 | 0.09 | LOC100630923, PRKRIP1 |
| chr7:102,865,284-102,883,525 | q22.1 | Allelic Imbalance | 18241 | 45.24 | 17.78 | 27.46 | 0.01 | 0.09 | SLC26A5 |
| chr7:102,915,230-102,929,485 | q22.1 | Allelic Imbalance | 14255 | 45.24 | 17.78 | 27.46 | 0.01 | 0.09 | RELN |
| chr7:102,969,120-102,974,634 | q22.1 | Allelic Imbalance | 5514 | 45.24 | 17.78 | 27.46 | 0.01 | 0.09 | RELN |
| chr7:105,066,767-105,102,336 | q22.2 | Allelic Imbalance | 35569 | 57.14 | 28.89 | 28.25 | 0.01 | 0.09 | ATXN7L1 |
| chr7:105,129,863-105,134,720 | q22.2 | Allelic Imbalance | 4857 | 57.14 | 28.89 | 28.25 | 0.01 | 0.09 | ATXN7L1 |
| chr7:105,156,528-105,176,377 | q22.2 | Allelic Imbalance | 19849 | 59.52 | 31.11 | 28.41 | 0.01 | 0.09 | ATXN7L1 |
| chr7:105,596,739-105,690,428 | q22.2 | Allelic Imbalance | 93689 | 61.90 | 33.33 | 28.57 | 0.01 | 0.09 | NAMPT |
| chr7:106,118,167-106,151,357 | q22.3 | Allelic Imbalance | 33190 | 59.52 | 31.11 | 28.41 | 0.01 | 0.09 |  |
| chr7:106,180,600-106,188,974 | q22.3 | Allelic Imbalance | 8374 | 59.52 | 31.11 | 28.41 | 0.01 | 0.09 |  |
| chr7:106,191,746-106,197,659 | q22.3 | Allelic Imbalance | 5913 | 61.90 | 33.33 | 28.57 | 0.01 | 0.09 |  |
| chr7:106,202,729-106,232,316 | q22.3 | Allelic Imbalance | 29587 | 61.90 | 33.33 | 28.57 | 0.01 | 0.09 |  |
| chr7:106,247,893-106,255,147 | q22.3 | Allelic Imbalance | 7254 | 61.90 | 33.33 | 28.57 | 0.01 | 0.09 |  |
| chr7:107,262,831-107,264,179 | q31.1 | Allelic Imbalance | 1348 | 57.14 | 28.89 | 28.25 | 0.01 | 0.09 |  |
| chr7:107,369,646-107,369,824 | q31.1 | Allelic Imbalance | 178 | 57.14 | 28.89 | 28.25 | 0.01 | 0.09 | LAMB1 |
| chr7:107,395,369-107,430,340 | q31.1 | Allelic Imbalance | 34971 | 59.52 | 31.11 | 28.41 | 0.01 | 0.09 | LAMB1 |
| chr7:107,935,405-107,972,407 | q31.1 | Allelic Imbalance | 37002 | 61.90 | 33.33 | 28.57 | 0.01 | 0.09 | PNPLA8 |
| chr7:108,686,027-108,736,708 | q31.1 | Allelic Imbalance | 50681 | 59.52 | 31.11 | 28.41 | 0.01 | 0.09 |  |
| chr7:108,891,097-108,894,937 | q31.1 | Allelic Imbalance | 3840 | 59.52 | 31.11 | 28.41 | 0.01 | 0.09 |  |
| chr7:108,897,946-108,914,463 | q31.1 | Allelic Imbalance | 16517 | 57.14 | 28.89 | 28.25 | 0.01 | 0.09 |  |
| chr7:108,986,799-109,022,721 | q31.1 | Allelic Imbalance | 35922 | 57.14 | 28.89 | 28.25 | 0.01 | 0.09 |  |
| chr7:109,079,606-109,092,651 | q31.1 | Allelic Imbalance | 13045 | 59.52 | 31.11 | 28.41 | 0.01 | 0.09 |  |
| chr7:109,759,587-109,770,172 | q31.1 | Allelic Imbalance | 10585 | 59.52 | 31.11 | 28.41 | 0.01 | 0.09 |  |
| chr7:109,900,565-109,936,365 | q31.1 | Allelic Imbalance | 35800 | 57.14 | 28.89 | 28.25 | 0.01 | 0.09 |  |
| chr7:109,949,203-109,976,335 | q31.1 | Allelic Imbalance | 27132 | 57.14 | 28.89 | 28.25 | 0.01 | 0.09 |  |
| chr7:109,995,593-110,058,837 | q31.1 | Allelic Imbalance | 63244 | 57.14 | 28.89 | 28.25 | 0.01 | 0.09 |  |
| chr7:111,118,883-111,170,843 | q31.1 | Allelic Imbalance | 51960 | 57.14 | 28.89 | 28.25 | 0.01 | 0.09 | DOCK4 |
| chr7:111,180,407-111,192,853 | q31.1 | Allelic Imbalance | 12446 | 59.52 | 31.11 | 28.41 | 0.01 | 0.09 | DOCK4 |
| chr7:111,192,853-111,200,942 | q31.1 | Allelic Imbalance | 8089 | 61.90 | 33.33 | 28.57 | 0.01 | 0.09 | DOCK4 |
| chr7:111,224,079-111,229,004 | q31.1 | Allelic Imbalance | 4925 | 59.52 | 31.11 | 28.41 | 0.01 | 0.09 | DOCK4 |
| chr7:111,340,063-111,379,731 | q31.1 | Allelic Imbalance | 39668 | 59.52 | 31.11 | 28.41 | 0.01 | 0.09 | DOCK4 |
| chr7:111,435,044-111,440,655 | q31.1 | Allelic Imbalance | 5611 | 59.52 | 31.11 | 28.41 | 0.01 | 0.09 | DOCK4 |
| chr7:112,916,347-113,010,824 | q31.1 | Allelic Imbalance | 94477 | 57.14 | 28.89 | 28.25 | 0.01 | 0.09 |  |
| chr7:113,109,535-113,501,722 | q31.1 | Allelic Imbalance | 392187 | 59.52 | 31.11 | 28.41 | 0.01 | 0.09 | PPP1R3A |
| chr7:113,948,266-113,970,300 | q31.1 | Allelic Imbalance | 22034 | 57.14 | 28.89 | 28.25 | 0.01 | 0.09 | FOXP2 |
| chr7:115,637,147-115,678,816 | q31.2 | Allelic Imbalance | 41669 | 57.14 | 28.89 | 28.25 | 0.01 | 0.09 | TES |
| chr7:116,327,489-116,334,071 | q31.2 | Allelic Imbalance | 6582 | 57.14 | 28.89 | 28.25 | 0.01 | 0.09 | CAPZA2 |
| chr7:116,384,973-116,446,680 | q31.2 | Allelic Imbalance | 61707 | 61.90 | 33.33 | 28.57 | 0.01 | 0.09 | ST7-OT4, ST7 |
| chr7:120,485,949-120,500,457 | q31.31 | Allelic Imbalance | 14508 | 59.52 | 31.11 | 28.41 | 0.01 | 0.09 | CPED1 |
| chr7:120,960,288-120,961,996 | q31.32 | Allelic Imbalance | 1708 | 64.29 | 35.56 | 28.73 | 0.01 | 0.09 |  |
| chr7:121,007,160-121,054,446 | q31.32 | Allelic Imbalance | 47286 | 69.05 | 40.00 | 29.05 | 0.01 | 0.09 |  |
| chr7:121,342,436-121,347,014 | q31.32 | Allelic Imbalance | 4578 | 66.67 | 37.78 | 28.89 | 0.01 | 0.09 | PTPRZ1 |
| chr7:121,382,601-121,510,922 | q31.32 | Allelic Imbalance | 128321 | 64.29 | 35.56 | 28.73 | 0.01 | 0.09 | PTPRZ1, AASS |
| chr7:122,561,902-122,776,052 | q31.32 | Allelic Imbalance | 214150 | 59.52 | 31.11 | 28.41 | 0.01 | 0.09 | SLC13A1 |
| chr7:122,792,347-123,002,777 | q31.32 | Allelic Imbalance | 210430 | 57.14 | 28.89 | 28.25 | 0.01 | 0.09 | IQUB, NDUFA5 |
| chr7:123,037,109-123,047,783 | q31.32 | Allelic Imbalance | 10674 | 57.14 | 28.89 | 28.25 | 0.01 | 0.09 | ASB15 |
| chr7:123,742,411-123,992,507 | q31.33 | Allelic Imbalance | 250096 | 57.14 | 28.89 | 28.25 | 0.01 | 0.09 |  |
| chr7:124,006,424-124,128,359 | q31.33 | Allelic Imbalance | 121935 | 59.52 | 31.11 | 28.41 | 0.01 | 0.09 |  |
| chr7:124,249,290-124,255,189 | q31.33 | Allelic Imbalance | 5899 | 57.14 | 28.89 | 28.25 | 0.01 | 0.09 | POT1 |
| chr7:124,656,313-125,208,951 | q31.33 | Allelic Imbalance | 552638 | 61.90 | 33.33 | 28.57 | 0.01 | 0.09 |  |
| chr7:125,769,768-125,831,111 | q31.33 | Allelic Imbalance | 61343 | 61.90 | 33.33 | 28.57 | 0.01 | 0.09 |  |
| chr7:125,831,111-125,905,018 | q31.33 | Allelic Imbalance | 73907 | 59.52 | 31.11 | 28.41 | 0.01 | 0.09 | GRM8 |
| chr7:126,190,216-126,264,961 | q31.33 | Allelic Imbalance | 74745 | 57.14 | 28.89 | 28.25 | 0.01 | 0.09 | GRM8 |
| chr7:126,273,175-126,285,811 | q31.33 | Allelic Imbalance | 12636 | 57.14 | 28.89 | 28.25 | 0.01 | 0.09 | GRM8 |
| chr7:126,934,959-126,992,452 | q32.1 | Allelic Imbalance | 57493 | 64.29 | 35.56 | 28.73 | 0.01 | 0.09 |  |
| chr7:127,500,108-127,519,185 | q32.1 | Allelic Imbalance | 19077 | 64.29 | 35.56 | 28.73 | 0.01 | 0.09 | SND1, MIR593 |
| chr7:127,829,629-127,842,653 | q32.1 | Allelic Imbalance | 13024 | 69.05 | 40.00 | 29.05 | 0.01 | 0.09 | IMPDH1 |
| chr7:128,165,629-128,171,934 | q32.1 | Allelic Imbalance | 6305 | 66.67 | 37.78 | 28.89 | 0.01 | 0.09 | CALU |
| chr7:130,356,225-130,549,164 | q32.3 | Allelic Imbalance | 192939 | 61.90 | 33.33 | 28.57 | 0.01 | 0.09 | FLJ43663, MKLN1 |
| chr7:130,830,162-130,892,784 | q32.3 | Allelic Imbalance | 62622 | 64.29 | 35.56 | 28.73 | 0.01 | 0.09 | MKLN1, PODXL |
| chr7:130,906,056-130,926,519 | q32.3 | Allelic Imbalance | 20463 | 66.67 | 37.78 | 28.89 | 0.01 | 0.09 |  |
| chr7:131,554,449-131,591,978 | q32.3 | Allelic Imbalance | 37529 | 64.29 | 35.56 | 28.73 | 0.01 | 0.09 | PLXNA4 |
| chr7:133,734,102-133,754,462 | q33 | Allelic Imbalance | 20360 | 66.67 | 37.78 | 28.89 | 0.01 | 0.09 |  |
| chr7:133,953,528-133,989,264 | q33 | Allelic Imbalance | 35736 | 69.05 | 40.00 | 29.05 | 0.01 | 0.09 | BPGM |
| chr7:134,246,701-134,277,814 | q33 | Allelic Imbalance | 31113 | 59.52 | 31.11 | 28.41 | 0.01 | 0.09 | CALD1 |
| chr7:134,558,472-134,593,048 | q33 | Allelic Imbalance | 34576 | 64.29 | 35.56 | 28.73 | 0.01 | 0.09 | STRA8 |
| chr7:134,659,253-134,664,343 | q33 | Allelic Imbalance | 5090 | 66.67 | 37.78 | 28.89 | 0.01 | 0.09 |  |
| chr7:135,082,209-135,136,322 | q33 | Allelic Imbalance | 54113 | 66.67 | 37.78 | 28.89 | 0.01 | 0.09 | FAM180A |
| chr7:136,247,419-136,249,607 | q33 | Allelic Imbalance | 2188 | 61.90 | 33.33 | 28.57 | 0.01 | 0.09 | CHRM2, LOC349160 |
| chr7:136,258,342-136,296,448 | q33 | Allelic Imbalance | 38106 | 61.90 | 33.33 | 28.57 | 0.01 | 0.09 | CHRM2, LOC349160 |
| chr7:136,356,383-136,410,915 | q33 | Allelic Imbalance | 54532 | 64.29 | 35.56 | 28.73 | 0.01 | 0.09 | LOC349160 |
| chr7:136,590,356-136,622,104 | q33 | Allelic Imbalance | 31748 | 66.67 | 37.78 | 28.89 | 0.01 | 0.09 | PTN |
| chr7:136,638,624-136,735,549 | q33 | Allelic Imbalance | 96925 | 66.67 | 37.78 | 28.89 | 0.01 | 0.09 | PTN, DGKI |
| chr7:136,750,546-136,999,861 | q33 | Allelic Imbalance | 249315 | 69.05 | 40.00 | 29.05 | 0.01 | 0.09 | DGKI |
| chr7:137,225,267-137,311,281 | q33 - q34 | Allelic Imbalance | 86014 | 66.67 | 37.78 | 28.89 | 0.01 | 0.09 | CREB3L2, LOC100130880 |
| chr7:137,339,485-137,420,093 | q34 | Allelic Imbalance | 80608 | 69.05 | 40.00 | 29.05 | 0.01 | 0.09 | AKR1D1 |
| chr7:137,728,000-137,748,721 | q34 | Allelic Imbalance | 20721 | 69.05 | 40.00 | 29.05 | 0.01 | 0.09 |  |
| chr7:137,765,515-137,823,300 | q34 | Allelic Imbalance | 57785 | 69.05 | 40.00 | 29.05 | 0.01 | 0.09 | TRIM24 |
| chr7:138,754,015-138,810,321 | q34 | Allelic Imbalance | 56306 | 66.67 | 37.78 | 28.89 | 0.01 | 0.09 | LUC7L2, C7orf55-LUC7L2, LOC100129148, KLRG2 |
| chr7:140,572,835-140,586,580 | q34 | Allelic Imbalance | 13745 | 59.52 | 31.11 | 28.41 | 0.01 | 0.09 | TMEM178B |
| chr7:140,765,311-140,773,863 | q34 | Allelic Imbalance | 8552 | 57.14 | 28.89 | 28.25 | 0.01 | 0.09 | TMEM178B |
| chr7:143,438,132-143,445,013 | q35 | Allelic Imbalance | 6881 | 61.90 | 33.33 | 28.57 | 0.01 | 0.09 | OR2A2 |
| chr7:143,464,041-143,603,500 | q35 | Allelic Imbalance | 139459 | 64.29 | 35.56 | 28.73 | 0.01 | 0.09 | CTAGE4, ARHGEF35, RNU6-57, OR2A42, OR2A1, OR2A9P, OR2A20P, OR2A7, CTAGE4, LOC728377 |
| chr7:144,213,180-144,554,339 | q35 | Allelic Imbalance | 341159 | 66.67 | 37.78 | 28.89 | 0.01 | 0.09 |  |
| chr7:144,969,753-145,095,279 | q35 | Allelic Imbalance | 125526 | 66.67 | 37.78 | 28.89 | 0.01 | 0.09 |  |
| chr7:145,404,680-145,555,078 | q35 | Allelic Imbalance | 150398 | 64.29 | 35.56 | 28.73 | 0.01 | 0.09 | CNTNAP2 |
| chr7:145,876,441-145,894,992 | q35 | Allelic Imbalance | 18551 | 64.29 | 35.56 | 28.73 | 0.01 | 0.09 | CNTNAP2 |
| chr7:147,253,309-147,291,414 | q35 | Allelic Imbalance | 38105 | 61.90 | 33.33 | 28.57 | 0.01 | 0.09 | CNTNAP2, MIR548T |
| chr7:147,660,466-147,753,297 | q36.1 | Allelic Imbalance | 92831 | 64.29 | 35.56 | 28.73 | 0.01 | 0.09 | CNTNAP2, MIR548T |
| chr7:149,156,722-149,196,629 | q36.1 | Allelic Imbalance | 39907 | 64.29 | 35.56 | 28.73 | 0.01 | 0.09 | SSPO, ZNF862, ATP6V0E2-AS1 |
| chr7:156,655,927-156,673,323 | q36.3 | Allelic Imbalance | 17396 | 61.90 | 33.33 | 28.57 | 0.01 | 0.09 | UBE3C |
| chr7:156,814,095-156,861,886 | q36.3 | Allelic Imbalance | 47791 | 61.90 | 33.33 | 28.57 | 0.01 | 0.09 | DNAJB6 |
| chr7:156,975,010-156,996,134 | q36.3 | Allelic Imbalance | 21124 | 64.29 | 35.56 | 28.73 | 0.01 | 0.09 |  |
| chr7:157,876,238-157,989,940 | q36.3 | Allelic Imbalance | 113702 | 59.52 | 31.11 | 28.41 | 0.01 | 0.09 | PTPRN2 |
| chr7:68,563,921-68,648,664 | q11.22 | Allelic Imbalance | 84743 | 45.24 | 17.78 | 27.46 | 0.01 | 0.09 |  |
| chr7:68,824,822-69,028,544 | q11.22 | Allelic Imbalance | 203722 | 45.24 | 17.78 | 27.46 | 0.01 | 0.09 | AUTS2 |
| chr7:85,454,603-85,779,804 | q21.11 | Allelic Imbalance | 325201 | 57.14 | 28.89 | 28.25 | 0.01 | 0.09 |  |
| chr7:86,596,827-86,603,195 | q21.12 | Allelic Imbalance | 6368 | 45.24 | 17.78 | 27.46 | 0.01 | 0.09 |  |
| chr7:87,815,311-87,817,299 | q21.12 | Allelic Imbalance | 1988 | 57.14 | 28.89 | 28.25 | 0.01 | 0.09 |  |
| chr7:88,062,447-88,072,451 | q21.13 | Allelic Imbalance | 10004 | 45.24 | 17.78 | 27.46 | 0.01 | 0.09 |  |
| chr7:88,625,431-88,629,666 | q21.13 | Allelic Imbalance | 4235 | 57.14 | 28.89 | 28.25 | 0.01 | 0.09 | ZNF804B |
| chr7:88,637,505-88,670,275 | q21.13 | Allelic Imbalance | 32770 | 57.14 | 28.89 | 28.25 | 0.01 | 0.09 | ZNF804B |
| chr7:88,813,145-88,829,987 | q21.13 | Allelic Imbalance | 16842 | 57.14 | 28.89 | 28.25 | 0.01 | 0.09 |  |
| chr7:88,869,448-88,893,075 | q21.13 | Allelic Imbalance | 23627 | 57.14 | 28.89 | 28.25 | 0.01 | 0.09 |  |
| chr7:89,090,384-89,140,165 | q21.13 | Allelic Imbalance | 49781 | 57.14 | 28.89 | 28.25 | 0.01 | 0.09 |  |
| chr7:89,181,425-89,184,336 | q21.13 | Allelic Imbalance | 2911 | 57.14 | 28.89 | 28.25 | 0.01 | 0.09 |  |
| chr7:89,490,864-89,512,503 | q21.13 | Allelic Imbalance | 21639 | 45.24 | 17.78 | 27.46 | 0.01 | 0.09 |  |
| chr7:90,146,948-90,357,518 | q21.13 | Allelic Imbalance | 210570 | 57.14 | 28.89 | 28.25 | 0.01 | 0.09 | CDK14 |
| chr7:91,016,399-91,018,505 | q21.2 | Allelic Imbalance | 2106 | 57.14 | 28.89 | 28.25 | 0.01 | 0.09 |  |
| chr7:92,913,539-92,916,949 | q21.3 | Allelic Imbalance | 3410 | 61.90 | 33.33 | 28.57 | 0.01 | 0.09 | CALCR |
| chr7:94,788,407-94,793,048 | q21.3 | Allelic Imbalance | 4641 | 57.14 | 28.89 | 28.25 | 0.01 | 0.09 | PON1 |
| chr7:95,370,280-95,389,065 | q21.3 | Allelic Imbalance | 18785 | 59.52 | 31.11 | 28.41 | 0.01 | 0.09 | DYNC1I1 |
| chr7:95,389,065-95,396,767 | q21.3 | Allelic Imbalance | 7702 | 57.14 | 28.89 | 28.25 | 0.01 | 0.09 | DYNC1I1 |
| chr7:95,451,192-95,457,161 | q21.3 | Allelic Imbalance | 5969 | 59.52 | 31.11 | 28.41 | 0.01 | 0.09 | DYNC1I1 |
| chr7:95,465,925-95,467,575 | q21.3 | Allelic Imbalance | 1650 | 57.14 | 28.89 | 28.25 | 0.01 | 0.09 | DYNC1I1 |
| chr7:95,484,680-95,512,156 | q21.3 | Allelic Imbalance | 27476 | 57.14 | 28.89 | 28.25 | 0.01 | 0.09 | DYNC1I1 |
| chr7:96,397,571-96,414,178 | q21.3 | Allelic Imbalance | 16607 | 64.29 | 35.56 | 28.73 | 0.01 | 0.09 |  |
| chr7:96,998,165-97,021,965 | q21.3 | Allelic Imbalance | 23800 | 61.90 | 33.33 | 28.57 | 0.01 | 0.09 |  |
| chr7:97,069,390-97,100,728 | q21.3 | Allelic Imbalance | 31338 | 61.90 | 33.33 | 28.57 | 0.01 | 0.09 |  |
| chr7:97,127,411-97,142,556 | q21.3 | Allelic Imbalance | 15145 | 59.52 | 31.11 | 28.41 | 0.01 | 0.09 |  |
| chr10:109,359,078-109,648,651 | q25.1 | Allelic Imbalance | 289573 | 47.62 | 20.00 | 27.62 | 0.01 | 0.10 |  |
| chr10:119,662,420-119,696,063 | q26.11 | Allelic Imbalance | 33643 | 47.62 | 20.00 | 27.62 | 0.01 | 0.10 |  |
| chr10:120,009,563-120,041,565 | q26.11 | Allelic Imbalance | 32002 | 47.62 | 20.00 | 27.62 | 0.01 | 0.10 |  |
| chr10:121,027,543-121,035,865 | q26.11 | Allelic Imbalance | 8322 | 47.62 | 20.00 | 27.62 | 0.01 | 0.10 | GRK5 |
| chr10:121,047,797-121,146,555 | q26.11 | Allelic Imbalance | 98758 | 47.62 | 20.00 | 27.62 | 0.01 | 0.10 | GRK5, MIR4681 |
| chr10:121,808,017-121,830,909 | q26.12 | Allelic Imbalance | 22892 | 47.62 | 20.00 | 27.62 | 0.01 | 0.10 |  |
| chr10:122,369,214-122,538,867 | q26.12 | Allelic Imbalance | 169653 | 47.62 | 20.00 | 27.62 | 0.01 | 0.10 | WDR11-AS1, MIR5694 |
| chr10:122,572,527-122,602,293 | q26.12 | Allelic Imbalance | 29766 | 47.62 | 20.00 | 27.62 | 0.01 | 0.10 | WDR11-AS1, MIR5694, WDR11 |
| chr10:126,011,468-126,021,388 | q26.13 | Allelic Imbalance | 9920 | 47.62 | 20.00 | 27.62 | 0.01 | 0.10 |  |
| chr10:132,703,108-132,716,437 | q26.3 | Allelic Imbalance | 13329 | 47.62 | 20.00 | 27.62 | 0.01 | 0.10 |  |
| chr10:132,762,633-132,820,857 | q26.3 | Allelic Imbalance | 58224 | 47.62 | 20.00 | 27.62 | 0.01 | 0.10 | TCERG1L |
| chr10:132,848,187-132,880,512 | q26.3 | Allelic Imbalance | 32325 | 47.62 | 20.00 | 27.62 | 0.01 | 0.10 | TCERG1L |
| chr10:73,086,864-73,117,668 | q22.1 | Allelic Imbalance | 30804 | 47.62 | 20.00 | 27.62 | 0.01 | 0.10 | CDH23 |
| chr7:102,943,377-102,963,966 | q22.1 | Allelic Imbalance | 20589 | 47.62 | 20.00 | 27.62 | 0.01 | 0.10 | RELN |
| chr7:102,979,216-102,980,678 | q22.1 | Allelic Imbalance | 1462 | 47.62 | 20.00 | 27.62 | 0.01 | 0.10 | RELN |
| chr7:103,132,496-103,176,413 | q22.1 | Allelic Imbalance | 43917 | 47.62 | 20.00 | 27.62 | 0.01 | 0.10 | RELN |
| chr7:103,643,542-103,686,567 | q22.1 | Allelic Imbalance | 43025 | 47.62 | 20.00 | 27.62 | 0.01 | 0.10 |  |
| chr7:140,452,289-140,454,757 | q34 | Allelic Imbalance | 2468 | 47.62 | 20.00 | 27.62 | 0.01 | 0.10 | TMEM178B |
| chr7:31,973,736-31,989,506 | p14.3 | Allelic Imbalance | 15770 | 47.62 | 20.00 | 27.62 | 0.01 | 0.10 | PDE1C |
| chr7:86,577,646-86,578,027 | q21.12 | Allelic Imbalance | 381 | 47.62 | 20.00 | 27.62 | 0.01 | 0.10 |  |
| chr7:88,517,101-88,521,184 | q21.13 | Allelic Imbalance | 4083 | 47.62 | 20.00 | 27.62 | 0.01 | 0.10 | ZNF804B |
| chr7:94,118,176-94,126,988 | q21.3 | Allelic Imbalance | 8812 | 47.62 | 20.00 | 27.62 | 0.01 | 0.10 | SGCE, PEG10 |
| chr7:94,373,142-94,381,075 | q21.3 | Allelic Imbalance | 7933 | 47.62 | 20.00 | 27.62 | 0.01 | 0.10 | PPP1R9A |
| chr7:95,838,252-95,864,546 | q21.3 | Allelic Imbalance | 26294 | 47.62 | 20.00 | 27.62 | 0.01 | 0.10 |  |
| chr7:98,345,537-98,390,937 | q22.1 | Allelic Imbalance | 45400 | 47.62 | 20.00 | 27.62 | 0.01 | 0.10 | TRRAP |
| chr10:119,524,346-119,662,420 | q26.11 | Allelic Imbalance | 138074 | 47.62 | 22.22 | 25.40 | 0.01 | 0.12 |  |
| chr10:120,041,565-120,055,761 | q26.11 | Allelic Imbalance | 14196 | 47.62 | 22.22 | 25.40 | 0.01 | 0.12 |  |
| chr10:121,035,865-121,047,797 | q26.11 | Allelic Imbalance | 11932 | 47.62 | 22.22 | 25.40 | 0.01 | 0.12 | GRK5 |
| chr10:121,830,909-121,939,285 | q26.12 | Allelic Imbalance | 108376 | 47.62 | 22.22 | 25.40 | 0.01 | 0.12 |  |
| chr10:132,604,727-132,701,690 | q26.3 | Allelic Imbalance | 96963 | 47.62 | 22.22 | 25.40 | 0.01 | 0.12 | MIR378C |
| chr7:103,040,850-103,059,796 | q22.1 | Allelic Imbalance | 18946 | 47.62 | 22.22 | 25.40 | 0.01 | 0.12 | RELN |
| chr7:103,065,699-103,097,147 | q22.1 | Allelic Imbalance | 31448 | 47.62 | 22.22 | 25.40 | 0.01 | 0.12 | RELN |
| chr7:103,184,985-103,185,310 | q22.1 | Allelic Imbalance | 325 | 47.62 | 22.22 | 25.40 | 0.01 | 0.12 | RELN |
| chr7:103,187,627-103,197,438 | q22.1 | Allelic Imbalance | 9811 | 47.62 | 22.22 | 25.40 | 0.01 | 0.12 | RELN |
| chr7:103,253,897-103,257,200 | q22.1 | Allelic Imbalance | 3303 | 47.62 | 22.22 | 25.40 | 0.01 | 0.12 | RELN |
| chr7:104,199,122-104,199,713 | q22.1 | Allelic Imbalance | 591 | 47.62 | 22.22 | 25.40 | 0.01 | 0.12 | LHFPL3 |
| chr7:104,328,777-104,371,503 | q22.1 | Allelic Imbalance | 42726 | 47.62 | 22.22 | 25.40 | 0.01 | 0.12 | LHFPL3, LHFPL3-AS2 |
| chr7:104,858,920-104,905,009 | q22.2 | Allelic Imbalance | 46089 | 47.62 | 22.22 | 25.40 | 0.01 | 0.12 | PUS7 |
| chr7:86,184,019-86,186,990 | q21.11 | Allelic Imbalance | 2971 | 47.62 | 22.22 | 25.40 | 0.01 | 0.12 | GRM3 |
| chr7:88,547,991-88,574,403 | q21.13 | Allelic Imbalance | 26412 | 47.62 | 22.22 | 25.40 | 0.01 | 0.12 | ZNF804B |
| chr7:89,424,635-89,437,954 | q21.13 | Allelic Imbalance | 13319 | 47.62 | 22.22 | 25.40 | 0.01 | 0.12 |  |
| chr7:91,384,963-91,459,958 | q21.2 | Allelic Imbalance | 74995 | 47.62 | 22.22 | 25.40 | 0.01 | 0.12 | AKAP9 |
| chr7:93,581,561-93,636,332 | q21.3 | Allelic Imbalance | 54771 | 47.62 | 22.22 | 25.40 | 0.01 | 0.12 |  |
| chr7:94,099,259-94,118,176 | q21.3 | Allelic Imbalance | 18917 | 47.62 | 22.22 | 25.40 | 0.01 | 0.12 | SGCE |
| chr7:94,364,443-94,373,142 | q21.3 | Allelic Imbalance | 8699 | 47.62 | 22.22 | 25.40 | 0.01 | 0.12 |  |
| chr7:98,390,937-98,479,020 | q22.1 | Allelic Imbalance | 88083 | 47.62 | 22.22 | 25.40 | 0.01 | 0.12 | TRRAP, SMURF1 |
| chr7:98,483,179-98,525,241 | q22.1 | Allelic Imbalance | 42062 | 47.62 | 22.22 | 25.40 | 0.01 | 0.12 | SMURF1 |
| chr10:89,512,141-89,512,509 | q23.2 | Allelic Imbalance | 368 | 50.00 | 24.44 | 25.56 | 0.02 | 0.12 | ATAD1 |
| chr7:103,236,253-103,245,369 | q22.1 | Allelic Imbalance | 9116 | 50.00 | 24.44 | 25.56 | 0.02 | 0.12 | RELN |
| chr7:103,263,744-103,322,851 | q22.1 | Allelic Imbalance | 59107 | 50.00 | 24.44 | 25.56 | 0.02 | 0.12 | RELN |
| chr7:103,323,638-103,324,227 | q22.1 | Allelic Imbalance | 589 | 50.00 | 24.44 | 25.56 | 0.02 | 0.12 | RELN |
| chr7:104,299,860-104,328,777 | q22.1 | Allelic Imbalance | 28917 | 50.00 | 24.44 | 25.56 | 0.02 | 0.12 | LHFPL3, LHFPL3-AS2 |
| chr7:116,040,196-116,103,900 | q31.2 | Allelic Imbalance | 63704 | 50.00 | 24.44 | 25.56 | 0.02 | 0.12 | MET |
| chr7:116,107,958-116,109,151 | q31.2 | Allelic Imbalance | 1193 | 50.00 | 24.44 | 25.56 | 0.02 | 0.12 | MET |
| chr7:116,162,520-116,221,935 | q31.2 | Allelic Imbalance | 59415 | 50.00 | 24.44 | 25.56 | 0.02 | 0.12 | MET |
| chr7:118,088,448-118,132,866 | q31.31 | Allelic Imbalance | 44418 | 50.00 | 24.44 | 25.56 | 0.02 | 0.12 |  |
| chr7:141,241,184-141,305,391 | q34 | Allelic Imbalance | 64207 | 50.00 | 24.44 | 25.56 | 0.02 | 0.12 | OR9A4, CLEC5A |
| chr7:87,688,763-87,708,355 | q21.12 | Allelic Imbalance | 19592 | 50.00 | 24.44 | 25.56 | 0.02 | 0.12 | SRI |
| chr7:90,056,278-90,058,536 | q21.13 | Allelic Imbalance | 2258 | 50.00 | 24.44 | 25.56 | 0.02 | 0.12 |  |
| chr7:93,539,228-93,581,561 | q21.3 | Allelic Imbalance | 42333 | 50.00 | 24.44 | 25.56 | 0.02 | 0.12 |  |
| chr7:94,300,598-94,301,736 | q21.3 | Allelic Imbalance | 1138 | 50.00 | 24.44 | 25.56 | 0.02 | 0.12 |  |
| chr7:94,891,063-94,895,160 | q21.3 | Allelic Imbalance | 4097 | 50.00 | 24.44 | 25.56 | 0.02 | 0.12 | PON2 |
| chr7:95,875,614-95,876,510 | q21.3 | Allelic Imbalance | 896 | 50.00 | 24.44 | 25.56 | 0.02 | 0.12 |  |
| chr7:95,886,483-95,909,431 | q21.3 | Allelic Imbalance | 22948 | 50.00 | 24.44 | 25.56 | 0.02 | 0.12 |  |
| chr7:98,066,752-98,070,991 | q22.1 | Allelic Imbalance | 4239 | 50.00 | 24.44 | 25.56 | 0.02 | 0.12 |  |
| chr7:98,309,185-98,338,181 | q22.1 | Allelic Imbalance | 28996 | 50.00 | 24.44 | 25.56 | 0.02 | 0.12 | MIR3609, TRRAP |
| chr7:103,995,196-104,000,950 | q22.1 | Allelic Imbalance | 5754 | 52.38 | 26.67 | 25.71 | 0.02 | 0.13 | LHFPL3 |
| chr7:105,032,301-105,035,531 | q22.2 | Allelic Imbalance | 3230 | 52.38 | 26.67 | 25.71 | 0.02 | 0.13 | ATXN7L1 |
| chr7:111,534,469-111,558,022 | q31.1 | Allelic Imbalance | 23553 | 52.38 | 26.67 | 25.71 | 0.02 | 0.13 | DOCK4 |
| chr7:112,126,498-112,199,169 | q31.1 | Allelic Imbalance | 72671 | 52.38 | 26.67 | 25.71 | 0.02 | 0.13 | TMEM168 |
| chr7:116,248,751-116,260,189 | q31.2 | Allelic Imbalance | 11438 | 52.38 | 26.67 | 25.71 | 0.02 | 0.13 |  |
| chr7:140,773,863-140,806,231 | q34 | Allelic Imbalance | 32368 | 52.38 | 26.67 | 25.71 | 0.02 | 0.13 | TMEM178B |
| chr7:141,305,391-141,326,488 | q34 | Allelic Imbalance | 21097 | 52.38 | 26.67 | 25.71 | 0.02 | 0.13 | TAS2R38 |
| chr7:86,502,273-86,509,585 | q21.12 | Allelic Imbalance | 7312 | 52.38 | 26.67 | 25.71 | 0.02 | 0.13 | KIAA1324L |
| chr7:86,516,522-86,529,900 | q21.12 | Allelic Imbalance | 13378 | 52.38 | 26.67 | 25.71 | 0.02 | 0.13 | KIAA1324L |
| chr7:89,184,336-89,211,933 | q21.13 | Allelic Imbalance | 27597 | 52.38 | 26.67 | 25.71 | 0.02 | 0.13 |  |
| chr7:91,126,045-91,139,284 | q21.2 | Allelic Imbalance | 13239 | 52.38 | 26.67 | 25.71 | 0.02 | 0.13 |  |
| chr7:91,189,790-91,205,070 | q21.2 | Allelic Imbalance | 15280 | 52.38 | 26.67 | 25.71 | 0.02 | 0.13 |  |
| chr7:91,268,541-91,274,728 | q21.2 | Allelic Imbalance | 6187 | 52.38 | 26.67 | 25.71 | 0.02 | 0.13 |  |
| chr7:93,803,514-93,804,884 | q21.3 | Allelic Imbalance | 1370 | 52.38 | 26.67 | 25.71 | 0.02 | 0.13 |  |
| chr7:94,831,057-94,835,180 | q21.3 | Allelic Imbalance | 4123 | 52.38 | 26.67 | 25.71 | 0.02 | 0.13 | PON3 |
| chr7:94,896,812-94,912,367 | q21.3 | Allelic Imbalance | 15555 | 52.38 | 26.67 | 25.71 | 0.02 | 0.13 | PON2 |
| chr7:95,014,651-95,057,251 | q21.3 | Allelic Imbalance | 42600 | 52.38 | 26.67 | 25.71 | 0.02 | 0.13 | PDK4 |
| chr7:98,027,138-98,033,391 | q22.1 | Allelic Imbalance | 6253 | 52.38 | 26.67 | 25.71 | 0.02 | 0.13 |  |
| chr7:98,047,655-98,055,733 | q22.1 | Allelic Imbalance | 8078 | 52.38 | 26.67 | 25.71 | 0.02 | 0.13 |  |
| chr7:98,099,897-98,115,932 | q22.1 | Allelic Imbalance | 16035 | 52.38 | 26.67 | 25.71 | 0.02 | 0.13 |  |
| chr7:100,360,088-100,434,694 | q22.1 | Allelic Imbalance | 74606 | 54.76 | 28.89 | 25.87 | 0.02 | 0.13 | MUC12 |
| chr7:101,786,014-101,810,575 | q22.1 | Allelic Imbalance | 24561 | 54.76 | 28.89 | 25.87 | 0.02 | 0.13 | LOC100289561, LOC100630923 |
| chr7:105,035,531-105,066,767 | q22.2 | Allelic Imbalance | 31236 | 54.76 | 28.89 | 25.87 | 0.02 | 0.13 | ATXN7L1 |
| chr7:106,278,115-106,280,211 | q22.3 | Allelic Imbalance | 2096 | 54.76 | 28.89 | 25.87 | 0.02 | 0.13 |  |
| chr7:107,371,778-107,380,329 | q31.1 | Allelic Imbalance | 8551 | 54.76 | 28.89 | 25.87 | 0.02 | 0.13 | LAMB1 |
| chr7:107,483,631-107,490,264 | q31.1 | Allelic Imbalance | 6633 | 54.76 | 28.89 | 25.87 | 0.02 | 0.13 | LAMB4 |
| chr7:109,936,365-109,949,203 | q31.1 | Allelic Imbalance | 12838 | 54.76 | 28.89 | 25.87 | 0.02 | 0.13 |  |
| chr7:114,382,106-114,397,163 | q31.1 | Allelic Imbalance | 15057 | 54.76 | 28.89 | 25.87 | 0.02 | 0.13 | MDFIC |
| chr7:115,104,728-115,319,826 | q31.2 | Allelic Imbalance | 215098 | 54.76 | 28.89 | 25.87 | 0.02 | 0.13 |  |
| chr7:115,507,302-115,637,147 | q31.2 | Allelic Imbalance | 129845 | 54.76 | 28.89 | 25.87 | 0.02 | 0.13 |  |
| chr7:116,269,147-116,327,489 | q31.2 | Allelic Imbalance | 58342 | 54.76 | 28.89 | 25.87 | 0.02 | 0.13 | CAPZA2 |
| chr7:116,705,938-116,719,366 | q31.2 | Allelic Imbalance | 13428 | 54.76 | 28.89 | 25.87 | 0.02 | 0.13 | WNT2 |
| chr7:123,047,783-123,140,318 | q31.32 | Allelic Imbalance | 92535 | 54.76 | 28.89 | 25.87 | 0.02 | 0.13 | ASB15, LMOD2, WASL |
| chr7:123,204,082-123,231,917 | q31.32 | Allelic Imbalance | 27835 | 54.76 | 28.89 | 25.87 | 0.02 | 0.13 |  |
| chr7:123,253,171-123,472,215 | q31.32 | Allelic Imbalance | 219044 | 54.76 | 28.89 | 25.87 | 0.02 | 0.13 | HYAL4, SPAM1, TMEM229A |
| chr7:126,113,506-126,190,216 | q31.33 | Allelic Imbalance | 76710 | 54.76 | 28.89 | 25.87 | 0.02 | 0.13 | GRM8 |
| chr7:133,771,784-133,909,697 | q33 | Allelic Imbalance | 137913 | 69.05 | 42.22 | 26.83 | 0.02 | 0.13 | AKR1B1, AKR1B10, AKR1B15 |
| chr7:133,989,264-134,046,219 | q33 | Allelic Imbalance | 56955 | 69.05 | 42.22 | 26.83 | 0.02 | 0.13 | BPGM |
| chr7:137,748,721-137,765,515 | q34 | Allelic Imbalance | 16794 | 69.05 | 42.22 | 26.83 | 0.02 | 0.13 |  |
| chr7:138,145,312-138,171,269 | q34 | Allelic Imbalance | 25957 | 69.05 | 42.22 | 26.83 | 0.02 | 0.13 | KIAA1549 |
| chr7:138,392,313-138,397,744 | q34 | Allelic Imbalance | 5431 | 69.05 | 42.22 | 26.83 | 0.02 | 0.13 | ZC3HAV1 |
| chr7:140,464,165-140,515,029 | q34 | Allelic Imbalance | 50864 | 54.76 | 28.89 | 25.87 | 0.02 | 0.13 | TMEM178B |
| chr7:141,617,197-141,678,090 | q34 | Allelic Imbalance | 60893 | 54.76 | 28.89 | 25.87 | 0.02 | 0.13 | LOC730441 |
| chr7:142,740,108-142,741,929 | q34 | Allelic Imbalance | 1821 | 54.76 | 28.89 | 25.87 | 0.02 | 0.13 | CLCN1 |
| chr7:143,971,150-144,058,341 | q35 | Allelic Imbalance | 87191 | 69.05 | 42.22 | 26.83 | 0.02 | 0.13 | TPK1 |
| chr7:158,371,146-158,821,424 | q36.3 | Allelic Imbalance | 450278 | 54.76 | 28.89 | 25.87 | 0.02 | 0.13 | WDR60, LINC00689, VIPR2 |
| chr7:85,779,804-85,784,363 | q21.11 | Allelic Imbalance | 4559 | 54.76 | 28.89 | 25.87 | 0.02 | 0.13 |  |
| chr7:86,382,347-86,475,190 | q21.12 | Allelic Imbalance | 92843 | 54.76 | 28.89 | 25.87 | 0.02 | 0.13 | KIAA1324L |
| chr7:88,575,355-88,625,431 | q21.13 | Allelic Imbalance | 50076 | 54.76 | 28.89 | 25.87 | 0.02 | 0.13 | ZNF804B |
| chr7:88,629,666-88,637,505 | q21.13 | Allelic Imbalance | 7839 | 54.76 | 28.89 | 25.87 | 0.02 | 0.13 | ZNF804B |
| chr7:88,670,275-88,788,917 | q21.13 | Allelic Imbalance | 118642 | 54.76 | 28.89 | 25.87 | 0.02 | 0.13 | ZNF804B |
| chr7:88,801,455-88,813,145 | q21.13 | Allelic Imbalance | 11690 | 54.76 | 28.89 | 25.87 | 0.02 | 0.13 | ZNF804B |
| chr7:91,046,085-91,056,093 | q21.2 | Allelic Imbalance | 10008 | 54.76 | 28.89 | 25.87 | 0.02 | 0.13 |  |
| chr7:94,793,048-94,795,160 | q21.3 | Allelic Imbalance | 2112 | 54.76 | 28.89 | 25.87 | 0.02 | 0.13 |  |
| chr7:95,396,767-95,400,169 | q21.3 | Allelic Imbalance | 3402 | 54.76 | 28.89 | 25.87 | 0.02 | 0.13 | DYNC1I1 |
| chr7:95,467,575-95,484,680 | q21.3 | Allelic Imbalance | 17105 | 54.76 | 28.89 | 25.87 | 0.02 | 0.13 | DYNC1I1 |
| chr10:100,085,322-100,150,978 | q24.2 | Allelic Imbalance | 65656 | 42.86 | 17.78 | 25.08 | 0.02 | 0.13 | MIR1287, PYROXD2 |
| chr10:103,747,468-103,786,465 | q24.32 | Allelic Imbalance | 38997 | 42.86 | 17.78 | 25.08 | 0.02 | 0.13 | C10orf76 |
| chr10:107,072,602-107,199,860 | q25.1 | Allelic Imbalance | 127258 | 42.86 | 17.78 | 25.08 | 0.02 | 0.13 |  |
| chr10:110,242,889-110,466,790 | q25.1 | Allelic Imbalance | 223901 | 42.86 | 17.78 | 25.08 | 0.02 | 0.13 |  |
| chr10:110,495,199-110,554,024 | q25.1 | Allelic Imbalance | 58825 | 42.86 | 17.78 | 25.08 | 0.02 | 0.13 |  |
| chr10:110,562,791-110,608,379 | q25.1 | Allelic Imbalance | 45588 | 42.86 | 17.78 | 25.08 | 0.02 | 0.13 |  |
| chr10:125,519,583-125,545,765 | q26.13 | Allelic Imbalance | 26182 | 42.86 | 17.78 | 25.08 | 0.02 | 0.13 | CPXM2 |
| chr10:126,081,112-126,263,789 | q26.13 | Allelic Imbalance | 182677 | 42.86 | 17.78 | 25.08 | 0.02 | 0.13 | OAT, NKX1-2, LHPP |
| chr10:131,379,721-131,392,552 | q26.3 | Allelic Imbalance | 12831 | 42.86 | 17.78 | 25.08 | 0.02 | 0.13 | MGMT |
| chr10:133,227,178-133,251,532 | q26.3 | Allelic Imbalance | 24354 | 42.86 | 17.78 | 25.08 | 0.02 | 0.13 |  |
| chr10:133,451,953-133,765,969 | q26.3 | Allelic Imbalance | 314016 | 42.86 | 17.78 | 25.08 | 0.02 | 0.13 | FLJ46300, PPP2R2D, BNIP3 |
| chr10:15,719,387-15,744,498 | p13 | Allelic Imbalance | 25111 | 42.86 | 17.78 | 25.08 | 0.02 | 0.13 | ITGA8 |
| chr10:15,749,419-15,810,454 | p13 | Allelic Imbalance | 61035 | 42.86 | 17.78 | 25.08 | 0.02 | 0.13 | ITGA8 |
| chr10:33,552,285-33,575,847 | p11.22 | Allelic Imbalance | 23562 | 42.86 | 17.78 | 25.08 | 0.02 | 0.13 | NRP1 |
| chr10:33,654,612-33,712,665 | p11.22 | Allelic Imbalance | 58053 | 42.86 | 17.78 | 25.08 | 0.02 | 0.13 | NRP1 |
| chr10:34,854,189-34,982,522 | p11.21 | Allelic Imbalance | 128333 | 42.86 | 17.78 | 25.08 | 0.02 | 0.13 | PARD3 |
| chr10:35,070,241-35,098,365 | p11.21 | Allelic Imbalance | 28124 | 42.86 | 17.78 | 25.08 | 0.02 | 0.13 | PARD3 |
| chr10:47,164,328-47,170,539 | q11.22 | Allelic Imbalance | 6211 | 42.86 | 17.78 | 25.08 | 0.02 | 0.13 |  |
| chr10:67,769,990-67,770,084 | q21.3 | Allelic Imbalance | 94 | 42.86 | 17.78 | 25.08 | 0.02 | 0.13 | CTNNA3 |
| chr10:73,209,270-73,212,190 | q22.1 | Allelic Imbalance | 2920 | 42.86 | 17.78 | 25.08 | 0.02 | 0.13 | CDH23 |
| chr10:80,225,006-80,315,194 | q22.3 | Allelic Imbalance | 90188 | 42.86 | 17.78 | 25.08 | 0.02 | 0.13 |  |
| chr10:80,873,383-80,887,921 | q22.3 | Allelic Imbalance | 14538 | 42.86 | 17.78 | 25.08 | 0.02 | 0.13 | ZCCHC24 |
| chr10:81,872,956-81,894,055 | q22.3 | Allelic Imbalance | 21099 | 42.86 | 17.78 | 25.08 | 0.02 | 0.13 | PLAC9 |
| chr10:86,263,931-86,333,402 | q23.1 | Allelic Imbalance | 69471 | 42.86 | 17.78 | 25.08 | 0.02 | 0.13 | CCSER2 |
| chr10:86,341,054-86,411,989 | q23.1 | Allelic Imbalance | 70935 | 42.86 | 17.78 | 25.08 | 0.02 | 0.13 |  |
| chr10:87,971,257-88,101,581 | q23.2 | Allelic Imbalance | 130324 | 42.86 | 17.78 | 25.08 | 0.02 | 0.13 | GRID1, MIR346 |
| chr10:88,525,460-88,552,733 | q23.2 | Allelic Imbalance | 27273 | 42.86 | 17.78 | 25.08 | 0.02 | 0.13 | BMPR1A |
| chr10:89,428,022-89,440,397 | q23.2 | Allelic Imbalance | 12375 | 42.86 | 17.78 | 25.08 | 0.02 | 0.13 | PAPSS2 |
| chr10:89,492,061-89,500,989 | q23.2 | Allelic Imbalance | 8928 | 42.86 | 17.78 | 25.08 | 0.02 | 0.13 | PAPSS2 |
| chr10:91,728,113-91,728,701 | q23.31 | Allelic Imbalance | 588 | 42.86 | 17.78 | 25.08 | 0.02 | 0.13 |  |
| chr10:92,073,985-92,094,560 | q23.31 | Allelic Imbalance | 20575 | 42.86 | 17.78 | 25.08 | 0.02 | 0.13 |  |
| chr10:92,759,099-92,851,783 | q23.31 | Allelic Imbalance | 92684 | 42.86 | 17.78 | 25.08 | 0.02 | 0.13 | LINC00502 |
| chr10:94,844,651-94,860,696 | q23.33 | Allelic Imbalance | 16045 | 42.86 | 17.78 | 25.08 | 0.02 | 0.13 |  |
| chr7:100,542,136-100,555,006 | q22.1 | Allelic Imbalance | 12870 | 57.14 | 31.11 | 26.03 | 0.02 | 0.13 |  |
| chr7:100,563,028-100,571,228 | q22.1 | Allelic Imbalance | 8200 | 59.52 | 33.33 | 26.19 | 0.02 | 0.13 | SERPINE1 |
| chr7:100,595,807-100,732,713 | q22.1 | Allelic Imbalance | 136906 | 59.52 | 33.33 | 26.19 | 0.02 | 0.13 | NAT16, MOGAT3, PLOD3, ZNHIT1, CLDN15, FIS1 |
| chr7:100,853,878-100,871,967 | q22.1 | Allelic Imbalance | 18089 | 57.14 | 31.11 | 26.03 | 0.02 | 0.13 | EMID2 |
| chr7:100,950,461-100,973,729 | q22.1 | Allelic Imbalance | 23268 | 59.52 | 33.33 | 26.19 | 0.02 | 0.13 | EMID2 |
| chr7:100,995,705-101,053,139 | q22.1 | Allelic Imbalance | 57434 | 59.52 | 33.33 | 26.19 | 0.02 | 0.13 | MYL10 |
| chr7:101,221,735-101,336,367 | q22.1 | Allelic Imbalance | 114632 | 59.52 | 33.33 | 26.19 | 0.02 | 0.13 | CUX1 |
| chr7:101,522,364-101,639,748 | q22.1 | Allelic Imbalance | 117384 | 59.52 | 33.33 | 26.19 | 0.02 | 0.13 | CUX1 |
| chr7:102,855,893-102,865,284 | q22.1 | Allelic Imbalance | 9391 | 42.86 | 17.78 | 25.08 | 0.02 | 0.13 | SLC26A5 |
| chr7:102,883,525-102,899,011 | q22.1 | Allelic Imbalance | 15486 | 42.86 | 17.78 | 25.08 | 0.02 | 0.13 |  |
| chr7:102,912,961-102,915,230 | q22.1 | Allelic Imbalance | 2269 | 42.86 | 17.78 | 25.08 | 0.02 | 0.13 | RELN |
| chr7:102,974,634-102,977,835 | q22.1 | Allelic Imbalance | 3201 | 42.86 | 17.78 | 25.08 | 0.02 | 0.13 | RELN |
| chr7:105,102,336-105,129,863 | q22.2 | Allelic Imbalance | 27527 | 57.14 | 31.11 | 26.03 | 0.02 | 0.13 | ATXN7L1 |
| chr7:105,690,428-105,728,345 | q22.2 | Allelic Imbalance | 37917 | 59.52 | 33.33 | 26.19 | 0.02 | 0.13 | NAMPT |
| chr7:106,001,484-106,118,167 | q22.3 | Allelic Imbalance | 116683 | 57.14 | 31.11 | 26.03 | 0.02 | 0.13 | CCDC71L |
| chr7:106,151,357-106,180,600 | q22.3 | Allelic Imbalance | 29243 | 57.14 | 31.11 | 26.03 | 0.02 | 0.13 |  |
| chr7:106,188,974-106,191,746 | q22.3 | Allelic Imbalance | 2772 | 59.52 | 33.33 | 26.19 | 0.02 | 0.13 |  |
| chr7:106,197,659-106,202,729 | q22.3 | Allelic Imbalance | 5070 | 59.52 | 33.33 | 26.19 | 0.02 | 0.13 |  |
| chr7:106,255,147-106,275,502 | q22.3 | Allelic Imbalance | 20355 | 59.52 | 33.33 | 26.19 | 0.02 | 0.13 |  |
| chr7:107,391,360-107,395,369 | q31.1 | Allelic Imbalance | 4009 | 57.14 | 31.11 | 26.03 | 0.02 | 0.13 | LAMB1 |
| chr7:107,430,340-107,441,815 | q31.1 | Allelic Imbalance | 11475 | 57.14 | 31.11 | 26.03 | 0.02 | 0.13 | LAMB1 |
| chr7:108,575,653-108,601,426 | q31.1 | Allelic Imbalance | 25773 | 59.52 | 33.33 | 26.19 | 0.02 | 0.13 |  |
| chr7:108,624,781-108,686,027 | q31.1 | Allelic Imbalance | 61246 | 57.14 | 31.11 | 26.03 | 0.02 | 0.13 |  |
| chr7:108,894,937-108,897,946 | q31.1 | Allelic Imbalance | 3009 | 57.14 | 31.11 | 26.03 | 0.02 | 0.13 |  |
| chr7:109,770,172-109,812,737 | q31.1 | Allelic Imbalance | 42565 | 57.14 | 31.11 | 26.03 | 0.02 | 0.13 |  |
| chr7:109,864,610-109,900,565 | q31.1 | Allelic Imbalance | 35955 | 57.14 | 31.11 | 26.03 | 0.02 | 0.13 |  |
| chr7:111,170,843-111,180,407 | q31.1 | Allelic Imbalance | 9564 | 57.14 | 31.11 | 26.03 | 0.02 | 0.13 | DOCK4 |
| chr7:113,010,824-113,109,535 | q31.1 | Allelic Imbalance | 98711 | 57.14 | 31.11 | 26.03 | 0.02 | 0.13 |  |
| chr7:116,592,568-116,646,629 | q31.2 | Allelic Imbalance | 54061 | 59.52 | 33.33 | 26.19 | 0.02 | 0.13 | ST7, ST7-OT3 |
| chr7:121,054,446-121,138,882 | q31.32 | Allelic Imbalance | 84436 | 66.67 | 40.00 | 26.67 | 0.02 | 0.13 |  |
| chr7:123,583,347-123,646,646 | q31.32 - q31.33 | Allelic Imbalance | 63299 | 57.14 | 31.11 | 26.03 | 0.02 | 0.13 |  |
| chr7:123,655,508-123,742,411 | q31.33 | Allelic Imbalance | 86903 | 57.14 | 31.11 | 26.03 | 0.02 | 0.13 |  |
| chr7:124,128,359-124,145,300 | q31.33 | Allelic Imbalance | 16941 | 57.14 | 31.11 | 26.03 | 0.02 | 0.13 |  |
| chr7:124,181,736-124,249,290 | q31.33 | Allelic Imbalance | 67554 | 57.14 | 31.11 | 26.03 | 0.02 | 0.13 | GPR37, LOC154872 |
| chr7:125,905,018-125,937,041 | q31.33 | Allelic Imbalance | 32023 | 57.14 | 31.11 | 26.03 | 0.02 | 0.13 | GRM8 |
| chr7:127,842,653-128,165,629 | q32.1 | Allelic Imbalance | 322976 | 66.67 | 40.00 | 26.67 | 0.02 | 0.13 | HILPDA, METTL2B, FLJ45340, FAM71F2, FAM71F1 |
| chr7:128,171,934-128,185,339 | q32.1 | Allelic Imbalance | 13405 | 66.67 | 40.00 | 26.67 | 0.02 | 0.13 | CALU |
| chr7:128,204,536-128,353,509 | q32.1 | Allelic Imbalance | 148973 | 66.67 | 40.00 | 26.67 | 0.02 | 0.13 | CCDC136, FLNC, ATP6V1F, LOC100130705, KCP |
| chr7:128,370,391-128,402,953 | q32.1 | Allelic Imbalance | 32562 | 66.67 | 40.00 | 26.67 | 0.02 | 0.13 | IRF5, TNPO3 |
| chr7:128,980,615-129,022,423 | q32.1 - q32.2 | Allelic Imbalance | 41808 | 64.29 | 37.78 | 26.51 | 0.02 | 0.13 |  |
| chr7:129,083,937-129,182,652 | q32.2 | Allelic Imbalance | 98715 | 64.29 | 37.78 | 26.51 | 0.02 | 0.13 | NRF1 |
| chr7:129,509,141-129,663,099 | q32.2 | Allelic Imbalance | 153958 | 66.67 | 40.00 | 26.67 | 0.02 | 0.13 | KLHDC10, TMEM209, C7orf45 |
| chr7:129,897,845-129,911,799 | q32.2 | Allelic Imbalance | 13954 | 59.52 | 33.33 | 26.19 | 0.02 | 0.13 |  |
| chr7:130,892,784-130,904,140 | q32.3 | Allelic Imbalance | 11356 | 64.29 | 37.78 | 26.51 | 0.02 | 0.13 |  |
| chr7:130,926,519-131,113,385 | q32.3 | Allelic Imbalance | 186866 | 64.29 | 37.78 | 26.51 | 0.02 | 0.13 |  |
| chr7:131,787,717-132,136,007 | q32.3 | Allelic Imbalance | 348290 | 64.29 | 37.78 | 26.51 | 0.02 | 0.13 | PLXNA4, FLJ40288, CHCHD3 |
| chr7:132,256,575-132,362,332 | q32.3 | Allelic Imbalance | 105757 | 66.67 | 40.00 | 26.67 | 0.02 | 0.13 | CHCHD3 |
| chr7:133,672,297-133,734,102 | q33 | Allelic Imbalance | 61805 | 66.67 | 40.00 | 26.67 | 0.02 | 0.13 |  |
| chr7:134,243,071-134,246,701 | q33 | Allelic Imbalance | 3630 | 57.14 | 31.11 | 26.03 | 0.02 | 0.13 | CALD1 |
| chr7:134,593,048-134,659,253 | q33 | Allelic Imbalance | 66205 | 64.29 | 37.78 | 26.51 | 0.02 | 0.13 | STRA8 |
| chr7:134,664,343-134,668,643 | q33 | Allelic Imbalance | 4300 | 66.67 | 40.00 | 26.67 | 0.02 | 0.13 |  |
| chr7:135,136,322-135,163,786 | q33 | Allelic Imbalance | 27464 | 64.29 | 37.78 | 26.51 | 0.02 | 0.13 |  |
| chr7:135,211,653-135,470,900 | q33 | Allelic Imbalance | 259247 | 64.29 | 37.78 | 26.51 | 0.02 | 0.13 | MTPN, LUZP6 |
| chr7:135,648,538-135,677,935 | q33 | Allelic Imbalance | 29397 | 66.67 | 40.00 | 26.67 | 0.02 | 0.13 |  |
| chr7:135,722,195-135,742,765 | q33 | Allelic Imbalance | 20570 | 64.29 | 37.78 | 26.51 | 0.02 | 0.13 |  |
| chr7:136,622,104-136,638,624 | q33 | Allelic Imbalance | 16520 | 64.29 | 37.78 | 26.51 | 0.02 | 0.13 | PTN |
| chr7:136,735,549-136,750,546 | q33 | Allelic Imbalance | 14997 | 66.67 | 40.00 | 26.67 | 0.02 | 0.13 | DGKI |
| chr7:136,999,861-137,036,914 | q33 | Allelic Imbalance | 37053 | 66.67 | 40.00 | 26.67 | 0.02 | 0.13 | DGKI |
| chr7:137,105,564-137,225,267 | q33 | Allelic Imbalance | 119703 | 64.29 | 37.78 | 26.51 | 0.02 | 0.13 | DGKI, CREB3L2 |
| chr7:138,406,865-138,754,015 | q34 | Allelic Imbalance | 347150 | 66.67 | 40.00 | 26.67 | 0.02 | 0.13 | ZC3HAV1, TTC26, UBN2, C7orf55, LUC7L2, C7orf55-LUC7L2, LOC100129148 |
| chr7:141,432,390-141,614,130 | q34 | Allelic Imbalance | 181740 | 57.14 | 31.11 | 26.03 | 0.02 | 0.13 | MGAM, LOC93432, MOXD2P, PRSS58 |
| chr7:143,423,544-143,438,132 | q35 | Allelic Imbalance | 14588 | 59.52 | 33.33 | 26.19 | 0.02 | 0.13 | OR2A12, OR2A2 |
| chr7:147,291,414-147,332,495 | q35 | Allelic Imbalance | 41081 | 59.52 | 33.33 | 26.19 | 0.02 | 0.13 | CNTNAP2, MIR548T |
| chr7:147,603,713-147,660,466 | q36.1 | Allelic Imbalance | 56753 | 64.29 | 37.78 | 26.51 | 0.02 | 0.13 | CNTNAP2, MIR548T |
| chr7:156,673,323-156,736,663 | q36.3 | Allelic Imbalance | 63340 | 59.52 | 33.33 | 26.19 | 0.02 | 0.13 | UBE3C |
| chr7:156,781,636-156,784,931 | q36.3 | Allelic Imbalance | 3295 | 59.52 | 33.33 | 26.19 | 0.02 | 0.13 |  |
| chr7:157,989,940-158,076,741 | q36.3 | Allelic Imbalance | 86801 | 57.14 | 31.11 | 26.03 | 0.02 | 0.13 | PTPRN2, MIR595 |
| chr7:68,648,664-68,824,822 | q11.22 | Allelic Imbalance | 176158 | 42.86 | 17.78 | 25.08 | 0.02 | 0.13 | AUTS2 |
| chr7:86,603,195-86,610,606 | q21.12 | Allelic Imbalance | 7411 | 42.86 | 17.78 | 25.08 | 0.02 | 0.13 |  |
| chr7:88,396,776-88,415,855 | q21.13 | Allelic Imbalance | 19079 | 42.86 | 17.78 | 25.08 | 0.02 | 0.13 | ZNF804B |
| chr7:88,829,987-88,869,448 | q21.13 | Allelic Imbalance | 39461 | 57.14 | 31.11 | 26.03 | 0.02 | 0.13 |  |
| chr7:88,893,075-88,953,791 | q21.13 | Allelic Imbalance | 60716 | 57.14 | 31.11 | 26.03 | 0.02 | 0.13 |  |
| chr7:89,479,875-89,490,864 | q21.13 | Allelic Imbalance | 10989 | 42.86 | 17.78 | 25.08 | 0.02 | 0.13 |  |
| chr7:89,512,503-89,524,349 | q21.13 | Allelic Imbalance | 11846 | 42.86 | 17.78 | 25.08 | 0.02 | 0.13 |  |
| chr7:89,526,931-89,531,650 | q21.13 | Allelic Imbalance | 4719 | 42.86 | 17.78 | 25.08 | 0.02 | 0.13 |  |
| chr7:90,665,855-90,669,833 | q21.13 | Allelic Imbalance | 3978 | 57.14 | 31.11 | 26.03 | 0.02 | 0.13 | CDK14 |
| chr7:91,018,505-91,024,349 | q21.2 | Allelic Imbalance | 5844 | 57.14 | 31.11 | 26.03 | 0.02 | 0.13 |  |
| chr7:91,045,738-91,046,085 | q21.2 | Allelic Imbalance | 347 | 57.14 | 31.11 | 26.03 | 0.02 | 0.13 |  |
| chr7:92,860,535-92,913,539 | q21.3 | Allelic Imbalance | 53004 | 59.52 | 33.33 | 26.19 | 0.02 | 0.13 | CALCR |
| chr7:92,916,949-92,917,618 | q21.3 | Allelic Imbalance | 669 | 59.52 | 33.33 | 26.19 | 0.02 | 0.13 | CALCR |
| chr7:95,265,303-95,280,936 | q21.3 | Allelic Imbalance | 15633 | 57.14 | 31.11 | 26.03 | 0.02 | 0.13 | DYNC1I1 |
| chr7:95,363,977-95,370,280 | q21.3 | Allelic Imbalance | 6303 | 57.14 | 31.11 | 26.03 | 0.02 | 0.13 | DYNC1I1 |
| chr7:95,400,169-95,451,192 | q21.3 | Allelic Imbalance | 51023 | 57.14 | 31.11 | 26.03 | 0.02 | 0.13 | DYNC1I1 |
| chr7:95,457,161-95,465,925 | q21.3 | Allelic Imbalance | 8764 | 57.14 | 31.11 | 26.03 | 0.02 | 0.13 | DYNC1I1 |
| chr7:95,913,460-95,922,745 | q21.3 | Allelic Imbalance | 9285 | 57.14 | 31.11 | 26.03 | 0.02 | 0.13 |  |
| chr7:95,957,599-95,972,578 | q21.3 | Allelic Imbalance | 14979 | 59.52 | 33.33 | 26.19 | 0.02 | 0.13 | C7orf76 |
| chr7:97,021,965-97,069,390 | q21.3 | Allelic Imbalance | 47425 | 59.52 | 33.33 | 26.19 | 0.02 | 0.13 |  |
| chr7:97,142,556-97,151,797 | q21.3 | Allelic Imbalance | 9241 | 57.14 | 31.11 | 26.03 | 0.02 | 0.13 |  |
| chr7:98,525,241-98,579,859 | q22.1 | Allelic Imbalance | 54618 | 42.86 | 17.78 | 25.08 | 0.02 | 0.13 | SMURF1 |
| chr7:126,992,452-127,023,438 | q32.1 | Allelic Imbalance | 30986 | 61.90 | 35.56 | 26.35 | 0.02 | 0.13 | GCC1, ARF5, FSCN3 |
| chr7:127,435,223-127,482,571 | q32.1 | Allelic Imbalance | 47348 | 61.90 | 35.56 | 26.35 | 0.02 | 0.13 | SND1, LRRC4 |
| chr7:130,286,279-130,356,225 | q32.3 | Allelic Imbalance | 69946 | 61.90 | 35.56 | 26.35 | 0.02 | 0.13 | FLJ43663 |
| chr7:130,549,164-130,830,162 | q32.3 | Allelic Imbalance | 280998 | 61.90 | 35.56 | 26.35 | 0.02 | 0.13 | MKLN1 |
| chr7:131,508,505-131,554,449 | q32.3 | Allelic Imbalance | 45944 | 61.90 | 35.56 | 26.35 | 0.02 | 0.13 | PLXNA4 |
| chr7:131,591,978-131,715,147 | q32.3 | Allelic Imbalance | 123169 | 61.90 | 35.56 | 26.35 | 0.02 | 0.13 | PLXNA4 |
| chr7:136,209,822-136,247,419 | q33 | Allelic Imbalance | 37597 | 61.90 | 35.56 | 26.35 | 0.02 | 0.13 | MIR490, CHRM2, LOC349160 |
| chr7:156,861,886-156,975,010 | q36.3 | Allelic Imbalance | 113124 | 61.90 | 35.56 | 26.35 | 0.02 | 0.13 | DNAJB6 |
| chr7:96,395,086-96,397,571 | q21.3 | Allelic Imbalance | 2485 | 61.90 | 35.56 | 26.35 | 0.02 | 0.13 |  |
| chr7:29,125,965-29,128,763 | p15.1 | CN Gain | 2798 | 85.71 | 11.11 | 74.60 | 0.00 | 0.00 | CPVL |
| chr7:21,866,745-21,922,352 | p15.3 | CN Gain | 55607 | 83.33 | 11.11 | 72.22 | 0.00 | 0.00 | DNAH11, CDCA7L |
| chr7:22,293,192-22,294,082 | p15.3 | CN Gain | 890 | 83.33 | 11.11 | 72.22 | 0.00 | 0.00 | RAPGEF5 |
| chr7:29,121,047-29,125,965 | p15.1 | CN Gain | 4918 | 85.71 | 13.33 | 72.38 | 0.00 | 0.00 | CPVL |
| chr7:29,128,763-29,219,824 | p15.1 | CN Gain | 91061 | 83.33 | 11.11 | 72.22 | 0.00 | 0.00 | CPVL, CHN2 |
| chr7:50,800,982-50,820,639 | p12.2 | CN Gain | 19657 | 78.57 | 6.67 | 71.90 | 0.00 | 0.00 | GRB10 |
| chr7:21,795,982-21,866,745 | p15.3 | CN Gain | 70763 | 83.33 | 13.33 | 70.00 | 0.00 | 0.00 | DNAH11 |
| chr7:21,922,352-22,005,453 | p15.3 | CN Gain | 83101 | 80.95 | 11.11 | 69.84 | 0.00 | 0.00 | CDCA7L |
| chr7:22,127,362-22,293,192 | p15.3 | CN Gain | 165830 | 83.33 | 13.33 | 70.00 | 0.00 | 0.00 | RAPGEF5 |
| chr7:22,294,082-22,725,211 | p15.3 | CN Gain | 431129 | 83.33 | 13.33 | 70.00 | 0.00 | 0.00 | RAPGEF5, STEAP1B, LOC100506178 |
| chr7:22,789,389-22,810,877 | p15.3 | CN Gain | 21488 | 83.33 | 13.33 | 70.00 | 0.00 | 0.00 |  |
| chr7:23,343,897-23,386,698 | p15.3 | CN Gain | 42801 | 80.95 | 11.11 | 69.84 | 0.00 | 0.00 | IGF2BP3 |
| chr7:24,731,441-24,736,860 | p15.3 | CN Gain | 5419 | 80.95 | 11.11 | 69.84 | 0.00 | 0.00 | DFNA5 |
| chr7:25,028,969-25,060,725 | p15.2 | CN Gain | 31756 | 80.95 | 11.11 | 69.84 | 0.00 | 0.00 |  |
| chr7:25,387,286-25,403,520 | p15.2 | CN Gain | 16234 | 83.33 | 13.33 | 70.00 | 0.00 | 0.00 |  |
| chr7:27,475,925-27,499,035 | p15.2 | CN Gain | 23110 | 83.33 | 13.33 | 70.00 | 0.00 | 0.00 |  |
| chr7:29,219,824-29,309,599 | p15.1 | CN Gain | 89775 | 80.95 | 11.11 | 69.84 | 0.00 | 0.00 | CHN2 |
| chr7:29,519,773-29,594,632 | p15.1 | CN Gain | 74859 | 78.57 | 8.89 | 69.68 | 0.00 | 0.00 | CHN2, PRR15 |
| chr7:29,875,001-29,964,685 | p15.1 | CN Gain | 89684 | 78.57 | 8.89 | 69.68 | 0.00 | 0.00 | WIPF3, SCRN1 |
| chr7:30,650,030-30,671,133 | p15.1 | CN Gain | 21103 | 83.33 | 13.33 | 70.00 | 0.00 | 0.00 | CRHR2 |
| chr7:33,669,276-33,716,469 | p14.3 | CN Gain | 47193 | 80.95 | 11.11 | 69.84 | 0.00 | 0.00 |  |
| chr7:43,198,089-43,571,582 | p14.1 - p13 | CN Gain | 373493 | 80.95 | 11.11 | 69.84 | 0.00 | 0.00 | HECW1, LOC100506895 |
| chr7:47,035,963-47,224,441 | p12.3 | CN Gain | 188478 | 78.57 | 8.89 | 69.68 | 0.00 | 0.00 |  |
| chr7:47,224,441-47,307,612 | p12.3 | CN Gain | 83171 | 80.95 | 11.11 | 69.84 | 0.00 | 0.00 | TNS3 |
| chr7:47,903,633-47,946,697 | p12.3 | CN Gain | 43064 | 80.95 | 11.11 | 69.84 | 0.00 | 0.00 | PKD1L1 |
| chr7:50,166,235-50,398,028 | p12.2 | CN Gain | 231793 | 69.05 | 2.22 | 66.83 | 0.00 | 0.00 | C7orf72, IKZF1 |
| chr7:50,780,963-50,800,982 | p12.2 | CN Gain | 20019 | 78.57 | 8.89 | 69.68 | 0.00 | 0.00 | GRB10 |
| chr7:50,820,639-50,833,031 | p12.2 | CN Gain | 12392 | 76.19 | 6.67 | 69.52 | 0.00 | 0.00 | GRB10 |
| chr7:50,833,031-50,898,976 | p12.2 | CN Gain | 65945 | 73.81 | 4.44 | 69.37 | 0.00 | 0.00 |  |
| chr7:55,189,699-55,219,961 | p11.2 | CN Gain | 30262 | 92.86 | 22.22 | 70.63 | 0.00 | 0.00 | EGFR, EGFR-AS1 |
| chr7:55,368,981-55,405,856 | p11.2 | CN Gain | 36875 | 80.95 | 11.11 | 69.84 | 0.00 | 0.00 | LANCL2 |
| chr7:55,454,264-55,462,651 | p11.2 | CN Gain | 8387 | 80.95 | 11.11 | 69.84 | 0.00 | 0.00 | LANCL2 |
| chr7:64,089,984-64,487,004 | q11.21 | CN Gain | 397020 | 78.57 | 8.89 | 69.68 | 0.00 | 0.00 | ERV3-1, CCT6P3, ZNF92 |
| chr7:50,025,684-50,166,235 | p12.2 | CN Gain | 140551 | 66.67 | 2.22 | 64.44 | 0.00 | 0.00 | ZPBP, C7orf72 |
| chr7:50,398,028-50,421,269 | p12.2 | CN Gain | 23241 | 66.67 | 2.22 | 64.44 | 0.00 | 0.00 | IKZF1 |
| chr7:50,898,976-51,078,764 | p12.2 - p12.1 | CN Gain | 179788 | 71.43 | 4.44 | 66.98 | 0.00 | 0.00 | COBL |
| chr7:55,188,127-55,189,699 | p11.2 | CN Gain | 1572 | 92.86 | 24.44 | 68.41 | 0.00 | 0.00 | EGFR |
| chr7:20,943,564-20,950,750 | p15.3 | CN Gain | 7186 | 78.57 | 11.11 | 67.46 | 0.00 | 0.00 |  |
| chr7:20,967,822-21,384,697 | p15.3 | CN Gain | 416875 | 80.95 | 13.33 | 67.62 | 0.00 | 0.00 |  |
| chr7:21,527,286-21,716,747 | p15.3 | CN Gain | 189461 | 78.57 | 11.11 | 67.46 | 0.00 | 0.00 | DNAH11 |
| chr7:21,745,840-21,767,381 | p15.3 | CN Gain | 21541 | 78.57 | 11.11 | 67.46 | 0.00 | 0.00 | DNAH11 |
| chr7:21,778,855-21,795,982 | p15.3 | CN Gain | 17127 | 80.95 | 13.33 | 67.62 | 0.00 | 0.00 | DNAH11 |
| chr7:22,005,453-22,070,472 | p15.3 | CN Gain | 65019 | 78.57 | 11.11 | 67.46 | 0.00 | 0.00 |  |
| chr7:22,079,539-22,127,362 | p15.3 | CN Gain | 47823 | 80.95 | 13.33 | 67.62 | 0.00 | 0.00 | RAPGEF5 |
| chr7:22,725,211-22,789,389 | p15.3 | CN Gain | 64178 | 83.33 | 15.56 | 67.78 | 0.00 | 0.00 | IL6 |
| chr7:22,810,877-22,902,076 | p15.3 | CN Gain | 91199 | 80.95 | 13.33 | 67.62 | 0.00 | 0.00 | TOMM7, SNORD93 |
| chr7:23,337,435-23,343,897 | p15.3 | CN Gain | 6462 | 80.95 | 13.33 | 67.62 | 0.00 | 0.00 | IGF2BP3 |
| chr7:23,386,698-23,492,907 | p15.3 | CN Gain | 106209 | 78.57 | 11.11 | 67.46 | 0.00 | 0.00 | IGF2BP3 |
| chr7:23,581,151-23,809,826 | p15.3 | CN Gain | 228675 | 78.57 | 11.11 | 67.46 | 0.00 | 0.00 | CLK2P, CCDC126, FAM221A, STK31 |
| chr7:24,567,103-24,731,441 | p15.3 | CN Gain | 164338 | 78.57 | 11.11 | 67.46 | 0.00 | 0.00 | MPP6, DFNA5 |
| chr7:24,736,860-25,028,969 | p15.3 - p15.2 | CN Gain | 292109 | 78.57 | 11.11 | 67.46 | 0.00 | 0.00 | DFNA5, OSBPL3 |
| chr7:25,060,725-25,258,723 | p15.2 | CN Gain | 197998 | 80.95 | 13.33 | 67.62 | 0.00 | 0.00 | CYCS, C7orf31, NPVF |
| chr7:25,278,822-25,387,286 | p15.2 | CN Gain | 108464 | 83.33 | 15.56 | 67.78 | 0.00 | 0.00 |  |
| chr7:25,403,520-26,057,683 | p15.2 | CN Gain | 654163 | 80.95 | 13.33 | 67.62 | 0.00 | 0.00 | MIR148A |
| chr7:26,957,527-27,103,266 | p15.2 | CN Gain | 145739 | 83.33 | 15.56 | 67.78 | 0.00 | 0.00 | HOXA1, HOTAIRM1 |
| chr7:27,453,036-27,475,925 | p15.2 | CN Gain | 22889 | 80.95 | 13.33 | 67.62 | 0.00 | 0.00 |  |
| chr7:29,078,904-29,121,047 | p15.1 | CN Gain | 42143 | 85.71 | 17.78 | 67.94 | 0.00 | 0.00 | CPVL |
| chr7:29,309,599-29,313,537 | p15.1 | CN Gain | 3938 | 78.57 | 11.11 | 67.46 | 0.00 | 0.00 | CHN2 |
| chr7:29,511,147-29,519,773 | p15.1 | CN Gain | 8626 | 76.19 | 8.89 | 67.30 | 0.00 | 0.00 | CHN2 |
| chr7:29,594,632-29,744,303 | p15.1 | CN Gain | 149671 | 76.19 | 8.89 | 67.30 | 0.00 | 0.00 | LOC646762, MIR550A3, ZNRF2P2, DPY19L2P3 |
| chr7:29,765,213-29,875,001 | p15.1 | CN Gain | 109788 | 76.19 | 8.89 | 67.30 | 0.00 | 0.00 | WIPF3 |
| chr7:30,630,134-30,637,019 | p15.1 | CN Gain | 6885 | 80.95 | 13.33 | 67.62 | 0.00 | 0.00 | GARS |
| chr7:30,671,133-30,681,115 | p15.1 | CN Gain | 9982 | 83.33 | 15.56 | 67.78 | 0.00 | 0.00 | CRHR2 |
| chr7:30,728,972-30,870,619 | p15.1 | CN Gain | 141647 | 83.33 | 15.56 | 67.78 | 0.00 | 0.00 | INMT, INMT-FAM188B, FAM188B |
| chr7:30,890,443-30,977,343 | p15.1 | CN Gain | 86900 | 85.71 | 17.78 | 67.94 | 0.00 | 0.00 | INMT-FAM188B, FAM188B, AQP1, GHRHR |
| chr7:31,104,374-31,106,049 | p15.1 | CN Gain | 1675 | 80.95 | 13.33 | 67.62 | 0.00 | 0.00 | ADCYAP1R1 |
| chr7:31,137,725-31,653,645 | p15.1 | CN Gain | 515920 | 78.57 | 11.11 | 67.46 | 0.00 | 0.00 | NEUROD6, CCDC129 |
| chr7:31,840,872-31,998,228 | p14.3 | CN Gain | 157356 | 78.57 | 11.11 | 67.46 | 0.00 | 0.00 | PDE1C |
| chr7:32,167,845-32,226,507 | p14.3 | CN Gain | 58662 | 76.19 | 8.89 | 67.30 | 0.00 | 0.00 | PDE1C |
| chr7:32,466,042-32,478,480 | p14.3 | CN Gain | 12438 | 76.19 | 8.89 | 67.30 | 0.00 | 0.00 |  |
| chr7:32,481,607-32,593,246 | p14.3 | CN Gain | 111639 | 78.57 | 11.11 | 67.46 | 0.00 | 0.00 | LSM5, AVL9, DPY19L1P1 |
| chr7:33,528,495-33,669,276 | p14.3 | CN Gain | 140781 | 78.57 | 11.11 | 67.46 | 0.00 | 0.00 | BBS9 |
| chr7:33,716,469-33,730,924 | p14.3 | CN Gain | 14455 | 78.57 | 11.11 | 67.46 | 0.00 | 0.00 |  |
| chr7:34,881,426-35,379,099 | p14.3 | CN Gain | 497673 | 78.57 | 11.11 | 67.46 | 0.00 | 0.00 | NPSR1, DPY19L1, DPY19L2P1, TBX20, LOC401324 |
| chr7:4,114,493-4,162,560 | p22.2 | CN Gain | 48067 | 76.19 | 8.89 | 67.30 | 0.00 | 0.00 | SDK1 |
| chr7:4,239,764-4,423,881 | p22.2 | CN Gain | 184117 | 76.19 | 8.89 | 67.30 | 0.00 | 0.00 | SDK1 |
| chr7:41,813,026-41,837,475 | p14.1 | CN Gain | 24449 | 76.19 | 8.89 | 67.30 | 0.00 | 0.00 |  |
| chr7:42,005,458-42,024,634 | p14.1 | CN Gain | 19176 | 78.57 | 11.11 | 67.46 | 0.00 | 0.00 | GLI3 |
| chr7:42,646,067-43,198,089 | p14.1 | CN Gain | 552022 | 78.57 | 11.11 | 67.46 | 0.00 | 0.00 | C7orf25, PSMA2, MRPL32, MIR3943, HECW1 |
| chr7:43,571,582-43,603,608 | p13 | CN Gain | 32026 | 80.95 | 13.33 | 67.62 | 0.00 | 0.00 | STK17A |
| chr7:46,766,095-47,035,963 | p12.3 | CN Gain | 269868 | 76.19 | 8.89 | 67.30 | 0.00 | 0.00 |  |
| chr7:47,636,066-47,785,480 | p12.3 | CN Gain | 149414 | 80.95 | 13.33 | 67.62 | 0.00 | 0.00 | C7orf65, LINC00525, PKD1L1 |
| chr7:47,852,293-47,903,633 | p12.3 | CN Gain | 51340 | 80.95 | 13.33 | 67.62 | 0.00 | 0.00 | PKD1L1 |
| chr7:47,946,697-48,485,285 | p12.3 | CN Gain | 538588 | 80.95 | 13.33 | 67.62 | 0.00 | 0.00 | PKD1L1, HUS1, SUN3, C7orf57, UPP1, ABCA13 |
| chr7:50,487,892-50,503,964 | p12.2 | CN Gain | 16072 | 69.05 | 4.44 | 64.60 | 0.00 | 0.00 | DDC |
| chr7:50,698,143-50,780,963 | p12.2 | CN Gain | 82820 | 76.19 | 8.89 | 67.30 | 0.00 | 0.00 | GRB10 |
| chr7:51,078,764-51,253,684 | p12.1 | CN Gain | 174920 | 69.05 | 4.44 | 64.60 | 0.00 | 0.00 | COBL |
| chr7:52,418,942-52,608,872 | p12.1 | CN Gain | 189930 | 69.05 | 4.44 | 64.60 | 0.00 | 0.00 |  |
| chr7:52,623,829-52,733,961 | p12.1 | CN Gain | 110132 | 69.05 | 4.44 | 64.60 | 0.00 | 0.00 |  |
| chr7:53,928,975-54,136,117 | p11.2 | CN Gain | 207142 | 76.19 | 8.89 | 67.30 | 0.00 | 0.00 |  |
| chr7:54,190,228-54,446,322 | p11.2 | CN Gain | 256094 | 78.57 | 11.11 | 67.46 | 0.00 | 0.00 | HPVC1 |
| chr7:54,446,322-54,649,547 | p11.2 | CN Gain | 203225 | 80.95 | 13.33 | 67.62 | 0.00 | 0.00 | VSTM2A, LOC285878 |
| chr7:55,280,332-55,368,981 | p11.2 | CN Gain | 88649 | 80.95 | 13.33 | 67.62 | 0.00 | 0.00 |  |
| chr7:55,405,856-55,454,264 | p11.2 | CN Gain | 48408 | 78.57 | 11.11 | 67.46 | 0.00 | 0.00 | LANCL2 |
| chr7:55,462,651-55,534,711 | p11.2 | CN Gain | 72060 | 80.95 | 13.33 | 67.62 | 0.00 | 0.00 | LANCL2, VOPP1 |
| chr7:55,612,319-55,633,578 | p11.2 | CN Gain | 21259 | 78.57 | 11.11 | 67.46 | 0.00 | 0.00 |  |
| chr7:63,984,562-64,089,984 | q11.21 | CN Gain | 105422 | 76.19 | 8.89 | 67.30 | 0.00 | 0.00 | ZNF273, ZNF117, ERV3-1 |
| chr7:64,487,004-64,513,633 | q11.21 | CN Gain | 26629 | 76.19 | 8.89 | 67.30 | 0.00 | 0.00 | ZNF92 |
| chr7:66,411,388-66,432,187 | q11.22 | CN Gain | 20799 | 78.57 | 11.11 | 67.46 | 0.00 | 0.00 | STAG3L4 |
| chr7:29,328,541-29,344,584 | p15.1 | CN Gain | 16043 | 73.81 | 8.89 | 64.92 | 0.00 | 0.00 | CHN2 |
| chr7:29,744,303-29,765,213 | p15.1 | CN Gain | 20910 | 73.81 | 8.89 | 64.92 | 0.00 | 0.00 |  |
| chr7:32,226,507-32,466,042 | p14.3 | CN Gain | 239535 | 73.81 | 8.89 | 64.92 | 0.00 | 0.00 | PDE1C, LOC100130673 |
| chr7:4,162,560-4,239,764 | p22.2 | CN Gain | 77204 | 73.81 | 8.89 | 64.92 | 0.00 | 0.00 | SDK1 |
| chr7:4,423,881-4,668,044 | p22.2 - p22.1 | CN Gain | 244163 | 73.81 | 8.89 | 64.92 | 0.00 | 0.00 |  |
| chr7:41,706,426-41,813,026 | p14.1 | CN Gain | 106600 | 73.81 | 8.89 | 64.92 | 0.00 | 0.00 | INHBA, INHBA-AS1 |
| chr7:46,698,158-46,766,095 | p12.3 | CN Gain | 67937 | 73.81 | 8.89 | 64.92 | 0.00 | 0.00 |  |
| chr7:48,967,936-49,094,070 | p12.3 | CN Gain | 126134 | 73.81 | 8.89 | 64.92 | 0.00 | 0.00 |  |
| chr7:53,918,629-53,928,975 | p11.2 | CN Gain | 10346 | 73.81 | 8.89 | 64.92 | 0.00 | 0.00 |  |
| chr7:55,796,838-55,862,308 | p11.2 | CN Gain | 65470 | 73.81 | 8.89 | 64.92 | 0.00 | 0.00 | 14-Sep |
| chr7:63,347,972-63,458,519 | q11.21 | CN Gain | 110547 | 73.81 | 8.89 | 64.92 | 0.00 | 0.00 | ZNF679, ZNF736 |
| chr7:63,954,741-63,984,562 | q11.21 | CN Gain | 29821 | 73.81 | 8.89 | 64.92 | 0.00 | 0.00 |  |
| chr7:64,513,633-64,727,417 | q11.21 | CN Gain | 213784 | 73.81 | 8.89 | 64.92 | 0.00 | 0.00 |  |
| chr7:49,094,070-49,824,191 | p12.3 - p12.2 | CN Gain | 730121 | 71.43 | 6.67 | 64.76 | 0.00 | 0.00 | VWC2 |
| chr7:51,317,819-51,355,238 | p12.1 | CN Gain | 37419 | 71.43 | 6.67 | 64.76 | 0.00 | 0.00 | COBL |
| chr7:52,258,119-52,418,942 | p12.1 | CN Gain | 160823 | 71.43 | 6.67 | 64.76 | 0.00 | 0.00 |  |
| chr7:53,286,535-53,476,633 | p12.1 | CN Gain | 190098 | 71.43 | 6.67 | 64.76 | 0.00 | 0.00 |  |
| chr7:53,698,754-53,918,629 | p12.1 - p11.2 | CN Gain | 219875 | 71.43 | 6.67 | 64.76 | 0.00 | 0.00 | FLJ45974 |
| chr7:21,716,747-21,745,840 | p15.3 | CN Gain | 29093 | 76.19 | 11.11 | 65.08 | 0.00 | 0.00 | DNAH11 |
| chr7:23,492,907-23,581,151 | p15.3 | CN Gain | 88244 | 76.19 | 11.11 | 65.08 | 0.00 | 0.00 | RPS2P32, TRA2A |
| chr7:24,305,877-24,567,103 | p15.3 | CN Gain | 261226 | 76.19 | 11.11 | 65.08 | 0.00 | 0.00 |  |
| chr7:29,313,537-29,328,541 | p15.1 | CN Gain | 15004 | 76.19 | 11.11 | 65.08 | 0.00 | 0.00 | CHN2 |
| chr7:29,504,719-29,511,147 | p15.1 | CN Gain | 6428 | 76.19 | 11.11 | 65.08 | 0.00 | 0.00 | CHN2 |
| chr7:31,653,645-31,782,540 | p15.1 | CN Gain | 128895 | 76.19 | 11.11 | 65.08 | 0.00 | 0.00 | CCDC129, PPP1R17, PDE1C |
| chr7:31,792,871-31,840,872 | p15.1 - p14.3 | CN Gain | 48001 | 76.19 | 11.11 | 65.08 | 0.00 | 0.00 | PDE1C |
| chr7:31,998,228-32,167,845 | p14.3 | CN Gain | 169617 | 76.19 | 11.11 | 65.08 | 0.00 | 0.00 | PDE1C |
| chr7:32,478,480-32,481,607 | p14.3 | CN Gain | 3127 | 76.19 | 11.11 | 65.08 | 0.00 | 0.00 |  |
| chr7:32,593,246-32,805,358 | p14.3 | CN Gain | 212112 | 76.19 | 11.11 | 65.08 | 0.00 | 0.00 | DPY19L1P1, ZNRF2P1, MIR550A2, MIR550B2, LOC401321 |
| chr7:33,506,101-33,528,495 | p14.3 | CN Gain | 22394 | 76.19 | 11.11 | 65.08 | 0.00 | 0.00 | BBS9 |
| chr7:33,730,924-33,783,542 | p14.3 | CN Gain | 52618 | 76.19 | 11.11 | 65.08 | 0.00 | 0.00 |  |
| chr7:34,824,456-34,881,426 | p14.3 | CN Gain | 56970 | 76.19 | 11.11 | 65.08 | 0.00 | 0.00 | NPSR1-AS1, NPSR1 |
| chr7:35,379,099-35,428,631 | p14.3 | CN Gain | 49532 | 76.19 | 11.11 | 65.08 | 0.00 | 0.00 | LOC401324 |
| chr7:35,591,637-35,610,059 | p14.3 - p14.2 | CN Gain | 18422 | 76.19 | 11.11 | 65.08 | 0.00 | 0.00 |  |
| chr7:36,854,706-36,898,293 | p14.2 | CN Gain | 43587 | 76.19 | 11.11 | 65.08 | 0.00 | 0.00 | ELMO1 |
| chr7:37,346,922-37,410,969 | p14.2 | CN Gain | 64047 | 76.19 | 11.11 | 65.08 | 0.00 | 0.00 | ELMO1 |
| chr7:4,066,475-4,114,493 | p22.2 | CN Gain | 48018 | 76.19 | 11.11 | 65.08 | 0.00 | 0.00 | SDK1 |
| chr7:41,837,475-42,005,458 | p14.1 | CN Gain | 167983 | 76.19 | 11.11 | 65.08 | 0.00 | 0.00 | GLI3 |
| chr7:42,024,634-42,040,820 | p14.1 | CN Gain | 16186 | 76.19 | 11.11 | 65.08 | 0.00 | 0.00 | GLI3 |
| chr7:42,128,394-42,646,067 | p14.1 | CN Gain | 517673 | 76.19 | 11.11 | 65.08 | 0.00 | 0.00 | GLI3 |
| chr7:48,663,685-48,838,951 | p12.3 | CN Gain | 175266 | 76.19 | 11.11 | 65.08 | 0.00 | 0.00 |  |
| chr7:54,136,117-54,190,228 | p11.2 | CN Gain | 54111 | 76.19 | 11.11 | 65.08 | 0.00 | 0.00 |  |
| chr7:55,219,961-55,221,989 | p11.2 | CN Gain | 2028 | 88.10 | 22.22 | 65.87 | 0.00 | 0.00 | EGFR, EGFR-AS1 |
| chr7:55,633,578-55,649,706 | p11.2 | CN Gain | 16128 | 76.19 | 11.11 | 65.08 | 0.00 | 0.00 |  |
| chr7:65,284,566-66,411,388 | q11.21 - q11.22 | CN Gain | 1126822 | 76.19 | 11.11 | 65.08 | 0.00 | 0.00 | TPST1, LINC00174, LOC493754, KCTD7, RABGEF1, GTF2IRD1P1, TMEM248, SBDS, MIR4650-1, MIR4650-2, TYW1, PMS2P4, STAG3L4 |
| chr7:66,432,187-66,491,350 | q11.22 | CN Gain | 59163 | 76.19 | 11.11 | 65.08 | 0.00 | 0.00 |  |
| chr7:49,828,091-50,025,684 | p12.2 | CN Gain | 197593 | 66.67 | 4.44 | 62.22 | 0.00 | 0.00 | VWC2, ZPBP |
| chr7:50,421,269-50,487,892 | p12.2 | CN Gain | 66623 | 66.67 | 4.44 | 62.22 | 0.00 | 0.00 | IKZF1, FIGNL1 |
| chr7:52,608,872-52,623,829 | p12.1 | CN Gain | 14957 | 66.67 | 4.44 | 62.22 | 0.00 | 0.00 |  |
| chr7:52,733,961-52,746,651 | p12.1 | CN Gain | 12690 | 66.67 | 4.44 | 62.22 | 0.00 | 0.00 |  |
| chr7:20,950,750-20,967,822 | p15.3 | CN Gain | 17072 | 78.57 | 13.33 | 65.24 | 0.00 | 0.00 |  |
| chr7:21,384,697-21,522,611 | p15.3 | CN Gain | 137914 | 80.95 | 15.56 | 65.40 | 0.00 | 0.00 | SP4 |
| chr7:21,767,381-21,778,855 | p15.3 | CN Gain | 11474 | 78.57 | 13.33 | 65.24 | 0.00 | 0.00 | DNAH11 |
| chr7:22,070,472-22,079,539 | p15.3 | CN Gain | 9067 | 78.57 | 13.33 | 65.24 | 0.00 | 0.00 |  |
| chr7:22,902,076-22,919,024 | p15.3 | CN Gain | 16948 | 80.95 | 15.56 | 65.40 | 0.00 | 0.00 |  |
| chr7:23,265,757-23,337,435 | p15.3 | CN Gain | 71678 | 80.95 | 15.56 | 65.40 | 0.00 | 0.00 | GPNMB, MALSU1, IGF2BP3 |
| chr7:25,258,723-25,278,822 | p15.2 | CN Gain | 20099 | 80.95 | 15.56 | 65.40 | 0.00 | 0.00 |  |
| chr7:26,057,683-26,170,659 | p15.2 | CN Gain | 112976 | 80.95 | 15.56 | 65.40 | 0.00 | 0.00 | NFE2L3 |
| chr7:26,641,238-26,832,500 | p15.2 | CN Gain | 191262 | 80.95 | 15.56 | 65.40 | 0.00 | 0.00 | C7orf71, SKAP2 |
| chr7:26,902,553-26,957,527 | p15.2 | CN Gain | 54974 | 83.33 | 17.78 | 65.56 | 0.00 | 0.00 |  |
| chr7:27,103,266-27,206,560 | p15.2 | CN Gain | 103294 | 83.33 | 17.78 | 65.56 | 0.00 | 0.00 | HOTAIRM1, HOXA2, HOXA3, HOXA4, HOXA5, HOXA6, HOXA-AS3, HOXA7, HOXA9, MIR196B, HOXA-AS4, HOXA10-HOXA9, HOXA10, HOXA11, HOXA11-AS, HOXA13 |
| chr7:27,240,938-27,453,036 | p15.2 | CN Gain | 212098 | 78.57 | 13.33 | 65.24 | 0.00 | 0.00 | EVX1 |
| chr7:27,499,035-27,513,350 | p15.2 | CN Gain | 14315 | 83.33 | 17.78 | 65.56 | 0.00 | 0.00 |  |
| chr7:28,333,198-28,453,828 | p15.1 | CN Gain | 120630 | 80.95 | 15.56 | 65.40 | 0.00 | 0.00 | CREB5 |
| chr7:29,067,262-29,078,904 | p15.1 | CN Gain | 11642 | 83.33 | 17.78 | 65.56 | 0.00 | 0.00 | CPVL |
| chr7:29,964,685-30,069,920 | p15.1 | CN Gain | 105235 | 78.57 | 13.33 | 65.24 | 0.00 | 0.00 | SCRN1, FKBP14, PLEKHA8 |
| chr7:30,399,028-30,630,134 | p15.1 | CN Gain | 231106 | 78.57 | 13.33 | 65.24 | 0.00 | 0.00 | NOD1, GGCT, LOC401320, GARS |
| chr7:30,637,019-30,650,030 | p15.1 | CN Gain | 13011 | 78.57 | 13.33 | 65.24 | 0.00 | 0.00 | GARS |
| chr7:30,681,115-30,682,833 | p15.1 | CN Gain | 1718 | 83.33 | 17.78 | 65.56 | 0.00 | 0.00 | CRHR2 |
| chr7:30,720,574-30,728,972 | p15.1 | CN Gain | 8398 | 83.33 | 17.78 | 65.56 | 0.00 | 0.00 |  |
| chr7:30,870,619-30,890,443 | p15.1 | CN Gain | 19824 | 83.33 | 17.78 | 65.56 | 0.00 | 0.00 | INMT-FAM188B, FAM188B |
| chr7:30,977,343-31,088,546 | p15.1 | CN Gain | 111203 | 85.71 | 20.00 | 65.71 | 0.00 | 0.00 | GHRHR, ADCYAP1R1 |
| chr7:31,088,546-31,098,328 | p15.1 | CN Gain | 9782 | 83.33 | 17.78 | 65.56 | 0.00 | 0.00 | ADCYAP1R1 |
| chr7:31,098,328-31,104,374 | p15.1 | CN Gain | 6046 | 80.95 | 15.56 | 65.40 | 0.00 | 0.00 | ADCYAP1R1 |
| chr7:31,106,049-31,137,725 | p15.1 | CN Gain | 31676 | 78.57 | 13.33 | 65.24 | 0.00 | 0.00 | ADCYAP1R1 |
| chr7:37,287,898-37,342,252 | p14.2 | CN Gain | 54354 | 78.57 | 13.33 | 65.24 | 0.00 | 0.00 | ELMO1 |
| chr7:4,668,044-4,686,706 | p22.1 | CN Gain | 18662 | 78.57 | 13.33 | 65.24 | 0.00 | 0.00 |  |
| chr7:43,603,608-43,880,348 | p13 | CN Gain | 276740 | 80.95 | 15.56 | 65.40 | 0.00 | 0.00 | STK17A, COA1, BLVRA, MRPS24, URGCP-MRPS24 |
| chr7:47,307,612-47,436,662 | p12.3 | CN Gain | 129050 | 80.95 | 15.56 | 65.40 | 0.00 | 0.00 | TNS3 |
| chr7:47,611,263-47,636,066 | p12.3 | CN Gain | 24803 | 80.95 | 15.56 | 65.40 | 0.00 | 0.00 |  |
| chr7:47,785,480-47,852,293 | p12.3 | CN Gain | 66813 | 80.95 | 15.56 | 65.40 | 0.00 | 0.00 | C7orf69, PKD1L1 |
| chr7:48,485,285-48,663,685 | p12.3 | CN Gain | 178400 | 78.57 | 13.33 | 65.24 | 0.00 | 0.00 | ABCA13 |
| chr7:54,649,547-54,663,589 | p11.2 | CN Gain | 14042 | 80.95 | 15.56 | 65.40 | 0.00 | 0.00 |  |
| chr7:55,260,487-55,280,332 | p11.2 | CN Gain | 19845 | 80.95 | 15.56 | 65.40 | 0.00 | 0.00 |  |
| chr7:55,534,711-55,612,319 | p11.2 | CN Gain | 77608 | 78.57 | 13.33 | 65.24 | 0.00 | 0.00 | VOPP1 |
| chr7:69,774,644-69,935,922 | q11.22 | CN Gain | 161278 | 78.57 | 13.33 | 65.24 | 0.00 | 0.00 | AUTS2 |
| chr7:71,789,465-71,947,941 | q11.22 - q11.23 | CN Gain | 158476 | 78.57 | 13.33 | 65.24 | 0.00 | 0.00 | TYW1B, MIR4650-1, MIR4650-2, SBDSP1 |
| chr7:55,185,130-55,188,127 | p11.2 | CN Gain | 2997 | 92.86 | 28.89 | 63.97 | 0.00 | 0.00 | EGFR |
| chr7:23,809,826-23,823,575 | p15.3 | CN Gain | 13749 | 71.43 | 8.89 | 62.54 | 0.00 | 0.00 | STK31 |
| chr7:29,344,584-29,362,209 | p15.1 | CN Gain | 17625 | 71.43 | 8.89 | 62.54 | 0.00 | 0.00 | CHN2 |
| chr7:3,178,637-3,251,938 | p22.2 | CN Gain | 73301 | 71.43 | 8.89 | 62.54 | 0.00 | 0.00 |  |
| chr7:41,342,956-41,706,426 | p14.1 | CN Gain | 363470 | 71.43 | 8.89 | 62.54 | 0.00 | 0.00 | INHBA, INHBA-AS1 |
| chr7:46,687,573-46,698,158 | p12.3 | CN Gain | 10585 | 71.43 | 8.89 | 62.54 | 0.00 | 0.00 |  |
| chr7:51,253,684-51,317,819 | p12.1 | CN Gain | 64135 | 69.05 | 6.67 | 62.38 | 0.00 | 0.00 | COBL |
| chr7:51,355,238-51,495,843 | p12.1 | CN Gain | 140605 | 69.05 | 6.67 | 62.38 | 0.00 | 0.00 |  |
| chr7:51,508,079-51,666,063 | p12.1 | CN Gain | 157984 | 69.05 | 6.67 | 62.38 | 0.00 | 0.00 |  |
| chr7:51,863,860-52,258,119 | p12.1 | CN Gain | 394259 | 69.05 | 6.67 | 62.38 | 0.00 | 0.00 |  |
| chr7:53,143,429-53,286,535 | p12.1 | CN Gain | 143106 | 69.05 | 6.67 | 62.38 | 0.00 | 0.00 |  |
| chr7:53,476,633-53,632,163 | p12.1 | CN Gain | 155530 | 69.05 | 6.67 | 62.38 | 0.00 | 0.00 |  |
| chr7:53,665,964-53,698,754 | p12.1 | CN Gain | 32790 | 69.05 | 6.67 | 62.38 | 0.00 | 0.00 | FLJ45974 |
| chr7:55,706,234-55,796,838 | p11.2 | CN Gain | 90604 | 71.43 | 8.89 | 62.54 | 0.00 | 0.00 | FKBP9L |
| chr7:55,862,308-55,904,456 | p11.2 | CN Gain | 42148 | 71.43 | 8.89 | 62.54 | 0.00 | 0.00 | 14-Sep |
| chr7:56,109,418-56,217,775 | p11.2 | CN Gain | 108357 | 71.43 | 8.89 | 62.54 | 0.00 | 0.00 | SUMF2, PHKG1, CHCHD2, NUPR1L |
| chr7:62,151,187-62,166,219 | q11.21 | CN Gain | 15032 | 71.43 | 8.89 | 62.54 | 0.00 | 0.00 |  |
| chr7:62,282,415-62,489,565 | q11.21 | CN Gain | 207150 | 71.43 | 8.89 | 62.54 | 0.00 | 0.00 | ZNF733P, LOC100287704, LOC100287834 |
| chr7:63,244,003-63,347,972 | q11.21 | CN Gain | 103969 | 71.43 | 8.89 | 62.54 | 0.00 | 0.00 | ZNF735, ZNF679 |
| chr7:63,458,519-63,954,741 | q11.21 | CN Gain | 496222 | 71.43 | 8.89 | 62.54 | 0.00 | 0.00 | LOC649395, ZNF680, LOC641746, ZNF107, ZNF138 |
| chr7:50,503,964-50,541,066 | p12.2 | CN Gain | 37102 | 64.29 | 4.44 | 59.84 | 0.00 | 0.00 | DDC |
| chr7:52,746,651-52,778,800 | p12.1 | CN Gain | 32149 | 64.29 | 4.44 | 59.84 | 0.00 | 0.00 |  |
| chr7:20,697,648-20,703,206 | p15.3 | CN Gain | 5558 | 73.81 | 11.11 | 62.70 | 0.00 | 0.00 | ABCB5 |
| chr7:20,858,000-20,943,564 | p15.3 | CN Gain | 85564 | 73.81 | 11.11 | 62.70 | 0.00 | 0.00 |  |
| chr7:29,485,324-29,504,719 | p15.1 | CN Gain | 19395 | 73.81 | 11.11 | 62.70 | 0.00 | 0.00 | CHN2 |
| chr7:3,905,150-4,066,475 | p22.2 | CN Gain | 161325 | 73.81 | 11.11 | 62.70 | 0.00 | 0.00 | SDK1 |
| chr7:31,782,540-31,792,871 | p15.1 | CN Gain | 10331 | 73.81 | 11.11 | 62.70 | 0.00 | 0.00 | PDE1C |
| chr7:32,805,358-33,506,101 | p14.3 | CN Gain | 700743 | 73.81 | 11.11 | 62.70 | 0.00 | 0.00 | KBTBD2, RP9P, FKBP9, NT5C3, RP9, BBS9 |
| chr7:33,922,058-34,824,456 | p14.3 | CN Gain | 902398 | 73.81 | 11.11 | 62.70 | 0.00 | 0.00 | BMPER, NPSR1-AS1, NPSR1 |
| chr7:35,428,631-35,591,637 | p14.3 | CN Gain | 163006 | 73.81 | 11.11 | 62.70 | 0.00 | 0.00 |  |
| chr7:36,544,349-36,854,706 | p14.2 | CN Gain | 310357 | 73.81 | 11.11 | 62.70 | 0.00 | 0.00 | AOAH-IT1, AOAH |
| chr7:37,410,969-37,920,941 | p14.2 - p14.1 | CN Gain | 509972 | 73.81 | 11.11 | 62.70 | 0.00 | 0.00 | ELMO1, GPR141, NME8, SFRP4 |
| chr7:38,451,422-38,459,202 | p14.1 | CN Gain | 7780 | 73.81 | 11.11 | 62.70 | 0.00 | 0.00 | AMPH |
| chr7:40,890,703-41,329,443 | p14.1 | CN Gain | 438740 | 73.81 | 11.11 | 62.70 | 0.00 | 0.00 |  |
| chr7:42,040,820-42,128,394 | p14.1 | CN Gain | 87574 | 73.81 | 11.11 | 62.70 | 0.00 | 0.00 | GLI3 |
| chr7:48,838,951-48,967,936 | p12.3 | CN Gain | 128985 | 73.81 | 11.11 | 62.70 | 0.00 | 0.00 | CDC14C |
| chr7:50,648,822-50,660,700 | p12.2 | CN Gain | 11878 | 73.81 | 11.11 | 62.70 | 0.00 | 0.00 | GRB10 |
| chr7:55,649,706-55,657,954 | p11.2 | CN Gain | 8248 | 73.81 | 11.11 | 62.70 | 0.00 | 0.00 |  |
| chr7:6,576,394-6,679,162 | p22.1 | CN Gain | 102768 | 73.81 | 11.11 | 62.70 | 0.00 | 0.00 | ZDHHC4, C7orf26, ZNF853 |
| chr7:64,727,417-65,284,566 | q11.21 | CN Gain | 557149 | 73.81 | 11.11 | 62.70 | 0.00 | 0.00 | INTS4L2, LOC441242, SNORA22, CCT6P1, VKORC1L1, GUSB, ASL, CRCP |
| chr7:66,491,350-66,718,559 | q11.22 | CN Gain | 227209 | 73.81 | 11.11 | 62.70 | 0.00 | 0.00 |  |
| chr7:33,783,542-33,824,476 | p14.3 | CN Gain | 40934 | 76.19 | 13.33 | 62.86 | 0.00 | 0.00 |  |
| chr7:35,610,059-35,621,552 | p14.2 | CN Gain | 11493 | 76.19 | 13.33 | 62.86 | 0.00 | 0.00 |  |
| chr7:36,898,293-37,287,898 | p14.2 | CN Gain | 389605 | 76.19 | 13.33 | 62.86 | 0.00 | 0.00 | MIR1200, ELMO1 |
| chr7:37,342,252-37,346,922 | p14.2 | CN Gain | 4670 | 76.19 | 13.33 | 62.86 | 0.00 | 0.00 | ELMO1 |
| chr7:50,660,700-50,666,845 | p12.2 | CN Gain | 6145 | 76.19 | 13.33 | 62.86 | 0.00 | 0.00 | GRB10 |
| chr7:50,672,637-50,698,143 | p12.2 | CN Gain | 25506 | 76.19 | 13.33 | 62.86 | 0.00 | 0.00 | GRB10 |
| chr7:54,767,240-54,830,301 | p11.2 | CN Gain | 63061 | 85.71 | 22.22 | 63.49 | 0.00 | 0.00 | SEC61G |
| chr7:55,221,989-55,229,478 | p11.2 | CN Gain | 7489 | 85.71 | 22.22 | 63.49 | 0.00 | 0.00 | EGFR, EGFR-AS1 |
| chr7:69,661,863-69,774,644 | q11.22 | CN Gain | 112781 | 76.19 | 13.33 | 62.86 | 0.00 | 0.00 | AUTS2 |
| chr7:69,935,922-70,130,243 | q11.22 | CN Gain | 194321 | 76.19 | 13.33 | 62.86 | 0.00 | 0.00 |  |
| chr7:55,180,181-55,185,130 | p11.2 | CN Gain | 4949 | 92.86 | 31.11 | 61.75 | 0.00 | 0.00 | EGFR |
| chr7:21,522,611-21,527,286 | p15.3 | CN Gain | 4675 | 78.57 | 15.56 | 63.02 | 0.00 | 0.00 |  |
| chr7:26,170,659-26,182,792 | p15.2 | CN Gain | 12133 | 78.57 | 15.56 | 63.02 | 0.00 | 0.00 | NFE2L3 |
| chr7:26,569,859-26,641,238 | p15.2 | CN Gain | 71379 | 78.57 | 15.56 | 63.02 | 0.00 | 0.00 |  |
| chr7:27,208,089-27,240,938 | p15.2 | CN Gain | 32849 | 78.57 | 15.56 | 63.02 | 0.00 | 0.00 | HOTTIP |
| chr7:28,453,828-28,467,257 | p15.1 | CN Gain | 13429 | 78.57 | 15.56 | 63.02 | 0.00 | 0.00 | CREB5 |
| chr7:29,065,632-29,067,262 | p15.1 | CN Gain | 1630 | 83.33 | 20.00 | 63.33 | 0.00 | 0.00 | CPVL |
| chr7:30,069,920-30,151,713 | p15.1 | CN Gain | 81793 | 78.57 | 15.56 | 63.02 | 0.00 | 0.00 | PLEKHA8, C7orf41 |
| chr7:30,238,804-30,399,028 | p15.1 | CN Gain | 160224 | 78.57 | 15.56 | 63.02 | 0.00 | 0.00 | MIR550A1, MIR550B1, ZNRF2, DKFZP586I1420 |
| chr7:30,682,833-30,720,574 | p15.1 | CN Gain | 37741 | 83.33 | 20.00 | 63.33 | 0.00 | 0.00 | CRHR2 |
| chr7:38,294,222-38,309,765 | p14.1 | CN Gain | 15543 | 83.33 | 20.00 | 63.33 | 0.00 | 0.00 |  |
| chr7:4,686,706-4,832,740 | p22.1 | CN Gain | 146034 | 78.57 | 15.56 | 63.02 | 0.00 | 0.00 | FOXK1, AP5Z1, MIR4656, RADIL |
| chr7:71,947,941-71,961,223 | q11.23 | CN Gain | 13282 | 78.57 | 15.56 | 63.02 | 0.00 | 0.00 |  |
| chr7:2,922,117-2,943,775 | p22.2 | CN Gain | 21658 | 80.95 | 17.78 | 63.17 | 0.00 | 0.00 | CARD11 |
| chr7:22,919,024-23,265,757 | p15.3 | CN Gain | 346733 | 80.95 | 17.78 | 63.17 | 0.00 | 0.00 | FAM126A, KLHL7-AS1, KLHL7, NUPL2, GPNMB |
| chr7:26,832,500-26,902,553 | p15.2 | CN Gain | 70053 | 80.95 | 17.78 | 63.17 | 0.00 | 0.00 | SKAP2 |
| chr7:28,238,603-28,333,198 | p15.1 | CN Gain | 94595 | 80.95 | 17.78 | 63.17 | 0.00 | 0.00 | JAZF1-AS1, CREB5 |
| chr7:38,289,333-38,294,222 | p14.1 | CN Gain | 4889 | 80.95 | 17.78 | 63.17 | 0.00 | 0.00 |  |
| chr7:38,325,095-38,349,942 | p14.1 | CN Gain | 24847 | 80.95 | 17.78 | 63.17 | 0.00 | 0.00 | LOC100506776 |
| chr7:43,880,348-44,307,448 | p13 | CN Gain | 427100 | 80.95 | 17.78 | 63.17 | 0.00 | 0.00 | URGCP-MRPS24, URGCP, UBE2D4, POLR2J4, SPDYE1, RASA4CP, FLJ35390, DBNL, PGAM2, POLM, AEBP1, MIR4649, POLD2, MYL7, GCK, YKT6, CAMK2B |
| chr7:47,436,662-47,611,263 | p12.3 | CN Gain | 174601 | 80.95 | 17.78 | 63.17 | 0.00 | 0.00 | TNS3 |
| chr7:54,663,589-54,745,335 | p11.2 | CN Gain | 81746 | 80.95 | 17.78 | 63.17 | 0.00 | 0.00 |  |
| chr7:55,259,517-55,260,487 | p11.2 | CN Gain | 970 | 80.95 | 17.78 | 63.17 | 0.00 | 0.00 |  |
| chr7:49,824,191-49,828,091 | p12.2 | CN Gain | 3900 | 66.67 | 6.67 | 60.00 | 0.00 | 0.00 | VWC2 |
| chr7:50,608,122-50,626,309 | p12.2 | CN Gain | 18187 | 66.67 | 6.67 | 60.00 | 0.00 | 0.00 | GRB10 |
| chr7:51,495,843-51,508,079 | p12.1 | CN Gain | 12236 | 66.67 | 6.67 | 60.00 | 0.00 | 0.00 |  |
| chr7:51,666,063-51,863,860 | p12.1 | CN Gain | 197797 | 66.67 | 6.67 | 60.00 | 0.00 | 0.00 |  |
| chr7:53,071,341-53,143,429 | p12.1 | CN Gain | 72088 | 66.67 | 6.67 | 60.00 | 0.00 | 0.00 | POM121L12 |
| chr7:53,632,163-53,665,964 | p12.1 | CN Gain | 33801 | 66.67 | 6.67 | 60.00 | 0.00 | 0.00 |  |
| chr7:29,362,209-29,369,735 | p15.1 | CN Gain | 7526 | 69.05 | 8.89 | 60.16 | 0.00 | 0.00 | CHN2 |
| chr7:3,251,938-3,552,124 | p22.2 | CN Gain | 300186 | 69.05 | 8.89 | 60.16 | 0.00 | 0.00 | SDK1 |
| chr7:46,641,581-46,687,573 | p12.3 | CN Gain | 45992 | 69.05 | 8.89 | 60.16 | 0.00 | 0.00 |  |
| chr7:55,904,456-56,109,418 | p11.2 | CN Gain | 204962 | 69.05 | 8.89 | 60.16 | 0.00 | 0.00 | ZNF713, MRPS17, GBAS, PSPH, CCT6A, SNORA15, SUMF2 |
| chr7:56,217,775-56,548,662 | p11.2 | CN Gain | 330887 | 69.05 | 8.89 | 60.16 | 0.00 | 0.00 | LOC650226, DKFZp434L192 |
| chr7:62,166,219-62,282,415 | q11.21 | CN Gain | 116196 | 69.05 | 8.89 | 60.16 | 0.00 | 0.00 |  |
| chr7:62,489,565-63,244,003 | q11.21 | CN Gain | 754438 | 69.05 | 8.89 | 60.16 | 0.00 | 0.00 | LOC100287834, MIR4283-1, MIR4283-2, LOC100506050, ZNF727 |
| chr7:50,541,066-50,608,122 | p12.2 | CN Gain | 67056 | 61.90 | 4.44 | 57.46 | 0.00 | 0.00 | DDC, LOC100129427 |
| chr7:52,778,800-52,797,666 | p12.1 | CN Gain | 18866 | 61.90 | 4.44 | 57.46 | 0.00 | 0.00 |  |
| chr7:52,963,968-52,972,637 | p12.1 | CN Gain | 8669 | 61.90 | 4.44 | 57.46 | 0.00 | 0.00 |  |
| chr7:20,703,206-20,858,000 | p15.3 | CN Gain | 154794 | 71.43 | 11.11 | 60.32 | 0.00 | 0.00 | ABCB5, SP8, RPL23P8 |
| chr7:24,278,536-24,305,877 | p15.3 | CN Gain | 27341 | 71.43 | 11.11 | 60.32 | 0.00 | 0.00 | NPY |
| chr7:29,453,408-29,485,324 | p15.1 | CN Gain | 31916 | 71.43 | 11.11 | 60.32 | 0.00 | 0.00 | CHN2 |
| chr7:3,011,400-3,178,637 | p22.2 | CN Gain | 167237 | 71.43 | 11.11 | 60.32 | 0.00 | 0.00 | CARD11 |
| chr7:3,644,011-3,905,150 | p22.2 | CN Gain | 261139 | 71.43 | 11.11 | 60.32 | 0.00 | 0.00 | SDK1 |
| chr7:38,459,202-38,513,254 | p14.1 | CN Gain | 54052 | 71.43 | 11.11 | 60.32 | 0.00 | 0.00 | AMPH |
| chr7:38,910,686-39,280,377 | p14.1 | CN Gain | 369691 | 71.43 | 11.11 | 60.32 | 0.00 | 0.00 | VPS41, POU6F2 |
| chr7:40,853,139-40,890,703 | p14.1 | CN Gain | 37564 | 71.43 | 11.11 | 60.32 | 0.00 | 0.00 | C7orf10 |
| chr7:41,329,443-41,342,956 | p14.1 | CN Gain | 13513 | 71.43 | 11.11 | 60.32 | 0.00 | 0.00 |  |
| chr7:50,626,309-50,648,822 | p12.2 | CN Gain | 22513 | 71.43 | 11.11 | 60.32 | 0.00 | 0.00 | GRB10 |
| chr7:55,657,954-55,706,234 | p11.2 | CN Gain | 48280 | 71.43 | 11.11 | 60.32 | 0.00 | 0.00 |  |
| chr7:56,647,884-56,717,793 | p11.2 | CN Gain | 69909 | 71.43 | 11.11 | 60.32 | 0.00 | 0.00 |  |
| chr7:56,940,786-57,307,306 | p11.2 | CN Gain | 366520 | 71.43 | 11.11 | 60.32 | 0.00 | 0.00 | MIR4283-1, MIR4283-2, ZNF479, GUSBP10 |
| chr7:6,679,162-6,945,359 | p22.1 | CN Gain | 266197 | 71.43 | 11.11 | 60.32 | 0.00 | 0.00 | ZNF12, PMS2CL, RSPH10B, RSPH10B2, CCZ1B |
| chr7:62,112,436-62,151,187 | q11.21 | CN Gain | 38751 | 71.43 | 11.11 | 60.32 | 0.00 | 0.00 |  |
| chr7:66,718,559-66,753,740 | q11.22 | CN Gain | 35181 | 71.43 | 11.11 | 60.32 | 0.00 | 0.00 |  |
| chr7:20,697,544-20,697,648 | p15.3 | CN Gain | 104 | 73.81 | 13.33 | 60.48 | 0.00 | 0.00 | ABCB5 |
| chr7:3,009,953-3,011,400 | p22.2 | CN Gain | 1447 | 73.81 | 13.33 | 60.48 | 0.00 | 0.00 | CARD11 |
| chr7:33,824,476-33,922,058 | p14.3 | CN Gain | 97582 | 73.81 | 13.33 | 60.48 | 0.00 | 0.00 | BMPER |
| chr7:36,469,984-36,544,349 | p14.2 | CN Gain | 74365 | 73.81 | 13.33 | 60.48 | 0.00 | 0.00 | AOAH |
| chr7:37,920,941-38,185,997 | p14.1 | CN Gain | 265056 | 73.81 | 13.33 | 60.48 | 0.00 | 0.00 | SFRP4, EPDR1, STARD3NL |
| chr7:38,441,839-38,451,422 | p14.1 | CN Gain | 9583 | 73.81 | 13.33 | 60.48 | 0.00 | 0.00 | AMPH |
| chr7:45,587,740-45,605,010 | p13 | CN Gain | 17270 | 73.81 | 13.33 | 60.48 | 0.00 | 0.00 | ADCY1 |
| chr7:6,419,874-6,576,394 | p22.1 | CN Gain | 156520 | 73.81 | 13.33 | 60.48 | 0.00 | 0.00 | DAGLB, KDELR2, GRID2IP |
| chr7:69,285,823-69,661,863 | q11.22 | CN Gain | 376040 | 73.81 | 13.33 | 60.48 | 0.00 | 0.00 | AUTS2 |
| chr7:7,034,254-7,071,183 | p22.1 | CN Gain | 36929 | 73.81 | 13.33 | 60.48 | 0.00 | 0.00 |  |
| chr7:70,130,243-70,348,268 | q11.22 | CN Gain | 218025 | 73.81 | 13.33 | 60.48 | 0.00 | 0.00 | WBSCR17 |
| chr7:71,611,578-71,789,465 | q11.22 | CN Gain | 177887 | 73.81 | 13.33 | 60.48 | 0.00 | 0.00 | TYW1B |
| chr7:150,121,663-150,127,585 | q36.1 | CN Gain | 5922 | 83.33 | 22.22 | 61.11 | 0.00 | 0.00 | TMEM176B |
| chr7:16,620,664-16,632,299 | p21.1 | CN Gain | 11635 | 76.19 | 15.56 | 60.63 | 0.00 | 0.00 | ANKMY2 |
| chr7:27,513,350-27,565,173 | p15.2 | CN Gain | 51823 | 83.33 | 22.22 | 61.11 | 0.00 | 0.00 | HIBADH |
| chr7:28,467,257-28,583,111 | p15.1 | CN Gain | 115854 | 76.19 | 15.56 | 60.63 | 0.00 | 0.00 | CREB5 |
| chr7:35,621,552-35,631,918 | p14.2 | CN Gain | 10366 | 76.19 | 15.56 | 60.63 | 0.00 | 0.00 |  |
| chr7:38,235,472-38,261,592 | p14.1 | CN Gain | 26120 | 76.19 | 15.56 | 60.63 | 0.00 | 0.00 | STARD3NL |
| chr7:38,397,945-38,441,839 | p14.1 | CN Gain | 43894 | 76.19 | 15.56 | 60.63 | 0.00 | 0.00 | AMPH |
| chr7:45,021,796-45,204,789 | p13 | CN Gain | 182993 | 76.19 | 15.56 | 60.63 | 0.00 | 0.00 | CCM2, NACAD, SNORA5A, SNORA5C, TBRG4, SNORA5B, RAMP3 |
| chr7:50,666,845-50,669,349 | p12.2 | CN Gain | 2504 | 76.19 | 15.56 | 60.63 | 0.00 | 0.00 | GRB10 |
| chr7:50,671,632-50,672,637 | p12.2 | CN Gain | 1005 | 76.19 | 15.56 | 60.63 | 0.00 | 0.00 | GRB10 |
| chr7:55,229,478-55,235,987 | p11.2 | CN Gain | 6509 | 83.33 | 22.22 | 61.11 | 0.00 | 0.00 | EGFR |
| chr7:61,068,460-62,035,216 | q11.1 - q11.21 | CN Gain | 966756 | 76.19 | 15.56 | 60.63 | 0.00 | 0.00 |  |
| chr7:2,902,609-2,922,117 | p22.2 | CN Gain | 19508 | 80.95 | 20.00 | 60.95 | 0.00 | 0.00 | CARD11 |
| chr7:2,943,775-2,959,556 | p22.2 | CN Gain | 15781 | 78.57 | 17.78 | 60.79 | 0.00 | 0.00 | CARD11 |
| chr7:20,589,709-20,637,961 | p15.3 | CN Gain | 48252 | 78.57 | 17.78 | 60.79 | 0.00 | 0.00 | ABCB5 |
| chr7:26,182,792-26,189,037 | p15.2 | CN Gain | 6245 | 78.57 | 17.78 | 60.79 | 0.00 | 0.00 | NFE2L3 |
| chr7:26,543,252-26,569,859 | p15.2 | CN Gain | 26607 | 78.57 | 17.78 | 60.79 | 0.00 | 0.00 | KIAA0087 |
| chr7:27,206,560-27,208,089 | p15.2 | CN Gain | 1529 | 78.57 | 17.78 | 60.79 | 0.00 | 0.00 | HOTTIP |
| chr7:28,611,110-28,651,387 | p15.1 | CN Gain | 40277 | 80.95 | 20.00 | 60.95 | 0.00 | 0.00 | CREB5 |
| chr7:30,151,713-30,238,804 | p15.1 | CN Gain | 87091 | 78.57 | 17.78 | 60.79 | 0.00 | 0.00 | C7orf41 |
| chr7:35,642,220-35,644,998 | p14.2 | CN Gain | 2778 | 78.57 | 17.78 | 60.79 | 0.00 | 0.00 | HERPUD2 |
| chr7:35,651,475-35,714,574 | p14.2 | CN Gain | 63099 | 80.95 | 20.00 | 60.95 | 0.00 | 0.00 | HERPUD2 |
| chr7:36,080,777-36,305,571 | p14.2 | CN Gain | 224794 | 78.57 | 17.78 | 60.79 | 0.00 | 0.00 | EEPD1 |
| chr7:38,261,592-38,289,333 | p14.1 | CN Gain | 27741 | 78.57 | 17.78 | 60.79 | 0.00 | 0.00 | TARP |
| chr7:38,309,765-38,325,095 | p14.1 | CN Gain | 15330 | 80.95 | 20.00 | 60.95 | 0.00 | 0.00 |  |
| chr7:38,349,942-38,356,352 | p14.1 | CN Gain | 6410 | 78.57 | 17.78 | 60.79 | 0.00 | 0.00 | LOC100506776 |
| chr7:4,832,740-4,866,805 | p22.1 | CN Gain | 34065 | 78.57 | 17.78 | 60.79 | 0.00 | 0.00 | RADIL, PAPOLB |
| chr7:44,307,448-44,685,827 | p13 | CN Gain | 378379 | 80.95 | 20.00 | 60.95 | 0.00 | 0.00 | CAMK2B, NUDCD3, NPC1L1, DDX56, TMED4, OGDH |
| chr7:45,006,088-45,021,796 | p13 | CN Gain | 15708 | 78.57 | 17.78 | 60.79 | 0.00 | 0.00 | CCM2 |
| chr7:54,745,335-54,767,240 | p11.2 | CN Gain | 21905 | 80.95 | 20.00 | 60.95 | 0.00 | 0.00 |  |
| chr7:55,248,582-55,259,517 | p11.2 | CN Gain | 10935 | 80.95 | 20.00 | 60.95 | 0.00 | 0.00 |  |
| chr7:71,961,223-71,990,023 | q11.23 | CN Gain | 28800 | 78.57 | 17.78 | 60.79 | 0.00 | 0.00 | SPDYE7P, POM121 |
| chr7:53,049,732-53,071,341 | p12.1 | CN Gain | 21609 | 64.29 | 6.67 | 57.62 | 0.00 | 0.00 | POM121L12 |
| chr7:52,797,666-52,963,968 | p12.1 | CN Gain | 166302 | 59.52 | 4.44 | 55.08 | 0.00 | 0.00 |  |
| chr7:3,552,124-3,644,011 | p22.2 | CN Gain | 91887 | 69.05 | 11.11 | 57.94 | 0.00 | 0.00 | SDK1 |
| chr7:38,513,254-38,573,586 | p14.1 | CN Gain | 60332 | 69.05 | 11.11 | 57.94 | 0.00 | 0.00 | AMPH |
| chr7:38,877,237-38,910,686 | p14.1 | CN Gain | 33449 | 69.05 | 11.11 | 57.94 | 0.00 | 0.00 | VPS41 |
| chr7:39,280,377-39,526,197 | p14.1 | CN Gain | 245820 | 69.05 | 11.11 | 57.94 | 0.00 | 0.00 | POU6F2, POU6F2-AS1 |
| chr7:40,361,712-40,739,177 | p14.1 | CN Gain | 377465 | 69.05 | 11.11 | 57.94 | 0.00 | 0.00 | C7orf10 |
| chr7:40,765,382-40,853,139 | p14.1 | CN Gain | 87757 | 69.05 | 11.11 | 57.94 | 0.00 | 0.00 | C7orf10 |
| chr7:56,548,662-56,647,884 | p11.2 | CN Gain | 99222 | 69.05 | 11.11 | 57.94 | 0.00 | 0.00 |  |
| chr7:57,307,306-57,358,655 | p11.2 | CN Gain | 51349 | 69.05 | 11.11 | 57.94 | 0.00 | 0.00 |  |
| chr7:66,753,740-66,792,412 | q11.22 | CN Gain | 38672 | 69.05 | 11.11 | 57.94 | 0.00 | 0.00 |  |
| chr7:23,823,575-23,901,446 | p15.3 | CN Gain | 77871 | 66.67 | 8.89 | 57.78 | 0.00 | 0.00 | STK31 |
| chr7:29,369,735-29,431,434 | p15.1 | CN Gain | 61699 | 66.67 | 8.89 | 57.78 | 0.00 | 0.00 | CHN2 |
| chr7:46,254,227-46,641,581 | p13 - p12.3 | CN Gain | 387354 | 66.67 | 8.89 | 57.78 | 0.00 | 0.00 |  |
| chr7:157,056,381-157,110,366 | q36.3 | CN Gain | 53985 | 85.71 | 26.67 | 59.05 | 0.00 | 0.00 | MIR153-2, PTPRN2 |
| chr7:27,695,546-27,853,968 | p15.2 | CN Gain | 158422 | 85.71 | 26.67 | 59.05 | 0.00 | 0.00 | TAX1BP1, JAZF1 |
| chr7:16,352,183-16,471,709 | p21.1 | CN Gain | 119526 | 71.43 | 13.33 | 58.10 | 0.00 | 0.00 | ISPD, SOSTDC1 |
| chr7:45,572,908-45,587,740 | p13 | CN Gain | 14832 | 71.43 | 13.33 | 58.10 | 0.00 | 0.00 | ADCY1 |
| chr7:45,605,010-45,614,352 | p13 | CN Gain | 9342 | 71.43 | 13.33 | 58.10 | 0.00 | 0.00 | ADCY1 |
| chr7:56,717,793-56,940,786 | p11.2 | CN Gain | 222993 | 71.43 | 13.33 | 58.10 | 0.00 | 0.00 | LOC100130849 |
| chr7:6,209,861-6,419,874 | p22.1 | CN Gain | 210013 | 71.43 | 13.33 | 58.10 | 0.00 | 0.00 | CYTH3, FAM220A, RAC1, DAGLB |
| chr7:6,945,359-7,034,254 | p22.1 | CN Gain | 88895 | 71.43 | 13.33 | 58.10 | 0.00 | 0.00 |  |
| chr7:62,084,576-62,112,436 | q11.21 | CN Gain | 27860 | 71.43 | 13.33 | 58.10 | 0.00 | 0.00 |  |
| chr7:7,071,183-7,224,058 | p22.1 - p21.3 | CN Gain | 152875 | 71.43 | 13.33 | 58.10 | 0.00 | 0.00 | LOC100131257, C1GALT1 |
| chr7:70,348,268-70,357,280 | q11.22 | CN Gain | 9012 | 71.43 | 13.33 | 58.10 | 0.00 | 0.00 | WBSCR17 |
| chr7:71,555,382-71,611,578 | q11.22 | CN Gain | 56196 | 71.43 | 13.33 | 58.10 | 0.00 | 0.00 |  |
| chr7:150,127,585-150,131,202 | q36.1 | CN Gain | 3617 | 83.33 | 24.44 | 58.89 | 0.00 | 0.00 | TMEM176B, TMEM176A |
| chr7:156,886,590-156,892,513 | q36.3 | CN Gain | 5923 | 83.33 | 24.44 | 58.89 | 0.00 | 0.00 | DNAJB6 |
| chr7:16,515,831-16,620,664 | p21.1 | CN Gain | 104833 | 73.81 | 15.56 | 58.25 | 0.00 | 0.00 | LRRC72, ANKMY2 |
| chr7:16,762,389-16,789,521 | p21.1 | CN Gain | 27132 | 73.81 | 15.56 | 58.25 | 0.00 | 0.00 | TSPAN13 |
| chr7:2,980,171-3,009,953 | p22.2 | CN Gain | 29782 | 73.81 | 15.56 | 58.25 | 0.00 | 0.00 | CARD11 |
| chr7:20,649,451-20,697,544 | p15.3 | CN Gain | 48093 | 73.81 | 15.56 | 58.25 | 0.00 | 0.00 | ABCB5 |
| chr7:27,565,173-27,619,643 | p15.2 | CN Gain | 54470 | 83.33 | 24.44 | 58.89 | 0.00 | 0.00 | HIBADH |
| chr7:28,100,428-28,235,970 | p15.1 | CN Gain | 135542 | 83.33 | 24.44 | 58.89 | 0.00 | 0.00 | JAZF1, JAZF1-AS1 |
| chr7:29,008,608-29,065,632 | p15.1 | CN Gain | 57024 | 83.33 | 24.44 | 58.89 | 0.00 | 0.00 | LOC100506497, CPVL |
| chr7:36,435,733-36,469,984 | p14.2 | CN Gain | 34251 | 73.81 | 15.56 | 58.25 | 0.00 | 0.00 | ANLN |
| chr7:38,185,997-38,235,472 | p14.1 | CN Gain | 49475 | 73.81 | 15.56 | 58.25 | 0.00 | 0.00 | STARD3NL |
| chr7:45,204,789-45,389,091 | p13 | CN Gain | 184302 | 73.81 | 15.56 | 58.25 | 0.00 | 0.00 |  |
| chr7:62,035,216-62,057,841 | q11.21 | CN Gain | 22625 | 73.81 | 15.56 | 58.25 | 0.00 | 0.00 |  |
| chr7:69,192,861-69,285,823 | q11.22 | CN Gain | 92962 | 73.81 | 15.56 | 58.25 | 0.00 | 0.00 | AUTS2 |
| chr7:76,120,252-76,723,790 | q11.23 | CN Gain | 603538 | 83.33 | 24.44 | 58.89 | 0.00 | 0.00 | DTX2P1-UPK3BP1-PMS2P11, LOC100132832, FGL2, CCDC146 |
| chr7:15,711,708-15,716,427 | p21.1 | CN Gain | 4719 | 76.19 | 17.78 | 58.41 | 0.00 | 0.00 |  |
| chr7:150,117,808-150,121,663 | q36.1 | CN Gain | 3855 | 80.95 | 22.22 | 58.73 | 0.00 | 0.00 | TMEM176B |
| chr7:16,632,299-16,752,851 | p21.1 | CN Gain | 120552 | 76.19 | 17.78 | 58.41 | 0.00 | 0.00 | ANKMY2, BZW2 |
| chr7:20,637,961-20,649,451 | p15.3 | CN Gain | 11490 | 76.19 | 17.78 | 58.41 | 0.00 | 0.00 | ABCB5 |
| chr7:28,235,970-28,238,603 | p15.1 | CN Gain | 2633 | 80.95 | 22.22 | 58.73 | 0.00 | 0.00 | JAZF1-AS1 |
| chr7:28,583,111-28,591,851 | p15.1 | CN Gain | 8740 | 76.19 | 17.78 | 58.41 | 0.00 | 0.00 | CREB5 |
| chr7:28,651,387-28,671,364 | p15.1 | CN Gain | 19977 | 80.95 | 22.22 | 58.73 | 0.00 | 0.00 | CREB5 |
| chr7:28,937,474-28,997,489 | p15.1 | CN Gain | 60015 | 80.95 | 22.22 | 58.73 | 0.00 | 0.00 | TRIL, LOC100506497 |
| chr7:35,631,918-35,642,220 | p14.2 | CN Gain | 10302 | 76.19 | 17.78 | 58.41 | 0.00 | 0.00 | HERPUD2 |
| chr7:36,032,394-36,080,777 | p14.2 | CN Gain | 48383 | 76.19 | 17.78 | 58.41 | 0.00 | 0.00 |  |
| chr7:36,305,571-36,370,603 | p14.2 | CN Gain | 65032 | 76.19 | 17.78 | 58.41 | 0.00 | 0.00 | EEPD1, KIAA0895 |
| chr7:38,356,352-38,397,945 | p14.1 | CN Gain | 41593 | 76.19 | 17.78 | 58.41 | 0.00 | 0.00 | LOC100506776, AMPH |
| chr7:50,669,349-50,671,632 | p12.2 | CN Gain | 2283 | 76.19 | 17.78 | 58.41 | 0.00 | 0.00 | GRB10 |
| chr7:52,972,637-53,049,732 | p12.1 | CN Gain | 77095 | 61.90 | 6.67 | 55.24 | 0.00 | 0.00 |  |
| chr7:55,235,987-55,248,582 | p11.2 | CN Gain | 12595 | 80.95 | 22.22 | 58.73 | 0.00 | 0.00 | EGFR |
| chr7:77,442,001-77,548,662 | q21.11 | CN Gain | 106661 | 80.95 | 22.22 | 58.73 | 0.00 | 0.00 | MAGI2 |
| chr7:2,877,377-2,902,609 | p22.2 | CN Gain | 25232 | 78.57 | 20.00 | 58.57 | 0.00 | 0.00 |  |
| chr7:20,478,016-20,589,709 | p15.3 | CN Gain | 111693 | 78.57 | 20.00 | 58.57 | 0.00 | 0.00 |  |
| chr7:26,536,161-26,543,252 | p15.2 | CN Gain | 7091 | 78.57 | 20.00 | 58.57 | 0.00 | 0.00 | KIAA0087 |
| chr7:35,644,998-35,651,475 | p14.2 | CN Gain | 6477 | 78.57 | 20.00 | 58.57 | 0.00 | 0.00 | HERPUD2 |
| chr7:4,866,805-5,091,823 | p22.1 | CN Gain | 225018 | 78.57 | 20.00 | 58.57 | 0.00 | 0.00 | RADIL, PAPOLB, MMD2, RNF216P1, RBAK, RBAK-LOC389458, LOC389458 |
| chr7:44,685,827-45,006,088 | p13 | CN Gain | 320261 | 78.57 | 20.00 | 58.57 | 0.00 | 0.00 | OGDH, ZMIZ2, PPIA, H2AFV, PURB, MIR4657, MYO1G, SNHG15, SNORA9, CCM2 |
| chr7:71,990,023-72,409,184 | q11.23 | CN Gain | 419161 | 78.57 | 20.00 | 58.57 | 0.00 | 0.00 | POM121, NSUN5P2, TRIM74, STAG3L3, SPDYE8P, PMS2L2, LOC100093631, GTF2IP1, NCF1B, GTF2IRD2P1, NSUN5, TRIM50, FKBP6 |
| chr7:24,268,864-24,278,536 | p15.3 | CN Gain | 9672 | 66.67 | 11.11 | 55.56 | 0.00 | 0.00 |  |
| chr7:29,431,434-29,453,408 | p15.1 | CN Gain | 21974 | 66.67 | 11.11 | 55.56 | 0.00 | 0.00 | CHN2 |
| chr7:38,573,586-38,747,997 | p14.1 | CN Gain | 174411 | 66.67 | 11.11 | 55.56 | 0.00 | 0.00 | AMPH, FAM183B, VPS41 |
| chr7:38,809,423-38,877,237 | p14.1 | CN Gain | 67814 | 66.67 | 11.11 | 55.56 | 0.00 | 0.00 | VPS41 |
| chr7:40,194,262-40,361,712 | p14.1 | CN Gain | 167450 | 66.67 | 11.11 | 55.56 | 0.00 | 0.00 | C7orf10 |
| chr7:40,739,177-40,765,382 | p14.1 | CN Gain | 26205 | 66.67 | 11.11 | 55.56 | 0.00 | 0.00 | C7orf10 |
| chr7:45,979,786-45,995,372 | p13 | CN Gain | 15586 | 66.67 | 11.11 | 55.56 | 0.00 | 0.00 |  |
| chr7:57,358,655-57,401,430 | p11.2 - p11.1 | CN Gain | 42775 | 66.67 | 11.11 | 55.56 | 0.00 | 0.00 |  |
| chr7:66,792,412-66,801,015 | q11.22 | CN Gain | 8603 | 66.67 | 11.11 | 55.56 | 0.00 | 0.00 |  |
| chr7:67,539,333-67,662,105 | q11.22 | CN Gain | 122772 | 66.67 | 11.11 | 55.56 | 0.00 | 0.00 |  |
| chr7:24,000,328-24,268,864 | p15.3 | CN Gain | 268536 | 64.29 | 8.89 | 55.40 | 0.00 | 0.00 |  |
| chr7:46,005,076-46,254,227 | p13 | CN Gain | 249151 | 64.29 | 8.89 | 55.40 | 0.00 | 0.00 |  |
| chr7:131,508,505-131,644,641 | q32.3 | CN Gain | 136136 | 85.71 | 28.89 | 56.83 | 0.00 | 0.00 | PLXNA4 |
| chr7:156,996,134-157,056,381 | q36.3 | CN Gain | 60247 | 85.71 | 28.89 | 56.83 | 0.00 | 0.00 | PTPRN2 |
| chr7:27,853,968-27,937,860 | p15.2 | CN Gain | 83892 | 85.71 | 28.89 | 56.83 | 0.00 | 0.00 | JAZF1 |
| chr7:39,526,197-39,569,493 | p14.1 | CN Gain | 43296 | 69.05 | 13.33 | 55.71 | 0.00 | 0.00 |  |
| chr7:39,869,503-40,099,921 | p14.1 | CN Gain | 230418 | 69.05 | 13.33 | 55.71 | 0.00 | 0.00 | CDK13 |
| chr7:57,401,430-58,014,651 | p11.1 | CN Gain | 613221 | 69.05 | 13.33 | 55.71 | 0.00 | 0.00 | MIR3147, ZNF716 |
| chr7:68,154,771-68,197,404 | q11.22 | CN Gain | 42633 | 69.05 | 13.33 | 55.71 | 0.00 | 0.00 |  |
| chr7:68,210,359-68,628,327 | q11.22 | CN Gain | 417968 | 69.05 | 13.33 | 55.71 | 0.00 | 0.00 |  |
| chr7:70,357,280-70,367,743 | q11.22 | CN Gain | 10463 | 69.05 | 13.33 | 55.71 | 0.00 | 0.00 | WBSCR17 |
| chr7:71,509,295-71,555,382 | q11.22 | CN Gain | 46087 | 69.05 | 13.33 | 55.71 | 0.00 | 0.00 | CALN1 |
| chr7:156,892,513-156,926,234 | q36.3 | CN Gain | 33721 | 83.33 | 26.67 | 56.67 | 0.00 | 0.00 | DNAJB6 |
| chr7:157,110,366-157,740,432 | q36.3 | CN Gain | 630066 | 83.33 | 26.67 | 56.67 | 0.00 | 0.00 | LOC100506585, PTPRN2 |
| chr7:27,619,643-27,695,546 | p15.2 | CN Gain | 75903 | 83.33 | 26.67 | 56.67 | 0.00 | 0.00 | HIBADH |
| chr7:16,019,589-16,052,142 | p21.1 | CN Gain | 32553 | 71.43 | 15.56 | 55.87 | 0.00 | 0.00 |  |
| chr7:16,471,709-16,515,831 | p21.1 | CN Gain | 44122 | 71.43 | 15.56 | 55.87 | 0.00 | 0.00 | SOSTDC1 |
| chr7:16,789,521-16,824,777 | p21.1 | CN Gain | 35256 | 71.43 | 15.56 | 55.87 | 0.00 | 0.00 | TSPAN13, AGR2 |
| chr7:39,569,493-39,851,800 | p14.1 | CN Gain | 282307 | 71.43 | 15.56 | 55.87 | 0.00 | 0.00 | YAE1D1, LOC646999, RALA, LINC00265 |
| chr7:45,389,091-45,572,908 | p13 | CN Gain | 183817 | 71.43 | 15.56 | 55.87 | 0.00 | 0.00 |  |
| chr7:45,614,352-45,722,719 | p13 | CN Gain | 108367 | 71.43 | 15.56 | 55.87 | 0.00 | 0.00 | ADCY1 |
| chr7:6,019,436-6,209,861 | p22.1 | CN Gain | 190425 | 71.43 | 15.56 | 55.87 | 0.00 | 0.00 | AIMP2, ANKRD61, EIF2AK1, USP42, CYTH3 |
| chr7:62,057,841-62,084,576 | q11.21 | CN Gain | 26735 | 71.43 | 15.56 | 55.87 | 0.00 | 0.00 |  |
| chr7:68,648,664-69,192,861 | q11.22 | CN Gain | 544197 | 71.43 | 15.56 | 55.87 | 0.00 | 0.00 | AUTS2 |
| chr7:105,434,634-105,469,449 | q22.2 | CN Gain | 34815 | 80.95 | 24.44 | 56.51 | 0.00 | 0.00 | CDHR3 |
| chr7:150,131,202-150,230,186 | q36.1 | CN Gain | 98984 | 80.95 | 24.44 | 56.51 | 0.00 | 0.00 | TMEM176A, ABP1 |
| chr7:156,843,582-156,886,590 | q36.3 | CN Gain | 43008 | 80.95 | 24.44 | 56.51 | 0.00 | 0.00 | DNAJB6 |
| chr7:28,671,364-28,701,983 | p15.1 | CN Gain | 30619 | 80.95 | 24.44 | 56.51 | 0.00 | 0.00 | CREB5 |
| chr7:28,787,664-28,819,131 | p15.1 | CN Gain | 31467 | 80.95 | 24.44 | 56.51 | 0.00 | 0.00 | CREB5 |
| chr7:28,997,489-29,008,608 | p15.1 | CN Gain | 11119 | 80.95 | 24.44 | 56.51 | 0.00 | 0.00 | LOC100506497, CPVL |
| chr7:76,723,790-76,795,745 | q11.23 | CN Gain | 71955 | 80.95 | 24.44 | 56.51 | 0.00 | 0.00 | CCDC146, PION |
| chr7:76,825,560-76,866,870 | q11.23 | CN Gain | 41310 | 80.95 | 24.44 | 56.51 | 0.00 | 0.00 | PION |
| chr7:77,440,786-77,442,001 | q21.11 | CN Gain | 1215 | 80.95 | 24.44 | 56.51 | 0.00 | 0.00 |  |
| chr7:77,548,662-77,551,704 | q21.11 | CN Gain | 3042 | 80.95 | 24.44 | 56.51 | 0.00 | 0.00 | MAGI2 |
| chr7:15,716,427-15,725,078 | p21.1 | CN Gain | 8651 | 73.81 | 17.78 | 56.03 | 0.00 | 0.00 |  |
| chr7:16,752,851-16,762,389 | p21.1 | CN Gain | 9538 | 73.81 | 17.78 | 56.03 | 0.00 | 0.00 | TSPAN13 |
| chr7:17,358,962-17,466,280 | p21.1 | CN Gain | 107318 | 73.81 | 17.78 | 56.03 | 0.00 | 0.00 |  |
| chr7:2,959,556-2,980,171 | p22.2 | CN Gain | 20615 | 73.81 | 17.78 | 56.03 | 0.00 | 0.00 | CARD11 |
| chr7:36,370,603-36,435,733 | p14.2 | CN Gain | 65130 | 73.81 | 17.78 | 56.03 | 0.00 | 0.00 | KIAA0895, ANLN |
| chr7:15,690,750-15,711,708 | p21.1 | CN Gain | 20958 | 76.19 | 20.00 | 56.19 | 0.00 | 0.00 | MEOX2 |
| chr7:150,082,700-150,117,808 | q36.1 | CN Gain | 35108 | 78.57 | 22.22 | 56.35 | 0.00 | 0.00 |  |
| chr7:19,803,612-20,013,365 | p15.3 | CN Gain | 209753 | 76.19 | 20.00 | 56.19 | 0.00 | 0.00 |  |
| chr7:2,812,958-2,877,377 | p22.2 | CN Gain | 64419 | 76.19 | 20.00 | 56.19 | 0.00 | 0.00 | GNA12 |
| chr7:20,027,858-20,053,113 | p15.3 | CN Gain | 25255 | 78.57 | 22.22 | 56.35 | 0.00 | 0.00 |  |
| chr7:26,189,037-26,197,014 | p15.2 | CN Gain | 7977 | 78.57 | 22.22 | 56.35 | 0.00 | 0.00 | NFE2L3, HNRNPA2B1 |
| chr7:26,461,548-26,536,161 | p15.2 | CN Gain | 74613 | 76.19 | 20.00 | 56.19 | 0.00 | 0.00 | LOC441204 |
| chr7:28,591,851-28,611,110 | p15.1 | CN Gain | 19259 | 76.19 | 20.00 | 56.19 | 0.00 | 0.00 | CREB5 |
| chr7:28,884,481-28,937,474 | p15.1 | CN Gain | 52993 | 78.57 | 22.22 | 56.35 | 0.00 | 0.00 |  |
| chr7:35,714,574-36,032,394 | p14.2 | CN Gain | 317820 | 76.19 | 20.00 | 56.19 | 0.00 | 0.00 | 7-Sep |
| chr7:5,091,823-5,230,208 | p22.1 | CN Gain | 138385 | 78.57 | 22.22 | 56.35 | 0.00 | 0.00 | ZNF890P, WIPI2 |
| chr7:72,409,184-72,786,211 | q11.23 | CN Gain | 377027 | 78.57 | 22.22 | 56.35 | 0.00 | 0.00 | FKBP6, FZD9, BAZ1B, BCL7B, TBL2, MLXIPL, VPS37D, DNAJC30, WBSCR22, STX1A, MIR4284 |
| chr7:75,190,943-75,238,241 | q11.23 | CN Gain | 47298 | 78.57 | 22.22 | 56.35 | 0.00 | 0.00 | HIP1, CCL26 |
| chr7:55,175,277-55,180,181 | p11.2 | CN Gain | 4904 | 88.10 | 33.33 | 54.76 | 0.00 | 0.00 | EGFR |
| chr7:38,747,997-38,809,423 | p14.1 | CN Gain | 61426 | 64.29 | 11.11 | 53.17 | 0.00 | 0.00 | VPS41 |
| chr7:45,995,372-46,005,076 | p13 | CN Gain | 9704 | 64.29 | 11.11 | 53.17 | 0.00 | 0.00 |  |
| chr7:66,801,015-67,539,333 | q11.22 | CN Gain | 738318 | 64.29 | 11.11 | 53.17 | 0.00 | 0.00 |  |
| chr7:67,662,105-67,688,202 | q11.22 | CN Gain | 26097 | 64.29 | 11.11 | 53.17 | 0.00 | 0.00 |  |
| chr7:67,862,541-67,993,274 | q11.22 | CN Gain | 130733 | 64.29 | 11.11 | 53.17 | 0.00 | 0.00 |  |
| chr7:23,901,446-24,000,328 | p15.3 | CN Gain | 98882 | 61.90 | 8.89 | 53.02 | 0.00 | 0.00 |  |
| chr7:1,217,412-2,178,655 | p22.3 - p22.2 | CN Gain | 961243 | 85.71 | 31.11 | 54.60 | 0.00 | 0.00 | UNCX, MICALL2, INTS1, MAFK, TMEM184A, PSMG3, PSMG3-AS1, TFAMP1, ELFN1, MIR4655, MAD1L1 |
| chr7:139,331,338-139,333,828 | q34 | CN Gain | 2490 | 85.71 | 31.11 | 54.60 | 0.00 | 0.00 | TBXAS1 |
| chr7:150,934,339-151,060,251 | q36.1 | CN Gain | 125912 | 85.71 | 31.11 | 54.60 | 0.00 | 0.00 | PRKAG2 |
| chr7:27,937,860-27,963,526 | p15.2 | CN Gain | 25666 | 85.71 | 31.11 | 54.60 | 0.00 | 0.00 | JAZF1 |
| chr7:54,830,301-54,834,444 | p11.2 | CN Gain | 4143 | 85.71 | 31.11 | 54.60 | 0.00 | 0.00 |  |
| chr7:40,099,921-40,194,262 | p14.1 | CN Gain | 94341 | 66.67 | 13.33 | 53.33 | 0.00 | 0.00 | CDK13, MPLKIP, C7orf10 |
| chr7:45,898,604-45,937,609 | p13 | CN Gain | 39005 | 66.67 | 13.33 | 53.33 | 0.00 | 0.00 | IGFBP1, IGFBP3 |
| chr7:45,966,857-45,979,786 | p13 | CN Gain | 12929 | 66.67 | 13.33 | 53.33 | 0.00 | 0.00 |  |
| chr7:68,076,181-68,154,771 | q11.22 | CN Gain | 78590 | 66.67 | 13.33 | 53.33 | 0.00 | 0.00 |  |
| chr7:68,197,404-68,210,359 | q11.22 | CN Gain | 12955 | 66.67 | 13.33 | 53.33 | 0.00 | 0.00 |  |
| chr7:70,367,743-70,544,019 | q11.22 | CN Gain | 176276 | 66.67 | 13.33 | 53.33 | 0.00 | 0.00 | MIR3914-1, MIR3914-2, WBSCR17 |
| chr7:130,906,056-130,947,682 | q32.3 | CN Gain | 41626 | 83.33 | 28.89 | 54.44 | 0.00 | 0.00 |  |
| chr7:131,495,253-131,508,505 | q32.3 | CN Gain | 13252 | 83.33 | 28.89 | 54.44 | 0.00 | 0.00 | PLXNA4 |
| chr7:131,644,641-132,078,164 | q32.3 | CN Gain | 433523 | 83.33 | 28.89 | 54.44 | 0.00 | 0.00 | PLXNA4, FLJ40288 |
| chr7:156,926,234-156,996,134 | q36.3 | CN Gain | 69900 | 83.33 | 28.89 | 54.44 | 0.00 | 0.00 |  |
| chr7:157,775,629-157,918,638 | q36.3 | CN Gain | 143009 | 83.33 | 28.89 | 54.44 | 0.00 | 0.00 | PTPRN2 |
| chr7:2,178,655-2,201,310 | p22.2 | CN Gain | 22655 | 83.33 | 28.89 | 54.44 | 0.00 | 0.00 | MAD1L1 |
| chr7:28,086,959-28,100,428 | p15.1 | CN Gain | 13469 | 83.33 | 28.89 | 54.44 | 0.00 | 0.00 | JAZF1 |
| chr7:75,938,049-76,120,252 | q11.23 | CN Gain | 182203 | 83.33 | 28.89 | 54.44 | 0.00 | 0.00 | FDPSL2A, DTX2, UPK3B, LOC100133091, POMZP3 |
| chr7:16,052,142-16,352,183 | p21.1 | CN Gain | 300041 | 69.05 | 15.56 | 53.49 | 0.00 | 0.00 | LOC100506025, ISPD |
| chr7:17,762,319-17,768,429 | p21.1 | CN Gain | 6110 | 69.05 | 15.56 | 53.49 | 0.00 | 0.00 |  |
| chr7:39,851,800-39,869,503 | p14.1 | CN Gain | 17703 | 69.05 | 15.56 | 53.49 | 0.00 | 0.00 |  |
| chr7:45,722,719-45,771,109 | p13 | CN Gain | 48390 | 69.05 | 15.56 | 53.49 | 0.00 | 0.00 | ADCY1, SEPT7P2 |
| chr7:6,012,880-6,019,436 | p22.1 | CN Gain | 6556 | 69.05 | 15.56 | 53.49 | 0.00 | 0.00 | PMS2, AIMP2 |
| chr7:68,628,327-68,648,664 | q11.22 | CN Gain | 20337 | 69.05 | 15.56 | 53.49 | 0.00 | 0.00 |  |
| chr7:150,230,186-150,289,733 | q36.1 | CN Gain | 59547 | 80.95 | 26.67 | 54.29 | 0.00 | 0.00 | KCNH2 |
| chr7:154,276,703-154,368,146 | q36.2 | CN Gain | 91443 | 80.95 | 26.67 | 54.29 | 0.00 | 0.00 | DPP6, LOC100132707, PAXIP1 |
| chr7:157,740,432-157,754,963 | q36.3 | CN Gain | 14531 | 80.95 | 26.67 | 54.29 | 0.00 | 0.00 | PTPRN2 |
| chr7:28,701,983-28,787,664 | p15.1 | CN Gain | 85681 | 80.95 | 26.67 | 54.29 | 0.00 | 0.00 | CREB5 |
| chr7:73,046,160-73,074,666 | q11.23 | CN Gain | 28506 | 80.95 | 26.67 | 54.29 | 0.00 | 0.00 |  |
| chr7:74,033,117-75,036,209 | q11.23 | CN Gain | 1003092 | 80.95 | 26.67 | 54.29 | 0.00 | 0.00 | GATSL1, WBSCR16, GTF2IRD2B, NCF1C, GTF2IP1, LOC100093631, GATSL2, SPDYE8P, SPDYE8P, PMS2L2, STAG3L1, LOC541473, TRIM73, NSUN5P1, POM121C, SPDYE5, PMS2P3, HIP1 |
| chr7:76,795,745-76,825,560 | q11.23 | CN Gain | 29815 | 80.95 | 26.67 | 54.29 | 0.00 | 0.00 | PION |
| chr7:76,866,870-76,882,978 | q11.23 | CN Gain | 16108 | 80.95 | 26.67 | 54.29 | 0.00 | 0.00 | PION |
| chr7:77,433,082-77,440,786 | q21.11 | CN Gain | 7704 | 80.95 | 26.67 | 54.29 | 0.00 | 0.00 |  |
| chr7:10,433,367-10,707,069 | p21.3 | CN Gain | 273702 | 71.43 | 17.78 | 53.65 | 0.00 | 0.00 |  |
| chr7:15,725,078-15,734,169 | p21.1 | CN Gain | 9091 | 71.43 | 17.78 | 53.65 | 0.00 | 0.00 |  |
| chr7:15,999,370-16,019,589 | p21.1 | CN Gain | 20219 | 71.43 | 17.78 | 53.65 | 0.00 | 0.00 |  |
| chr7:16,824,777-17,019,608 | p21.1 | CN Gain | 194831 | 71.43 | 17.78 | 53.65 | 0.00 | 0.00 | AGR3 |
| chr7:7,254,395-7,387,070 | p21.3 | CN Gain | 132675 | 71.43 | 17.78 | 53.65 | 0.00 | 0.00 | C1GALT1, COL28A1 |
| chr7:105,469,449-105,554,518 | q22.2 | CN Gain | 85069 | 78.57 | 24.44 | 54.13 | 0.00 | 0.00 | SYPL1 |
| chr7:155,357,538-155,461,918 | q36.3 | CN Gain | 104380 | 78.57 | 24.44 | 54.13 | 0.00 | 0.00 |  |
| chr7:156,768,592-156,843,582 | q36.3 | CN Gain | 74990 | 78.57 | 24.44 | 54.13 | 0.00 | 0.00 | DNAJB6 |
| chr7:19,319,384-19,378,758 | p21.1 | CN Gain | 59374 | 78.57 | 24.44 | 54.13 | 0.00 | 0.00 |  |
| chr7:20,053,113-20,154,489 | p15.3 | CN Gain | 101376 | 78.57 | 24.44 | 54.13 | 0.00 | 0.00 | MACC1-AS1, MACC1 |
| chr7:20,407,548-20,478,016 | p15.3 | CN Gain | 70468 | 78.57 | 24.44 | 54.13 | 0.00 | 0.00 | ITGB8 |
| chr7:26,456,874-26,461,548 | p15.2 | CN Gain | 4674 | 78.57 | 24.44 | 54.13 | 0.00 | 0.00 | LOC441204 |
| chr7:28,819,131-28,884,481 | p15.1 | CN Gain | 65350 | 78.57 | 24.44 | 54.13 | 0.00 | 0.00 | CREB5 |
| chr7:72,786,211-72,962,357 | q11.23 | CN Gain | 176146 | 78.57 | 24.44 | 54.13 | 0.00 | 0.00 | ABHD11-AS1, ABHD11, CLDN3, CLDN4, WBSCR27, WBSCR28 |
| chr7:75,036,209-75,190,943 | q11.23 | CN Gain | 154734 | 78.57 | 24.44 | 54.13 | 0.00 | 0.00 | HIP1 |
| chr7:75,238,241-75,280,376 | q11.23 | CN Gain | 42135 | 78.57 | 24.44 | 54.13 | 0.00 | 0.00 | CCL26, CCL24 |
| chr7:77,551,704-77,578,075 | q21.11 | CN Gain | 26371 | 78.57 | 24.44 | 54.13 | 0.00 | 0.00 | MAGI2 |
| chr7:10,353,331-10,395,609 | p21.3 | CN Gain | 42278 | 73.81 | 20.00 | 53.81 | 0.00 | 0.00 |  |
| chr7:106,278,115-106,305,696 | q22.3 | CN Gain | 27581 | 73.81 | 20.00 | 53.81 | 0.00 | 0.00 | PIK3CG |
| chr7:17,019,608-17,358,962 | p21.1 | CN Gain | 339354 | 73.81 | 20.00 | 53.81 | 0.00 | 0.00 | AHR |
| chr7:19,762,917-19,803,612 | p15.3 | CN Gain | 40695 | 73.81 | 20.00 | 53.81 | 0.00 | 0.00 | TMEM196 |
| chr7:105,025,576-105,134,720 | q22.2 | CN Gain | 109144 | 76.19 | 22.22 | 53.97 | 0.00 | 0.00 | ATXN7L1 |
| chr7:105,362,214-105,364,395 | q22.2 | CN Gain | 2181 | 76.19 | 22.22 | 53.97 | 0.00 | 0.00 |  |
| chr7:105,410,120-105,428,092 | q22.2 | CN Gain | 17972 | 76.19 | 22.22 | 53.97 | 0.00 | 0.00 | CDHR3 |
| chr7:105,618,674-105,738,850 | q22.2 | CN Gain | 120176 | 76.19 | 22.22 | 53.97 | 0.00 | 0.00 | NAMPT |
| chr7:15,636,775-15,690,750 | p21.1 | CN Gain | 53975 | 76.19 | 22.22 | 53.97 | 0.00 | 0.00 | MEOX2 |
| chr7:150,063,512-150,082,700 | q36.1 | CN Gain | 19188 | 76.19 | 22.22 | 53.97 | 0.00 | 0.00 | GIMAP1-GIMAP5, GIMAP5 |
| chr7:18,884,619-19,312,666 | p21.1 | CN Gain | 428047 | 76.19 | 22.22 | 53.97 | 0.00 | 0.00 | HDAC9, TWIST1, FERD3L |
| chr7:2,791,657-2,812,958 | p22.2 | CN Gain | 21301 | 76.19 | 22.22 | 53.97 | 0.00 | 0.00 | GNA12 |
| chr7:20,013,365-20,027,858 | p15.3 | CN Gain | 14493 | 76.19 | 22.22 | 53.97 | 0.00 | 0.00 |  |
| chr7:150,786,410-150,794,343 | q36.1 | CN Gain | 7933 | 88.10 | 35.56 | 52.54 | 0.00 | 0.00 | RHEB |
| chr7:55,147,471-55,175,277 | p11.2 | CN Gain | 27806 | 88.10 | 35.56 | 52.54 | 0.00 | 0.00 | EGFR |
| chr7:1,133,376-1,217,412 | p22.3 | CN Gain | 84036 | 85.71 | 33.33 | 52.38 | 0.00 | 0.00 | C7orf50, ZFAND2A |
| chr7:139,333,828-139,341,457 | q34 | CN Gain | 7629 | 85.71 | 33.33 | 52.38 | 0.00 | 0.00 | TBXAS1 |
| chr7:139,365,012-139,365,975 | q34 | CN Gain | 963 | 85.71 | 33.33 | 52.38 | 0.00 | 0.00 | TBXAS1 |
| chr7:150,910,701-150,934,339 | q36.1 | CN Gain | 23638 | 85.71 | 33.33 | 52.38 | 0.00 | 0.00 | PRKAG2 |
| chr7:54,834,444-54,866,255 | p11.2 | CN Gain | 31811 | 85.71 | 33.33 | 52.38 | 0.00 | 0.00 |  |
| chr7:45,937,609-45,966,857 | p13 | CN Gain | 29248 | 64.29 | 13.33 | 50.95 | 0.00 | 0.00 |  |
| chr7:67,993,274-68,076,181 | q11.22 | CN Gain | 82907 | 64.29 | 13.33 | 50.95 | 0.00 | 0.00 |  |
| chr7:70,544,019-70,590,464 | q11.22 | CN Gain | 46445 | 64.29 | 13.33 | 50.95 | 0.00 | 0.00 | WBSCR17 |
| chr7:71,098,009-71,148,476 | q11.22 | CN Gain | 50467 | 64.29 | 13.33 | 50.95 | 0.00 | 0.00 | CALN1 |
| chr7:71,440,758-71,509,295 | q11.22 | CN Gain | 68537 | 64.29 | 13.33 | 50.95 | 0.00 | 0.00 | CALN1 |
| chr10:14,007,788-14,015,410 | p13 | CN Gain | 7622 | 0.00 | 40.00 | -40.00 | 0.00 | 0.00 | FRMD4A |
| chr10:14,081,121-14,116,582 | p13 | CN Gain | 35461 | 0.00 | 40.00 | -40.00 | 0.00 | 0.00 | FRMD4A |
| chr10:27,062,397-27,118,784 | p12.1 | CN Gain | 56387 | 0.00 | 40.00 | -40.00 | 0.00 | 0.00 | PDSS1, ABI1 |
| chr10:31,921,706-32,135,284 | p11.22 | CN Gain | 213578 | 0.00 | 40.00 | -40.00 | 0.00 | 0.00 | ARHGAP12 |
| chr11:95,321,522-95,363,267 | q21 | CN Gain | 41745 | 0.00 | 40.00 | -40.00 | 0.00 | 0.00 | MAML2 |
| chr7:130,892,784-130,906,056 | q32.3 | CN Gain | 13272 | 83.33 | 31.11 | 52.22 | 0.00 | 0.00 |  |
| chr7:137,384,735-137,524,318 | q34 | CN Gain | 139583 | 83.33 | 31.11 | 52.22 | 0.00 | 0.00 | AKR1D1, MIR4468 |
| chr7:151,060,251-151,151,528 | q36.1 | CN Gain | 91277 | 83.33 | 31.11 | 52.22 | 0.00 | 0.00 | PRKAG2 |
| chr7:157,918,638-158,037,412 | q36.3 | CN Gain | 118774 | 83.33 | 31.11 | 52.22 | 0.00 | 0.00 | PTPRN2, MIR595 |
| chr7:27,963,526-28,086,959 | p15.2 - p15.1 | CN Gain | 123433 | 83.33 | 31.11 | 52.22 | 0.00 | 0.00 | JAZF1 |
| chr7:75,926,859-75,938,049 | q11.23 | CN Gain | 11190 | 83.33 | 31.11 | 52.22 | 0.00 | 0.00 | FDPSL2A, DTX2 |
| chr7:17,697,529-17,708,337 | p21.1 | CN Gain | 10808 | 66.67 | 15.56 | 51.11 | 0.00 | 0.00 |  |
| chr7:17,741,772-17,762,319 | p21.1 | CN Gain | 20547 | 66.67 | 15.56 | 51.11 | 0.00 | 0.00 |  |
| chr7:45,771,109-45,898,604 | p13 | CN Gain | 127495 | 66.67 | 15.56 | 51.11 | 0.00 | 0.00 | SEPT7P2, IGFBP1 |
| chr7:6,011,314-6,012,880 | p22.1 | CN Gain | 1566 | 66.67 | 15.56 | 51.11 | 0.00 | 0.00 | PMS2 |
| chr7:67,688,202-67,862,541 | q11.22 | CN Gain | 174339 | 61.90 | 11.11 | 50.79 | 0.00 | 0.00 |  |
| chr7:127,562,661-127,630,406 | q32.1 | CN Gain | 67745 | 80.95 | 28.89 | 52.06 | 0.00 | 0.00 |  |
| chr7:130,947,682-131,113,385 | q32.3 | CN Gain | 165703 | 80.95 | 28.89 | 52.06 | 0.00 | 0.00 |  |
| chr7:131,399,814-131,495,253 | q32.3 | CN Gain | 95439 | 80.95 | 28.89 | 52.06 | 0.00 | 0.00 | PLXNA4 |
| chr7:132,078,164-132,174,852 | q32.3 | CN Gain | 96688 | 80.95 | 28.89 | 52.06 | 0.00 | 0.00 | CHCHD3 |
| chr7:139,371,943-139,387,224 | q34 | CN Gain | 15281 | 80.95 | 28.89 | 52.06 | 0.00 | 0.00 | PARP12 |
| chr7:150,289,733-150,319,475 | q36.1 | CN Gain | 29742 | 80.95 | 28.89 | 52.06 | 0.00 | 0.00 | KCNH2, NOS3 |
| chr7:157,754,963-157,775,629 | q36.3 | CN Gain | 20666 | 80.95 | 28.89 | 52.06 | 0.00 | 0.00 | PTPRN2 |
| chr7:158,037,412-158,239,309 | q36.3 | CN Gain | 201897 | 80.95 | 28.89 | 52.06 | 0.00 | 0.00 | PTPRN2, MIR5707, NCAPG2, ESYT2 |
| chr7:2,201,310-2,324,081 | p22.2 | CN Gain | 122771 | 80.95 | 28.89 | 52.06 | 0.00 | 0.00 | MAD1L1, FTSJ2, NUDT1, SNX8 |
| chr7:73,074,666-73,156,969 | q11.23 | CN Gain | 82303 | 80.95 | 28.89 | 52.06 | 0.00 | 0.00 | ELN, LIMK1 |
| chr7:73,695,900-73,832,620 | q11.23 | CN Gain | 136720 | 80.95 | 28.89 | 52.06 | 0.00 | 0.00 | GTF2I, NCF1 |
| chr7:73,935,060-74,033,117 | q11.23 | CN Gain | 98057 | 80.95 | 28.89 | 52.06 | 0.00 | 0.00 | STAG3L2, PMS2P5, GATSL1 |
| chr7:75,325,708-75,519,648 | q11.23 | CN Gain | 193940 | 80.95 | 28.89 | 52.06 | 0.00 | 0.00 | RHBDD2, MIR4651, SNORA14A, POR, TMEM120A, STYXL1, MDH2 |
| chr7:76,882,978-76,939,066 | q11.23 | CN Gain | 56088 | 80.95 | 28.89 | 52.06 | 0.00 | 0.00 | PION |
| chr7:77,412,964-77,433,082 | q21.11 | CN Gain | 20118 | 80.95 | 28.89 | 52.06 | 0.00 | 0.00 | PHTF2 |
| chr7:10,707,069-10,834,367 | p21.3 | CN Gain | 127298 | 69.05 | 17.78 | 51.27 | 0.00 | 0.00 |  |
| chr7:15,734,169-15,885,002 | p21.1 | CN Gain | 150833 | 69.05 | 17.78 | 51.27 | 0.00 | 0.00 |  |
| chr7:15,932,227-15,999,370 | p21.1 | CN Gain | 67143 | 69.05 | 17.78 | 51.27 | 0.00 | 0.00 |  |
| chr7:17,466,280-17,471,992 | p21.1 | CN Gain | 5712 | 69.05 | 17.78 | 51.27 | 0.00 | 0.00 |  |
| chr7:7,224,058-7,254,395 | p21.3 | CN Gain | 30337 | 69.05 | 17.78 | 51.27 | 0.00 | 0.00 | C1GALT1 |
| chr7:7,387,070-7,414,047 | p21.3 | CN Gain | 26977 | 69.05 | 17.78 | 51.27 | 0.00 | 0.00 | COL28A1 |
| chr7:107,514,599-107,543,366 | q31.1 | CN Gain | 28767 | 78.57 | 26.67 | 51.90 | 0.00 | 0.00 | LAMB4 |
| chr7:11,586,087-11,587,221 | p21.3 | CN Gain | 1134 | 78.57 | 26.67 | 51.90 | 0.00 | 0.00 | THSD7A |
| chr7:126,975,103-127,010,131 | q32.1 | CN Gain | 35028 | 78.57 | 26.67 | 51.90 | 0.00 | 0.00 | GCC1 |
| chr7:154,224,828-154,276,703 | q36.2 | CN Gain | 51875 | 78.57 | 26.67 | 51.90 | 0.00 | 0.00 | DPP6 |
| chr7:154,368,146-154,597,596 | q36.2 | CN Gain | 229450 | 78.57 | 26.67 | 51.90 | 0.00 | 0.00 | LOC100132707, PAXIP1, LOC202781, LOC100128264, HTR5A |
| chr7:155,157,970-155,252,119 | q36.3 | CN Gain | 94149 | 78.57 | 26.67 | 51.90 | 0.00 | 0.00 | RBM33 |
| chr7:19,378,758-19,446,954 | p21.1 | CN Gain | 68196 | 78.57 | 26.67 | 51.90 | 0.00 | 0.00 |  |
| chr7:20,154,489-20,407,548 | p15.3 | CN Gain | 253059 | 78.57 | 26.67 | 51.90 | 0.00 | 0.00 | MACC1-AS1, MACC1, ITGB8 |
| chr7:26,197,014-26,456,874 | p15.2 | CN Gain | 259860 | 78.57 | 26.67 | 51.90 | 0.00 | 0.00 | HNRNPA2B1, CBX3, SNX10, LOC441204 |
| chr7:5,230,208-5,243,488 | p22.1 | CN Gain | 13280 | 78.57 | 26.67 | 51.90 | 0.00 | 0.00 | WIPI2 |
| chr7:72,962,357-73,046,160 | q11.23 | CN Gain | 83803 | 78.57 | 26.67 | 51.90 | 0.00 | 0.00 |  |
| chr7:75,280,376-75,325,708 | q11.23 | CN Gain | 45332 | 78.57 | 26.67 | 51.90 | 0.00 | 0.00 | CCL24 |
| chr7:10,395,609-10,433,367 | p21.3 | CN Gain | 37758 | 71.43 | 20.00 | 51.43 | 0.00 | 0.00 |  |
| chr7:10,904,491-10,966,682 | p21.3 | CN Gain | 62191 | 71.43 | 20.00 | 51.43 | 0.00 | 0.00 | NDUFA4 |
| chr7:101,522,364-101,886,798 | q22.1 | CN Gain | 364434 | 71.43 | 20.00 | 51.43 | 0.00 | 0.00 | CUX1, MIR4285, SH2B2, SPDYE6, LOC100289561, LOC100630923, PRKRIP1, ORAI2, ALKBH4 |
| chr7:103,362,053-103,374,174 | q22.1 | CN Gain | 12121 | 71.43 | 20.00 | 51.43 | 0.00 | 0.00 | RELN |
| chr7:106,305,696-106,448,841 | q22.3 | CN Gain | 143145 | 71.43 | 20.00 | 51.43 | 0.00 | 0.00 | PIK3CG |
| chr7:17,768,429-17,783,924 | p21.1 | CN Gain | 15495 | 71.43 | 20.00 | 51.43 | 0.00 | 0.00 |  |
| chr7:10,151,321-10,302,444 | p21.3 | CN Gain | 151123 | 76.19 | 24.44 | 51.75 | 0.00 | 0.00 |  |
| chr7:10,302,444-10,353,331 | p21.3 | CN Gain | 50887 | 73.81 | 22.22 | 51.59 | 0.00 | 0.00 |  |
| chr7:103,160,474-103,229,165 | q22.1 | CN Gain | 68691 | 73.81 | 22.22 | 51.59 | 0.00 | 0.00 | RELN |
| chr7:104,987,993-104,994,585 | q22.2 | CN Gain | 6592 | 76.19 | 24.44 | 51.75 | 0.00 | 0.00 | RINT1, EFCAB10 |
| chr7:104,994,585-105,025,576 | q22.2 | CN Gain | 30991 | 73.81 | 22.22 | 51.59 | 0.00 | 0.00 | RINT1, EFCAB10 |
| chr7:105,134,720-105,255,129 | q22.2 | CN Gain | 120409 | 73.81 | 22.22 | 51.59 | 0.00 | 0.00 | ATXN7L1 |
| chr7:105,263,509-105,362,214 | q22.2 | CN Gain | 98705 | 73.81 | 22.22 | 51.59 | 0.00 | 0.00 | ATXN7L1 |
| chr7:105,364,395-105,410,120 | q22.2 | CN Gain | 45725 | 73.81 | 22.22 | 51.59 | 0.00 | 0.00 | CDHR3 |
| chr7:105,428,092-105,434,634 | q22.2 | CN Gain | 6542 | 76.19 | 24.44 | 51.75 | 0.00 | 0.00 | CDHR3 |
| chr7:105,554,518-105,618,674 | q22.2 | CN Gain | 64156 | 76.19 | 24.44 | 51.75 | 0.00 | 0.00 |  |
| chr7:105,738,850-106,013,258 | q22.2 - q22.3 | CN Gain | 274408 | 73.81 | 22.22 | 51.59 | 0.00 | 0.00 |  |
| chr7:106,235,581-106,278,115 | q22.3 | CN Gain | 42534 | 73.81 | 22.22 | 51.59 | 0.00 | 0.00 |  |
| chr7:107,298,120-107,306,406 | q31.1 | CN Gain | 8286 | 73.81 | 22.22 | 51.59 | 0.00 | 0.00 |  |
| chr7:149,954,914-150,063,512 | q36.1 | CN Gain | 108598 | 76.19 | 24.44 | 51.75 | 0.00 | 0.00 | GIMAP6, GIMAP2, GIMAP1, GIMAP1-GIMAP5 |
| chr7:15,324,432-15,367,844 | p21.1 | CN Gain | 43412 | 76.19 | 24.44 | 51.75 | 0.00 | 0.00 | AGMO |
| chr7:15,577,150-15,636,775 | p21.1 | CN Gain | 59625 | 73.81 | 22.22 | 51.59 | 0.00 | 0.00 | MEOX2 |
| chr7:155,332,517-155,357,538 | q36.3 | CN Gain | 25021 | 76.19 | 24.44 | 51.75 | 0.00 | 0.00 |  |
| chr7:155,461,918-155,491,637 | q36.3 | CN Gain | 29719 | 76.19 | 24.44 | 51.75 | 0.00 | 0.00 |  |
| chr7:156,751,050-156,768,592 | q36.3 | CN Gain | 17542 | 76.19 | 24.44 | 51.75 | 0.00 | 0.00 | UBE3C |
| chr7:18,684,892-18,884,619 | p21.1 | CN Gain | 199727 | 73.81 | 22.22 | 51.59 | 0.00 | 0.00 | HDAC9 |
| chr7:19,312,666-19,319,384 | p21.1 | CN Gain | 6718 | 76.19 | 24.44 | 51.75 | 0.00 | 0.00 |  |
| chr7:2,756,752-2,791,657 | p22.2 | CN Gain | 34905 | 76.19 | 24.44 | 51.75 | 0.00 | 0.00 | GNA12 |
| chr7:77,578,075-77,609,277 | q21.11 | CN Gain | 31202 | 76.19 | 24.44 | 51.75 | 0.00 | 0.00 | MAGI2 |
| chr7:55,144,366-55,147,471 | p11.2 | CN Gain | 3105 | 88.10 | 37.78 | 50.32 | 0.00 | 0.00 | EGFR |
| chr11:95,367,230-95,371,155 | q21 | CN Gain | 3925 | 2.38 | 44.44 | -42.06 | 0.00 | 0.00 | MAML2 |
| chr8:89,409,440-89,412,317 | q21.3 | CN Gain | 2877 | 2.38 | 44.44 | -42.06 | 0.00 | 0.00 |  |
| chr11:95,389,205-95,561,919 | q21 | CN Gain | 172714 | 4.76 | 48.89 | -44.13 | 0.00 | 0.00 | MAML2 |
| chr7:0-309,845 | p22.3 | CN Gain | 309845 | 85.71 | 35.56 | 50.16 | 0.00 | 0.00 | FAM20C |
| chr7:1,103,464-1,133,376 | p22.3 | CN Gain | 29912 | 85.71 | 35.56 | 50.16 | 0.00 | 0.00 | C7orf50 |
| chr7:139,341,457-139,365,012 | q34 | CN Gain | 23555 | 85.71 | 35.56 | 50.16 | 0.00 | 0.00 | TBXAS1 |
| chr7:150,612,175-150,786,410 | q36.1 | CN Gain | 174235 | 85.71 | 35.56 | 50.16 | 0.00 | 0.00 | NUB1, WDR86, WDR86-AS1, MIR3907, CRYGN |
| chr7:54,866,255-54,945,565 | p11.2 | CN Gain | 79310 | 85.71 | 35.56 | 50.16 | 0.00 | 0.00 |  |
| chr7:70,590,464-70,645,882 | q11.22 | CN Gain | 55418 | 61.90 | 13.33 | 48.57 | 0.00 | 0.00 | WBSCR17 |
| chr7:71,079,378-71,098,009 | q11.22 | CN Gain | 18631 | 61.90 | 13.33 | 48.57 | 0.00 | 0.00 | CALN1 |
| chr7:71,148,476-71,440,758 | q11.22 | CN Gain | 292282 | 61.90 | 13.33 | 48.57 | 0.00 | 0.00 | CALN1 |
| chr10:14,015,410-14,081,121 | p13 | CN Gain | 65711 | 0.00 | 37.78 | -37.78 | 0.00 | 0.00 | FRMD4A |
| chr10:14,116,582-14,159,119 | p13 | CN Gain | 42537 | 0.00 | 37.78 | -37.78 | 0.00 | 0.00 | FRMD4A |
| chr10:27,042,163-27,062,397 | p12.1 | CN Gain | 20234 | 0.00 | 37.78 | -37.78 | 0.00 | 0.00 | PDSS1 |
| chr10:27,118,784-27,211,850 | p12.1 | CN Gain | 93066 | 0.00 | 37.78 | -37.78 | 0.00 | 0.00 | ABI1 |
| chr10:31,630,308-31,921,706 | p11.22 | CN Gain | 291398 | 0.00 | 37.78 | -37.78 | 0.00 | 0.00 | ZEB1-AS1, ZEB1 |
| chr10:32,135,284-32,175,041 | p11.22 | CN Gain | 39757 | 0.00 | 37.78 | -37.78 | 0.00 | 0.00 | ARHGAP12 |
| chr11:95,214,456-95,321,522 | q21 | CN Gain | 107066 | 0.00 | 37.78 | -37.78 | 0.00 | 0.00 | MTMR2 |
| chr7:136,352,529-136,385,963 | q33 | CN Gain | 33434 | 83.33 | 33.33 | 50.00 | 0.00 | 0.00 | LOC349160 |
| chr7:137,137,761-137,384,735 | q33 - q34 | CN Gain | 246974 | 83.33 | 33.33 | 50.00 | 0.00 | 0.00 | DGKI, CREB3L2, LOC100130880 |
| chr7:137,524,318-137,925,410 | q34 | CN Gain | 401092 | 83.33 | 33.33 | 50.00 | 0.00 | 0.00 | TRIM24 |
| chr7:139,365,975-139,371,019 | q34 | CN Gain | 5044 | 83.33 | 33.33 | 50.00 | 0.00 | 0.00 | TBXAS1, PARP12 |
| chr7:150,507,569-150,575,233 | q36.1 | CN Gain | 67664 | 83.33 | 33.33 | 50.00 | 0.00 | 0.00 | ASB10, ABCF2, CHPF2, MIR671, SMARCD3 |
| chr7:75,784,078-75,926,859 | q11.23 | CN Gain | 142781 | 83.33 | 33.33 | 50.00 | 0.00 | 0.00 | YWHAG, SRCRB4D, ZP3 |
| chr7:17,638,623-17,688,887 | p21.1 | CN Gain | 50264 | 64.29 | 15.56 | 48.73 | 0.00 | 0.00 |  |
| chr7:17,708,337-17,741,772 | p21.1 | CN Gain | 33435 | 64.29 | 15.56 | 48.73 | 0.00 | 0.00 |  |
| chr7:5,833,608-6,011,314 | p22.1 | CN Gain | 177706 | 64.29 | 15.56 | 48.73 | 0.00 | 0.00 | ZNF815P, OCM, CCZ1, RSPH10B, RSPH10B2, PMS2 |
| chr7:127,630,406-127,665,761 | q32.1 | CN Gain | 35355 | 80.95 | 31.11 | 49.84 | 0.00 | 0.00 | MIR129-1 |
| chr7:132,174,852-132,308,049 | q32.3 | CN Gain | 133197 | 80.95 | 31.11 | 49.84 | 0.00 | 0.00 | CHCHD3 |
| chr7:134,170,512-134,172,193 | q33 | CN Gain | 1681 | 80.95 | 31.11 | 49.84 | 0.00 | 0.00 | CALD1 |
| chr7:136,230,660-136,300,799 | q33 | CN Gain | 70139 | 80.95 | 31.11 | 49.84 | 0.00 | 0.00 | MIR490, CHRM2, LOC349160 |
| chr7:139,322,511-139,331,338 | q34 | CN Gain | 8827 | 80.95 | 31.11 | 49.84 | 0.00 | 0.00 | TBXAS1 |
| chr7:139,371,019-139,371,943 | q34 | CN Gain | 924 | 80.95 | 31.11 | 49.84 | 0.00 | 0.00 | PARP12 |
| chr7:150,319,475-150,336,934 | q36.1 | CN Gain | 17459 | 80.95 | 31.11 | 49.84 | 0.00 | 0.00 | NOS3 |
| chr7:151,151,528-151,152,187 | q36.1 | CN Gain | 659 | 80.95 | 31.11 | 49.84 | 0.00 | 0.00 | PRKAG2 |
| chr7:73,156,969-73,185,356 | q11.23 | CN Gain | 28387 | 80.95 | 31.11 | 49.84 | 0.00 | 0.00 | LIMK1 |
| chr7:73,471,675-73,695,900 | q11.23 | CN Gain | 224225 | 80.95 | 31.11 | 49.84 | 0.00 | 0.00 | GTF2IRD1 |
| chr7:73,832,620-73,935,060 | q11.23 | CN Gain | 102440 | 80.95 | 31.11 | 49.84 | 0.00 | 0.00 | NCF1, GTF2IRD2 |
| chr7:75,519,648-75,529,187 | q11.23 | CN Gain | 9539 | 80.95 | 31.11 | 49.84 | 0.00 | 0.00 | MDH2 |
| chr7:76,939,066-76,969,577 | q11.23 | CN Gain | 30511 | 80.95 | 31.11 | 49.84 | 0.00 | 0.00 |  |
| chr7:15,885,002-15,932,227 | p21.1 | CN Gain | 47225 | 66.67 | 17.78 | 48.89 | 0.00 | 0.00 |  |
| chr7:17,471,992-17,596,224 | p21.1 | CN Gain | 124232 | 66.67 | 17.78 | 48.89 | 0.00 | 0.00 |  |
| chr7:5,800,427-5,833,608 | p22.1 | CN Gain | 33181 | 66.67 | 17.78 | 48.89 | 0.00 | 0.00 | ZNF815P |
| chr7:107,021,102-107,038,994 | q22.3 | CN Gain | 17892 | 78.57 | 28.89 | 49.68 | 0.00 | 0.00 | BCAP29 |
| chr7:107,543,366-107,559,542 | q31.1 | CN Gain | 16176 | 78.57 | 28.89 | 49.68 | 0.00 | 0.00 | LAMB4 |
| chr7:11,587,221-11,817,736 | p21.3 | CN Gain | 230515 | 78.57 | 28.89 | 49.68 | 0.00 | 0.00 | THSD7A |
| chr7:127,010,131-127,041,286 | q32.1 | CN Gain | 31155 | 78.57 | 28.89 | 49.68 | 0.00 | 0.00 | GCC1, ARF5, FSCN3, PAX4 |
| chr7:127,449,378-127,562,661 | q32.1 | CN Gain | 113283 | 78.57 | 28.89 | 49.68 | 0.00 | 0.00 | SND1, LRRC4, MIR593 |
| chr7:131,113,385-131,399,814 | q32.3 | CN Gain | 286429 | 78.57 | 28.89 | 49.68 | 0.00 | 0.00 |  |
| chr7:133,683,181-133,754,462 | q33 | CN Gain | 71281 | 78.57 | 28.89 | 49.68 | 0.00 | 0.00 |  |
| chr7:139,387,224-139,545,515 | q34 | CN Gain | 158291 | 78.57 | 28.89 | 49.68 | 0.00 | 0.00 | PARP12, JHDM1D, LOC100134229 |
| chr7:154,160,644-154,224,828 | q36.2 | CN Gain | 64184 | 78.57 | 28.89 | 49.68 | 0.00 | 0.00 | DPP6 |
| chr7:158,239,309-158,821,424 | q36.3 | CN Gain | 582115 | 78.57 | 28.89 | 49.68 | 0.00 | 0.00 | ESYT2, WDR60, LINC00689, VIPR2 |
| chr7:2,324,081-2,336,756 | p22.2 | CN Gain | 12675 | 78.57 | 28.89 | 49.68 | 0.00 | 0.00 |  |
| chr7:10,834,367-10,904,491 | p21.3 | CN Gain | 70124 | 69.05 | 20.00 | 49.05 | 0.00 | 0.00 |  |
| chr7:101,494,484-101,522,364 | q22.1 | CN Gain | 27880 | 69.05 | 20.00 | 49.05 | 0.00 | 0.00 | CUX1 |
| chr7:101,886,798-102,147,051 | q22.1 | CN Gain | 260253 | 69.05 | 20.00 | 49.05 | 0.00 | 0.00 | ALKBH4, MIR5090, LRWD1, MIR4467, POLR2J, SPDYE2, SPDYE2L, RASA4, POLR2J3, UPK3BL, POLR2J2, SPDYE2, SPDYE2L |
| chr7:102,268,551-102,280,503 | q22.1 | CN Gain | 11952 | 69.05 | 20.00 | 49.05 | 0.00 | 0.00 | FBXL13 |
| chr7:103,294,730-103,362,053 | q22.1 | CN Gain | 67323 | 69.05 | 20.00 | 49.05 | 0.00 | 0.00 | RELN |
| chr7:103,374,174-103,384,607 | q22.1 | CN Gain | 10433 | 69.05 | 20.00 | 49.05 | 0.00 | 0.00 | RELN |
| chr7:7,414,047-7,437,148 | p21.3 | CN Gain | 23101 | 69.05 | 20.00 | 49.05 | 0.00 | 0.00 | COL28A1 |
| chr7:103,153,997-103,160,474 | q22.1 | CN Gain | 6477 | 71.43 | 22.22 | 49.21 | 0.00 | 0.00 | RELN |
| chr7:103,229,165-103,290,426 | q22.1 | CN Gain | 61261 | 71.43 | 22.22 | 49.21 | 0.00 | 0.00 | RELN |
| chr7:105,255,129-105,263,509 | q22.2 | CN Gain | 8380 | 71.43 | 22.22 | 49.21 | 0.00 | 0.00 | ATXN7L1 |
| chr7:106,013,258-106,235,581 | q22.3 | CN Gain | 222323 | 71.43 | 22.22 | 49.21 | 0.00 | 0.00 | CCDC71L |
| chr7:106,448,841-106,451,491 | q22.3 | CN Gain | 2650 | 71.43 | 22.22 | 49.21 | 0.00 | 0.00 |  |
| chr7:107,044,923-107,112,019 | q22.3 | CN Gain | 67096 | 73.81 | 24.44 | 49.37 | 0.00 | 0.00 | BCAP29, SLC26A4-AS1, SLC26A4 |
| chr7:107,292,943-107,298,120 | q31.1 | CN Gain | 5177 | 71.43 | 22.22 | 49.21 | 0.00 | 0.00 |  |
| chr7:107,306,406-107,316,687 | q31.1 | CN Gain | 10281 | 73.81 | 24.44 | 49.37 | 0.00 | 0.00 |  |
| chr7:107,325,545-107,514,599 | q31.1 | CN Gain | 189054 | 76.19 | 26.67 | 49.52 | 0.00 | 0.00 | DLD, LAMB1, LAMB4 |
| chr7:11,225,515-11,371,445 | p21.3 | CN Gain | 145930 | 76.19 | 26.67 | 49.52 | 0.00 | 0.00 |  |
| chr7:11,490,785-11,586,087 | p21.3 | CN Gain | 95302 | 76.19 | 26.67 | 49.52 | 0.00 | 0.00 | THSD7A |
| chr7:11,876,769-11,879,365 | p21.3 | CN Gain | 2596 | 76.19 | 26.67 | 49.52 | 0.00 | 0.00 |  |
| chr7:11,881,317-12,089,037 | p21.3 | CN Gain | 207720 | 73.81 | 24.44 | 49.37 | 0.00 | 0.00 |  |
| chr7:126,960,124-126,975,103 | q32.1 | CN Gain | 14979 | 76.19 | 26.67 | 49.52 | 0.00 | 0.00 |  |
| chr7:148,240,747-148,277,844 | q36.1 | CN Gain | 37097 | 73.81 | 24.44 | 49.37 | 0.00 | 0.00 |  |
| chr7:149,844,348-149,954,914 | q36.1 | CN Gain | 110566 | 73.81 | 24.44 | 49.37 | 0.00 | 0.00 | GIMAP7, GIMAP4, GIMAP6 |
| chr7:15,263,573-15,324,432 | p21.1 | CN Gain | 60859 | 76.19 | 26.67 | 49.52 | 0.00 | 0.00 | AGMO |
| chr7:15,367,844-15,414,704 | p21.1 | CN Gain | 46860 | 73.81 | 24.44 | 49.37 | 0.00 | 0.00 | AGMO |
| chr7:15,430,727-15,577,150 | p21.1 | CN Gain | 146423 | 71.43 | 22.22 | 49.21 | 0.00 | 0.00 | AGMO |
| chr7:150,794,343-150,795,792 | q36.1 | CN Gain | 1449 | 88.10 | 40.00 | 48.10 | 0.00 | 0.00 | RHEB |
| chr7:154,597,596-154,640,599 | q36.2 | CN Gain | 43003 | 76.19 | 26.67 | 49.52 | 0.00 | 0.00 |  |
| chr7:154,996,334-155,157,970 | q36.3 | CN Gain | 161636 | 76.19 | 26.67 | 49.52 | 0.00 | 0.00 | CNPY1, RBM33 |
| chr7:155,252,119-155,332,517 | q36.3 | CN Gain | 80398 | 73.81 | 24.44 | 49.37 | 0.00 | 0.00 | RBM33, SHH |
| chr7:155,491,637-155,500,299 | q36.3 | CN Gain | 8662 | 73.81 | 24.44 | 49.37 | 0.00 | 0.00 |  |
| chr7:156,638,258-156,751,050 | q36.3 | CN Gain | 112792 | 76.19 | 26.67 | 49.52 | 0.00 | 0.00 | UBE3C |
| chr7:17,783,924-17,789,266 | p21.1 | CN Gain | 5342 | 71.43 | 22.22 | 49.21 | 0.00 | 0.00 |  |
| chr7:17,929,857-18,275,054 | p21.1 | CN Gain | 345197 | 73.81 | 24.44 | 49.37 | 0.00 | 0.00 | SNX13, PRPS1L1, HDAC9 |
| chr7:18,676,236-18,684,892 | p21.1 | CN Gain | 8656 | 73.81 | 24.44 | 49.37 | 0.00 | 0.00 | HDAC9 |
| chr7:19,446,954-19,510,555 | p21.1 - p15.3 | CN Gain | 63601 | 76.19 | 26.67 | 49.52 | 0.00 | 0.00 |  |
| chr7:2,336,756-2,380,187 | p22.2 | CN Gain | 43431 | 76.19 | 26.67 | 49.52 | 0.00 | 0.00 | EIF3B |
| chr7:2,742,527-2,756,752 | p22.2 | CN Gain | 14225 | 76.19 | 26.67 | 49.52 | 0.00 | 0.00 | GNA12 |
| chr7:5,243,488-5,483,402 | p22.1 | CN Gain | 239914 | 76.19 | 26.67 | 49.52 | 0.00 | 0.00 | SLC29A4, TNRC18, FBXL18 |
| chr7:5,483,402-5,495,408 | p22.1 | CN Gain | 12006 | 73.81 | 24.44 | 49.37 | 0.00 | 0.00 | FBXL18 |
| chr7:5,685,837-5,703,263 | p22.1 | CN Gain | 17426 | 73.81 | 24.44 | 49.37 | 0.00 | 0.00 | RNF216-IT1, RNF216 |
| chr7:5,703,263-5,711,439 | p22.1 | CN Gain | 8176 | 71.43 | 22.22 | 49.21 | 0.00 | 0.00 | RNF216 |
| chr7:55,050,384-55,060,282 | p11.2 | CN Gain | 9898 | 88.10 | 40.00 | 48.10 | 0.00 | 0.00 | EGFR |
| chr7:55,141,816-55,144,366 | p11.2 | CN Gain | 2550 | 88.10 | 40.00 | 48.10 | 0.00 | 0.00 | EGFR |
| chr7:77,609,277-77,629,163 | q21.11 | CN Gain | 19886 | 76.19 | 26.67 | 49.52 | 0.00 | 0.00 | MAGI2 |
| chr7:8,699,079-8,756,003 | p21.3 | CN Gain | 56924 | 76.19 | 26.67 | 49.52 | 0.00 | 0.00 | NXPH1 |
| chr7:8,972,539-9,004,583 | p21.3 | CN Gain | 32044 | 76.19 | 26.67 | 49.52 | 0.00 | 0.00 |  |
| chr7:85,443,979-85,855,426 | q21.11 | CN Gain | 411447 | 73.81 | 24.44 | 49.37 | 0.00 | 0.00 |  |
| chr7:85,941,701-86,101,931 | q21.11 | CN Gain | 160230 | 76.19 | 26.67 | 49.52 | 0.00 | 0.00 |  |
| chr7:89,329,549-89,390,936 | q21.13 | CN Gain | 61387 | 73.81 | 24.44 | 49.37 | 0.00 | 0.00 |  |
| chr7:89,546,777-89,686,772 | q21.13 | CN Gain | 139995 | 73.81 | 24.44 | 49.37 | 0.00 | 0.00 | DPY19L2P4, STEAP1, STEAP2 |
| chr7:9,923,433-10,151,321 | p21.3 | CN Gain | 227888 | 76.19 | 26.67 | 49.52 | 0.00 | 0.00 |  |
| chr7:95,162,536-95,213,763 | q21.3 | CN Gain | 51227 | 73.81 | 24.44 | 49.37 | 0.00 | 0.00 |  |
| chr11:95,363,267-95,367,230 | q21 | CN Gain | 3963 | 2.38 | 42.22 | -39.84 | 0.00 | 0.00 | MAML2 |
| chr8:89,412,317-89,427,803 | q21.3 | CN Gain | 15486 | 2.38 | 42.22 | -39.84 | 0.00 | 0.00 |  |
| chr11:95,371,155-95,389,205 | q21 | CN Gain | 18050 | 4.76 | 46.67 | -41.90 | 0.00 | 0.00 | MAML2 |
| chr7:150,585,156-150,612,175 | q36.1 | CN Gain | 27019 | 85.71 | 37.78 | 47.94 | 0.00 | 0.00 | SMARCD3 |
| chr7:309,845-414,658 | p22.3 | CN Gain | 104813 | 85.71 | 37.78 | 47.94 | 0.00 | 0.00 |  |
| chr7:54,945,565-55,026,725 | p11.2 | CN Gain | 81160 | 85.71 | 37.78 | 47.94 | 0.00 | 0.00 |  |
| chr10:14,159,119-14,160,782 | p13 | CN Gain | 1663 | 0.00 | 35.56 | -35.56 | 0.00 | 0.00 | FRMD4A |
| chr10:31,596,961-31,630,308 | p11.22 | CN Gain | 33347 | 0.00 | 35.56 | -35.56 | 0.00 | 0.00 |  |
| chr10:32,175,041-32,197,012 | p11.22 | CN Gain | 21971 | 0.00 | 35.56 | -35.56 | 0.00 | 0.00 | ARHGAP12 |
| chr11:105,108,545-105,197,082 | q22.3 | CN Gain | 88537 | 0.00 | 35.56 | -35.56 | 0.00 | 0.00 | GRIA4 |
| chr11:95,145,784-95,214,456 | q21 | CN Gain | 68672 | 0.00 | 35.56 | -35.56 | 0.00 | 0.00 | FAM76B, CEP57, MTMR2 |
| chr7:130,845,627-130,892,784 | q32.3 | CN Gain | 47157 | 83.33 | 35.56 | 47.78 | 0.00 | 0.00 | PODXL |
| chr7:132,513,831-132,558,439 | q33 | CN Gain | 44608 | 83.33 | 35.56 | 47.78 | 0.00 | 0.00 |  |
| chr7:136,385,963-136,473,685 | q33 | CN Gain | 87722 | 83.33 | 35.56 | 47.78 | 0.00 | 0.00 | LOC349160 |
| chr7:136,803,900-137,137,761 | q33 | CN Gain | 333861 | 83.33 | 35.56 | 47.78 | 0.00 | 0.00 | DGKI |
| chr7:150,575,233-150,585,156 | q36.1 | CN Gain | 9923 | 83.33 | 35.56 | 47.78 | 0.00 | 0.00 | SMARCD3 |
| chr7:75,783,498-75,784,078 | q11.23 | CN Gain | 580 | 83.33 | 35.56 | 47.78 | 0.00 | 0.00 |  |
| chr7:82,421,434-82,596,437 | q21.11 | CN Gain | 175003 | 83.33 | 35.56 | 47.78 | 0.00 | 0.00 | PCLO |
| chr7:17,688,887-17,697,529 | p21.1 | CN Gain | 8642 | 61.90 | 15.56 | 46.35 | 0.00 | 0.00 |  |
| chr7:127,975,435-128,197,042 | q32.1 | CN Gain | 221607 | 80.95 | 33.33 | 47.62 | 0.00 | 0.00 | FLJ45340, FAM71F2, FAM71F1, CALU |
| chr7:132,308,049-132,328,807 | q32.3 | CN Gain | 20758 | 80.95 | 33.33 | 47.62 | 0.00 | 0.00 | CHCHD3 |
| chr7:132,583,865-133,006,687 | q33 | CN Gain | 422822 | 80.95 | 33.33 | 47.62 | 0.00 | 0.00 | EXOC4 |
| chr7:134,172,193-134,200,563 | q33 | CN Gain | 28370 | 80.95 | 33.33 | 47.62 | 0.00 | 0.00 | CALD1 |
| chr7:134,227,942-134,304,697 | q33 | CN Gain | 76755 | 80.95 | 33.33 | 47.62 | 0.00 | 0.00 | CALD1 |
| chr7:136,300,799-136,352,529 | q33 | CN Gain | 51730 | 80.95 | 33.33 | 47.62 | 0.00 | 0.00 | CHRM2, LOC349160 |
| chr7:137,925,410-137,945,778 | q34 | CN Gain | 20368 | 80.95 | 33.33 | 47.62 | 0.00 | 0.00 | SVOPL |
| chr7:138,960,534-138,973,822 | q34 | CN Gain | 13288 | 80.95 | 33.33 | 47.62 | 0.00 | 0.00 | HIPK2 |
| chr7:150,336,934-150,363,570 | q36.1 | CN Gain | 26636 | 80.95 | 33.33 | 47.62 | 0.00 | 0.00 | NOS3, ATG9B, ABCB8 |
| chr7:150,383,596-150,507,569 | q36.1 | CN Gain | 123973 | 80.95 | 33.33 | 47.62 | 0.00 | 0.00 | CDK5, SLC4A2, FASTK, TMUB1, AGAP3, GBX1, ASB10 |
| chr7:150,906,772-150,910,701 | q36.1 | CN Gain | 3929 | 80.95 | 33.33 | 47.62 | 0.00 | 0.00 | PRKAG2 |
| chr7:153,849,788-154,064,256 | q36.2 | CN Gain | 214468 | 80.95 | 33.33 | 47.62 | 0.00 | 0.00 | DPP6 |
| chr7:73,185,356-73,265,885 | q11.23 | CN Gain | 80529 | 80.95 | 33.33 | 47.62 | 0.00 | 0.00 | EIF4H, MIR590, LAT2 |
| chr7:73,296,210-73,471,675 | q11.23 | CN Gain | 175465 | 80.95 | 33.33 | 47.62 | 0.00 | 0.00 | RFC2, CLIP2 |
| chr7:75,529,187-75,534,140 | q11.23 | CN Gain | 4953 | 80.95 | 33.33 | 47.62 | 0.00 | 0.00 | MDH2 |
| chr7:76,969,577-77,412,964 | q11.23 - q21.11 | CN Gain | 443387 | 80.95 | 33.33 | 47.62 | 0.00 | 0.00 | PTPN12, RSBN1L-AS1, RSBN1L, TMEM60, PHTF2 |
| chr7:17,596,224-17,638,623 | p21.1 | CN Gain | 42399 | 64.29 | 17.78 | 46.51 | 0.00 | 0.00 |  |
| chr7:106,771,769-106,861,775 | q22.3 | CN Gain | 90006 | 78.57 | 31.11 | 47.46 | 0.00 | 0.00 | COG5 |
| chr7:127,359,691-127,449,378 | q32.1 | CN Gain | 89687 | 78.57 | 31.11 | 47.46 | 0.00 | 0.00 | SND1, SND1-IT1 |
| chr7:13,650,153-13,670,085 | p21.2 | CN Gain | 19932 | 78.57 | 31.11 | 47.46 | 0.00 | 0.00 |  |
| chr7:133,754,462-133,816,395 | q33 | CN Gain | 61933 | 78.57 | 31.11 | 47.46 | 0.00 | 0.00 | AKR1B1 |
| chr7:135,804,466-136,230,660 | q33 | CN Gain | 426194 | 78.57 | 31.11 | 47.46 | 0.00 | 0.00 | CHRM2 |
| chr7:139,163,036-139,322,511 | q34 | CN Gain | 159475 | 78.57 | 31.11 | 47.46 | 0.00 | 0.00 | TBXAS1 |
| chr7:14,918,106-14,977,053 | p21.2 | CN Gain | 58947 | 78.57 | 31.11 | 47.46 | 0.00 | 0.00 |  |
| chr7:86,971,354-87,016,825 | q21.12 | CN Gain | 45471 | 78.57 | 31.11 | 47.46 | 0.00 | 0.00 | ABCB1 |
| chr7:102,147,051-102,268,551 | q22.1 | CN Gain | 121500 | 66.67 | 20.00 | 46.67 | 0.00 | 0.00 | FAM185A, FBXL13 |
| chr7:5,779,129-5,800,427 | p22.1 | CN Gain | 21298 | 66.67 | 20.00 | 46.67 | 0.00 | 0.00 | RNF216 |
| chr7:55,060,282-55,141,816 | p11.2 | CN Gain | 81534 | 88.10 | 42.22 | 45.87 | 0.00 | 0.00 | EGFR |
| chr7:70,645,882-70,782,552 | q11.22 | CN Gain | 136670 | 59.52 | 13.33 | 46.19 | 0.00 | 0.00 | WBSCR17 |
| chr7:70,813,658-70,954,838 | q11.22 | CN Gain | 141180 | 59.52 | 13.33 | 46.19 | 0.00 | 0.00 | WBSCR17, CALN1 |
| chr7:71,069,600-71,079,378 | q11.22 | CN Gain | 9778 | 59.52 | 13.33 | 46.19 | 0.00 | 0.00 | CALN1 |
| chr10:13,957,606-14,007,788 | p13 | CN Gain | 50182 | 2.38 | 40.00 | -37.62 | 0.00 | 0.00 | FRMD4A |
| chr11:82,107,748-82,172,974 | q14.1 | CN Gain | 65226 | 2.38 | 40.00 | -37.62 | 0.00 | 0.00 | FAM181B |
| chr11:95,561,919-95,639,629 | q21 | CN Gain | 77710 | 7.14 | 48.89 | -41.75 | 0.00 | 0.00 | MAML2 |
| chr7:10,966,682-10,982,709 | p21.3 | CN Gain | 16027 | 71.43 | 24.44 | 46.98 | 0.00 | 0.00 | PHF14 |
| chr7:101,355,347-101,494,484 | q22.1 | CN Gain | 139137 | 69.05 | 22.22 | 46.83 | 0.00 | 0.00 | CUX1 |
| chr7:102,280,503-102,515,628 | q22.1 | CN Gain | 235125 | 69.05 | 22.22 | 46.83 | 0.00 | 0.00 | LRRC17, FBXL13, ARMC10 |
| chr7:103,132,496-103,153,997 | q22.1 | CN Gain | 21501 | 69.05 | 22.22 | 46.83 | 0.00 | 0.00 | RELN |
| chr7:103,290,426-103,294,730 | q22.1 | CN Gain | 4304 | 69.05 | 22.22 | 46.83 | 0.00 | 0.00 | RELN |
| chr7:103,695,072-103,750,190 | q22.1 | CN Gain | 55118 | 73.81 | 26.67 | 47.14 | 0.00 | 0.00 |  |
| chr7:104,894,417-104,987,993 | q22.2 | CN Gain | 93576 | 76.19 | 28.89 | 47.30 | 0.00 | 0.00 | PUS7, RINT1 |
| chr7:106,451,491-106,550,268 | q22.3 | CN Gain | 98777 | 71.43 | 24.44 | 46.98 | 0.00 | 0.00 | PRKAR2B |
| chr7:107,112,019-107,177,320 | q22.3 | CN Gain | 65301 | 71.43 | 24.44 | 46.98 | 0.00 | 0.00 | SLC26A4, CBLL1 |
| chr7:107,185,501-107,189,334 | q22.3 | CN Gain | 3833 | 69.05 | 22.22 | 46.83 | 0.00 | 0.00 | CBLL1 |
| chr7:107,282,605-107,292,943 | q31.1 | CN Gain | 10338 | 69.05 | 22.22 | 46.83 | 0.00 | 0.00 |  |
| chr7:107,316,687-107,325,545 | q31.1 | CN Gain | 8858 | 73.81 | 26.67 | 47.14 | 0.00 | 0.00 | DLD |
| chr7:107,559,542-107,570,960 | q31.1 | CN Gain | 11418 | 76.19 | 28.89 | 47.30 | 0.00 | 0.00 |  |
| chr7:11,211,524-11,225,515 | p21.3 | CN Gain | 13991 | 76.19 | 28.89 | 47.30 | 0.00 | 0.00 |  |
| chr7:11,371,445-11,490,785 | p21.3 | CN Gain | 119340 | 76.19 | 28.89 | 47.30 | 0.00 | 0.00 | THSD7A |
| chr7:11,817,736-11,876,769 | p21.3 | CN Gain | 59033 | 76.19 | 28.89 | 47.30 | 0.00 | 0.00 | THSD7A |
| chr7:11,879,365-11,881,317 | p21.3 | CN Gain | 1952 | 73.81 | 26.67 | 47.14 | 0.00 | 0.00 |  |
| chr7:12,089,037-12,198,433 | p21.3 | CN Gain | 109396 | 73.81 | 26.67 | 47.14 | 0.00 | 0.00 |  |
| chr7:126,550,310-126,577,210 | q31.33 | CN Gain | 26900 | 73.81 | 26.67 | 47.14 | 0.00 | 0.00 | GRM8 |
| chr7:126,661,477-126,960,124 | q31.33 - q32.1 | CN Gain | 298647 | 73.81 | 26.67 | 47.14 | 0.00 | 0.00 | GRM8, ZNF800 |
| chr7:127,041,286-127,076,067 | q32.1 | CN Gain | 34781 | 76.19 | 28.89 | 47.30 | 0.00 | 0.00 | PAX4 |
| chr7:133,663,077-133,683,181 | q33 | CN Gain | 20104 | 76.19 | 28.89 | 47.30 | 0.00 | 0.00 |  |
| chr7:139,545,515-139,806,629 | q34 | CN Gain | 261114 | 76.19 | 28.89 | 47.30 | 0.00 | 0.00 | SLC37A3, RAB19, MKRN1 |
| chr7:141,103,720-141,125,010 | q34 | CN Gain | 21290 | 76.19 | 28.89 | 47.30 | 0.00 | 0.00 | TAS2R3, TAS2R4 |
| chr7:142,478,924-142,610,029 | q34 | CN Gain | 131105 | 71.43 | 24.44 | 46.98 | 0.00 | 0.00 | PIP, TAS2R39 |
| chr7:142,729,350-142,811,982 | q34 - q35 | CN Gain | 82632 | 73.81 | 26.67 | 47.14 | 0.00 | 0.00 | CLCN1, FAM131B, ZYX, EPHA1 |
| chr7:147,889,037-147,963,025 | q36.1 | CN Gain | 73988 | 73.81 | 26.67 | 47.14 | 0.00 | 0.00 | C7orf33 |
| chr7:148,080,289-148,240,747 | q36.1 | CN Gain | 160458 | 73.81 | 26.67 | 47.14 | 0.00 | 0.00 | CUL1, EZH2 |
| chr7:148,277,844-148,303,811 | q36.1 | CN Gain | 25967 | 71.43 | 24.44 | 46.98 | 0.00 | 0.00 |  |
| chr7:148,396,034-148,535,604 | q36.1 | CN Gain | 139570 | 71.43 | 24.44 | 46.98 | 0.00 | 0.00 | ZNF786, ZNF425, ZNF398, ZNF282 |
| chr7:149,773,216-149,844,348 | q36.1 | CN Gain | 71132 | 71.43 | 24.44 | 46.98 | 0.00 | 0.00 | LOC285972, GIMAP8, GIMAP7 |
| chr7:15,414,704-15,430,727 | p21.1 | CN Gain | 16023 | 71.43 | 24.44 | 46.98 | 0.00 | 0.00 | AGMO |
| chr7:151,167,892-151,214,176 | q36.1 | CN Gain | 46284 | 76.19 | 28.89 | 47.30 | 0.00 | 0.00 | PRKAG2, PRKAG2-AS1 |
| chr7:151,256,167-151,263,015 | q36.1 | CN Gain | 6848 | 73.81 | 26.67 | 47.14 | 0.00 | 0.00 |  |
| chr7:154,125,121-154,134,196 | q36.2 | CN Gain | 9075 | 76.19 | 28.89 | 47.30 | 0.00 | 0.00 | DPP6 |
| chr7:154,138,790-154,160,644 | q36.2 | CN Gain | 21854 | 76.19 | 28.89 | 47.30 | 0.00 | 0.00 | DPP6 |
| chr7:154,640,599-154,996,334 | q36.2 - q36.3 | CN Gain | 355735 | 73.81 | 26.67 | 47.14 | 0.00 | 0.00 | INSIG1, EN2, CNPY1 |
| chr7:155,500,299-155,505,297 | q36.3 | CN Gain | 4998 | 71.43 | 24.44 | 46.98 | 0.00 | 0.00 |  |
| chr7:156,503,804-156,638,258 | q36.3 | CN Gain | 134454 | 73.81 | 26.67 | 47.14 | 0.00 | 0.00 | UBE3C |
| chr7:17,789,266-17,929,857 | p21.1 | CN Gain | 140591 | 71.43 | 24.44 | 46.98 | 0.00 | 0.00 | SNX13 |
| chr7:18,275,054-18,676,236 | p21.1 | CN Gain | 401182 | 73.81 | 26.67 | 47.14 | 0.00 | 0.00 | HDAC9 |
| chr7:19,510,555-19,518,565 | p15.3 | CN Gain | 8010 | 76.19 | 28.89 | 47.30 | 0.00 | 0.00 |  |
| chr7:19,590,300-19,762,917 | p15.3 | CN Gain | 172617 | 73.81 | 26.67 | 47.14 | 0.00 | 0.00 | TWISTNB, MIR3146, TMEM196 |
| chr7:2,380,187-2,742,527 | p22.2 | CN Gain | 362340 | 76.19 | 28.89 | 47.30 | 0.00 | 0.00 | EIF3B, CHST12, LFNG, MIR4648, BRAT1, IQCE, TTYH3, AMZ1, GNA12 |
| chr7:5,495,408-5,685,837 | p22.1 | CN Gain | 190429 | 73.81 | 26.67 | 47.14 | 0.00 | 0.00 | FBXL18, MIR589, ACTB, FSCN1, RNF216-IT1, RNF216 |
| chr7:5,711,439-5,779,129 | p22.1 | CN Gain | 67690 | 69.05 | 22.22 | 46.83 | 0.00 | 0.00 | RNF216 |
| chr7:7,437,148-7,470,264 | p21.3 | CN Gain | 33116 | 69.05 | 22.22 | 46.83 | 0.00 | 0.00 | COL28A1 |
| chr7:77,629,163-77,722,079 | q21.11 | CN Gain | 92916 | 76.19 | 28.89 | 47.30 | 0.00 | 0.00 | MAGI2 |
| chr7:8,694,332-8,699,079 | p21.3 | CN Gain | 4747 | 76.19 | 28.89 | 47.30 | 0.00 | 0.00 | NXPH1 |
| chr7:8,756,003-8,972,539 | p21.3 | CN Gain | 216536 | 73.81 | 26.67 | 47.14 | 0.00 | 0.00 | NXPH1 |
| chr7:85,439,310-85,443,979 | q21.11 | CN Gain | 4669 | 71.43 | 24.44 | 46.98 | 0.00 | 0.00 |  |
| chr7:85,855,426-85,941,701 | q21.11 | CN Gain | 86275 | 73.81 | 26.67 | 47.14 | 0.00 | 0.00 |  |
| chr7:86,101,931-86,186,454 | q21.11 | CN Gain | 84523 | 76.19 | 28.89 | 47.30 | 0.00 | 0.00 | GRM3 |
| chr7:87,019,952-87,039,933 | q21.12 | CN Gain | 19981 | 76.19 | 28.89 | 47.30 | 0.00 | 0.00 | ABCB1 |
| chr7:88,340,749-88,395,501 | q21.13 | CN Gain | 54752 | 76.19 | 28.89 | 47.30 | 0.00 | 0.00 | ZNF804B |
| chr7:89,223,538-89,329,549 | q21.13 | CN Gain | 106011 | 71.43 | 24.44 | 46.98 | 0.00 | 0.00 |  |
| chr7:89,390,936-89,546,777 | q21.13 | CN Gain | 155841 | 73.81 | 26.67 | 47.14 | 0.00 | 0.00 |  |
| chr7:89,686,772-89,691,066 | q21.13 | CN Gain | 4294 | 73.81 | 26.67 | 47.14 | 0.00 | 0.00 | STEAP2 |
| chr7:9,004,583-9,143,479 | p21.3 | CN Gain | 138896 | 73.81 | 26.67 | 47.14 | 0.00 | 0.00 |  |
| chr7:9,202,760-9,597,808 | p21.3 | CN Gain | 395048 | 76.19 | 28.89 | 47.30 | 0.00 | 0.00 |  |
| chr7:9,640,411-9,826,506 | p21.3 | CN Gain | 186095 | 76.19 | 28.89 | 47.30 | 0.00 | 0.00 | PER4 |
| chr7:9,838,969-9,923,433 | p21.3 | CN Gain | 84464 | 73.81 | 26.67 | 47.14 | 0.00 | 0.00 |  |
| chr7:90,592,344-90,629,235 | q21.13 | CN Gain | 36891 | 73.81 | 26.67 | 47.14 | 0.00 | 0.00 | CDK14 |
| chr7:90,669,833-90,672,550 | q21.13 | CN Gain | 2717 | 76.19 | 28.89 | 47.30 | 0.00 | 0.00 | CDK14 |
| chr7:92,626,086-92,679,897 | q21.3 | CN Gain | 53811 | 73.81 | 26.67 | 47.14 | 0.00 | 0.00 | HEPACAM2 |
| chr7:93,720,351-93,852,743 | q21.3 | CN Gain | 132392 | 73.81 | 26.67 | 47.14 | 0.00 | 0.00 |  |
| chr7:95,161,927-95,162,536 | q21.3 | CN Gain | 609 | 73.81 | 26.67 | 47.14 | 0.00 | 0.00 |  |
| chr7:95,213,763-95,265,303 | q21.3 | CN Gain | 51540 | 73.81 | 26.67 | 47.14 | 0.00 | 0.00 | DYNC1I1 |
| chr10:11,280,583-11,385,787 | p14 | CN Gain | 105204 | 0.00 | 33.33 | -33.33 | 0.00 | 0.00 | CELF2 |
| chr10:14,160,782-14,168,213 | p13 | CN Gain | 7431 | 0.00 | 33.33 | -33.33 | 0.00 | 0.00 | FRMD4A |
| chr11:105,006,086-105,108,545 | q22.3 | CN Gain | 102459 | 0.00 | 33.33 | -33.33 | 0.00 | 0.00 | GRIA4 |
| chr11:117,938,763-117,957,479 | q23.3 | CN Gain | 18716 | 0.00 | 33.33 | -33.33 | 0.00 | 0.00 | IFT46, ARCN1 |
| chr7:150,795,792-150,796,233 | q36.1 | CN Gain | 441 | 85.71 | 40.00 | 45.71 | 0.00 | 0.00 | RHEB |
| chr7:414,658-1,103,464 | p22.3 | CN Gain | 688806 | 85.71 | 40.00 | 45.71 | 0.00 | 0.00 | PDGFA, FLJ44511, PRKAR1B, HEATR2, SUN1, GET4, ADAP1, COX19, CYP2W1, MIR339, GPR146, C7orf50, GPER |
| chr7:55,026,725-55,050,384 | p11.2 | CN Gain | 23659 | 85.71 | 40.00 | 45.71 | 0.00 | 0.00 |  |
| chr7:130,844,517-130,845,627 | q32.3 | CN Gain | 1110 | 83.33 | 37.78 | 45.56 | 0.00 | 0.00 | PODXL |
| chr7:136,473,685-136,485,324 | q33 | CN Gain | 11639 | 83.33 | 37.78 | 45.56 | 0.00 | 0.00 | LOC349160 |
| chr7:136,777,262-136,803,900 | q33 | CN Gain | 26638 | 83.33 | 37.78 | 45.56 | 0.00 | 0.00 | DGKI |
| chr7:75,679,756-75,783,498 | q11.23 | CN Gain | 103742 | 83.33 | 37.78 | 45.56 | 0.00 | 0.00 | SRRM3, HSPB1 |
| chr7:127,665,761-127,684,120 | q32.1 | CN Gain | 18359 | 80.95 | 35.56 | 45.40 | 0.00 | 0.00 | LEP |
| chr7:127,886,322-127,975,435 | q32.1 | CN Gain | 89113 | 80.95 | 35.56 | 45.40 | 0.00 | 0.00 | METTL2B |
| chr7:128,197,042-128,402,953 | q32.1 | CN Gain | 205911 | 80.95 | 35.56 | 45.40 | 0.00 | 0.00 | CALU, OPN1SW, CCDC136, FLNC, ATP6V1F, LOC100130705, KCP, IRF5, TNPO3 |
| chr7:129,636,191-129,658,814 | q32.2 | CN Gain | 22623 | 80.95 | 35.56 | 45.40 | 0.00 | 0.00 | C7orf45 |
| chr7:129,697,254-129,816,245 | q32.2 | CN Gain | 118991 | 80.95 | 35.56 | 45.40 | 0.00 | 0.00 | CPA2, CPA4, CPA5, CPA1 |
| chr7:129,820,710-130,123,505 | q32.2 - q32.3 | CN Gain | 302795 | 80.95 | 35.56 | 45.40 | 0.00 | 0.00 | CEP41, MESTIT1, MIR335, MEST, COPG2, TSGA13, KLF14 |
| chr7:132,328,807-132,513,831 | q32.3 - q33 | CN Gain | 185024 | 80.95 | 35.56 | 45.40 | 0.00 | 0.00 | CHCHD3 |
| chr7:132,558,439-132,583,865 | q33 | CN Gain | 25426 | 80.95 | 35.56 | 45.40 | 0.00 | 0.00 |  |
| chr7:134,200,563-134,227,942 | q33 | CN Gain | 27379 | 80.95 | 35.56 | 45.40 | 0.00 | 0.00 | CALD1 |
| chr7:134,304,697-134,408,553 | q33 | CN Gain | 103856 | 80.95 | 35.56 | 45.40 | 0.00 | 0.00 | CALD1, AGBL3 |
| chr7:138,973,822-138,980,276 | q34 | CN Gain | 6454 | 80.95 | 35.56 | 45.40 | 0.00 | 0.00 | HIPK2 |
| chr7:150,363,570-150,383,596 | q36.1 | CN Gain | 20026 | 80.95 | 35.56 | 45.40 | 0.00 | 0.00 | ABCB8, ASIC3, CDK5 |
| chr7:150,899,920-150,906,772 | q36.1 | CN Gain | 6852 | 80.95 | 35.56 | 45.40 | 0.00 | 0.00 | PRKAG2 |
| chr7:73,265,885-73,296,210 | q11.23 | CN Gain | 30325 | 80.95 | 35.56 | 45.40 | 0.00 | 0.00 | LAT2, RFC2 |
| chr7:75,534,140-75,679,756 | q11.23 | CN Gain | 145616 | 80.95 | 35.56 | 45.40 | 0.00 | 0.00 | SRRM3 |
| chr7:82,400,356-82,421,434 | q21.11 | CN Gain | 21078 | 80.95 | 35.56 | 45.40 | 0.00 | 0.00 | PCLO |
| chr7:82,596,437-82,665,248 | q21.11 | CN Gain | 68811 | 80.95 | 35.56 | 45.40 | 0.00 | 0.00 | PCLO |
| chr7:98,736,712-98,841,656 | q22.1 | CN Gain | 104944 | 80.95 | 35.56 | 45.40 | 0.00 | 0.00 | ARPC1A, ARPC1B, PDAP1 |
| chr10:13,913,191-13,957,606 | p13 | CN Gain | 44415 | 2.38 | 37.78 | -35.40 | 0.00 | 0.00 | FRMD4A |
| chr10:55,969,174-56,276,742 | q21.1 | CN Gain | 307568 | 2.38 | 37.78 | -35.40 | 0.00 | 0.00 | PCDH15 |
| chr7:70,782,552-70,813,658 | q11.22 | CN Gain | 31106 | 57.14 | 13.33 | 43.81 | 0.00 | 0.00 | WBSCR17 |
| chr7:70,954,838-71,069,600 | q11.22 | CN Gain | 114762 | 57.14 | 13.33 | 43.81 | 0.00 | 0.00 | CALN1 |
| chr11:95,639,629-95,650,487 | q21 | CN Gain | 10858 | 7.14 | 46.67 | -39.52 | 0.00 | 0.00 | MAML2 |
| chr7:106,861,775-107,021,102 | q22.3 | CN Gain | 159327 | 78.57 | 33.33 | 45.24 | 0.00 | 0.00 | COG5, GPR22, DUS4L, BCAP29 |
| chr7:111,936,141-112,393,863 | q31.1 | CN Gain | 457722 | 78.57 | 33.33 | 45.24 | 0.00 | 0.00 | TMEM168, C7orf60 |
| chr7:133,006,687-133,069,810 | q33 | CN Gain | 63123 | 78.57 | 33.33 | 45.24 | 0.00 | 0.00 | EXOC4 |
| chr7:137,945,778-137,961,238 | q34 | CN Gain | 15460 | 78.57 | 33.33 | 45.24 | 0.00 | 0.00 | SVOPL |
| chr7:138,943,813-138,960,534 | q34 | CN Gain | 16721 | 78.57 | 33.33 | 45.24 | 0.00 | 0.00 | HIPK2 |
| chr7:14,908,153-14,918,106 | p21.2 | CN Gain | 9953 | 78.57 | 33.33 | 45.24 | 0.00 | 0.00 |  |
| chr7:140,364,044-140,374,660 | q34 | CN Gain | 10616 | 78.57 | 33.33 | 45.24 | 0.00 | 0.00 |  |
| chr7:153,731,212-153,742,234 | q36.2 | CN Gain | 11022 | 78.57 | 33.33 | 45.24 | 0.00 | 0.00 | DPP6 |
| chr7:153,808,274-153,849,788 | q36.2 | CN Gain | 41514 | 78.57 | 33.33 | 45.24 | 0.00 | 0.00 | DPP6 |
| chr7:8,100,204-8,160,361 | p21.3 | CN Gain | 60157 | 78.57 | 33.33 | 45.24 | 0.00 | 0.00 | ICA1 |
| chr8:114,450,244-114,606,424 | q23.3 | CN Gain | 156180 | 7.14 | 46.67 | -39.52 | 0.00 | 0.00 | CSMD3 |
| chr8:89,399,420-89,409,440 | q21.3 | CN Gain | 10020 | 7.14 | 46.67 | -39.52 | 0.00 | 0.00 | MMP16 |
| chr7:103,384,607-103,390,319 | q22.1 | CN Gain | 5712 | 64.29 | 20.00 | 44.29 | 0.00 | 0.00 | RELN |
| chr7:104,854,039-104,859,856 | q22.2 | CN Gain | 5817 | 76.19 | 31.11 | 45.08 | 0.00 | 0.00 |  |
| chr7:127,076,067-127,359,691 | q32.1 | CN Gain | 283624 | 76.19 | 31.11 | 45.08 | 0.00 | 0.00 | SND1 |
| chr7:13,218,344-13,433,896 | p21.3 - p21.2 | CN Gain | 215552 | 76.19 | 31.11 | 45.08 | 0.00 | 0.00 |  |
| chr7:13,628,203-13,650,153 | p21.2 | CN Gain | 21950 | 76.19 | 31.11 | 45.08 | 0.00 | 0.00 |  |
| chr7:13,670,085-13,757,177 | p21.2 | CN Gain | 87092 | 76.19 | 31.11 | 45.08 | 0.00 | 0.00 |  |
| chr7:133,816,395-133,835,132 | q33 | CN Gain | 18737 | 76.19 | 31.11 | 45.08 | 0.00 | 0.00 |  |
| chr7:134,164,973-134,170,512 | q33 | CN Gain | 5539 | 76.19 | 31.11 | 45.08 | 0.00 | 0.00 | CALD1 |
| chr7:135,742,765-135,804,466 | q33 | CN Gain | 61701 | 76.19 | 31.11 | 45.08 | 0.00 | 0.00 |  |
| chr7:138,803,994-138,904,866 | q34 | CN Gain | 100872 | 76.19 | 31.11 | 45.08 | 0.00 | 0.00 | KLRG2, CLEC2L, HIPK2 |
| chr7:139,110,439-139,163,036 | q34 | CN Gain | 52597 | 76.19 | 31.11 | 45.08 | 0.00 | 0.00 | HIPK2, TBXAS1 |
| chr7:139,806,629-139,900,303 | q34 | CN Gain | 93674 | 76.19 | 31.11 | 45.08 | 0.00 | 0.00 | MKRN1, DENND2A |
| chr7:14,977,053-15,263,573 | p21.2 - p21.1 | CN Gain | 286520 | 76.19 | 31.11 | 45.08 | 0.00 | 0.00 | AGMO |
| chr7:140,280,063-140,286,272 | q34 | CN Gain | 6209 | 76.19 | 31.11 | 45.08 | 0.00 | 0.00 |  |
| chr7:140,806,231-141,103,720 | q34 | CN Gain | 297489 | 76.19 | 31.11 | 45.08 | 0.00 | 0.00 | TMEM178B, AGK, KIAA1147, WEE2, FLJ40852, SSBP1 |
| chr7:151,152,187-151,167,892 | q36.1 | CN Gain | 15705 | 76.19 | 31.11 | 45.08 | 0.00 | 0.00 | PRKAG2 |
| chr7:154,064,256-154,125,121 | q36.2 | CN Gain | 60865 | 76.19 | 31.11 | 45.08 | 0.00 | 0.00 | DPP6 |
| chr7:78,766,522-78,825,298 | q21.11 | CN Gain | 58776 | 76.19 | 31.11 | 45.08 | 0.00 | 0.00 | MAGI2 |
| chr7:8,218,756-8,694,332 | p21.3 | CN Gain | 475576 | 76.19 | 31.11 | 45.08 | 0.00 | 0.00 | ICA1, NXPH1 |
| chr7:86,879,360-86,897,940 | q21.12 | CN Gain | 18580 | 76.19 | 31.11 | 45.08 | 0.00 | 0.00 | ABCB4 |
| chr7:86,971,077-86,971,354 | q21.12 | CN Gain | 277 | 76.19 | 31.11 | 45.08 | 0.00 | 0.00 | ABCB1 |
| chr7:87,016,825-87,019,952 | q21.12 | CN Gain | 3127 | 76.19 | 31.11 | 45.08 | 0.00 | 0.00 | ABCB1 |
| chr7:87,039,933-87,052,865 | q21.12 | CN Gain | 12932 | 76.19 | 31.11 | 45.08 | 0.00 | 0.00 | ABCB1 |
| chr7:9,597,808-9,640,411 | p21.3 | CN Gain | 42603 | 76.19 | 31.11 | 45.08 | 0.00 | 0.00 |  |
| chr7:10,982,709-11,063,439 | p21.3 | CN Gain | 80730 | 71.43 | 26.67 | 44.76 | 0.00 | 0.00 | PHF14 |
| chr7:100,500,124-100,571,228 | q22.1 | CN Gain | 71104 | 69.05 | 24.44 | 44.60 | 0.00 | 0.00 | TRIM56, SERPINE1 |
| chr7:101,336,367-101,355,347 | q22.1 | CN Gain | 18980 | 66.67 | 22.22 | 44.44 | 0.00 | 0.00 | CUX1 |
| chr7:102,515,628-102,676,234 | q22.1 | CN Gain | 160606 | 69.05 | 24.44 | 44.60 | 0.00 | 0.00 | ARMC10, NAPEPLD, RPL19P12, DPY19L2P2 |
| chr7:103,090,204-103,132,496 | q22.1 | CN Gain | 42292 | 66.67 | 22.22 | 44.44 | 0.00 | 0.00 | RELN |
| chr7:103,557,892-103,571,100 | q22.1 | CN Gain | 13208 | 71.43 | 26.67 | 44.76 | 0.00 | 0.00 | ORC5 |
| chr7:103,686,567-103,695,072 | q22.1 | CN Gain | 8505 | 73.81 | 28.89 | 44.92 | 0.00 | 0.00 |  |
| chr7:103,750,190-103,754,586 | q22.1 | CN Gain | 4396 | 71.43 | 26.67 | 44.76 | 0.00 | 0.00 |  |
| chr7:104,859,856-104,894,417 | q22.2 | CN Gain | 34561 | 73.81 | 28.89 | 44.92 | 0.00 | 0.00 | PUS7 |
| chr7:106,550,268-106,568,113 | q22.3 | CN Gain | 17845 | 71.43 | 26.67 | 44.76 | 0.00 | 0.00 | PRKAR2B |
| chr7:107,038,994-107,044,923 | q22.3 | CN Gain | 5929 | 73.81 | 28.89 | 44.92 | 0.00 | 0.00 | BCAP29 |
| chr7:107,177,320-107,185,501 | q22.3 | CN Gain | 8181 | 69.05 | 24.44 | 44.60 | 0.00 | 0.00 | CBLL1 |
| chr7:107,269,108-107,282,605 | q31.1 | CN Gain | 13497 | 66.67 | 22.22 | 44.44 | 0.00 | 0.00 |  |
| chr7:109,890,488-109,917,946 | q31.1 | CN Gain | 27458 | 73.81 | 28.89 | 44.92 | 0.00 | 0.00 |  |
| chr7:11,161,875-11,211,524 | p21.3 | CN Gain | 49649 | 73.81 | 28.89 | 44.92 | 0.00 | 0.00 | PHF14 |
| chr7:115,678,816-115,690,895 | q31.2 | CN Gain | 12079 | 73.81 | 28.89 | 44.92 | 0.00 | 0.00 | TES |
| chr7:12,198,433-12,532,900 | p21.3 | CN Gain | 334467 | 71.43 | 26.67 | 44.76 | 0.00 | 0.00 | TMEM106B, VWDE |
| chr7:126,509,315-126,550,310 | q31.33 | CN Gain | 40995 | 71.43 | 26.67 | 44.76 | 0.00 | 0.00 | GRM8 |
| chr7:126,577,210-126,661,477 | q31.33 | CN Gain | 84267 | 71.43 | 26.67 | 44.76 | 0.00 | 0.00 | GRM8 |
| chr7:133,336,029-133,583,574 | q33 | CN Gain | 247545 | 73.81 | 28.89 | 44.92 | 0.00 | 0.00 | EXOC4, LRGUK |
| chr7:133,615,617-133,663,077 | q33 | CN Gain | 47460 | 73.81 | 28.89 | 44.92 | 0.00 | 0.00 | SLC35B4 |
| chr7:137,961,238-138,019,541 | q34 | CN Gain | 58303 | 73.81 | 28.89 | 44.92 | 0.00 | 0.00 | SVOPL |
| chr7:138,071,653-138,171,269 | q34 | CN Gain | 99616 | 73.81 | 28.89 | 44.92 | 0.00 | 0.00 | ATP6V0A4, TMEM213, KIAA1549 |
| chr7:141,125,010-141,241,184 | q34 | CN Gain | 116174 | 73.81 | 28.89 | 44.92 | 0.00 | 0.00 | TAS2R4, TAS2R5, PRSS37 |
| chr7:142,271,158-142,287,704 | q34 | CN Gain | 16546 | 73.81 | 28.89 | 44.92 | 0.00 | 0.00 | EPHB6, TRPV6 |
| chr7:142,287,704-142,293,504 | q34 | CN Gain | 5800 | 71.43 | 26.67 | 44.76 | 0.00 | 0.00 | TRPV6 |
| chr7:142,332,641-142,344,636 | q34 | CN Gain | 11995 | 69.05 | 24.44 | 44.60 | 0.00 | 0.00 | TRPV5 |
| chr7:142,367,272-142,478,924 | q34 | CN Gain | 111652 | 69.05 | 24.44 | 44.60 | 0.00 | 0.00 | KEL, OR9A2, OR6V1, OR6W1P |
| chr7:142,610,029-142,729,350 | q34 | CN Gain | 119321 | 71.43 | 26.67 | 44.76 | 0.00 | 0.00 | TAS2R40, GSTK1, TMEM139, CASP2, CLCN1 |
| chr7:142,811,982-142,922,006 | q35 | CN Gain | 110024 | 71.43 | 26.67 | 44.76 | 0.00 | 0.00 | EPHA1, TAS2R60, EPHA1-AS1, TAS2R41 |
| chr7:147,023,317-147,131,428 | q35 | CN Gain | 108111 | 71.43 | 26.67 | 44.76 | 0.00 | 0.00 | CNTNAP2 |
| chr7:147,181,731-147,189,020 | q35 | CN Gain | 7289 | 73.81 | 28.89 | 44.92 | 0.00 | 0.00 | CNTNAP2 |
| chr7:147,766,184-147,889,037 | q36.1 | CN Gain | 122853 | 73.81 | 28.89 | 44.92 | 0.00 | 0.00 |  |
| chr7:147,963,025-148,080,289 | q36.1 | CN Gain | 117264 | 71.43 | 26.67 | 44.76 | 0.00 | 0.00 | CUL1 |
| chr7:148,303,811-148,396,034 | q36.1 | CN Gain | 92223 | 69.05 | 24.44 | 44.60 | 0.00 | 0.00 | PDIA4 |
| chr7:148,535,604-148,684,981 | q36.1 | CN Gain | 149377 | 71.43 | 26.67 | 44.76 | 0.00 | 0.00 | ZNF282, ZNF212, ZNF783, LOC155060 |
| chr7:148,688,124-148,691,325 | q36.1 | CN Gain | 3201 | 73.81 | 28.89 | 44.92 | 0.00 | 0.00 |  |
| chr7:149,218,345-149,378,035 | q36.1 | CN Gain | 159690 | 73.81 | 28.89 | 44.92 | 0.00 | 0.00 |  |
| chr7:149,407,978-149,773,216 | q36.1 | CN Gain | 365238 | 71.43 | 26.67 | 44.76 | 0.00 | 0.00 | ACTR3C, LRRC61, C7orf29, RARRES2, RNU6-33, RNU6-34, REPIN1, ZNF775, LOC728743, LOC285972 |
| chr7:151,214,176-151,256,167 | q36.1 | CN Gain | 41991 | 73.81 | 28.89 | 44.92 | 0.00 | 0.00 |  |
| chr7:151,263,015-151,270,817 | q36.1 | CN Gain | 7802 | 71.43 | 26.67 | 44.76 | 0.00 | 0.00 |  |
| chr7:151,433,103-151,489,380 | q36.1 | CN Gain | 56277 | 71.43 | 26.67 | 44.76 | 0.00 | 0.00 | GALNT11, MLL3 |
| chr7:151,899,796-151,962,889 | q36.1 | CN Gain | 63093 | 71.43 | 26.67 | 44.76 | 0.00 | 0.00 |  |
| chr7:152,234,508-152,564,494 | q36.2 | CN Gain | 329986 | 69.05 | 24.44 | 44.60 | 0.00 | 0.00 |  |
| chr7:153,692,954-153,696,503 | q36.2 | CN Gain | 3549 | 73.81 | 28.89 | 44.92 | 0.00 | 0.00 | DPP6 |
| chr7:154,134,196-154,138,790 | q36.2 | CN Gain | 4594 | 73.81 | 28.89 | 44.92 | 0.00 | 0.00 | DPP6 |
| chr7:155,619,534-155,672,135 | q36.3 | CN Gain | 52601 | 69.05 | 24.44 | 44.60 | 0.00 | 0.00 |  |
| chr7:155,718,090-156,049,105 | q36.3 | CN Gain | 331015 | 69.05 | 24.44 | 44.60 | 0.00 | 0.00 | LOC285889, LINC00244 |
| chr7:156,167,006-156,246,461 | q36.3 | CN Gain | 79455 | 71.43 | 26.67 | 44.76 | 0.00 | 0.00 | LMBR1 |
| chr7:156,407,649-156,503,804 | q36.3 | CN Gain | 96155 | 71.43 | 26.67 | 44.76 | 0.00 | 0.00 | NOM1, MNX1, LOC645249 |
| chr7:19,518,565-19,590,300 | p15.3 | CN Gain | 71735 | 73.81 | 28.89 | 44.92 | 0.00 | 0.00 |  |
| chr7:7,470,264-7,482,373 | p21.3 | CN Gain | 12109 | 69.05 | 24.44 | 44.60 | 0.00 | 0.00 | COL28A1 |
| chr7:7,482,373-7,527,731 | p21.3 | CN Gain | 45358 | 71.43 | 26.67 | 44.76 | 0.00 | 0.00 | COL28A1 |
| chr7:7,554,528-7,607,718 | p21.3 | CN Gain | 53190 | 73.81 | 28.89 | 44.92 | 0.00 | 0.00 | MIOS |
| chr7:77,722,079-77,826,162 | q21.11 | CN Gain | 104083 | 73.81 | 28.89 | 44.92 | 0.00 | 0.00 | RPL13AP17, MAGI2 |
| chr7:78,537,354-78,758,313 | q21.11 | CN Gain | 220959 | 73.81 | 28.89 | 44.92 | 0.00 | 0.00 | MAGI2 |
| chr7:85,434,840-85,439,310 | q21.11 | CN Gain | 4470 | 69.05 | 24.44 | 44.60 | 0.00 | 0.00 |  |
| chr7:86,186,454-86,186,990 | q21.11 | CN Gain | 536 | 73.81 | 28.89 | 44.92 | 0.00 | 0.00 | GRM3 |
| chr7:88,163,180-88,340,749 | q21.13 | CN Gain | 177569 | 73.81 | 28.89 | 44.92 | 0.00 | 0.00 | C7orf62, ZNF804B |
| chr7:88,395,501-88,499,892 | q21.13 | CN Gain | 104391 | 73.81 | 28.89 | 44.92 | 0.00 | 0.00 | ZNF804B |
| chr7:89,184,336-89,223,538 | q21.13 | CN Gain | 39202 | 69.05 | 24.44 | 44.60 | 0.00 | 0.00 |  |
| chr7:89,691,066-89,699,797 | q21.13 | CN Gain | 8731 | 71.43 | 26.67 | 44.76 | 0.00 | 0.00 | STEAP2 |
| chr7:9,143,479-9,202,760 | p21.3 | CN Gain | 59281 | 73.81 | 28.89 | 44.92 | 0.00 | 0.00 |  |
| chr7:9,826,506-9,838,969 | p21.3 | CN Gain | 12463 | 73.81 | 28.89 | 44.92 | 0.00 | 0.00 |  |
| chr7:90,550,913-90,592,344 | q21.13 | CN Gain | 41431 | 71.43 | 26.67 | 44.76 | 0.00 | 0.00 | CDK14 |
| chr7:90,629,235-90,669,833 | q21.13 | CN Gain | 40598 | 73.81 | 28.89 | 44.92 | 0.00 | 0.00 | CDK14 |
| chr7:90,672,550-90,722,181 | q21.13 | CN Gain | 49631 | 73.81 | 28.89 | 44.92 | 0.00 | 0.00 | CDK14 |
| chr7:92,498,375-92,626,086 | q21.2 - q21.3 | CN Gain | 127711 | 73.81 | 28.89 | 44.92 | 0.00 | 0.00 | SAMD9, SAMD9L |
| chr7:92,679,897-92,753,222 | q21.3 | CN Gain | 73325 | 73.81 | 28.89 | 44.92 | 0.00 | 0.00 | HEPACAM2, CCDC132 |
| chr7:93,003,123-93,234,315 | q21.3 | CN Gain | 231192 | 71.43 | 26.67 | 44.76 | 0.00 | 0.00 | CALCR, MIR4652 |
| chr7:93,704,640-93,720,351 | q21.3 | CN Gain | 15711 | 71.43 | 26.67 | 44.76 | 0.00 | 0.00 |  |
| chr7:93,852,743-93,879,635 | q21.3 | CN Gain | 26892 | 73.81 | 28.89 | 44.92 | 0.00 | 0.00 | COL1A2 |
| chr7:95,118,963-95,161,927 | q21.3 | CN Gain | 42964 | 71.43 | 26.67 | 44.76 | 0.00 | 0.00 |  |
| chr7:95,265,303-95,280,936 | q21.3 | CN Gain | 15633 | 71.43 | 26.67 | 44.76 | 0.00 | 0.00 | DYNC1I1 |
| chr7:97,264,175-97,264,483 | q21.3 | CN Gain | 308 | 66.67 | 22.22 | 44.44 | 0.00 | 0.00 |  |
| chr7:98,483,179-98,540,063 | q22.1 | CN Gain | 56884 | 73.81 | 28.89 | 44.92 | 0.00 | 0.00 | SMURF1 |
| chr10:11,162,431-11,280,583 | p14 | CN Gain | 118152 | 0.00 | 31.11 | -31.11 | 0.00 | 0.00 | CELF2-AS2, CELF2 |
| chr10:22,878,333-22,881,004 | p12.2 | CN Gain | 2671 | 0.00 | 31.11 | -31.11 | 0.00 | 0.00 | PIP4K2A |
| chr10:22,910,533-22,916,076 | p12.2 | CN Gain | 5543 | 0.00 | 31.11 | -31.11 | 0.00 | 0.00 | PIP4K2A |
| chr10:22,957,483-23,242,994 | p12.2 | CN Gain | 285511 | 0.00 | 31.11 | -31.11 | 0.00 | 0.00 | PIP4K2A |
| chr10:27,211,850-27,245,731 | p12.1 | CN Gain | 33881 | 0.00 | 31.11 | -31.11 | 0.00 | 0.00 |  |
| chr10:32,197,012-32,262,576 | p11.22 | CN Gain | 65564 | 0.00 | 31.11 | -31.11 | 0.00 | 0.00 | ARHGAP12 |
| chr10:55,513,510-55,561,596 | q21.1 | CN Gain | 48086 | 0.00 | 31.11 | -31.11 | 0.00 | 0.00 | PCDH15 |
| chr10:60,500,529-60,786,467 | q21.1 | CN Gain | 285938 | 0.00 | 31.11 | -31.11 | 0.00 | 0.00 | PHYHIPL, FAM13C |
[truncated: 394,146 more chars]
