# Supplementary material for: DNA copy number analysis of Grade II–III and Grade IV gliomas reveals differences in molecular ontogeny including chromothripsis associated with IDH mutation status
Source: Acta Neuropathol Commun. 2015 Jun 20;3:34. doi: 10.1186/s40478-015-0213-3 (PMC4474351; doi:10.1186/s40478-015-0213-3)
Supplement: Additional file 3: Table S3. — Loci with copy number alterations that are significantly different between low and high grade IDH wt gliomas with FDR <0.25. [file 40478_2015_213_MOESM3_ESM.docx]

| Region | Cytoband Location | Event | Region Length | Freq. in <4_wt> (%) | Freq. in <2_3_wt> (%) | Difference | p-value | q-bound | Gene Symbols |
| --- | --- | --- | --- | --- | --- | --- | --- | --- | --- |
| chr12:2,764,195-2,783,862 | p13.33 | CN Gain | 19667 | 0 | 47.06 | -47.06 | 0.00 | 0.13 | FKBP4 |
| chr12:56,461,933-56,468,659 | q14.1 | CN Gain | 6726 | 8 | 64.71 | -56.71 | 0.00 | 0.13 | METTL21B, TSFM |
| chr12:56,468,659-56,487,437 | q14.1 | CN Gain | 18778 | 4 | 58.82 | -54.82 | 0.00 | 0.13 | TSFM, AVIL |
| chr12:107,961,659-108,036,561 | q24.11 | CN Gain | 74902 | 0 | 47.06 | -47.06 | 0.00 | 0.13 | USP30-AS1, USP30, ALKBH2, UNG |
| chr12:123,815,302-123,863,636 | q24.31 | CN Gain | 48334 | 0 | 47.06 | -47.06 | 0.00 | 0.13 | SCARB1 |
| chr12:131,774,436-131,959,716 | q24.33 | CN Gain | 185280 | 0 | 47.06 | -47.06 | 0.00 | 0.13 | PXMP2, PGAM5, ANKLE2, GOLGA3, CHFR |
| chr12:132,010,325-132,349,534 | q24.33 | CN Gain | 339209 | 0 | 47.06 | -47.06 | 0.00 | 0.13 | ZNF605, ZNF26, ZNF84, ZNF140, ZNF10, ZNF268 |
| chr17:7,529,928-7,546,495 | p13.1 | CN Gain | 16567 | 0 | 47.06 | -47.06 | 0.00 | 0.13 | TP53, WRAP53 |
| chr19:49,830,869-49,837,053 | q13.31 | CN Gain | 6184 | 4 | 58.82 | -54.82 | 0.00 | 0.13 | IGSF23 |
| chr19:49,890,921-49,912,454 | q13.31 | CN Gain | 21533 | 8 | 64.71 | -56.71 | 0.00 | 0.13 | CEACAM16 |
| chr19:49,925,599-49,964,056 | q13.31 | CN Gain | 38457 | 12 | 70.59 | -58.59 | 0.00 | 0.13 | BCL3 |
| chr19:51,512,085-51,518,269 | q13.32 | CN Gain | 6184 | 0 | 47.06 | -47.06 | 0.00 | 0.13 | HIF3A |
| chr19:54,617,076-54,674,035 | q13.33 | CN Gain | 56959 | 8 | 64.71 | -56.71 | 0.00 | 0.13 | PTH2, LOC100507003, SLC17A7, PIH1D1, ALDH16A1, FLT3LG |
| chr11:62,027,651-62,059,870 | q12.3 | CN Gain | 32219 | 0 | 41.18 | -41.18 | 0.00 | 0.15 | AHNAK |
| chr11:62,358,359-62,463,550 | q12.3 | CN Gain | 105191 | 0 | 41.18 | -41.18 | 0.00 | 0.15 | WDR74, SNORD22, SNORD31, SNORD30, SNORD29, SNHG1, SNORD28, SNORD27, SNORD26, SNORD25, SLC3A2, CHRM1 |
| chr11:66,461,021-66,887,792 | q13.1 | CN Gain | 426771 | 0 | 41.18 | -41.18 | 0.00 | 0.15 | PC, C11orf86, SYT12, RHOD, KDM2A, ADRBK1, ANKRD13D, SSH3, POLD4, LOC100130987 |
| chr12:2,750,733-2,764,195 | p13.33 | CN Gain | 13462 | 4 | 52.94 | -48.94 | 0.00 | 0.15 | LOC283440 |
| chr12:2,783,862-2,884,739 | p13.33 | CN Gain | 100877 | 0 | 41.18 | -41.18 | 0.00 | 0.15 | FKBP4, ITFG2, NRIP2, LOC100507424, FOXM1, RHNO1, TULP3 |
| chr12:3,168,280-3,191,502 | p13.32 | CN Gain | 23222 | 4 | 52.94 | -48.94 | 0.00 | 0.15 | TSPAN9 |
| chr12:3,223,211-3,322,716 | p13.32 | CN Gain | 99505 | 4 | 52.94 | -48.94 | 0.00 | 0.15 | TSPAN9 |
| chr12:51,106,751-51,302,668 | q13.13 | CN Gain | 195917 | 0 | 41.18 | -41.18 | 0.00 | 0.15 | KRT75, KRT6B, KRT6C, KRT6A, KRT5, KRT71, KRT74, KRT72, KRT73 |
| chr12:52,331,542-52,500,842 | q13.13 | CN Gain | 169300 | 0 | 41.18 | -41.18 | 0.00 | 0.15 | ATP5G2, CALCOCO1 |
| chr12:52,866,010-52,881,955 | q13.13 | CN Gain | 15945 | 0 | 41.18 | -41.18 | 0.00 | 0.15 | SMUG1 |
| chr12:55,290,967-55,321,255 | q13.3 | CN Gain | 30288 | 0 | 41.18 | -41.18 | 0.00 | 0.15 | BAZ2A, ATP5B |
| chr12:56,487,437-56,506,820 | q14.1 | CN Gain | 19383 | 4 | 52.94 | -48.94 | 0.00 | 0.15 | AVIL, MIR26A2, CTDSP2 |
| chr12:103,506,753-103,588,280 | q23.3 | CN Gain | 81527 | 0 | 41.18 | -41.18 | 0.00 | 0.15 | MIR3922, CHST11 |
| chr12:107,825,196-107,961,659 | q24.11 | CN Gain | 136463 | 0 | 41.18 | -41.18 | 0.00 | 0.15 | SVOP |
| chr12:108,036,561-108,091,824 | q24.11 | CN Gain | 55263 | 0 | 41.18 | -41.18 | 0.00 | 0.15 | ACACB |
| chr12:123,792,278-123,806,839 | q24.31 | CN Gain | 14561 | 4 | 52.94 | -48.94 | 0.00 | 0.15 |  |
| chr12:123,863,636-123,971,574 | q24.31 | CN Gain | 107938 | 0 | 41.18 | -41.18 | 0.00 | 0.15 | SCARB1, UBC, MIR5188 |
| chr12:131,959,716-132,010,325 | q24.33 | CN Gain | 50609 | 0 | 41.18 | -41.18 | 0.00 | 0.15 | CHFR, ZNF605 |
| chr17:7,546,495-7,561,724 | p13.1 | CN Gain | 15229 | 4 | 52.94 | -48.94 | 0.00 | 0.15 | WRAP53, EFNB3 |
| chr17:24,074,302-24,109,563 | q11.2 | CN Gain | 35261 | 0 | 41.18 | -41.18 | 0.00 | 0.15 | RPL23A, SNORD42A, SNORD4B, TLCD1, NEK8, TRAF4, FAM222B |
| chr17:24,259,108-24,364,036 | q11.2 | CN Gain | 104928 | 0 | 41.18 | -41.18 | 0.00 | 0.15 | PHF12, SEZ6 |
| chr19:49,912,454-49,925,599 | q13.31 | CN Gain | 13145 | 12 | 64.71 | -52.71 | 0.00 | 0.15 |  |
| chr19:49,964,056-50,162,149 | q13.31 - q13.32 | CN Gain | 198093 | 12 | 64.71 | -52.71 | 0.00 | 0.15 | CBLC, BCAM, PVRL2, TOMM40, APOE, APOC1, APOC1P1, APOC4, APOC4-APOC2, APOC2, CLPTM1 |
| chr19:51,232,736-51,512,085 | q13.32 | CN Gain | 279349 | 0 | 41.18 | -41.18 | 0.00 | 0.15 | IGFL4, IGFL3, IGFL2, DKFZp434J0226, IGFL1, RNU6-66, HIF3A |
| chr19:53,317,720-53,344,929 | q13.32 | CN Gain | 27209 | 0 | 41.18 | -41.18 | 0.00 | 0.15 | LIG1 |
| chr19:51,601,357-51,654,090 | q13.32 | CN Gain | 52733 | 16 | 70.59 | -54.59 | 0.00 | 0.18 | CCDC8 |
| chr19:49,837,053-49,890,921 | q13.31 | CN Gain | 53868 | 8 | 58.82 | -50.82 | 0.00 | 0.20 | MIR4531, PVR, CEACAM19 |
| chr19:54,391,312-54,617,076 | q13.33 | CN Gain | 225764 | 8 | 58.82 | -50.82 | 0.00 | 0.20 | TRPM4, SLC6A16, MIR4324, CD37, TEAD2, DKKL1, CCDC155 |
| chr1:154,825,039-154,880,400 | q23.1 | CN Gain | 55361 | 16 | 64.71 | -48.71 | 0.00 | 0.22 | APOA1BP, GPATCH4, HAPLN2, BCAN |
| chr4:7,867,091-7,981,283 | p16.1 | CN Gain | 114192 | 0 | 35.29 | -35.29 | 0.00 | 0.22 | AFAP1 |
| chr5:172,049,834-172,065,353 | q35.1 | CN Gain | 15519 | 0 | 35.29 | -35.29 | 0.00 | 0.22 | NEURL1B |
| chr11:62,463,550-62,521,563 | q12.3 | CN Gain | 58013 | 0 | 35.29 | -35.29 | 0.00 | 0.22 | SLC22A6, SLC22A8 |
| chr11:63,349,189-63,419,660 | q13.1 | CN Gain | 70471 | 0 | 35.29 | -35.29 | 0.00 | 0.22 | C11orf84, MARK2 |
| chr11:63,898,601-64,314,523 | q13.1 | CN Gain | 415922 | 0 | 35.29 | -35.29 | 0.00 | 0.22 | LOC100996455, SLC22A11, SLC22A12, NRXN2, RASGRP2, PYGM, SF1, MAP4K2 |
| chr11:66,364,964-66,461,021 | q13.1 | CN Gain | 96057 | 0 | 35.29 | -35.29 | 0.00 | 0.22 | C11orf80, RCE1, LRFN4, PC |
| chr11:66,887,792-67,116,509 | q13.1 - q13.2 | CN Gain | 228717 | 0 | 35.29 | -35.29 | 0.00 | 0.22 | LOC100130987, CLCF1, RAD9A, PPP1CA, TBC1D10C, CARNS1, RPS6KB2, PTPRCAP, CORO1B, GPR152, CABP4, TMEM134, AIP, PITPNM1, CDK2AP2, CABP2, GSTP1 |
| chr11:68,781,115-68,989,467 | q13.2 | CN Gain | 208352 | 0 | 35.29 | -35.29 | 0.00 | 0.22 | MYEOV |
| chr12:2,581,177-2,750,733 | p13.33 | CN Gain | 169556 | 4 | 47.06 | -43.06 | 0.00 | 0.22 | CACNA1C, CACNA1C-AS1, LOC283440 |
| chr12:2,884,739-2,987,189 | p13.33 | CN Gain | 102450 | 0 | 35.29 | -35.29 | 0.00 | 0.22 | TULP3, TEAD4 |
| chr12:3,161,381-3,168,280 | p13.32 | CN Gain | 6899 | 4 | 47.06 | -43.06 | 0.00 | 0.22 | TSPAN9 |
| chr12:46,407,862-46,441,665 | q13.11 | CN Gain | 33803 | 0 | 35.29 | -35.29 | 0.00 | 0.22 | RAPGEF3 |
| chr12:52,193,690-52,331,542 | q13.13 | CN Gain | 137852 | 0 | 35.29 | -35.29 | 0.00 | 0.22 | LOC100652999, ATF7 |
| chr12:52,500,842-52,866,010 | q13.13 | CN Gain | 365168 | 0 | 35.29 | -35.29 | 0.00 | 0.22 | HOXC-AS5, HOXC13, HOXC12, HOTAIR, HOXC11, HOXC10, MIR196A2, HOXC9, HOXC8, HOXC6, HOXC5, MIR615, HOXC4, LOC100240735, FLJ12825, LOC100240734, LOC400043, SMUG1 |
| chr12:52,881,955-53,045,844 | q13.13 | CN Gain | 163889 | 0 | 35.29 | -35.29 | 0.00 | 0.22 | MIR3198-2, CBX5, HNRNPA1P10, HNRNPA1, NFE2, MIR148B, COPZ1, GPR84 |
| chr12:55,122,412-55,290,967 | q13.2 - q13.3 | CN Gain | 168555 | 0 | 35.29 | -35.29 | 0.00 | 0.22 | TIMELESS, MIP, SPRYD4, GLS2, RBMS2, BAZ2A |
| chr12:55,447,016-55,506,862 | q13.3 | CN Gain | 59846 | 0 | 35.29 | -35.29 | 0.00 | 0.22 | HSD17B6 |
| chr12:55,600,818-55,605,724 | q13.3 | CN Gain | 4906 | 0 | 35.29 | -35.29 | 0.00 | 0.22 | SDR9C7 |
| chr12:56,430,990-56,461,933 | q14.1 | CN Gain | 30943 | 16 | 64.71 | -48.71 | 0.00 | 0.22 | CDK4, MARCH9, CYP27B1, METTL1, METTL21B |
| chr12:103,449,202-103,506,753 | q23.3 | CN Gain | 57551 | 0 | 35.29 | -35.29 | 0.00 | 0.22 | CHST11 |
| chr12:108,091,824-108,106,990 | q24.11 | CN Gain | 15166 | 0 | 35.29 | -35.29 | 0.00 | 0.22 | ACACB |
| chr12:119,337,431-119,394,152 | q24.31 | CN Gain | 56721 | 0 | 35.29 | -35.29 | 0.00 | 0.22 | COX6A1, TRIAP1, GATC, SRSF9, DYNLL1 |
| chr12:120,036,508-120,074,771 | q24.31 | CN Gain | 38263 | 0 | 35.29 | -35.29 | 0.00 | 0.22 | P2RX7 |
| chr12:123,706,066-123,792,278 | q24.31 | CN Gain | 86212 | 4 | 47.06 | -43.06 | 0.00 | 0.22 |  |
| chr12:123,806,839-123,815,302 | q24.31 | CN Gain | 8463 | 4 | 47.06 | -43.06 | 0.00 | 0.22 |  |
| chr12:123,971,574-123,999,326 | q24.31 | CN Gain | 27752 | 0 | 35.29 | -35.29 | 0.00 | 0.22 | DHX37 |
| chr12:131,763,405-131,774,436 | q24.33 | CN Gain | 11031 | 4 | 47.06 | -43.06 | 0.00 | 0.22 | POLE, PXMP2 |
| chr16:55,195,410-55,226,555 | q13 | CN Gain | 31145 | 0 | 35.29 | -35.29 | 0.00 | 0.22 | MT2A, MT1L, MT1E, MT1M |
| chr17:7,521,221-7,529,928 | p13.1 | CN Gain | 8707 | 4 | 47.06 | -43.06 | 0.00 | 0.22 | TP53 |
| chr17:23,975,618-24,004,294 | q11.2 | CN Gain | 28676 | 4 | 47.06 | -43.06 | 0.00 | 0.22 | KIAA0100, SDF2 |
| chr17:39,046,810-39,131,390 | q21.31 | CN Gain | 84580 | 0 | 35.29 | -35.29 | 0.00 | 0.22 | MEOX1 |
| chr17:41,354,498-41,384,605 | q21.31 | CN Gain | 30107 | 0 | 35.29 | -35.29 | 0.00 | 0.22 | MAPT |
| chr19:39,578,324-39,685,339 | q13.11 | CN Gain | 107015 | 16 | 64.71 | -48.71 | 0.00 | 0.22 | GPI, PDCD2L, UBA2, WTIP |
| chr19:48,742,537-48,744,846 | q13.31 | CN Gain | 2309 | 16 | 64.71 | -48.71 | 0.00 | 0.22 | XRCC1 |
| chr19:48,833,711-48,865,328 | q13.31 | CN Gain | 31617 | 16 | 64.71 | -48.71 | 0.00 | 0.22 | CADM4, PLAUR |
| chr19:49,818,903-49,830,869 | q13.31 | CN Gain | 11966 | 4 | 47.06 | -43.06 | 0.00 | 0.22 | IGSF23 |
| chr19:50,162,149-50,617,324 | q13.32 | CN Gain | 455175 | 16 | 64.71 | -48.71 | 0.00 | 0.22 | CLPTM1, RELB, CLASRP, ZNF296, GEMIN7, PPP1R37, NKPD1, TRAPPC6A, BLOC1S3, EXOC3L2, MARK4, CKM, KLC3, ERCC2, PPP1R13L, CD3EAP, ERCC1 |
| chr19:50,836,850-50,895,106 | q13.32 | CN Gain | 58256 | 12 | 58.82 | -46.82 | 0.00 | 0.22 | EML2, LOC100287177, MIR642B, MIR642A, GIPR, SNRPD2, QPCTL |
| chr19:50,895,106-51,058,457 | q13.32 | CN Gain | 163351 | 4 | 47.06 | -43.06 | 0.00 | 0.22 | QPCTL, FBXO46, LOC388553, SIX5, DMPK, DMWD, RSPH6A, SYMPK |
| chr19:51,518,269-51,529,300 | q13.32 | CN Gain | 11031 | 12 | 58.82 | -46.82 | 0.00 | 0.22 | HIF3A |
| chr19:51,539,782-51,587,851 | q13.32 | CN Gain | 48069 | 16 | 64.71 | -48.71 | 0.00 | 0.22 | PPP5C |
| chr19:51,587,851-51,601,357 | q13.32 | CN Gain | 13506 | 20 | 70.59 | -50.59 | 0.00 | 0.22 |  |
| chr19:51,654,090-51,736,229 | q13.32 | CN Gain | 82139 | 16 | 64.71 | -48.71 | 0.00 | 0.22 | PNMAL1, PNMAL2, PPP5D1 |
| chr19:51,894,830-52,050,432 | q13.32 | CN Gain | 155602 | 12 | 58.82 | -46.82 | 0.00 | 0.22 | PRKD2, MIR320E, STRN4, FKRP, SLC1A5, SNAR-E, AP2S1 |
| chr19:53,024,298-53,317,720 | q13.32 | CN Gain | 293422 | 0 | 35.29 | -35.29 | 0.00 | 0.22 | CRX, SULT2A1, SNAR-A12, SNAR-A13, SNAR-C1, SNAR-C2, SNAR-C5, SNAR-A1, SNAR-A2, SNAR-A3, SNAR-A4, SNAR-A5, SNAR-A6, SNAR-A7, SNAR-A8, SNAR-A9, SNAR-A10, SNAR-A11, SNAR-A14, SNAR-C1, SNAR-C2, SNAR-C5, SNAR-A1, SNAR-A2, SNAR-C4, SNAR-A12, SNAR-A13, SNAR-C3, SNAR-C1, SNAR-C2, SNAR-C5, BSPH1, ELSPBP1, CABP5, PLA2G4C, LIG1 |
| chr19:54,346,000-54,391,312 | q13.33 | CN Gain | 45312 | 12 | 58.82 | -46.82 | 0.00 | 0.22 | PPFIA3, HRC, TRPM4 |
| chr19:54,674,035-54,926,224 | q13.33 | CN Gain | 252189 | 16 | 64.71 | -48.71 | 0.00 | 0.22 | FLT3LG, RPL13AP5, RPL13A, SNORD32A, SNORD33, SNORD34, SNORD35A, SNORD35B, RPS11, MIR150, FCGRT, RCN3, NOSIP, PRRG2, PRR12, RRAS, SCAF1, IRF3, BCL2L12, MIR5088, PRMT1, ADM5, CPT1C |
| chr19:55,127,589-55,202,834 | q13.33 | CN Gain | 75245 | 4 | 47.06 | -43.06 | 0.00 | 0.22 | ATF5, MIR4751, SIGLEC11, SIGLEC16, VRK3 |
| chr19:56,733,528-56,812,735 | q13.33 | CN Gain | 79207 | 0 | 35.29 | -35.29 | 0.00 | 0.22 | ZNF175, FLJ30403, SIGLEC5 |
| chr19:56,927,216-56,948,370 | q13.33 | CN Gain | 21154 | 0 | 35.29 | -35.29 | 0.00 | 0.22 | FPR1 |
| chr12:3,191,502-3,223,211 | p13.32 | CN Gain | 31709 | 8 | 52.94 | -44.94 | 0.00 | 0.24 | TSPAN9 |
| chr19:53,616,675-53,620,715 | q13.32 | CN Gain | 4040 | 8 | 52.94 | -44.94 | 0.00 | 0.24 | GRIN2D |
| chr19:55,074,578-55,114,110 | q13.33 | CN Gain | 39532 | 8 | 52.94 | -44.94 | 0.00 | 0.24 | TBC1D17, MIR4750, IL4I1, NUP62 |
|  |  |  |  |  |  |  |  |  |  |
|  |  |  |  |  |  |  |  |  |  |
|  |  |  |  |  |  |  |  |  |  |
|  |  |  |  |  |  |  |  |  |  |
|  |  |  |  |  |  |  |  |  |  |
|  |  |  |  |  |  |  |  |  |  |
|  |  |  |  |  |  |  |  |  |  |
|  |  |  |  |  |  |  |  |  |  |
|  |  |  |  |  |  |  |  |  |  |
|  |  |  |  |  |  |  |  |  |  |
|  |  |  |  |  |  |  |  |  |  |
|  |  |  |  |  |  |  |  |  |  |
|  |  |  |  |  |  |  |  |  |  |
|  |  |  |  |  |  |  |  |  |  |
|  |  |  |  |  |  |  |  |  |  |
