# Supplementary material for: DNA copy number analysis of Grade II–III and Grade IV gliomas reveals differences in molecular ontogeny including chromothripsis associated with IDH mutation status
Source: Acta Neuropathol Commun. 2015 Jun 20;3:34. doi: 10.1186/s40478-015-0213-3 (PMC4474351; doi:10.1186/s40478-015-0213-3)
Supplement: Additional file 4: Table S4. — Loci with copy number alterations that are significantly different between the two clusters of IDH mut gliomas identified by hierarchical clustering with FDR <0.25. [file 40478_2015_213_MOESM4_ESM.doc]

| **Region** | **Cytoband Location** | **Event** | **Region Length** | **Freq. in <m3cl3> (%)** | **Freq. in <m3cl1> (%)** | **Difference** | **p-value** | **q-bound** | **Gene Symbols** |
| --- | --- | --- | --- | --- | --- | --- | --- | --- | --- |
| **chr10:131,188,181-131,199,146** | q26.3 | CN Loss | 10965 | 9.09 | 80.65 | -71.55 | 0.00 | 0.07 | MGMT |
| **chr10:131,208,988-131,319,598** | q26.3 | CN Loss | 110610 | 9.09 | 80.65 | -71.55 | 0.00 | 0.07 | MGMT |
| **chr10:130,051,271-130,999,895** | q26.2 - q26.3 | CN Loss | 948624 | 9.09 | 77.42 | -68.33 | 0.00 | 0.07 |  |
| **chr10:131,183,816-131,188,181** | q26.3 | CN Loss | 4365 | 9.09 | 77.42 | -68.33 | 0.00 | 0.07 | MGMT |
| **chr10:131,199,146-131,208,988** | q26.3 | CN Loss | 9842 | 9.09 | 77.42 | -68.33 | 0.00 | 0.07 | MGMT |
| **chr10:131,319,598-131,794,751** | q26.3 | CN Loss | 475153 | 9.09 | 77.42 | -68.33 | 0.00 | 0.07 | MGMT, MIR4297, EBF3, LOC387723, CTAGE7P |
| **chr10:132,349,445-133,227,178** | q26.3 | CN Loss | 877733 | 9.09 | 77.42 | -68.33 | 0.00 | 0.07 | MIR378C, TCERG1L |
| **chr10:125,448,376-125,545,765** | q26.13 | CN Loss | 97389 | 9.09 | 74.19 | -65.10 | 0.00 | 0.08 | CPXM2 |
| **chr10:129,999,793-130,051,271** | q26.2 | CN Loss | 51478 | 9.09 | 74.19 | -65.10 | 0.00 | 0.08 |  |
| **chr10:130,999,895-131,183,816** | q26.3 | CN Loss | 183921 | 9.09 | 74.19 | -65.10 | 0.00 | 0.08 | MGMT |
| **chr10:133,227,178-133,251,532** | q26.3 | CN Loss | 24354 | 9.09 | 74.19 | -65.10 | 0.00 | 0.08 |  |
| **chr10:134,861,030-135,142,974** | q26.3 | CN Loss | 281944 | 9.09 | 74.19 | -65.10 | 0.00 | 0.08 | KNDC1, UTF1, VENTX, MIR202, ADAM8, TUBGCP2, ZNF511, CALY, PRAP1, FUOM, ECHS1, MIR3944, PAOX, MTG1, SPRN, LOC619207 |
| **chr11:5,920,467-6,073,690** | p15.4 | CN Loss | 153223 | 0.00 | 64.52 | -64.52 | 0.00 | 0.08 | OR56A3, OR56A5, OR52L1, OR56A4, OR56A1 |
| **chr11:6,073,690-6,316,962** | p15.4 | CN Loss | 243272 | 0.00 | 61.29 | -61.29 | 0.00 | 0.08 | OR56B4, OR52B2, OR52W1, C11orf42, FAM160A2, CNGA4, CCKBR, PRKCDBP |
| **chr11:6,506,620-6,544,833** | p15.4 | CN Loss | 38213 | 0.00 | 61.29 | -61.29 | 0.00 | 0.08 | DNHD1 |
| **chr11:6,680,229-8,137,397** | p15.4 | CN Loss | 1457168 | 0.00 | 61.29 | -61.29 | 0.00 | 0.08 | GVINP1, OR2AG2, OR2AG1, OR6A2, OR10A5, OR10A2, OR10A4, OR2D2, OR2D3, ZNF215, ZNF214, NLRP14, RBMXL2, SYT9, OLFML1, PPFIBP2, CYB5R2, OVCH2, OR5P2, OR5P3, OR5E1P, LOC283299, OR10A6, OR10A3, NLRP10, EIF3F, TUB, RIC3 |
| **chr11:10,907,408-11,757,094** | p15.3 | CN Loss | 849686 | 0.00 | 61.29 | -61.29 | 0.00 | 0.08 | CSNK2A1P, GALNTL4, MIR4299 |
| **chr11:12,017,060-12,616,888** | p15.3 - p15.2 | CN Loss | 599828 | 0.00 | 61.29 | -61.29 | 0.00 | 0.08 | MICAL2, MICALCL, PARVA |
| **chr19:51,980,237-52,110,891** | q13.32 | CN Loss | 130654 | 63.64 | 6.45 | 57.18 | 0.00 | 0.08 | SLC1A5, SNAR-E, AP2S1 |
| **chr10:131,901,494-131,958,065** | q26.3 | CN Loss | 56571 | 18.18 | 80.65 | -62.46 | 0.00 | 0.09 |  |
| **chr10:125,227,844-125,448,376** | q26.13 | CN Loss | 220532 | 9.09 | 70.97 | -61.88 | 0.00 | 0.10 | GPR26 |
| **chr10:125,545,765-125,574,980** | q26.13 | CN Loss | 29215 | 9.09 | 70.97 | -61.88 | 0.00 | 0.10 | CPXM2 |
| **chr10:129,790,475-129,999,793** | q26.2 | CN Loss | 209318 | 9.09 | 70.97 | -61.88 | 0.00 | 0.10 | MKI67 |
| **chr10:134,454,942-134,861,030** | q26.3 | CN Loss | 406088 | 9.09 | 70.97 | -61.88 | 0.00 | 0.10 | TTC40, LOC399829, GPR123, KNDC1 |
| **chr10:135,142,974-135,374,737** | q26.3 | CN Loss | 231763 | 9.09 | 70.97 | -61.88 | 0.00 | 0.10 | CYP2E1, SYCE1, SPRNP1, FRG2B, DUX4, DUX2, DUX4L2, DUX4L3, DUX4L5, DUX4L6, DUX4L7, DUX2, DUX4L |
| **chr10:131,794,751-131,901,494** | q26.3 | CN Loss | 106743 | 18.18 | 77.42 | -59.24 | 0.00 | 0.10 | LOC387723, CTAGE7P, GLRX3 |
| **chr10:131,958,065-132,349,445** | q26.3 | CN Loss | 391380 | 18.18 | 77.42 | -59.24 | 0.00 | 0.10 |  |
| **chr10:118,927,309-119,015,879** | q25.3 | CN Loss | 88570 | 9.09 | 67.74 | -58.65 | 0.00 | 0.10 | KCNK18, SLC18A2 |
| **chr10:125,137,437-125,227,844** | q26.13 | CN Loss | 90407 | 9.09 | 67.74 | -58.65 | 0.00 | 0.10 |  |
| **chr10:125,574,980-125,677,736** | q26.13 | CN Loss | 102756 | 9.09 | 67.74 | -58.65 | 0.00 | 0.10 | CPXM2 |
| **chr10:126,878,407-127,340,062** | q26.13 | CN Loss | 461655 | 9.09 | 67.74 | -58.65 | 0.00 | 0.10 | LOC100169752, TEX36 |
| **chr10:133,251,532-133,681,936** | q26.3 | CN Loss | 430404 | 9.09 | 67.74 | -58.65 | 0.00 | 0.10 | FLJ46300, PPP2R2D, BNIP3 |
| **chr11:2,089,547-2,550,210** | p15.5 | CN Loss | 460663 | 9.09 | 67.74 | -58.65 | 0.00 | 0.10 | MIR483, IGF2, IGF2-AS, INS-IGF2, INS, TH, MIR4686, ASCL2, C11orf21, TSPAN32, CD81, TSSC4, TRPM5, KCNQ1 |
| **chr11:6,316,962-6,506,620** | p15.4 | CN Loss | 189658 | 0.00 | 58.06 | -58.06 | 0.00 | 0.10 | SMPD1, APBB1, HPX, TRIM3, ARFIP2, TIMM10B, DNHD1 |
| **chr11:6,544,833-6,680,229** | p15.4 | CN Loss | 135396 | 0.00 | 58.06 | -58.06 | 0.00 | 0.10 | DNHD1, RRP8, ILK, TAF10, TPP1, DCHS1, MRPL17 |
| **chr11:8,137,397-8,227,848** | p15.4 | CN Loss | 90451 | 0.00 | 58.06 | -58.06 | 0.00 | 0.10 | RIC3, LMO1 |
| **chr11:9,309,338-10,907,408** | p15.4 - p15.3 | CN Loss | 1598070 | 0.00 | 58.06 | -58.06 | 0.00 | 0.10 | IPO7, SNORA23, LOC644656, ZNF143, WEE1, SWAP70, LOC440028, SBF2-AS1, SBF2, ADM, AMPD3, MIR4485, MTRNR2L8, RNF141, LYVE1, MRVI1-AS1, MRVI1, CTR9, SNORD97, EIF4G2, ZBED5, LOC729013 |
| **chr11:11,757,094-12,017,060** | p15.3 | CN Loss | 259966 | 0.00 | 58.06 | -58.06 | 0.00 | 0.10 | USP47, DKK3 |
| **chr11:12,616,888-13,177,393** | p15.2 | CN Loss | 560505 | 0.00 | 58.06 | -58.06 | 0.00 | 0.10 | TEAD1, LOC100506305, RASSF10 |
| **chr11:13,344,664-13,603,453** | p15.2 | CN Loss | 258789 | 0.00 | 58.06 | -58.06 | 0.00 | 0.10 | ARNTL, BTBD10, PTH |
| **chr11:8,227,848-9,309,338** | p15.4 | CN Loss | 1081490 | 0.00 | 54.84 | -54.84 | 0.00 | 0.10 | LMO1, STK33, TRIM66, SNORA3, SNORA45, RPL27A, ST5, AKIP1, C11orf16, ASCL3, TMEM9B, TMEM9B-AS1, NRIP3, SCUBE2, MIR5691, KRT8P41, DENND5A, TMEM41B |
| **chr11:13,177,393-13,344,664** | p15.2 | CN Loss | 167271 | 0.00 | 54.84 | -54.84 | 0.00 | 0.10 | ARNTL |
| **chr11:13,603,453-13,851,198** | p15.2 | CN Loss | 247745 | 0.00 | 54.84 | -54.84 | 0.00 | 0.10 | FAR1 |
| **chr11:17,342,597-18,294,801** | p15.1 | CN Loss | 952204 | 0.00 | 54.84 | -54.84 | 0.00 | 0.10 | NCR3LG1, KCNJ11, ABCC8, USH1C, MYOD1, KCNC1, SERGEF, TPH1, SAAL1, SAA3P, MRGPRX3, MRGPRX4, LOC494141, SAA4, SAA2-SAA4, SAA2, SAA1, HPS5 |
| **chr19:51,754,973-51,809,907** | q13.32 | CN Loss | 54934 | 54.55 | 3.23 | 51.32 | 0.00 | 0.10 | PPP5D1, CALM3 |
| **chr19:52,110,891-52,597,147** | q13.32 | CN Loss | 486256 | 63.64 | 9.68 | 53.96 | 0.00 | 0.10 | ARHGAP35, NPAS1, TMEM160, ZC3H4, SAE1, BBC3, MIR3190, MIR3191, CCDC9, PRR24, C5AR1, GPR77, DHX34 |
| **chr19:53,351,823-53,381,372** | q13.32 | CN Loss | 29549 | 63.64 | 9.68 | 53.96 | 0.00 | 0.10 | LIG1 |
| **chr10:118,896,262-118,927,309** | q25.3 | CN Loss | 31047 | 9.09 | 64.52 | -55.43 | 0.00 | 0.14 | MIR3663 |
| **chr10:125,062,918-125,137,437** | q26.13 | CN Loss | 74519 | 9.09 | 64.52 | -55.43 | 0.00 | 0.14 |  |
| **chr10:125,677,736-126,032,578** | q26.13 | CN Loss | 354842 | 9.09 | 64.52 | -55.43 | 0.00 | 0.14 | CHST15 |
| **chr10:126,841,730-126,878,407** | q26.13 | CN Loss | 36677 | 9.09 | 64.52 | -55.43 | 0.00 | 0.14 |  |
| **chr10:127,340,062-127,605,604** | q26.13 - q26.2 | CN Loss | 265542 | 9.09 | 64.52 | -55.43 | 0.00 | 0.14 | TEX36, LOC283038, FLJ37035, C10orf137, MMP21, UROS, MIR4484, BCCIP, DHX32, FANK1 |
| **chr10:129,129,363-129,208,544** | q26.2 | CN Loss | 79181 | 9.09 | 64.52 | -55.43 | 0.00 | 0.14 | DOCK1 |
| **chr10:129,788,561-129,790,475** | q26.2 | CN Loss | 1914 | 9.09 | 64.52 | -55.43 | 0.00 | 0.14 | MKI67 |
| **chr10:133,681,936-134,454,942** | q26.3 | CN Loss | 773006 | 9.09 | 64.52 | -55.43 | 0.00 | 0.14 | JAKMIP3, DPYSL4, STK32C, LRRC27, PWWP2B, C10orf91, INPP5A, NKX6-2 |
| **chr11:1,825,949-2,089,547** | p15.5 | CN Loss | 263598 | 9.09 | 64.52 | -55.43 | 0.00 | 0.14 | MIR4298, LSP1, TNNT3, MRPL23, MRPL23-AS1, H19, MIR675 |
| **chr11:2,550,210-3,373,323** | p15.5 - p15.4 | CN Loss | 823113 | 9.09 | 64.52 | -55.43 | 0.00 | 0.14 | KCNQ1, KCNQ1OT1, KCNQ1DN, CDKN1C, SLC22A18AS, SLC22A18, PHLDA2, SNORA54, NAP1L4, CARS, OSBPL5, MRGPRG, MRGPRG-AS1, MRGPRE, ZNF195, OR7E12P, LOC650368 |
| **chr11:4,123,479-4,781,681** | p15.4 | CN Loss | 658202 | 9.09 | 64.52 | -55.43 | 0.00 | 0.14 | LOC100506082, OR52B4, TRIM21, OR52K2, OR52K1, OR52M1, C11orf40, OR52I2, OR52I1, TRIM68, OR51D1, OR51E1, OR51E2, OR51F1, OR52R1 |
| **chr11:4,907,587-5,192,562** | p15.4 | CN Loss | 284975 | 9.09 | 64.52 | -55.43 | 0.00 | 0.14 | OR51A4, OR51A2, MMP26, OR51L1, OR52J3, OR52E2, OR52A5, OR52A1, OR51V1 |
| **chr11:5,279,480-5,495,980** | p15.4 | CN Loss | 216500 | 9.09 | 64.52 | -55.43 | 0.00 | 0.14 | OR51B4, OR51B2, OR51B6, OR51M1, OR51Q1, OR51B5, OR51I1, OR51I2, OR52D1, UBQLN3, UBQLNL |
| **chr11:5,815,233-5,920,467** | p15.4 | CN Loss | 105234 | 9.09 | 64.52 | -55.43 | 0.00 | 0.14 | OR52E6, OR52E8, OR52E4 |
| **chr10:117,991,926-118,526,776** | q25.3 | CN Loss | 534850 | 9.09 | 61.29 | -52.20 | 0.00 | 0.14 | GFRA1, CCDC172, PNLIPRP3, PNLIP, PNLIPRP1, PNLIPRP2, C10orf82, HSPA12A |
| **chr10:118,758,661-118,896,262** | q25.3 | CN Loss | 137601 | 9.09 | 61.29 | -52.20 | 0.00 | 0.14 | KIAA1598, VAX1 |
| **chr10:120,500,697-120,678,806** | q26.11 | CN Loss | 178109 | 9.09 | 61.29 | -52.20 | 0.00 | 0.14 | CACUL1 |
| **chr10:121,675,950-121,830,909** | q26.11 - q26.12 | CN Loss | 154959 | 9.09 | 61.29 | -52.20 | 0.00 | 0.14 | SEC23IP, MIR4682 |
| **chr10:122,135,966-122,236,785** | q26.12 | CN Loss | 100819 | 9.09 | 61.29 | -52.20 | 0.00 | 0.14 | PPAPDC1A |
| **chr10:123,626,944-123,896,105** | q26.13 | CN Loss | 269161 | 9.09 | 61.29 | -52.20 | 0.00 | 0.14 | ATE1, NSMCE4A, TACC2 |
| **chr10:124,967,257-125,062,918** | q26.13 | CN Loss | 95661 | 9.09 | 61.29 | -52.20 | 0.00 | 0.14 |  |
| **chr10:126,032,578-126,040,107** | q26.13 | CN Loss | 7529 | 9.09 | 61.29 | -52.20 | 0.00 | 0.14 |  |
| **chr10:127,605,604-127,711,662** | q26.2 | CN Loss | 106058 | 9.09 | 61.29 | -52.20 | 0.00 | 0.14 | FANK1, ADAM12 |
| **chr10:127,721,128-127,764,392** | q26.2 | CN Loss | 43264 | 9.09 | 61.29 | -52.20 | 0.00 | 0.14 | ADAM12 |
| **chr10:128,105,772-128,567,061** | q26.2 | CN Loss | 461289 | 9.09 | 61.29 | -52.20 | 0.00 | 0.14 | C10orf90 |
| **chr10:128,692,103-129,129,363** | q26.2 | CN Loss | 437260 | 9.09 | 61.29 | -52.20 | 0.00 | 0.14 | FAM196A, DOCK1 |
| **chr10:129,208,544-129,240,032** | q26.2 | CN Loss | 31488 | 9.09 | 61.29 | -52.20 | 0.00 | 0.14 | NPS |
| **chr10:129,504,531-129,532,139** | q26.2 | CN Loss | 27608 | 9.09 | 61.29 | -52.20 | 0.00 | 0.14 |  |
| **chr11:1,021,541-1,825,949** | p15.5 | CN Loss | 804408 | 9.09 | 61.29 | -52.20 | 0.00 | 0.14 | MUC6, MUC2, MUC5B, TOLLIP, LOC255512, BRSK2, DUSP8, KRTAP5-1, LOC338651, KRTAP5-2, KRTAP5-3, MOB2, KRTAP5-4, KRTAP5-5, FAM99A, FAM99B, KRTAP5-6, IFITM10, CTSD, SYT8, TNNI2 |
| **chr11:3,373,323-4,123,479** | p15.4 | CN Loss | 750156 | 9.09 | 61.29 | -52.20 | 0.00 | 0.14 | LOC650368, TRPC2, ART5, ART1, CHRNA10, NUP98, PGAP2, RHOG, MIR4687, STIM1, RRM1 |
| **chr11:4,781,681-4,907,587** | p15.4 | CN Loss | 125906 | 9.09 | 61.29 | -52.20 | 0.00 | 0.14 | OR52R1, OR51F2, OR51S1, OR51T1, OR51A7, OR51G2, OR51G1 |
| **chr11:5,192,562-5,279,480** | p15.4 | CN Loss | 86918 | 9.09 | 61.29 | -52.20 | 0.00 | 0.14 | HBB, HBD, HBBP1, HBG1, HBG2, HBE1, OR51B4 |
| **chr11:5,495,980-5,815,233** | p15.4 | CN Loss | 319253 | 9.09 | 61.29 | -52.20 | 0.00 | 0.14 | OR52H1, OR52B6, TRIM6, TRIM6-TRIM34, TRIM34, TRIM5, TRIM22, OR56B1, OR52N4, OR52N5, OR52N1, OR52N2 |
| **chr10:96,966,771-97,034,592** | q23.33 | CN Loss | 67821 | 0.00 | 51.61 | -51.61 | 0.00 | 0.14 | C10orf129, PDLIM1 |
| **chr10:100,232,107-100,391,829** | q24.2 | CN Loss | 159722 | 0.00 | 51.61 | -51.61 | 0.00 | 0.14 | HPSE2 |
| **chr10:102,301,616-102,328,704** | q24.31 | CN Loss | 27088 | 0.00 | 51.61 | -51.61 | 0.00 | 0.14 | HIF1AN |
| **chr10:112,114,718-112,517,423** | q25.2 | CN Loss | 402705 | 0.00 | 51.61 | -51.61 | 0.00 | 0.14 | DUSP5, SMC3, RBM20 |
| **chr11:13,851,198-14,386,323** | p15.2 | CN Loss | 535125 | 0.00 | 51.61 | -51.61 | 0.00 | 0.14 | SPON1, RRAS2 |
| **chr11:17,325,284-17,342,597** | p15.1 | CN Loss | 17313 | 0.00 | 51.61 | -51.61 | 0.00 | 0.14 | NCR3LG1 |
| **chr11:18,294,801-19,060,077** | p15.1 | CN Loss | 765276 | 0.00 | 51.61 | -51.61 | 0.00 | 0.14 | HPS5, GTF2H1, LDHA, LDHC, LDHAL6A, TSG101, UEVLD, SPTY2D1-AS1, SPTY2D1, TMEM86A, IGSF22, PTPN5, MRGPRX1, MRGPRX2 |
| **chr11:20,505,590-21,139,131** | p15.1 | CN Loss | 633541 | 0.00 | 51.61 | -51.61 | 0.00 | 0.14 | SLC6A5, NELL1 |
| **chr10:96,459,916-96,966,771** | q23.33 | CN Loss | 506855 | 0.00 | 48.39 | -48.39 | 0.00 | 0.14 | CYP2C18, CYP2C19, CYP2C9, CYP2C8, C10orf129 |
| **chr10:97,034,592-97,052,963** | q23.33 | CN Loss | 18371 | 0.00 | 48.39 | -48.39 | 0.00 | 0.14 | PDLIM1 |
| **chr10:100,165,321-100,232,107** | q24.2 | CN Loss | 66786 | 0.00 | 48.39 | -48.39 | 0.00 | 0.14 | MIR4685, HPS1, HPSE2 |
| **chr10:100,391,829-100,458,408** | q24.2 | CN Loss | 66579 | 0.00 | 48.39 | -48.39 | 0.00 | 0.14 | HPSE2 |
| **chr10:101,537,734-101,598,648** | q24.2 | CN Loss | 60914 | 0.00 | 48.39 | -48.39 | 0.00 | 0.14 | ABCC2 |
| **chr10:102,091,098-102,301,616** | q24.31 | CN Loss | 210518 | 0.00 | 48.39 | -48.39 | 0.00 | 0.14 | SCD, LINC00263, WNT8B, SEC31B, NDUFB8, HIF1AN |
| **chr10:102,328,704-102,371,986** | q24.31 | CN Loss | 43282 | 0.00 | 48.39 | -48.39 | 0.00 | 0.14 |  |
| **chr10:105,254,885-105,352,021** | q24.33 | CN Loss | 97136 | 0.00 | 48.39 | -48.39 | 0.00 | 0.14 | NEURL, SH3PXD2A |
| **chr10:109,749,422-110,495,199** | q25.1 | CN Loss | 745777 | 0.00 | 48.39 | -48.39 | 0.00 | 0.14 |  |
| **chr10:110,562,791-111,593,017** | q25.1 | CN Loss | 1030226 | 0.00 | 48.39 | -48.39 | 0.00 | 0.14 | RNU6-53 |
| **chr10:112,074,327-112,114,718** | q25.2 | CN Loss | 40391 | 0.00 | 48.39 | -48.39 | 0.00 | 0.14 |  |
| **chr10:112,517,423-112,531,964** | q25.2 | CN Loss | 14541 | 0.00 | 48.39 | -48.39 | 0.00 | 0.14 | RBM20 |
| **chr10:112,771,127-112,956,768** | q25.2 | CN Loss | 185641 | 0.00 | 48.39 | -48.39 | 0.00 | 0.14 | ADRA2A |
| **chr11:14,386,323-14,588,046** | p15.2 | CN Loss | 201723 | 0.00 | 48.39 | -48.39 | 0.00 | 0.14 | COPB1, PSMA1 |
| **chr11:14,879,817-15,232,705** | p15.2 | CN Loss | 352888 | 0.00 | 48.39 | -48.39 | 0.00 | 0.14 | CALCA, CALCB, INSC |
| **chr11:15,655,575-15,891,204** | p15.2 | CN Loss | 235629 | 0.00 | 48.39 | -48.39 | 0.00 | 0.14 |  |
| **chr11:16,473,284-17,325,284** | p15.1 | CN Loss | 852000 | 0.00 | 48.39 | -48.39 | 0.00 | 0.14 | C11orf58, PLEKHA7, OR7E14P, RPS13, PIK3C2A, NUCB2 |
| **chr11:19,060,077-19,307,987** | p15.1 | CN Loss | 247910 | 0.00 | 48.39 | -48.39 | 0.00 | 0.14 | ZDHHC13, CSRP3, E2F8 |
| **chr11:20,089,325-20,505,590** | p15.1 | CN Loss | 416265 | 0.00 | 48.39 | -48.39 | 0.00 | 0.14 | NAV2, DBX1, HTATIP2, PRMT3 |
| **chr11:21,139,131-21,543,635** | p15.1 | CN Loss | 404504 | 0.00 | 48.39 | -48.39 | 0.00 | 0.14 | NELL1 |
| **chr11:22,601,782-24,984,663** | p14.3 | CN Loss | 2382881 | 0.00 | 48.39 | -48.39 | 0.00 | 0.14 | FANCF, GAS2, SVIP, CCDC179, LUZP2 |
| **chr11:27,696,427-28,004,181** | p14.1 | CN Loss | 307754 | 0.00 | 48.39 | -48.39 | 0.00 | 0.14 | BDNF, KIF18A |
| **chr11:28,203,806-29,167,179** | p14.1 | CN Loss | 963373 | 0.00 | 48.39 | -48.39 | 0.00 | 0.14 | METTL15 |
| **chr11:31,869,107-32,478,193** | p13 | CN Loss | 609086 | 0.00 | 48.39 | -48.39 | 0.00 | 0.14 | RCN1, WT1, WT1-AS |
| **chr11:36,630,870-36,978,924** | p12 | CN Loss | 348054 | 0.00 | 48.39 | -48.39 | 0.00 | 0.14 | C11orf74 |
| **chr19:51,518,269-51,754,973** | q13.32 | CN Loss | 236704 | 54.55 | 6.45 | 48.09 | 0.00 | 0.14 | HIF3A, PPP5C, CCDC8, PNMAL1, PNMAL2, PPP5D1 |
| **chr19:51,809,907-51,980,237** | q13.32 | CN Loss | 170330 | 54.55 | 6.45 | 48.09 | 0.00 | 0.14 | PTGIR, GNG8, DACT3, DACT3-AS1, PRKD2, MIR320E, STRN4, FKRP, SLC1A5 |
| **chr19:52,597,147-52,948,738** | q13.32 | CN Loss | 351591 | 63.64 | 12.90 | 50.73 | 0.00 | 0.14 | MEIS3, SLC8A2, KPTN, NAPA-AS1, NAPA, ZNF541, GLTSCR1, EHD2, GLTSCR2 |
| **chr19:53,345,429-53,351,823** | q13.32 | CN Loss | 6394 | 63.64 | 12.90 | 50.73 | 0.00 | 0.14 | LIG1 |
| **chr19:53,381,372-53,467,557** | q13.32 | CN Loss | 86185 | 63.64 | 12.90 | 50.73 | 0.00 | 0.14 | CARD8, LOC100505812, ZNF114 |
| **chr19:56,964,412-57,629,007** | q13.33 - q13.41 | CN Loss | 664595 | 63.64 | 12.90 | 50.73 | 0.00 | 0.14 | FPR2, FPR3, ZNF577, ZNF649, ZNF613, ZNF350, ZNF615, ZNF614, ZNF432, ZNF841, ZNF616, ZNF836, PPP2R1A, ZNF766, MIR643, ZNF480, ZNF610, ZNF880, ZNF528, ZNF534 |
| **chr10:117,703,536-117,991,926** | q25.3 | CN Loss | 288390 | 9.09 | 58.06 | -48.97 | 0.01 | 0.16 | GFRA1 |
| **chr10:118,526,776-118,758,661** | q25.3 | CN Loss | 231885 | 9.09 | 58.06 | -48.97 | 0.01 | 0.16 | ENO4, KIAA1598 |
| **chr10:120,009,563-120,500,697** | q26.11 | CN Loss | 491134 | 9.09 | 58.06 | -48.97 | 0.01 | 0.16 | FAM204A, PRLHR, CACUL1 |
| **chr10:120,678,806-120,765,353** | q26.11 | CN Loss | 86547 | 9.09 | 58.06 | -48.97 | 0.01 | 0.16 |  |
| **chr10:121,103,299-121,675,950** | q26.11 | CN Loss | 572651 | 9.09 | 58.06 | -48.97 | 0.01 | 0.16 | GRK5, MIR4681, RGS10, TIAL1, BAG3, INPP5F, MCMBP, SEC23IP |
| **chr10:121,830,909-122,135,966** | q26.12 | CN Loss | 305057 | 9.09 | 58.06 | -48.97 | 0.01 | 0.16 |  |
| **chr10:122,236,785-122,384,657** | q26.12 | CN Loss | 147872 | 9.09 | 58.06 | -48.97 | 0.01 | 0.16 | PPAPDC1A, MIR5694 |
| **chr10:123,356,844-123,626,944** | q26.13 | CN Loss | 270100 | 9.09 | 58.06 | -48.97 | 0.01 | 0.16 | ATE1 |
| **chr10:123,896,105-124,090,650** | q26.13 | CN Loss | 194545 | 9.09 | 58.06 | -48.97 | 0.01 | 0.16 | TACC2, BTBD16 |
| **chr10:124,819,559-124,967,257** | q26.13 | CN Loss | 147698 | 9.09 | 58.06 | -48.97 | 0.01 | 0.16 | HMX3, HMX2, BUB3 |
| **chr10:126,040,107-126,081,112** | q26.13 | CN Loss | 41005 | 9.09 | 58.06 | -48.97 | 0.01 | 0.16 | OAT |
| **chr10:126,811,709-126,841,730** | q26.13 | CN Loss | 30021 | 9.09 | 58.06 | -48.97 | 0.01 | 0.16 | CTBP2 |
| **chr10:127,711,662-127,721,128** | q26.2 | CN Loss | 9466 | 9.09 | 58.06 | -48.97 | 0.01 | 0.16 | ADAM12 |
| **chr10:127,764,392-128,105,772** | q26.2 | CN Loss | 341380 | 9.09 | 58.06 | -48.97 | 0.01 | 0.16 | ADAM12, LINC00601, C10orf90 |
| **chr10:128,567,061-128,692,103** | q26.2 | CN Loss | 125042 | 9.09 | 58.06 | -48.97 | 0.01 | 0.16 | DOCK1 |
| **chr10:129,532,139-129,553,629** | q26.2 | CN Loss | 21490 | 9.09 | 58.06 | -48.97 | 0.01 | 0.16 |  |
| **chr10:129,777,017-129,788,561** | q26.2 | CN Loss | 11544 | 9.09 | 58.06 | -48.97 | 0.01 | 0.16 | MKI67 |
| **chr11:848,511-1,021,541** | p15.5 | CN Loss | 173030 | 9.09 | 58.06 | -48.97 | 0.01 | 0.16 | TSPAN4, CHID1, AP2A2, MUC6 |
| **chr19:48,765,386-48,849,820** | q13.31 | CN Loss | 84434 | 54.55 | 9.68 | 44.87 | 0.01 | 0.16 | XRCC1, PINLYP, IRGQ, ZNF576, SRRM5, ZNF428, CADM4, PLAUR |
| **chr19:49,567,826-49,690,757** | q13.31 | CN Loss | 122931 | 54.55 | 9.68 | 44.87 | 0.01 | 0.16 | ZNF285, ZNF229, ZNF180 |
| **chr19:49,925,599-50,297,066** | q13.31 - q13.32 | CN Loss | 371467 | 54.55 | 9.68 | 44.87 | 0.01 | 0.16 | BCL3, CBLC, BCAM, PVRL2, TOMM40, APOE, APOC1, APOC1P1, APOC4, APOC4-APOC2, APOC2, CLPTM1, RELB, CLASRP, ZNF296, GEMIN7, PPP1R37 |
| **chr19:51,289,262-51,518,269** | q13.32 | CN Loss | 229007 | 54.55 | 9.68 | 44.87 | 0.01 | 0.16 | IGFL3, IGFL2, DKFZp434J0226, IGFL1, RNU6-66, HIF3A |
| **chr19:52,948,738-53,345,429** | q13.32 | CN Loss | 396691 | 63.64 | 16.13 | 47.51 | 0.01 | 0.16 | GLTSCR2, SNORD23, SEPW1, TPRX1, CRX, SULT2A1, SNAR-A12, SNAR-A13, SNAR-C1, SNAR-C2, SNAR-C5, SNAR-A1, SNAR-A2, SNAR-A3, SNAR-A4, SNAR-A5, SNAR-A6, SNAR-A7, SNAR-A8, SNAR-A9, SNAR-A10, SNAR-A11, SNAR-A14, SNAR-C1, SNAR-C2, SNAR-C5, SNAR-A1, SNAR-A2, SNAR-C4, SNAR-A12, SNAR-A13, SNAR-C3, SNAR-C1, SNAR-C2, SNAR-C5, BSPH1, ELSPBP1, CABP5, PLA2G4C, LIG1 |
| **chr19:53,467,557-53,847,358** | q13.32 - q13.33 | CN Loss | 379801 | 63.64 | 16.13 | 47.51 | 0.01 | 0.16 | ZNF114, CCDC114, EMP3, TMEM143, SYNGR4, KDELR1, GRIN2D, GRWD1, KCNJ14, CYTH2, LMTK3, SULT2B1, FAM83E, SPACA4, RPL18, SPHK2, DBP, CA11, SEC1P |
| **chr19:56,837,831-56,964,412** | q13.33 | CN Loss | 126581 | 63.64 | 16.13 | 47.51 | 0.01 | 0.16 | SIGLEC14, MIR99B, MIRLET7E, MIR125A, LINC00085, HAS1, FPR1, FPR2 |
| **chr19:57,629,007-58,207,937** | q13.41 | CN Loss | 578930 | 63.64 | 16.13 | 47.51 | 0.01 | 0.16 | ZNF534, ZNF578, ZNF808, ZNF701, ZNF137P, ZNF83, ZNF611, ZNF600, ZNF28, ZNF468, ZNF320, ZNF321P, ZNF816-ZNF321P, ZNF816, ZNF702P |
| **chr19:58,345,699-58,750,948** | q13.41 | CN Loss | 405249 | 63.64 | 16.13 | 47.51 | 0.01 | 0.16 | ZNF347, ZNF665, ZNF818P, ZNF677, VN1R2, VN1R4, FAM90A27P, BIRC8, ZNF845, ZNF525, ZNF765, TPM3P9, ZNF761, ZNF813, ZNF331 |
| **chr19:62,572,244-63,036,091** | q13.43 | CN Loss | 463847 | 63.64 | 16.13 | 47.51 | 0.01 | 0.16 | ZNF547, ZNF548, ZNF17, ZNF749, VN1R1, ZNF772, ZNF419, ZNF773, ZNF549, ZNF550, ZNF416, ZIK1, ZNF530, ZNF134, ZNF211, ZSCAN4, ZNF551, ZNF154, ZNF671, ZNF776, ZNF586, ZNF552, FKBP1AP1, ZNF587B |
| **chr10:89,719,312-90,826,079** | q23.31 | CN Loss | 1106767 | 0.00 | 45.16 | -45.16 | 0.01 | 0.17 | RNLS, LIPJ, LIPF, LIPK, LIPN, LIPM, ANKRD22, STAMBPL1, ACTA2, FAS-AS1, FAS, MIR4679-2, MIR4679-1 |
| **chr10:91,151,848-96,459,916** | q23.31 - q23.33 | CN Loss | 5308068 | 0.00 | 45.16 | -45.16 | 0.01 | 0.17 | IFIT1, IFIT5, SLC16A12, MIR107, PANK1, FLJ37201, KIF20B, LOC643529, HTR7, RPP30, ANKRD1, LINC00502, NUDT9P1, PCGF5, LOC100188947, HECTD2, PPP1R3C, TNKS2, FGFBP3, BTAF1, CPEB3, MARCH5, MARK2P9, IDE, KIF11, HHEX, EXOC6, CYP26C1, CYP26A1, MYOF, CEP55, FFAR4, RBP4, PDE6C, FRA10AC1, LGI1, SLC35G1, PIPSL, PLCE1, LOC100128054, NOC3L, TBC1D12, HELLS, CYP2C18 |
| **chr10:97,052,963-97,456,334** | q23.33 | CN Loss | 403371 | 0.00 | 45.16 | -45.16 | 0.01 | 0.17 | SORBS1, ALDH18A1, TCTN3 |
| **chr10:99,941,849-100,165,321** | q24.2 | CN Loss | 223472 | 0.00 | 45.16 | -45.16 | 0.01 | 0.17 | R3HCC1L, LOXL4, MIR1287, PYROXD2 |
| **chr10:100,458,408-101,361,412** | q24.2 | CN Loss | 903004 | 0.00 | 45.16 | -45.16 | 0.01 | 0.17 | HPSE2, CNNM1, GOT1, NKX2-3, SLC25A28 |
| **chr10:101,500,437-101,537,734** | q24.2 | CN Loss | 37297 | 0.00 | 45.16 | -45.16 | 0.01 | 0.17 | CUTC, ABCC2 |
| **chr10:101,598,648-102,091,098** | q24.2 - q24.31 | CN Loss | 492450 | 0.00 | 45.16 | -45.16 | 0.01 | 0.17 | ABCC2, DNMBP, DNMBP-AS1, CPN1, ERLIN1, CHUK, SNORA12, CWF19L1, BLOC1S2, PKD2L1 |
| **chr10:102,371,986-103,216,580** | q24.31 - q24.32 | CN Loss | 844594 | 0.00 | 45.16 | -45.16 | 0.01 | 0.17 | PAX2, FAM178A, MIR608, SEMA4G, MRPL43, C10orf2, LZTS2, PDZD7, SFXN3, KAZALD1, TLX1NB, TLX1, LBX1, FLJ41350, BTRC |
| **chr10:105,226,176-105,254,885** | q24.33 | CN Loss | 28709 | 0.00 | 45.16 | -45.16 | 0.01 | 0.17 | CALHM3, NEURL |
| **chr10:105,352,021-105,622,209** | q24.33 | CN Loss | 270188 | 0.00 | 45.16 | -45.16 | 0.01 | 0.17 | SH3PXD2A, LOC100505839 |
| **chr10:105,773,364-105,783,594** | q25.1 | CN Loss | 10230 | 0.00 | 45.16 | -45.16 | 0.01 | 0.17 | SLK, COL17A1 |
| **chr10:106,089,855-108,127,310** | q25.1 | CN Loss | 2037455 | 0.00 | 45.16 | -45.16 | 0.01 | 0.17 | CCDC147, SORCS3 |
| **chr10:110,495,199-110,562,791** | q25.1 | CN Loss | 67592 | 0.00 | 45.16 | -45.16 | 0.01 | 0.17 |  |
| **chr10:111,593,017-111,672,784** | q25.1 | CN Loss | 79767 | 0.00 | 45.16 | -45.16 | 0.01 | 0.17 | XPNPEP1 |
| **chr10:111,867,071-112,074,327** | q25.2 | CN Loss | 207256 | 0.00 | 45.16 | -45.16 | 0.01 | 0.17 | ADD3, MXI1, SMNDC1 |
| **chr10:112,531,964-112,771,127** | q25.2 | CN Loss | 239163 | 0.00 | 45.16 | -45.16 | 0.01 | 0.17 | RBM20, LOC282997, PDCD4, MIR4680, BBIP1, RPL13AP6, SHOC2 |
| **chr11:14,588,046-14,879,817** | p15.2 | CN Loss | 291771 | 0.00 | 45.16 | -45.16 | 0.01 | 0.17 | PSMA1, PDE3B, CYP2R1 |
| **chr11:15,232,705-15,655,575** | p15.2 | CN Loss | 422870 | 0.00 | 45.16 | -45.16 | 0.01 | 0.17 |  |
| **chr11:15,891,204-16,010,451** | p15.2 | CN Loss | 119247 | 0.00 | 45.16 | -45.16 | 0.01 | 0.17 | SOX6 |
| **chr11:19,307,987-20,089,325** | p15.1 | CN Loss | 781338 | 0.00 | 45.16 | -45.16 | 0.01 | 0.17 | NAV2-AS5, NAV2-AS4, MIR4486, LOC100126784, NAV2, MIR4694 |
| **chr11:21,543,635-22,601,782** | p15.1 - p14.3 | CN Loss | 1058147 | 0.00 | 45.16 | -45.16 | 0.01 | 0.17 | NELL1, ANO5, SLC17A6, FANCF |
| **chr11:24,984,663-25,642,694** | p14.3 | CN Loss | 658031 | 0.00 | 45.16 | -45.16 | 0.01 | 0.17 | LUZP2 |
| **chr11:27,485,128-27,696,427** | p14.1 | CN Loss | 211299 | 0.00 | 45.16 | -45.16 | 0.01 | 0.17 | BDNF-AS, BDNF |
| **chr11:28,004,181-28,203,806** | p14.1 | CN Loss | 199625 | 0.00 | 45.16 | -45.16 | 0.01 | 0.17 | MIR610, KIF18A, METTL15 |
| **chr11:29,167,179-31,325,066** | p14.1 - p13 | CN Loss | 2157887 | 0.00 | 45.16 | -45.16 | 0.01 | 0.17 | KCNA4, FSHB, ARL14EP, MPPED2, DCDC5, DCDC1 |
| **chr11:31,748,669-31,869,107** | p13 | CN Loss | 120438 | 0.00 | 45.16 | -45.16 | 0.01 | 0.17 | ELP4, PAX6, DKFZp686K1684 |
| **chr11:32,478,193-32,881,163** | p13 | CN Loss | 402970 | 0.00 | 45.16 | -45.16 | 0.01 | 0.17 | EIF3M, CCDC73, PRRG4, QSER1 |
| **chr11:34,636,066-35,148,740** | p13 | CN Loss | 512674 | 0.00 | 45.16 | -45.16 | 0.01 | 0.17 | EHF, APIP, MIR1343, PDHX, CD44 |
| **chr11:36,486,099-36,630,870** | p12 | CN Loss | 144771 | 0.00 | 45.16 | -45.16 | 0.01 | 0.17 | TRAF6, RAG1, RAG2, C11orf74 |
| **chr11:36,978,924-39,297,444** | p12 | CN Loss | 2318520 | 0.00 | 45.16 | -45.16 | 0.01 | 0.17 |  |
| **chr11:44,242,113-44,456,124** | p11.2 | CN Loss | 214011 | 0.00 | 45.16 | -45.16 | 0.01 | 0.17 | ALX4 |
| **chr10:86,818,846-87,355,430** | q23.1 | CN Loss | 536584 | 0.00 | 41.94 | -41.94 | 0.01 | 0.19 | GRID1-AS1, GRID1 |
| **chr10:89,206,055-89,606,163** | q23.2 - q23.31 | CN Loss | 400108 | 0.00 | 41.94 | -41.94 | 0.01 | 0.19 | MIR4678, MINPP1, PAPSS2, ATAD1, CFL1P1 |
| **chr10:89,706,490-89,719,312** | q23.31 | CN Loss | 12822 | 0.00 | 41.94 | -41.94 | 0.01 | 0.19 | PTEN |
| **chr10:90,826,079-91,151,848** | q23.31 | CN Loss | 325769 | 0.00 | 41.94 | -41.94 | 0.01 | 0.19 | CH25H, LIPA, IFIT2, IFIT3, IFIT1B, IFIT1 |
| **chr10:97,456,334-98,796,665** | q23.33 - q24.1 | CN Loss | 1340331 | 0.00 | 41.94 | -41.94 | 0.01 | 0.19 | ENTPD1, LOC728558, C10orf131, CC2D2B, CCNJ, MIR3157, ZNF518A, BLNK, DNTT, OPALIN, TLL2, TM9SF3, PIK3AP1, LCOR, C10orf12, SLIT1 |
| **chr10:99,490,555-99,941,849** | q24.2 | CN Loss | 451294 | 0.00 | 41.94 | -41.94 | 0.01 | 0.19 | ZFYVE27, SFRP5, GOLGA7B, CRTAC1, R3HCC1L |
| **chr10:101,361,412-101,500,437** | q24.2 | CN Loss | 139025 | 0.00 | 41.94 | -41.94 | 0.01 | 0.19 | SLC25A28, ENTPD7, COX15, CUTC |
| **chr10:103,216,580-103,228,696** | q24.32 | CN Loss | 12116 | 0.00 | 41.94 | -41.94 | 0.01 | 0.19 | BTRC |
| **chr10:104,613,355-105,226,176** | q24.32 - q24.33 | CN Loss | 612821 | 0.00 | 41.94 | -41.94 | 0.01 | 0.19 | C10orf32, C10orf32-AS3MT, AS3MT, CNNM2, NT5C2, LOC729020, INA, PCGF6, TAF5, USMG5, MIR1307, PDCD11, CALHM2, CALHM1, CALHM3 |
| **chr10:105,622,209-105,773,364** | q24.33 - q25.1 | CN Loss | 151155 | 0.00 | 41.94 | -41.94 | 0.01 | 0.19 | OBFC1, SLK |
| **chr10:105,783,594-106,089,855** | q25.1 | CN Loss | 306261 | 0.00 | 41.94 | -41.94 | 0.01 | 0.19 | MIR936, COL17A1, SFR1, WDR96, MIR609, GSTO1, MIR4482-1, GSTO2, ITPRIP |
| **chr10:111,672,784-111,867,071** | q25.1 - q25.2 | CN Loss | 194287 | 0.00 | 41.94 | -41.94 | 0.01 | 0.19 | XPNPEP1, LOC100505933, ADD3 |
| **chr11:16,010,451-16,473,284** | p15.2 - p15.1 | CN Loss | 462833 | 0.00 | 41.94 | -41.94 | 0.01 | 0.19 | SOX6 |
| **chr11:25,642,694-27,485,128** | p14.3 - p14.1 | CN Loss | 1842434 | 0.00 | 41.94 | -41.94 | 0.01 | 0.19 | ANO3, MUC15, SLC5A12, FIBIN, BBOX1, CCDC34, LGR4, LIN7C, BDNF-AS |
| **chr11:31,325,066-31,748,669** | p13 | CN Loss | 423603 | 0.00 | 41.94 | -41.94 | 0.01 | 0.19 | DCDC1, DNAJC24, IMMP1L, ELP4 |
| **chr11:33,351,265-34,636,066** | p13 | CN Loss | 1284801 | 0.00 | 41.94 | -41.94 | 0.01 | 0.19 | KIAA1549L, C11orf91, CD59, FBXO3, LMO2, CAPRIN1, NAT10, ABTB2, CAT, ELF5, EHF |
| **chr11:35,148,740-35,173,493** | p13 | CN Loss | 24753 | 0.00 | 41.94 | -41.94 | 0.01 | 0.19 | CD44 |
| **chr11:35,211,122-36,486,099** | p13 - p12 | CN Loss | 1274977 | 0.00 | 41.94 | -41.94 | 0.01 | 0.19 | SLC1A2, PAMR1, FJX1, TRIM44, MIR3973, LDLRAD3, COMMD9, PRR5L, TRAF6 |
| **chr11:39,297,444-40,631,577** | p12 | CN Loss | 1334133 | 0.00 | 41.94 | -41.94 | 0.01 | 0.19 | LRRC4C |
| **chr11:42,604,765-43,421,651** | p12 - p11.2 | CN Loss | 816886 | 0.00 | 41.94 | -41.94 | 0.01 | 0.19 | HNRNPKP3, API5, TTC17 |
| **chr11:44,234,575-44,242,113** | p11.2 | CN Loss | 7538 | 0.00 | 41.94 | -41.94 | 0.01 | 0.19 | ALX4 |
| **chr11:44,456,124-44,901,319** | p11.2 | CN Loss | 445195 | 0.00 | 41.94 | -41.94 | 0.01 | 0.19 | CD82, TSPAN18 |
| **chr11:45,500,538-45,757,793** | p11.2 | CN Loss | 257255 | 0.00 | 41.94 | -41.94 | 0.01 | 0.19 | CHST1, LOC100507384, DKFZp779M0652 |
| **chr19:48,622,234-48,765,386** | q13.31 | CN Loss | 143152 | 54.55 | 12.90 | 41.64 | 0.01 | 0.22 | LYPD3, PHLDB3, ETHE1, ZNF575, XRCC1 |
| **chr19:48,849,820-49,024,269** | q13.31 | CN Loss | 174449 | 54.55 | 12.90 | 41.64 | 0.01 | 0.22 | PLAUR, IRGC, SMG9, KCNN4, LYPD5, ZNF283 |
| **chr19:49,690,757-49,925,599** | q13.31 | CN Loss | 234842 | 54.55 | 12.90 | 41.64 | 0.01 | 0.22 | ZNF180, CEACAM20, CEACAM22P, IGSF23, MIR4531, PVR, CEACAM19, CEACAM16 |
| **chr19:50,297,066-50,648,189** | q13.32 | CN Loss | 351123 | 54.55 | 12.90 | 41.64 | 0.01 | 0.22 | PPP1R37, NKPD1, TRAPPC6A, BLOC1S3, EXOC3L2, MARK4, CKM, KLC3, ERCC2, PPP1R13L, CD3EAP, ERCC1 |
| **chr10:119,015,879-119,164,210** | q25.3 - q26.11 | CN Loss | 148331 | 18.18 | 67.74 | -49.56 | 0.01 | 0.22 | SLC18A2, PDZD8 |
| **chr10:119,205,769-119,460,169** | q26.11 | CN Loss | 254400 | 18.18 | 67.74 | -49.56 | 0.01 | 0.22 | EMX2OS, EMX2 |
| **chr10:113,661,226-114,220,256** | q25.2 | CN Loss | 559030 | 9.09 | 54.84 | -45.75 | 0.01 | 0.23 | GPAM, TECTB, GUCY2GP, ACSL5, ZDHHC6, VTI1A |
| **chr10:117,609,199-117,703,536** | q25.3 | CN Loss | 94337 | 9.09 | 54.84 | -45.75 | 0.01 | 0.23 | ATRNL1 |
| **chr10:120,765,353-121,103,299** | q26.11 | CN Loss | 337946 | 9.09 | 54.84 | -45.75 | 0.01 | 0.23 | NANOS1, EIF3A, SNORA19, FAM45A, FAM45B, SFXN4, PRDX3, GRK5 |
| **chr10:122,384,657-122,592,109** | q26.12 | CN Loss | 207452 | 9.09 | 54.84 | -45.75 | 0.01 | 0.23 | WDR11-AS1, MIR5694 |
| **chr10:122,657,539-123,168,874** | q26.12 - q26.13 | CN Loss | 511335 | 9.09 | 54.84 | -45.75 | 0.01 | 0.23 | MIR5694, WDR11 |
| **chr10:123,215,901-123,233,091** | q26.13 | CN Loss | 17190 | 9.09 | 54.84 | -45.75 | 0.01 | 0.23 | FGFR2 |
| **chr10:123,304,704-123,356,844** | q26.13 | CN Loss | 52140 | 9.09 | 54.84 | -45.75 | 0.01 | 0.23 | FGFR2 |
| **chr10:124,090,650-124,097,254** | q26.13 | CN Loss | 6604 | 9.09 | 54.84 | -45.75 | 0.01 | 0.23 |  |
| **chr10:124,307,061-124,634,591** | q26.13 | CN Loss | 327530 | 9.09 | 54.84 | -45.75 | 0.01 | 0.23 | DMBT1, C10orf120, FLJ46361, CUZD1, FAM24B-CUZD1, FAM24B, LOC399815 |
| **chr10:126,081,112-126,139,826** | q26.13 | CN Loss | 58714 | 9.09 | 54.84 | -45.75 | 0.01 | 0.23 | OAT, NKX1-2 |
| **chr10:126,222,887-126,811,709** | q26.13 | CN Loss | 588822 | 9.09 | 54.84 | -45.75 | 0.01 | 0.23 | LHPP, FAM53B, METTL10, FAM175B, ZRANB1, MIR4296, CTBP2 |
| **chr10:129,553,629-129,777,017** | q26.2 | CN Loss | 223388 | 9.09 | 54.84 | -45.75 | 0.01 | 0.23 | CLRN3, PTPRE |
| **chr11:554,770-848,511** | p15.5 | CN Loss | 293741 | 9.09 | 54.84 | -45.75 | 0.01 | 0.23 | MIR210HG, MIR210, LOC143666, PHRF1, IRF7, CDHR5, SCT, DRD4, DEAF1, TMEM80, EPS8L2, TALDO1, PDDC1, NS3BP, CEND1, SLC25A22, PIDD, RPLP2, SNORA52, PNPLA2, EFCAB4A, CD151, POLR2L, TSPAN4 |
| **chr6:112,542,780-112,660,867** | q21 | CN Loss | 118087 | 36.36 | 3.23 | 33.14 | 0.01 | 0.23 | LAMA4 |
| **chr10:119,164,210-119,205,769** | q26.11 | CN Loss | 41559 | 18.18 | 64.52 | -46.33 | 0.01 | 0.24 |  |
| **chr10:119,460,169-119,511,817** | q26.11 | CN Loss | 51648 | 18.18 | 64.52 | -46.33 | 0.01 | 0.24 |  |
