# Supplementary material for: DNA copy number analysis of Grade II–III and Grade IV gliomas reveals differences in molecular ontogeny including chromothripsis associated with IDH mutation status
Source: Acta Neuropathol Commun. 2015 Jun 20;3:34. doi: 10.1186/s40478-015-0213-3 (PMC4474351; doi:10.1186/s40478-015-0213-3)
Supplement: Additional file 5: Table S5. — Loci with copy number alterations that are significantly different between low and high grade IDH mut gliomas with FDR <0.25. [file 40478_2015_213_MOESM5_ESM.docx]

| Region | Cytoband Location | Event | Region Length | Freq. in <2_3_mut> (%) | Freq. in <4_mut>(%) | Difference | p-value | q-bound | Gene Symbols |
| --- | --- | --- | --- | --- | --- | --- | --- | --- | --- |
| chr1:181,695,458-181,796,900 | q25.3 | CN Gain | 101442 | 0.00 | 45.45 | -45.45 | 0.00 | 0.50 | SMG7-AS1, SMG7, NCF2 |
| chr1:198,799,426-198,833,403 | q32.1 | CN Gain | 33977 | 0.00 | 45.45 | -45.45 | 0.00 | 0.50 | KIF14 |
| chr1:198,833,403-198,881,618 | q32.1 | CN Gain | 48215 | 0.00 | 50.00 | -50.00 | 0.00 | 0.43 | KIF14, DDX59 |
| chr1:201,478,013-201,543,422 | q32.1 | CN Gain | 65409 | 17.39 | 72.73 | -55.34 | 0.00 | 0.56 | LOC730227, BTG2 |
| chr3:50,373,069-51,961,630 | p21.31 - p21.1 | CN Loss | 1588561 | 0.00 | 45.45 | -45.45 | 0.00 | 0.26 | CACNA2D2, C3orf18, HEMK1, CISH, MAPKAPK3, MIR4787, DOCK3, MANF, RBM15B, VPRBP, RAD54L2, TEX264, GRM2, IQCF6, IQCF4, IQCF3, IQCF2, IQCF5, IQCF1, RRP9, PARP3 |
| chr3:53,041,951-53,117,536 | p21.1 | CN Loss | 75585 | 0.00 | 45.45 | -45.45 | 0.00 | 0.26 | SFMBT1, RFT1 |
| chr6:44,308,055-44,327,246 | p21.1 | CN Gain | 19191 | 0.00 | 50.00 | -50.00 | 0.00 | 0.43 | SLC29A1, HSP90AB1 |
| chr6:44,327,246-44,348,765 | p21.1 | CN Gain | 21519 | 0.00 | 45.45 | -45.45 | 0.00 | 0.50 | HSP90AB1, MIR4647, SLC35B2, NFKBIE, TMEM151B |
| chr11:731,692-1,825,949 | p15.5 | LOH | 1094257 | 4.35 | 59.09 | -54.74 | 0.00 | 0.08 | TALDO1, PDDC1, NS3BP, CEND1, SLC25A22, PIDD, RPLP2, SNORA52, PNPLA2, EFCAB4A, CD151, POLR2L, TSPAN4, CHID1, AP2A2, MUC6, MUC2, MUC5B, TOLLIP, LOC255512, BRSK2, DUSP8, KRTAP5-1, LOC338651, KRTAP5-2, KRTAP5-3, MOB2, KRTAP5-4, KRTAP5-5, FAM99A, FAM99B, KRTAP5-6, IFITM10, CTSD, SYT8, TNNI2 |
| chr11:1,825,949-2,022,733 | p15.5 | LOH | 196784 | 4.35 | 63.64 | -59.29 | 0.00 | 0.05 | MIR4298, LSP1, TNNT3, MRPL23, MRPL23-AS1, H19, MIR675 |
| chr11:2,022,733-2,385,565 | p15.5 | LOH | 362832 | 4.35 | 59.09 | -54.74 | 0.00 | 0.08 | MIR483, IGF2, IGF2-AS, INS-IGF2, INS, TH, MIR4686, ASCL2, C11orf21, TSPAN32, CD81, TSSC4, TRPM5 |
| chr11:2,385,565-2,613,398 | p15.5 | LOH | 227833 | 4.35 | 54.55 | -50.20 | 0.00 | 0.16 | TRPM5, KCNQ1, KCNQ1OT1 |
| chr11:2,613,398-3,136,236 | p15.5 - p15.4 | LOH | 522838 | 4.35 | 59.09 | -54.74 | 0.00 | 0.08 | KCNQ1, KCNQ1OT1, KCNQ1DN, CDKN1C, SLC22A18AS, SLC22A18, PHLDA2, SNORA54, NAP1L4, CARS, OSBPL5 |
| chr11:3,136,236-3,253,968 | p15.4 | LOH | 117732 | 4.35 | 63.64 | -59.29 | 0.00 | 0.05 | OSBPL5, MRGPRG, MRGPRG-AS1, MRGPRE |
| chr11:3,253,968-3,289,428 | p15.4 | LOH | 35460 | 4.35 | 68.18 | -63.83 | 0.00 | 0.05 |  |
| chr11:3,289,428-3,323,140 | p15.4 | LOH | 33712 | 4.35 | 72.73 | -68.38 | 0.00 | 0.03 |  |
| chr11:3,323,140-3,373,323 | p15.4 | LOH | 50183 | 8.70 | 72.73 | -64.03 | 0.00 | 0.05 | ZNF195, OR7E12P, LOC650368 |
| chr11:3,373,323-3,533,844 | p15.4 | LOH | 160521 | 8.70 | 68.18 | -59.49 | 0.00 | 0.07 | LOC650368 |
| chr11:3,533,844-3,648,686 | p15.4 | LOH | 114842 | 8.70 | 72.73 | -64.03 | 0.00 | 0.05 | TRPC2, ART5, ART1, CHRNA10 |
| chr11:3,648,686-3,796,784 | p15.4 | LOH | 148098 | 8.70 | 68.18 | -59.49 | 0.00 | 0.07 | CHRNA10, NUP98, PGAP2 |
| chr11:3,796,784-3,841,095 | p15.4 | LOH | 44311 | 8.70 | 63.64 | -54.94 | 0.00 | 0.15 | PGAP2, RHOG, MIR4687, STIM1 |
| chr11:3,841,095-3,870,162 | p15.4 | LOH | 29067 | 8.70 | 68.18 | -59.49 | 0.00 | 0.07 | STIM1 |
| chr11:3,870,162-3,881,765 | p15.4 | LOH | 11603 | 13.04 | 68.18 | -55.14 | 0.00 | 0.16 | STIM1 |
| chr11:4,773,651-5,320,948 | p15.4 | LOH | 547297 | 4.35 | 54.55 | -50.20 | 0.00 | 0.16 | OR52R1, OR51F2, OR51S1, OR51T1, OR51A7, OR51G2, OR51G1, OR51A4, OR51A2, MMP26, OR51L1, OR52J3, OR52E2, OR52A5, OR52A1, OR51V1, HBB, HBD, HBBP1, HBG1, HBG2, HBE1, OR51B4, OR51B2, OR51B5 |
| chr11:7,362,797-7,575,680 | p15.4 | LOH | 212883 | 4.35 | 54.55 | -50.20 | 0.00 | 0.16 | SYT9, OLFML1, PPFIBP2 |
| chr11:10,942,698-11,147,505 | p15.3 | LOH | 204807 | 4.35 | 54.55 | -50.20 | 0.00 | 0.16 |  |
| chr11:11,147,505-11,234,327 | p15.3 | LOH | 86822 | 0.00 | 54.55 | -54.55 | 0.00 | 0.05 |  |
| chr11:11,234,327-11,403,561 | p15.3 | LOH | 169234 | 0.00 | 50.00 | -50.00 | 0.00 | 0.08 | CSNK2A1P, GALNTL4 |
| chr11:31,325,066-31,763,862 | p13 | LOH | 438796 | 4.35 | 54.55 | -50.20 | 0.00 | 0.16 | DCDC1, DNAJC24, IMMP1L, ELP4, PAX6 |
