# Supplementary material for: DNA copy number analysis of Grade II–III and Grade IV gliomas reveals differences in molecular ontogeny including chromothripsis associated with IDH mutation status
Source: Acta Neuropathol Commun. 2015 Jun 20;3:34. doi: 10.1186/s40478-015-0213-3 (PMC4474351; doi:10.1186/s40478-015-0213-3)
Supplement: Additional file 6: Table S6. — Gene Ontology categories and pathways enriched using the ToppGene algorithm for (A) IDH mut Grade II–III gliomas, (B) IDH mut GBM, (C) IDH wt Grade II–III gliomas, (D) IDH wt GBM, (E) differences between IDH mut lower grade and high grade gliomas, and (F) differences between IDH wt low and high grade gliomas. [file 40478_2015_213_MOESM6_ESM.docx]

A IDH mutant, grade II-III

| GO | ID | Name | Source | pValue | FDR B&H | FDR B&Y | Bonferroni | Genes from Input | Genes in Annotation |
| --- | --- | --- | --- | --- | --- | --- | --- | --- | --- |
| 1 | GO:0004816 | asparagine-tRNA ligase activity |  | 2.29E-05 | 7.13E-03 | 4.51E-02 | 7.13E-03 | [2](http://toppgene.cchmc.org/showQueryTerms.jsp?userdata_id=46e8fe9c-285e-43e2-9e4f-18c592f84902&feature=gof&row=0) | [2](http://toppgene.cchmc.org/showTermDetail.jsp?userdata_id=46e8fe9c-285e-43e2-9e4f-18c592f84902&category=GeneOntologyMolecularFunction&id=GO:0004816) |

| Pathway | ID | Name | Source | pValue | FDR B&H | FDR B&Y | Bonferroni | Genes from Input | Genes in Annotation |
| --- | --- | --- | --- | --- | --- | --- | --- | --- | --- |
| 1 | 83114 | Melanoma | [BioSystems: KEGG](http://www.ncbi.nlm.nih.gov/biosystems/83114) | 4.54E-05 | 1.29E-02 | 9.03E-02 | 2.85E-02 | [5](http://toppgene.cchmc.org/showQueryTerms.jsp?userdata_id=46e8fe9c-285e-43e2-9e4f-18c592f84902&feature=pt&row=0) | [71](http://toppgene.cchmc.org/showTermDetail.jsp?userdata_id=46e8fe9c-285e-43e2-9e4f-18c592f84902&category=Pathway&id=83114) |
| 2 | 833829 | Role of LAT2/NTAL/LAB on calcium mobilization | [BioSystems: REACTOME](http://www.ncbi.nlm.nih.gov/biosystems/833829) | 6.36E-05 | 1.29E-02 | 9.03E-02 | 4.00E-02 | [6](http://toppgene.cchmc.org/showQueryTerms.jsp?userdata_id=46e8fe9c-285e-43e2-9e4f-18c592f84902&feature=pt&row=1) | [124](http://toppgene.cchmc.org/showTermDetail.jsp?userdata_id=46e8fe9c-285e-43e2-9e4f-18c592f84902&category=Pathway&id=833829) |
| 3 | 833825 | Fc epsilon receptor (FCERI) signaling | [BioSystems: REACTOME](http://www.ncbi.nlm.nih.gov/biosystems/833825) | 9.77E-05 | 1.29E-02 | 9.03E-02 | 6.13E-02 | [7](http://toppgene.cchmc.org/showQueryTerms.jsp?userdata_id=46e8fe9c-285e-43e2-9e4f-18c592f84902&feature=pt&row=2) | [194](http://toppgene.cchmc.org/showTermDetail.jsp?userdata_id=46e8fe9c-285e-43e2-9e4f-18c592f84902&category=Pathway&id=833825) |
| 4 | 477120 | Signaling by SCF-KIT | [BioSystems: REACTOME](http://www.ncbi.nlm.nih.gov/biosystems/477120) | 1.15E-04 | 1.29E-02 | 9.03E-02 | 7.24E-02 | [6](http://toppgene.cchmc.org/showQueryTerms.jsp?userdata_id=46e8fe9c-285e-43e2-9e4f-18c592f84902&feature=pt&row=3) | [138](http://toppgene.cchmc.org/showTermDetail.jsp?userdata_id=46e8fe9c-285e-43e2-9e4f-18c592f84902&category=Pathway&id=477120) |
| 5 | 685535 | Constitutive PI3K/AKT Signaling in Cancer | [BioSystems: REACTOME](http://www.ncbi.nlm.nih.gov/biosystems/685535) | 1.20E-04 | 1.29E-02 | 9.03E-02 | 7.56E-02 | [5](http://toppgene.cchmc.org/showQueryTerms.jsp?userdata_id=46e8fe9c-285e-43e2-9e4f-18c592f84902&feature=pt&row=4) | [87](http://toppgene.cchmc.org/showTermDetail.jsp?userdata_id=46e8fe9c-285e-43e2-9e4f-18c592f84902&category=Pathway&id=685535) |
| 6 | 685534 | PI3K/AKT Signaling in Cancer | [BioSystems: REACTOME](http://www.ncbi.nlm.nih.gov/biosystems/685534) | 2.21E-04 | 1.29E-02 | 9.03E-02 | 1.39E-01 | [5](http://toppgene.cchmc.org/showQueryTerms.jsp?userdata_id=46e8fe9c-285e-43e2-9e4f-18c592f84902&feature=pt&row=5) | [99](http://toppgene.cchmc.org/showTermDetail.jsp?userdata_id=46e8fe9c-285e-43e2-9e4f-18c592f84902&category=Pathway&id=685534) |
| 7 | 530737 | PI3K events in ERBB2 signaling | [BioSystems: REACTOME](http://www.ncbi.nlm.nih.gov/biosystems/530737) | 2.21E-04 | 1.29E-02 | 9.03E-02 | 1.39E-01 | [5](http://toppgene.cchmc.org/showQueryTerms.jsp?userdata_id=46e8fe9c-285e-43e2-9e4f-18c592f84902&feature=pt&row=6) | [99](http://toppgene.cchmc.org/showTermDetail.jsp?userdata_id=46e8fe9c-285e-43e2-9e4f-18c592f84902&category=Pathway&id=530737) |
| 8 | 366239 | PIP3 activates AKT signaling | [BioSystems: REACTOME](http://www.ncbi.nlm.nih.gov/biosystems/366239) | 2.21E-04 | 1.29E-02 | 9.03E-02 | 1.39E-01 | [5](http://toppgene.cchmc.org/showQueryTerms.jsp?userdata_id=46e8fe9c-285e-43e2-9e4f-18c592f84902&feature=pt&row=7) | [99](http://toppgene.cchmc.org/showTermDetail.jsp?userdata_id=46e8fe9c-285e-43e2-9e4f-18c592f84902&category=Pathway&id=366239) |
| 9 | 530743 | PI3K events in ERBB4 signaling | [BioSystems: REACTOME](http://www.ncbi.nlm.nih.gov/biosystems/530743) | 2.21E-04 | 1.29E-02 | 9.03E-02 | 1.39E-01 | [5](http://toppgene.cchmc.org/showQueryTerms.jsp?userdata_id=46e8fe9c-285e-43e2-9e4f-18c592f84902&feature=pt&row=8) | [99](http://toppgene.cchmc.org/showTermDetail.jsp?userdata_id=46e8fe9c-285e-43e2-9e4f-18c592f84902&category=Pathway&id=530743) |
| 10 | 160961 | PI-3K cascade | [BioSystems: REACTOME](http://www.ncbi.nlm.nih.gov/biosystems/160961) | 2.21E-04 | 1.29E-02 | 9.03E-02 | 1.39E-01 | [5](http://toppgene.cchmc.org/showQueryTerms.jsp?userdata_id=46e8fe9c-285e-43e2-9e4f-18c592f84902&feature=pt&row=9) | [99](http://toppgene.cchmc.org/showTermDetail.jsp?userdata_id=46e8fe9c-285e-43e2-9e4f-18c592f84902&category=Pathway&id=160961) |
| 11 | 782000 | Proteoglycans in cancer | [BioSystems: KEGG](http://www.ncbi.nlm.nih.gov/biosystems/782000) | 2.45E-04 | 1.29E-02 | 9.03E-02 | 1.54E-01 | [7](http://toppgene.cchmc.org/showQueryTerms.jsp?userdata_id=46e8fe9c-285e-43e2-9e4f-18c592f84902&feature=pt&row=10) | [225](http://toppgene.cchmc.org/showTermDetail.jsp?userdata_id=46e8fe9c-285e-43e2-9e4f-18c592f84902&category=Pathway&id=782000) |
| 12 | 106474 | PI3K/AKT activation | [BioSystems: REACTOME](http://www.ncbi.nlm.nih.gov/biosystems/106474) | 2.55E-04 | 1.29E-02 | 9.03E-02 | 1.60E-01 | [5](http://toppgene.cchmc.org/showQueryTerms.jsp?userdata_id=46e8fe9c-285e-43e2-9e4f-18c592f84902&feature=pt&row=11) | [102](http://toppgene.cchmc.org/showTermDetail.jsp?userdata_id=46e8fe9c-285e-43e2-9e4f-18c592f84902&category=Pathway&id=106474) |
| 13 | 106341 | GAB1 signalosome | [BioSystems: REACTOME](http://www.ncbi.nlm.nih.gov/biosystems/106341) | 2.66E-04 | 1.29E-02 | 9.03E-02 | 1.67E-01 | [5](http://toppgene.cchmc.org/showQueryTerms.jsp?userdata_id=46e8fe9c-285e-43e2-9e4f-18c592f84902&feature=pt&row=12) | [103](http://toppgene.cchmc.org/showTermDetail.jsp?userdata_id=46e8fe9c-285e-43e2-9e4f-18c592f84902&category=Pathway&id=106341) |
| 14 | 576250 | Downstream Signaling Events Of B Cell Receptor (BCR) | [BioSystems: REACTOME](http://www.ncbi.nlm.nih.gov/biosystems/576250) | 3.70E-04 | 1.65E-02 | 1.16E-01 | 2.32E-01 | [6](http://toppgene.cchmc.org/showQueryTerms.jsp?userdata_id=46e8fe9c-285e-43e2-9e4f-18c592f84902&feature=pt&row=13) | [171](http://toppgene.cchmc.org/showTermDetail.jsp?userdata_id=46e8fe9c-285e-43e2-9e4f-18c592f84902&category=Pathway&id=576250) |
| 15 | 645274 | Signaling by FGFR in disease | [BioSystems: REACTOME](http://www.ncbi.nlm.nih.gov/biosystems/645274) | 3.93E-04 | 1.65E-02 | 1.16E-01 | 2.47E-01 | [6](http://toppgene.cchmc.org/showQueryTerms.jsp?userdata_id=46e8fe9c-285e-43e2-9e4f-18c592f84902&feature=pt&row=14) | [173](http://toppgene.cchmc.org/showTermDetail.jsp?userdata_id=46e8fe9c-285e-43e2-9e4f-18c592f84902&category=Pathway&id=645274) |
| 16 | 106384 | Signaling by PDGF | [BioSystems: REACTOME](http://www.ncbi.nlm.nih.gov/biosystems/106384) | 4.72E-04 | 1.85E-02 | 1.30E-01 | 2.96E-01 | [6](http://toppgene.cchmc.org/showQueryTerms.jsp?userdata_id=46e8fe9c-285e-43e2-9e4f-18c592f84902&feature=pt&row=15) | [179](http://toppgene.cchmc.org/showTermDetail.jsp?userdata_id=46e8fe9c-285e-43e2-9e4f-18c592f84902&category=Pathway&id=106384) |
| 17 | 645276 | Signaling by FGFR1 mutants | [BioSystems: REACTOME](http://www.ncbi.nlm.nih.gov/biosystems/645276) | 6.07E-04 | 2.23E-02 | 1.57E-01 | 3.81E-01 | [3](http://toppgene.cchmc.org/showQueryTerms.jsp?userdata_id=46e8fe9c-285e-43e2-9e4f-18c592f84902&feature=pt&row=16) | [30](http://toppgene.cchmc.org/showTermDetail.jsp?userdata_id=46e8fe9c-285e-43e2-9e4f-18c592f84902&category=Pathway&id=645276) |
| 18 | 692234 | PI3K-Akt signaling pathway | [BioSystems: KEGG](http://www.ncbi.nlm.nih.gov/biosystems/692234) | 6.40E-04 | 2.23E-02 | 1.57E-01 | 4.02E-01 | [8](http://toppgene.cchmc.org/showQueryTerms.jsp?userdata_id=46e8fe9c-285e-43e2-9e4f-18c592f84902&feature=pt&row=17) | [346](http://toppgene.cchmc.org/showTermDetail.jsp?userdata_id=46e8fe9c-285e-43e2-9e4f-18c592f84902&category=Pathway&id=692234) |
| 19 | 138037 | EGFR-dependent Endothelin signaling events | [BioSystems: Pathway Interaction Database](http://www.ncbi.nlm.nih.gov/biosystems/138037) | 8.44E-04 | 2.79E-02 | 1.96E-01 | 5.30E-01 | [2](http://toppgene.cchmc.org/showQueryTerms.jsp?userdata_id=46e8fe9c-285e-43e2-9e4f-18c592f84902&feature=pt&row=18) | [8](http://toppgene.cchmc.org/showTermDetail.jsp?userdata_id=46e8fe9c-285e-43e2-9e4f-18c592f84902&category=Pathway&id=138037) |
| 20 | 672458 | Signaling Pathways in Glioblastoma | [BioSystems: WikiPathways](http://www.ncbi.nlm.nih.gov/biosystems/672458) | 1.02E-03 | 3.12E-02 | 2.19E-01 | 6.40E-01 | [4](http://toppgene.cchmc.org/showQueryTerms.jsp?userdata_id=46e8fe9c-285e-43e2-9e4f-18c592f84902&feature=pt&row=19) | [80](http://toppgene.cchmc.org/showTermDetail.jsp?userdata_id=46e8fe9c-285e-43e2-9e4f-18c592f84902&category=Pathway&id=672458) |
| 21 | 169350 | Signaling events mediated by TCPTP | [BioSystems: Pathway Interaction Database](http://www.ncbi.nlm.nih.gov/biosystems/169350) | 1.04E-03 | 3.12E-02 | 2.19E-01 | 6.55E-01 | [3](http://toppgene.cchmc.org/showQueryTerms.jsp?userdata_id=46e8fe9c-285e-43e2-9e4f-18c592f84902&feature=pt&row=20) | [36](http://toppgene.cchmc.org/showTermDetail.jsp?userdata_id=46e8fe9c-285e-43e2-9e4f-18c592f84902&category=Pathway&id=169350) |
| 22 | 160957 | Downstream signaling of activated FGFR | [BioSystems: REACTOME](http://www.ncbi.nlm.nih.gov/biosystems/160957) | 1.23E-03 | 3.38E-02 | 2.37E-01 | 7.72E-01 | [5](http://toppgene.cchmc.org/showQueryTerms.jsp?userdata_id=46e8fe9c-285e-43e2-9e4f-18c592f84902&feature=pt&row=21) | [144](http://toppgene.cchmc.org/showTermDetail.jsp?userdata_id=46e8fe9c-285e-43e2-9e4f-18c592f84902&category=Pathway&id=160957) |
| 23 | 852705 | MicroRNAs in cancer | [BioSystems: KEGG](http://www.ncbi.nlm.nih.gov/biosystems/852705) | 1.25E-03 | 3.38E-02 | 2.37E-01 | 7.88E-01 | [7](http://toppgene.cchmc.org/showQueryTerms.jsp?userdata_id=46e8fe9c-285e-43e2-9e4f-18c592f84902&feature=pt&row=22) | [296](http://toppgene.cchmc.org/showTermDetail.jsp?userdata_id=46e8fe9c-285e-43e2-9e4f-18c592f84902&category=Pathway&id=852705) |
| 24 | 576248 | Signaling by the B Cell Receptor (BCR) | [BioSystems: REACTOME](http://www.ncbi.nlm.nih.gov/biosystems/576248) | 1.29E-03 | 3.38E-02 | 2.37E-01 | 8.10E-01 | [6](http://toppgene.cchmc.org/showQueryTerms.jsp?userdata_id=46e8fe9c-285e-43e2-9e4f-18c592f84902&feature=pt&row=23) | [217](http://toppgene.cchmc.org/showTermDetail.jsp?userdata_id=46e8fe9c-285e-43e2-9e4f-18c592f84902&category=Pathway&id=576248) |
| 25 | 530741 | Signaling by ERBB4 | [BioSystems: REACTOME](http://www.ncbi.nlm.nih.gov/biosystems/530741) | 1.43E-03 | 3.59E-02 | 2.52E-01 | 8.98E-01 | [5](http://toppgene.cchmc.org/showQueryTerms.jsp?userdata_id=46e8fe9c-285e-43e2-9e4f-18c592f84902&feature=pt&row=24) | [149](http://toppgene.cchmc.org/showTermDetail.jsp?userdata_id=46e8fe9c-285e-43e2-9e4f-18c592f84902&category=Pathway&id=530741) |
| 26 | 868085 | Ras signaling pathway | [BioSystems: KEGG](http://www.ncbi.nlm.nih.gov/biosystems/868085) | 1.62E-03 | 3.68E-02 | 2.58E-01 | 1.00E+00 | [6](http://toppgene.cchmc.org/showQueryTerms.jsp?userdata_id=46e8fe9c-285e-43e2-9e4f-18c592f84902&feature=pt&row=25) | [227](http://toppgene.cchmc.org/showTermDetail.jsp?userdata_id=46e8fe9c-285e-43e2-9e4f-18c592f84902&category=Pathway&id=868085) |
| 27 | 645279 | Signaling by activated point mutants of FGFR1 | [BioSystems: REACTOME](http://www.ncbi.nlm.nih.gov/biosystems/645279) | 1.64E-03 | 3.68E-02 | 2.58E-01 | 1.00E+00 | [2](http://toppgene.cchmc.org/showQueryTerms.jsp?userdata_id=46e8fe9c-285e-43e2-9e4f-18c592f84902&feature=pt&row=26) | [11](http://toppgene.cchmc.org/showTermDetail.jsp?userdata_id=46e8fe9c-285e-43e2-9e4f-18c592f84902&category=Pathway&id=645279) |
| 28 | 106347 | FGFR1c ligand binding and activation | [BioSystems: REACTOME](http://www.ncbi.nlm.nih.gov/biosystems/106347) | 1.64E-03 | 3.68E-02 | 2.58E-01 | 1.00E+00 | [2](http://toppgene.cchmc.org/showQueryTerms.jsp?userdata_id=46e8fe9c-285e-43e2-9e4f-18c592f84902&feature=pt&row=27) | [11](http://toppgene.cchmc.org/showTermDetail.jsp?userdata_id=46e8fe9c-285e-43e2-9e4f-18c592f84902&category=Pathway&id=106347) |
| 29 | 106385 | Downstream signal transduction | [BioSystems: REACTOME](http://www.ncbi.nlm.nih.gov/biosystems/106385) | 1.75E-03 | 3.74E-02 | 2.62E-01 | 1.00E+00 | [5](http://toppgene.cchmc.org/showQueryTerms.jsp?userdata_id=46e8fe9c-285e-43e2-9e4f-18c592f84902&feature=pt&row=28) | [156](http://toppgene.cchmc.org/showTermDetail.jsp?userdata_id=46e8fe9c-285e-43e2-9e4f-18c592f84902&category=Pathway&id=106385) |
| 30 | 106343 | Signaling by FGFR | [BioSystems: REACTOME](http://www.ncbi.nlm.nih.gov/biosystems/106343) | 1.85E-03 | 3.74E-02 | 2.62E-01 | 1.00E+00 | [5](http://toppgene.cchmc.org/showQueryTerms.jsp?userdata_id=46e8fe9c-285e-43e2-9e4f-18c592f84902&feature=pt&row=29) | [158](http://toppgene.cchmc.org/showTermDetail.jsp?userdata_id=46e8fe9c-285e-43e2-9e4f-18c592f84902&category=Pathway&id=106343) |
| 31 | 530734 | Signaling by ERBB2 | [BioSystems: REACTOME](http://www.ncbi.nlm.nih.gov/biosystems/530734) | 1.91E-03 | 3.74E-02 | 2.62E-01 | 1.00E+00 | [5](http://toppgene.cchmc.org/showQueryTerms.jsp?userdata_id=46e8fe9c-285e-43e2-9e4f-18c592f84902&feature=pt&row=30) | [159](http://toppgene.cchmc.org/showTermDetail.jsp?userdata_id=46e8fe9c-285e-43e2-9e4f-18c592f84902&category=Pathway&id=530734) |
| 32 | 685550 | DAP12 signaling | [BioSystems: REACTOME](http://www.ncbi.nlm.nih.gov/biosystems/685550) | 1.91E-03 | 3.74E-02 | 2.62E-01 | 1.00E+00 | [5](http://toppgene.cchmc.org/showQueryTerms.jsp?userdata_id=46e8fe9c-285e-43e2-9e4f-18c592f84902&feature=pt&row=31) | [159](http://toppgene.cchmc.org/showTermDetail.jsp?userdata_id=46e8fe9c-285e-43e2-9e4f-18c592f84902&category=Pathway&id=685550) |
| 33 | 645275 | Signaling by FGFR mutants | [BioSystems: REACTOME](http://www.ncbi.nlm.nih.gov/biosystems/645275) | 2.00E-03 | 3.81E-02 | 2.67E-01 | 1.00E+00 | [3](http://toppgene.cchmc.org/showQueryTerms.jsp?userdata_id=46e8fe9c-285e-43e2-9e4f-18c592f84902&feature=pt&row=32) | [45](http://toppgene.cchmc.org/showTermDetail.jsp?userdata_id=46e8fe9c-285e-43e2-9e4f-18c592f84902&category=Pathway&id=645275) |
| 34 | 477129 | Developmental Biology | [BioSystems: REACTOME](http://www.ncbi.nlm.nih.gov/biosystems/477129) | 2.19E-03 | 3.81E-02 | 2.68E-01 | 1.00E+00 | [8](http://toppgene.cchmc.org/showQueryTerms.jsp?userdata_id=46e8fe9c-285e-43e2-9e4f-18c592f84902&feature=pt&row=33) | [419](http://toppgene.cchmc.org/showTermDetail.jsp?userdata_id=46e8fe9c-285e-43e2-9e4f-18c592f84902&category=Pathway&id=477129) |
| 35 | 83105 | Pathways in cancer | [BioSystems: KEGG](http://www.ncbi.nlm.nih.gov/biosystems/83105) | 2.22E-03 | 3.81E-02 | 2.68E-01 | 1.00E+00 | [7](http://toppgene.cchmc.org/showQueryTerms.jsp?userdata_id=46e8fe9c-285e-43e2-9e4f-18c592f84902&feature=pt&row=34) | [327](http://toppgene.cchmc.org/showTermDetail.jsp?userdata_id=46e8fe9c-285e-43e2-9e4f-18c592f84902&category=Pathway&id=83105) |
| 36 | 106351 | FGFR2c ligand binding and activation | [BioSystems: REACTOME](http://www.ncbi.nlm.nih.gov/biosystems/106351) | 2.31E-03 | 3.81E-02 | 2.68E-01 | 1.00E+00 | [2](http://toppgene.cchmc.org/showQueryTerms.jsp?userdata_id=46e8fe9c-285e-43e2-9e4f-18c592f84902&feature=pt&row=35) | [13](http://toppgene.cchmc.org/showTermDetail.jsp?userdata_id=46e8fe9c-285e-43e2-9e4f-18c592f84902&category=Pathway&id=106351) |
| 37 | 106355 | FGFR4 ligand binding and activation | [BioSystems: REACTOME](http://www.ncbi.nlm.nih.gov/biosystems/106355) | 2.31E-03 | 3.81E-02 | 2.68E-01 | 1.00E+00 | [2](http://toppgene.cchmc.org/showQueryTerms.jsp?userdata_id=46e8fe9c-285e-43e2-9e4f-18c592f84902&feature=pt&row=36) | [13](http://toppgene.cchmc.org/showTermDetail.jsp?userdata_id=46e8fe9c-285e-43e2-9e4f-18c592f84902&category=Pathway&id=106355) |
| 38 | M16973 | CBL mediated ligand-induced downregulation of EGF receptors | [MSigDB C2: BioCarta](http://www.broadinstitute.org/gsea/msigdb/cards/BIOCARTA_CBL_PATHWAY.html) | 2.31E-03 | 3.81E-02 | 2.68E-01 | 1.00E+00 | [2](http://toppgene.cchmc.org/showQueryTerms.jsp?userdata_id=46e8fe9c-285e-43e2-9e4f-18c592f84902&feature=pt&row=37) | [13](http://toppgene.cchmc.org/showTermDetail.jsp?userdata_id=46e8fe9c-285e-43e2-9e4f-18c592f84902&category=Pathway&id=M16973) |
| 39 | 106345 | FGFR1 ligand binding and activation | [BioSystems: REACTOME](http://www.ncbi.nlm.nih.gov/biosystems/106345) | 2.68E-03 | 4.32E-02 | 3.03E-01 | 1.00E+00 | [2](http://toppgene.cchmc.org/showQueryTerms.jsp?userdata_id=46e8fe9c-285e-43e2-9e4f-18c592f84902&feature=pt&row=38) | [14](http://toppgene.cchmc.org/showTermDetail.jsp?userdata_id=46e8fe9c-285e-43e2-9e4f-18c592f84902&category=Pathway&id=106345) |
| 40 | 106337 | Signaling by EGFR | [BioSystems: REACTOME](http://www.ncbi.nlm.nih.gov/biosystems/106337) | 2.96E-03 | 4.65E-02 | 3.27E-01 | 1.00E+00 | [5](http://toppgene.cchmc.org/showQueryTerms.jsp?userdata_id=46e8fe9c-285e-43e2-9e4f-18c592f84902&feature=pt&row=39) | [176](http://toppgene.cchmc.org/showTermDetail.jsp?userdata_id=46e8fe9c-285e-43e2-9e4f-18c592f84902&category=Pathway&id=106337) |
| 41 | 530768 | Signaling by EGFR in Cancer | [BioSystems: REACTOME](http://www.ncbi.nlm.nih.gov/biosystems/530768) | 3.11E-03 | 4.77E-02 | 3.35E-01 | 1.00E+00 | [5](http://toppgene.cchmc.org/showQueryTerms.jsp?userdata_id=46e8fe9c-285e-43e2-9e4f-18c592f84902&feature=pt&row=40) | [178](http://toppgene.cchmc.org/showTermDetail.jsp?userdata_id=46e8fe9c-285e-43e2-9e4f-18c592f84902&category=Pathway&id=530768) |
| 42 | 685549 | DAP12 interactions | [BioSystems: REACTOME](http://www.ncbi.nlm.nih.gov/biosystems/685549) | 3.19E-03 | 4.77E-02 | 3.35E-01 | 1.00E+00 | [5](http://toppgene.cchmc.org/showQueryTerms.jsp?userdata_id=46e8fe9c-285e-43e2-9e4f-18c592f84902&feature=pt&row=41) | [179](http://toppgene.cchmc.org/showTermDetail.jsp?userdata_id=46e8fe9c-285e-43e2-9e4f-18c592f84902&category=Pathway&id=685549) |

B IDH mutant, GBM

| GO | ID | Name | Source | pValue | FDR B&H | FDR B&Y | Bonferroni | Genes from Input | Genes in Annotation |
| --- | --- | --- | --- | --- | --- | --- | --- | --- | --- |
| 1 | GO:0042127 | regulation of cell proliferation |  | 1.63E-08 | 3.85E-05 | 3.21E-04 | 3.85E-05 | [25](http://toppgene.cchmc.org/showQueryTerms.jsp?userdata_id=41d53c5f-f68c-4e9f-8754-37ad37cd4269&feature=gop&row=0) | [1362](http://toppgene.cchmc.org/showTermDetail.jsp?userdata_id=41d53c5f-f68c-4e9f-8754-37ad37cd4269&category=GeneOntologyBiologicalProcess&id=GO:0042127) |
| 2 | GO:0061180 | mammary gland epithelium development |  | 2.69E-06 | 2.30E-03 | 1.92E-02 | 6.34E-03 | [6](http://toppgene.cchmc.org/showQueryTerms.jsp?userdata_id=41d53c5f-f68c-4e9f-8754-37ad37cd4269&feature=gop&row=1) | [76](http://toppgene.cchmc.org/showTermDetail.jsp?userdata_id=41d53c5f-f68c-4e9f-8754-37ad37cd4269&category=GeneOntologyBiologicalProcess&id=GO:0061180) |
| 3 | GO:0048732 | gland development |  | 2.93E-06 | 2.30E-03 | 1.92E-02 | 6.89E-03 | [10](http://toppgene.cchmc.org/showQueryTerms.jsp?userdata_id=41d53c5f-f68c-4e9f-8754-37ad37cd4269&feature=gop&row=2) | [294](http://toppgene.cchmc.org/showTermDetail.jsp?userdata_id=41d53c5f-f68c-4e9f-8754-37ad37cd4269&category=GeneOntologyBiologicalProcess&id=GO:0048732) |
| 4 | GO:0030879 | mammary gland development |  | 7.84E-06 | 3.50E-03 | 2.92E-02 | 1.85E-02 | [7](http://toppgene.cchmc.org/showQueryTerms.jsp?userdata_id=41d53c5f-f68c-4e9f-8754-37ad37cd4269&feature=gop&row=3) | [139](http://toppgene.cchmc.org/showTermDetail.jsp?userdata_id=41d53c5f-f68c-4e9f-8754-37ad37cd4269&category=GeneOntologyBiologicalProcess&id=GO:0030879) |
| 5 | GO:0071900 | regulation of protein serine/threonine kinase activity |  | 8.44E-06 | 3.50E-03 | 2.92E-02 | 1.99E-02 | [11](http://toppgene.cchmc.org/showQueryTerms.jsp?userdata_id=41d53c5f-f68c-4e9f-8754-37ad37cd4269&feature=gop&row=4) | [408](http://toppgene.cchmc.org/showTermDetail.jsp?userdata_id=41d53c5f-f68c-4e9f-8754-37ad37cd4269&category=GeneOntologyBiologicalProcess&id=GO:0071900) |
| 6 | GO:0008284 | positive regulation of cell proliferation |  | 8.90E-06 | 3.50E-03 | 2.92E-02 | 2.10E-02 | [15](http://toppgene.cchmc.org/showQueryTerms.jsp?userdata_id=41d53c5f-f68c-4e9f-8754-37ad37cd4269&feature=gop&row=5) | [767](http://toppgene.cchmc.org/showTermDetail.jsp?userdata_id=41d53c5f-f68c-4e9f-8754-37ad37cd4269&category=GeneOntologyBiologicalProcess&id=GO:0008284) |
| 7 | GO:0045859 | regulation of protein kinase activity |  | 1.14E-05 | 3.83E-03 | 3.20E-02 | 2.68E-02 | [14](http://toppgene.cchmc.org/showQueryTerms.jsp?userdata_id=41d53c5f-f68c-4e9f-8754-37ad37cd4269&feature=gop&row=6) | [686](http://toppgene.cchmc.org/showTermDetail.jsp?userdata_id=41d53c5f-f68c-4e9f-8754-37ad37cd4269&category=GeneOntologyBiologicalProcess&id=GO:0045859) |
| 8 | GO:0006468 | protein phosphorylation |  | 1.85E-05 | 5.27E-03 | 4.40E-02 | 4.36E-02 | [20](http://toppgene.cchmc.org/showQueryTerms.jsp?userdata_id=41d53c5f-f68c-4e9f-8754-37ad37cd4269&feature=gop&row=7) | [1366](http://toppgene.cchmc.org/showTermDetail.jsp?userdata_id=41d53c5f-f68c-4e9f-8754-37ad37cd4269&category=GeneOntologyBiologicalProcess&id=GO:0006468) |
| 9 | GO:0043549 | regulation of kinase activity |  | 2.11E-05 | 5.27E-03 | 4.40E-02 | 4.97E-02 | [14](http://toppgene.cchmc.org/showQueryTerms.jsp?userdata_id=41d53c5f-f68c-4e9f-8754-37ad37cd4269&feature=gop&row=8) | [725](http://toppgene.cchmc.org/showTermDetail.jsp?userdata_id=41d53c5f-f68c-4e9f-8754-37ad37cd4269&category=GeneOntologyBiologicalProcess&id=GO:0043549) |
| 10 | GO:0050673 | epithelial cell proliferation |  | 2.24E-05 | 5.27E-03 | 4.40E-02 | 5.27E-02 | [9](http://toppgene.cchmc.org/showQueryTerms.jsp?userdata_id=41d53c5f-f68c-4e9f-8754-37ad37cd4269&feature=gop&row=9) | [295](http://toppgene.cchmc.org/showTermDetail.jsp?userdata_id=41d53c5f-f68c-4e9f-8754-37ad37cd4269&category=GeneOntologyBiologicalProcess&id=GO:0050673) |
| 11 | GO:0051338 | regulation of transferase activity |  | 2.93E-05 | 6.01E-03 | 5.02E-02 | 6.91E-02 | [14](http://toppgene.cchmc.org/showQueryTerms.jsp?userdata_id=41d53c5f-f68c-4e9f-8754-37ad37cd4269&feature=gop&row=10) | [747](http://toppgene.cchmc.org/showTermDetail.jsp?userdata_id=41d53c5f-f68c-4e9f-8754-37ad37cd4269&category=GeneOntologyBiologicalProcess&id=GO:0051338) |
| 12 | GO:0038093 | Fc receptor signaling pathway |  | 3.50E-05 | 6.01E-03 | 5.02E-02 | 8.23E-02 | [8](http://toppgene.cchmc.org/showQueryTerms.jsp?userdata_id=41d53c5f-f68c-4e9f-8754-37ad37cd4269&feature=gop&row=11) | [240](http://toppgene.cchmc.org/showTermDetail.jsp?userdata_id=41d53c5f-f68c-4e9f-8754-37ad37cd4269&category=GeneOntologyBiologicalProcess&id=GO:0038093) |
| 13 | GO:0030900 | forebrain development |  | 3.56E-05 | 6.01E-03 | 5.02E-02 | 8.38E-02 | [9](http://toppgene.cchmc.org/showQueryTerms.jsp?userdata_id=41d53c5f-f68c-4e9f-8754-37ad37cd4269&feature=gop&row=12) | [313](http://toppgene.cchmc.org/showTermDetail.jsp?userdata_id=41d53c5f-f68c-4e9f-8754-37ad37cd4269&category=GeneOntologyBiologicalProcess&id=GO:0030900) |
| 14 | GO:0021543 | pallium development |  | 3.57E-05 | 6.01E-03 | 5.02E-02 | 8.42E-02 | [6](http://toppgene.cchmc.org/showQueryTerms.jsp?userdata_id=41d53c5f-f68c-4e9f-8754-37ad37cd4269&feature=gop&row=13) | [119](http://toppgene.cchmc.org/showTermDetail.jsp?userdata_id=41d53c5f-f68c-4e9f-8754-37ad37cd4269&category=GeneOntologyBiologicalProcess&id=GO:0021543) |
| 15 | GO:0050678 | regulation of epithelial cell proliferation |  | 4.05E-05 | 6.35E-03 | 5.30E-02 | 9.53E-02 | [8](http://toppgene.cchmc.org/showQueryTerms.jsp?userdata_id=41d53c5f-f68c-4e9f-8754-37ad37cd4269&feature=gop&row=14) | [245](http://toppgene.cchmc.org/showTermDetail.jsp?userdata_id=41d53c5f-f68c-4e9f-8754-37ad37cd4269&category=GeneOntologyBiologicalProcess&id=GO:0050678) |
| 16 | GO:0014002 | astrocyte development |  | 4.71E-05 | 6.93E-03 | 5.78E-02 | 1.11E-01 | [3](http://toppgene.cchmc.org/showQueryTerms.jsp?userdata_id=41d53c5f-f68c-4e9f-8754-37ad37cd4269&feature=gop&row=15) | [14](http://toppgene.cchmc.org/showTermDetail.jsp?userdata_id=41d53c5f-f68c-4e9f-8754-37ad37cd4269&category=GeneOntologyBiologicalProcess&id=GO:0014002) |
| 17 | GO:0021537 | telencephalon development |  | 5.14E-05 | 7.02E-03 | 5.86E-02 | 1.21E-01 | [7](http://toppgene.cchmc.org/showQueryTerms.jsp?userdata_id=41d53c5f-f68c-4e9f-8754-37ad37cd4269&feature=gop&row=16) | [186](http://toppgene.cchmc.org/showTermDetail.jsp?userdata_id=41d53c5f-f68c-4e9f-8754-37ad37cd4269&category=GeneOntologyBiologicalProcess&id=GO:0021537) |
| 18 | GO:0071902 | positive regulation of protein serine/threonine kinase activity |  | 5.37E-05 | 7.02E-03 | 5.86E-02 | 1.26E-01 | [8](http://toppgene.cchmc.org/showQueryTerms.jsp?userdata_id=41d53c5f-f68c-4e9f-8754-37ad37cd4269&feature=gop&row=17) | [255](http://toppgene.cchmc.org/showTermDetail.jsp?userdata_id=41d53c5f-f68c-4e9f-8754-37ad37cd4269&category=GeneOntologyBiologicalProcess&id=GO:0071902) |
| 19 | GO:0050730 | regulation of peptidyl-tyrosine phosphorylation |  | 6.08E-05 | 7.54E-03 | 6.29E-02 | 1.43E-01 | [7](http://toppgene.cchmc.org/showQueryTerms.jsp?userdata_id=41d53c5f-f68c-4e9f-8754-37ad37cd4269&feature=gop&row=18) | [191](http://toppgene.cchmc.org/showTermDetail.jsp?userdata_id=41d53c5f-f68c-4e9f-8754-37ad37cd4269&category=GeneOntologyBiologicalProcess&id=GO:0050730) |
| 20 | GO:0043550 | regulation of lipid kinase activity |  | 6.51E-05 | 7.60E-03 | 6.34E-02 | 1.53E-01 | [4](http://toppgene.cchmc.org/showQueryTerms.jsp?userdata_id=41d53c5f-f68c-4e9f-8754-37ad37cd4269&feature=gop&row=19) | [42](http://toppgene.cchmc.org/showTermDetail.jsp?userdata_id=41d53c5f-f68c-4e9f-8754-37ad37cd4269&category=GeneOntologyBiologicalProcess&id=GO:0043550) |
| 21 | GO:0008285 | negative regulation of cell proliferation |  | 6.80E-05 | 7.60E-03 | 6.34E-02 | 1.60E-01 | [12](http://toppgene.cchmc.org/showQueryTerms.jsp?userdata_id=41d53c5f-f68c-4e9f-8754-37ad37cd4269&feature=gop&row=20) | [606](http://toppgene.cchmc.org/showTermDetail.jsp?userdata_id=41d53c5f-f68c-4e9f-8754-37ad37cd4269&category=GeneOntologyBiologicalProcess&id=GO:0008285) |
| 22 | GO:0033599 | regulation of mammary gland epithelial cell proliferation |  | 7.19E-05 | 7.60E-03 | 6.34E-02 | 1.69E-01 | [3](http://toppgene.cchmc.org/showQueryTerms.jsp?userdata_id=41d53c5f-f68c-4e9f-8754-37ad37cd4269&feature=gop&row=21) | [16](http://toppgene.cchmc.org/showTermDetail.jsp?userdata_id=41d53c5f-f68c-4e9f-8754-37ad37cd4269&category=GeneOntologyBiologicalProcess&id=GO:0033599) |
| 23 | GO:0032270 | positive regulation of cellular protein metabolic process |  | 7.42E-05 | 7.60E-03 | 6.34E-02 | 1.75E-01 | [15](http://toppgene.cchmc.org/showQueryTerms.jsp?userdata_id=41d53c5f-f68c-4e9f-8754-37ad37cd4269&feature=gop&row=22) | [921](http://toppgene.cchmc.org/showTermDetail.jsp?userdata_id=41d53c5f-f68c-4e9f-8754-37ad37cd4269&category=GeneOntologyBiologicalProcess&id=GO:0032270) |
| 24 | GO:0002768 | immune response-regulating cell surface receptor signaling pathway |  | 7.89E-05 | 7.60E-03 | 6.34E-02 | 1.86E-01 | [9](http://toppgene.cchmc.org/showQueryTerms.jsp?userdata_id=41d53c5f-f68c-4e9f-8754-37ad37cd4269&feature=gop&row=23) | [347](http://toppgene.cchmc.org/showTermDetail.jsp?userdata_id=41d53c5f-f68c-4e9f-8754-37ad37cd4269&category=GeneOntologyBiologicalProcess&id=GO:0002768) |
| 25 | GO:0043408 | regulation of MAPK cascade |  | 8.07E-05 | 7.60E-03 | 6.34E-02 | 1.90E-01 | [11](http://toppgene.cchmc.org/showQueryTerms.jsp?userdata_id=41d53c5f-f68c-4e9f-8754-37ad37cd4269&feature=gop&row=24) | [522](http://toppgene.cchmc.org/showTermDetail.jsp?userdata_id=41d53c5f-f68c-4e9f-8754-37ad37cd4269&category=GeneOntologyBiologicalProcess&id=GO:0043408) |
| 26 | GO:0046777 | protein autophosphorylation |  | 8.92E-05 | 7.73E-03 | 6.45E-02 | 2.10E-01 | [7](http://toppgene.cchmc.org/showQueryTerms.jsp?userdata_id=41d53c5f-f68c-4e9f-8754-37ad37cd4269&feature=gop&row=25) | [203](http://toppgene.cchmc.org/showTermDetail.jsp?userdata_id=41d53c5f-f68c-4e9f-8754-37ad37cd4269&category=GeneOntologyBiologicalProcess&id=GO:0046777) |
| 27 | GO:0043410 | positive regulation of MAPK cascade |  | 9.58E-05 | 7.73E-03 | 6.45E-02 | 2.26E-01 | [9](http://toppgene.cchmc.org/showQueryTerms.jsp?userdata_id=41d53c5f-f68c-4e9f-8754-37ad37cd4269&feature=gop&row=26) | [356](http://toppgene.cchmc.org/showTermDetail.jsp?userdata_id=41d53c5f-f68c-4e9f-8754-37ad37cd4269&category=GeneOntologyBiologicalProcess&id=GO:0043410) |
| 28 | GO:0043085 | positive regulation of catalytic activity |  | 9.74E-05 | 7.73E-03 | 6.45E-02 | 2.30E-01 | [16](http://toppgene.cchmc.org/showQueryTerms.jsp?userdata_id=41d53c5f-f68c-4e9f-8754-37ad37cd4269&feature=gop&row=27) | [1056](http://toppgene.cchmc.org/showTermDetail.jsp?userdata_id=41d53c5f-f68c-4e9f-8754-37ad37cd4269&category=GeneOntologyBiologicalProcess&id=GO:0043085) |
| 29 | GO:0048015 | phosphatidylinositol-mediated signaling |  | 9.77E-05 | 7.73E-03 | 6.45E-02 | 2.30E-01 | [7](http://toppgene.cchmc.org/showQueryTerms.jsp?userdata_id=41d53c5f-f68c-4e9f-8754-37ad37cd4269&feature=gop&row=28) | [206](http://toppgene.cchmc.org/showTermDetail.jsp?userdata_id=41d53c5f-f68c-4e9f-8754-37ad37cd4269&category=GeneOntologyBiologicalProcess&id=GO:0048015) |
| 30 | GO:0048017 | inositol lipid-mediated signaling |  | 1.01E-04 | 7.73E-03 | 6.45E-02 | 2.37E-01 | [7](http://toppgene.cchmc.org/showQueryTerms.jsp?userdata_id=41d53c5f-f68c-4e9f-8754-37ad37cd4269&feature=gop&row=29) | [207](http://toppgene.cchmc.org/showTermDetail.jsp?userdata_id=41d53c5f-f68c-4e9f-8754-37ad37cd4269&category=GeneOntologyBiologicalProcess&id=GO:0048017) |
| 31 | GO:0042177 | negative regulation of protein catabolic process |  | 1.02E-04 | 7.73E-03 | 6.45E-02 | 2.40E-01 | [4](http://toppgene.cchmc.org/showQueryTerms.jsp?userdata_id=41d53c5f-f68c-4e9f-8754-37ad37cd4269&feature=gop&row=30) | [47](http://toppgene.cchmc.org/showTermDetail.jsp?userdata_id=41d53c5f-f68c-4e9f-8754-37ad37cd4269&category=GeneOntologyBiologicalProcess&id=GO:0042177) |
| 32 | GO:0018108 | peptidyl-tyrosine phosphorylation |  | 1.06E-04 | 7.78E-03 | 6.49E-02 | 2.49E-01 | [8](http://toppgene.cchmc.org/showQueryTerms.jsp?userdata_id=41d53c5f-f68c-4e9f-8754-37ad37cd4269&feature=gop&row=31) | [281](http://toppgene.cchmc.org/showTermDetail.jsp?userdata_id=41d53c5f-f68c-4e9f-8754-37ad37cd4269&category=GeneOntologyBiologicalProcess&id=GO:0018108) |
| 33 | GO:0018212 | peptidyl-tyrosine modification |  | 1.11E-04 | 7.92E-03 | 6.61E-02 | 2.61E-01 | [8](http://toppgene.cchmc.org/showQueryTerms.jsp?userdata_id=41d53c5f-f68c-4e9f-8754-37ad37cd4269&feature=gop&row=32) | [283](http://toppgene.cchmc.org/showTermDetail.jsp?userdata_id=41d53c5f-f68c-4e9f-8754-37ad37cd4269&category=GeneOntologyBiologicalProcess&id=GO:0018212) |
| 34 | GO:0048708 | astrocyte differentiation |  | 1.30E-04 | 8.99E-03 | 7.50E-02 | 3.06E-01 | [4](http://toppgene.cchmc.org/showQueryTerms.jsp?userdata_id=41d53c5f-f68c-4e9f-8754-37ad37cd4269&feature=gop&row=33) | [50](http://toppgene.cchmc.org/showTermDetail.jsp?userdata_id=41d53c5f-f68c-4e9f-8754-37ad37cd4269&category=GeneOntologyBiologicalProcess&id=GO:0048708) |
| 35 | GO:0045860 | positive regulation of protein kinase activity |  | 1.35E-04 | 9.06E-03 | 7.56E-02 | 3.17E-01 | [10](http://toppgene.cchmc.org/showQueryTerms.jsp?userdata_id=41d53c5f-f68c-4e9f-8754-37ad37cd4269&feature=gop&row=34) | [460](http://toppgene.cchmc.org/showTermDetail.jsp?userdata_id=41d53c5f-f68c-4e9f-8754-37ad37cd4269&category=GeneOntologyBiologicalProcess&id=GO:0045860) |
| 36 | GO:0007265 | Ras protein signal transduction |  | 1.42E-04 | 9.21E-03 | 7.68E-02 | 3.34E-01 | [10](http://toppgene.cchmc.org/showQueryTerms.jsp?userdata_id=41d53c5f-f68c-4e9f-8754-37ad37cd4269&feature=gop&row=35) | [463](http://toppgene.cchmc.org/showTermDetail.jsp?userdata_id=41d53c5f-f68c-4e9f-8754-37ad37cd4269&category=GeneOntologyBiologicalProcess&id=GO:0007265) |
| 37 | GO:0048584 | positive regulation of response to stimulus |  | 1.45E-04 | 9.21E-03 | 7.68E-02 | 3.41E-01 | [19](http://toppgene.cchmc.org/showQueryTerms.jsp?userdata_id=41d53c5f-f68c-4e9f-8754-37ad37cd4269&feature=gop&row=36) | [1455](http://toppgene.cchmc.org/showTermDetail.jsp?userdata_id=41d53c5f-f68c-4e9f-8754-37ad37cd4269&category=GeneOntologyBiologicalProcess&id=GO:0048584) |
| 38 | GO:0009967 | positive regulation of signal transduction |  | 1.71E-04 | 1.04E-02 | 8.68E-02 | 4.02E-01 | [15](http://toppgene.cchmc.org/showQueryTerms.jsp?userdata_id=41d53c5f-f68c-4e9f-8754-37ad37cd4269&feature=gop&row=37) | [993](http://toppgene.cchmc.org/showTermDetail.jsp?userdata_id=41d53c5f-f68c-4e9f-8754-37ad37cd4269&category=GeneOntologyBiologicalProcess&id=GO:0009967) |
| 39 | GO:0007173 | epidermal growth factor receptor signaling pathway |  | 1.78E-04 | 1.04E-02 | 8.68E-02 | 4.20E-01 | [7](http://toppgene.cchmc.org/showQueryTerms.jsp?userdata_id=41d53c5f-f68c-4e9f-8754-37ad37cd4269&feature=gop&row=38) | [227](http://toppgene.cchmc.org/showTermDetail.jsp?userdata_id=41d53c5f-f68c-4e9f-8754-37ad37cd4269&category=GeneOntologyBiologicalProcess&id=GO:0007173) |
| 40 | GO:0048103 | somatic stem cell division |  | 1.93E-04 | 1.04E-02 | 8.68E-02 | 4.55E-01 | [3](http://toppgene.cchmc.org/showQueryTerms.jsp?userdata_id=41d53c5f-f68c-4e9f-8754-37ad37cd4269&feature=gop&row=39) | [22](http://toppgene.cchmc.org/showTermDetail.jsp?userdata_id=41d53c5f-f68c-4e9f-8754-37ad37cd4269&category=GeneOntologyBiologicalProcess&id=GO:0048103) |
| 41 | GO:0048863 | stem cell differentiation |  | 1.94E-04 | 1.04E-02 | 8.68E-02 | 4.57E-01 | [8](http://toppgene.cchmc.org/showQueryTerms.jsp?userdata_id=41d53c5f-f68c-4e9f-8754-37ad37cd4269&feature=gop&row=40) | [307](http://toppgene.cchmc.org/showTermDetail.jsp?userdata_id=41d53c5f-f68c-4e9f-8754-37ad37cd4269&category=GeneOntologyBiologicalProcess&id=GO:0048863) |
| 42 | GO:0050680 | negative regulation of epithelial cell proliferation |  | 1.97E-04 | 1.04E-02 | 8.68E-02 | 4.64E-01 | [5](http://toppgene.cchmc.org/showQueryTerms.jsp?userdata_id=41d53c5f-f68c-4e9f-8754-37ad37cd4269&feature=gop&row=41) | [103](http://toppgene.cchmc.org/showTermDetail.jsp?userdata_id=41d53c5f-f68c-4e9f-8754-37ad37cd4269&category=GeneOntologyBiologicalProcess&id=GO:0050680) |
| 43 | GO:0038127 | ERBB signaling pathway |  | 1.99E-04 | 1.04E-02 | 8.68E-02 | 4.67E-01 | [7](http://toppgene.cchmc.org/showQueryTerms.jsp?userdata_id=41d53c5f-f68c-4e9f-8754-37ad37cd4269&feature=gop&row=42) | [231](http://toppgene.cchmc.org/showTermDetail.jsp?userdata_id=41d53c5f-f68c-4e9f-8754-37ad37cd4269&category=GeneOntologyBiologicalProcess&id=GO:0038127) |
| 44 | GO:0032870 | cellular response to hormone stimulus |  | 2.00E-04 | 1.04E-02 | 8.68E-02 | 4.71E-01 | [10](http://toppgene.cchmc.org/showQueryTerms.jsp?userdata_id=41d53c5f-f68c-4e9f-8754-37ad37cd4269&feature=gop&row=43) | [483](http://toppgene.cchmc.org/showTermDetail.jsp?userdata_id=41d53c5f-f68c-4e9f-8754-37ad37cd4269&category=GeneOntologyBiologicalProcess&id=GO:0032870) |
| 45 | GO:0051247 | positive regulation of protein metabolic process |  | 2.01E-04 | 1.04E-02 | 8.68E-02 | 4.74E-01 | [15](http://toppgene.cchmc.org/showQueryTerms.jsp?userdata_id=41d53c5f-f68c-4e9f-8754-37ad37cd4269&feature=gop&row=44) | [1008](http://toppgene.cchmc.org/showTermDetail.jsp?userdata_id=41d53c5f-f68c-4e9f-8754-37ad37cd4269&category=GeneOntologyBiologicalProcess&id=GO:0051247) |
| 46 | GO:0033674 | positive regulation of kinase activity |  | 2.03E-04 | 1.04E-02 | 8.68E-02 | 4.79E-01 | [10](http://toppgene.cchmc.org/showQueryTerms.jsp?userdata_id=41d53c5f-f68c-4e9f-8754-37ad37cd4269&feature=gop&row=45) | [484](http://toppgene.cchmc.org/showTermDetail.jsp?userdata_id=41d53c5f-f68c-4e9f-8754-37ad37cd4269&category=GeneOntologyBiologicalProcess&id=GO:0033674) |
| 47 | GO:0071495 | cellular response to endogenous stimulus |  | 2.26E-04 | 1.13E-02 | 9.43E-02 | 5.32E-01 | [14](http://toppgene.cchmc.org/showQueryTerms.jsp?userdata_id=41d53c5f-f68c-4e9f-8754-37ad37cd4269&feature=gop&row=46) | [905](http://toppgene.cchmc.org/showTermDetail.jsp?userdata_id=41d53c5f-f68c-4e9f-8754-37ad37cd4269&category=GeneOntologyBiologicalProcess&id=GO:0071495) |
| 48 | GO:0010627 | regulation of intracellular protein kinase cascade |  | 2.32E-04 | 1.13E-02 | 9.44E-02 | 5.45E-01 | [13](http://toppgene.cchmc.org/showQueryTerms.jsp?userdata_id=41d53c5f-f68c-4e9f-8754-37ad37cd4269&feature=gop&row=47) | [797](http://toppgene.cchmc.org/showTermDetail.jsp?userdata_id=41d53c5f-f68c-4e9f-8754-37ad37cd4269&category=GeneOntologyBiologicalProcess&id=GO:0010627) |
| 49 | GO:0007264 | small GTPase mediated signal transduction |  | 2.36E-04 | 1.13E-02 | 9.44E-02 | 5.55E-01 | [12](http://toppgene.cchmc.org/showQueryTerms.jsp?userdata_id=41d53c5f-f68c-4e9f-8754-37ad37cd4269&feature=gop&row=48) | [692](http://toppgene.cchmc.org/showTermDetail.jsp?userdata_id=41d53c5f-f68c-4e9f-8754-37ad37cd4269&category=GeneOntologyBiologicalProcess&id=GO:0007264) |
| 50 | GO:0051347 | positive regulation of transferase activity |  | 2.47E-04 | 1.15E-02 | 9.60E-02 | 5.83E-01 | [10](http://toppgene.cchmc.org/showQueryTerms.jsp?userdata_id=41d53c5f-f68c-4e9f-8754-37ad37cd4269&feature=gop&row=49) | [496](http://toppgene.cchmc.org/showTermDetail.jsp?userdata_id=41d53c5f-f68c-4e9f-8754-37ad37cd4269&category=GeneOntologyBiologicalProcess&id=GO:0051347) |

| Pathway | ID | Name | Source | pValue | FDR B&H | FDR B&Y | Bonferroni | Genes from Input | Genes in Annotation |
| --- | --- | --- | --- | --- | --- | --- | --- | --- | --- |
| 1 | 672458 | Signaling Pathways in Glioblastoma | [BioSystems: WikiPathways](http://www.ncbi.nlm.nih.gov/biosystems/672458) | 1.87E-08 | 1.27E-05 | 9.04E-05 | 1.27E-05 | [8](http://toppgene.cchmc.org/showQueryTerms.jsp?userdata_id=41d53c5f-f68c-4e9f-8754-37ad37cd4269&feature=pt&row=0) | [80](http://toppgene.cchmc.org/showTermDetail.jsp?userdata_id=41d53c5f-f68c-4e9f-8754-37ad37cd4269&category=Pathway&id=672458) |
| 2 | 692234 | PI3K-Akt signaling pathway | [BioSystems: KEGG](http://www.ncbi.nlm.nih.gov/biosystems/692234) | 6.38E-07 | 1.89E-04 | 1.34E-03 | 4.35E-04 | [12](http://toppgene.cchmc.org/showQueryTerms.jsp?userdata_id=41d53c5f-f68c-4e9f-8754-37ad37cd4269&feature=pt&row=1) | [346](http://toppgene.cchmc.org/showTermDetail.jsp?userdata_id=41d53c5f-f68c-4e9f-8754-37ad37cd4269&category=Pathway&id=692234) |
| 3 | 83119 | Non-small cell lung cancer | [BioSystems: KEGG](http://www.ncbi.nlm.nih.gov/biosystems/83119) | 8.32E-07 | 1.89E-04 | 1.34E-03 | 5.68E-04 | [6](http://toppgene.cchmc.org/showQueryTerms.jsp?userdata_id=41d53c5f-f68c-4e9f-8754-37ad37cd4269&feature=pt&row=2) | [56](http://toppgene.cchmc.org/showTermDetail.jsp?userdata_id=41d53c5f-f68c-4e9f-8754-37ad37cd4269&category=Pathway&id=83119) |
| 4 | 83110 | Glioma | [BioSystems: KEGG](http://www.ncbi.nlm.nih.gov/biosystems/83110) | 2.03E-06 | 3.04E-04 | 2.16E-03 | 1.39E-03 | [6](http://toppgene.cchmc.org/showQueryTerms.jsp?userdata_id=41d53c5f-f68c-4e9f-8754-37ad37cd4269&feature=pt&row=3) | [65](http://toppgene.cchmc.org/showTermDetail.jsp?userdata_id=41d53c5f-f68c-4e9f-8754-37ad37cd4269&category=Pathway&id=83110) |
| 5 | 83108 | Pancreatic cancer | [BioSystems: KEGG](http://www.ncbi.nlm.nih.gov/biosystems/83108) | 2.23E-06 | 3.04E-04 | 2.16E-03 | 1.52E-03 | [6](http://toppgene.cchmc.org/showQueryTerms.jsp?userdata_id=41d53c5f-f68c-4e9f-8754-37ad37cd4269&feature=pt&row=4) | [66](http://toppgene.cchmc.org/showTermDetail.jsp?userdata_id=41d53c5f-f68c-4e9f-8754-37ad37cd4269&category=Pathway&id=83108) |
| 6 | 83114 | Melanoma | [BioSystems: KEGG](http://www.ncbi.nlm.nih.gov/biosystems/83114) | 3.43E-06 | 3.90E-04 | 2.77E-03 | 2.34E-03 | [6](http://toppgene.cchmc.org/showQueryTerms.jsp?userdata_id=41d53c5f-f68c-4e9f-8754-37ad37cd4269&feature=pt&row=5) | [71](http://toppgene.cchmc.org/showTermDetail.jsp?userdata_id=41d53c5f-f68c-4e9f-8754-37ad37cd4269&category=Pathway&id=83114) |
| 7 | 83111 | Prostate cancer | [BioSystems: KEGG](http://www.ncbi.nlm.nih.gov/biosystems/83111) | 1.28E-05 | 1.25E-03 | 8.87E-03 | 8.74E-03 | [6](http://toppgene.cchmc.org/showQueryTerms.jsp?userdata_id=41d53c5f-f68c-4e9f-8754-37ad37cd4269&feature=pt&row=6) | [89](http://toppgene.cchmc.org/showTermDetail.jsp?userdata_id=41d53c5f-f68c-4e9f-8754-37ad37cd4269&category=Pathway&id=83111) |
| 8 | 83089 | Regulation of actin cytoskeleton | [BioSystems: KEGG](http://www.ncbi.nlm.nih.gov/biosystems/83089) | 3.44E-05 | 2.93E-03 | 2.08E-02 | 2.35E-02 | [8](http://toppgene.cchmc.org/showQueryTerms.jsp?userdata_id=41d53c5f-f68c-4e9f-8754-37ad37cd4269&feature=pt&row=7) | [215](http://toppgene.cchmc.org/showTermDetail.jsp?userdata_id=41d53c5f-f68c-4e9f-8754-37ad37cd4269&category=Pathway&id=83089) |
| 9 | 868085 | Ras signaling pathway | [BioSystems: KEGG](http://www.ncbi.nlm.nih.gov/biosystems/868085) | 5.06E-05 | 3.84E-03 | 2.73E-02 | 3.45E-02 | [8](http://toppgene.cchmc.org/showQueryTerms.jsp?userdata_id=41d53c5f-f68c-4e9f-8754-37ad37cd4269&feature=pt&row=8) | [227](http://toppgene.cchmc.org/showTermDetail.jsp?userdata_id=41d53c5f-f68c-4e9f-8754-37ad37cd4269&category=Pathway&id=868085) |
| 10 | 83116 | Chronic myeloid leukemia | [BioSystems: KEGG](http://www.ncbi.nlm.nih.gov/biosystems/83116) | 6.58E-05 | 4.09E-03 | 2.91E-02 | 4.49E-02 | [5](http://toppgene.cchmc.org/showQueryTerms.jsp?userdata_id=41d53c5f-f68c-4e9f-8754-37ad37cd4269&feature=pt&row=9) | [73](http://toppgene.cchmc.org/showTermDetail.jsp?userdata_id=41d53c5f-f68c-4e9f-8754-37ad37cd4269&category=Pathway&id=83116) |
| 11 | 83115 | Bladder cancer | [BioSystems: KEGG](http://www.ncbi.nlm.nih.gov/biosystems/83115) | 6.87E-05 | 4.09E-03 | 2.91E-02 | 4.69E-02 | [4](http://toppgene.cchmc.org/showQueryTerms.jsp?userdata_id=41d53c5f-f68c-4e9f-8754-37ad37cd4269&feature=pt&row=10) | [38](http://toppgene.cchmc.org/showTermDetail.jsp?userdata_id=41d53c5f-f68c-4e9f-8754-37ad37cd4269&category=Pathway&id=83115) |
| 12 | 106337 | Signaling by EGFR | [BioSystems: REACTOME](http://www.ncbi.nlm.nih.gov/biosystems/106337) | 7.26E-05 | 4.09E-03 | 2.91E-02 | 4.95E-02 | [7](http://toppgene.cchmc.org/showQueryTerms.jsp?userdata_id=41d53c5f-f68c-4e9f-8754-37ad37cd4269&feature=pt&row=11) | [176](http://toppgene.cchmc.org/showTermDetail.jsp?userdata_id=41d53c5f-f68c-4e9f-8754-37ad37cd4269&category=Pathway&id=106337) |
| 13 | 530768 | Signaling by EGFR in Cancer | [BioSystems: REACTOME](http://www.ncbi.nlm.nih.gov/biosystems/530768) | 7.80E-05 | 4.09E-03 | 2.91E-02 | 5.32E-02 | [7](http://toppgene.cchmc.org/showQueryTerms.jsp?userdata_id=41d53c5f-f68c-4e9f-8754-37ad37cd4269&feature=pt&row=12) | [178](http://toppgene.cchmc.org/showTermDetail.jsp?userdata_id=41d53c5f-f68c-4e9f-8754-37ad37cd4269&category=Pathway&id=530768) |
| 14 | 83105 | Pathways in cancer | [BioSystems: KEGG](http://www.ncbi.nlm.nih.gov/biosystems/83105) | 1.13E-04 | 5.52E-03 | 3.92E-02 | 7.72E-02 | [9](http://toppgene.cchmc.org/showQueryTerms.jsp?userdata_id=41d53c5f-f68c-4e9f-8754-37ad37cd4269&feature=pt&row=13) | [327](http://toppgene.cchmc.org/showTermDetail.jsp?userdata_id=41d53c5f-f68c-4e9f-8754-37ad37cd4269&category=Pathway&id=83105) |
| 15 | 119304 | Progesterone-mediated oocyte maturation | [BioSystems: KEGG](http://www.ncbi.nlm.nih.gov/biosystems/119304) | 1.44E-04 | 6.10E-03 | 4.33E-02 | 9.81E-02 | [5](http://toppgene.cchmc.org/showQueryTerms.jsp?userdata_id=41d53c5f-f68c-4e9f-8754-37ad37cd4269&feature=pt&row=14) | [86](http://toppgene.cchmc.org/showTermDetail.jsp?userdata_id=41d53c5f-f68c-4e9f-8754-37ad37cd4269&category=Pathway&id=119304) |
| 16 | 477120 | Signaling by SCF-KIT | [BioSystems: REACTOME](http://www.ncbi.nlm.nih.gov/biosystems/477120) | 1.52E-04 | 6.10E-03 | 4.33E-02 | 1.03E-01 | [6](http://toppgene.cchmc.org/showQueryTerms.jsp?userdata_id=41d53c5f-f68c-4e9f-8754-37ad37cd4269&feature=pt&row=15) | [138](http://toppgene.cchmc.org/showTermDetail.jsp?userdata_id=41d53c5f-f68c-4e9f-8754-37ad37cd4269&category=Pathway&id=477120) |
| 17 | 685535 | Constitutive PI3K/AKT Signaling in Cancer | [BioSystems: REACTOME](http://www.ncbi.nlm.nih.gov/biosystems/685535) | 1.52E-04 | 6.10E-03 | 4.33E-02 | 1.04E-01 | [5](http://toppgene.cchmc.org/showQueryTerms.jsp?userdata_id=41d53c5f-f68c-4e9f-8754-37ad37cd4269&feature=pt&row=16) | [87](http://toppgene.cchmc.org/showTermDetail.jsp?userdata_id=41d53c5f-f68c-4e9f-8754-37ad37cd4269&category=Pathway&id=685535) |
| 18 | 160957 | Downstream signaling of activated FGFR | [BioSystems: REACTOME](http://www.ncbi.nlm.nih.gov/biosystems/160957) | 1.91E-04 | 6.53E-03 | 4.64E-02 | 1.30E-01 | [6](http://toppgene.cchmc.org/showQueryTerms.jsp?userdata_id=41d53c5f-f68c-4e9f-8754-37ad37cd4269&feature=pt&row=17) | [144](http://toppgene.cchmc.org/showTermDetail.jsp?userdata_id=41d53c5f-f68c-4e9f-8754-37ad37cd4269&category=Pathway&id=160957) |
| 19 | 198874 | Regulation of Actin Cytoskeleton | [BioSystems: WikiPathways](http://www.ncbi.nlm.nih.gov/biosystems/198874) | 2.14E-04 | 6.53E-03 | 4.64E-02 | 1.46E-01 | [6](http://toppgene.cchmc.org/showQueryTerms.jsp?userdata_id=41d53c5f-f68c-4e9f-8754-37ad37cd4269&feature=pt&row=18) | [147](http://toppgene.cchmc.org/showTermDetail.jsp?userdata_id=41d53c5f-f68c-4e9f-8754-37ad37cd4269&category=Pathway&id=198874) |
| 20 | 530741 | Signaling by ERBB4 | [BioSystems: REACTOME](http://www.ncbi.nlm.nih.gov/biosystems/530741) | 2.30E-04 | 6.53E-03 | 4.64E-02 | 1.57E-01 | [6](http://toppgene.cchmc.org/showQueryTerms.jsp?userdata_id=41d53c5f-f68c-4e9f-8754-37ad37cd4269&feature=pt&row=19) | [149](http://toppgene.cchmc.org/showTermDetail.jsp?userdata_id=41d53c5f-f68c-4e9f-8754-37ad37cd4269&category=Pathway&id=530741) |
| 21 | 868086 | Rap1 signaling pathway | [BioSystems: KEGG](http://www.ncbi.nlm.nih.gov/biosystems/868086) | 2.38E-04 | 6.53E-03 | 4.64E-02 | 1.62E-01 | [7](http://toppgene.cchmc.org/showQueryTerms.jsp?userdata_id=41d53c5f-f68c-4e9f-8754-37ad37cd4269&feature=pt&row=20) | [213](http://toppgene.cchmc.org/showTermDetail.jsp?userdata_id=41d53c5f-f68c-4e9f-8754-37ad37cd4269&category=Pathway&id=868086) |
| 22 | 219801 | Integrated Breast Cancer Pathway | [BioSystems: WikiPathways](http://www.ncbi.nlm.nih.gov/biosystems/219801) | 2.75E-04 | 6.53E-03 | 4.64E-02 | 1.88E-01 | [6](http://toppgene.cchmc.org/showQueryTerms.jsp?userdata_id=41d53c5f-f68c-4e9f-8754-37ad37cd4269&feature=pt&row=21) | [154](http://toppgene.cchmc.org/showTermDetail.jsp?userdata_id=41d53c5f-f68c-4e9f-8754-37ad37cd4269&category=Pathway&id=219801) |
| 23 | 685534 | PI3K/AKT Signaling in Cancer | [BioSystems: REACTOME](http://www.ncbi.nlm.nih.gov/biosystems/685534) | 2.79E-04 | 6.53E-03 | 4.64E-02 | 1.90E-01 | [5](http://toppgene.cchmc.org/showQueryTerms.jsp?userdata_id=41d53c5f-f68c-4e9f-8754-37ad37cd4269&feature=pt&row=22) | [99](http://toppgene.cchmc.org/showTermDetail.jsp?userdata_id=41d53c5f-f68c-4e9f-8754-37ad37cd4269&category=Pathway&id=685534) |
| 24 | 530737 | PI3K events in ERBB2 signaling | [BioSystems: REACTOME](http://www.ncbi.nlm.nih.gov/biosystems/530737) | 2.79E-04 | 6.53E-03 | 4.64E-02 | 1.90E-01 | [5](http://toppgene.cchmc.org/showQueryTerms.jsp?userdata_id=41d53c5f-f68c-4e9f-8754-37ad37cd4269&feature=pt&row=23) | [99](http://toppgene.cchmc.org/showTermDetail.jsp?userdata_id=41d53c5f-f68c-4e9f-8754-37ad37cd4269&category=Pathway&id=530737) |
| 25 | 366239 | PIP3 activates AKT signaling | [BioSystems: REACTOME](http://www.ncbi.nlm.nih.gov/biosystems/366239) | 2.79E-04 | 6.53E-03 | 4.64E-02 | 1.90E-01 | [5](http://toppgene.cchmc.org/showQueryTerms.jsp?userdata_id=41d53c5f-f68c-4e9f-8754-37ad37cd4269&feature=pt&row=24) | [99](http://toppgene.cchmc.org/showTermDetail.jsp?userdata_id=41d53c5f-f68c-4e9f-8754-37ad37cd4269&category=Pathway&id=366239) |
| 26 | 530743 | PI3K events in ERBB4 signaling | [BioSystems: REACTOME](http://www.ncbi.nlm.nih.gov/biosystems/530743) | 2.79E-04 | 6.53E-03 | 4.64E-02 | 1.90E-01 | [5](http://toppgene.cchmc.org/showQueryTerms.jsp?userdata_id=41d53c5f-f68c-4e9f-8754-37ad37cd4269&feature=pt&row=25) | [99](http://toppgene.cchmc.org/showTermDetail.jsp?userdata_id=41d53c5f-f68c-4e9f-8754-37ad37cd4269&category=Pathway&id=530743) |
| 27 | 160961 | PI-3K cascade | [BioSystems: REACTOME](http://www.ncbi.nlm.nih.gov/biosystems/160961) | 2.79E-04 | 6.53E-03 | 4.64E-02 | 1.90E-01 | [5](http://toppgene.cchmc.org/showQueryTerms.jsp?userdata_id=41d53c5f-f68c-4e9f-8754-37ad37cd4269&feature=pt&row=26) | [99](http://toppgene.cchmc.org/showTermDetail.jsp?userdata_id=41d53c5f-f68c-4e9f-8754-37ad37cd4269&category=Pathway&id=160961) |
| 28 | 138006 | ATF-2 transcription factor network | [BioSystems: Pathway Interaction Database](http://www.ncbi.nlm.nih.gov/biosystems/138006) | 2.95E-04 | 6.53E-03 | 4.64E-02 | 2.01E-01 | [4](http://toppgene.cchmc.org/showQueryTerms.jsp?userdata_id=41d53c5f-f68c-4e9f-8754-37ad37cd4269&feature=pt&row=27) | [55](http://toppgene.cchmc.org/showTermDetail.jsp?userdata_id=41d53c5f-f68c-4e9f-8754-37ad37cd4269&category=Pathway&id=138006) |
| 29 | 106385 | Downstream signal transduction | [BioSystems: REACTOME](http://www.ncbi.nlm.nih.gov/biosystems/106385) | 2.95E-04 | 6.53E-03 | 4.64E-02 | 2.01E-01 | [6](http://toppgene.cchmc.org/showQueryTerms.jsp?userdata_id=41d53c5f-f68c-4e9f-8754-37ad37cd4269&feature=pt&row=28) | [156](http://toppgene.cchmc.org/showTermDetail.jsp?userdata_id=41d53c5f-f68c-4e9f-8754-37ad37cd4269&category=Pathway&id=106385) |
| 30 | M17770 | Cyclins and Cell Cycle Regulation | [MSigDB C2: BioCarta](http://www.broadinstitute.org/gsea/msigdb/cards/BIOCARTA_CELLCYCLE_PATHWAY.html) | 3.15E-04 | 6.53E-03 | 4.64E-02 | 2.15E-01 | [3](http://toppgene.cchmc.org/showQueryTerms.jsp?userdata_id=41d53c5f-f68c-4e9f-8754-37ad37cd4269&feature=pt&row=29) | [23](http://toppgene.cchmc.org/showTermDetail.jsp?userdata_id=41d53c5f-f68c-4e9f-8754-37ad37cd4269&category=Pathway&id=M17770) |
| 31 | 106343 | Signaling by FGFR | [BioSystems: REACTOME](http://www.ncbi.nlm.nih.gov/biosystems/106343) | 3.16E-04 | 6.53E-03 | 4.64E-02 | 2.16E-01 | [6](http://toppgene.cchmc.org/showQueryTerms.jsp?userdata_id=41d53c5f-f68c-4e9f-8754-37ad37cd4269&feature=pt&row=30) | [158](http://toppgene.cchmc.org/showTermDetail.jsp?userdata_id=41d53c5f-f68c-4e9f-8754-37ad37cd4269&category=Pathway&id=106343) |
| 32 | 106474 | PI3K/AKT activation | [BioSystems: REACTOME](http://www.ncbi.nlm.nih.gov/biosystems/106474) | 3.20E-04 | 6.53E-03 | 4.64E-02 | 2.18E-01 | [5](http://toppgene.cchmc.org/showQueryTerms.jsp?userdata_id=41d53c5f-f68c-4e9f-8754-37ad37cd4269&feature=pt&row=31) | [102](http://toppgene.cchmc.org/showTermDetail.jsp?userdata_id=41d53c5f-f68c-4e9f-8754-37ad37cd4269&category=Pathway&id=106474) |
| 33 | 530734 | Signaling by ERBB2 | [BioSystems: REACTOME](http://www.ncbi.nlm.nih.gov/biosystems/530734) | 3.27E-04 | 6.53E-03 | 4.64E-02 | 2.23E-01 | [6](http://toppgene.cchmc.org/showQueryTerms.jsp?userdata_id=41d53c5f-f68c-4e9f-8754-37ad37cd4269&feature=pt&row=32) | [159](http://toppgene.cchmc.org/showTermDetail.jsp?userdata_id=41d53c5f-f68c-4e9f-8754-37ad37cd4269&category=Pathway&id=530734) |
| 34 | 685550 | DAP12 signaling | [BioSystems: REACTOME](http://www.ncbi.nlm.nih.gov/biosystems/685550) | 3.27E-04 | 6.53E-03 | 4.64E-02 | 2.23E-01 | [6](http://toppgene.cchmc.org/showQueryTerms.jsp?userdata_id=41d53c5f-f68c-4e9f-8754-37ad37cd4269&feature=pt&row=33) | [159](http://toppgene.cchmc.org/showTermDetail.jsp?userdata_id=41d53c5f-f68c-4e9f-8754-37ad37cd4269&category=Pathway&id=685550) |
| 35 | 106341 | GAB1 signalosome | [BioSystems: REACTOME](http://www.ncbi.nlm.nih.gov/biosystems/106341) | 3.35E-04 | 6.53E-03 | 4.64E-02 | 2.29E-01 | [5](http://toppgene.cchmc.org/showQueryTerms.jsp?userdata_id=41d53c5f-f68c-4e9f-8754-37ad37cd4269&feature=pt&row=34) | [103](http://toppgene.cchmc.org/showTermDetail.jsp?userdata_id=41d53c5f-f68c-4e9f-8754-37ad37cd4269&category=Pathway&id=106341) |
| 36 | 576250 | Downstream Signaling Events Of B Cell Receptor (BCR) | [BioSystems: REACTOME](http://www.ncbi.nlm.nih.gov/biosystems/576250) | 4.82E-04 | 9.14E-03 | 6.49E-02 | 3.29E-01 | [6](http://toppgene.cchmc.org/showQueryTerms.jsp?userdata_id=41d53c5f-f68c-4e9f-8754-37ad37cd4269&feature=pt&row=35) | [171](http://toppgene.cchmc.org/showTermDetail.jsp?userdata_id=41d53c5f-f68c-4e9f-8754-37ad37cd4269&category=Pathway&id=576250) |
| 37 | 645274 | Signaling by FGFR in disease | [BioSystems: REACTOME](http://www.ncbi.nlm.nih.gov/biosystems/645274) | 5.13E-04 | 9.45E-03 | 6.72E-02 | 3.50E-01 | [6](http://toppgene.cchmc.org/showQueryTerms.jsp?userdata_id=41d53c5f-f68c-4e9f-8754-37ad37cd4269&feature=pt&row=36) | [173](http://toppgene.cchmc.org/showTermDetail.jsp?userdata_id=41d53c5f-f68c-4e9f-8754-37ad37cd4269&category=Pathway&id=645274) |
| 38 | M648 | Cell Cycle: G1/S Check Point | [MSigDB C2: BioCarta](http://www.broadinstitute.org/gsea/msigdb/cards/BIOCARTA_G1_PATHWAY.html) | 5.70E-04 | 1.02E-02 | 7.27E-02 | 3.89E-01 | [3](http://toppgene.cchmc.org/showQueryTerms.jsp?userdata_id=41d53c5f-f68c-4e9f-8754-37ad37cd4269&feature=pt&row=37) | [28](http://toppgene.cchmc.org/showTermDetail.jsp?userdata_id=41d53c5f-f68c-4e9f-8754-37ad37cd4269&category=Pathway&id=M648) |
| 39 | 106384 | Signaling by PDGF | [BioSystems: REACTOME](http://www.ncbi.nlm.nih.gov/biosystems/106384) | 6.14E-04 | 1.05E-02 | 7.44E-02 | 4.19E-01 | [6](http://toppgene.cchmc.org/showQueryTerms.jsp?userdata_id=41d53c5f-f68c-4e9f-8754-37ad37cd4269&feature=pt&row=38) | [179](http://toppgene.cchmc.org/showTermDetail.jsp?userdata_id=41d53c5f-f68c-4e9f-8754-37ad37cd4269&category=Pathway&id=106384) |
| 40 | 685549 | DAP12 interactions | [BioSystems: REACTOME](http://www.ncbi.nlm.nih.gov/biosystems/685549) | 6.14E-04 | 1.05E-02 | 7.44E-02 | 4.19E-01 | [6](http://toppgene.cchmc.org/showQueryTerms.jsp?userdata_id=41d53c5f-f68c-4e9f-8754-37ad37cd4269&feature=pt&row=39) | [179](http://toppgene.cchmc.org/showTermDetail.jsp?userdata_id=41d53c5f-f68c-4e9f-8754-37ad37cd4269&category=Pathway&id=685549) |
| 41 | 198805 | G1 to S cell cycle control | [BioSystems: WikiPathways](http://www.ncbi.nlm.nih.gov/biosystems/198805) | 6.65E-04 | 1.11E-02 | 7.85E-02 | 4.53E-01 | [4](http://toppgene.cchmc.org/showQueryTerms.jsp?userdata_id=41d53c5f-f68c-4e9f-8754-37ad37cd4269&feature=pt&row=40) | [68](http://toppgene.cchmc.org/showTermDetail.jsp?userdata_id=41d53c5f-f68c-4e9f-8754-37ad37cd4269&category=Pathway&id=198805) |
| 42 | 833829 | Role of LAT2/NTAL/LAB on calcium mobilization | [BioSystems: REACTOME](http://www.ncbi.nlm.nih.gov/biosystems/833829) | 7.84E-04 | 1.27E-02 | 9.04E-02 | 5.35E-01 | [5](http://toppgene.cchmc.org/showQueryTerms.jsp?userdata_id=41d53c5f-f68c-4e9f-8754-37ad37cd4269&feature=pt&row=41) | [124](http://toppgene.cchmc.org/showTermDetail.jsp?userdata_id=41d53c5f-f68c-4e9f-8754-37ad37cd4269&category=Pathway&id=833829) |
| 43 | 814182 | Prolactin signaling pathway | [BioSystems: KEGG](http://www.ncbi.nlm.nih.gov/biosystems/814182) | 8.25E-04 | 1.29E-02 | 9.14E-02 | 5.63E-01 | [4](http://toppgene.cchmc.org/showQueryTerms.jsp?userdata_id=41d53c5f-f68c-4e9f-8754-37ad37cd4269&feature=pt&row=42) | [72](http://toppgene.cchmc.org/showTermDetail.jsp?userdata_id=41d53c5f-f68c-4e9f-8754-37ad37cd4269&category=Pathway&id=814182) |
| 44 | 373901 | HTLV-I infection | [BioSystems: KEGG](http://www.ncbi.nlm.nih.gov/biosystems/373901) | 8.44E-04 | 1.29E-02 | 9.14E-02 | 5.76E-01 | [7](http://toppgene.cchmc.org/showQueryTerms.jsp?userdata_id=41d53c5f-f68c-4e9f-8754-37ad37cd4269&feature=pt&row=43) | [263](http://toppgene.cchmc.org/showTermDetail.jsp?userdata_id=41d53c5f-f68c-4e9f-8754-37ad37cd4269&category=Pathway&id=373901) |
| 45 | 905992 | Oncogene Induced Senescence | [BioSystems: REACTOME](http://www.ncbi.nlm.nih.gov/biosystems/905992) | 8.49E-04 | 1.29E-02 | 9.14E-02 | 5.79E-01 | [3](http://toppgene.cchmc.org/showQueryTerms.jsp?userdata_id=41d53c5f-f68c-4e9f-8754-37ad37cd4269&feature=pt&row=44) | [32](http://toppgene.cchmc.org/showTermDetail.jsp?userdata_id=41d53c5f-f68c-4e9f-8754-37ad37cd4269&category=Pathway&id=905992) |
| 46 | 833825 | Fc epsilon receptor (FCERI) signaling | [BioSystems: REACTOME](http://www.ncbi.nlm.nih.gov/biosystems/833825) | 9.36E-04 | 1.36E-02 | 9.65E-02 | 6.38E-01 | [6](http://toppgene.cchmc.org/showQueryTerms.jsp?userdata_id=41d53c5f-f68c-4e9f-8754-37ad37cd4269&feature=pt&row=45) | [194](http://toppgene.cchmc.org/showTermDetail.jsp?userdata_id=41d53c5f-f68c-4e9f-8754-37ad37cd4269&category=Pathway&id=833825) |
| 47 | 711360 | Integrated Pancreatic Cancer Pathway | [BioSystems: WikiPathways](http://www.ncbi.nlm.nih.gov/biosystems/711360) | 9.36E-04 | 1.36E-02 | 9.65E-02 | 6.38E-01 | [6](http://toppgene.cchmc.org/showQueryTerms.jsp?userdata_id=41d53c5f-f68c-4e9f-8754-37ad37cd4269&feature=pt&row=46) | [194](http://toppgene.cchmc.org/showTermDetail.jsp?userdata_id=41d53c5f-f68c-4e9f-8754-37ad37cd4269&category=Pathway&id=711360) |
| 48 | P00033 | Insulin/IGF pathway-protein kinase B signaling cascade | [PantherDB](https://toppgene.cchmc.org/output.jsp?userdata_id=41d53c5f-f68c-4e9f-8754-37ad37cd4269) | 1.02E-03 | 1.44E-02 | 1.03E-01 | 6.93E-01 | [3](http://toppgene.cchmc.org/showQueryTerms.jsp?userdata_id=41d53c5f-f68c-4e9f-8754-37ad37cd4269&feature=pt&row=47) | [34](http://toppgene.cchmc.org/showTermDetail.jsp?userdata_id=41d53c5f-f68c-4e9f-8754-37ad37cd4269&category=Pathway&id=P00033) |
| 49 | 106459 | NGF signalling via TRKA from the plasma membrane | [BioSystems: REACTOME](http://www.ncbi.nlm.nih.gov/biosystems/106459) | 1.13E-03 | 1.57E-02 | 1.11E-01 | 7.67E-01 | [6](http://toppgene.cchmc.org/showQueryTerms.jsp?userdata_id=41d53c5f-f68c-4e9f-8754-37ad37cd4269&feature=pt&row=48) | [201](http://toppgene.cchmc.org/showTermDetail.jsp?userdata_id=41d53c5f-f68c-4e9f-8754-37ad37cd4269&category=Pathway&id=106459) |
| 50 | 645296 | EGFR Transactivation by Gastrin | [BioSystems: REACTOME](http://www.ncbi.nlm.nih.gov/biosystems/645296) | 1.19E-03 | 1.62E-02 | 1.15E-01 | 8.12E-01 | [2](http://toppgene.cchmc.org/showQueryTerms.jsp?userdata_id=41d53c5f-f68c-4e9f-8754-37ad37cd4269&feature=pt&row=49) | [9](http://toppgene.cchmc.org/showTermDetail.jsp?userdata_id=41d53c5f-f68c-4e9f-8754-37ad37cd4269&category=Pathway&id=645296) |

C IDH wild-type, grade II-III

| GO | ID | Name | Source | pValue | FDR B&H | FDR B&Y | Bonferroni | Genes from Input | Genes in Annotation |
| --- | --- | --- | --- | --- | --- | --- | --- | --- | --- |
| 1 | GO:0019901 | protein kinase binding |  | 3.76E-05 | 1.16E-02 | 7.28E-02 | 1.16E-02 | [10](http://toppgene.cchmc.org/showQueryTerms.jsp?userdata_id=5a6cd15e-c5c4-4917-8922-2aa74aaa92a0&feature=gof&row=0) | [452](http://toppgene.cchmc.org/showTermDetail.jsp?userdata_id=5a6cd15e-c5c4-4917-8922-2aa74aaa92a0&category=GeneOntologyMolecularFunction&id=GO:0019901) |
| 2 | GO:0019900 | kinase binding |  | 8.74E-05 | 1.34E-02 | 8.46E-02 | 2.68E-02 | [10](http://toppgene.cchmc.org/showQueryTerms.jsp?userdata_id=5a6cd15e-c5c4-4917-8922-2aa74aaa92a0&feature=gof&row=1) | [500](http://toppgene.cchmc.org/showTermDetail.jsp?userdata_id=5a6cd15e-c5c4-4917-8922-2aa74aaa92a0&category=GeneOntologyMolecularFunction&id=GO:0019900) |

| Pathway | ID | Name | Source | pValue | FDR B&H | FDR B&Y | Bonferroni | Genes from Input | Genes in Annotation |
| --- | --- | --- | --- | --- | --- | --- | --- | --- | --- |
| 1 | 83110 | Glioma | [BioSystems: KEGG](http://www.ncbi.nlm.nih.gov/biosystems/83110) | 5.01E-06 | 2.75E-03 | 1.96E-02 | 3.55E-03 | [5](http://toppgene.cchmc.org/showQueryTerms.jsp?userdata_id=5a6cd15e-c5c4-4917-8922-2aa74aaa92a0&feature=pt&row=0) | [65](http://toppgene.cchmc.org/showTermDetail.jsp?userdata_id=5a6cd15e-c5c4-4917-8922-2aa74aaa92a0&category=Pathway&id=83110) |
| 2 | 83114 | Melanoma | [BioSystems: KEGG](http://www.ncbi.nlm.nih.gov/biosystems/83114) | 7.76E-06 | 2.75E-03 | 1.96E-02 | 5.50E-03 | [5](http://toppgene.cchmc.org/showQueryTerms.jsp?userdata_id=5a6cd15e-c5c4-4917-8922-2aa74aaa92a0&feature=pt&row=1) | [71](http://toppgene.cchmc.org/showTermDetail.jsp?userdata_id=5a6cd15e-c5c4-4917-8922-2aa74aaa92a0&category=Pathway&id=83114) |
| 3 | 672458 | Signaling Pathways in Glioblastoma | [BioSystems: WikiPathways](http://www.ncbi.nlm.nih.gov/biosystems/672458) | 1.40E-05 | 2.76E-03 | 1.97E-02 | 9.89E-03 | [5](http://toppgene.cchmc.org/showQueryTerms.jsp?userdata_id=5a6cd15e-c5c4-4917-8922-2aa74aaa92a0&feature=pt&row=2) | [80](http://toppgene.cchmc.org/showTermDetail.jsp?userdata_id=5a6cd15e-c5c4-4917-8922-2aa74aaa92a0&category=Pathway&id=672458) |
| 4 | M18159 | RB Tumor Suppressor/Checkpoint Signaling in response to DNA damage | [MSigDB C2: BioCarta](http://www.broadinstitute.org/gsea/msigdb/cards/BIOCARTA_RB_PATHWAY.html) | 1.56E-05 | 2.76E-03 | 1.97E-02 | 1.10E-02 | [3](http://toppgene.cchmc.org/showQueryTerms.jsp?userdata_id=5a6cd15e-c5c4-4917-8922-2aa74aaa92a0&feature=pt&row=3) | [13](http://toppgene.cchmc.org/showTermDetail.jsp?userdata_id=5a6cd15e-c5c4-4917-8922-2aa74aaa92a0&category=Pathway&id=M18159) |
| 5 | 83105 | Pathways in cancer | [BioSystems: KEGG](http://www.ncbi.nlm.nih.gov/biosystems/83105) | 3.27E-05 | 4.64E-03 | 3.32E-02 | 2.32E-02 | [8](http://toppgene.cchmc.org/showQueryTerms.jsp?userdata_id=5a6cd15e-c5c4-4917-8922-2aa74aaa92a0&feature=pt&row=4) | [327](http://toppgene.cchmc.org/showTermDetail.jsp?userdata_id=5a6cd15e-c5c4-4917-8922-2aa74aaa92a0&category=Pathway&id=83105) |
| 6 | 692234 | PI3K-Akt signaling pathway | [BioSystems: KEGG](http://www.ncbi.nlm.nih.gov/biosystems/692234) | 4.89E-05 | 5.78E-03 | 4.13E-02 | 3.47E-02 | [8](http://toppgene.cchmc.org/showQueryTerms.jsp?userdata_id=5a6cd15e-c5c4-4917-8922-2aa74aaa92a0&feature=pt&row=5) | [346](http://toppgene.cchmc.org/showTermDetail.jsp?userdata_id=5a6cd15e-c5c4-4917-8922-2aa74aaa92a0&category=Pathway&id=692234) |
| 7 | M8560 | Cell Cycle: G2/M Checkpoint | [MSigDB C2: BioCarta](http://www.broadinstitute.org/gsea/msigdb/cards/BIOCARTA_G2_PATHWAY.html) | 1.07E-04 | 9.79E-03 | 6.99E-02 | 7.57E-02 | [3](http://toppgene.cchmc.org/showQueryTerms.jsp?userdata_id=5a6cd15e-c5c4-4917-8922-2aa74aaa92a0&feature=pt&row=6) | [24](http://toppgene.cchmc.org/showTermDetail.jsp?userdata_id=5a6cd15e-c5c4-4917-8922-2aa74aaa92a0&category=Pathway&id=M8560) |
| 8 | 83108 | Pancreatic cancer | [BioSystems: KEGG](http://www.ncbi.nlm.nih.gov/biosystems/83108) | 1.23E-04 | 9.79E-03 | 6.99E-02 | 8.71E-02 | [4](http://toppgene.cchmc.org/showQueryTerms.jsp?userdata_id=5a6cd15e-c5c4-4917-8922-2aa74aaa92a0&feature=pt&row=7) | [66](http://toppgene.cchmc.org/showTermDetail.jsp?userdata_id=5a6cd15e-c5c4-4917-8922-2aa74aaa92a0&category=Pathway&id=83108) |
| 9 | M14512 | Influence of Ras and Rho proteins on G1 to S Transition | [MSigDB C2: BioCarta](http://www.broadinstitute.org/gsea/msigdb/cards/BIOCARTA_RACCYCD_PATHWAY.html) | 1.36E-04 | 9.79E-03 | 6.99E-02 | 9.67E-02 | [3](http://toppgene.cchmc.org/showQueryTerms.jsp?userdata_id=5a6cd15e-c5c4-4917-8922-2aa74aaa92a0&feature=pt&row=8) | [26](http://toppgene.cchmc.org/showTermDetail.jsp?userdata_id=5a6cd15e-c5c4-4917-8922-2aa74aaa92a0&category=Pathway&id=M14512) |
| 10 | 198805 | G1 to S cell cycle control | [BioSystems: WikiPathways](http://www.ncbi.nlm.nih.gov/biosystems/198805) | 1.38E-04 | 9.79E-03 | 6.99E-02 | 9.79E-02 | [4](http://toppgene.cchmc.org/showQueryTerms.jsp?userdata_id=5a6cd15e-c5c4-4917-8922-2aa74aaa92a0&feature=pt&row=9) | [68](http://toppgene.cchmc.org/showTermDetail.jsp?userdata_id=5a6cd15e-c5c4-4917-8922-2aa74aaa92a0&category=Pathway&id=198805) |
| 11 | 83116 | Chronic myeloid leukemia | [BioSystems: KEGG](http://www.ncbi.nlm.nih.gov/biosystems/83116) | 1.82E-04 | 1.17E-02 | 8.37E-02 | 1.29E-01 | [4](http://toppgene.cchmc.org/showQueryTerms.jsp?userdata_id=5a6cd15e-c5c4-4917-8922-2aa74aaa92a0&feature=pt&row=10) | [73](http://toppgene.cchmc.org/showTermDetail.jsp?userdata_id=5a6cd15e-c5c4-4917-8922-2aa74aaa92a0&category=Pathway&id=83116) |
| 12 | 694606 | Hepatitis B | [BioSystems: KEGG](http://www.ncbi.nlm.nih.gov/biosystems/694606) | 2.48E-04 | 1.40E-02 | 9.97E-02 | 1.76E-01 | [5](http://toppgene.cchmc.org/showQueryTerms.jsp?userdata_id=5a6cd15e-c5c4-4917-8922-2aa74aaa92a0&feature=pt&row=11) | [146](http://toppgene.cchmc.org/showTermDetail.jsp?userdata_id=5a6cd15e-c5c4-4917-8922-2aa74aaa92a0&category=Pathway&id=694606) |
| 13 | 905992 | Oncogene Induced Senescence | [BioSystems: REACTOME](http://www.ncbi.nlm.nih.gov/biosystems/905992) | 2.56E-04 | 1.40E-02 | 9.97E-02 | 1.82E-01 | [3](http://toppgene.cchmc.org/showQueryTerms.jsp?userdata_id=5a6cd15e-c5c4-4917-8922-2aa74aaa92a0&feature=pt&row=12) | [32](http://toppgene.cchmc.org/showTermDetail.jsp?userdata_id=5a6cd15e-c5c4-4917-8922-2aa74aaa92a0&category=Pathway&id=905992) |
| 14 | 219801 | Integrated Breast Cancer Pathway | [BioSystems: WikiPathways](http://www.ncbi.nlm.nih.gov/biosystems/219801) | 3.17E-04 | 1.58E-02 | 1.13E-01 | 2.25E-01 | [5](http://toppgene.cchmc.org/showQueryTerms.jsp?userdata_id=5a6cd15e-c5c4-4917-8922-2aa74aaa92a0&feature=pt&row=13) | [154](http://toppgene.cchmc.org/showTermDetail.jsp?userdata_id=5a6cd15e-c5c4-4917-8922-2aa74aaa92a0&category=Pathway&id=219801) |
| 15 | 672450 | Integrated Cancer pathway | [BioSystems: WikiPathways](http://www.ncbi.nlm.nih.gov/biosystems/672450) | 3.35E-04 | 1.58E-02 | 1.13E-01 | 2.38E-01 | [3](http://toppgene.cchmc.org/showQueryTerms.jsp?userdata_id=5a6cd15e-c5c4-4917-8922-2aa74aaa92a0&feature=pt&row=14) | [35](http://toppgene.cchmc.org/showTermDetail.jsp?userdata_id=5a6cd15e-c5c4-4917-8922-2aa74aaa92a0&category=Pathway&id=672450) |
| 16 | 920977 | RB in Cancer | [BioSystems: WikiPathways](http://www.ncbi.nlm.nih.gov/biosystems/920977) | 3.58E-04 | 1.58E-02 | 1.13E-01 | 2.54E-01 | [4](http://toppgene.cchmc.org/showQueryTerms.jsp?userdata_id=5a6cd15e-c5c4-4917-8922-2aa74aaa92a0&feature=pt&row=15) | [87](http://toppgene.cchmc.org/showTermDetail.jsp?userdata_id=5a6cd15e-c5c4-4917-8922-2aa74aaa92a0&category=Pathway&id=920977) |
| 17 | 83111 | Prostate cancer | [BioSystems: KEGG](http://www.ncbi.nlm.nih.gov/biosystems/83111) | 3.90E-04 | 1.60E-02 | 1.14E-01 | 2.77E-01 | [4](http://toppgene.cchmc.org/showQueryTerms.jsp?userdata_id=5a6cd15e-c5c4-4917-8922-2aa74aaa92a0&feature=pt&row=16) | [89](http://toppgene.cchmc.org/showTermDetail.jsp?userdata_id=5a6cd15e-c5c4-4917-8922-2aa74aaa92a0&category=Pathway&id=83111) |
| 18 | 83115 | Bladder cancer | [BioSystems: KEGG](http://www.ncbi.nlm.nih.gov/biosystems/83115) | 4.28E-04 | 1.60E-02 | 1.14E-01 | 3.04E-01 | [3](http://toppgene.cchmc.org/showQueryTerms.jsp?userdata_id=5a6cd15e-c5c4-4917-8922-2aa74aaa92a0&feature=pt&row=17) | [38](http://toppgene.cchmc.org/showTermDetail.jsp?userdata_id=5a6cd15e-c5c4-4917-8922-2aa74aaa92a0&category=Pathway&id=83115) |
| 19 | 198779 | MAPK signaling pathway | [BioSystems: WikiPathways](http://www.ncbi.nlm.nih.gov/biosystems/198779) | 4.60E-04 | 1.60E-02 | 1.14E-01 | 3.26E-01 | [5](http://toppgene.cchmc.org/showQueryTerms.jsp?userdata_id=5a6cd15e-c5c4-4917-8922-2aa74aaa92a0&feature=pt&row=18) | [167](http://toppgene.cchmc.org/showTermDetail.jsp?userdata_id=5a6cd15e-c5c4-4917-8922-2aa74aaa92a0&category=Pathway&id=198779) |
| 20 | 137935 | FOXM1 transcription factor network | [BioSystems: Pathway Interaction Database](http://www.ncbi.nlm.nih.gov/biosystems/137935) | 4.63E-04 | 1.60E-02 | 1.14E-01 | 3.28E-01 | [3](http://toppgene.cchmc.org/showQueryTerms.jsp?userdata_id=5a6cd15e-c5c4-4917-8922-2aa74aaa92a0&feature=pt&row=19) | [39](http://toppgene.cchmc.org/showTermDetail.jsp?userdata_id=5a6cd15e-c5c4-4917-8922-2aa74aaa92a0&category=Pathway&id=137935) |
| 21 | 83048 | MAPK signaling pathway | [BioSystems: KEGG](http://www.ncbi.nlm.nih.gov/biosystems/83048) | 4.73E-04 | 1.60E-02 | 1.14E-01 | 3.36E-01 | [6](http://toppgene.cchmc.org/showQueryTerms.jsp?userdata_id=5a6cd15e-c5c4-4917-8922-2aa74aaa92a0&feature=pt&row=20) | [259](http://toppgene.cchmc.org/showTermDetail.jsp?userdata_id=5a6cd15e-c5c4-4917-8922-2aa74aaa92a0&category=Pathway&id=83048) |
| 22 | 198811 | Cell cycle | [BioSystems: WikiPathways](http://www.ncbi.nlm.nih.gov/biosystems/198811) | 6.54E-04 | 2.11E-02 | 1.51E-01 | 4.64E-01 | [4](http://toppgene.cchmc.org/showQueryTerms.jsp?userdata_id=5a6cd15e-c5c4-4917-8922-2aa74aaa92a0&feature=pt&row=21) | [102](http://toppgene.cchmc.org/showTermDetail.jsp?userdata_id=5a6cd15e-c5c4-4917-8922-2aa74aaa92a0&category=Pathway&id=198811) |
| 23 | 755440 | Prostate Cancer | [BioSystems: WikiPathways](http://www.ncbi.nlm.nih.gov/biosystems/755440) | 8.69E-04 | 2.68E-02 | 1.91E-01 | 6.16E-01 | [4](http://toppgene.cchmc.org/showQueryTerms.jsp?userdata_id=5a6cd15e-c5c4-4917-8922-2aa74aaa92a0&feature=pt&row=22) | [110](http://toppgene.cchmc.org/showTermDetail.jsp?userdata_id=5a6cd15e-c5c4-4917-8922-2aa74aaa92a0&category=Pathway&id=755440) |
| 24 | 852705 | MicroRNAs in cancer | [BioSystems: KEGG](http://www.ncbi.nlm.nih.gov/biosystems/852705) | 9.55E-04 | 2.82E-02 | 2.01E-01 | 6.77E-01 | [6](http://toppgene.cchmc.org/showQueryTerms.jsp?userdata_id=5a6cd15e-c5c4-4917-8922-2aa74aaa92a0&feature=pt&row=23) | [296](http://toppgene.cchmc.org/showTermDetail.jsp?userdata_id=5a6cd15e-c5c4-4917-8922-2aa74aaa92a0&category=Pathway&id=852705) |
| 25 | P05912 | Dopamine receptor mediated signaling pathway | [PantherDB](https://toppgene.cchmc.org/output.jsp?userdata_id=5a6cd15e-c5c4-4917-8922-2aa74aaa92a0) | 1.08E-03 | 3.06E-02 | 2.19E-01 | 7.66E-01 | [3](http://toppgene.cchmc.org/showQueryTerms.jsp?userdata_id=5a6cd15e-c5c4-4917-8922-2aa74aaa92a0&feature=pt&row=24) | [52](http://toppgene.cchmc.org/showTermDetail.jsp?userdata_id=5a6cd15e-c5c4-4917-8922-2aa74aaa92a0&category=Pathway&id=P05912) |
| 26 | PW:0000713 | vasopressin signaling pathway via receptor type 2 | [Pathway Ontology](http://rgd.mcw.edu/rgdweb/ontology/view.html?acc_id=PW:0000713) | 1.14E-03 | 3.10E-02 | 2.21E-01 | 8.06E-01 | [2](http://toppgene.cchmc.org/showQueryTerms.jsp?userdata_id=5a6cd15e-c5c4-4917-8922-2aa74aaa92a0&feature=pt&row=25) | [13](http://toppgene.cchmc.org/showTermDetail.jsp?userdata_id=5a6cd15e-c5c4-4917-8922-2aa74aaa92a0&category=Pathway&id=PW:0000713) |
| 27 | 83119 | Non-small cell lung cancer | [BioSystems: KEGG](http://www.ncbi.nlm.nih.gov/biosystems/83119) | 1.34E-03 | 3.37E-02 | 2.41E-01 | 9.50E-01 | [3](http://toppgene.cchmc.org/showQueryTerms.jsp?userdata_id=5a6cd15e-c5c4-4917-8922-2aa74aaa92a0&feature=pt&row=26) | [56](http://toppgene.cchmc.org/showTermDetail.jsp?userdata_id=5a6cd15e-c5c4-4917-8922-2aa74aaa92a0&category=Pathway&id=83119) |
| 28 | 83054 | Cell cycle | [BioSystems: KEGG](http://www.ncbi.nlm.nih.gov/biosystems/83054) | 1.36E-03 | 3.37E-02 | 2.41E-01 | 9.62E-01 | [4](http://toppgene.cchmc.org/showQueryTerms.jsp?userdata_id=5a6cd15e-c5c4-4917-8922-2aa74aaa92a0&feature=pt&row=27) | [124](http://toppgene.cchmc.org/showTermDetail.jsp?userdata_id=5a6cd15e-c5c4-4917-8922-2aa74aaa92a0&category=Pathway&id=83054) |
| 29 | 868086 | Rap1 signaling pathway | [BioSystems: KEGG](http://www.ncbi.nlm.nih.gov/biosystems/868086) | 1.38E-03 | 3.37E-02 | 2.41E-01 | 9.78E-01 | [5](http://toppgene.cchmc.org/showQueryTerms.jsp?userdata_id=5a6cd15e-c5c4-4917-8922-2aa74aaa92a0&feature=pt&row=28) | [213](http://toppgene.cchmc.org/showTermDetail.jsp?userdata_id=5a6cd15e-c5c4-4917-8922-2aa74aaa92a0&category=Pathway&id=868086) |
| 30 | M1529 | Cdk2, 4, and 6 bind cyclin D in G1, while cdk2/cyclin E promotes the G1/S transition. | [MSigDB C2: SigmaAldrich](http://www.broadinstitute.org/gsea/msigdb/cards/SA_G1_AND_S_PHASES.html) | 1.52E-03 | 3.60E-02 | 2.57E-01 | 1.00E+00 | [2](http://toppgene.cchmc.org/showQueryTerms.jsp?userdata_id=5a6cd15e-c5c4-4917-8922-2aa74aaa92a0&feature=pt&row=29) | [15](http://toppgene.cchmc.org/showTermDetail.jsp?userdata_id=5a6cd15e-c5c4-4917-8922-2aa74aaa92a0&category=Pathway&id=M1529) |
| 31 | 187108 | Rap1 signalling | [BioSystems: REACTOME](http://www.ncbi.nlm.nih.gov/biosystems/187108) | 1.74E-03 | 3.70E-02 | 2.64E-01 | 1.00E+00 | [2](http://toppgene.cchmc.org/showQueryTerms.jsp?userdata_id=5a6cd15e-c5c4-4917-8922-2aa74aaa92a0&feature=pt&row=30) | [16](http://toppgene.cchmc.org/showTermDetail.jsp?userdata_id=5a6cd15e-c5c4-4917-8922-2aa74aaa92a0&category=Pathway&id=187108) |
| 32 | M14863 | p53 Signaling Pathway | [MSigDB C2: BioCarta](http://www.broadinstitute.org/gsea/msigdb/cards/BIOCARTA_P53_PATHWAY.html) | 1.74E-03 | 3.70E-02 | 2.64E-01 | 1.00E+00 | [2](http://toppgene.cchmc.org/showQueryTerms.jsp?userdata_id=5a6cd15e-c5c4-4917-8922-2aa74aaa92a0&feature=pt&row=31) | [16](http://toppgene.cchmc.org/showTermDetail.jsp?userdata_id=5a6cd15e-c5c4-4917-8922-2aa74aaa92a0&category=Pathway&id=M14863) |
| 33 | 173973 | Hepatitis C | [BioSystems: KEGG](http://www.ncbi.nlm.nih.gov/biosystems/173973) | 1.76E-03 | 3.70E-02 | 2.64E-01 | 1.00E+00 | [4](http://toppgene.cchmc.org/showQueryTerms.jsp?userdata_id=5a6cd15e-c5c4-4917-8922-2aa74aaa92a0&feature=pt&row=32) | [133](http://toppgene.cchmc.org/showTermDetail.jsp?userdata_id=5a6cd15e-c5c4-4917-8922-2aa74aaa92a0&category=Pathway&id=173973) |
| 34 | 213306 | Measles | [BioSystems: KEGG](http://www.ncbi.nlm.nih.gov/biosystems/213306) | 1.81E-03 | 3.70E-02 | 2.64E-01 | 1.00E+00 | [4](http://toppgene.cchmc.org/showQueryTerms.jsp?userdata_id=5a6cd15e-c5c4-4917-8922-2aa74aaa92a0&feature=pt&row=33) | [134](http://toppgene.cchmc.org/showTermDetail.jsp?userdata_id=5a6cd15e-c5c4-4917-8922-2aa74aaa92a0&category=Pathway&id=213306) |
| 35 | 868085 | Ras signaling pathway | [BioSystems: KEGG](http://www.ncbi.nlm.nih.gov/biosystems/868085) | 1.83E-03 | 3.70E-02 | 2.64E-01 | 1.00E+00 | [5](http://toppgene.cchmc.org/showQueryTerms.jsp?userdata_id=5a6cd15e-c5c4-4917-8922-2aa74aaa92a0&feature=pt&row=34) | [227](http://toppgene.cchmc.org/showTermDetail.jsp?userdata_id=5a6cd15e-c5c4-4917-8922-2aa74aaa92a0&category=Pathway&id=868085) |
| 36 | 125137 | Cytosolic DNA-sensing pathway | [BioSystems: KEGG](http://www.ncbi.nlm.nih.gov/biosystems/125137) | 1.88E-03 | 3.71E-02 | 2.65E-01 | 1.00E+00 | [3](http://toppgene.cchmc.org/showQueryTerms.jsp?userdata_id=5a6cd15e-c5c4-4917-8922-2aa74aaa92a0&feature=pt&row=35) | [63](http://toppgene.cchmc.org/showTermDetail.jsp?userdata_id=5a6cd15e-c5c4-4917-8922-2aa74aaa92a0&category=Pathway&id=125137) |
| 37 | PW:0000491 | vasopressin signaling | [Pathway Ontology](http://rgd.mcw.edu/rgdweb/ontology/view.html?acc_id=PW:0000491) | 1.96E-03 | 3.76E-02 | 2.69E-01 | 1.00E+00 | [2](http://toppgene.cchmc.org/showQueryTerms.jsp?userdata_id=5a6cd15e-c5c4-4917-8922-2aa74aaa92a0&feature=pt&row=36) | [17](http://toppgene.cchmc.org/showTermDetail.jsp?userdata_id=5a6cd15e-c5c4-4917-8922-2aa74aaa92a0&category=Pathway&id=PW:0000491) |
| 38 | M10401 | Telomeres, Telomerase, Cellular Aging, and Immortality | [MSigDB C2: BioCarta](http://www.broadinstitute.org/gsea/msigdb/cards/BIOCARTA_TEL_PATHWAY.html) | 2.20E-03 | 3.96E-02 | 2.83E-01 | 1.00E+00 | [2](http://toppgene.cchmc.org/showQueryTerms.jsp?userdata_id=5a6cd15e-c5c4-4917-8922-2aa74aaa92a0&feature=pt&row=37) | [18](http://toppgene.cchmc.org/showTermDetail.jsp?userdata_id=5a6cd15e-c5c4-4917-8922-2aa74aaa92a0&category=Pathway&id=M10401) |
| 39 | M7552 | Role of EGF Receptor Transactivation by GPCRs in Cardiac Hypertrophy | [MSigDB C2: BioCarta](http://www.broadinstitute.org/gsea/msigdb/cards/BIOCARTA_CARDIACEGF_PATHWAY.html) | 2.20E-03 | 3.96E-02 | 2.83E-01 | 1.00E+00 | [2](http://toppgene.cchmc.org/showQueryTerms.jsp?userdata_id=5a6cd15e-c5c4-4917-8922-2aa74aaa92a0&feature=pt&row=38) | [18](http://toppgene.cchmc.org/showTermDetail.jsp?userdata_id=5a6cd15e-c5c4-4917-8922-2aa74aaa92a0&category=Pathway&id=M7552) |
| 40 | 198788 | DNA damage response | [BioSystems: WikiPathways](http://www.ncbi.nlm.nih.gov/biosystems/198788) | 2.25E-03 | 3.96E-02 | 2.83E-01 | 1.00E+00 | [3](http://toppgene.cchmc.org/showQueryTerms.jsp?userdata_id=5a6cd15e-c5c4-4917-8922-2aa74aaa92a0&feature=pt&row=39) | [67](http://toppgene.cchmc.org/showTermDetail.jsp?userdata_id=5a6cd15e-c5c4-4917-8922-2aa74aaa92a0&category=Pathway&id=198788) |
| 41 | 83055 | p53 signaling pathway | [BioSystems: KEGG](http://www.ncbi.nlm.nih.gov/biosystems/83055) | 2.35E-03 | 3.96E-02 | 2.83E-01 | 1.00E+00 | [3](http://toppgene.cchmc.org/showQueryTerms.jsp?userdata_id=5a6cd15e-c5c4-4917-8922-2aa74aaa92a0&feature=pt&row=40) | [68](http://toppgene.cchmc.org/showTermDetail.jsp?userdata_id=5a6cd15e-c5c4-4917-8922-2aa74aaa92a0&category=Pathway&id=83055) |
| 42 | 576255 | Cytosolic sensors of pathogen-associated DNA | [BioSystems: REACTOME](http://www.ncbi.nlm.nih.gov/biosystems/576255) | 2.35E-03 | 3.96E-02 | 2.83E-01 | 1.00E+00 | [3](http://toppgene.cchmc.org/showQueryTerms.jsp?userdata_id=5a6cd15e-c5c4-4917-8922-2aa74aaa92a0&feature=pt&row=41) | [68](http://toppgene.cchmc.org/showTermDetail.jsp?userdata_id=5a6cd15e-c5c4-4917-8922-2aa74aaa92a0&category=Pathway&id=576255) |
| 43 | M10066 | Corticosteroids and cardioprotection | [MSigDB C2: BioCarta](http://www.broadinstitute.org/gsea/msigdb/cards/BIOCARTA_GCR_PATHWAY.html) | 2.72E-03 | 4.38E-02 | 3.13E-01 | 1.00E+00 | [2](http://toppgene.cchmc.org/showQueryTerms.jsp?userdata_id=5a6cd15e-c5c4-4917-8922-2aa74aaa92a0&feature=pt&row=42) | [20](http://toppgene.cchmc.org/showTermDetail.jsp?userdata_id=5a6cd15e-c5c4-4917-8922-2aa74aaa92a0&category=Pathway&id=M10066) |
| 44 | M10628 | ATM Signaling Pathway | [MSigDB C2: BioCarta](http://www.broadinstitute.org/gsea/msigdb/cards/BIOCARTA_ATM_PATHWAY.html) | 2.72E-03 | 4.38E-02 | 3.13E-01 | 1.00E+00 | [2](http://toppgene.cchmc.org/showQueryTerms.jsp?userdata_id=5a6cd15e-c5c4-4917-8922-2aa74aaa92a0&feature=pt&row=43) | [20](http://toppgene.cchmc.org/showTermDetail.jsp?userdata_id=5a6cd15e-c5c4-4917-8922-2aa74aaa92a0&category=Pathway&id=M10628) |
| 45 | 576258 | RIP-mediated NFkB activation via ZBP1 | [BioSystems: REACTOME](http://www.ncbi.nlm.nih.gov/biosystems/576258) | 3.00E-03 | 4.73E-02 | 3.38E-01 | 1.00E+00 | [2](http://toppgene.cchmc.org/showQueryTerms.jsp?userdata_id=5a6cd15e-c5c4-4917-8922-2aa74aaa92a0&feature=pt&row=44) | [21](http://toppgene.cchmc.org/showTermDetail.jsp?userdata_id=5a6cd15e-c5c4-4917-8922-2aa74aaa92a0&category=Pathway&id=576258) |

D IDH wild-type, GBM

| GO:Fuction | ID | Name | Source | pValue | FDR B&H | FDR B&Y | Bonferroni | Genes from Input | Genes in Annotation |
| --- | --- | --- | --- | --- | --- | --- | --- | --- | --- |
| 1 | GO:0005006 | epidermal growth factor-activated receptor activity |  | 1.10E-04 | 3.15E-02 | 1.96E-01 | 3.15E-02 | [2](http://toppgene.cchmc.org/showQueryTerms.jsp?userdata_id=4424d37c-e975-4a14-99f5-c7d702647239&feature=gof&row=0) | [4](http://toppgene.cchmc.org/showTermDetail.jsp?userdata_id=4424d37c-e975-4a14-99f5-c7d702647239&category=GeneOntologyMolecularFunction&id=GO:0005006) |
| 2 | GO:0055103 | ligase regulator activity |  | 2.74E-04 | 3.91E-02 | 2.44E-01 | 7.83E-02 | [2](http://toppgene.cchmc.org/showQueryTerms.jsp?userdata_id=4424d37c-e975-4a14-99f5-c7d702647239&feature=gof&row=1) | [6](http://toppgene.cchmc.org/showTermDetail.jsp?userdata_id=4424d37c-e975-4a14-99f5-c7d702647239&category=GeneOntologyMolecularFunction&id=GO:0055103) |

| GO:Process | ID | Name | Source | pValue | FDR B&H | FDR B&Y | Bonferroni | Genes from Input | Genes in Annotation |
| --- | --- | --- | --- | --- | --- | --- | --- | --- | --- |
| 1 | GO:0048732 | gland development |  | 3.46E-05 | 2.21E-02 | 1.81E-01 | 6.93E-02 | [8](http://toppgene.cchmc.org/showQueryTerms.jsp?userdata_id=4424d37c-e975-4a14-99f5-c7d702647239&feature=gop&row=0) | [294](http://toppgene.cchmc.org/showTermDetail.jsp?userdata_id=4424d37c-e975-4a14-99f5-c7d702647239&category=GeneOntologyBiologicalProcess&id=GO:0048732) |
| 2 | GO:0048853 | forebrain morphogenesis |  | 4.79E-05 | 2.21E-02 | 1.81E-01 | 9.60E-02 | [3](http://toppgene.cchmc.org/showQueryTerms.jsp?userdata_id=4424d37c-e975-4a14-99f5-c7d702647239&feature=gop&row=1) | [17](http://toppgene.cchmc.org/showTermDetail.jsp?userdata_id=4424d37c-e975-4a14-99f5-c7d702647239&category=GeneOntologyBiologicalProcess&id=GO:0048853) |
| 3 | GO:0009967 | positive regulation of signal transduction |  | 6.58E-05 | 2.21E-02 | 1.81E-01 | 1.32E-01 | [14](http://toppgene.cchmc.org/showQueryTerms.jsp?userdata_id=4424d37c-e975-4a14-99f5-c7d702647239&feature=gop&row=2) | [993](http://toppgene.cchmc.org/showTermDetail.jsp?userdata_id=4424d37c-e975-4a14-99f5-c7d702647239&category=GeneOntologyBiologicalProcess&id=GO:0009967) |
| 4 | GO:0050680 | negative regulation of epithelial cell proliferation |  | 7.65E-05 | 2.21E-02 | 1.81E-01 | 1.53E-01 | [5](http://toppgene.cchmc.org/showQueryTerms.jsp?userdata_id=4424d37c-e975-4a14-99f5-c7d702647239&feature=gop&row=3) | [103](http://toppgene.cchmc.org/showTermDetail.jsp?userdata_id=4424d37c-e975-4a14-99f5-c7d702647239&category=GeneOntologyBiologicalProcess&id=GO:0050680) |
| 5 | GO:0050678 | regulation of epithelial cell proliferation |  | 8.10E-05 | 2.21E-02 | 1.81E-01 | 1.62E-01 | [7](http://toppgene.cchmc.org/showQueryTerms.jsp?userdata_id=4424d37c-e975-4a14-99f5-c7d702647239&feature=gop&row=4) | [245](http://toppgene.cchmc.org/showTermDetail.jsp?userdata_id=4424d37c-e975-4a14-99f5-c7d702647239&category=GeneOntologyBiologicalProcess&id=GO:0050678) |
| 6 | GO:0033088 | negative regulation of immature T cell proliferation in thymus |  | 1.06E-04 | 2.21E-02 | 1.81E-01 | 2.13E-01 | [2](http://toppgene.cchmc.org/showQueryTerms.jsp?userdata_id=4424d37c-e975-4a14-99f5-c7d702647239&feature=gop&row=5) | [4](http://toppgene.cchmc.org/showTermDetail.jsp?userdata_id=4424d37c-e975-4a14-99f5-c7d702647239&category=GeneOntologyBiologicalProcess&id=GO:0033088) |
| 7 | GO:0033087 | negative regulation of immature T cell proliferation |  | 1.06E-04 | 2.21E-02 | 1.81E-01 | 2.13E-01 | [2](http://toppgene.cchmc.org/showQueryTerms.jsp?userdata_id=4424d37c-e975-4a14-99f5-c7d702647239&feature=gop&row=6) | [4](http://toppgene.cchmc.org/showTermDetail.jsp?userdata_id=4424d37c-e975-4a14-99f5-c7d702647239&category=GeneOntologyBiologicalProcess&id=GO:0033087) |
| 8 | GO:0001953 | negative regulation of cell-matrix adhesion |  | 1.07E-04 | 2.21E-02 | 1.81E-01 | 2.14E-01 | [3](http://toppgene.cchmc.org/showQueryTerms.jsp?userdata_id=4424d37c-e975-4a14-99f5-c7d702647239&feature=gop&row=7) | [22](http://toppgene.cchmc.org/showTermDetail.jsp?userdata_id=4424d37c-e975-4a14-99f5-c7d702647239&category=GeneOntologyBiologicalProcess&id=GO:0001953) |
| 9 | GO:0023056 | positive regulation of signaling |  | 1.19E-04 | 2.21E-02 | 1.81E-01 | 2.39E-01 | [14](http://toppgene.cchmc.org/showQueryTerms.jsp?userdata_id=4424d37c-e975-4a14-99f5-c7d702647239&feature=gop&row=8) | [1050](http://toppgene.cchmc.org/showTermDetail.jsp?userdata_id=4424d37c-e975-4a14-99f5-c7d702647239&category=GeneOntologyBiologicalProcess&id=GO:0023056) |
| 10 | GO:0031032 | actomyosin structure organization |  | 1.22E-04 | 2.21E-02 | 1.81E-01 | 2.45E-01 | [4](http://toppgene.cchmc.org/showQueryTerms.jsp?userdata_id=4424d37c-e975-4a14-99f5-c7d702647239&feature=gop&row=9) | [60](http://toppgene.cchmc.org/showTermDetail.jsp?userdata_id=4424d37c-e975-4a14-99f5-c7d702647239&category=GeneOntologyBiologicalProcess&id=GO:0031032) |
| 11 | GO:0010647 | positive regulation of cell communication |  | 1.25E-04 | 2.21E-02 | 1.81E-01 | 2.51E-01 | [14](http://toppgene.cchmc.org/showQueryTerms.jsp?userdata_id=4424d37c-e975-4a14-99f5-c7d702647239&feature=gop&row=10) | [1055](http://toppgene.cchmc.org/showTermDetail.jsp?userdata_id=4424d37c-e975-4a14-99f5-c7d702647239&category=GeneOntologyBiologicalProcess&id=GO:0010647) |
| 12 | GO:0007010 | cytoskeleton organization |  | 1.32E-04 | 2.21E-02 | 1.81E-01 | 2.65E-01 | [13](http://toppgene.cchmc.org/showQueryTerms.jsp?userdata_id=4424d37c-e975-4a14-99f5-c7d702647239&feature=gop&row=11) | [928](http://toppgene.cchmc.org/showTermDetail.jsp?userdata_id=4424d37c-e975-4a14-99f5-c7d702647239&category=GeneOntologyBiologicalProcess&id=GO:0007010) |
| 13 | GO:0042063 | gliogenesis |  | 1.95E-04 | 3.01E-02 | 2.46E-01 | 3.91E-01 | [6](http://toppgene.cchmc.org/showQueryTerms.jsp?userdata_id=4424d37c-e975-4a14-99f5-c7d702647239&feature=gop&row=12) | [198](http://toppgene.cchmc.org/showTermDetail.jsp?userdata_id=4424d37c-e975-4a14-99f5-c7d702647239&category=GeneOntologyBiologicalProcess&id=GO:0042063) |
| 14 | GO:0050673 | epithelial cell proliferation |  | 2.55E-04 | 3.65E-02 | 2.98E-01 | 5.11E-01 | [7](http://toppgene.cchmc.org/showQueryTerms.jsp?userdata_id=4424d37c-e975-4a14-99f5-c7d702647239&feature=gop&row=13) | [295](http://toppgene.cchmc.org/showTermDetail.jsp?userdata_id=4424d37c-e975-4a14-99f5-c7d702647239&category=GeneOntologyBiologicalProcess&id=GO:0050673) |
| 15 | GO:0050821 | protein stabilization |  | 3.06E-04 | 4.08E-02 | 3.34E-01 | 6.12E-01 | [4](http://toppgene.cchmc.org/showQueryTerms.jsp?userdata_id=4424d37c-e975-4a14-99f5-c7d702647239&feature=gop&row=14) | [76](http://toppgene.cchmc.org/showTermDetail.jsp?userdata_id=4424d37c-e975-4a14-99f5-c7d702647239&category=GeneOntologyBiologicalProcess&id=GO:0050821) |
| 16 | GO:0048854 | brain morphogenesis |  | 3.34E-04 | 4.18E-02 | 3.42E-01 | 6.69E-01 | [3](http://toppgene.cchmc.org/showQueryTerms.jsp?userdata_id=4424d37c-e975-4a14-99f5-c7d702647239&feature=gop&row=15) | [32](http://toppgene.cchmc.org/showTermDetail.jsp?userdata_id=4424d37c-e975-4a14-99f5-c7d702647239&category=GeneOntologyBiologicalProcess&id=GO:0048854) |
| 17 | GO:0033084 | regulation of immature T cell proliferation in thymus |  | 3.68E-04 | 4.31E-02 | 3.53E-01 | 7.38E-01 | [2](http://toppgene.cchmc.org/showQueryTerms.jsp?userdata_id=4424d37c-e975-4a14-99f5-c7d702647239&feature=gop&row=16) | [7](http://toppgene.cchmc.org/showTermDetail.jsp?userdata_id=4424d37c-e975-4a14-99f5-c7d702647239&category=GeneOntologyBiologicalProcess&id=GO:0033084) |
| 18 | GO:0009891 | positive regulation of biosynthetic process |  | 4.17E-04 | 4.31E-02 | 3.53E-01 | 8.36E-01 | [16](http://toppgene.cchmc.org/showQueryTerms.jsp?userdata_id=4424d37c-e975-4a14-99f5-c7d702647239&feature=gop&row=17) | [1482](http://toppgene.cchmc.org/showTermDetail.jsp?userdata_id=4424d37c-e975-4a14-99f5-c7d702647239&category=GeneOntologyBiologicalProcess&id=GO:0009891) |
| 19 | GO:2000113 | negative regulation of cellular macromolecule biosynthetic process |  | 4.65E-04 | 4.31E-02 | 3.53E-01 | 9.32E-01 | [13](http://toppgene.cchmc.org/showQueryTerms.jsp?userdata_id=4424d37c-e975-4a14-99f5-c7d702647239&feature=gop&row=18) | [1056](http://toppgene.cchmc.org/showTermDetail.jsp?userdata_id=4424d37c-e975-4a14-99f5-c7d702647239&category=GeneOntologyBiologicalProcess&id=GO:2000113) |
| 20 | GO:0010557 | positive regulation of macromolecule biosynthetic process |  | 4.71E-04 | 4.31E-02 | 3.53E-01 | 9.44E-01 | [15](http://toppgene.cchmc.org/showQueryTerms.jsp?userdata_id=4424d37c-e975-4a14-99f5-c7d702647239&feature=gop&row=19) | [1347](http://toppgene.cchmc.org/showTermDetail.jsp?userdata_id=4424d37c-e975-4a14-99f5-c7d702647239&category=GeneOntologyBiologicalProcess&id=GO:0010557) |
| 21 | GO:0033080 | immature T cell proliferation in thymus |  | 4.90E-04 | 4.31E-02 | 3.53E-01 | 9.81E-01 | [2](http://toppgene.cchmc.org/showQueryTerms.jsp?userdata_id=4424d37c-e975-4a14-99f5-c7d702647239&feature=gop&row=20) | [8](http://toppgene.cchmc.org/showTermDetail.jsp?userdata_id=4424d37c-e975-4a14-99f5-c7d702647239&category=GeneOntologyBiologicalProcess&id=GO:0033080) |
| 22 | GO:0033083 | regulation of immature T cell proliferation |  | 4.90E-04 | 4.31E-02 | 3.53E-01 | 9.81E-01 | [2](http://toppgene.cchmc.org/showQueryTerms.jsp?userdata_id=4424d37c-e975-4a14-99f5-c7d702647239&feature=gop&row=21) | [8](http://toppgene.cchmc.org/showTermDetail.jsp?userdata_id=4424d37c-e975-4a14-99f5-c7d702647239&category=GeneOntologyBiologicalProcess&id=GO:0033083) |
| 23 | GO:0010812 | negative regulation of cell-substrate adhesion |  | 5.15E-04 | 4.31E-02 | 3.53E-01 | 1.00E+00 | [3](http://toppgene.cchmc.org/showQueryTerms.jsp?userdata_id=4424d37c-e975-4a14-99f5-c7d702647239&feature=gop&row=22) | [37](http://toppgene.cchmc.org/showTermDetail.jsp?userdata_id=4424d37c-e975-4a14-99f5-c7d702647239&category=GeneOntologyBiologicalProcess&id=GO:0010812) |
| 24 | GO:0010627 | regulation of intracellular protein kinase cascade |  | 5.20E-04 | 4.31E-02 | 3.53E-01 | 1.00E+00 | [11](http://toppgene.cchmc.org/showQueryTerms.jsp?userdata_id=4424d37c-e975-4a14-99f5-c7d702647239&feature=gop&row=23) | [797](http://toppgene.cchmc.org/showTermDetail.jsp?userdata_id=4424d37c-e975-4a14-99f5-c7d702647239&category=GeneOntologyBiologicalProcess&id=GO:0010627) |
| 25 | GO:0033554 | cellular response to stress |  | 5.63E-04 | 4.31E-02 | 3.53E-01 | 1.00E+00 | [15](http://toppgene.cchmc.org/showQueryTerms.jsp?userdata_id=4424d37c-e975-4a14-99f5-c7d702647239&feature=gop&row=24) | [1370](http://toppgene.cchmc.org/showTermDetail.jsp?userdata_id=4424d37c-e975-4a14-99f5-c7d702647239&category=GeneOntologyBiologicalProcess&id=GO:0033554) |
| 26 | GO:0033079 | immature T cell proliferation |  | 6.28E-04 | 4.31E-02 | 3.53E-01 | 1.00E+00 | [2](http://toppgene.cchmc.org/showQueryTerms.jsp?userdata_id=4424d37c-e975-4a14-99f5-c7d702647239&feature=gop&row=25) | [9](http://toppgene.cchmc.org/showTermDetail.jsp?userdata_id=4424d37c-e975-4a14-99f5-c7d702647239&category=GeneOntologyBiologicalProcess&id=GO:0033079) |
| 27 | GO:0034063 | stress granule assembly |  | 6.28E-04 | 4.31E-02 | 3.53E-01 | 1.00E+00 | [2](http://toppgene.cchmc.org/showQueryTerms.jsp?userdata_id=4424d37c-e975-4a14-99f5-c7d702647239&feature=gop&row=26) | [9](http://toppgene.cchmc.org/showTermDetail.jsp?userdata_id=4424d37c-e975-4a14-99f5-c7d702647239&category=GeneOntologyBiologicalProcess&id=GO:0034063) |
| 28 | GO:0031657 | regulation of cyclin-dependent protein serine/threonine kinase activity involved in G1/S transition of mitotic cell cycle |  | 6.28E-04 | 4.31E-02 | 3.53E-01 | 1.00E+00 | [2](http://toppgene.cchmc.org/showQueryTerms.jsp?userdata_id=4424d37c-e975-4a14-99f5-c7d702647239&feature=gop&row=27) | [9](http://toppgene.cchmc.org/showTermDetail.jsp?userdata_id=4424d37c-e975-4a14-99f5-c7d702647239&category=GeneOntologyBiologicalProcess&id=GO:0031657) |
| 29 | GO:0010001 | glial cell differentiation |  | 6.29E-04 | 4.31E-02 | 3.53E-01 | 1.00E+00 | [5](http://toppgene.cchmc.org/showQueryTerms.jsp?userdata_id=4424d37c-e975-4a14-99f5-c7d702647239&feature=gop&row=28) | [162](http://toppgene.cchmc.org/showTermDetail.jsp?userdata_id=4424d37c-e975-4a14-99f5-c7d702647239&category=GeneOntologyBiologicalProcess&id=GO:0010001) |
| 30 | GO:0006974 | cellular response to DNA damage stimulus |  | 6.54E-04 | 4.31E-02 | 3.53E-01 | 1.00E+00 | [10](http://toppgene.cchmc.org/showQueryTerms.jsp?userdata_id=4424d37c-e975-4a14-99f5-c7d702647239&feature=gop&row=29) | [690](http://toppgene.cchmc.org/showTermDetail.jsp?userdata_id=4424d37c-e975-4a14-99f5-c7d702647239&category=GeneOntologyBiologicalProcess&id=GO:0006974) |
| 31 | GO:0045786 | negative regulation of cell cycle |  | 6.85E-04 | 4.31E-02 | 3.53E-01 | 1.00E+00 | [7](http://toppgene.cchmc.org/showQueryTerms.jsp?userdata_id=4424d37c-e975-4a14-99f5-c7d702647239&feature=gop&row=30) | [348](http://toppgene.cchmc.org/showTermDetail.jsp?userdata_id=4424d37c-e975-4a14-99f5-c7d702647239&category=GeneOntologyBiologicalProcess&id=GO:0045786) |
| 32 | GO:0010558 | negative regulation of macromolecule biosynthetic process |  | 6.89E-04 | 4.31E-02 | 3.53E-01 | 1.00E+00 | [13](http://toppgene.cchmc.org/showQueryTerms.jsp?userdata_id=4424d37c-e975-4a14-99f5-c7d702647239&feature=gop&row=31) | [1101](http://toppgene.cchmc.org/showTermDetail.jsp?userdata_id=4424d37c-e975-4a14-99f5-c7d702647239&category=GeneOntologyBiologicalProcess&id=GO:0010558) |
| 33 | GO:0050765 | negative regulation of phagocytosis |  | 7.83E-04 | 4.75E-02 | 3.89E-01 | 1.00E+00 | [2](http://toppgene.cchmc.org/showQueryTerms.jsp?userdata_id=4424d37c-e975-4a14-99f5-c7d702647239&feature=gop&row=32) | [10](http://toppgene.cchmc.org/showTermDetail.jsp?userdata_id=4424d37c-e975-4a14-99f5-c7d702647239&category=GeneOntologyBiologicalProcess&id=GO:0050765) |

| Pathway | ID | Name | Source | pValue | FDR B&H | FDR B&Y | Bonferroni | Genes from Input | Genes in Annotation |
| --- | --- | --- | --- | --- | --- | --- | --- | --- | --- |
| 1 | 672458 | Signaling Pathways in Glioblastoma | [BioSystems: WikiPathways](http://www.ncbi.nlm.nih.gov/biosystems/672458) | 6.04E-07 | 3.49E-04 | 2.42E-03 | 3.49E-04 | [6](http://toppgene.cchmc.org/showQueryTerms.jsp?userdata_id=4424d37c-e975-4a14-99f5-c7d702647239&feature=pt&row=0) | [80](http://toppgene.cchmc.org/showTermDetail.jsp?userdata_id=4424d37c-e975-4a14-99f5-c7d702647239&category=Pathway&id=672458) |
| 2 | 83110 | Glioma | [BioSystems: KEGG](http://www.ncbi.nlm.nih.gov/biosystems/83110) | 5.01E-06 | 1.45E-03 | 1.00E-02 | 2.89E-03 | [5](http://toppgene.cchmc.org/showQueryTerms.jsp?userdata_id=4424d37c-e975-4a14-99f5-c7d702647239&feature=pt&row=1) | [65](http://toppgene.cchmc.org/showTermDetail.jsp?userdata_id=4424d37c-e975-4a14-99f5-c7d702647239&category=Pathway&id=83110) |
| 3 | 83114 | Melanoma | [BioSystems: KEGG](http://www.ncbi.nlm.nih.gov/biosystems/83114) | 7.76E-06 | 1.49E-03 | 1.04E-02 | 4.48E-03 | [5](http://toppgene.cchmc.org/showQueryTerms.jsp?userdata_id=4424d37c-e975-4a14-99f5-c7d702647239&feature=pt&row=2) | [71](http://toppgene.cchmc.org/showTermDetail.jsp?userdata_id=4424d37c-e975-4a14-99f5-c7d702647239&category=Pathway&id=83114) |
| 4 | 477120 | Signaling by SCF-KIT | [BioSystems: REACTOME](http://www.ncbi.nlm.nih.gov/biosystems/477120) | 1.46E-05 | 1.73E-03 | 1.20E-02 | 8.44E-03 | [6](http://toppgene.cchmc.org/showQueryTerms.jsp?userdata_id=4424d37c-e975-4a14-99f5-c7d702647239&feature=pt&row=3) | [138](http://toppgene.cchmc.org/showTermDetail.jsp?userdata_id=4424d37c-e975-4a14-99f5-c7d702647239&category=Pathway&id=477120) |
| 5 | 685535 | Constitutive PI3K/AKT Signaling in Cancer | [BioSystems: REACTOME](http://www.ncbi.nlm.nih.gov/biosystems/685535) | 2.10E-05 | 1.73E-03 | 1.20E-02 | 1.22E-02 | [5](http://toppgene.cchmc.org/showQueryTerms.jsp?userdata_id=4424d37c-e975-4a14-99f5-c7d702647239&feature=pt&row=4) | [87](http://toppgene.cchmc.org/showTermDetail.jsp?userdata_id=4424d37c-e975-4a14-99f5-c7d702647239&category=Pathway&id=685535) |
| 6 | 83111 | Prostate cancer | [BioSystems: KEGG](http://www.ncbi.nlm.nih.gov/biosystems/83111) | 2.35E-05 | 1.73E-03 | 1.20E-02 | 1.36E-02 | [5](http://toppgene.cchmc.org/showQueryTerms.jsp?userdata_id=4424d37c-e975-4a14-99f5-c7d702647239&feature=pt&row=5) | [89](http://toppgene.cchmc.org/showTermDetail.jsp?userdata_id=4424d37c-e975-4a14-99f5-c7d702647239&category=Pathway&id=83111) |
| 7 | 106385 | Downstream signal transduction | [BioSystems: REACTOME](http://www.ncbi.nlm.nih.gov/biosystems/106385) | 2.93E-05 | 1.73E-03 | 1.20E-02 | 1.69E-02 | [6](http://toppgene.cchmc.org/showQueryTerms.jsp?userdata_id=4424d37c-e975-4a14-99f5-c7d702647239&feature=pt&row=6) | [156](http://toppgene.cchmc.org/showTermDetail.jsp?userdata_id=4424d37c-e975-4a14-99f5-c7d702647239&category=Pathway&id=106385) |
| 8 | 530734 | Signaling by ERBB2 | [BioSystems: REACTOME](http://www.ncbi.nlm.nih.gov/biosystems/530734) | 3.26E-05 | 1.73E-03 | 1.20E-02 | 1.89E-02 | [6](http://toppgene.cchmc.org/showQueryTerms.jsp?userdata_id=4424d37c-e975-4a14-99f5-c7d702647239&feature=pt&row=7) | [159](http://toppgene.cchmc.org/showTermDetail.jsp?userdata_id=4424d37c-e975-4a14-99f5-c7d702647239&category=Pathway&id=530734) |
| 9 | 685534 | PI3K/AKT Signaling in Cancer | [BioSystems: REACTOME](http://www.ncbi.nlm.nih.gov/biosystems/685534) | 3.93E-05 | 1.73E-03 | 1.20E-02 | 2.27E-02 | [5](http://toppgene.cchmc.org/showQueryTerms.jsp?userdata_id=4424d37c-e975-4a14-99f5-c7d702647239&feature=pt&row=8) | [99](http://toppgene.cchmc.org/showTermDetail.jsp?userdata_id=4424d37c-e975-4a14-99f5-c7d702647239&category=Pathway&id=685534) |
| 10 | 530737 | PI3K events in ERBB2 signaling | [BioSystems: REACTOME](http://www.ncbi.nlm.nih.gov/biosystems/530737) | 3.93E-05 | 1.73E-03 | 1.20E-02 | 2.27E-02 | [5](http://toppgene.cchmc.org/showQueryTerms.jsp?userdata_id=4424d37c-e975-4a14-99f5-c7d702647239&feature=pt&row=9) | [99](http://toppgene.cchmc.org/showTermDetail.jsp?userdata_id=4424d37c-e975-4a14-99f5-c7d702647239&category=Pathway&id=530737) |
| 11 | 366239 | PIP3 activates AKT signaling | [BioSystems: REACTOME](http://www.ncbi.nlm.nih.gov/biosystems/366239) | 3.93E-05 | 1.73E-03 | 1.20E-02 | 2.27E-02 | [5](http://toppgene.cchmc.org/showQueryTerms.jsp?userdata_id=4424d37c-e975-4a14-99f5-c7d702647239&feature=pt&row=10) | [99](http://toppgene.cchmc.org/showTermDetail.jsp?userdata_id=4424d37c-e975-4a14-99f5-c7d702647239&category=Pathway&id=366239) |
| 12 | 530743 | PI3K events in ERBB4 signaling | [BioSystems: REACTOME](http://www.ncbi.nlm.nih.gov/biosystems/530743) | 3.93E-05 | 1.73E-03 | 1.20E-02 | 2.27E-02 | [5](http://toppgene.cchmc.org/showQueryTerms.jsp?userdata_id=4424d37c-e975-4a14-99f5-c7d702647239&feature=pt&row=11) | [99](http://toppgene.cchmc.org/showTermDetail.jsp?userdata_id=4424d37c-e975-4a14-99f5-c7d702647239&category=Pathway&id=530743) |
| 13 | 160961 | PI-3K cascade | [BioSystems: REACTOME](http://www.ncbi.nlm.nih.gov/biosystems/160961) | 3.93E-05 | 1.73E-03 | 1.20E-02 | 2.27E-02 | [5](http://toppgene.cchmc.org/showQueryTerms.jsp?userdata_id=4424d37c-e975-4a14-99f5-c7d702647239&feature=pt&row=12) | [99](http://toppgene.cchmc.org/showTermDetail.jsp?userdata_id=4424d37c-e975-4a14-99f5-c7d702647239&category=Pathway&id=160961) |
| 14 | 106474 | PI3K/AKT activation | [BioSystems: REACTOME](http://www.ncbi.nlm.nih.gov/biosystems/106474) | 4.54E-05 | 1.73E-03 | 1.20E-02 | 2.62E-02 | [5](http://toppgene.cchmc.org/showQueryTerms.jsp?userdata_id=4424d37c-e975-4a14-99f5-c7d702647239&feature=pt&row=13) | [102](http://toppgene.cchmc.org/showTermDetail.jsp?userdata_id=4424d37c-e975-4a14-99f5-c7d702647239&category=Pathway&id=106474) |
| 15 | 106341 | GAB1 signalosome | [BioSystems: REACTOME](http://www.ncbi.nlm.nih.gov/biosystems/106341) | 4.76E-05 | 1.73E-03 | 1.20E-02 | 2.75E-02 | [5](http://toppgene.cchmc.org/showQueryTerms.jsp?userdata_id=4424d37c-e975-4a14-99f5-c7d702647239&feature=pt&row=14) | [103](http://toppgene.cchmc.org/showTermDetail.jsp?userdata_id=4424d37c-e975-4a14-99f5-c7d702647239&category=Pathway&id=106341) |
| 16 | 83109 | Endometrial cancer | [BioSystems: KEGG](http://www.ncbi.nlm.nih.gov/biosystems/83109) | 4.80E-05 | 1.73E-03 | 1.20E-02 | 2.78E-02 | [4](http://toppgene.cchmc.org/showQueryTerms.jsp?userdata_id=4424d37c-e975-4a14-99f5-c7d702647239&feature=pt&row=15) | [52](http://toppgene.cchmc.org/showTermDetail.jsp?userdata_id=4424d37c-e975-4a14-99f5-c7d702647239&category=Pathway&id=83109) |
| 17 | 645274 | Signaling by FGFR in disease | [BioSystems: REACTOME](http://www.ncbi.nlm.nih.gov/biosystems/645274) | 5.24E-05 | 1.78E-03 | 1.24E-02 | 3.03E-02 | [6](http://toppgene.cchmc.org/showQueryTerms.jsp?userdata_id=4424d37c-e975-4a14-99f5-c7d702647239&feature=pt&row=16) | [173](http://toppgene.cchmc.org/showTermDetail.jsp?userdata_id=4424d37c-e975-4a14-99f5-c7d702647239&category=Pathway&id=645274) |
| 18 | 106384 | Signaling by PDGF | [BioSystems: REACTOME](http://www.ncbi.nlm.nih.gov/biosystems/106384) | 6.33E-05 | 1.96E-03 | 1.36E-02 | 3.66E-02 | [6](http://toppgene.cchmc.org/showQueryTerms.jsp?userdata_id=4424d37c-e975-4a14-99f5-c7d702647239&feature=pt&row=17) | [179](http://toppgene.cchmc.org/showTermDetail.jsp?userdata_id=4424d37c-e975-4a14-99f5-c7d702647239&category=Pathway&id=106384) |
| 19 | 83119 | Non-small cell lung cancer | [BioSystems: KEGG](http://www.ncbi.nlm.nih.gov/biosystems/83119) | 6.44E-05 | 1.96E-03 | 1.36E-02 | 3.72E-02 | [4](http://toppgene.cchmc.org/showQueryTerms.jsp?userdata_id=4424d37c-e975-4a14-99f5-c7d702647239&feature=pt&row=18) | [56](http://toppgene.cchmc.org/showTermDetail.jsp?userdata_id=4424d37c-e975-4a14-99f5-c7d702647239&category=Pathway&id=83119) |
| 20 | 711360 | Integrated Pancreatic Cancer Pathway | [BioSystems: WikiPathways](http://www.ncbi.nlm.nih.gov/biosystems/711360) | 9.89E-05 | 2.86E-03 | 1.98E-02 | 5.72E-02 | [6](http://toppgene.cchmc.org/showQueryTerms.jsp?userdata_id=4424d37c-e975-4a14-99f5-c7d702647239&feature=pt&row=19) | [194](http://toppgene.cchmc.org/showTermDetail.jsp?userdata_id=4424d37c-e975-4a14-99f5-c7d702647239&category=Pathway&id=711360) |
| 21 | 833829 | Role of LAT2/NTAL/LAB on calcium mobilization | [BioSystems: REACTOME](http://www.ncbi.nlm.nih.gov/biosystems/833829) | 1.15E-04 | 3.17E-03 | 2.20E-02 | 6.66E-02 | [5](http://toppgene.cchmc.org/showQueryTerms.jsp?userdata_id=4424d37c-e975-4a14-99f5-c7d702647239&feature=pt&row=20) | [124](http://toppgene.cchmc.org/showTermDetail.jsp?userdata_id=4424d37c-e975-4a14-99f5-c7d702647239&category=Pathway&id=833829) |
| 22 | 83108 | Pancreatic cancer | [BioSystems: KEGG](http://www.ncbi.nlm.nih.gov/biosystems/83108) | 1.23E-04 | 3.23E-03 | 2.24E-02 | 7.10E-02 | [4](http://toppgene.cchmc.org/showQueryTerms.jsp?userdata_id=4424d37c-e975-4a14-99f5-c7d702647239&feature=pt&row=21) | [66](http://toppgene.cchmc.org/showTermDetail.jsp?userdata_id=4424d37c-e975-4a14-99f5-c7d702647239&category=Pathway&id=83108) |
| 23 | P00030 | Hypoxia response via HIF activation | [PantherDB](https://toppgene.cchmc.org/output.jsp?userdata_id=4424d37c-e975-4a14-99f5-c7d702647239) | 1.36E-04 | 3.43E-03 | 2.38E-02 | 7.89E-02 | [3](http://toppgene.cchmc.org/showQueryTerms.jsp?userdata_id=4424d37c-e975-4a14-99f5-c7d702647239&feature=pt&row=22) | [26](http://toppgene.cchmc.org/showTermDetail.jsp?userdata_id=4424d37c-e975-4a14-99f5-c7d702647239&category=Pathway&id=P00030) |
| 24 | PW:0000622 | altered phosphatodylinositol 3-kinase-Akt signaling | [Pathway Ontology](http://rgd.mcw.edu/rgdweb/ontology/view.html?acc_id=PW:0000622) | 1.49E-04 | 3.44E-03 | 2.38E-02 | 8.59E-02 | [2](http://toppgene.cchmc.org/showQueryTerms.jsp?userdata_id=4424d37c-e975-4a14-99f5-c7d702647239&feature=pt&row=23) | [5](http://toppgene.cchmc.org/showTermDetail.jsp?userdata_id=4424d37c-e975-4a14-99f5-c7d702647239&category=Pathway&id=PW:0000622) |
| 25 | 530739 | GRB7 events in ERBB2 signaling | [BioSystems: REACTOME](http://www.ncbi.nlm.nih.gov/biosystems/530739) | 1.49E-04 | 3.44E-03 | 2.38E-02 | 8.59E-02 | [2](http://toppgene.cchmc.org/showQueryTerms.jsp?userdata_id=4424d37c-e975-4a14-99f5-c7d702647239&feature=pt&row=24) | [5](http://toppgene.cchmc.org/showTermDetail.jsp?userdata_id=4424d37c-e975-4a14-99f5-c7d702647239&category=Pathway&id=530739) |
| 26 | 160957 | Downstream signaling of activated FGFR | [BioSystems: REACTOME](http://www.ncbi.nlm.nih.gov/biosystems/160957) | 2.32E-04 | 4.93E-03 | 3.42E-02 | 1.34E-01 | [5](http://toppgene.cchmc.org/showQueryTerms.jsp?userdata_id=4424d37c-e975-4a14-99f5-c7d702647239&feature=pt&row=25) | [144](http://toppgene.cchmc.org/showTermDetail.jsp?userdata_id=4424d37c-e975-4a14-99f5-c7d702647239&category=Pathway&id=160957) |
| 27 | 868085 | Ras signaling pathway | [BioSystems: KEGG](http://www.ncbi.nlm.nih.gov/biosystems/868085) | 2.34E-04 | 4.93E-03 | 3.42E-02 | 1.35E-01 | [6](http://toppgene.cchmc.org/showQueryTerms.jsp?userdata_id=4424d37c-e975-4a14-99f5-c7d702647239&feature=pt&row=26) | [227](http://toppgene.cchmc.org/showTermDetail.jsp?userdata_id=4424d37c-e975-4a14-99f5-c7d702647239&category=Pathway&id=868085) |
| 28 | P00059 | p53 pathway | [PantherDB](https://toppgene.cchmc.org/output.jsp?userdata_id=4424d37c-e975-4a14-99f5-c7d702647239) | 2.47E-04 | 4.93E-03 | 3.42E-02 | 1.43E-01 | [4](http://toppgene.cchmc.org/showQueryTerms.jsp?userdata_id=4424d37c-e975-4a14-99f5-c7d702647239&feature=pt&row=27) | [79](http://toppgene.cchmc.org/showTermDetail.jsp?userdata_id=4424d37c-e975-4a14-99f5-c7d702647239&category=Pathway&id=P00059) |
| 29 | 83105 | Pathways in cancer | [BioSystems: KEGG](http://www.ncbi.nlm.nih.gov/biosystems/83105) | 2.47E-04 | 4.93E-03 | 3.42E-02 | 1.43E-01 | [7](http://toppgene.cchmc.org/showQueryTerms.jsp?userdata_id=4424d37c-e975-4a14-99f5-c7d702647239&feature=pt&row=28) | [327](http://toppgene.cchmc.org/showTermDetail.jsp?userdata_id=4424d37c-e975-4a14-99f5-c7d702647239&category=Pathway&id=83105) |
| 30 | 530741 | Signaling by ERBB4 | [BioSystems: REACTOME](http://www.ncbi.nlm.nih.gov/biosystems/530741) | 2.72E-04 | 5.24E-03 | 3.64E-02 | 1.57E-01 | [5](http://toppgene.cchmc.org/showQueryTerms.jsp?userdata_id=4424d37c-e975-4a14-99f5-c7d702647239&feature=pt&row=29) | [149](http://toppgene.cchmc.org/showTermDetail.jsp?userdata_id=4424d37c-e975-4a14-99f5-c7d702647239&category=Pathway&id=530741) |
| 31 | M3270 | Integrin Signaling Pathway | [MSigDB C2: Signaling Transduction KE](http://www.broadinstitute.org/gsea/msigdb/cards/ST_INTEGRIN_SIGNALING_PATHWAY.html) | 2.85E-04 | 5.31E-03 | 3.69E-02 | 1.65E-01 | [4](http://toppgene.cchmc.org/showQueryTerms.jsp?userdata_id=4424d37c-e975-4a14-99f5-c7d702647239&feature=pt&row=30) | [82](http://toppgene.cchmc.org/showTermDetail.jsp?userdata_id=4424d37c-e975-4a14-99f5-c7d702647239&category=Pathway&id=M3270) |
| 32 | PW:0000232 | phosphatidylinositol 3-kinase-Akt signaling | [Pathway Ontology](http://rgd.mcw.edu/rgdweb/ontology/view.html?acc_id=PW:0000232) | 3.07E-04 | 5.55E-03 | 3.85E-02 | 1.78E-01 | [3](http://toppgene.cchmc.org/showQueryTerms.jsp?userdata_id=4424d37c-e975-4a14-99f5-c7d702647239&feature=pt&row=31) | [34](http://toppgene.cchmc.org/showTermDetail.jsp?userdata_id=4424d37c-e975-4a14-99f5-c7d702647239&category=Pathway&id=PW:0000232) |
| 33 | 106343 | Signaling by FGFR | [BioSystems: REACTOME](http://www.ncbi.nlm.nih.gov/biosystems/106343) | 3.57E-04 | 6.07E-03 | 4.21E-02 | 2.06E-01 | [5](http://toppgene.cchmc.org/showQueryTerms.jsp?userdata_id=4424d37c-e975-4a14-99f5-c7d702647239&feature=pt&row=32) | [158](http://toppgene.cchmc.org/showTermDetail.jsp?userdata_id=4424d37c-e975-4a14-99f5-c7d702647239&category=Pathway&id=106343) |
| 34 | M1315 | Genes related to PIP3 signaling in B lymphocytes | [MSigDB C2: Signaling Gateway](http://www.broadinstitute.org/gsea/msigdb/cards/SIG_PIP3_SIGNALING_IN_B_LYMPHOCYTES.html) | 3.65E-04 | 6.07E-03 | 4.21E-02 | 2.11E-01 | [3](http://toppgene.cchmc.org/showQueryTerms.jsp?userdata_id=4424d37c-e975-4a14-99f5-c7d702647239&feature=pt&row=33) | [36](http://toppgene.cchmc.org/showTermDetail.jsp?userdata_id=4424d37c-e975-4a14-99f5-c7d702647239&category=Pathway&id=M1315) |
| 35 | 685550 | DAP12 signaling | [BioSystems: REACTOME](http://www.ncbi.nlm.nih.gov/biosystems/685550) | 3.68E-04 | 6.07E-03 | 4.21E-02 | 2.12E-01 | [5](http://toppgene.cchmc.org/showQueryTerms.jsp?userdata_id=4424d37c-e975-4a14-99f5-c7d702647239&feature=pt&row=34) | [159](http://toppgene.cchmc.org/showTermDetail.jsp?userdata_id=4424d37c-e975-4a14-99f5-c7d702647239&category=Pathway&id=685550) |
| 36 | M14532 | PI3K Pathway | [MSigDB C2: Signaling Transduction KE](http://www.broadinstitute.org/gsea/msigdb/cards/ST_PHOSPHOINOSITIDE_3_KINASE_PATHWAY.html) | 3.96E-04 | 6.35E-03 | 4.41E-02 | 2.29E-01 | [3](http://toppgene.cchmc.org/showQueryTerms.jsp?userdata_id=4424d37c-e975-4a14-99f5-c7d702647239&feature=pt&row=35) | [37](http://toppgene.cchmc.org/showTermDetail.jsp?userdata_id=4424d37c-e975-4a14-99f5-c7d702647239&category=Pathway&id=M14532) |
| 37 | 106477 | Negative regulation of the PI3K/AKT network | [BioSystems: REACTOME](http://www.ncbi.nlm.nih.gov/biosystems/106477) | 4.13E-04 | 6.35E-03 | 4.41E-02 | 2.39E-01 | [2](http://toppgene.cchmc.org/showQueryTerms.jsp?userdata_id=4424d37c-e975-4a14-99f5-c7d702647239&feature=pt&row=36) | [8](http://toppgene.cchmc.org/showTermDetail.jsp?userdata_id=4424d37c-e975-4a14-99f5-c7d702647239&category=Pathway&id=106477) |
| 38 | 198827 | DNA damage response (only ATM dependent) | [BioSystems: WikiPathways](http://www.ncbi.nlm.nih.gov/biosystems/198827) | 4.24E-04 | 6.35E-03 | 4.41E-02 | 2.45E-01 | [4](http://toppgene.cchmc.org/showQueryTerms.jsp?userdata_id=4424d37c-e975-4a14-99f5-c7d702647239&feature=pt&row=37) | [91](http://toppgene.cchmc.org/showTermDetail.jsp?userdata_id=4424d37c-e975-4a14-99f5-c7d702647239&category=Pathway&id=198827) |
| 39 | 83115 | Bladder cancer | [BioSystems: KEGG](http://www.ncbi.nlm.nih.gov/biosystems/83115) | 4.28E-04 | 6.35E-03 | 4.41E-02 | 2.48E-01 | [3](http://toppgene.cchmc.org/showQueryTerms.jsp?userdata_id=4424d37c-e975-4a14-99f5-c7d702647239&feature=pt&row=38) | [38](http://toppgene.cchmc.org/showTermDetail.jsp?userdata_id=4424d37c-e975-4a14-99f5-c7d702647239&category=Pathway&id=83115) |
| 40 | 83048 | MAPK signaling pathway | [BioSystems: KEGG](http://www.ncbi.nlm.nih.gov/biosystems/83048) | 4.73E-04 | 6.84E-03 | 4.75E-02 | 2.74E-01 | [6](http://toppgene.cchmc.org/showQueryTerms.jsp?userdata_id=4424d37c-e975-4a14-99f5-c7d702647239&feature=pt&row=39) | [259](http://toppgene.cchmc.org/showTermDetail.jsp?userdata_id=4424d37c-e975-4a14-99f5-c7d702647239&category=Pathway&id=83048) |
| 41 | 576250 | Downstream Signaling Events Of B Cell Receptor (BCR) | [BioSystems: REACTOME](http://www.ncbi.nlm.nih.gov/biosystems/576250) | 5.13E-04 | 7.23E-03 | 5.02E-02 | 2.97E-01 | [5](http://toppgene.cchmc.org/showQueryTerms.jsp?userdata_id=4424d37c-e975-4a14-99f5-c7d702647239&feature=pt&row=40) | [171](http://toppgene.cchmc.org/showTermDetail.jsp?userdata_id=4424d37c-e975-4a14-99f5-c7d702647239&category=Pathway&id=576250) |
| 42 | 645322 | Hydrolysis of LPC | [BioSystems: REACTOME](http://www.ncbi.nlm.nih.gov/biosystems/645322) | 5.30E-04 | 7.29E-03 | 5.06E-02 | 3.06E-01 | [2](http://toppgene.cchmc.org/showQueryTerms.jsp?userdata_id=4424d37c-e975-4a14-99f5-c7d702647239&feature=pt&row=41) | [9](http://toppgene.cchmc.org/showTermDetail.jsp?userdata_id=4424d37c-e975-4a14-99f5-c7d702647239&category=Pathway&id=645322) |
| 43 | 106337 | Signaling by EGFR | [BioSystems: REACTOME](http://www.ncbi.nlm.nih.gov/biosystems/106337) | 5.85E-04 | 7.87E-03 | 5.46E-02 | 3.38E-01 | [5](http://toppgene.cchmc.org/showQueryTerms.jsp?userdata_id=4424d37c-e975-4a14-99f5-c7d702647239&feature=pt&row=42) | [176](http://toppgene.cchmc.org/showTermDetail.jsp?userdata_id=4424d37c-e975-4a14-99f5-c7d702647239&category=Pathway&id=106337) |
| 44 | 530768 | Signaling by EGFR in Cancer | [BioSystems: REACTOME](http://www.ncbi.nlm.nih.gov/biosystems/530768) | 6.16E-04 | 8.09E-03 | 5.61E-02 | 3.56E-01 | [5](http://toppgene.cchmc.org/showQueryTerms.jsp?userdata_id=4424d37c-e975-4a14-99f5-c7d702647239&feature=pt&row=43) | [178](http://toppgene.cchmc.org/showTermDetail.jsp?userdata_id=4424d37c-e975-4a14-99f5-c7d702647239&category=Pathway&id=530768) |
| 45 | 685549 | DAP12 interactions | [BioSystems: REACTOME](http://www.ncbi.nlm.nih.gov/biosystems/685549) | 6.32E-04 | 8.12E-03 | 5.63E-02 | 3.65E-01 | [5](http://toppgene.cchmc.org/showQueryTerms.jsp?userdata_id=4424d37c-e975-4a14-99f5-c7d702647239&feature=pt&row=44) | [179](http://toppgene.cchmc.org/showTermDetail.jsp?userdata_id=4424d37c-e975-4a14-99f5-c7d702647239&category=Pathway&id=685549) |
| 46 | 198795 | Focal Adhesion | [BioSystems: WikiPathways](http://www.ncbi.nlm.nih.gov/biosystems/198795) | 7.34E-04 | 9.22E-03 | 6.40E-02 | 4.24E-01 | [5](http://toppgene.cchmc.org/showQueryTerms.jsp?userdata_id=4424d37c-e975-4a14-99f5-c7d702647239&feature=pt&row=45) | [185](http://toppgene.cchmc.org/showTermDetail.jsp?userdata_id=4424d37c-e975-4a14-99f5-c7d702647239&category=Pathway&id=198795) |
| 47 | 695200 | HIF-1 signaling pathway | [BioSystems: KEGG](http://www.ncbi.nlm.nih.gov/biosystems/695200) | 7.56E-04 | 9.27E-03 | 6.43E-02 | 4.37E-01 | [4](http://toppgene.cchmc.org/showQueryTerms.jsp?userdata_id=4424d37c-e975-4a14-99f5-c7d702647239&feature=pt&row=46) | [106](http://toppgene.cchmc.org/showTermDetail.jsp?userdata_id=4424d37c-e975-4a14-99f5-c7d702647239&category=Pathway&id=695200) |
| 48 | 106439 | Signalling by NGF | [BioSystems: REACTOME](http://www.ncbi.nlm.nih.gov/biosystems/106439) | 7.70E-04 | 9.27E-03 | 6.43E-02 | 4.45E-01 | [6](http://toppgene.cchmc.org/showQueryTerms.jsp?userdata_id=4424d37c-e975-4a14-99f5-c7d702647239&feature=pt&row=47) | [284](http://toppgene.cchmc.org/showTermDetail.jsp?userdata_id=4424d37c-e975-4a14-99f5-c7d702647239&category=Pathway&id=106439) |
| 49 | 833825 | Fc epsilon receptor (FCERI) signaling | [BioSystems: REACTOME](http://www.ncbi.nlm.nih.gov/biosystems/833825) | 9.09E-04 | 1.07E-02 | 7.44E-02 | 5.25E-01 | [5](http://toppgene.cchmc.org/showQueryTerms.jsp?userdata_id=4424d37c-e975-4a14-99f5-c7d702647239&feature=pt&row=48) | [194](http://toppgene.cchmc.org/showTermDetail.jsp?userdata_id=4424d37c-e975-4a14-99f5-c7d702647239&category=Pathway&id=833825) |
| 50 | M7955 | Genes related to the insulin receptor pathway | [MSigDB C2: Signaling Gateway](http://www.broadinstitute.org/gsea/msigdb/cards/SIG_INSULIN_RECEPTOR_PATHWAY_IN_CARDIAC_MYOCYTES.html) | 1.02E-03 | 1.18E-02 | 8.19E-02 | 5.90E-01 | [3](http://toppgene.cchmc.org/showQueryTerms.jsp?userdata_id=4424d37c-e975-4a14-99f5-c7d702647239&feature=pt&row=49) | [51](http://toppgene.cchmc.org/showTermDetail.jsp?userdata_id=4424d37c-e975-4a14-99f5-c7d702647239&category=Pathway&id=M7955) |

E IDH mutant, grade II-III vs GBM

| GO | ID | Name | Source | pValue | FDR B&H | FDR B&Y | Bonferroni | Genes from Input | Genes in Annotation |
| --- | --- | --- | --- | --- | --- | --- | --- | --- | --- |
| 1 | GO:0071705 | nitrogen compound transport |  | 2.40E-05 | 4.44E-02 | 3.60E-01 | 4.44E-02 | [13](http://toppgene.cchmc.org/showQueryTerms.jsp?userdata_id=d453bf9e-2657-42bc-948f-53b6aeb93052&feature=gop&row=0) | [682](http://toppgene.cchmc.org/showTermDetail.jsp?userdata_id=d453bf9e-2657-42bc-948f-53b6aeb93052&category=GeneOntologyBiologicalProcess&id=GO:0071705) |

F IDH wild-type, grade II-III vs GBM

| GO | ID | Name | Source | pValue | FDR B&H | FDR B&Y | Bonferroni | Genes from Input | Genes in Annotation |
| --- | --- | --- | --- | --- | --- | --- | --- | --- | --- |
| 1 | GO:0006284 | base-excision repair |  | 5.93E-07 | 1.79E-03 | 1.54E-02 | 1.79E-03 | [7](http://toppgene.cchmc.org/showQueryTerms.jsp?userdata_id=667ce17e-82ba-4110-82f4-8e5fcde34b0d&feature=gop&row=0) | [40](http://toppgene.cchmc.org/showTermDetail.jsp?userdata_id=667ce17e-82ba-4110-82f4-8e5fcde34b0d&category=GeneOntologyBiologicalProcess&id=GO:0006284) |
| 2 | GO:0006706 | steroid catabolic process |  | 1.59E-05 | 2.41E-02 | 2.07E-01 | 4.82E-02 | [5](http://toppgene.cchmc.org/showQueryTerms.jsp?userdata_id=667ce17e-82ba-4110-82f4-8e5fcde34b0d&feature=gop&row=1) | [26](http://toppgene.cchmc.org/showTermDetail.jsp?userdata_id=667ce17e-82ba-4110-82f4-8e5fcde34b0d&category=GeneOntologyBiologicalProcess&id=GO:0006706) |

| Pathway | ID | Name | Source | pValue | FDR B&H | FDR B&Y | Bonferroni | Genes from Input | Genes in Annotation |
| --- | --- | --- | --- | --- | --- | --- | --- | --- | --- |
| 1 | 83043 | Base excision repair | [BioSystems: KEGG](http://www.ncbi.nlm.nih.gov/biosystems/83043) | 3.68E-06 | 4.01E-03 | 3.03E-02 | 4.01E-03 | [6](http://toppgene.cchmc.org/showQueryTerms.jsp?userdata_id=667ce17e-82ba-4110-82f4-8e5fcde34b0d&feature=pt&row=0) | [33](http://toppgene.cchmc.org/showTermDetail.jsp?userdata_id=667ce17e-82ba-4110-82f4-8e5fcde34b0d&category=Pathway&id=83043) |
| 2 | 105837 | DNA Repair | [BioSystems: REACTOME](http://www.ncbi.nlm.nih.gov/biosystems/105837) | 1.61E-05 | 8.75E-03 | 6.63E-02 | 1.75E-02 | [9](http://toppgene.cchmc.org/showQueryTerms.jsp?userdata_id=667ce17e-82ba-4110-82f4-8e5fcde34b0d&feature=pt&row=1) | [113](http://toppgene.cchmc.org/showTermDetail.jsp?userdata_id=667ce17e-82ba-4110-82f4-8e5fcde34b0d&category=Pathway&id=105837) |
| 3 | 106541 | Extension of Telomeres | [BioSystems: REACTOME](http://www.ncbi.nlm.nih.gov/biosystems/106541) | 2.72E-05 | 9.88E-03 | 7.48E-02 | 2.96E-02 | [5](http://toppgene.cchmc.org/showQueryTerms.jsp?userdata_id=667ce17e-82ba-4110-82f4-8e5fcde34b0d&feature=pt&row=2) | [28](http://toppgene.cchmc.org/showTermDetail.jsp?userdata_id=667ce17e-82ba-4110-82f4-8e5fcde34b0d&category=Pathway&id=106541) |
| 4 | 105884 | Global Genomic NER (GG-NER) | [BioSystems: REACTOME](http://www.ncbi.nlm.nih.gov/biosystems/105884) | 7.24E-05 | 1.68E-02 | 1.27E-01 | 7.88E-02 | [5](http://toppgene.cchmc.org/showQueryTerms.jsp?userdata_id=667ce17e-82ba-4110-82f4-8e5fcde34b0d&feature=pt&row=3) | [34](http://toppgene.cchmc.org/showTermDetail.jsp?userdata_id=667ce17e-82ba-4110-82f4-8e5fcde34b0d&category=Pathway&id=105884) |
| 5 | 105846 | Resolution of Abasic Sites (AP sites) | [BioSystems: REACTOME](http://www.ncbi.nlm.nih.gov/biosystems/105846) | 9.26E-05 | 1.68E-02 | 1.27E-01 | 1.01E-01 | [4](http://toppgene.cchmc.org/showQueryTerms.jsp?userdata_id=667ce17e-82ba-4110-82f4-8e5fcde34b0d&feature=pt&row=4) | [19](http://toppgene.cchmc.org/showTermDetail.jsp?userdata_id=667ce17e-82ba-4110-82f4-8e5fcde34b0d&category=Pathway&id=105846) |
| 6 | 105838 | Base Excision Repair | [BioSystems: REACTOME](http://www.ncbi.nlm.nih.gov/biosystems/105838) | 9.26E-05 | 1.68E-02 | 1.27E-01 | 1.01E-01 | [4](http://toppgene.cchmc.org/showQueryTerms.jsp?userdata_id=667ce17e-82ba-4110-82f4-8e5fcde34b0d&feature=pt&row=5) | [19](http://toppgene.cchmc.org/showTermDetail.jsp?userdata_id=667ce17e-82ba-4110-82f4-8e5fcde34b0d&category=Pathway&id=105838) |
| 7 | 106543 | Telomere C-strand (Lagging Strand) Synthesis | [BioSystems: REACTOME](http://www.ncbi.nlm.nih.gov/biosystems/106543) | 1.70E-04 | 2.64E-02 | 2.00E-01 | 1.85E-01 | [4](http://toppgene.cchmc.org/showQueryTerms.jsp?userdata_id=667ce17e-82ba-4110-82f4-8e5fcde34b0d&feature=pt&row=6) | [22](http://toppgene.cchmc.org/showTermDetail.jsp?userdata_id=667ce17e-82ba-4110-82f4-8e5fcde34b0d&category=Pathway&id=106543) |
| 8 | 105890 | Transcription-coupled NER (TC-NER) | [BioSystems: REACTOME](http://www.ncbi.nlm.nih.gov/biosystems/105890) | 3.15E-04 | 4.21E-02 | 3.19E-01 | 3.42E-01 | [5](http://toppgene.cchmc.org/showQueryTerms.jsp?userdata_id=667ce17e-82ba-4110-82f4-8e5fcde34b0d&feature=pt&row=7) | [46](http://toppgene.cchmc.org/showTermDetail.jsp?userdata_id=667ce17e-82ba-4110-82f4-8e5fcde34b0d&category=Pathway&id=105890) |
| 9 | 83044 | Nucleotide excision repair | [BioSystems: KEGG](http://www.ncbi.nlm.nih.gov/biosystems/83044) | 3.49E-04 | 4.21E-02 | 3.19E-01 | 3.79E-01 | [5](http://toppgene.cchmc.org/showQueryTerms.jsp?userdata_id=667ce17e-82ba-4110-82f4-8e5fcde34b0d&feature=pt&row=8) | [47](http://toppgene.cchmc.org/showTermDetail.jsp?userdata_id=667ce17e-82ba-4110-82f4-8e5fcde34b0d&category=Pathway&id=83044) |
