# Supplementary material for: DNA copy number analysis of Grade II–III and Grade IV gliomas reveals differences in molecular ontogeny including chromothripsis associated with IDH mutation status
Source: Acta Neuropathol Commun. 2015 Jun 20;3:34. doi: 10.1186/s40478-015-0213-3 (PMC4474351; doi:10.1186/s40478-015-0213-3)
Supplement: Additional file 7: Table S7. — Univariate and multivariate survival analysis in the entire population and within the four subgroups of 1p/19q non-co-deleted gliomas. [file 40478_2015_213_MOESM7_ESM.doc]

A. Univariate Cox Proportional Hazard Analysis of All Gliomas

| **Effect** | **DF** | **Score** | **Pr > ChiSq** |
| --- | --- | --- | --- |
|  |  | **Chi-Square** |  |
| **IDH_mutant_2** | 1 | 93.6288 | <.0001 |
| **Grade** | 1 | 9.2667 | 0.0023 |
| **ANKRD30A** | 2 | 53.9092 | <.0001 |
| **AGAP2** | 2 | 1.9862 | 0.3704 |
| **PDGFRA** | 2 | 5.1171 | 0.0774 |
| **ESRRB** | 2 | 11.7836 | 0.0028 |
| **DUXA** | 2 | 8.4504 | 0.0146 |
| **cdk6** | 2 | 4.6152 | 0.0995 |
| **parp11** | 2 | 0.8189 | 0.664 |
| **park2** | 2 | 8.0228 | 0.0181 |
| **MIR4417** | 2 | 4.7088 | 0.095 |
| **mgmt** | 1 | 7.7195 | 0.0055 |
| **_p19qcodelete** | 1 | 3.2274 | 0.0724 |
| **CDKN2A** | 2 | 10.5036 | 0.0052 |
| **stringent_chromothri** | 1 | 0.9007 | 0.3426 |
| **Gender** | 2 | 3.6931 | 0.1578 |
| **chrX_40114495_401184** | 2 | 3.5024 | 0.1736 |
| **chrX_141365813_14188** | 2 | 3.1596 | 0.206 |
| **chr10_131901494_1319** | 1 | 7.0129 | 0.0081 |
| **chr13_48222949_48273** | 2 | 7.3343 | 0.0255 |
| **chr11_5279480_537067** | 2 | 7.5181 | 0.0233 |
| **chr4_190450033_19053** | 2 | 1.7551 | 0.4158 |
| **chr22_32874326_33313** | 2 | 0.7712 | 0.68 |
| **chr15_18421386_18507** | 2 | 1.7546 | 0.4159 |
| **chr1_202800228_20280** | 1 | 0.4414 | 0.5065 |
| **chr12_58840996_58859** | 2 | 5.6898 | 0.0581 |
| **chr2_16049873_161195** | 1 | 0.2461 | 0.6198 |
| **EGFR** | 1 | 25.8271 | <.0001 |
| **PTEN** | 2 | 25.1218 | <.0001 |
| **CDKN2C** | 2 | 0.4507 | 0.7982 |
| **CDKN1C** | 2 | 7.7595 | 0.0207 |
| **PDGFRB** | 2 | 0.6312 | 0.7294 |
| **PDGFA** | 2 | 13.7119 | 0.0011 |
| **PDGFB** | 2 | 1.4983 | 0.4728 |
| **NF1** | 2 | 7.3927 | 0.0248 |
| **TP53** | 2 | 20.202 | <.0001 |

B. Univariate Cox Proportional Hazard Analysis of All Gliomas

| **Parameter** |  | **DF** | **Parameter** | **Standard** | **Chi-Square** | **Pr > ChiSq** | **Hazard** |
| --- | --- | --- | --- | --- | --- | --- | --- |
|  |  |  | **Estimate** | **Error** |  |  | **Ratio** |
| **IDH_mutant_2** | **IDHmut** | 1 | -3.85132 | 0.50392 | 58.4106 | <.0001 | 0.021 |
| **Grade** | **IV** | 1 | 1.55822 | 0.35323 | 19.4603 | <.0001 | 4.75 |
| **ESRRB** | **1** | 1 | 0.71712 | 0.80961 | 0.7846 | 0.3757 | 2.049 |
| **ESRRB** | **-1** | 1 | 0.83899 | 0.28126 | 8.8979 | 0.0029 | 2.314 |
| **CDKN1C** | **CNGain** | 1 | 1.37878 | 0.58497 | 5.5555 | 0.0184 | 3.97 |
| **CDKN1C** | **CNLoss** | 1 | -0.31209 | 0.39013 | 0.6399 | 0.4237 | 0.732 |
| **TP53** | **CNGain** | 1 | -0.2564 | 0.32072 | 0.6391 | 0.424 | 0.774 |
| **TP53** | **CNLoss** | 1 | 1.80499 | 0.62868 | 8.243 | 0.0041 | 6.08 |

C. Univariate Cox Proportional Hazard Analysis of IDH Mutated Low Grade Gliomas

| **Effect** | **DF** | **Score** | **Pr > ChiSq** |
| --- | --- | --- | --- |
|  |  | **Chi-Square** |  |
| **ANKRD30A** | 1 | 14.5282 | 0.0001 |
| **AGAP2** | 2 | 0.6886 | 0.7087 |
| **PDGFRA** | 1 | 0.0004 | 0.9834 |
| **ESRRB** | 1 | 8.6022 | 0.0034 |
| **DUXA** | 1 | 0.0208 | 0.8852 |
| **cdk6** | 2 | 0.0316 | 0.9843 |
| **parp11** | 2 | 1.2021 | 0.5482 |
| **park2** | 1 | 5.5947 | 0.018 |
| **MIR4417** | 2 | 0.0139 | 0.9931 |
| **mgmt** | 1 | 1.5701 | 0.2102 |
| **_p19qcodelete** | 0 | . | . |
| **CDKN2A** | 2 | 4.7084 | 0.095 |
| **stringent_chromothri** | 1 | 0.8255 | 0.3636 |
| **Gender** | 1 | 0.4371 | 0.5085 |
| **chrX_40114495_401184** | 2 | 12.1509 | 0.0023 |
| **chrX_141365813_14188** | 2 | 5.2738 | 0.0716 |
| **chr10_131901494_1319** | 1 | 2.9137 | 0.0878 |
| **chr13_48222949_48273** | 1 | 2.2179 | 0.1364 |
| **chr11_5279480_537067** | 2 | 23.4584 | <.0001 |
| **chr4_190450033_19053** | 1 | 0.4717 | 0.4922 |
| **chr22_32874326_33313** | 1 | 1.3169 | 0.2511 |
| **chr15_18421386_18507** | 2 | 0.9336 | 0.627 |
| **chr1_202800228_20280** | 1 | 0.0107 | 0.9176 |
| **chr12_58840996_58859** | 2 | 2.0185 | 0.3645 |
| **chr2_16049873_161195** | 1 | 0.4867 | 0.4854 |
| **EGFR** | 1 | 1.0797 | 0.2988 |
| **PTEN** | 2 | 0.8278 | 0.6611 |
| **CDKN2C** | 2 | 26.1314 | <.0001 |
| **CDKN1C** | 2 | 22.8725 | <.0001 |
| **PDGFRB** | 2 | 1.1452 | 0.5641 |
| **PDGFA** | 2 | 0.2729 | 0.8725 |
| **PDGFB** | 2 | 1.2456 | 0.5364 |
| **NF1** | 1 | 1.6135 | 0.204 |
| **TP53** | 1 | 0.3341 | 0.5633 |

D. Multivariate Cox Proportional Hazard Analysis of IDH Mutated Low Grade Gliomas

| **Parameter** |  | **DF** | **Parameter** | **Standard** | **Chi-Square** | **Pr > ChiSq** | **Hazard** | **Label** |
| --- | --- | --- | --- | --- | --- | --- | --- | --- |
|  |  |  | **Estimate** | **Error** |  |  | **Ratio** |  |
| **ESRRB** | **-1** | 1 | 2.16664 | 0.85535 | 6.4164 | 0.0113 | 8.729 | ESRRB -1 |
| **chr10_131901494_1319** | **-1** | 1 | 2.69513 | 0.98848 | 7.434 | 0.0064 | 14.807 | chr10_131901494_131949667 -1 |
| **CDKN1C** | **CNGain** | 1 | 22.67417 | 51920 | 0 | 0.9997 | 7.04E+09 | CDKN1C CNGain |
| **CDKN1C** | **CNLoss** | 1 | -3.09791 | 1.25945 | 6.0503 | 0.0139 | 0.045 | CDKN1C CNLoss |

E. Univariate Cox Proportional Hazard Analysis of IDH Wild Type Low Grade Gliomas

| **Effect** | **DF** | **Score** | **Pr > ChiSq** |
| --- | --- | --- | --- |
|  |  | **Chi-Square** |  |
| **ANKRD30A** | 1 | 0.5622 | 0.4534 |
| **AGAP2** | 1 | 1.0378 | 0.3083 |
| **PDGFRA** | 1 | 0.3678 | 0.5442 |
| **ESRRB** | 1 | 2.6784 | 0.1017 |
| **DUXA** | 1 | 1.2236 | 0.2687 |
| **cdk6** | 1 | 1.1196 | 0.29 |
| **parp11** | 1 | 1.5112 | 0.219 |
| **park2** | 2 | 4.855 | 0.0883 |
| **MIR4417** | 2 | 2.3088 | 0.3152 |
| **mgmt** | 1 | 0.0531 | 0.8177 |
| **_p19qcodelete** | 0 | . | . |
| **CDKN2A** | 2 | 3.0053 | 0.2225 |
| **stringent_chromothri** | 1 | 0.0236 | 0.8778 |
| **Gender** | 1 | 0 | 0.9974 |
| **chrX_40114495_401184** | 1 | 0.0236 | 0.8778 |
| **chrX_141365813_14188** | 1 | 0.132 | 0.7163 |
| **chr10_131901494_1319** | 1 | 0.0531 | 0.8177 |
| **chr13_48222949_48273** | 1 | 0.5667 | 0.4516 |
| **chr11_5279480_537067** | 0 | . | . |
| **chr4_190450033_19053** | 1 | 1.2247 | 0.2684 |
| **chr22_32874326_33313** | 2 | 6.6874 | 0.0353 |
| **chr15_18421386_18507** | 1 | 8.8929 | 0.0029 |
| **chr1_202800228_20280** | 1 | 0.3968 | 0.5288 |
| **chr12_58840996_58859** | 2 | 0.6587 | 0.7194 |
| **chr2_16049873_161195** | 1 | 0.1545 | 0.6943 |
| **EGFR** | 1 | 0.079 | 0.7787 |
| **PTEN** | 1 | 0.4141 | 0.5199 |
| **CDKN2C** | 1 | 16 | <.0001 |
| **CDKN1C** | 1 | 0.2696 | 0.6036 |
| **PDGFRB** | 1 | 0.0828 | 0.7735 |
| **PDGFA** | 1 | 0.079 | 0.7787 |
| **PDGFB** | 2 | 3.2801 | 0.194 |
| **NF1** | 2 | 2.4848 | 0.2887 |
| **TP53** | 1 | 0.3012 | 0.5831 |

F. Multivariate Cox Proportional Hazard Analysis of IDH Wild Type Low Grade Gliomas

| **Parameter** |  | **DF** | **Parameter** | **Standard** | **Chi-Square** | **Pr > ChiSq** | **Hazard** | **Label** |
| --- | --- | --- | --- | --- | --- | --- | --- | --- |
|  |  |  | **Estimate** | **Error** |  |  | **Ratio** |  |
| **chr15_18421386_18507** | **-1** | 1 | 1.90258 | 0.72705 | 6.8479 | 0.0089 | 6.703 | chr15_18421386_18507053 -1 |

G. Univariate Cox Proportional Hazard Analysis of IDH Mutated Grade IV Gliomas

| **Effect** | **DF** | **Score** | **Pr > ChiSq** |
| --- | --- | --- | --- |
|  |  | **Chi-Square** |  |
| **ANKRD30A** | 2 | 1.6836 | 0.4309 |
| **AGAP2** | 1 | 0.0099 | 0.9206 |
| **PDGFRA** | 1 | 0.0841 | 0.7718 |
| **ESRRB** | 2 | 2.3211 | 0.3133 |
| **DUXA** | 2 | 1.3585 | 0.507 |
| **cdk6** | 1 | 0.479 | 0.4889 |
| **parp11** | 2 | 4.666 | 0.097 |
| **park2** | 2 | 4.6062 | 0.0999 |
| **MIR4417** | 2 | 3.0519 | 0.2174 |
| **mgmt** | 1 | 0.197 | 0.6571 |
| **_p19qcodelete** | 0 | . | . |
| **CDKN2A** | 2 | 2.1247 | 0.3456 |
| **stringent_chromothri** | 1 | 0.4616 | 0.4969 |
| **Gender** | 2 | 6.1081 | 0.0472 |
| **chrX_40114495_401184** | 1 | 0.9624 | 0.3266 |
| **chrX_141365813_14188** | 2 | 2.9017 | 0.2344 |
| **chr10_131901494_1319** | 1 | 3.5366 | 0.06 |
| **chr13_48222949_48273** | 1 | 2.5842 | 0.1079 |
| **chr11_5279480_537067** | 1 | 0.5612 | 0.4538 |
| **chr4_190450033_19053** | 2 | 0.3965 | 0.8202 |
| **chr22_32874326_33313** | 1 | 0.496 | 0.4813 |
| **chr15_18421386_18507** | 2 | 1.4428 | 0.4861 |
| **chr1_202800228_20280** | 1 | 0.6042 | 0.437 |
| **chr12_58840996_58859** | 2 | 2.5451 | 0.2801 |
| **chr2_16049873_161195** | 1 | 0.6321 | 0.4266 |
| **EGFR** | 1 | 1.6134 | 0.204 |
| **PTEN** | 1 | 0.7297 | 0.393 |
| **CDKN2C** | 2 | 0.0365 | 0.9819 |
| **CDKN1C** | 1 | 0.0389 | 0.8437 |
| **PDGFRB** | 2 | 1.3676 | 0.5047 |
| **PDGFA** | 1 | 1.4209 | 0.2333 |
| **PDGFB** | 2 | 1.3421 | 0.5112 |
| **NF1** | 1 | 0.2496 | 0.6174 |
| **TP53** | 1 | 0.0316 | 0.8589 |

H. Multivariate Cox Proportional Hazard Analysis of IDH Mutated grade IV Gliomas

| **Parameter** |  | **DF** | **Parameter** | **Standard** | **Chi-Square** | **Pr > ChiSq** | **Hazard** | **Label** |
| --- | --- | --- | --- | --- | --- | --- | --- | --- |
|  |  |  | **Estimate** | **Error** |  |  | **Ratio** |  |
| **park2** | **1** | 1 | 1.20107 | 1.18252 | 1.0316 | 0.3098 | 3.324 | park2 1 |
| **park2** | **-1** | 1 | 2.45952 | 0.96163 | 6.5416 | 0.0105 | 11.699 | park2 -1 |
| **Gender** | **Male** | 1 | 2.74922 | 1.0549 | 6.792 | 0.0092 | 15.63 | Gender Male |
| **Gender** | **Unknown** | 1 | 0.49445 | 1.25374 | 0.1555 | 0.6933 | 1.64 | Gender Unknown |

I. Univariate Cox Proportional Hazard Analysis of IDH Wild Type Grade IV Gliomas

| **Effect** | **DF** | **Score** | **Pr > ChiSq** |
| --- | --- | --- | --- |
|  |  | **Chi-Square** |  |
| **ANKRD30A** | 1 | 0.0864 | 0.7688 |
| **AGAP2** | 1 | 0.0657 | 0.7977 |
| **PDGFRA** | 1 | 0.599 | 0.439 |
| **ESRRB** | 1 | 0.6067 | 0.436 |
| **DUXA** | 2 | 3.7678 | 0.152 |
| **cdk6** | 1 | 0.2581 | 0.6114 |
| **parp11** | 1 | 1.5072 | 0.2196 |
| **park2** | 1 | 0.1943 | 0.6594 |
| **MIR4417** | 2 | 2.4716 | 0.2906 |
| **mgmt** | 1 | 0.8915 | 0.3451 |
| **_p19qcodelete** | 0 | . | . |
| **CDKN2A** | 2 | 0.8758 | 0.6454 |
| **stringent_chromothri** | 1 | 0.7368 | 0.3907 |
| **Gender** | 2 | 6.0139 | 0.0494 |
| **chrX_40114495_401184** | 1 | 1.5072 | 0.2196 |
| **chrX_141365813_14188** | 1 | 1.5072 | 0.2196 |
| **chr10_131901494_1319** | 1 | 1.706 | 0.1915 |
| **chr13_48222949_48273** | 2 | 4.5387 | 0.1034 |
| **chr11_5279480_537067** | 1 | 2.3162 | 0.128 |
| **chr4_190450033_19053** | 1 | 2.8384 | 0.092 |
| **chr22_32874326_33313** | 1 | 0.0078 | 0.9295 |
| **chr15_18421386_18507** | 1 | 0.5845 | 0.4445 |
| **chr1_202800228_20280** | 1 | 0.9586 | 0.3275 |
| **chr12_58840996_58859** | 2 | 0.3603 | 0.8352 |
| **chr2_16049873_161195** | 1 | 2.4199 | 0.1198 |
| **EGFR** | 1 | 0.0228 | 0.8799 |
| **PTEN** | 2 | 4.5693 | 0.1018 |
| **CDKN2C** | 2 | 1.5621 | 0.4579 |
| **CDKN1C** | 1 | 0.1441 | 0.7042 |
| **PDGFRB** | 1 | 1.0216 | 0.3121 |
| **PDGFA** | 1 | 0.1566 | 0.6923 |
| **PDGFB** | 2 | 1.3205 | 0.5167 |
| **NF1** | 2 | 1.1715 | 0.5567 |
| **TP53** | 1 | 14.1239 | 0.0002 |

J. Multivariate Cox Proportional Hazard Analysis of IDH Wild Type Grade IV Gliomas

No significant variables
